# Supplementary material for: HINGE: long-read assembly achieves optimal repeat resolution
Source: Genome Res. 2017 May;27(5):747–56. doi: 10.1101/gr.216465.116 (PMC5411769; doi:10.1101/gr.216465.116)
Supplement: Supplemental Material [file supp_27_5_747__index.html]

HINGE: long-read assembly achieves optimal repeat resolution — Supplemental Material 

# HINGE: long-read assembly achieves optimal repeat resolution

## Supplemental Material

- Supplemental\_Table\_S3.xlsx
- Supplemental\_Table\_S4.pdf
- Supplemental\_Source\_Code.zip
- Supplemental\_Material.pdf
