# Supplementary material for: HINGE: long-read assembly achieves optimal repeat resolution
Source: Genome Res. 2017 May;27(5):747–56. doi: 10.1101/gr.216465.116 (PMC5411769; doi:10.1101/gr.216465.116)
Supplement: Supplemental Material [file supp_gr.216465.116_Supplemental_Table_S4.pdf]

Performance of HINGE on NCTC 3000

In case of any questions or concerns please contact Govinda Kamath (gkamath@stanford.edu), Fei Xia (feixia@stanford.edu), and Ilan Shomorony (ilan.shomorony@berkeley.edu).

ID	Name	Coverage Depth	NCTC Pipeline			HINGE		Miniasm
			#Chromosome Contigs	#Plasmid Contigs	#Unidentified Contigs	Visualisation	Classification	
NCTC74	*Salmonella enterica subsp. enterica serovar Typhimurium*	47	1	1	0	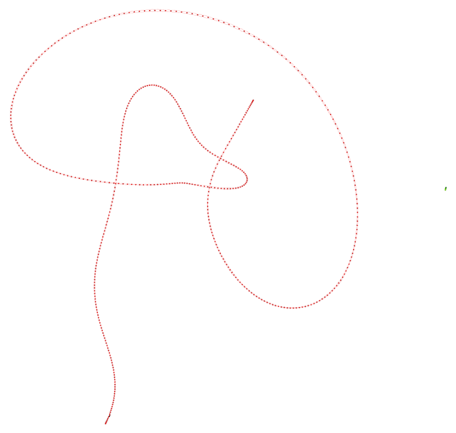	Finished assembly (lacking circularisation)	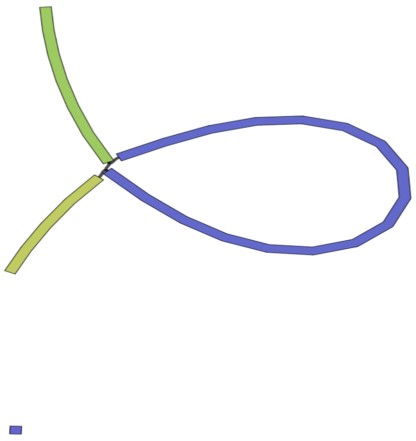
NCTC86	*Escherichia coli*	87	1	0	0	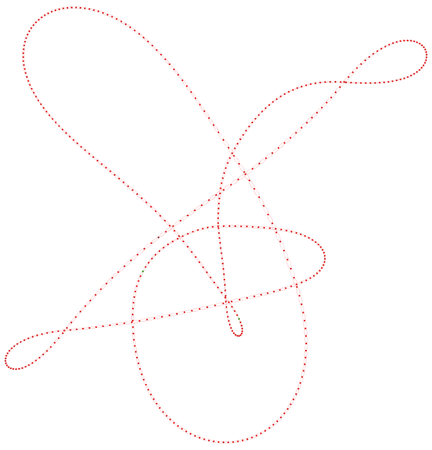	Finished circular assembly	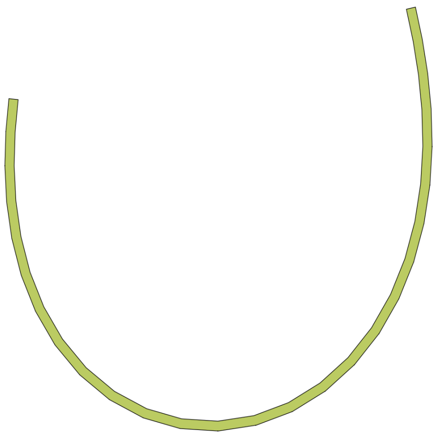
NCTC92	*Salmonella enterica subsp. enterica*	75	1	0	1	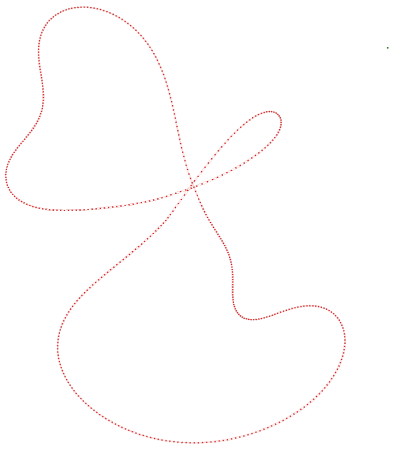	Finished circular assembly	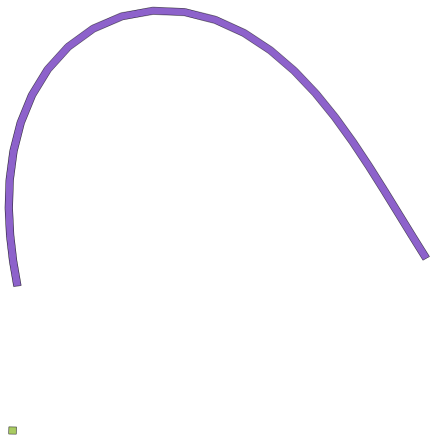
NCTC129	*Salmonella enterica subsp. enterica*	68	1	0	0	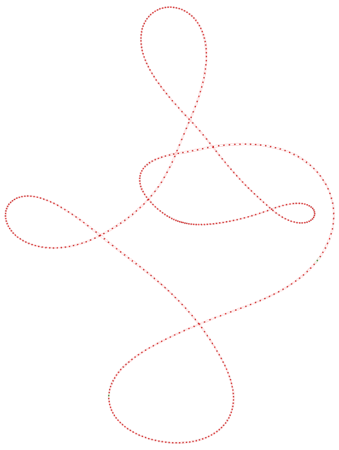	Finished circular assembly	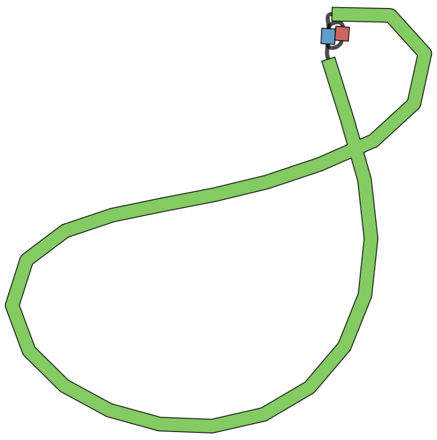
NCTC204	*Klebsiella pneumoniae*	20	0	0	4	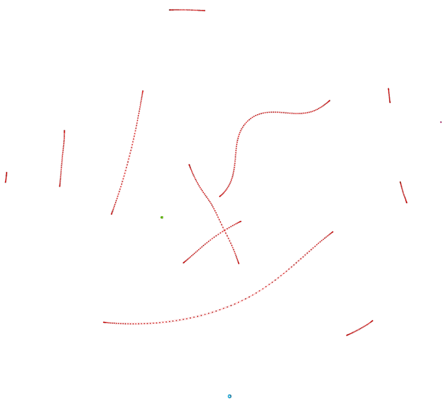	Mis-assembly/Fragmented	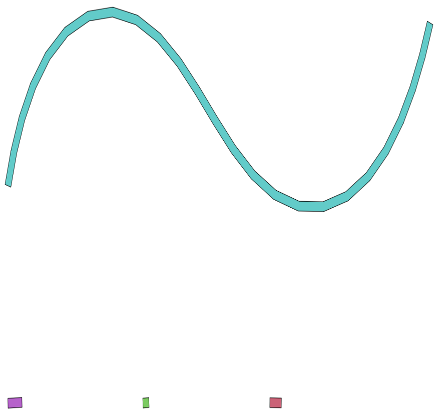
NCTC235	*Morganella morganii*	73	1	0	0		Finished circular assembly	

						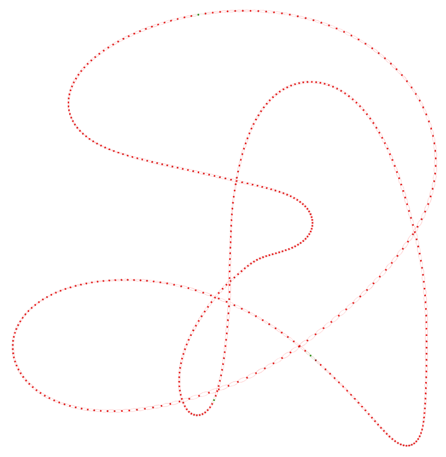		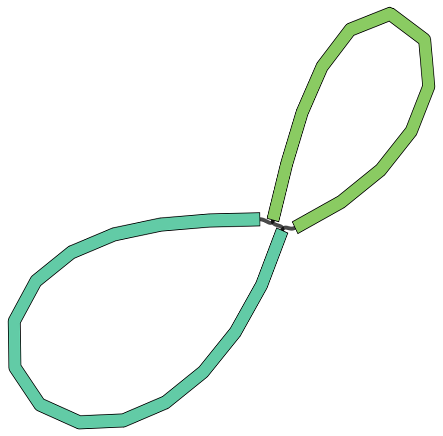
NCTC418	*Enterobacter aerogenes*	56	Pending	Pending	Pending	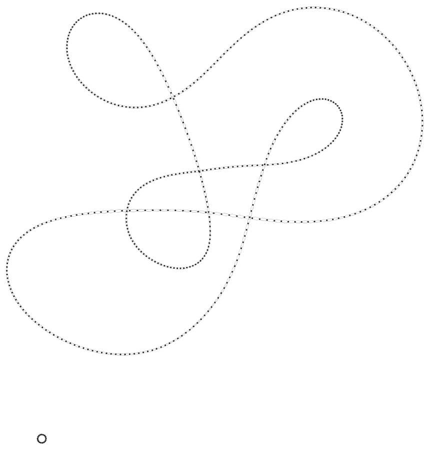	Finished circular assembly	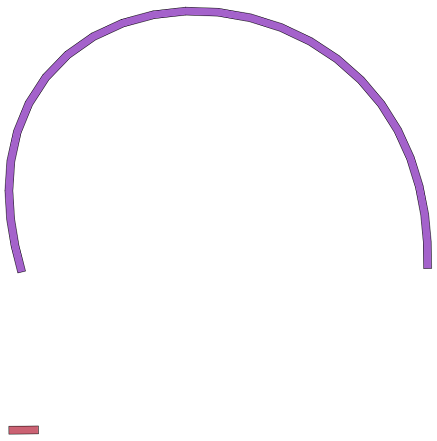
NCTC1080	*Streptococcus sp.*	170	1	0	1	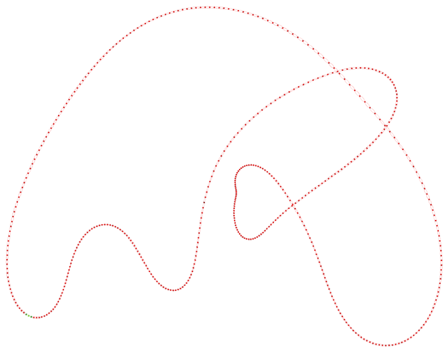	Finished circular assembly	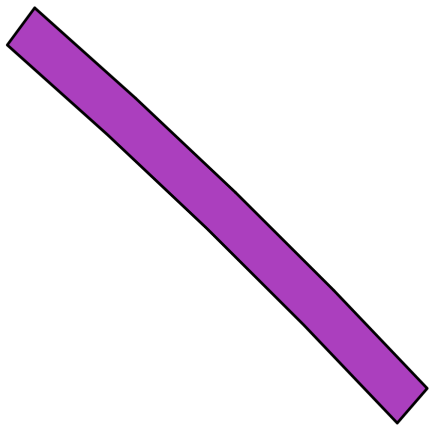
NCTC1936	*Klebsiella pneumoniae*	64	1	1	0	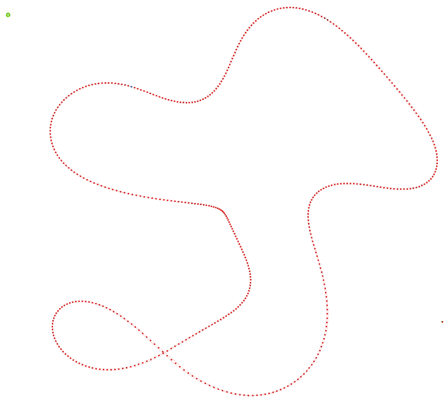	Finished circular assembly	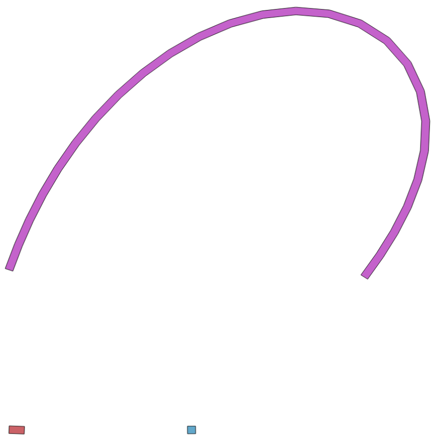
NCTC2218	*Streptococcus pyogenes*	119	1	0	1	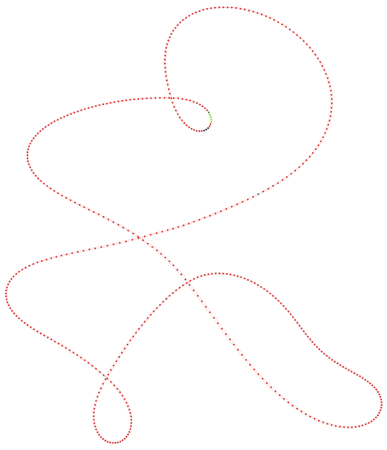	Finished circular assembly	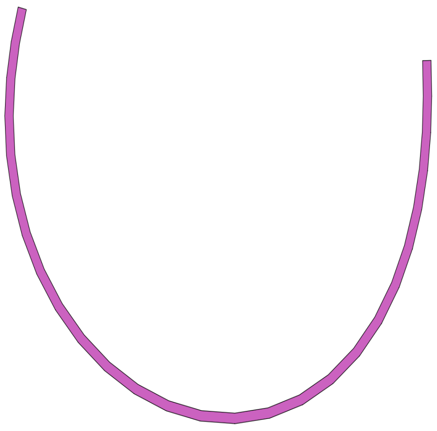
NCTC2366	*Streptococcus pyogenes*	150	Pending	Pending	Pending	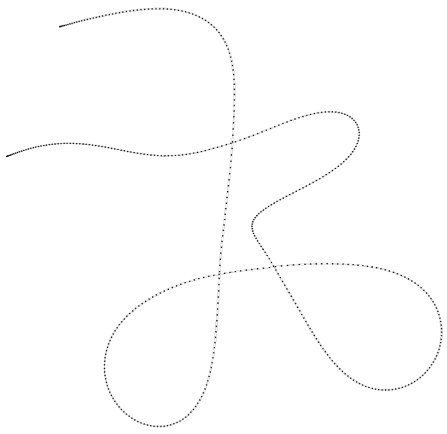	Finished assembly (lacking circularisation)	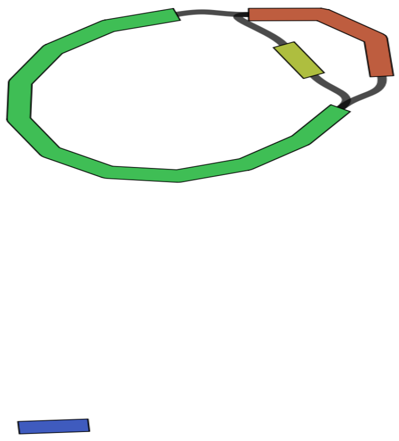
NCTC2669	*Staphylococcus aureus*	162	1	1	0		Finished circular assembly	

						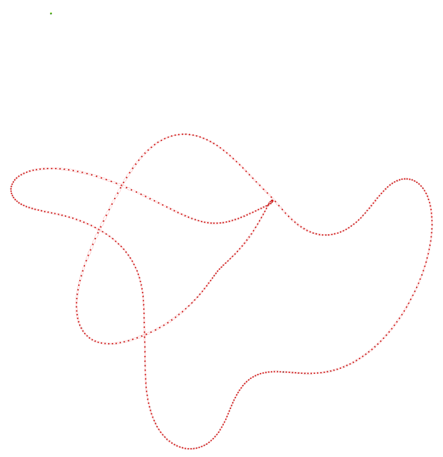		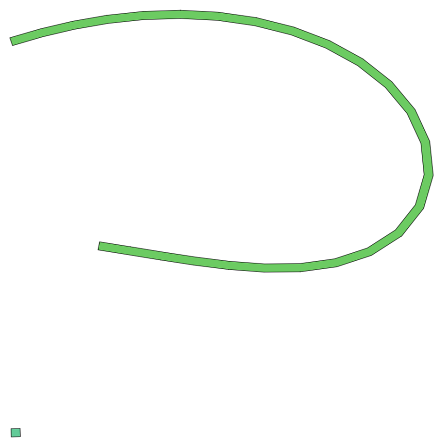
NCTC3046	*Salmonella sp.*	71	1	1	0	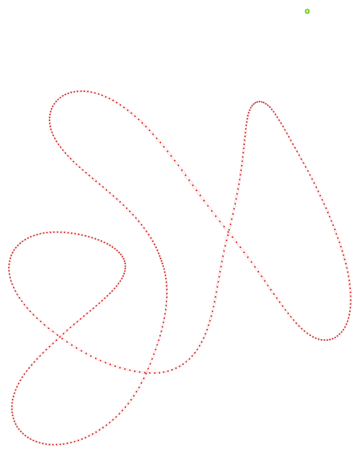	Finished circular assembly	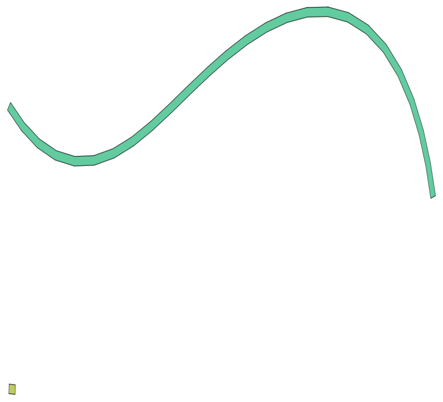
NCTC3166	*Streptococcus viridans*	167	Pending	Pending	Pending	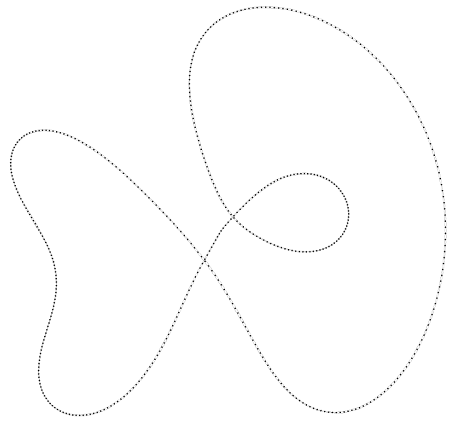	Finished circular assembly	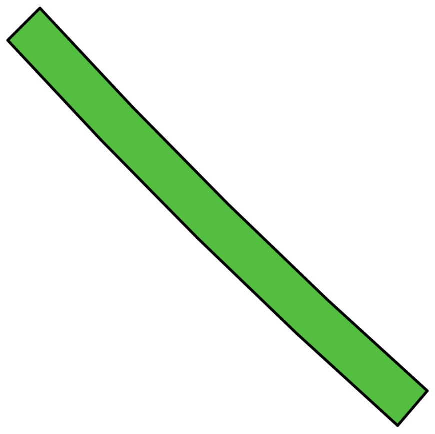
NCTC3168	*Streptococcus sanguinis*	189	Pending	Pending	Pending	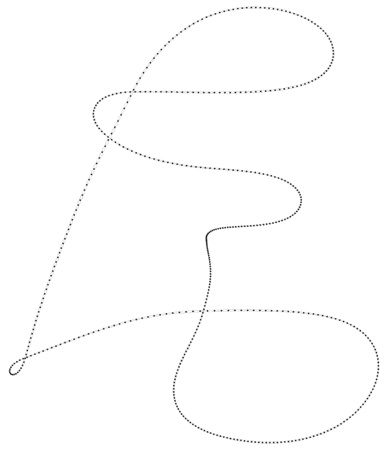	Finished circular assembly	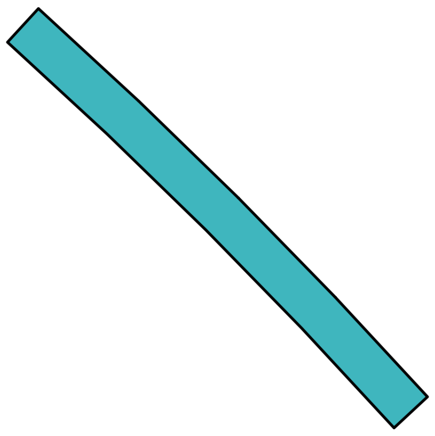
NCTC3438	*Avibacterium volantium*	136	Pending	Pending	Pending	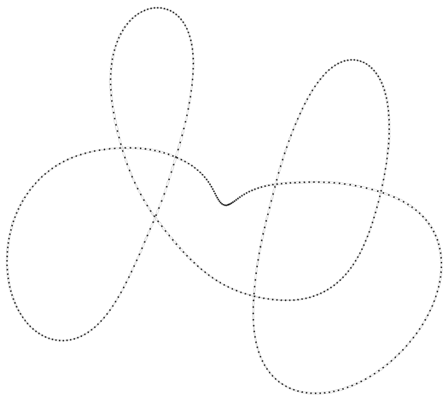	Finished circular assembly	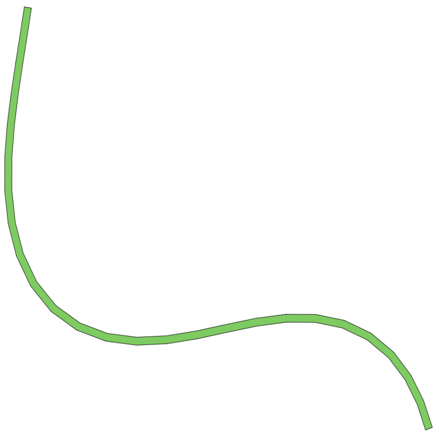
NCTC3750	*Staphylococcus aureus*	117	0	0	3	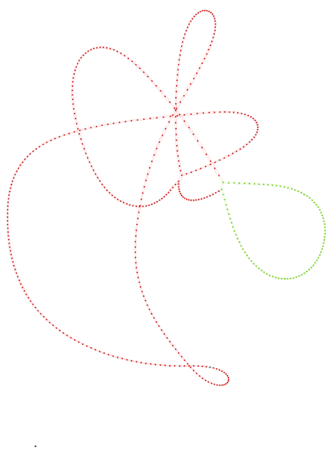	Finished assembly with multiple traversals	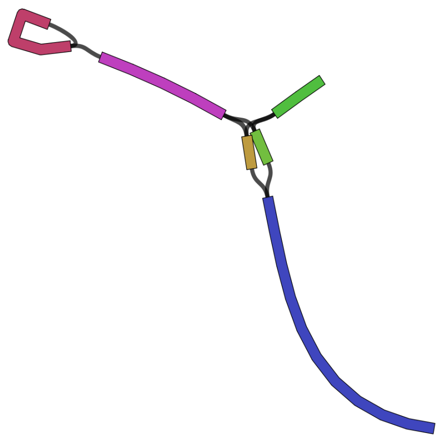
NCTC3761	*Staphylococcus aureus*	115	1	0	0		Finished circular assembly	

						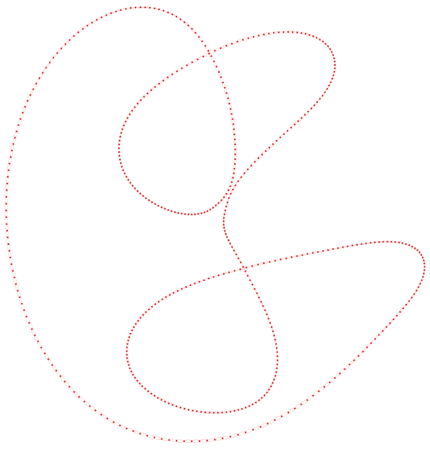		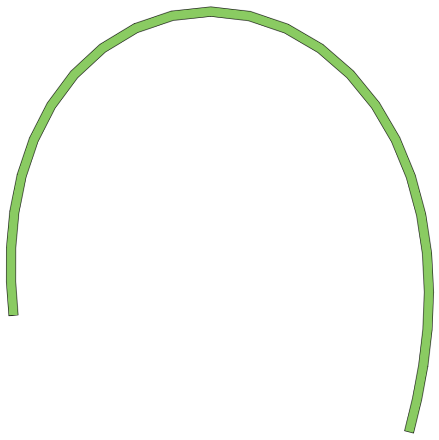
NCTC4001	*Streptococcus pyogenes*	108	1	0	0	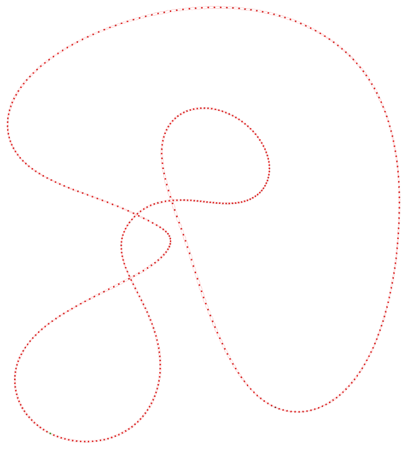	Finished circular assembly	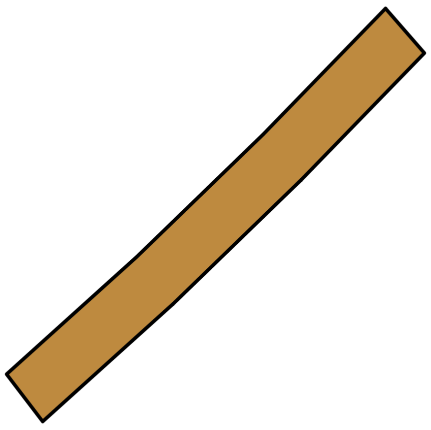
NCTC4133	*Staphylococcus epidermidis*	159	1	0	0	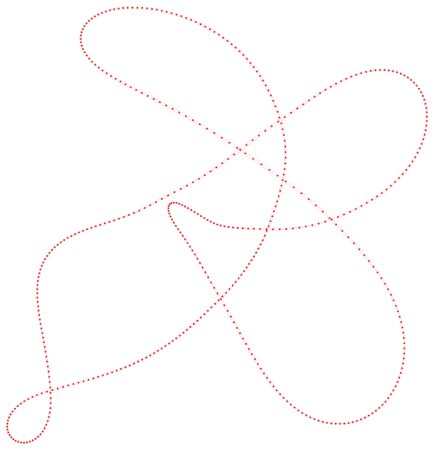	Finished circular assembly	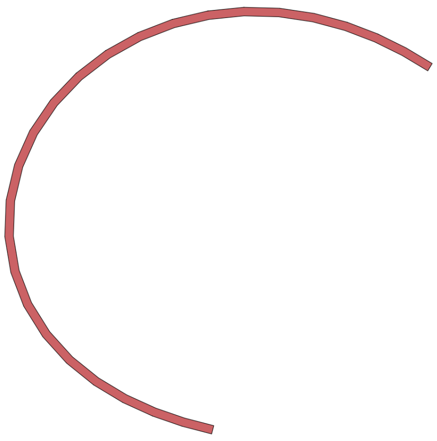
NCTC4136	*Staphylococcus aureus*	108	1	1	0	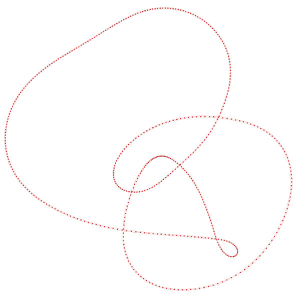	Finished circular assembly	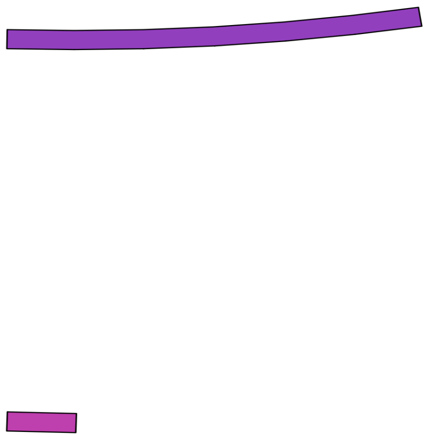
NCTC4137	*Staphylococcus aureus*	102	1	0	0	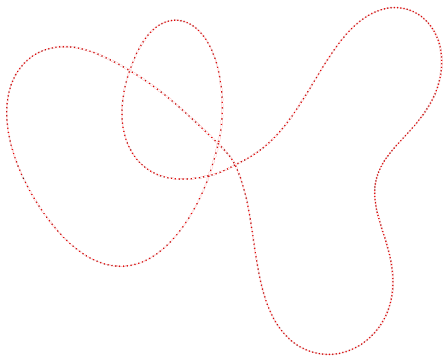	Finished circular assembly	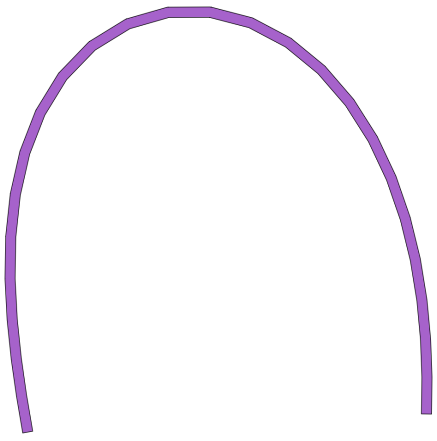
NCTC4163	*Staphylococcus aureus*	92	1	0	0	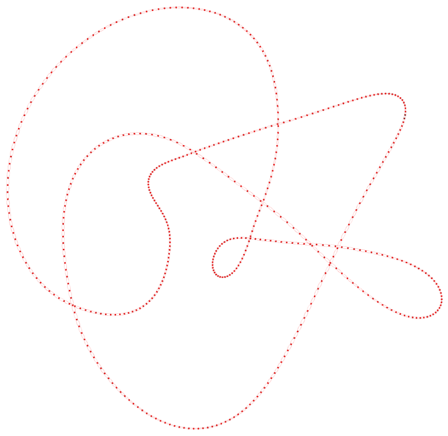	Finished circular assembly	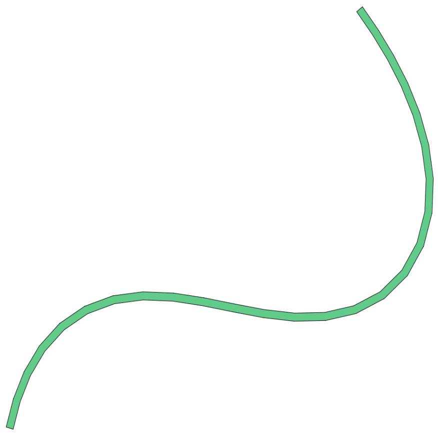
NCTC4168	*Escherichia dispar*	43	1	0	5		Finished assembly with multiple traversals	

						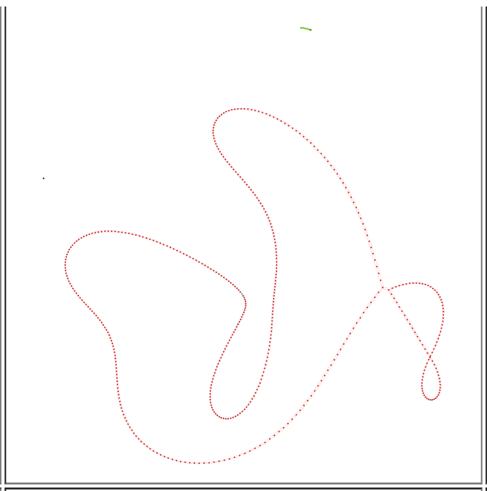		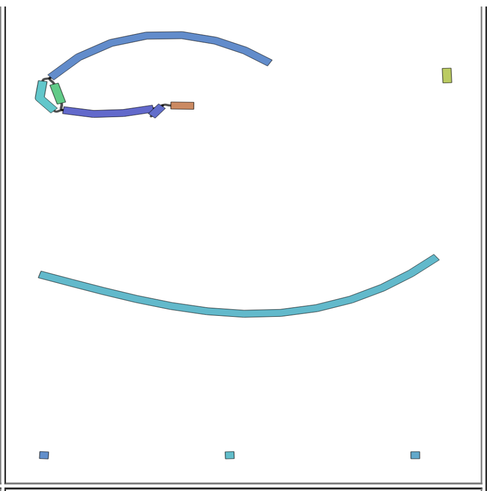
NCTC4169	*Escherichia coli*	66	0	0	6	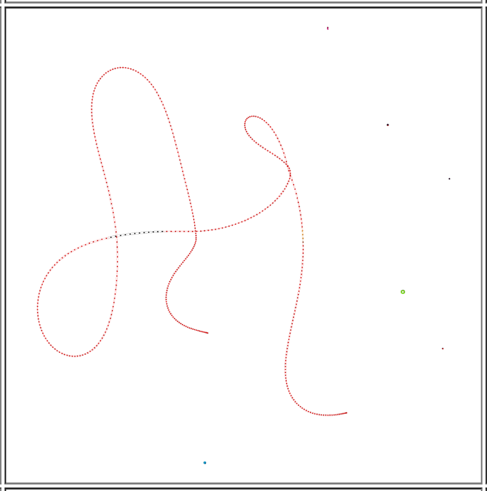	Finished assembly (lacking circularisation)	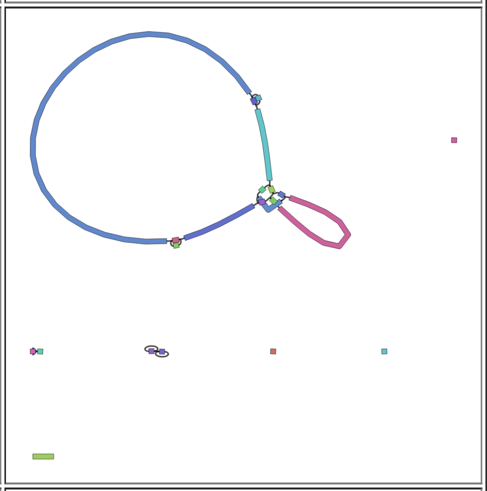
NCTC4174	*Escherichia coli*	36	Pending	Pending	Pending	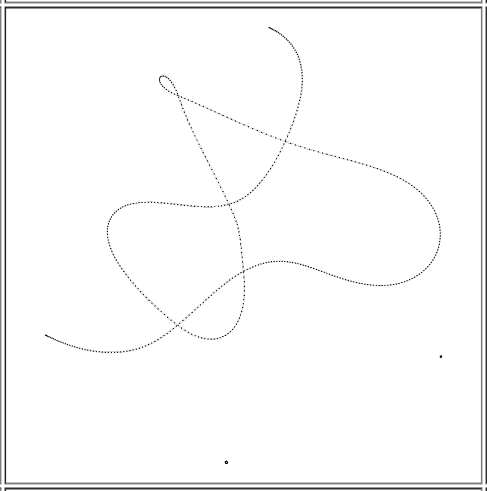	Finished assembly (lacking circularisation)	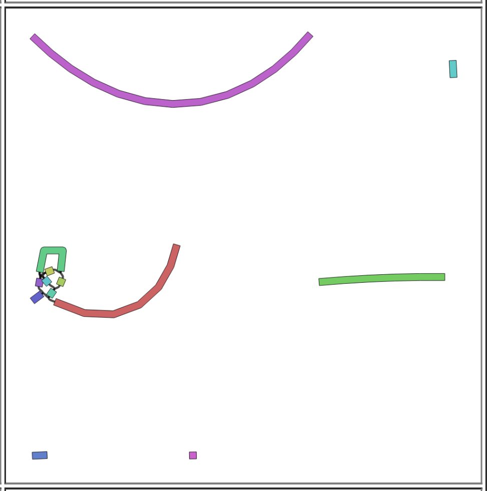
NCTC4199	*Proteus mirabilis*	69	1	0	0	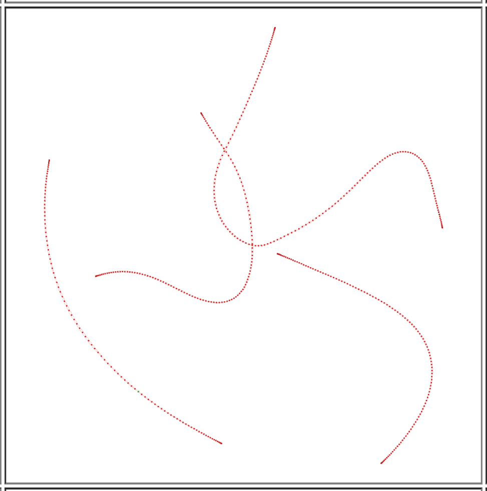	Mis-assembly/Fragmented	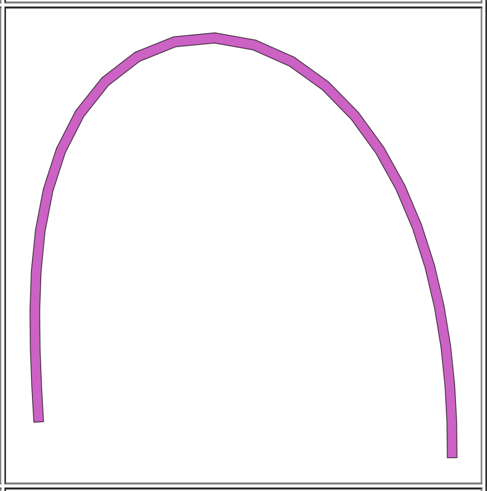
NCTC4444	*Salmonella enterica subsp. enterica*	35	1	1	0	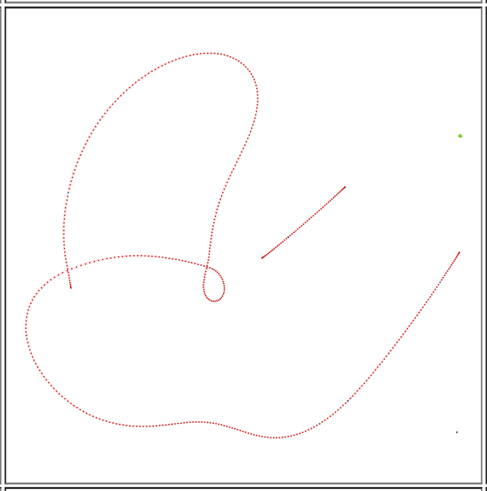	Mis-assembly/Fragmented	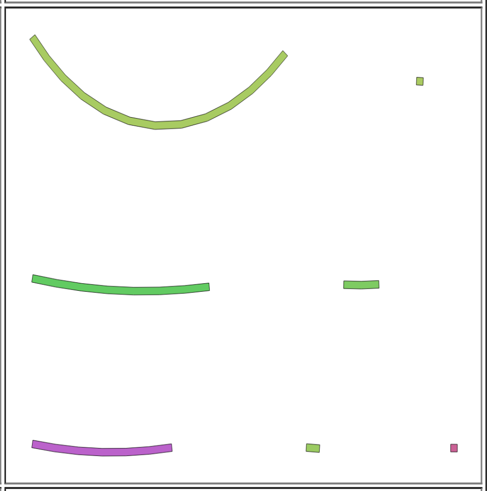
NCTC4450	*Escherichia coli*	52	0	0	159	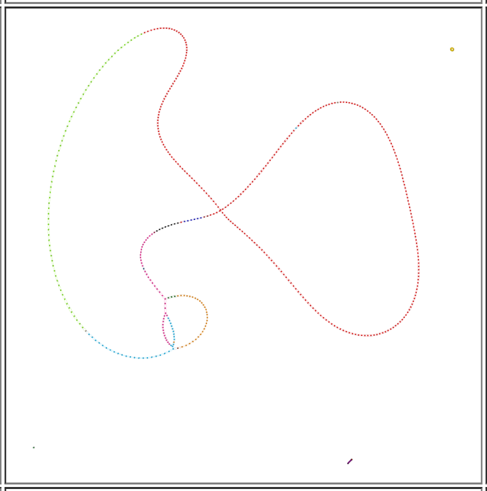	Finished assembly with multiple traversals	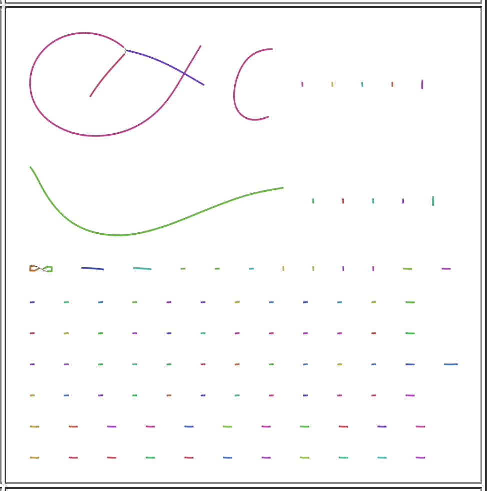
NCTC4669	*Streptococcus dysgalactiae*	51	Pending	Pending	Pending		Finished circular assembly	

						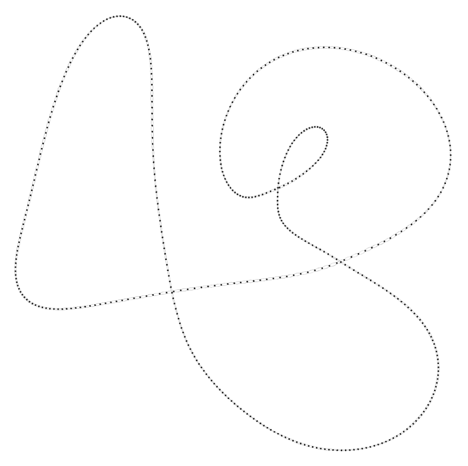		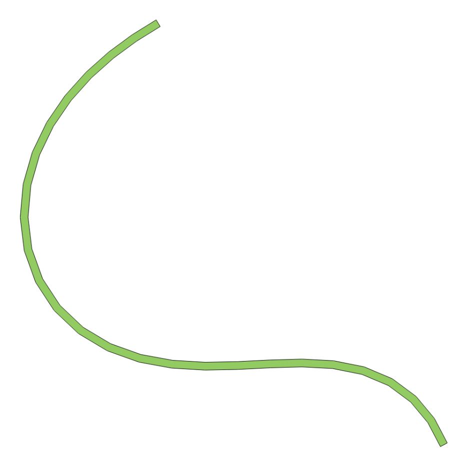
NCTC4671	*Streptococcus dysgalactiae*	103	1	0	2	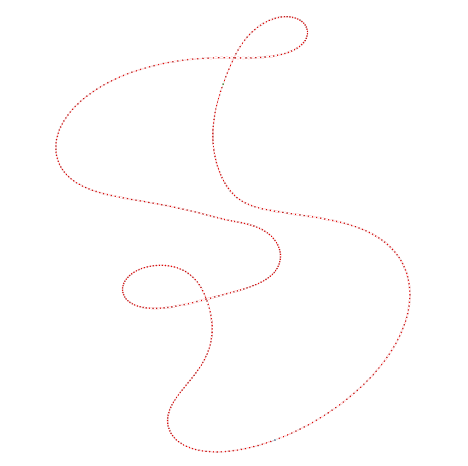	Finished circular assembly	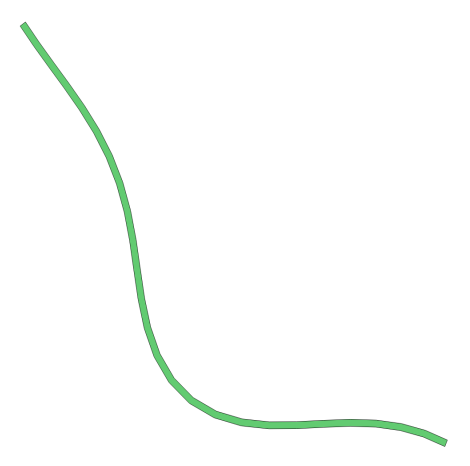
NCTC4672	*Streptococcus uberis*	10	0	0	3	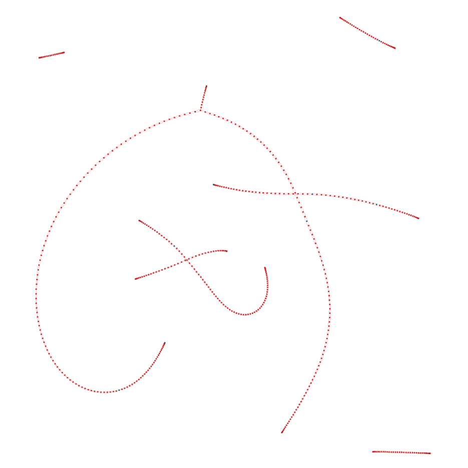	Mis-assembly/Fragmented	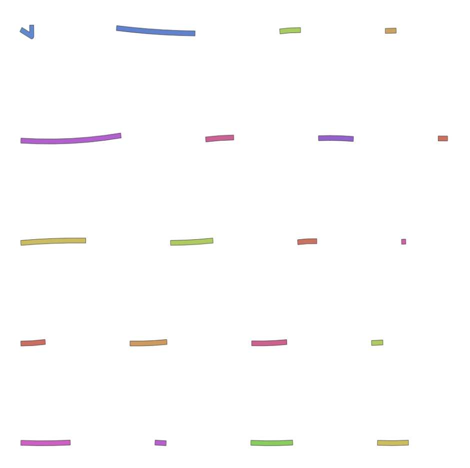
NCTC4673	*Streptococcus uberis*	123	1	0	2	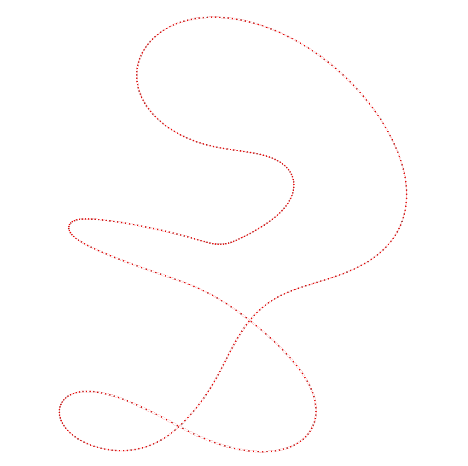	Finished circular assembly	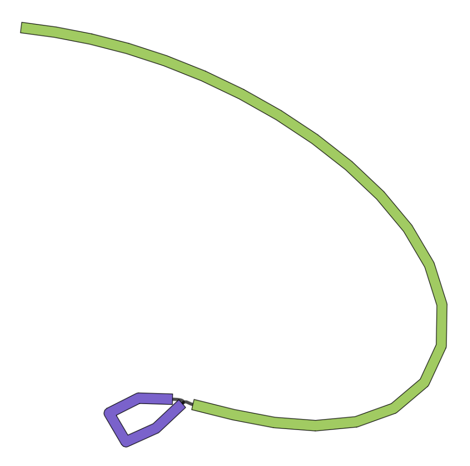
NCTC4675	*Streptococcus sp*	139	1	0	0	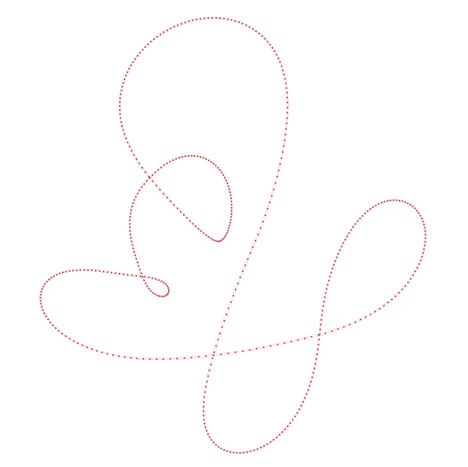	Finished circular assembly	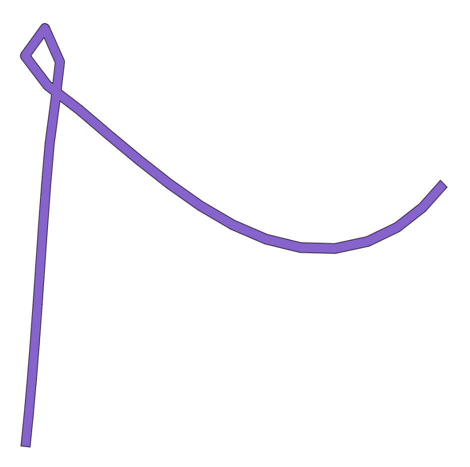
NCTC4725	*Streptococcus sp.*	61	Pending	Pending	Pending	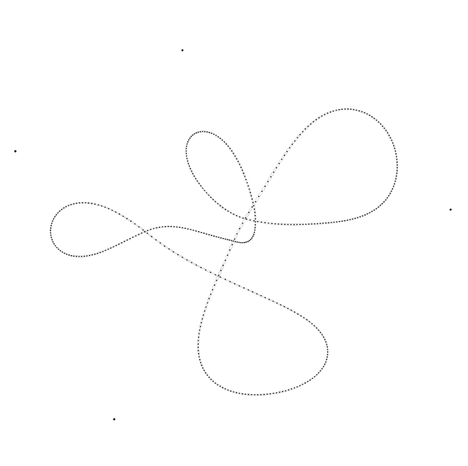	Finished circular assembly	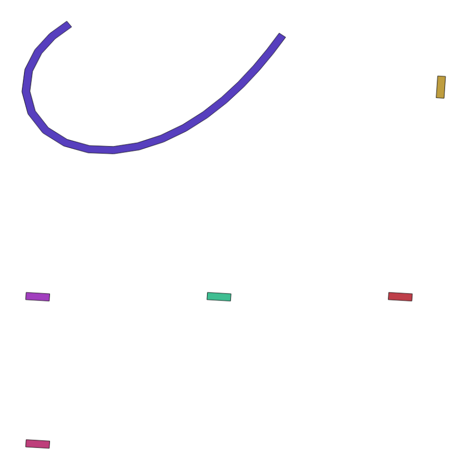
NCTC4840	*Salmonella enterica subsp. enterica*	43	1	0	0		Finished circular assembly	

						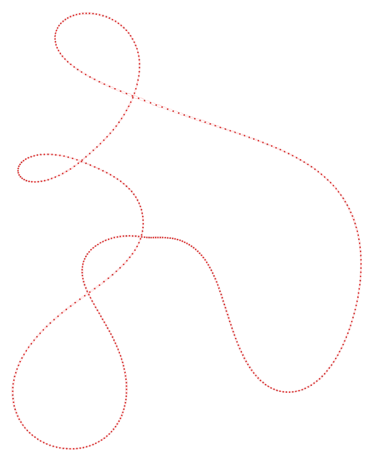		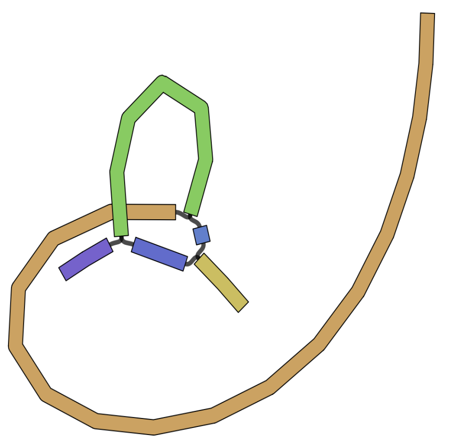
NCTC5046	*Klebsiella pneumoniae subsp. rhinoscleromatis*	65	1	1	0	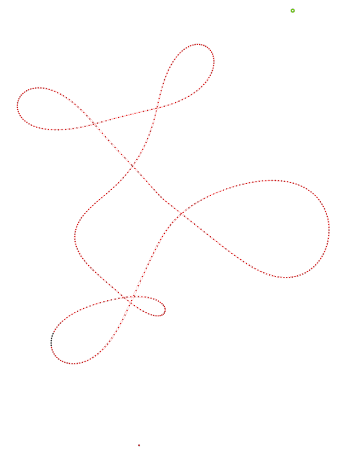	Finished circular assembly	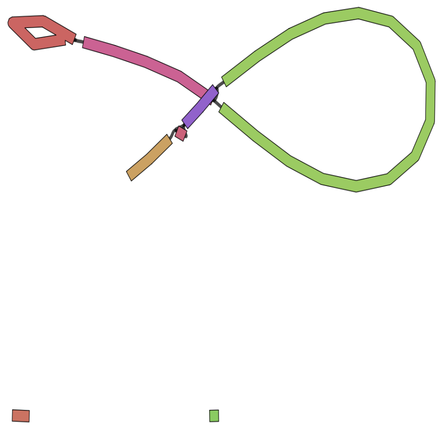
NCTC5047	*Klebsiella pneumoniae*	21	0	0	6	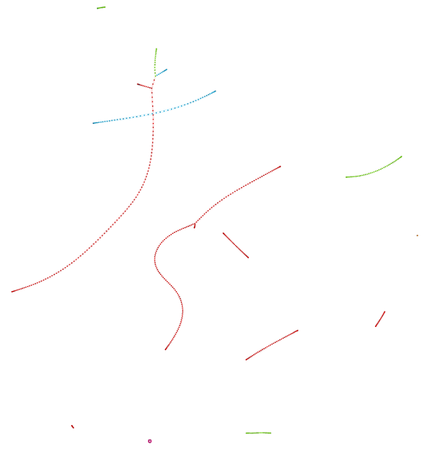	Mis-assembly/Fragmented	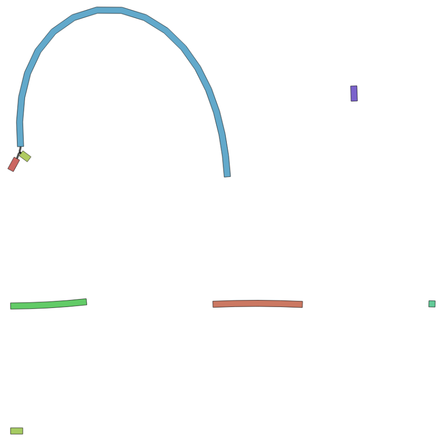
NCTC5050	*Klebsiella pneumoniae subsp. ozaenae*	54	2	3	0	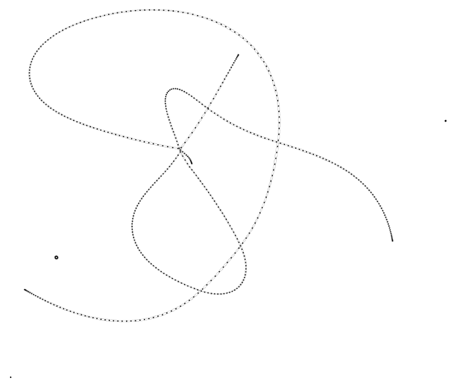	Mis-assembly/Fragmented	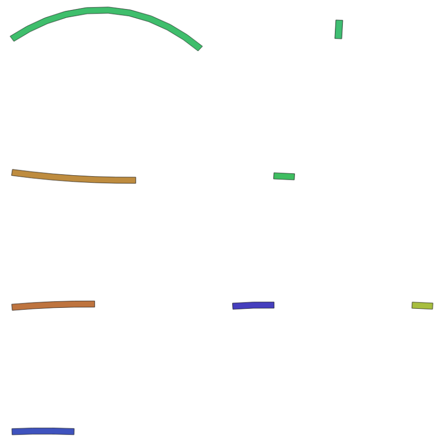
NCTC5051	*Klebsiella pneumoniae*	53	1	3	1	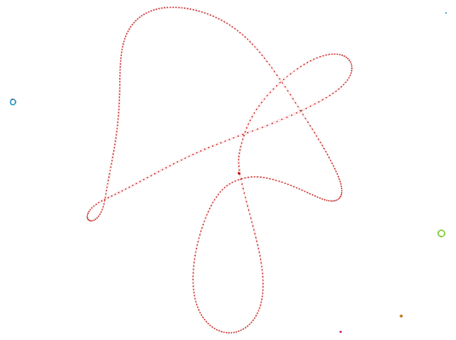	Finished circular assembly	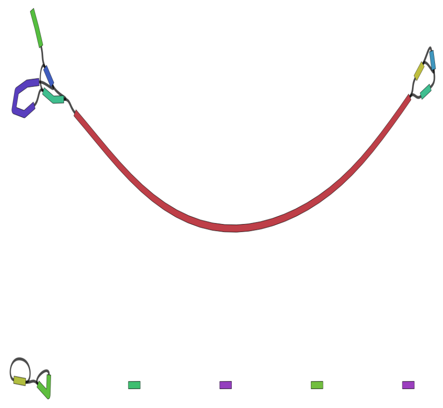
NCTC5052	*Klebsiella pneumoniae*	69	0	0	8	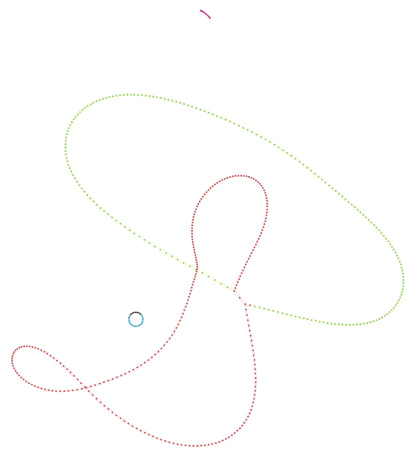	Finished assembly with multiple traversals	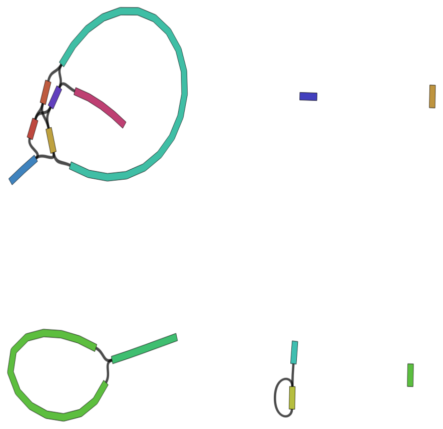
NCTC5053	*Klebsiella pneumoniae*	28	0	0	7		Mis-assembly/Fragmented	

						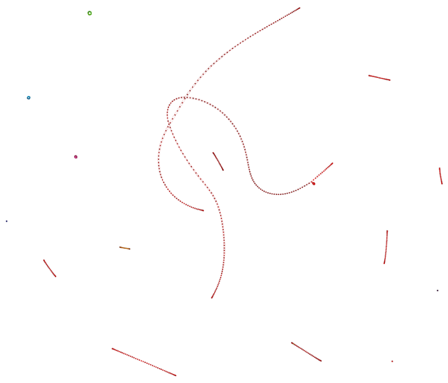		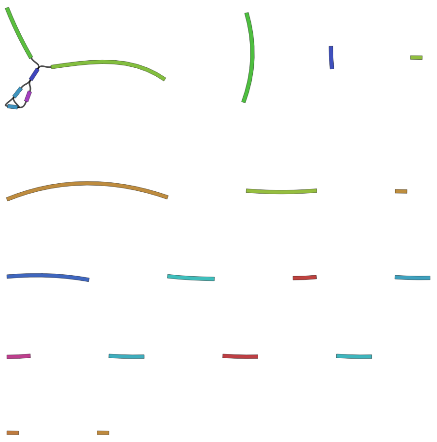
NCTC5054	*Klebsiella sp.*	67	1	3	1	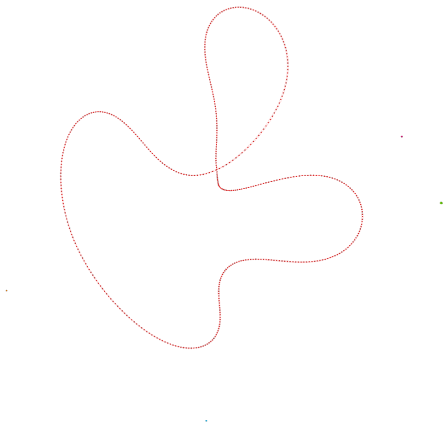	Finished circular assembly	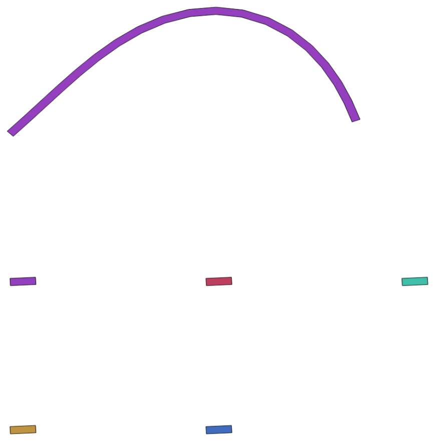
NCTC5055	*Klebsiella pneumoniae*	69	1	0	2	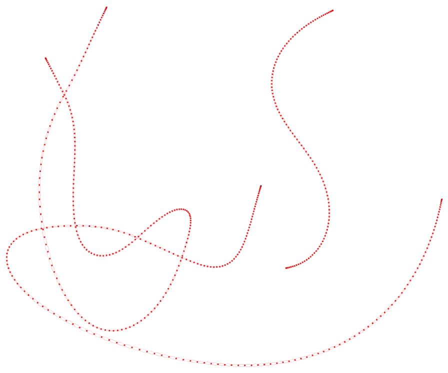	Mis-assembly/Fragmented	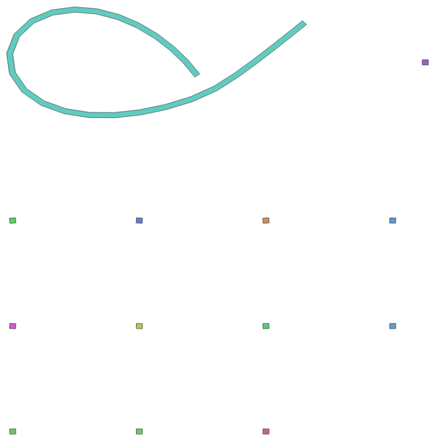
NCTC5056	*Klebsiella pneumoniae*	62	1	3	2	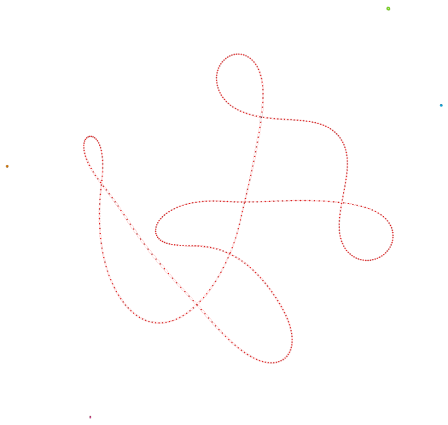	Finished circular assembly	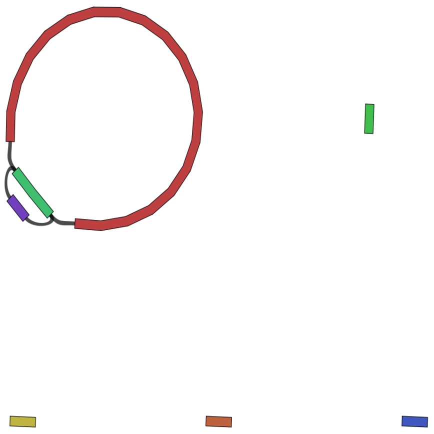
NCTC5057	*Klebsiella sp.*	68	0	0	2	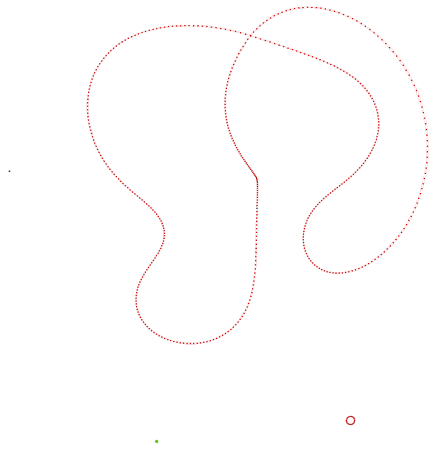	Finished circular assembly	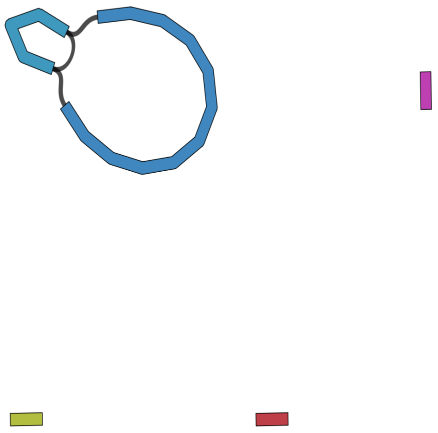
NCTC5163	*Streptococcus pyogenes*	118	1	0	0	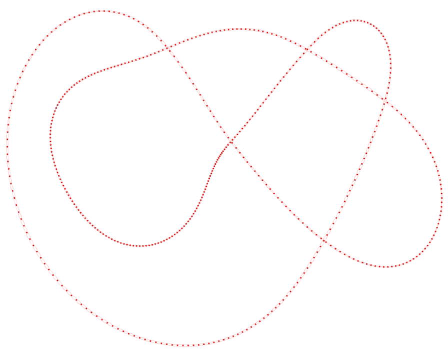	Finished circular assembly	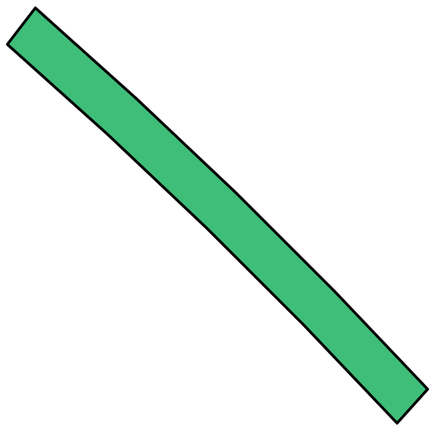
NCTC5164	*Streptococcus pyogenes*	199	1	0	0		Finished circular assembly	

						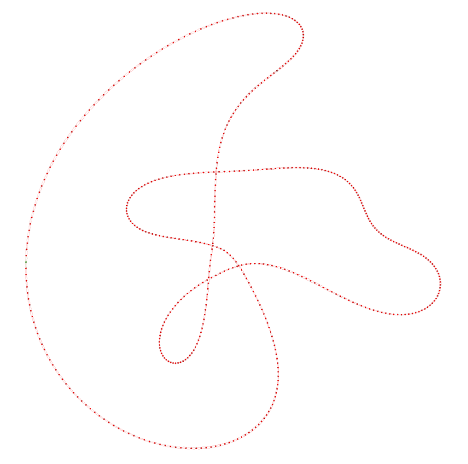		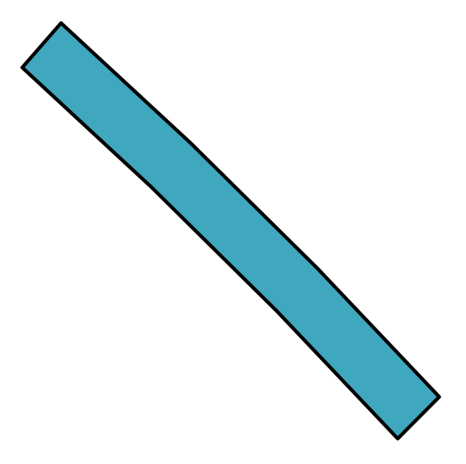
NCTC5338	*Streptococcus sp.*	204	Pending	Pending	Pending	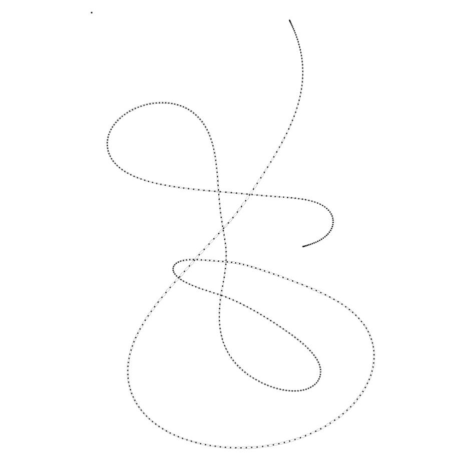	Finished assembly (lacking circularisation)	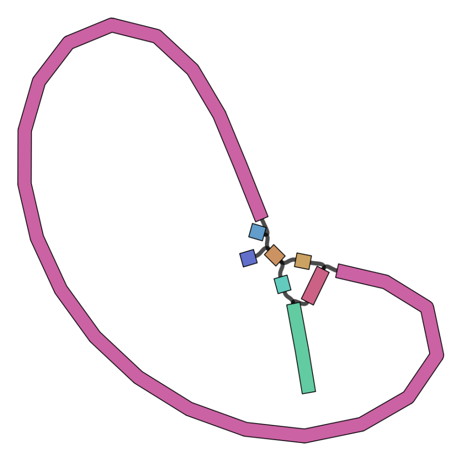
NCTC5370	*Streptococcus dysgalactiae subsp. equisimilis*	125	1	0	0	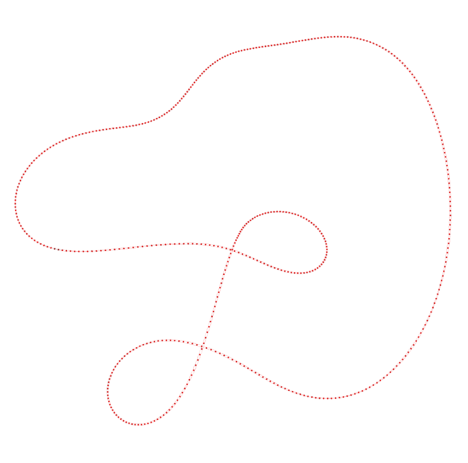	Finished circular assembly	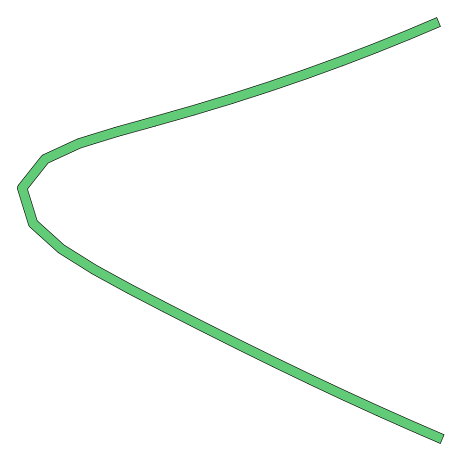
NCTC5371	*Streptococcus dysgalactiae subsp. equisimilis*	140	Pending	Pending	Pending	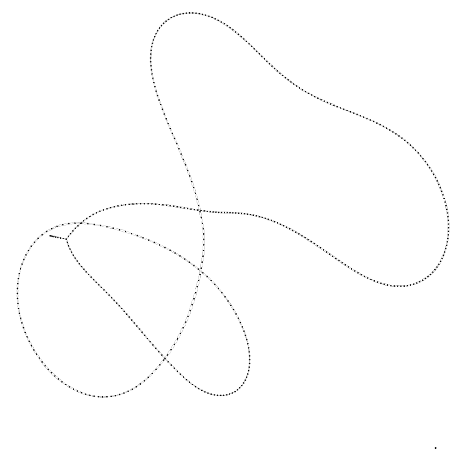	Finished circular assembly	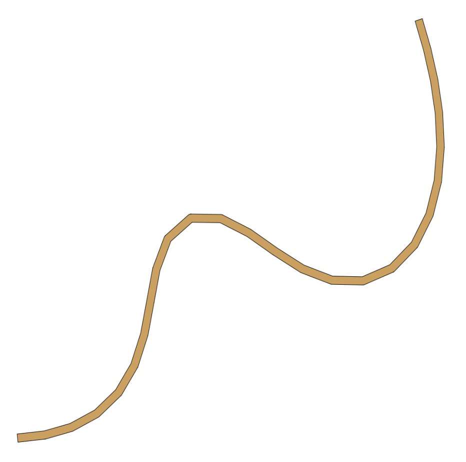
NCTC5385	*Streptococcus sp.*	156	1	0	0	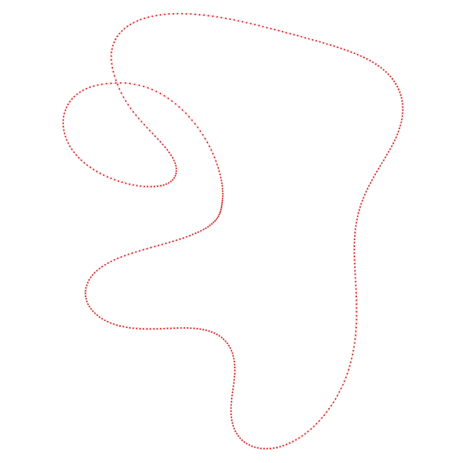	Finished circular assembly	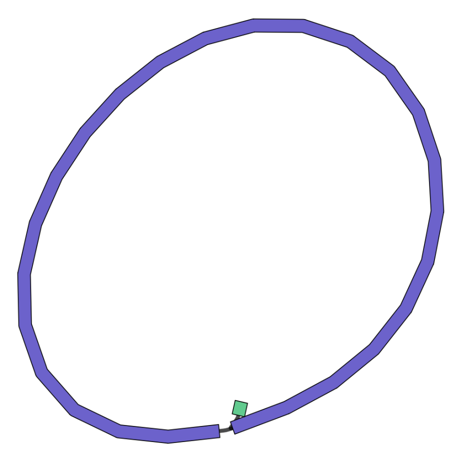
NCTC5386	*Streptococcus sp.*	129	1	0	2	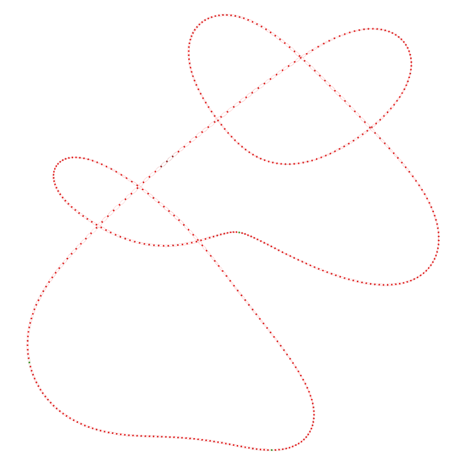	Finished circular assembly	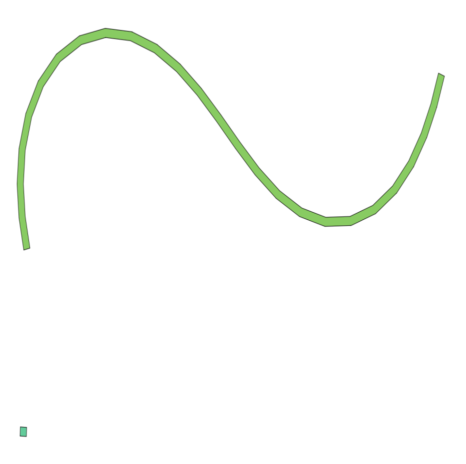
NCTC5655	*Staphylococcus aureus*	75	0	0	16		Finished circular assembly	

						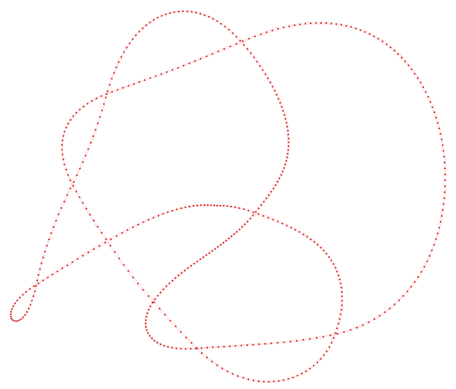		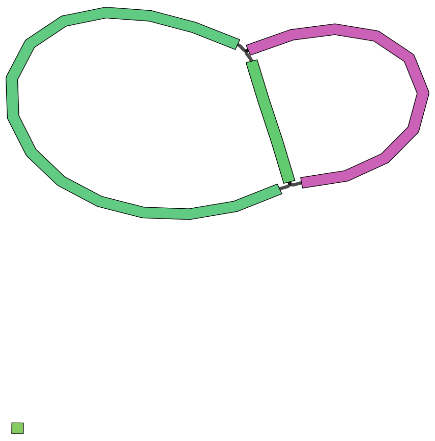
NCTC5656	*Staphylococcus aureus*	90	0	0	2	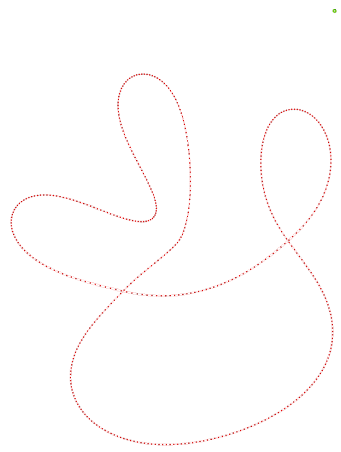	Finished circular assembly	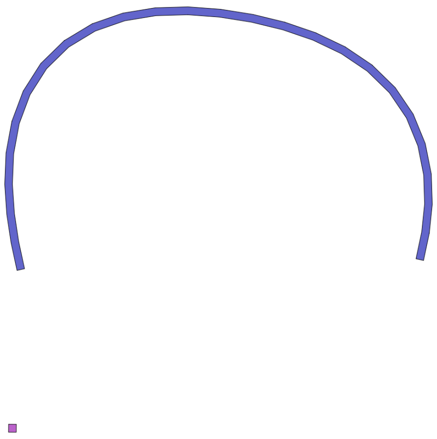
NCTC5657	*Staphylococcus aureus*	49	1	1	0	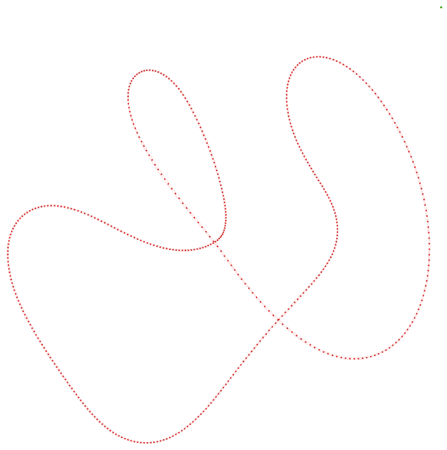	Finished circular assembly	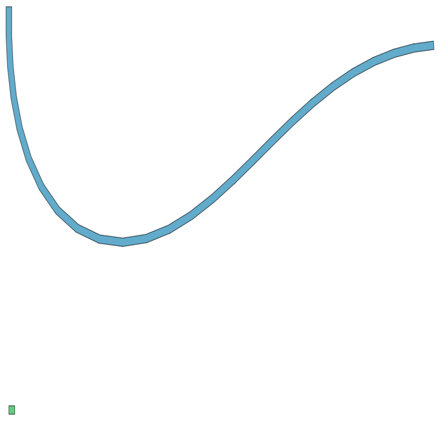
NCTC5658	*Staphylococcus aureus*	157	1	0	1	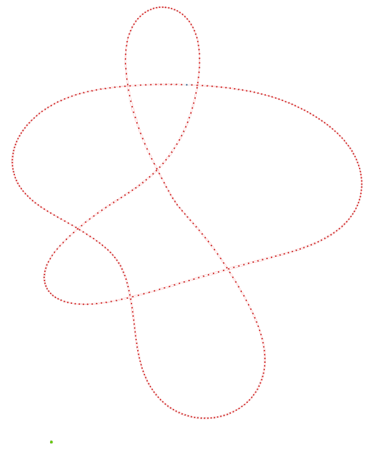	Finished circular assembly	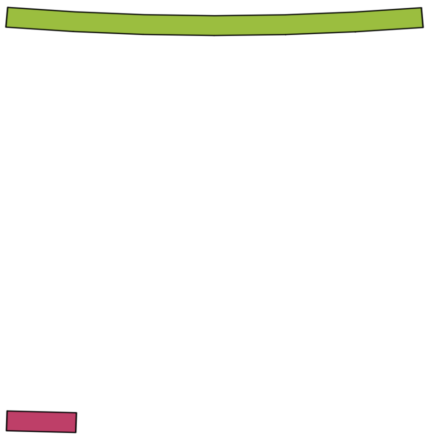
NCTC5660	*Staphylococcus aureus*	45	1	0	0	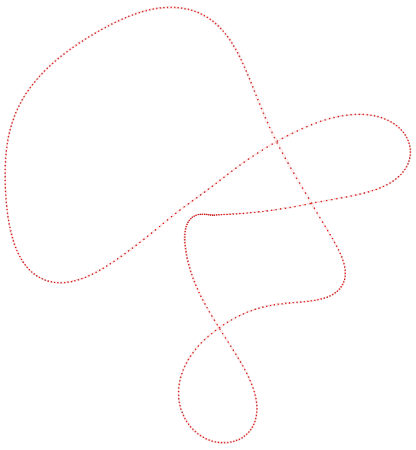	Finished circular assembly	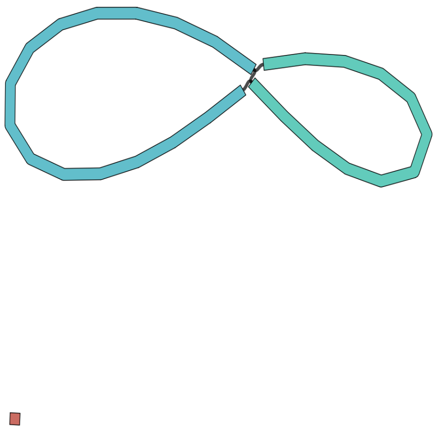
NCTC5661	*Staphylococcus aureus*	142	1	0	0	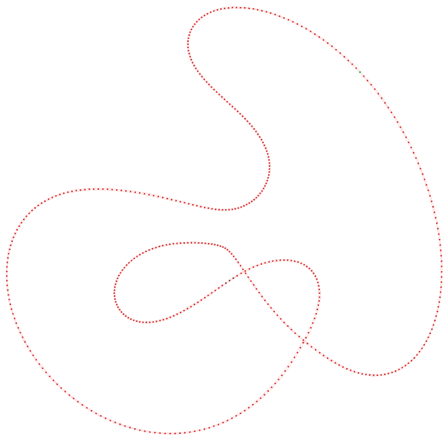	Finished circular assembly	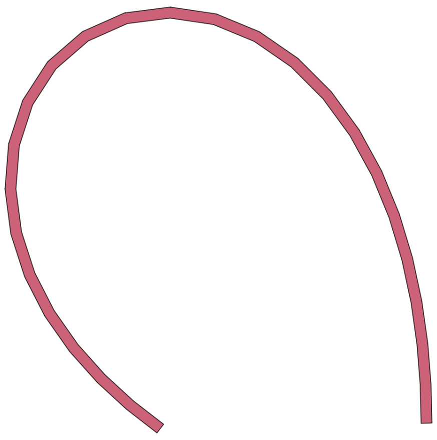
NCTC5663	*Staphylococcus aureus*	106	1	0	0		Finished circular assembly	

						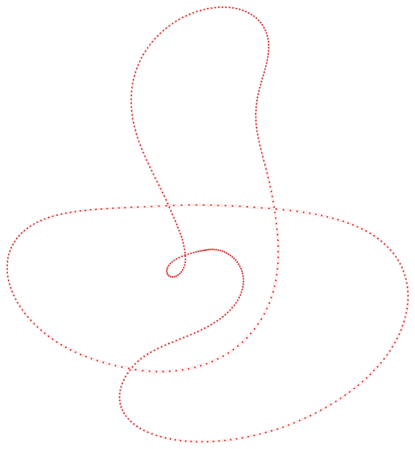		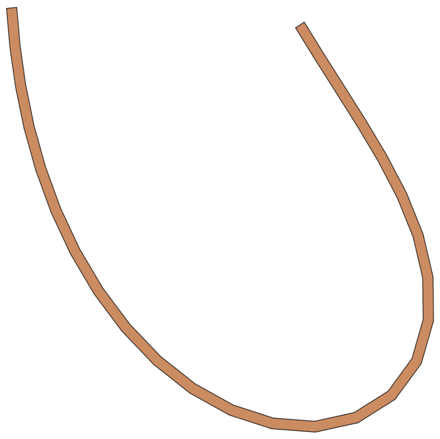
NCTC5664	*Staphylococcus aureus*	21	0	0	4	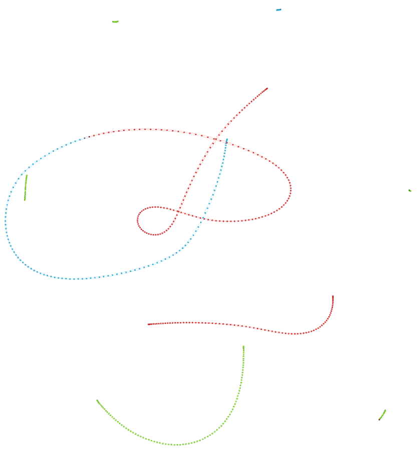	Mis-assembly/Fragmented	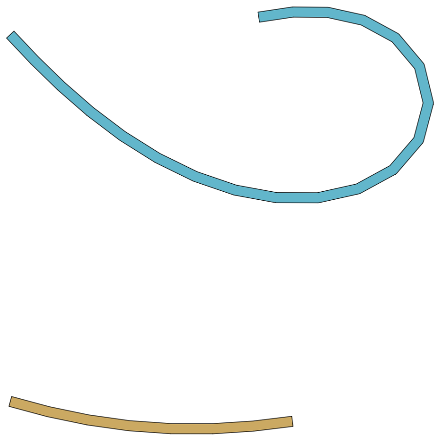
NCTC5706	*Salmonella enterica subsp. enterica serovar Java*	69	1	0	0	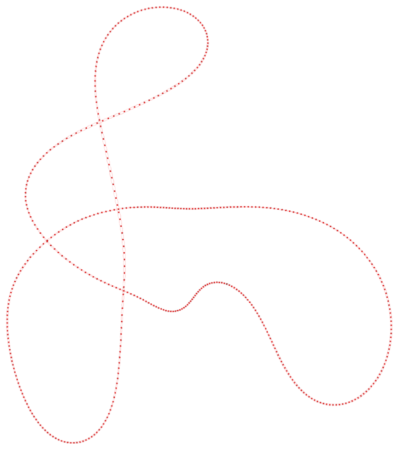	Finished circular assembly	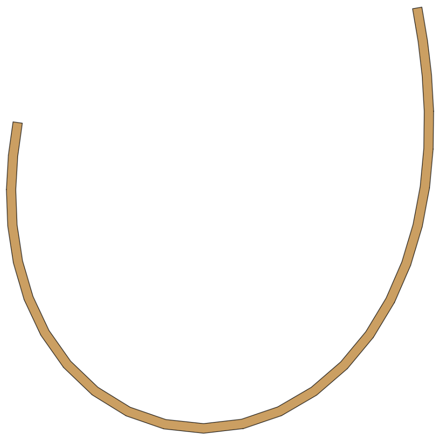
NCTC5713	*Salmonella enterica subsp. enterica*	74	1	1	0	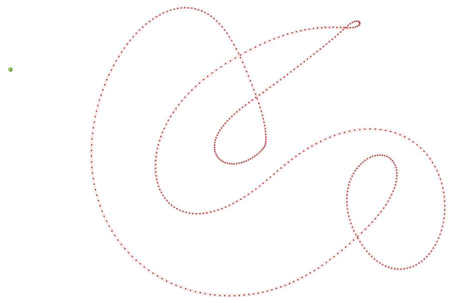	Finished circular assembly	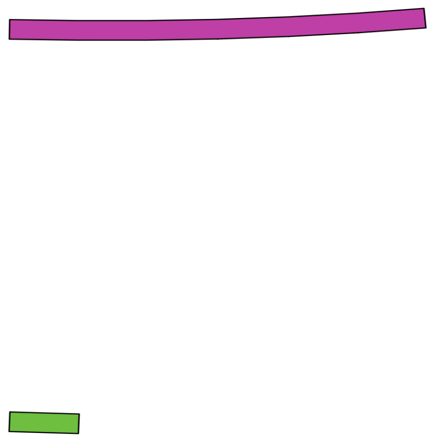
NCTC5717	*Salmonella enterica*	56	1	0	0	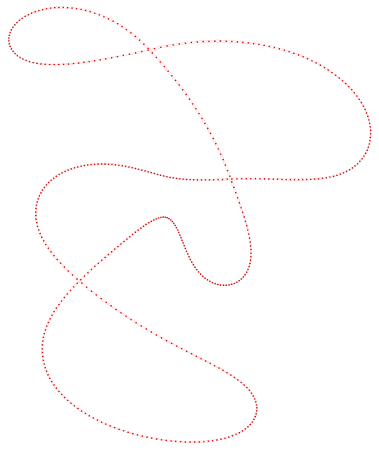	Finished circular assembly	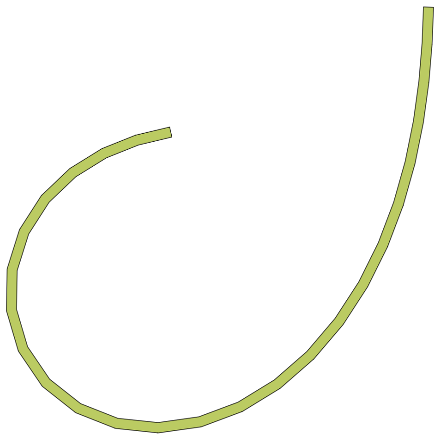
NCTC5722	*Salmonella sp.*	59	1	0	0	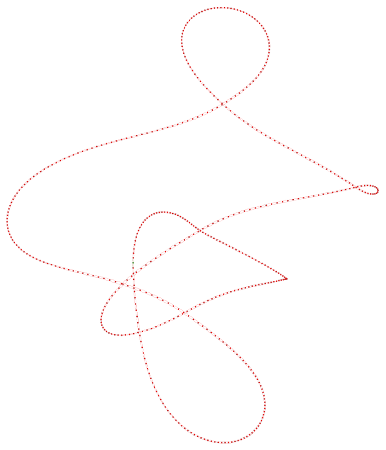	Finished circular assembly	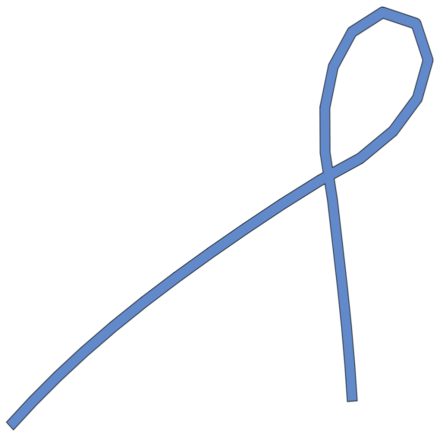
NCTC5741	*Salmonella enterica subsp. enterica*	84	0	0	1		Finished circular assembly	

						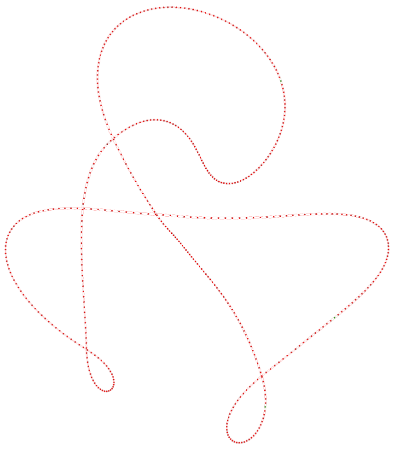		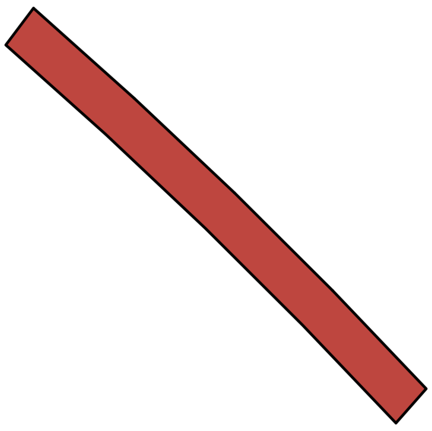
NCTC5742	*Salmonella enterica subsp. enterica*	82	1	1	0	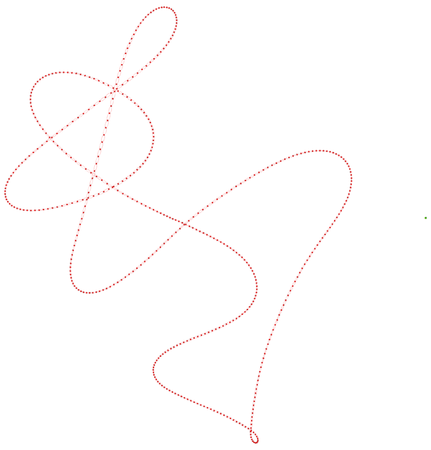	Finished circular assembly	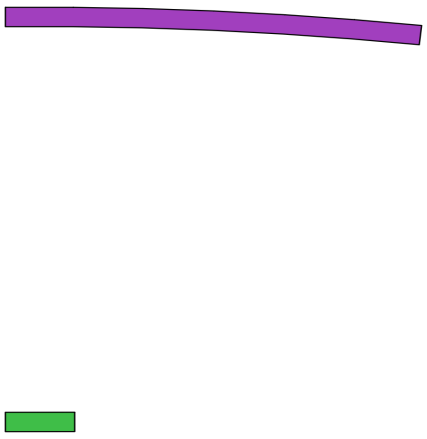
NCTC5743	*Salmonella enterica subsp. enterica serovar Oranienburg*	79	1	0	1	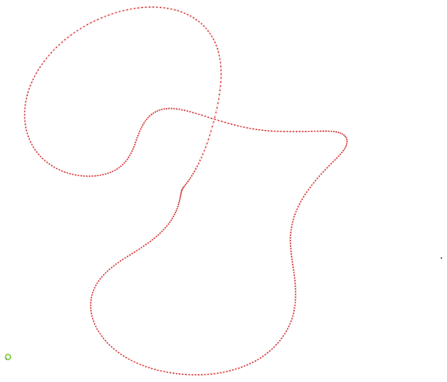	Finished circular assembly	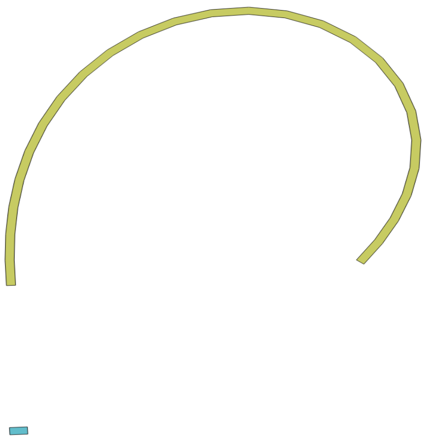
NCTC5750	*Salmonella enterica subsp. enterica serovar Braenderup*	55	0	0	3	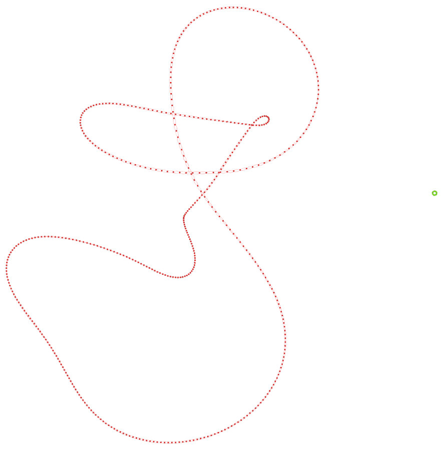	Finished circular assembly	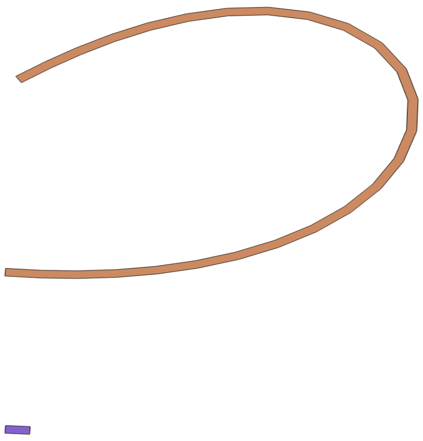
NCTC5773	*Salmonella enterica subsp. salamae*	29	1	0	0	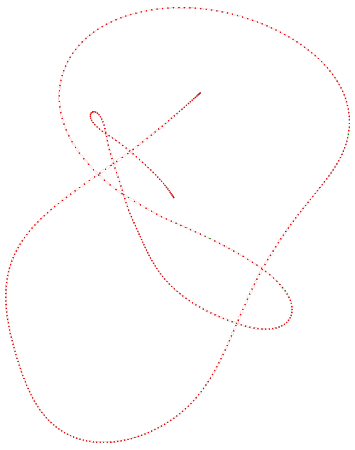	Finished assembly (lacking circularisation)	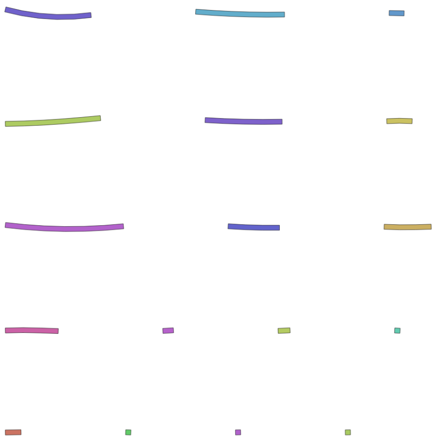
NCTC5793	*Salmonella enterica*	91	1	1	1	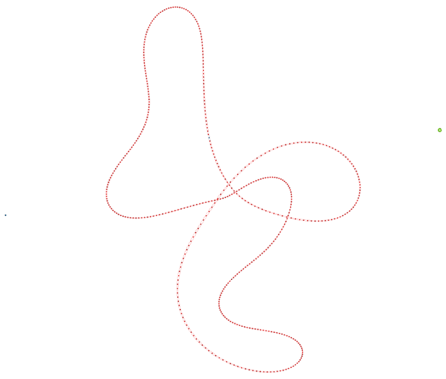	Finished circular assembly	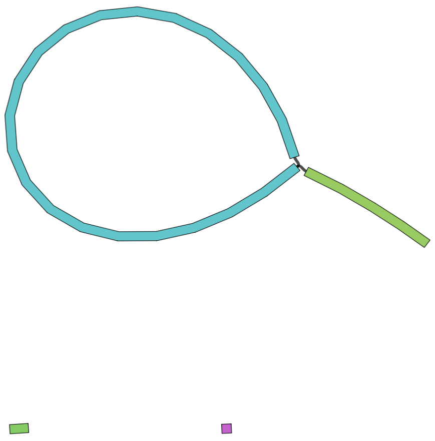
NCTC5798	*Salmonella enterica subsp. enterica*	32	1	1	0		Finished circular assembly	

						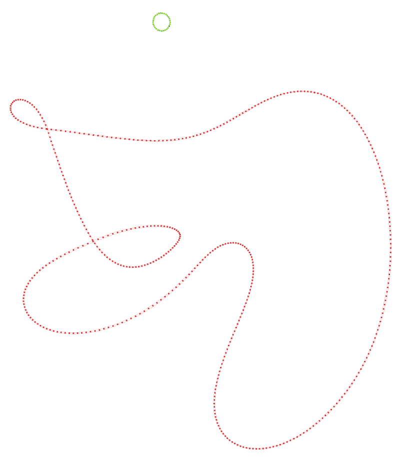		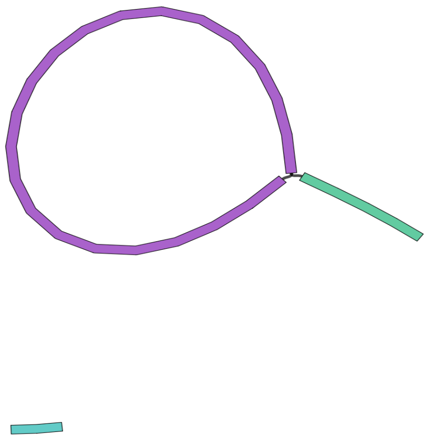
NCTC5934	*Escherichia coli*	51	1	0	0	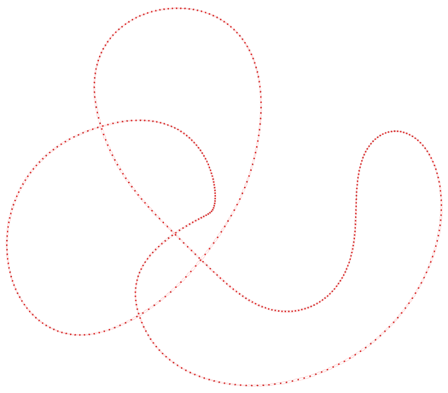	Finished circular assembly	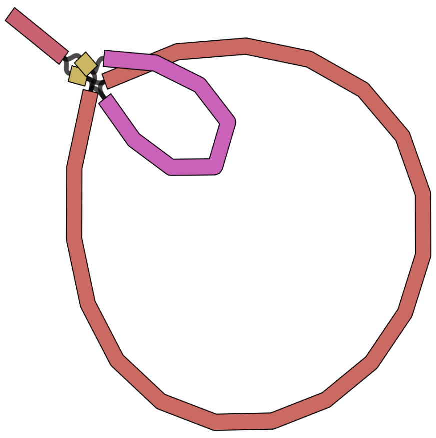
NCTC5968	*Streptococcus sp.*	166	1	0	2	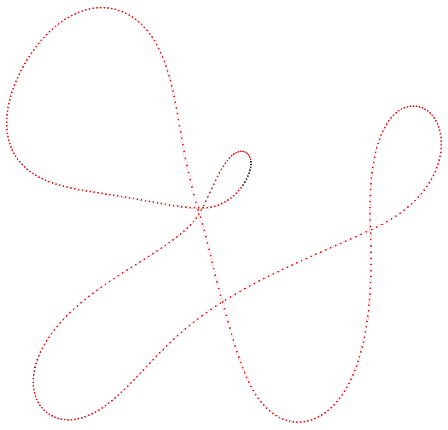	Finished circular assembly	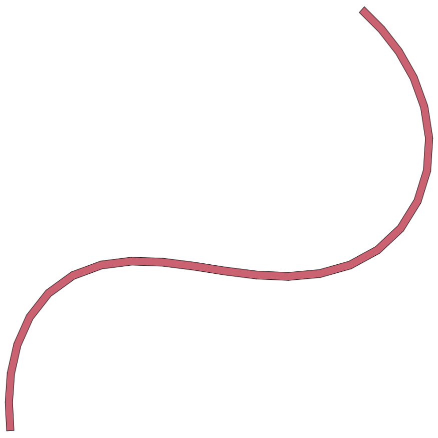
NCTC5969	*Streptococcus sp.*	141	Pending	Pending	Pending	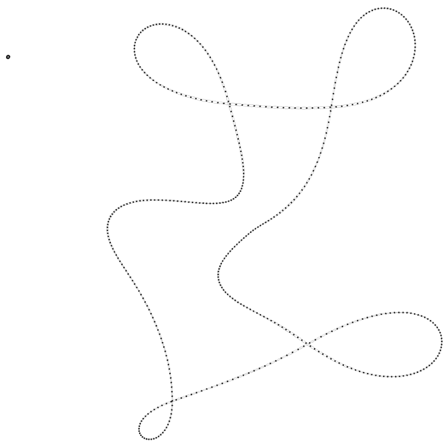	Finished circular assembly	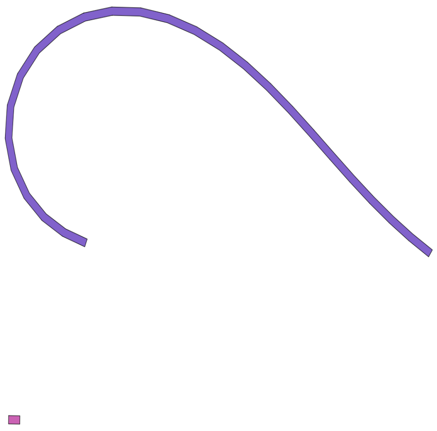
NCTC6017	*Salmonella enterica subsp. enterica*	64	1	0	0	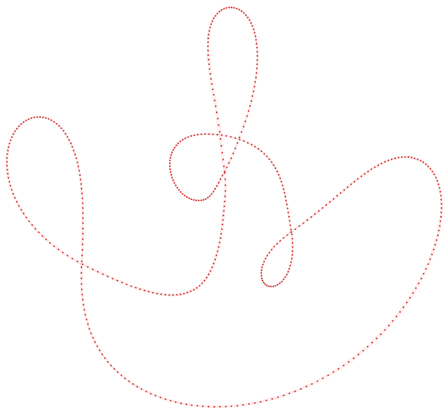	Finished circular assembly	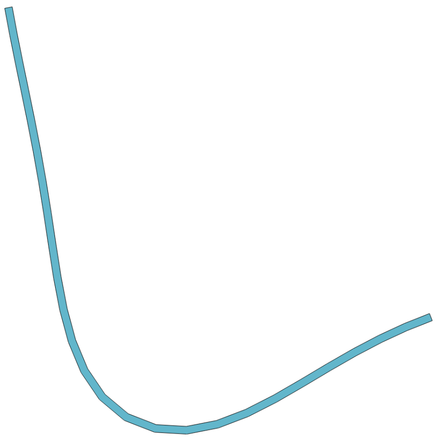
NCTC6024	*Salmonella enterica subsp. enterica*	75	1	0	0	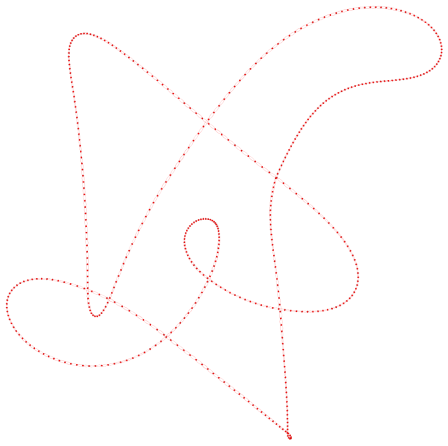	Finished circular assembly	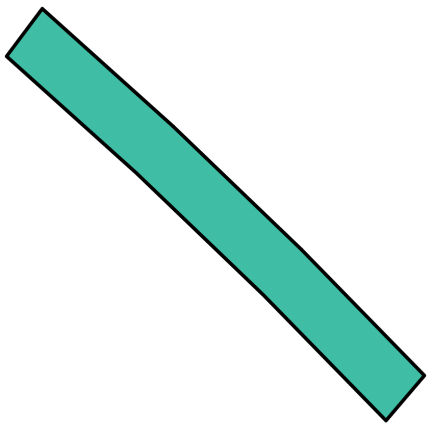
NCTC6086	*Salmonella enterica*	48	0	0	1		Finished circular assembly	

						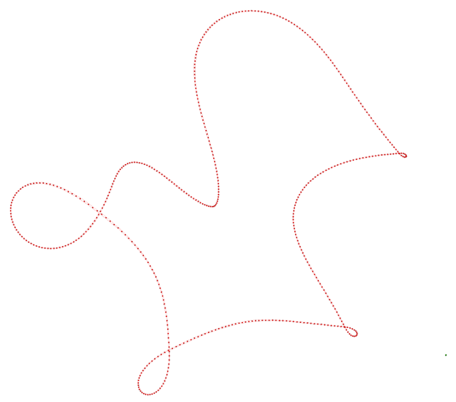			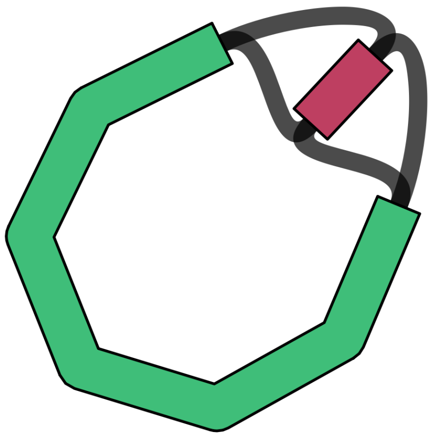
NCTC6131	*Staphylococcus aureus*	43	1	0	0	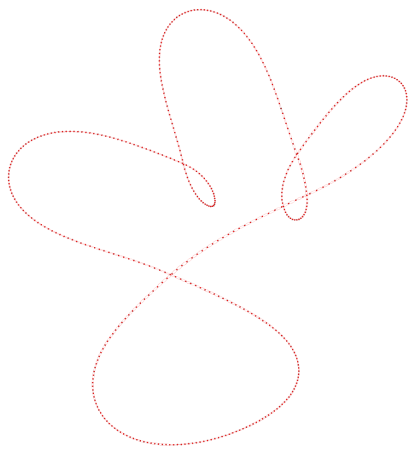		Finished circular assembly	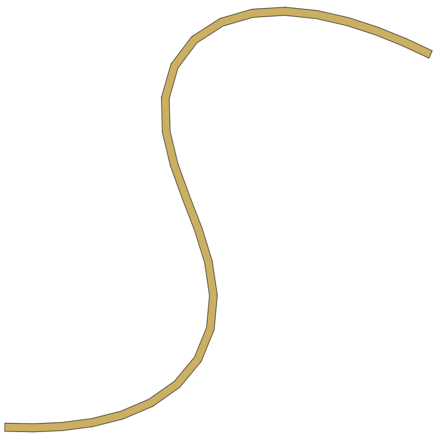
NCTC6133	*Staphylococcus aureus*	25	1	1	0	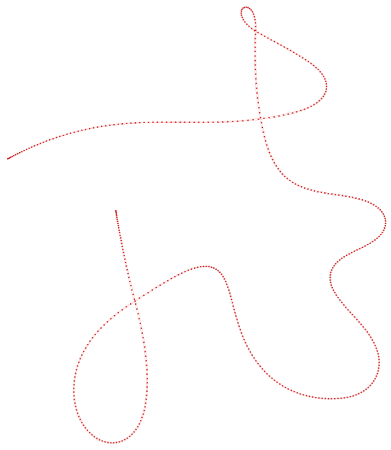		Finished assembly (lacking circularisation)	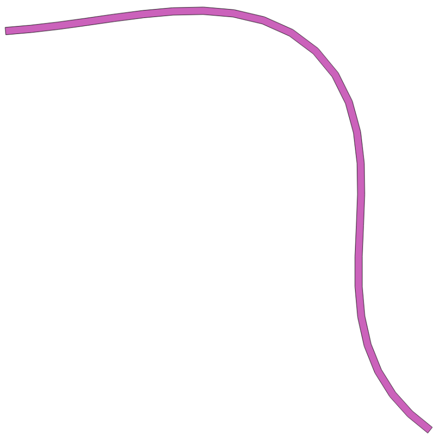
NCTC6134	*Staphylococcus aureus*	38	0	0	5	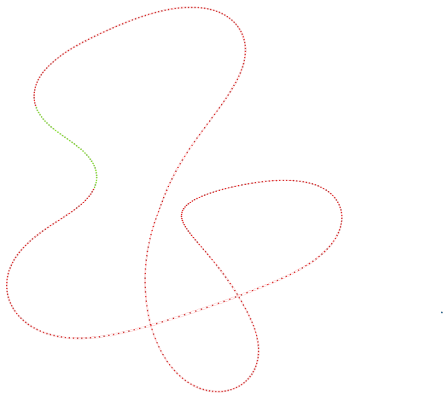		Finished circular assembly	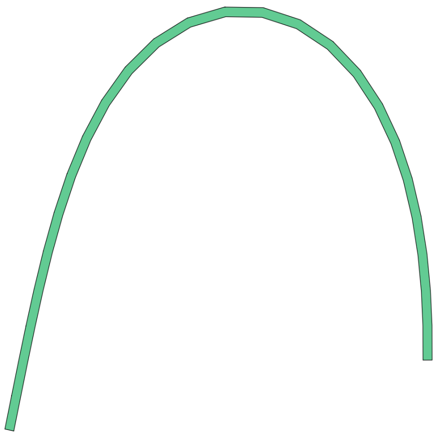
NCTC6135	*Staphylococcus aureus*	118	1	1	0	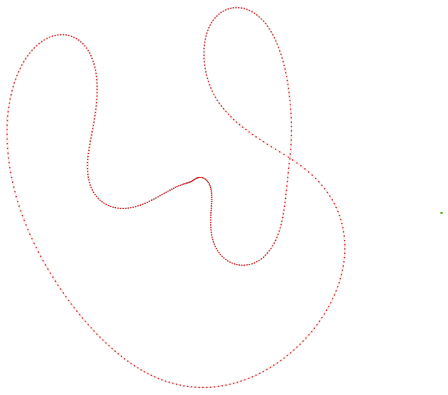		Finished circular assembly	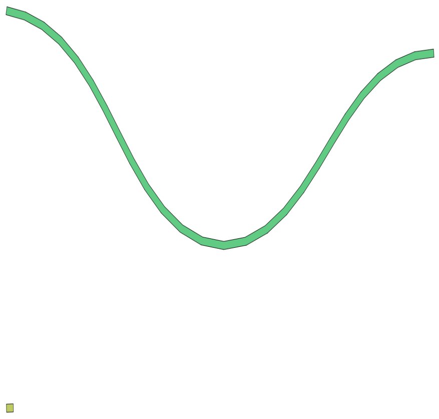
NCTC6136	*Staphylococcus aureus*	112	1	0	0	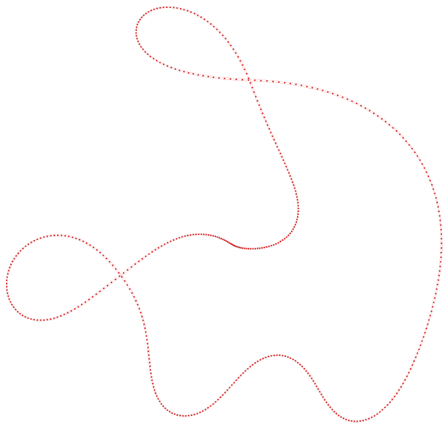		Finished circular assembly	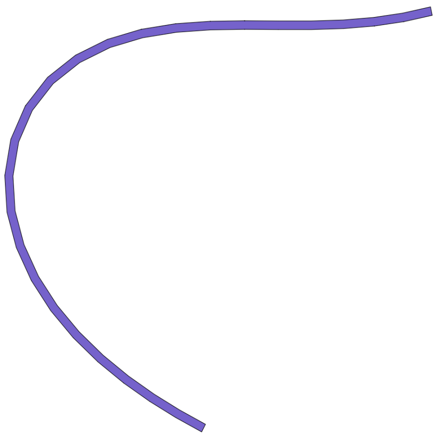
NCTC6137	*Staphylococcus aureus*	127	0	0	0			Finished circular assembly	

						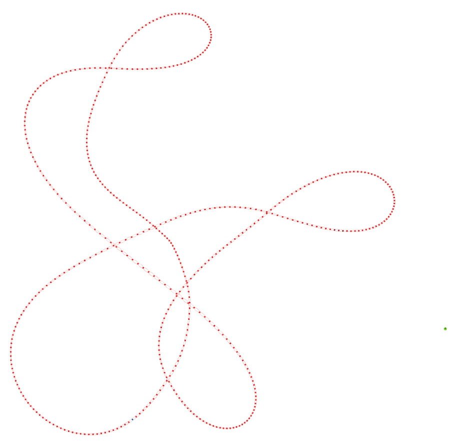		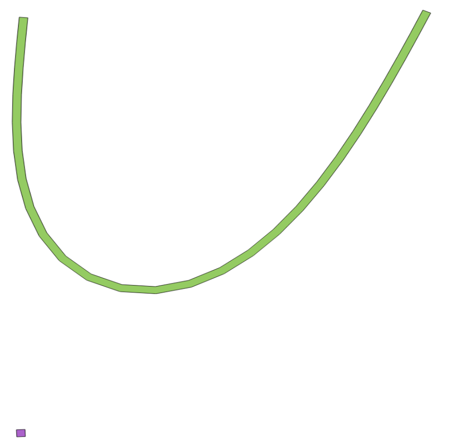
NCTC6179	*Streptococcus dysgalactiae subsp. equisimilis*	85	Pending	Pending	Pending	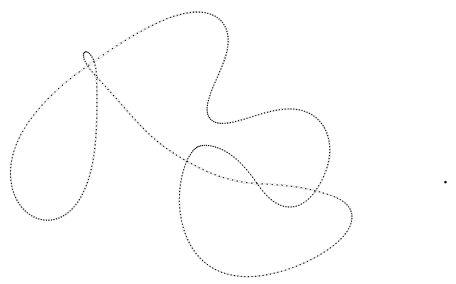	Finished circular assembly	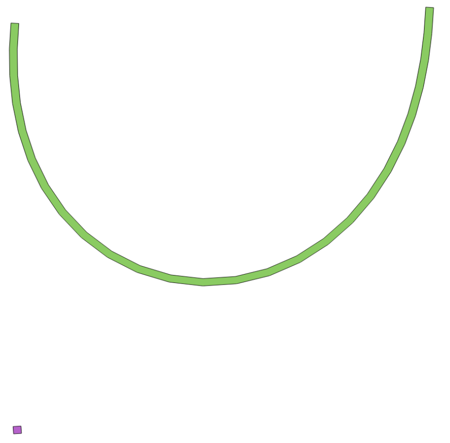
NCTC6198	*Streptococcus sp.*	102	Pending	Pending	Pending	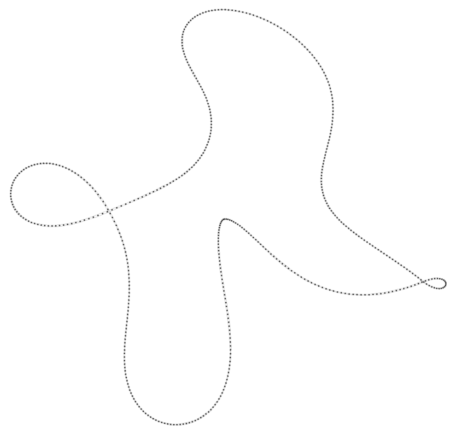	Finished circular assembly	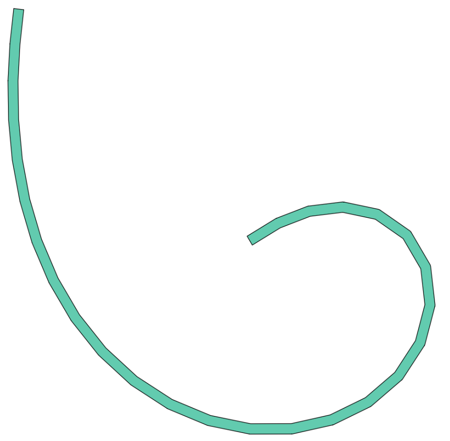
NCTC6245	*Salmonella enterica subsp. enterica*	30	1	1	0	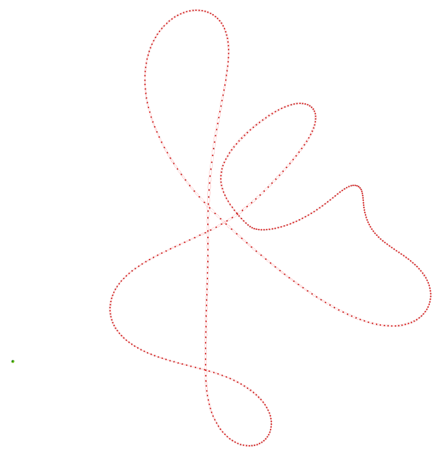	Finished circular assembly	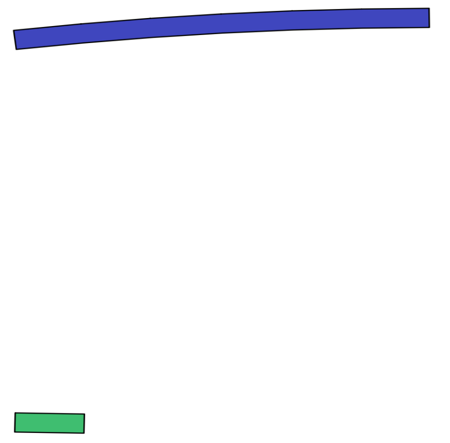
NCTC6267	*Citrobacter freundii*	55	1	0	2	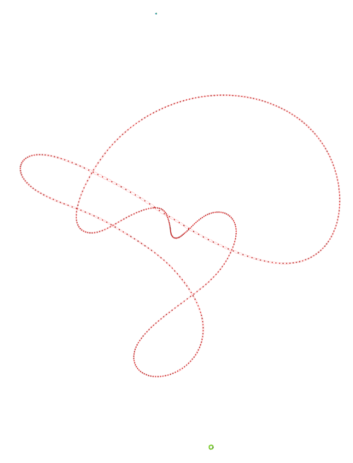	Finished circular assembly	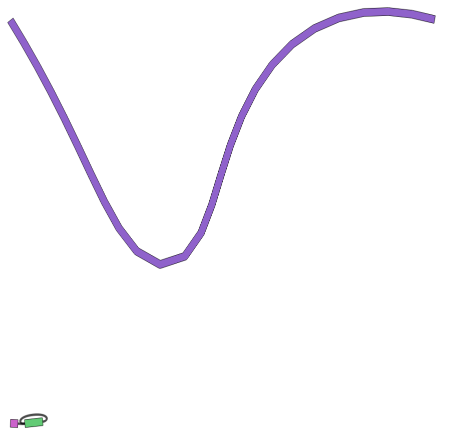
NCTC6385	*Salmonella enterica*	66	1	1	1	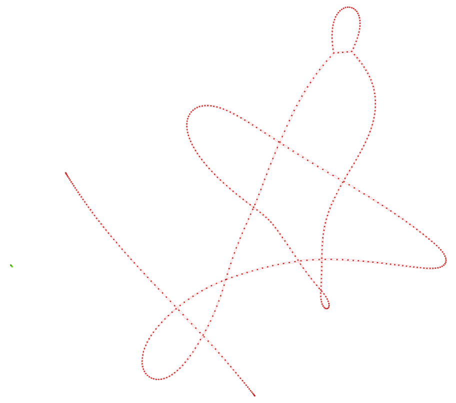	Mis-assembly/Fragmented	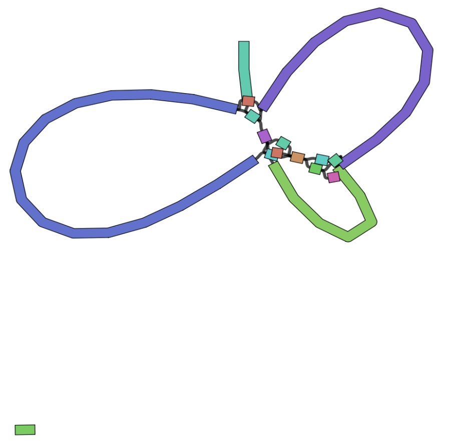
NCTC6403	*Streptococcus sp.*	41	Pending	Pending	Pending		Mis-assembly/Fragmented	

						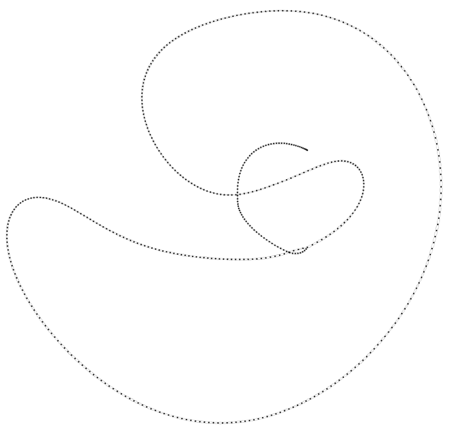		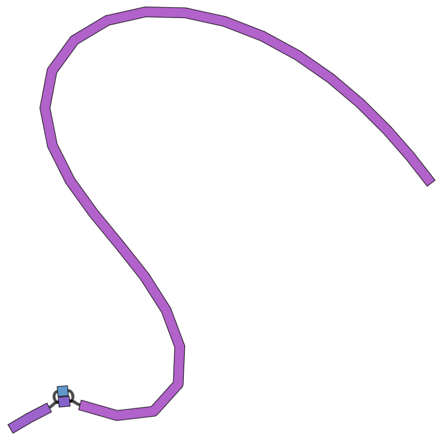
NCTC6407	*Streptococcus sp.*	164	1	0	1	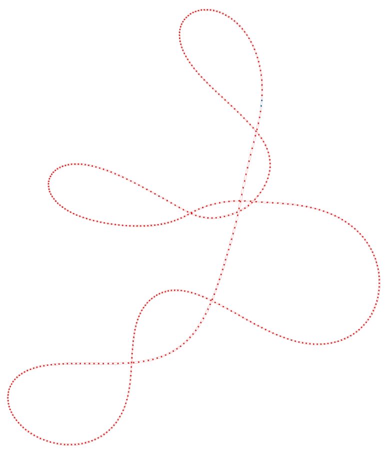	Finished circular assembly	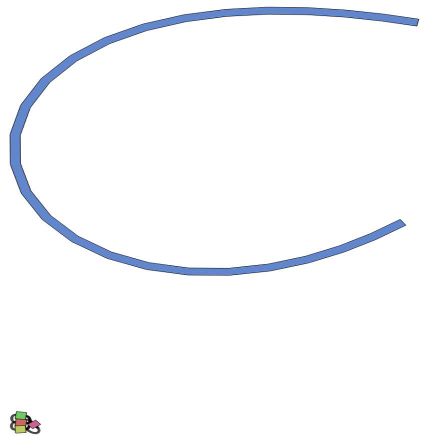
NCTC6482	*Salmonella enterica subsp. enterica serovar Madelia*	24	1	0	1	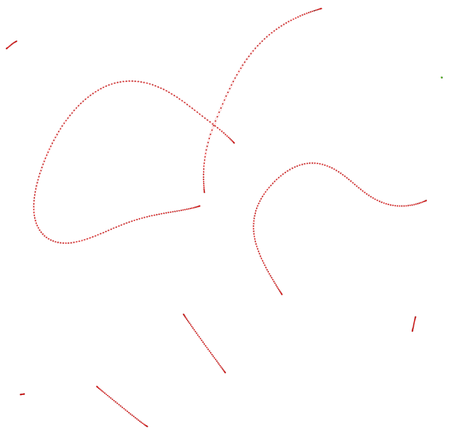	Mis-assembly/Fragmented	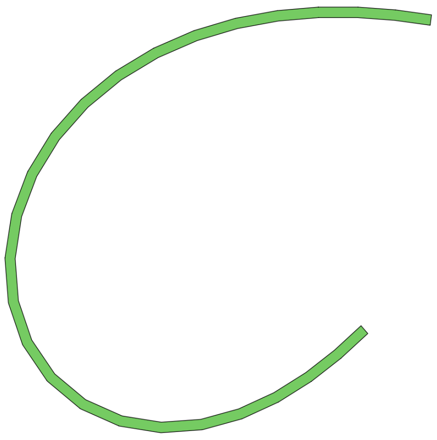
NCTC6507	*Staphylococcus aureus*	109	1	1	0	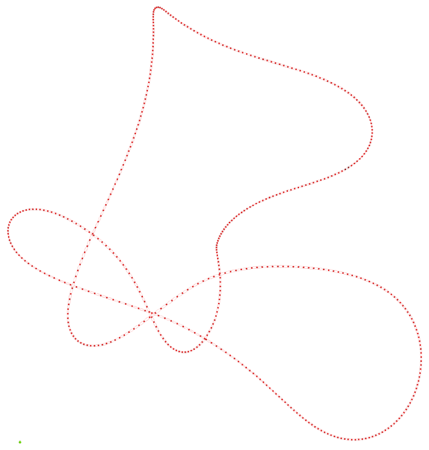	Finished circular assembly	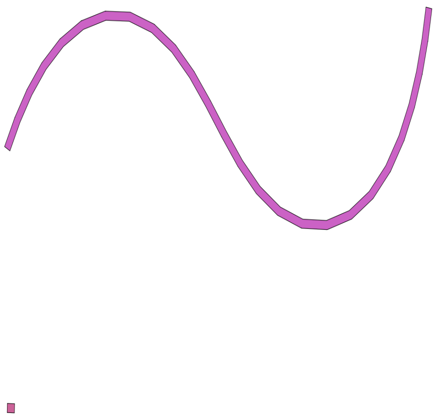
NCTC6513	*Staphylococcus epidermidis*	155	1	2	0	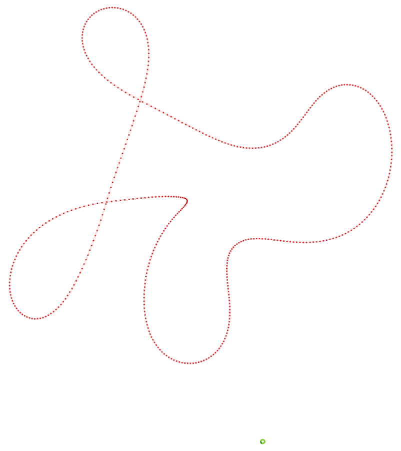	Finished circular assembly	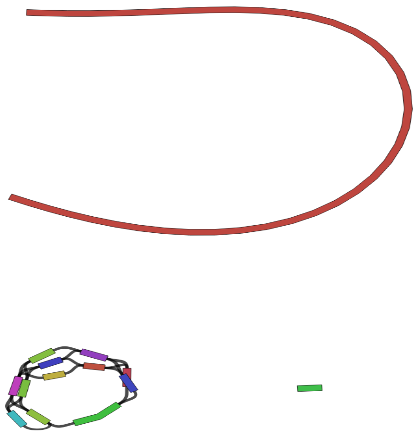
NCTC6571	*Staphylococcus aureus*	138	1	1	0	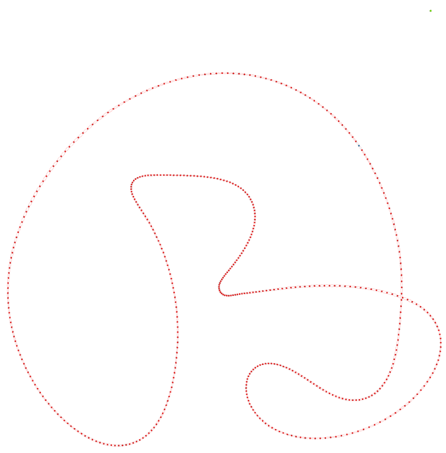	Finished circular assembly	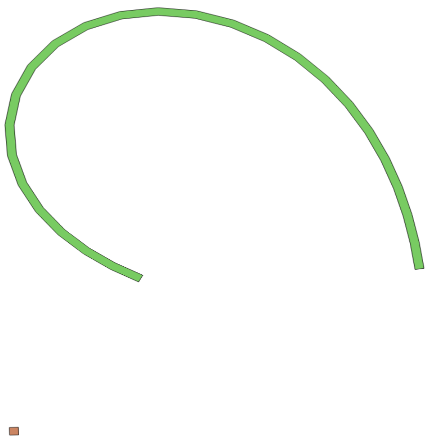
NCTC6754	*Salmonella*	25	0	0	1		Finished circular assembly	

						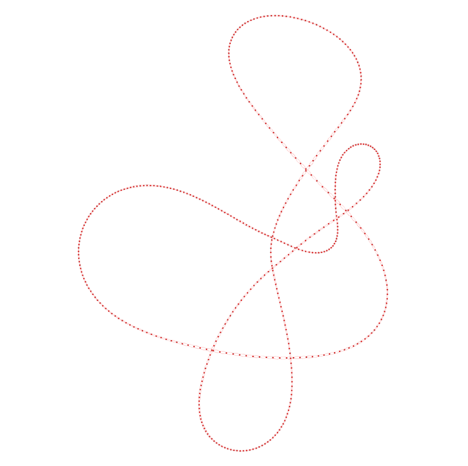		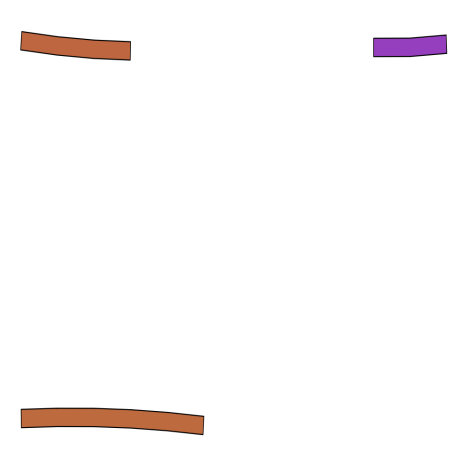
NCTC6759	*Salmonella enterica subsp. enterica*	79	1	0	0	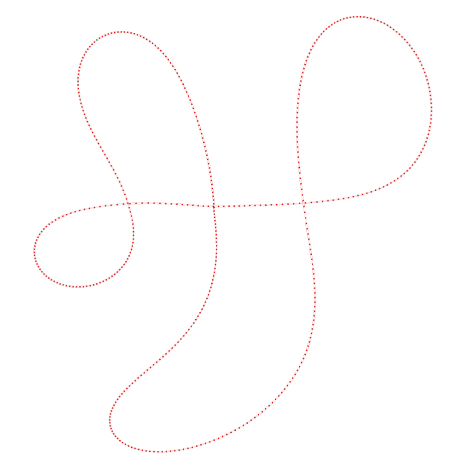	Finished circular assembly	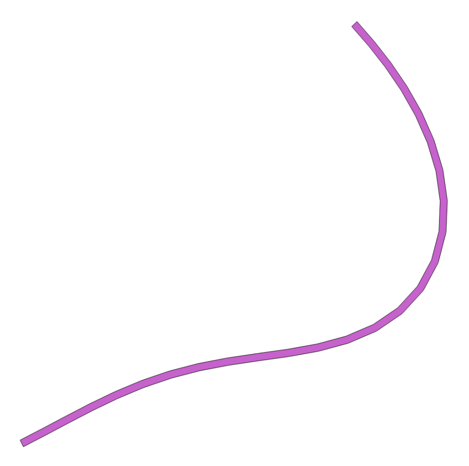
NCTC6802	*Salmonella enterica*	39	1	1	0	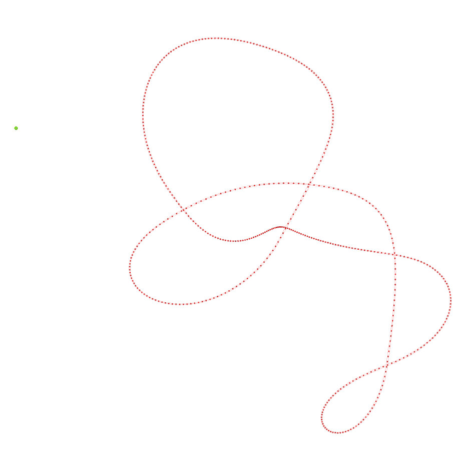	Finished circular assembly	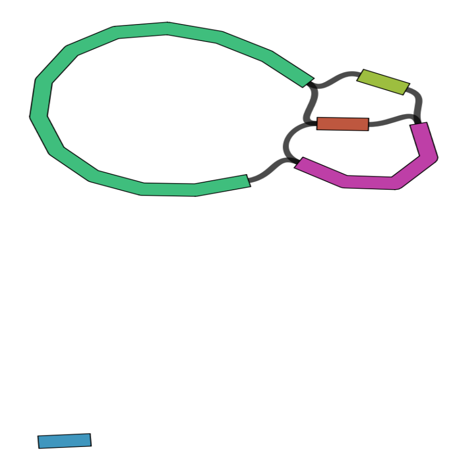
NCTC6933	*Providencia rustigianii*	52	1	0	0	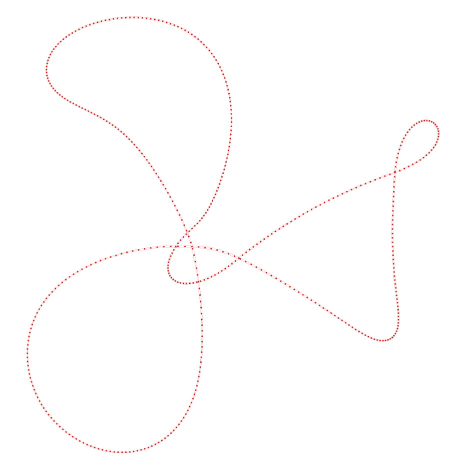	Finished circular assembly	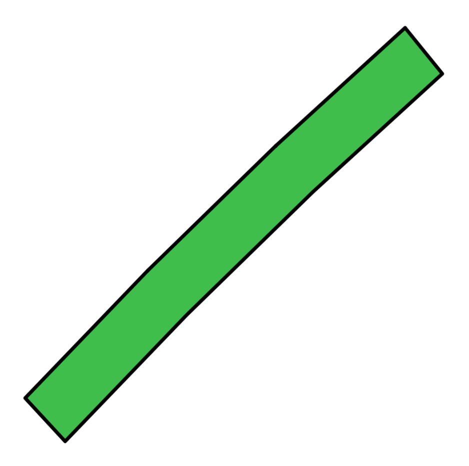
NCTC6947	*Salmonella sp.*	40	1	1	0	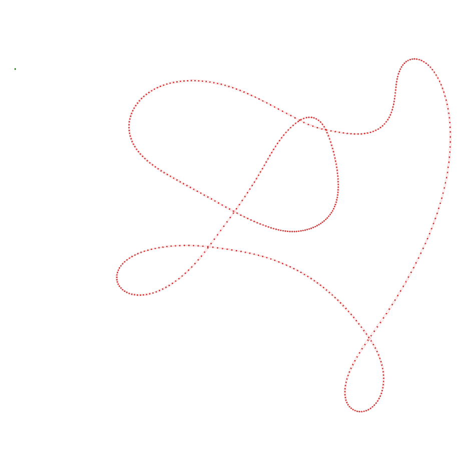	Finished circular assembly	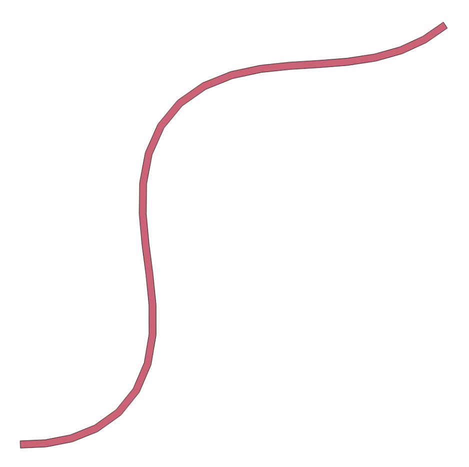
NCTC6966	*Staphylococcus aureus*	83	1	1	0	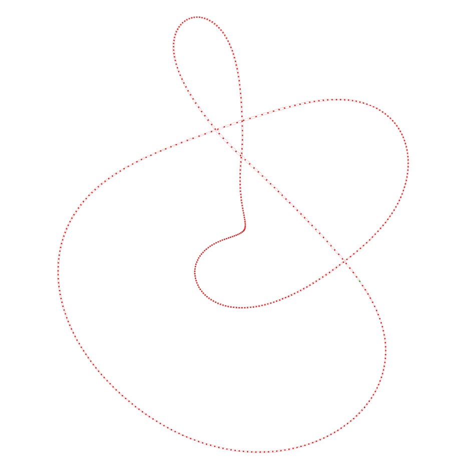	Finished circular assembly	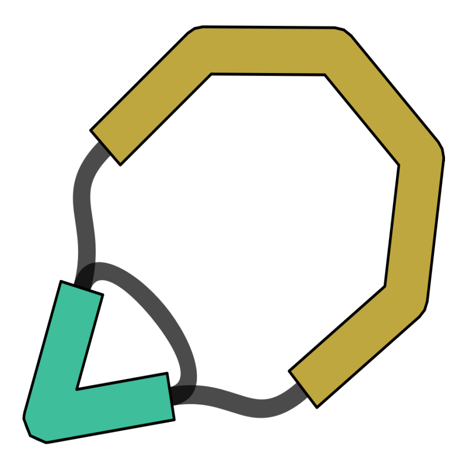
NCTC7022	*Streptococcus sp.*	172	1	0	0		Finished circular assembly	

						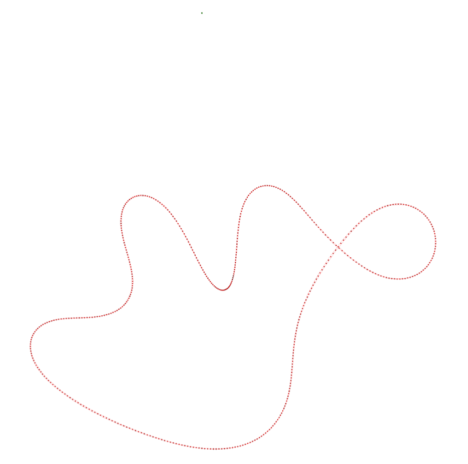		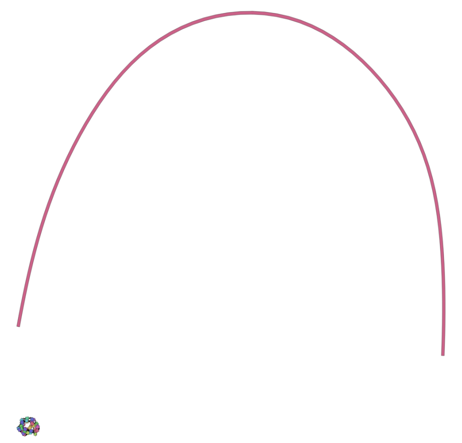
NCTC7023	*Streptococcus sp*	94	0	0	2	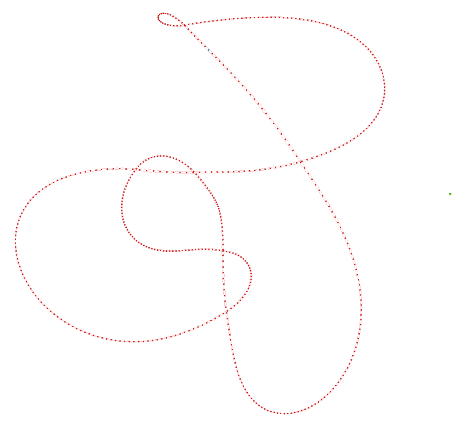	Finished circular assembly	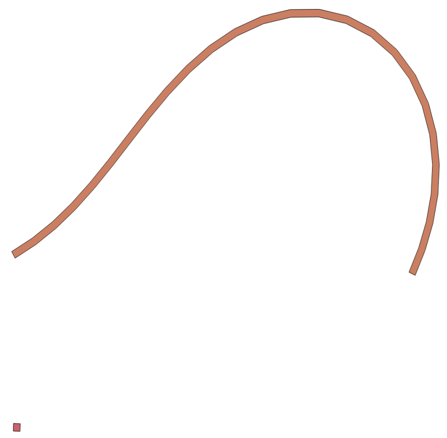
NCTC7102	*Salmonella enterica subsp. enterica*	29	1	0	0	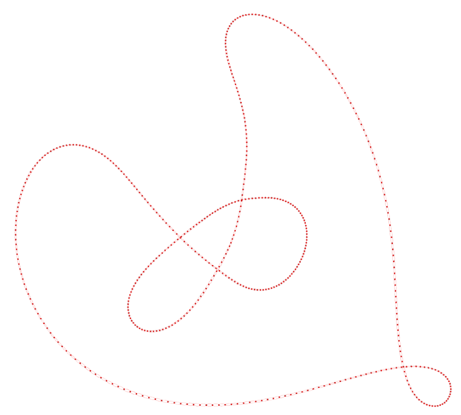	Finished circular assembly	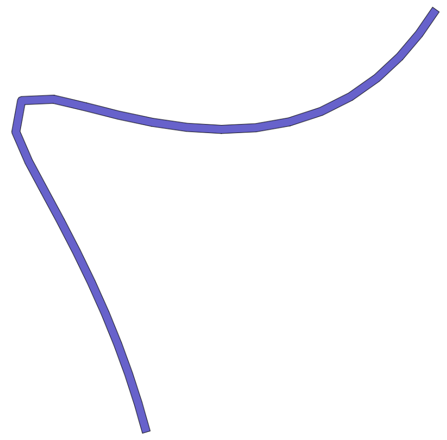
NCTC7121	*Staphylococcus aureus*	82	1	0	0	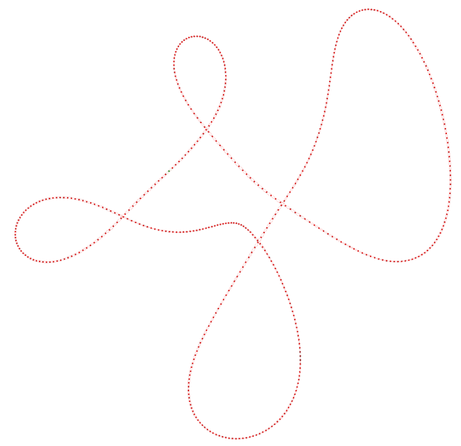	Finished circular assembly	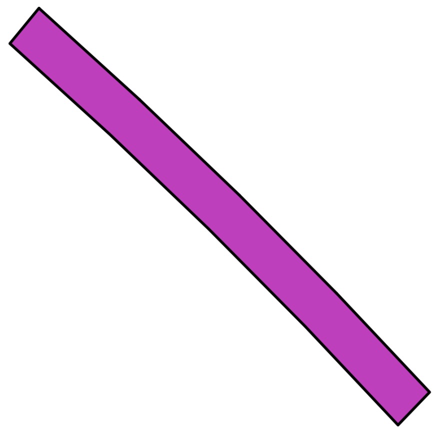
NCTC7151	*Staphylococcus aureus*	115	0	0	5	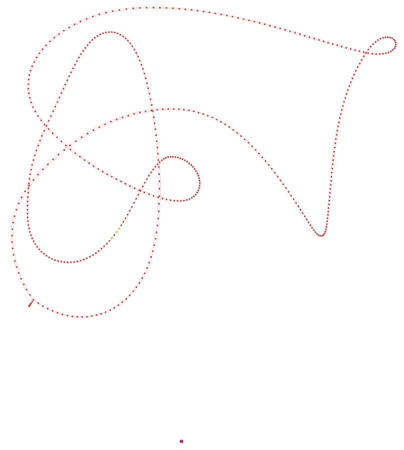	Finished circular assembly	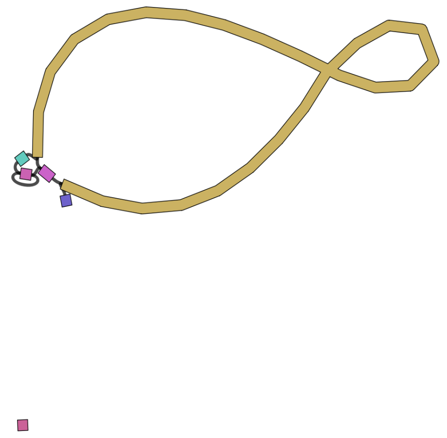
NCTC7152	*Escherichia coli*	49	1	0	4	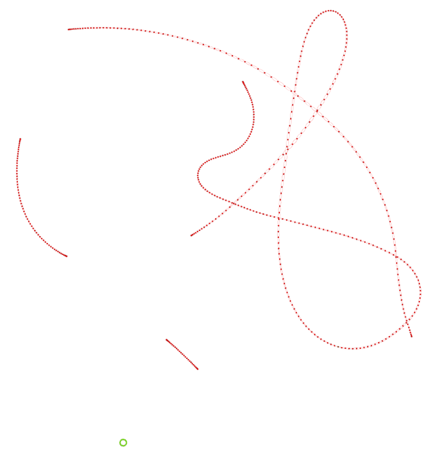	Mis-assembly/Fragmented	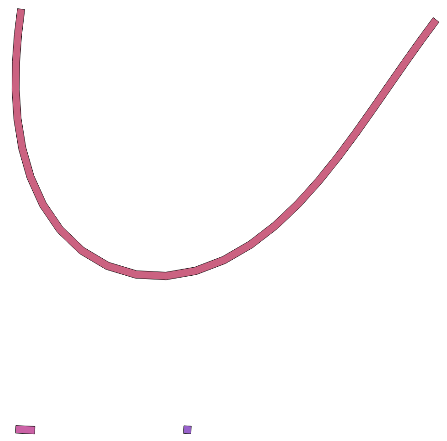
NCTC7164	*Streptococcus sp.*	109	Pending	Pending	Pending		Finished circular assembly	

						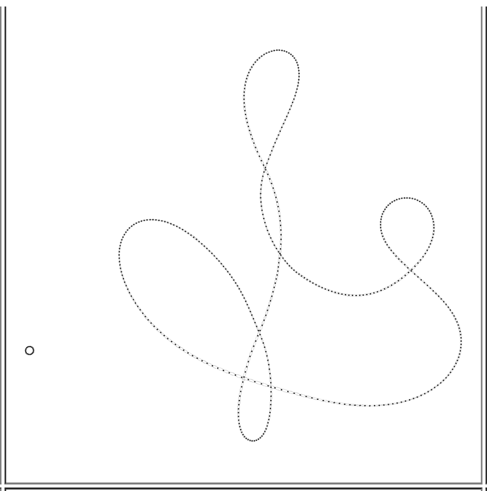		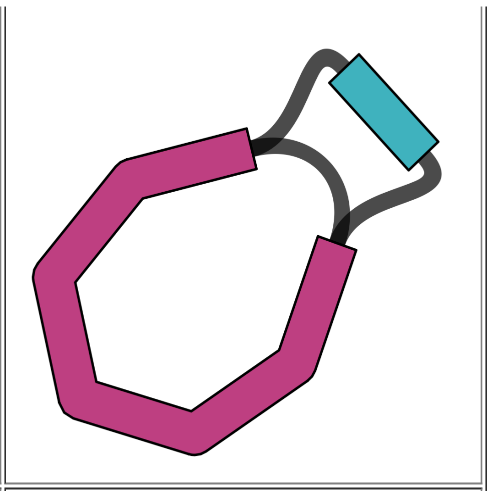
NCTC7169	*Streptococcus sp.*	98	Pending	Pending	Pending	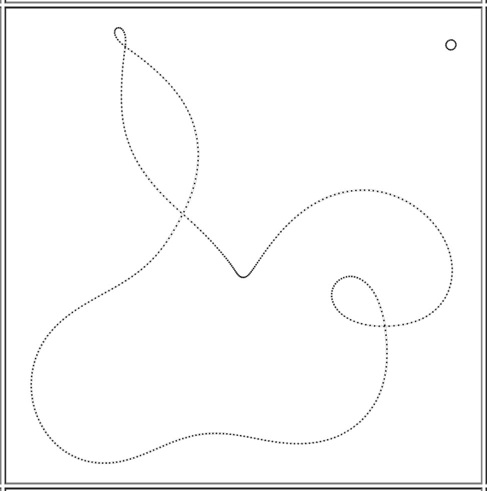	Finished circular assembly	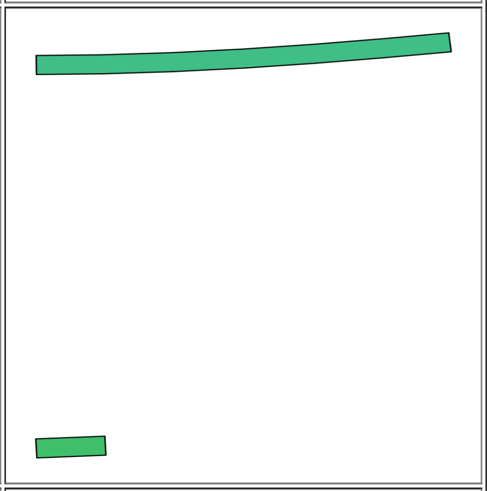
NCTC7170	*Streptococcus sp.*	113	Pending	Pending	Pending	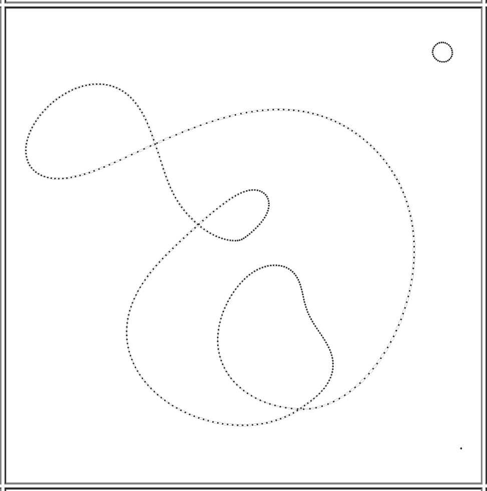	Finished circular assembly	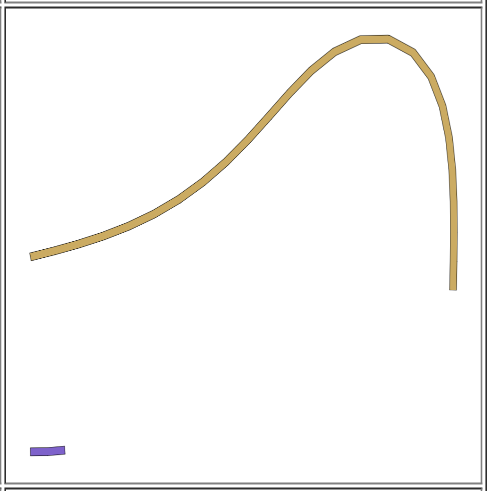
NCTC7173	*Streptococcus sp.*	146	Pending	Pending	Pending	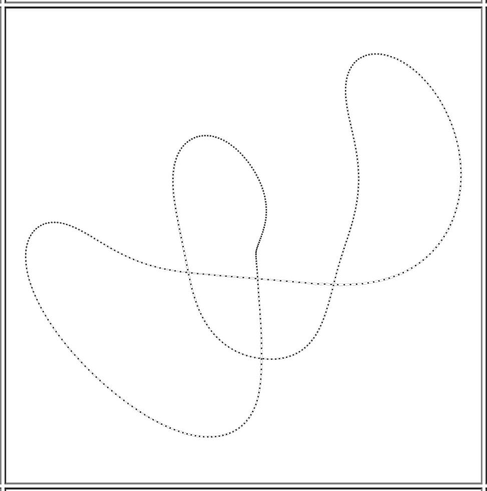	Finished circular assembly	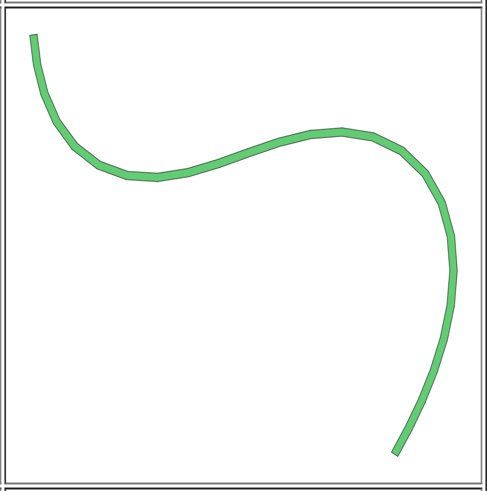
NCTC7175	*Streptococcus sp.*	88	Pending	Pending	Pending	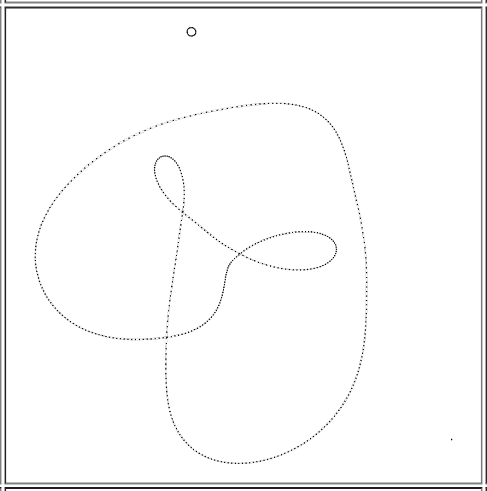	Finished circular assembly	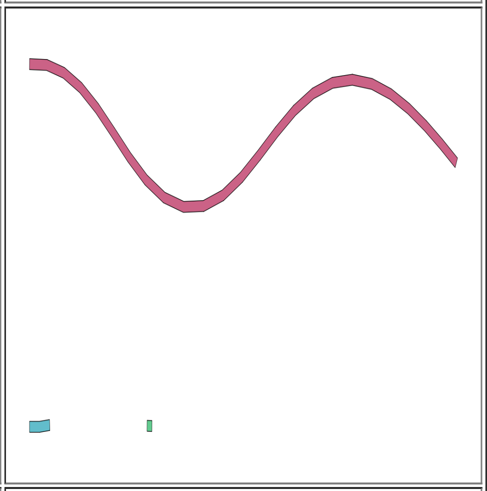
NCTC7179	*Streptococcus sp.*	6	Pending	Pending	Pending	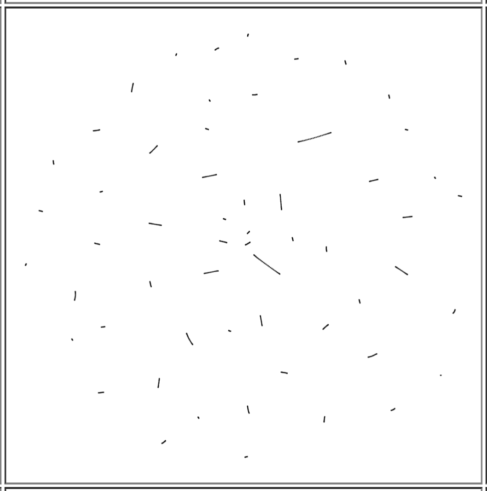	Mis-assembly/Fragmented	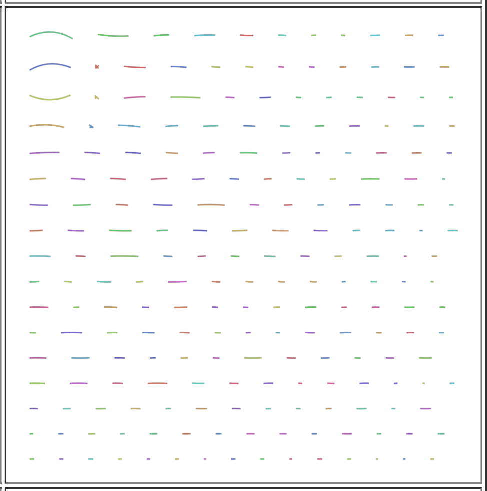
NCTC7242	*Klebsiella sp.*	49	Pending	Pending	Pending		Finished circular assembly	

						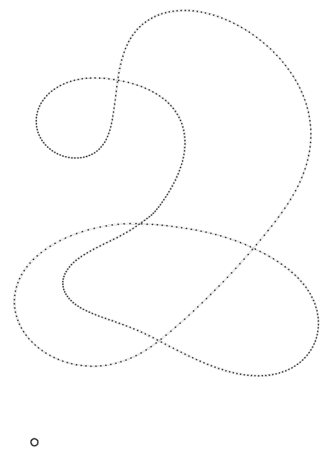		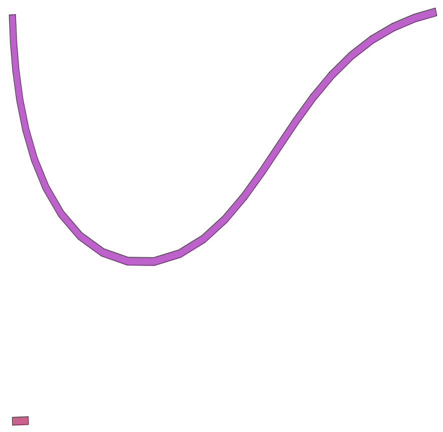
NCTC7291	*Staphylococcus warneri*	156	1	0	0	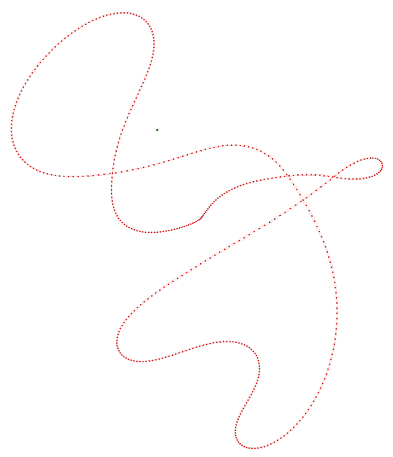	Finished circular assembly	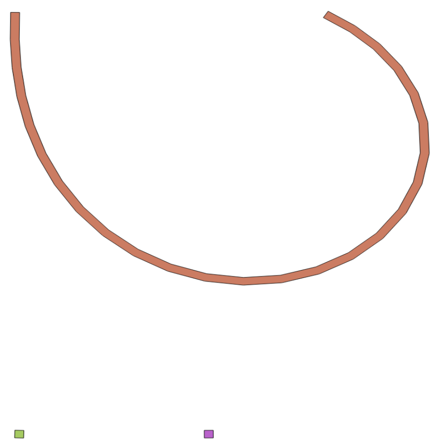
NCTC7292	*Staphylococcus saprophyticus*	101	0	0	3	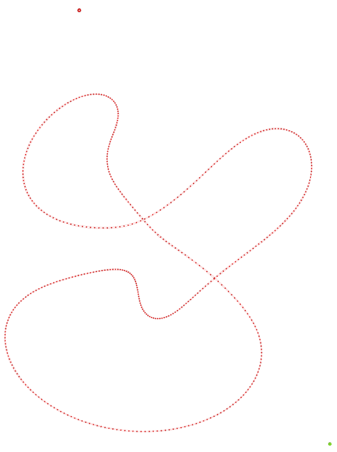	Finished circular assembly	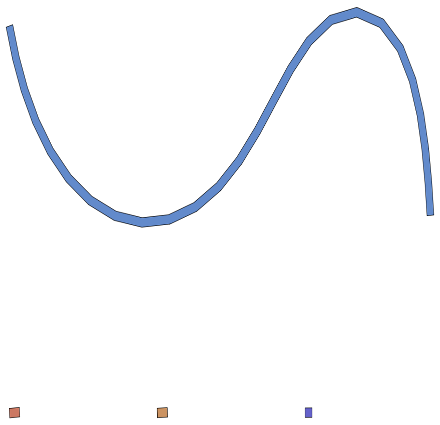
NCTC7295	*Salmonella enterica subsp. arizonae*	49	1	1	0	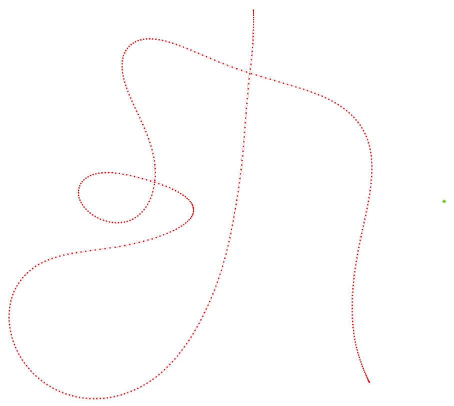	Finished assembly (lacking circularisation)	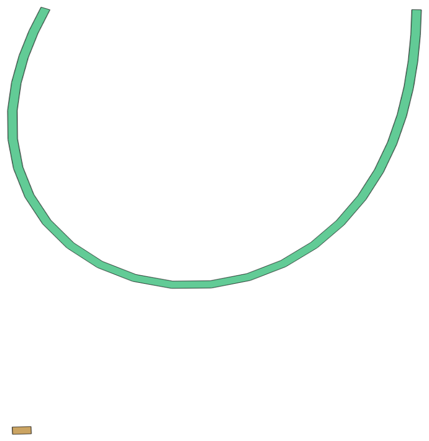
NCTC7297	*Salmonella sp.*	27	1	0	0	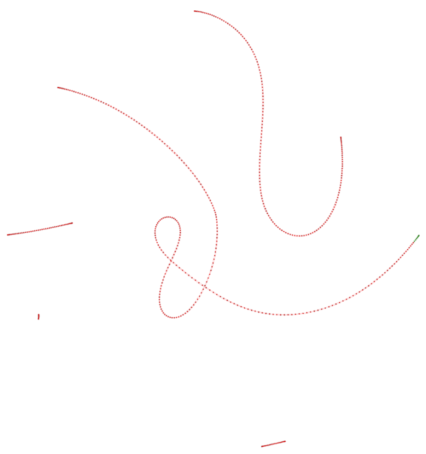	Mis-assembly/Fragmented	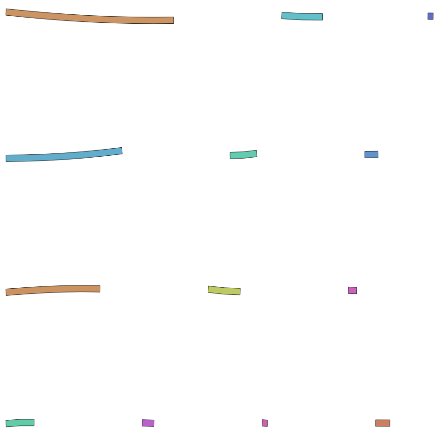
NCTC7299	*Salmonella enterica subsp. arizonae*	45	1	1	0	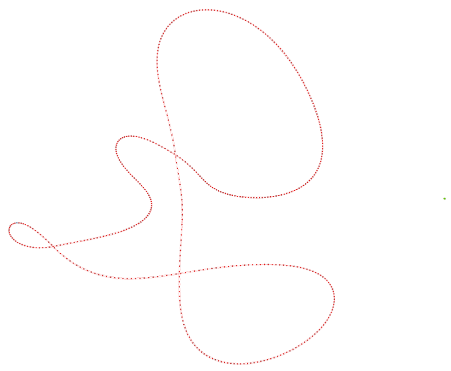	Finished circular assembly	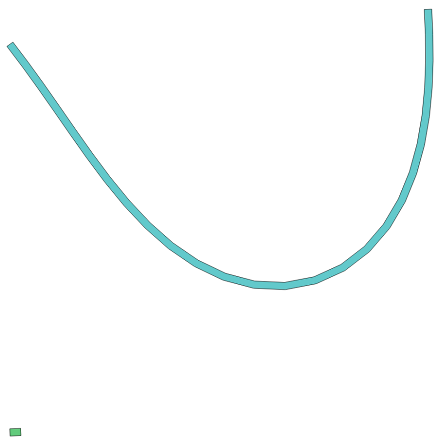
NCTC7300	*Salmonella enterica subsp. arizonae*	38	1	1	0		Finished circular assembly	

						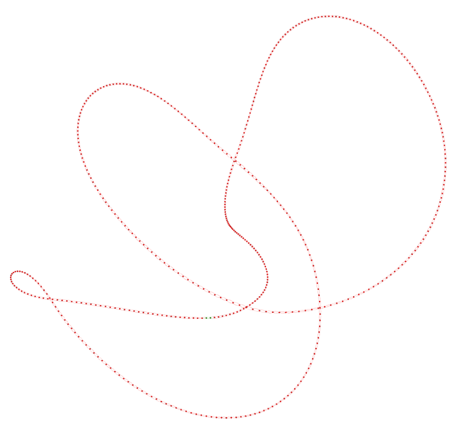		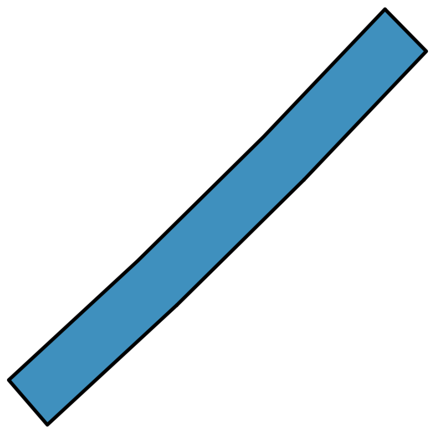
NCTC7302	*Salmonella enterica subsp. arizonae*	54	1	0	0	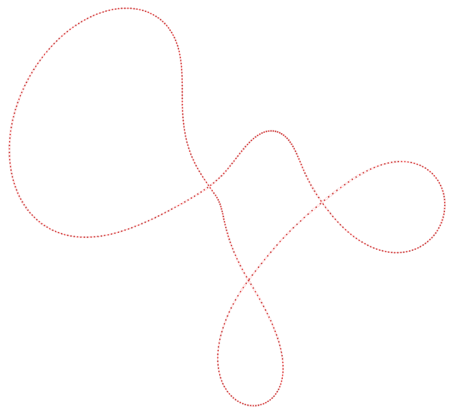	Finished circular assembly	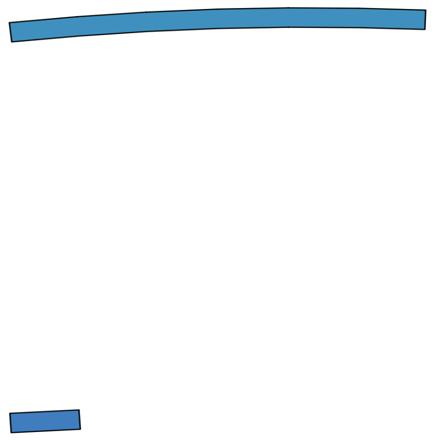
NCTC7303	*Salmonella enterica subsp. arizonae*	43	1	1	0	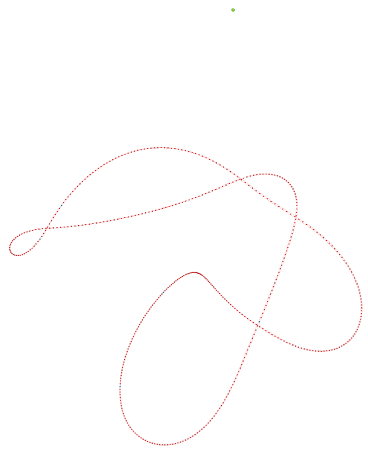	Finished circular assembly	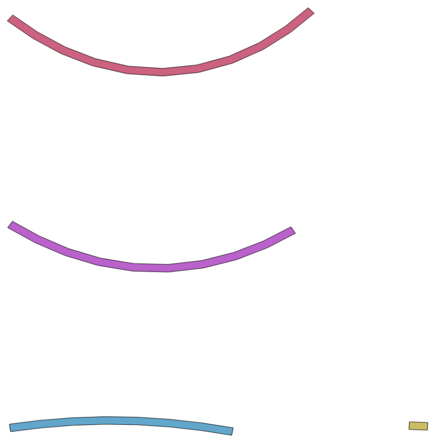
NCTC7304	*Salmonella enterica subsp. arizonae*	34	1	0	1	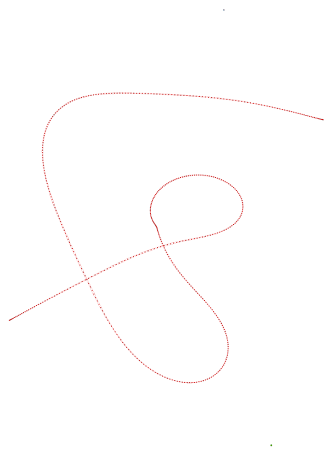	Finished assembly (lacking circularisation)	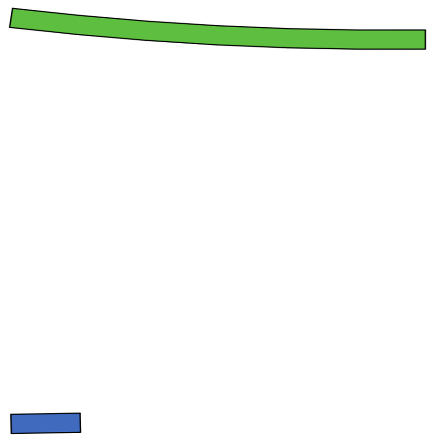
NCTC7306	*Salmonella enterica subsp. arizonae*	92	0	0	3	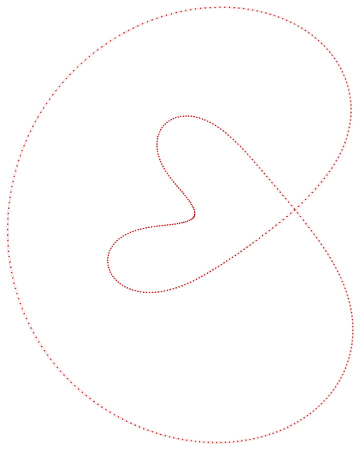	Finished circular assembly	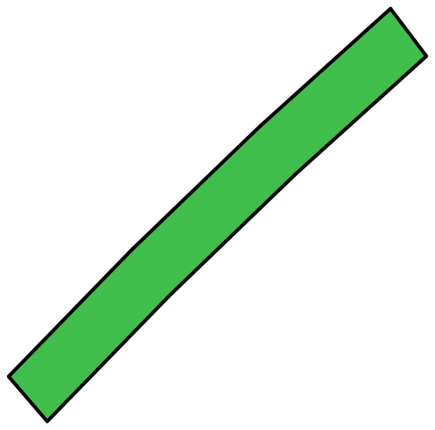
NCTC7307	*Salmonella enterica subsp. arizonae*	32	1	0	0	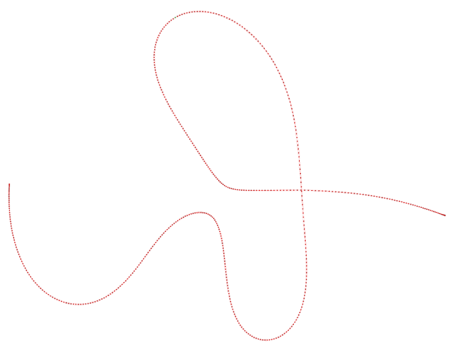	Finished assembly (lacking circularisation)	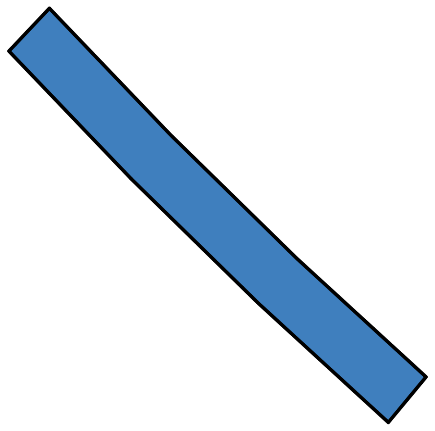
NCTC7308	*Salmonella enterica subsp. arizonae*	91	1	2	0		Finished assembly with multiple traversals	

						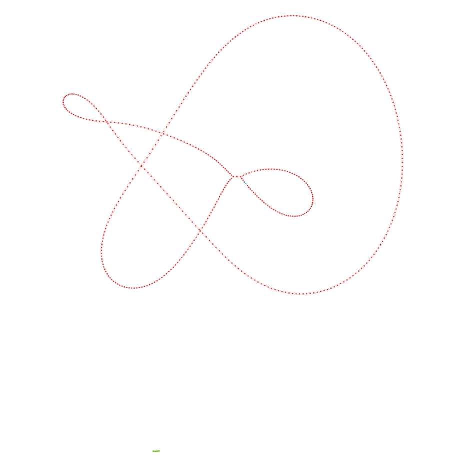		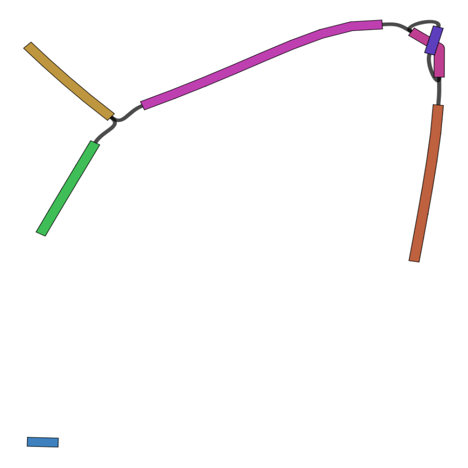
NCTC7318	*Salmonella enterica subsp. houtenae*	284	1	0	0	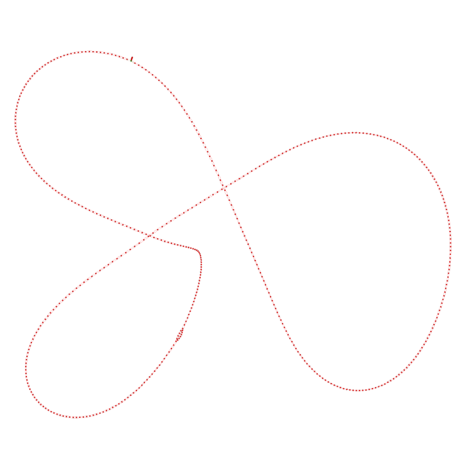	Finished circular assembly	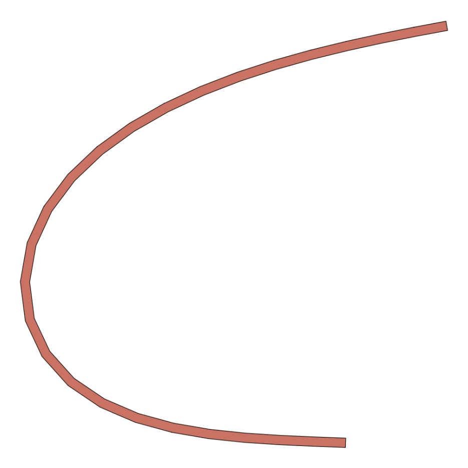
NCTC7323	*Salmonella enterica subsp. arizonae*	58	1	0	0	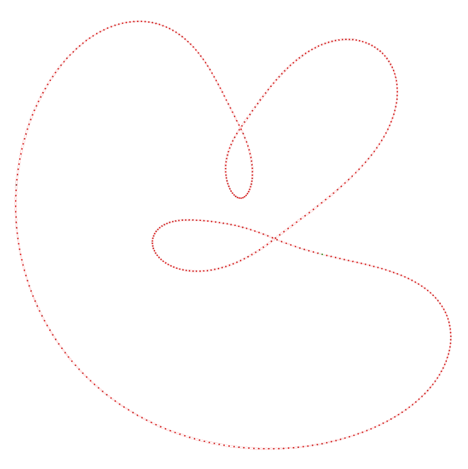	Finished circular assembly	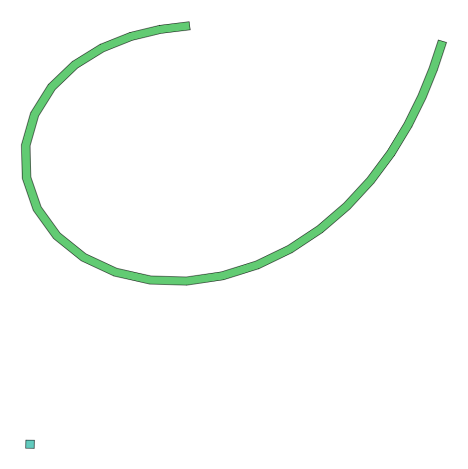
NCTC7349	*Salmonella enterica subsp. arizonae*	46	1	0	1	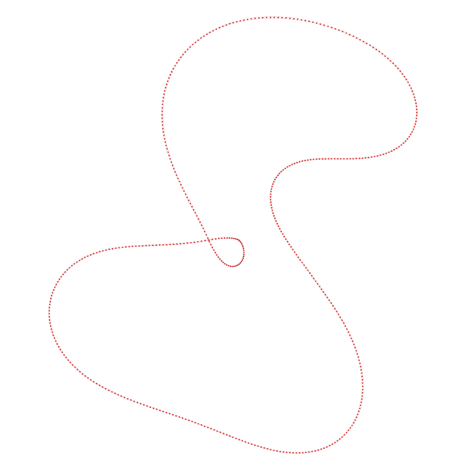	Finished circular assembly	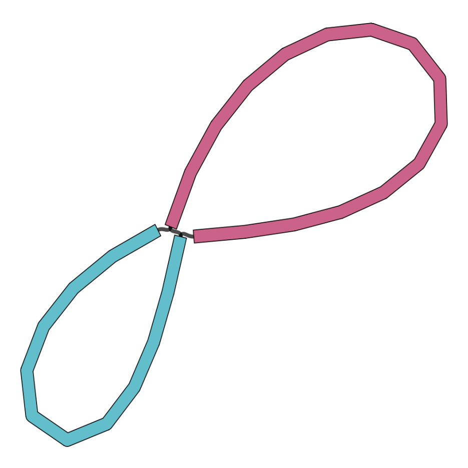
NCTC7360	*Escherichia coli*	45	1	1	0	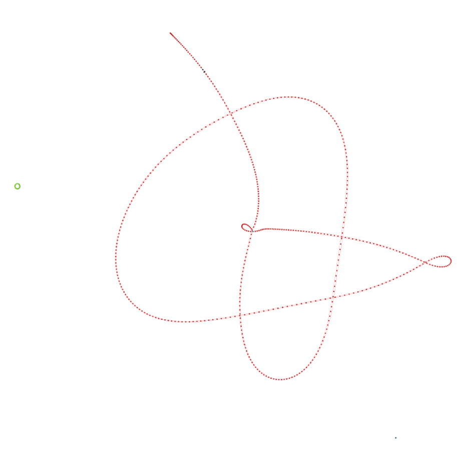	Mis-assembly/Fragmented	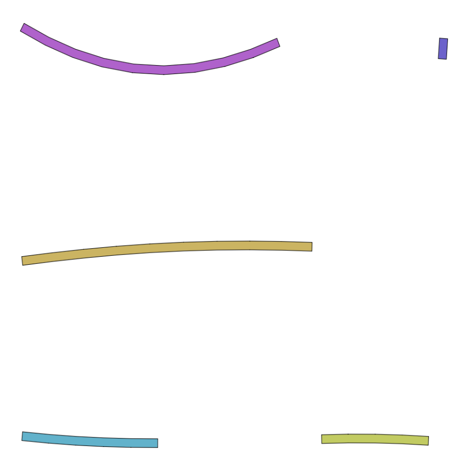
NCTC7361	*Staphylococcus aureus*	101	0	0	2	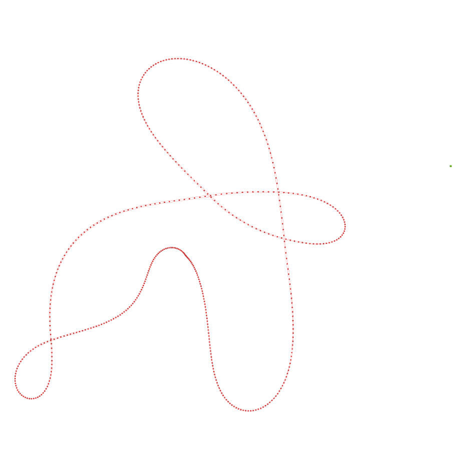	Finished circular assembly	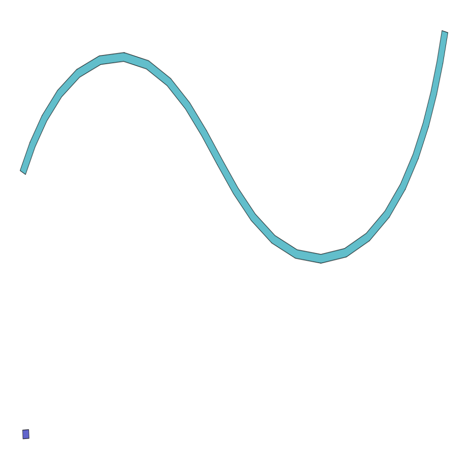
NCTC7362	*Escherichia coli*	70	0	0	4		Finished circular assembly	

						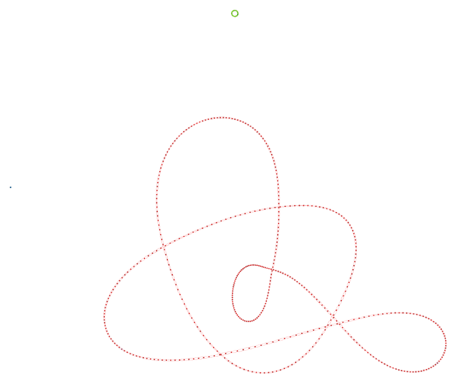		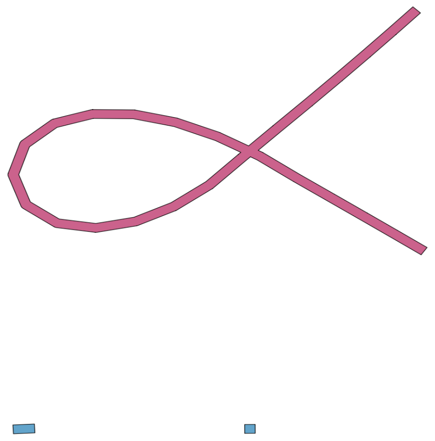
NCTC7364	*Acinetobacter calcoaceticus*	64	1	1	0	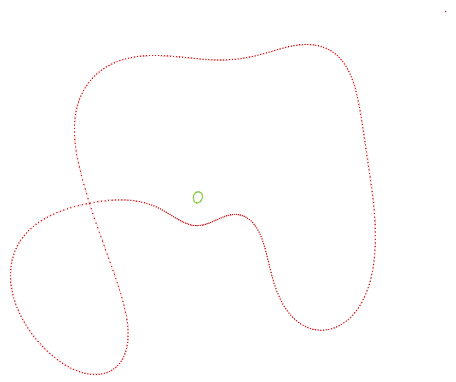	Finished circular assembly	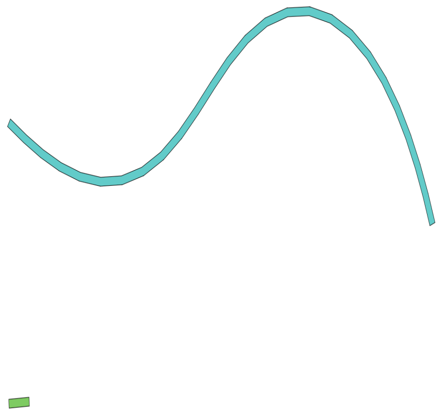
NCTC7366	*Streptococcus salivarius*	110	Pending	Pending	Pending	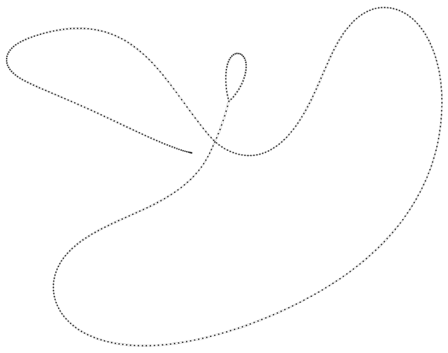	Finished assembly (lacking circularisation)	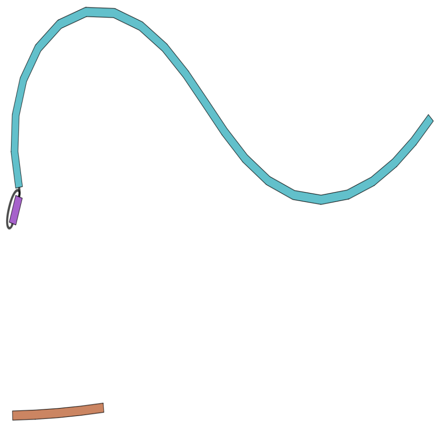
NCTC7377	*Streptococcus sp.*	93	Pending	Pending	Pending	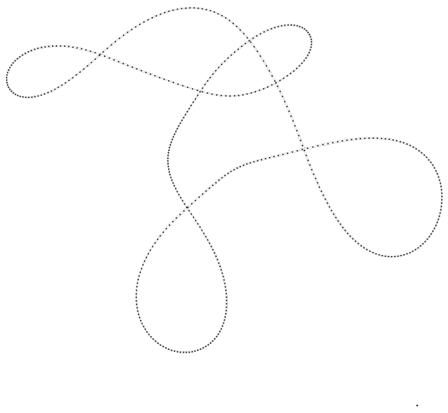	Finished circular assembly	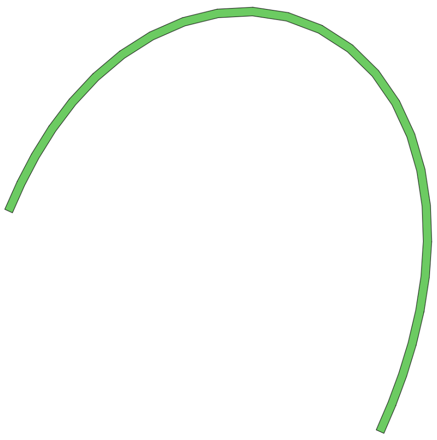
NCTC7404	*Salmonella enterica subsp. enterica*	83	1	0	0	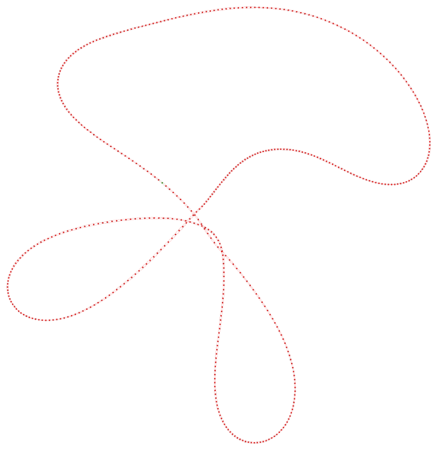	Finished circular assembly	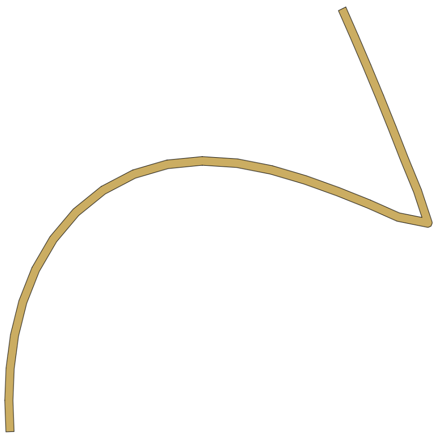
NCTC7406	*Salmonella sp.*	42	1	0	0	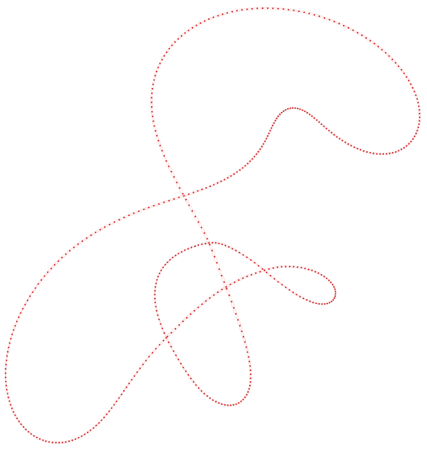	Finished circular assembly	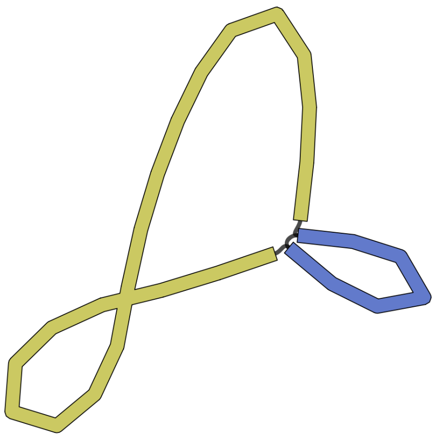
NCTC7411	*Salmonella enterica subsp. enterica*	55	1	0	0		Finished circular assembly	

						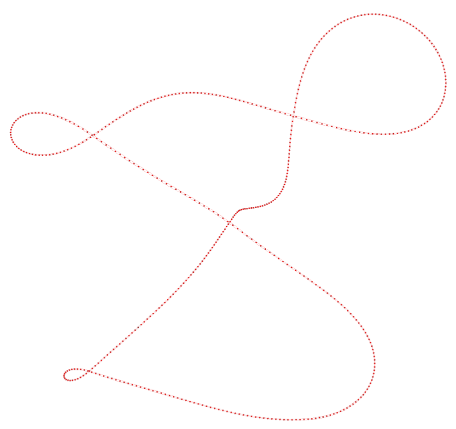		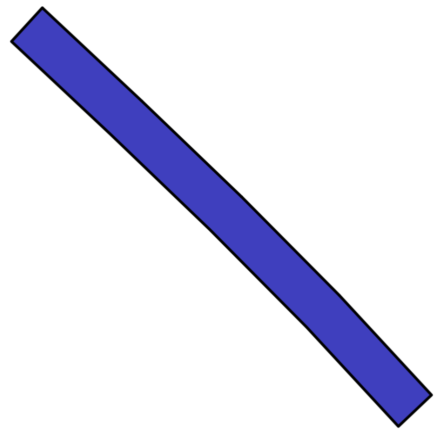
NCTC7414	*Staphylococcus aureus*	81	1	0	1	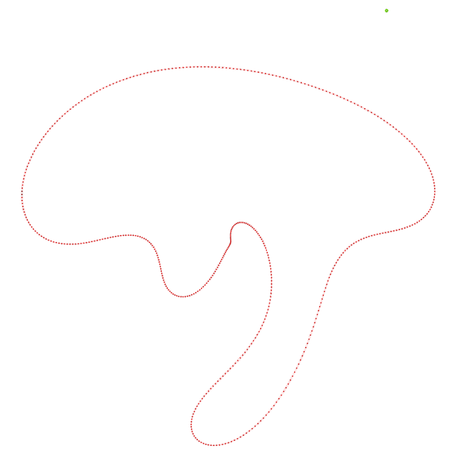	Finished circular assembly	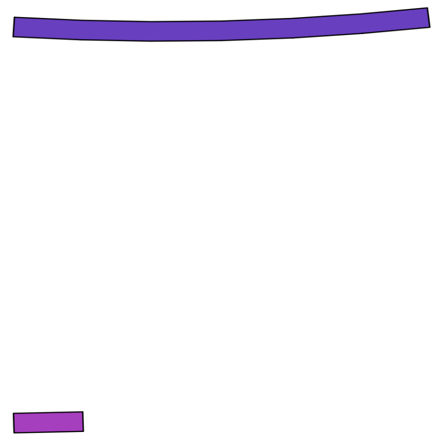
NCTC7415	*Staphylococcus aureus*	119	1	1	1	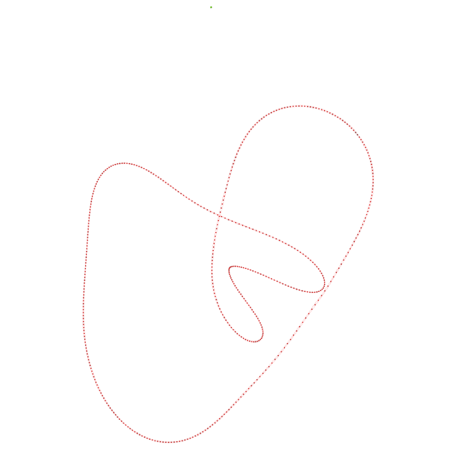	Finished circular assembly	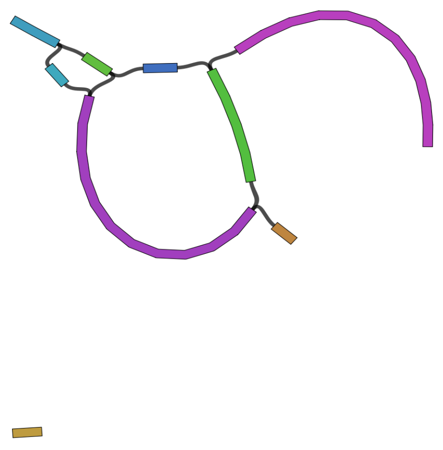
NCTC7427	*Klebsiella pneumoniae*	50	1	0	2	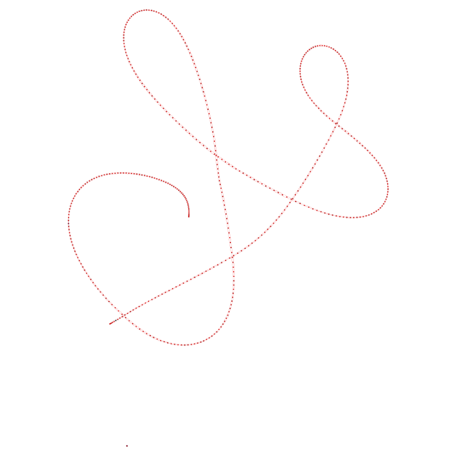	Finished assembly (lacking circularisation)	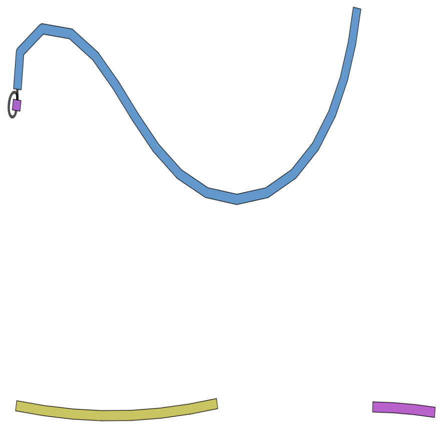
NCTC7428	*Staphylococcus aureus*	69	1	0	1	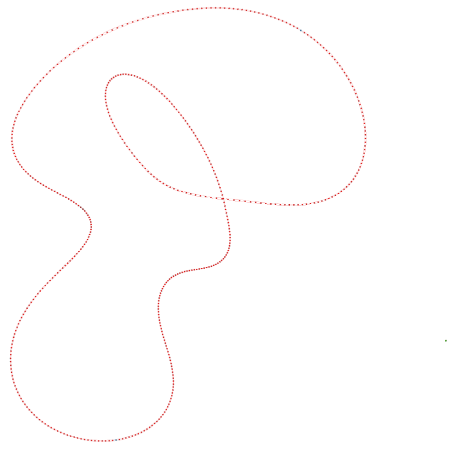	Finished circular assembly	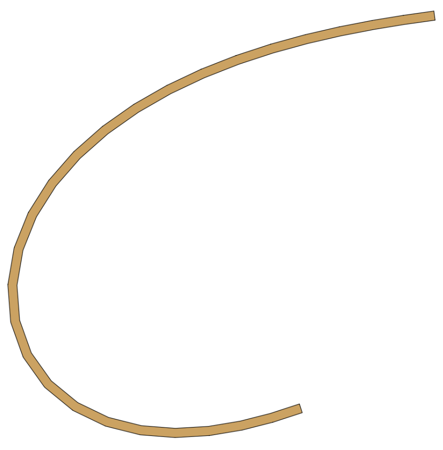
NCTC7445	*Staphylococcus aureus*	100	1	1	0	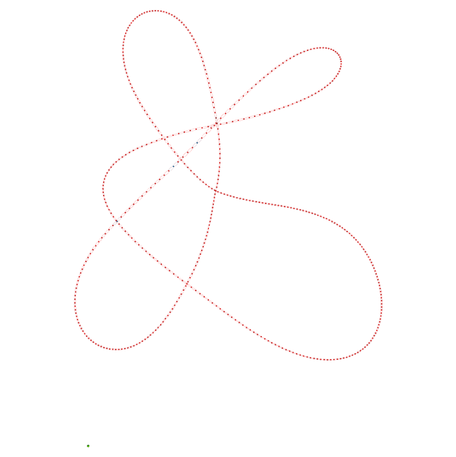	Finished circular assembly	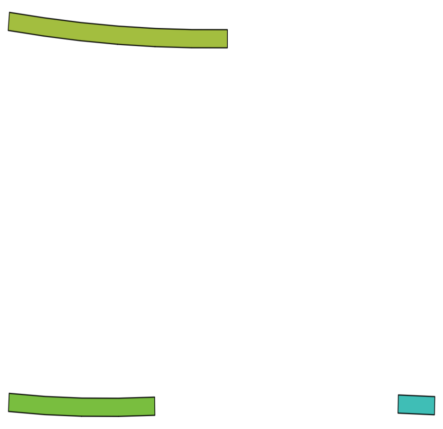
NCTC7446	*Staphylococcus aureus*	68	1	1	0		Finished circular assembly	

						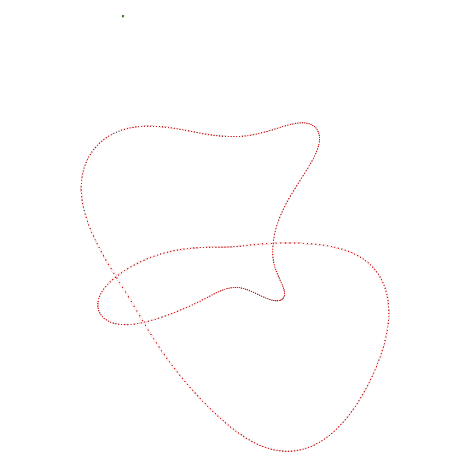		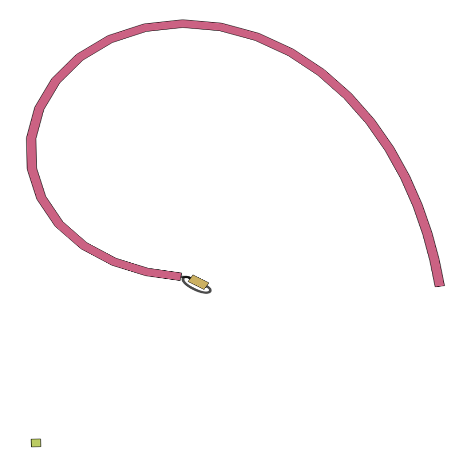
NCTC7447	*Staphylococcus aureus*	42	1	0	1	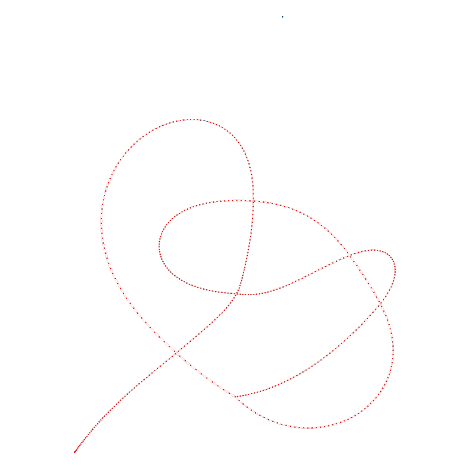	Finished assembly (lacking circularisation)	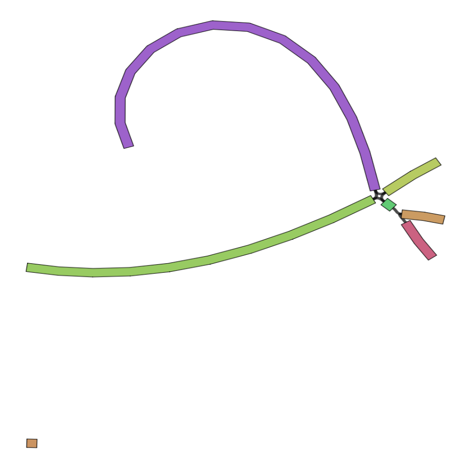
NCTC7465	*Streptococcus pneumoniae*	82	1	0	0	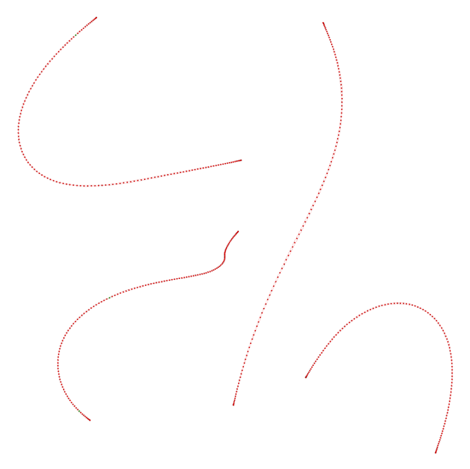	Mis-assembly/Fragmented	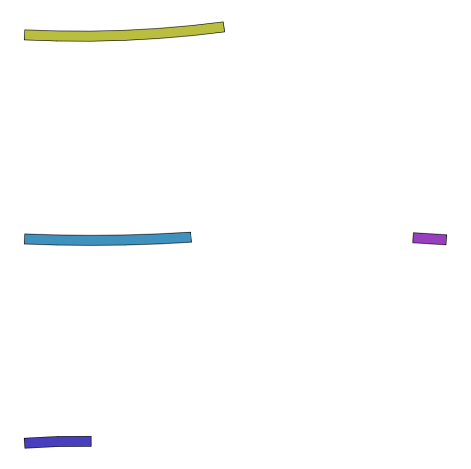
NCTC7474	*Staphylococcus epidermidis*	161	1	1	1	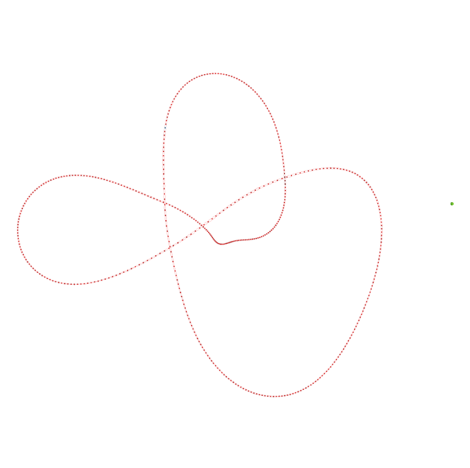	Finished circular assembly	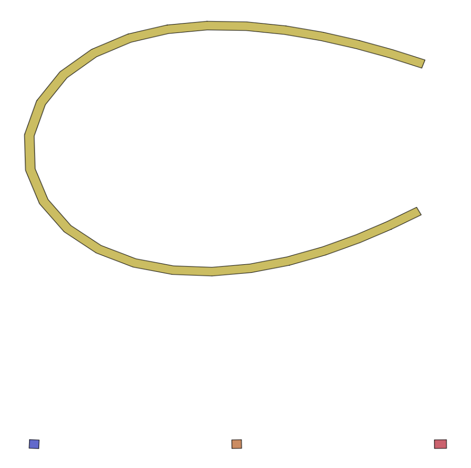
NCTC7485	*Staphylococcus aureus*	169	1	0	0	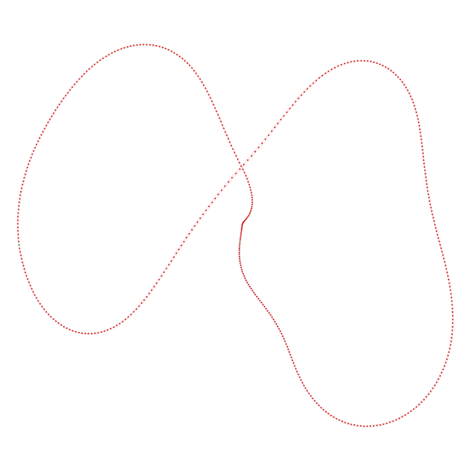	Finished circular assembly	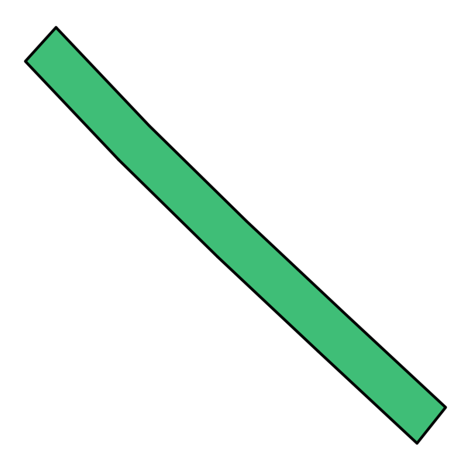
NCTC7486	*Staphylococcus sp.*	103	1	2	0	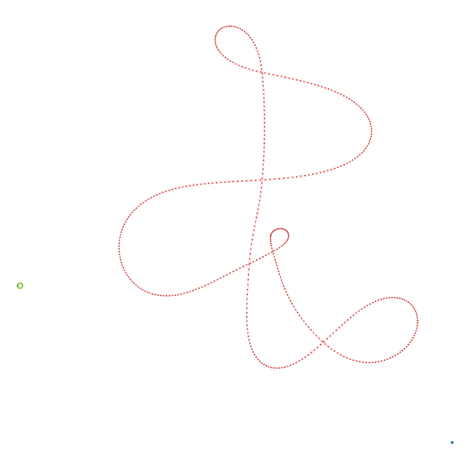	Finished circular assembly	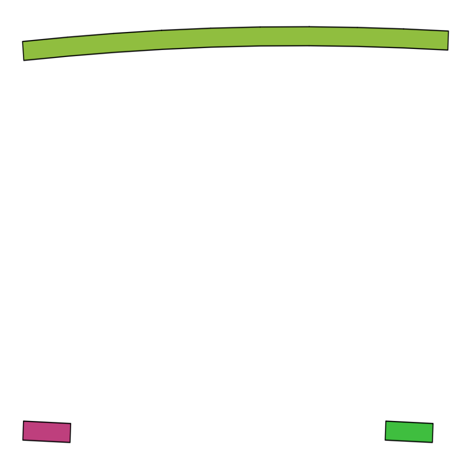
NCTC7526	*Staphylococcus saprophyticus subsp. saprophyticus*	146	Pending	Pending	Pending		Finished circular assembly	

						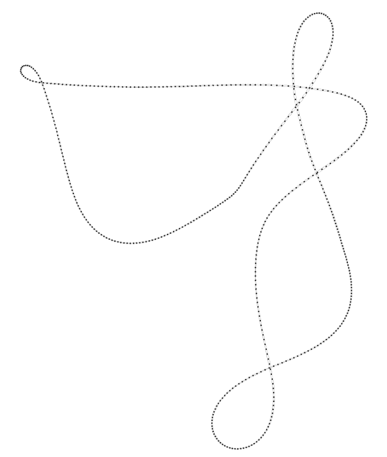		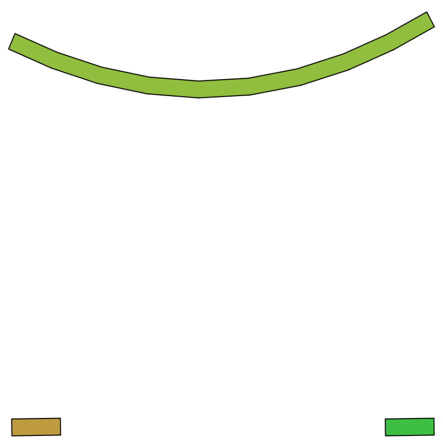
NCTC7540	*Staphylococcus saprophyticus*	172	1	3	0	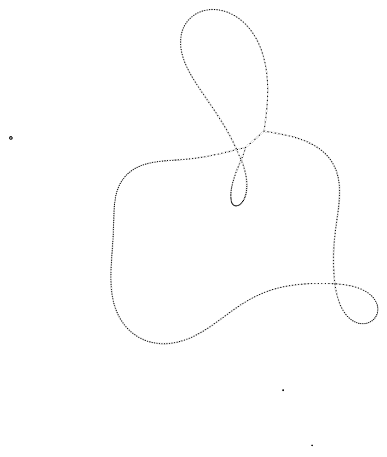	Finished assembly with multiple traversals	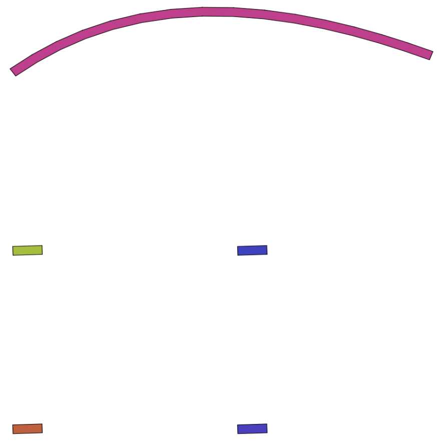
NCTC7666	*Staphylococcus saprophyticus*	107	1	0	0	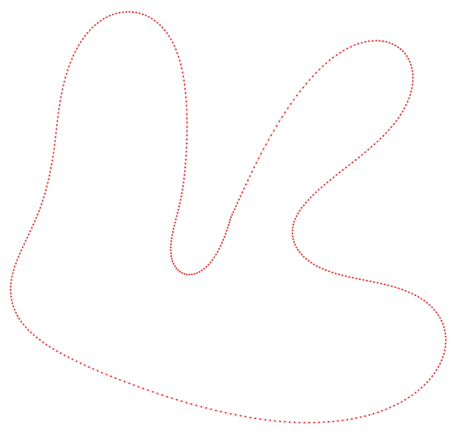	Finished circular assembly	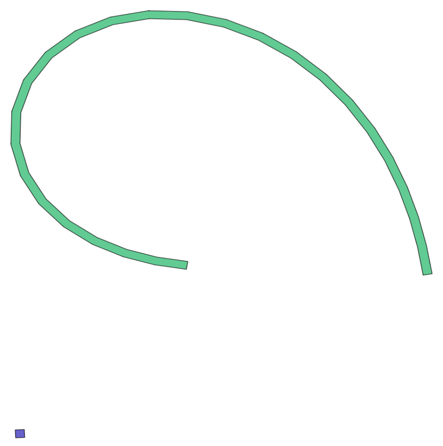
NCTC7687	*Staphylococcus saprophyticus*	115	0	0	14	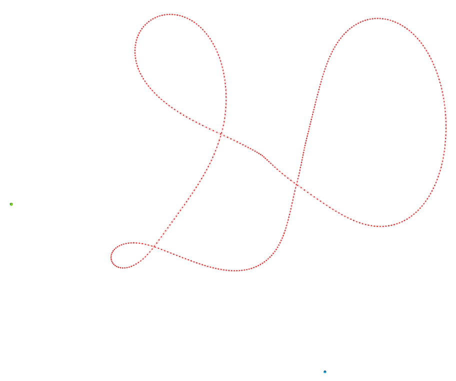	Finished circular assembly	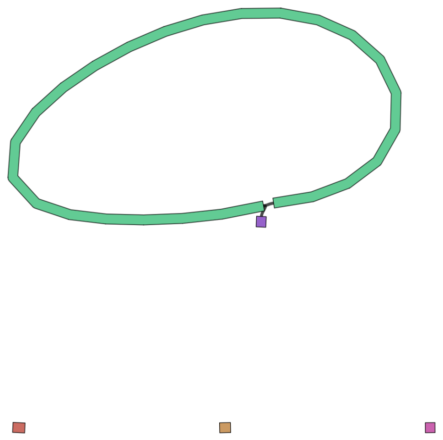
NCTC7692	*Staphylococcus saprophyticus*	122	1	1	1	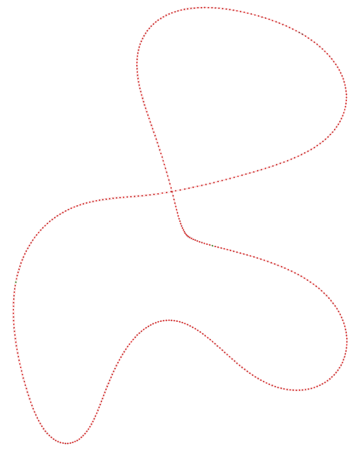	Finished circular assembly	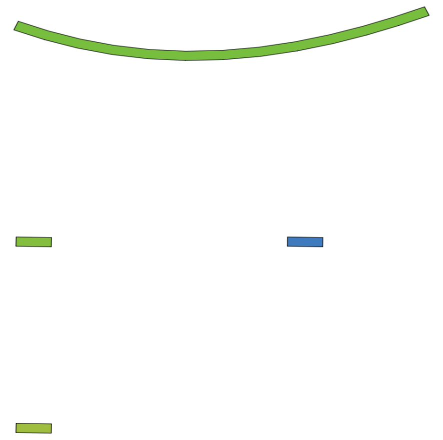
NCTC7712	*Staphylococcus aureus*	124	1	0	1	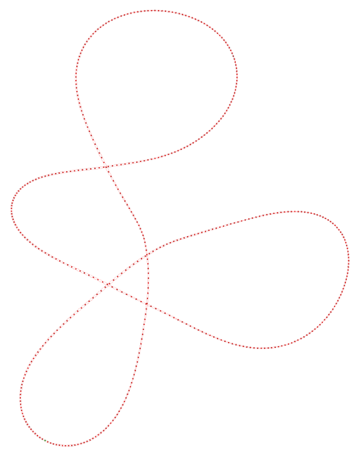	Finished circular assembly	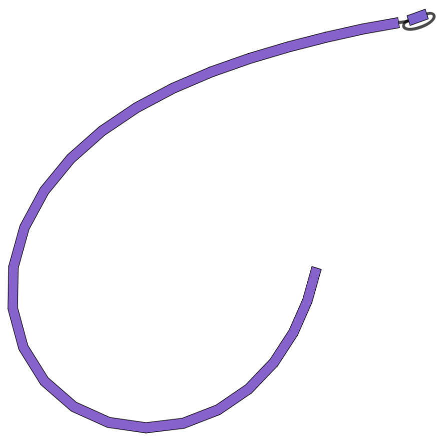
NCTC7718	*Staphylococcus sp.*	84	0	0	3		Finished circular assembly	

						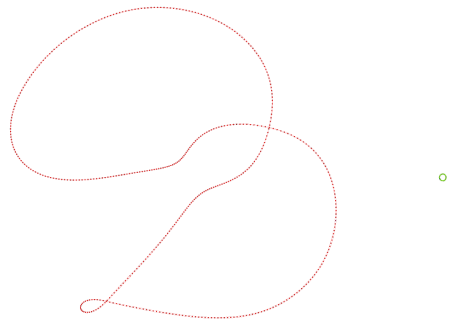		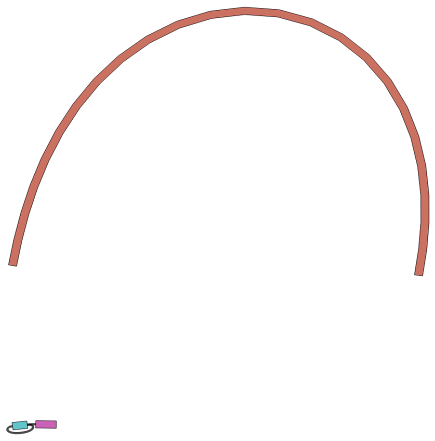
NCTC7791	*Staphylococcus aureus*	64	0	0	2	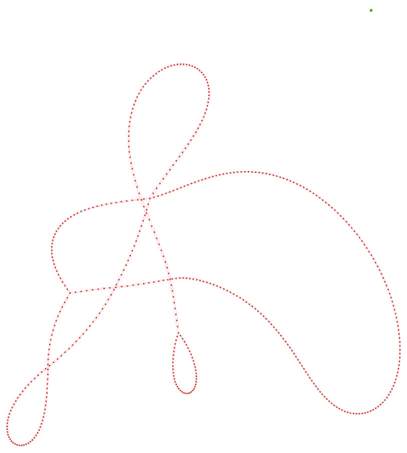	Mis-assembly/Fragmented	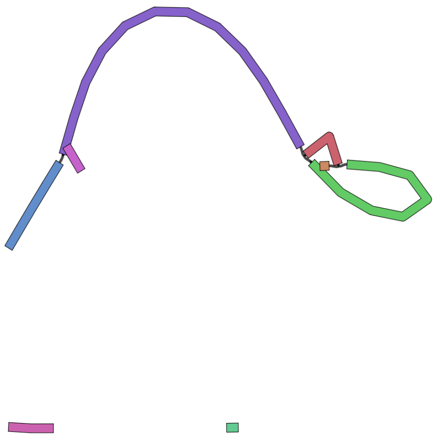
NCTC7799	*Klebsiella pneumoniae*	73	0	0	4	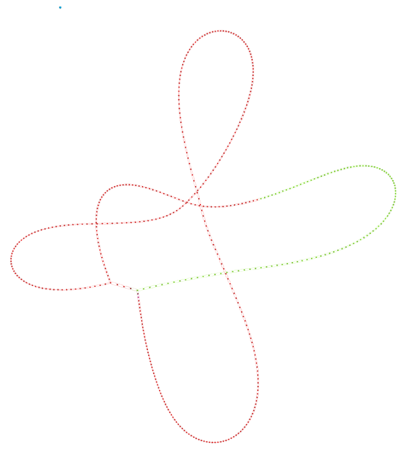	Finished assembly with multiple traversals	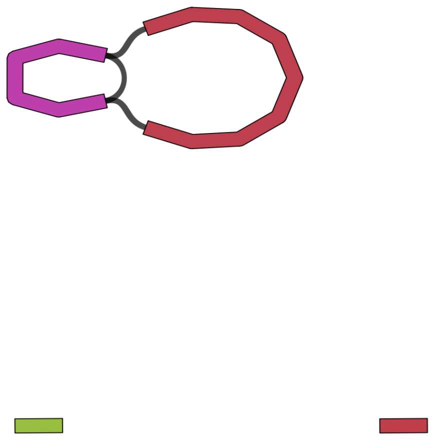
NCTC7802	*Staphylococcus saprophyticus*	152	0	0	2	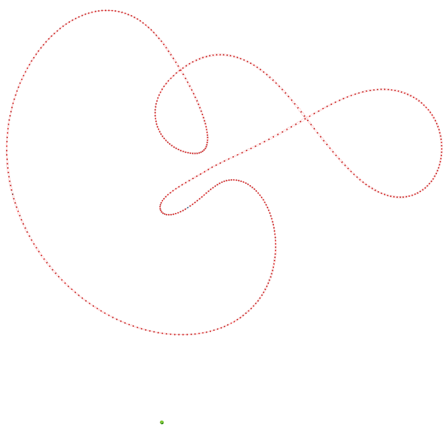	Finished circular assembly	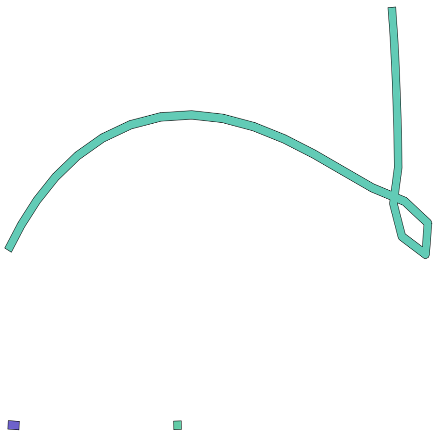
NCTC7831	*Salmonella enterica subsp. enterica*	38	0	0	1	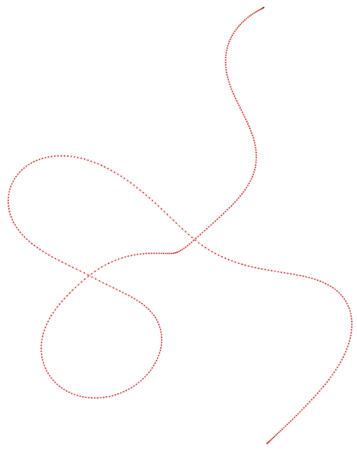	Finished assembly (lacking circularisation)	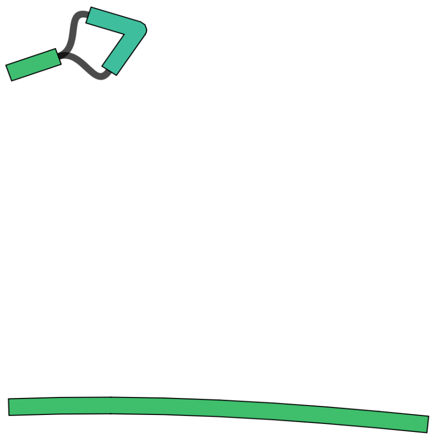
NCTC7832	*Salmonella enterica subsp. enterica*	37	1	0	2	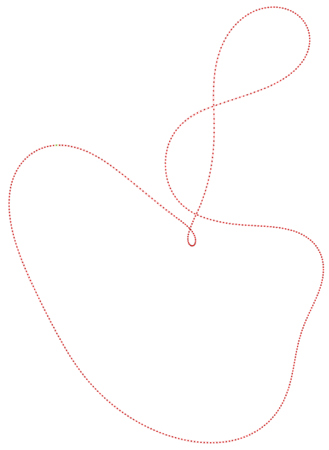	Finished circular assembly	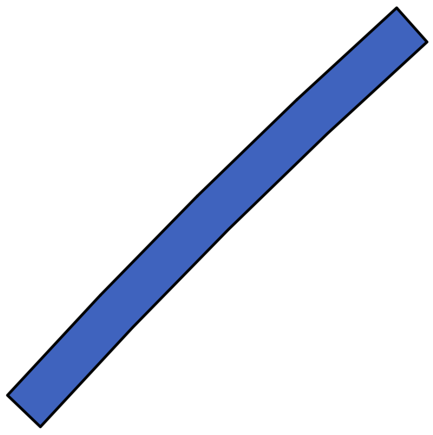
NCTC7836	*Salmonella enterica subsp. enterica*	32	1	0	0		Finished circular assembly	

						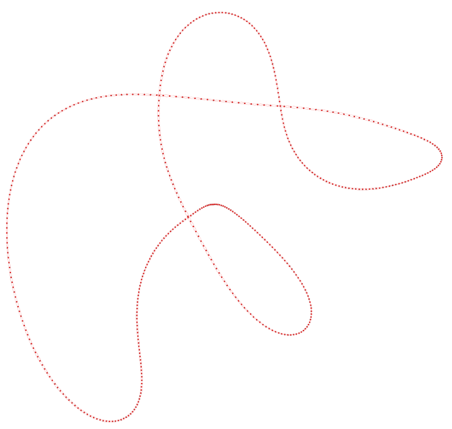		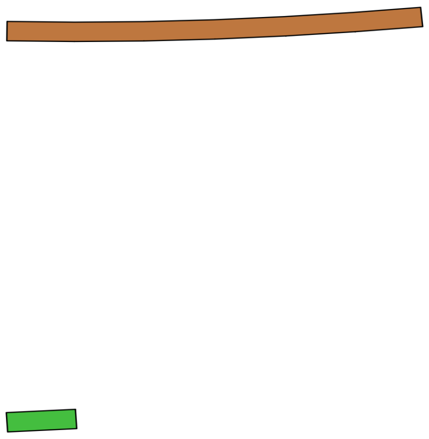
NCTC7856	*Staphylococcus aureus*	66	1	1	0	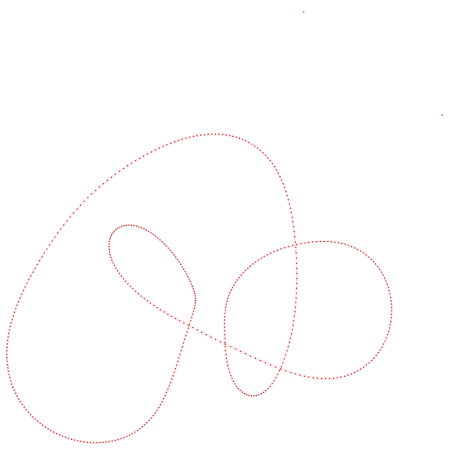	Finished circular assembly	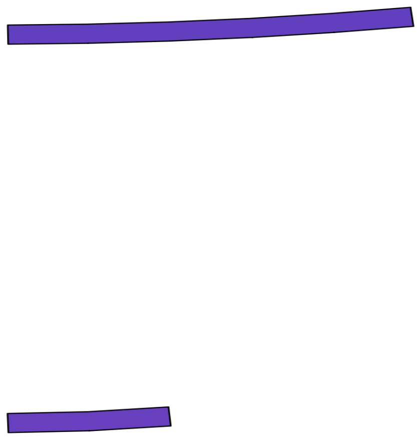
NCTC7865	*Streptococcus gordonii*	137	1	0	0	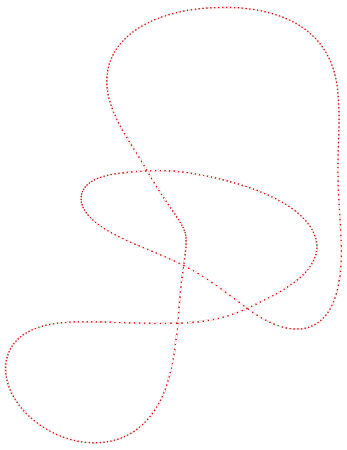	Finished circular assembly	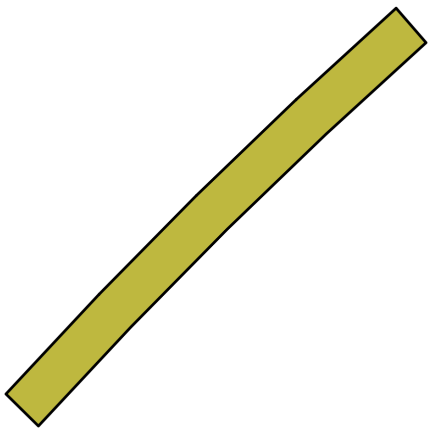
NCTC7868	*Streptococcus gordonii*	172	Pending	Pending	Pending	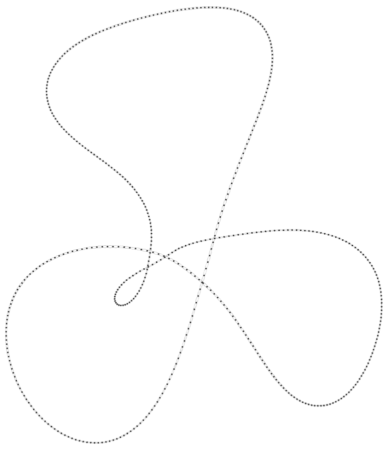	Finished circular assembly	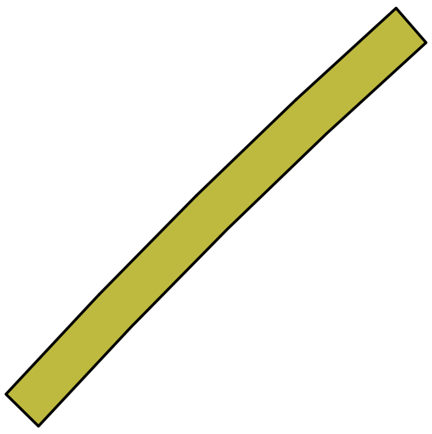
NCTC7869	*Streptococcus sp.*	22	Pending	Pending	Pending	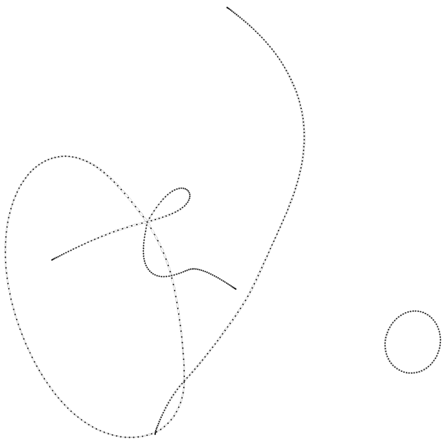	Mis-assembly/Fragmented	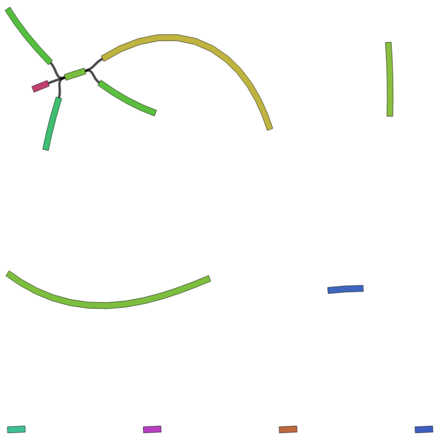
NCTC7878	*Staphylococcus aureus*	27	Pending	Pending	Pending	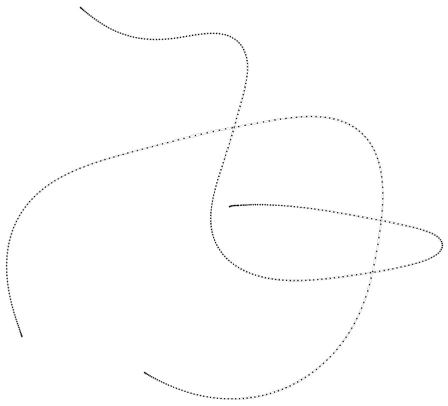	Mis-assembly/Fragmented	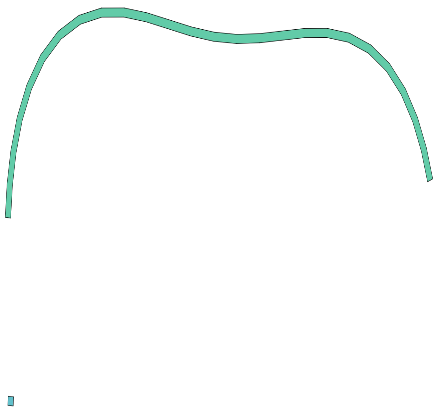
NCTC7887	*Staphylococcus aureus*	45	1	0	1		Finished circular assembly	

						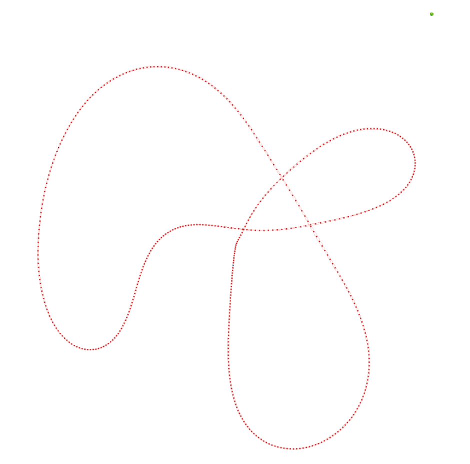		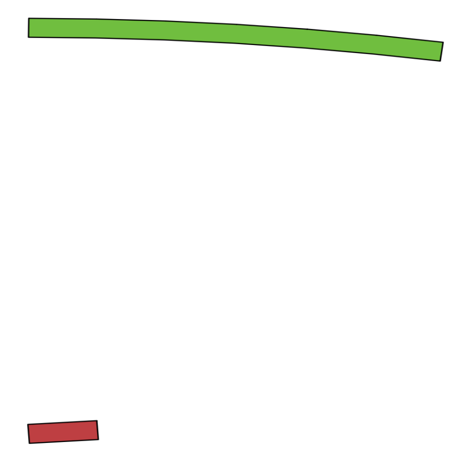
NCTC7921	*Escherichia coli*	43	0	0	7	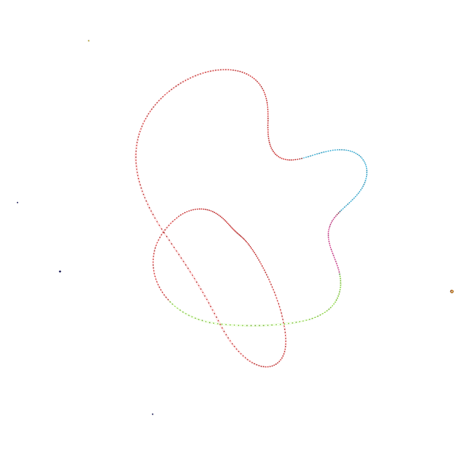	Finished circular assembly	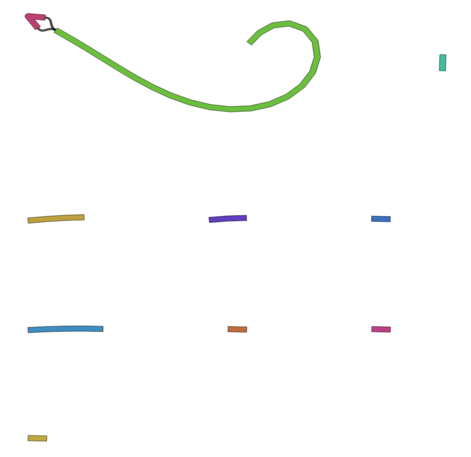
NCTC7922	*Escherichia coli*	26	0	0	6	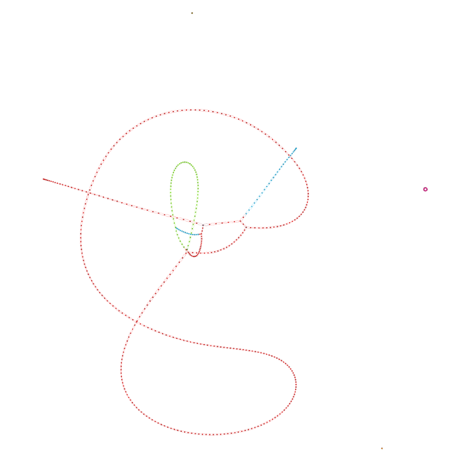	Mis-assembly/Fragmented	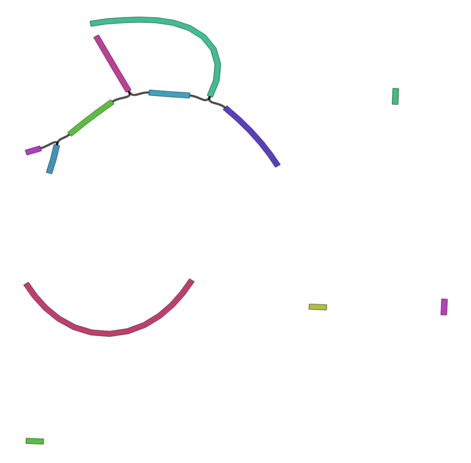
NCTC7926	*Escherichia coli*	45	0	0	3	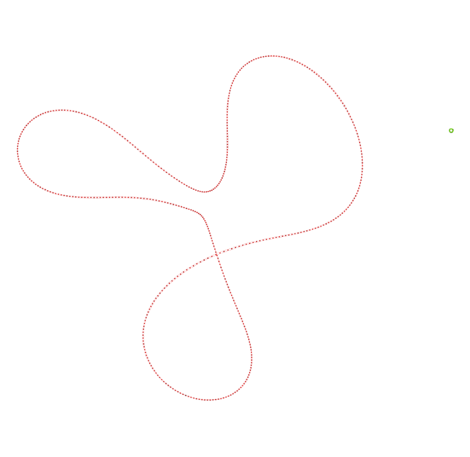	Finished circular assembly	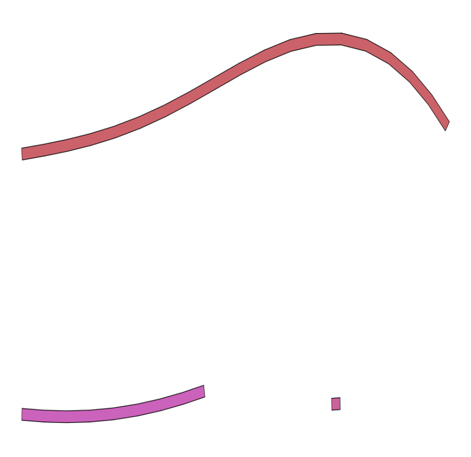
NCTC7927	*Escherichia coli*	47	1	1	3	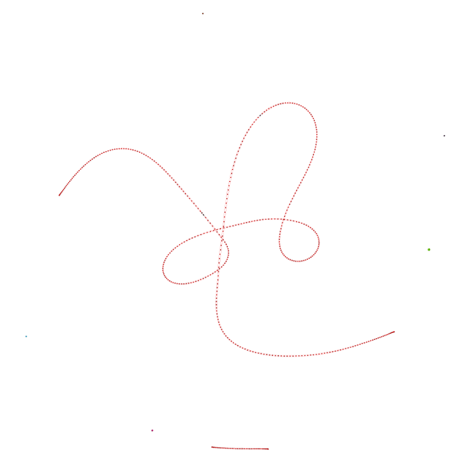	Finished assembly (lacking circularisation)	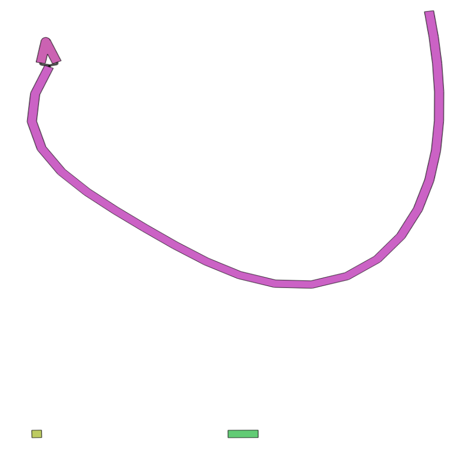
NCTC7928	*Escherichia coli*	30	Pending	Pending	Pending	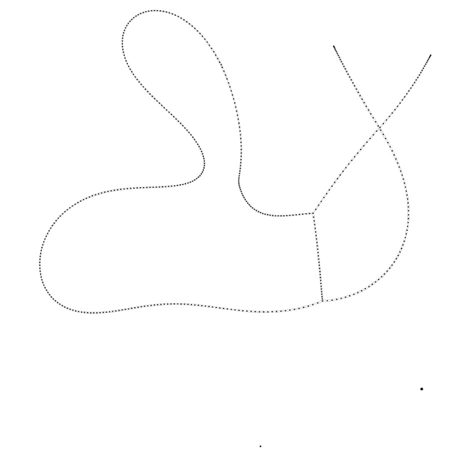	Mis-assembly/Fragmented	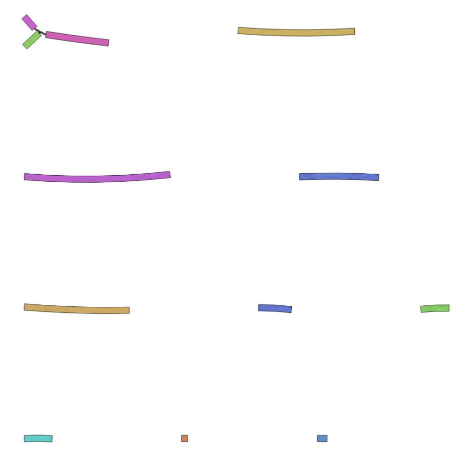
NCTC7944	*Staphylococcus hyicus*	82	1	0	0		Finished circular assembly	

						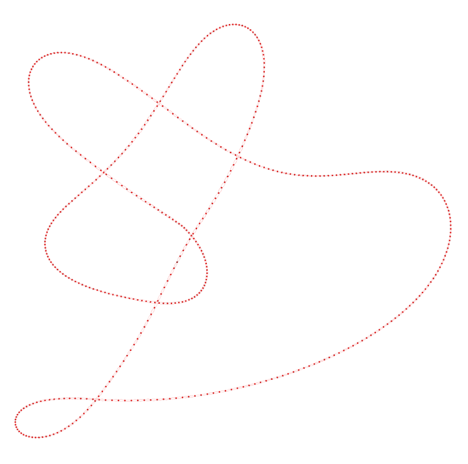		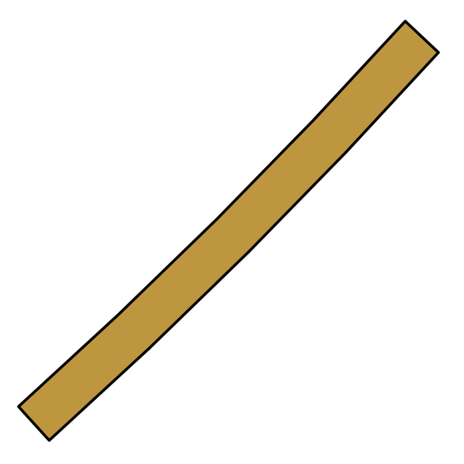
NCTC7972	*Staphylococcus aureus*	108	0	0	7	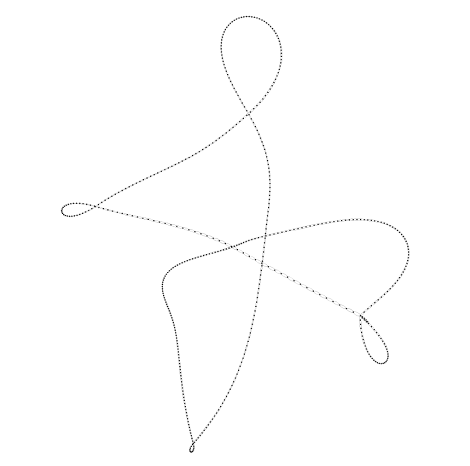	Finished assembly with multiple traversals	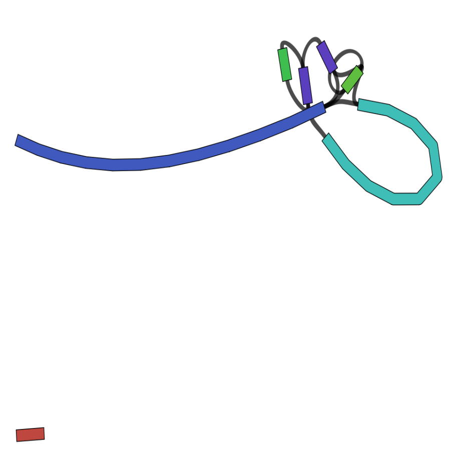
NCTC7982	*Streptococcus sp.*	70	Pending	Pending	Pending	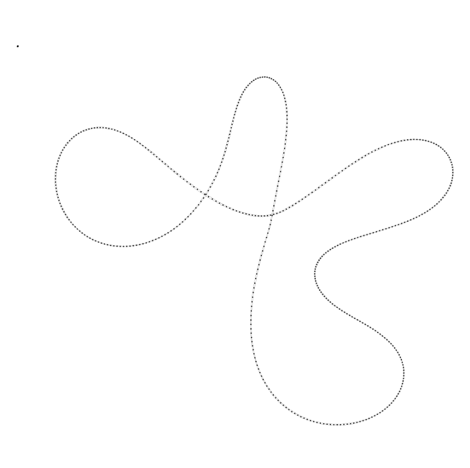	Finished circular assembly	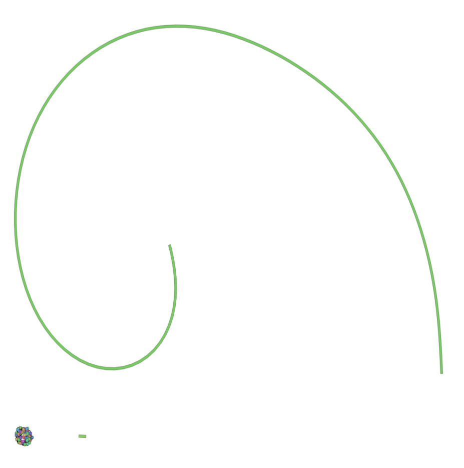
NCTC7988	*Staphylococcus aureus*	87	Pending	Pending	Pending	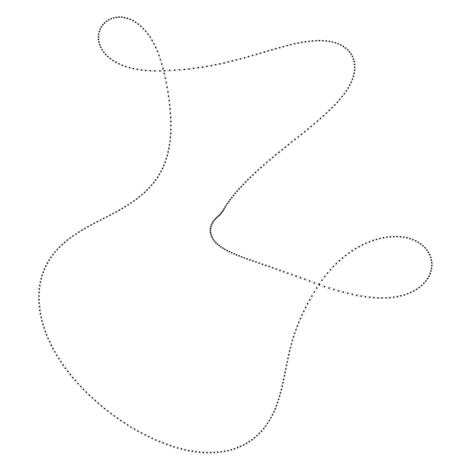	Finished circular assembly	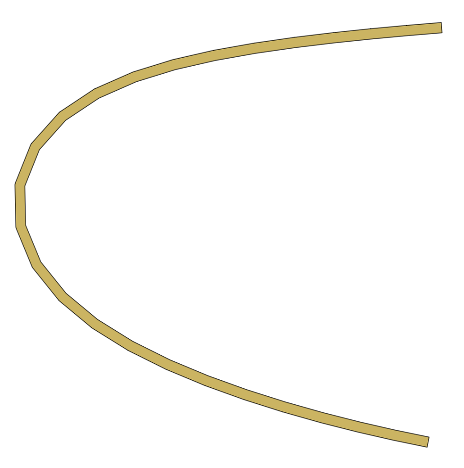
NCTC7990	*Staphylococcus lugdunensis*	166	1	0	0	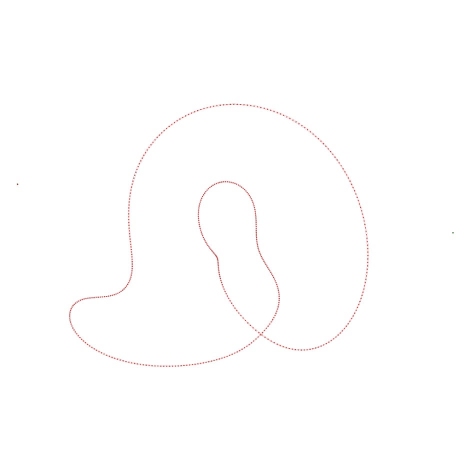	Finished circular assembly	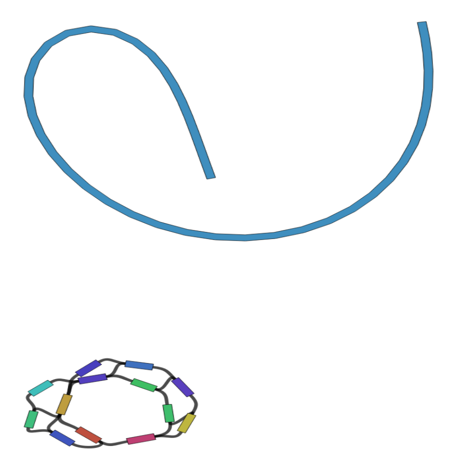
NCTC8003	*Escherichia coli*	49	0	0	4	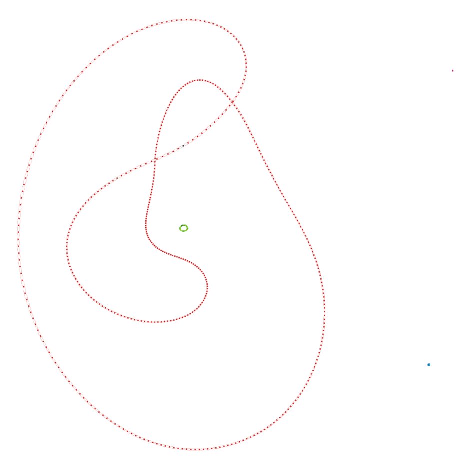	Finished circular assembly	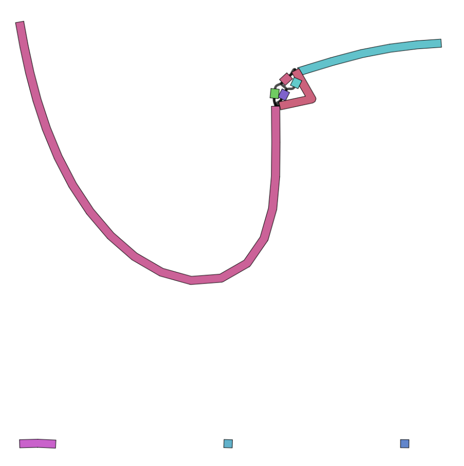
NCTC8004	*Staphylococcus aureus*	112	1	0	2		Finished circular assembly	

						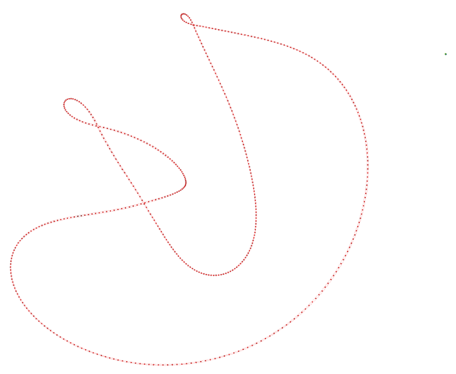		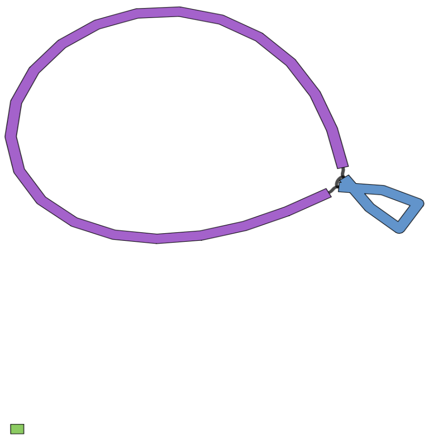
NCTC8007	*Escherichia coli*	63	0	0	12	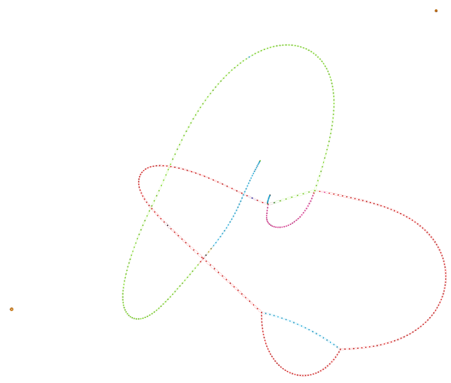	Mis-assembly/Fragmented	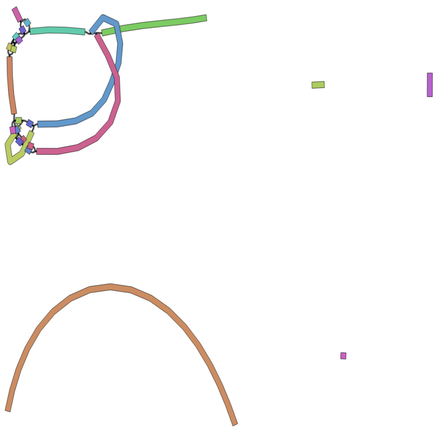
NCTC8008	*Escherichia coli*	86	1	1	2	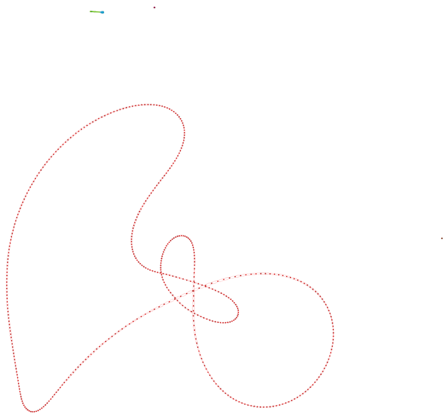	Finished circular assembly	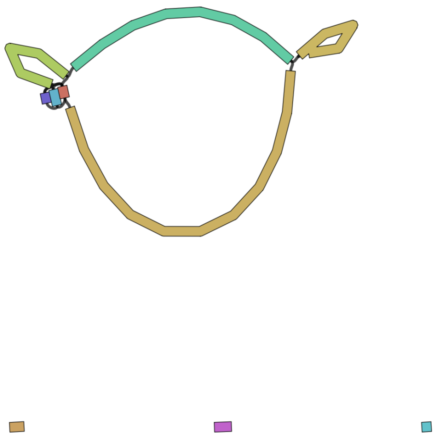
NCTC8009	*Escherichia coli*	67	0	0	11	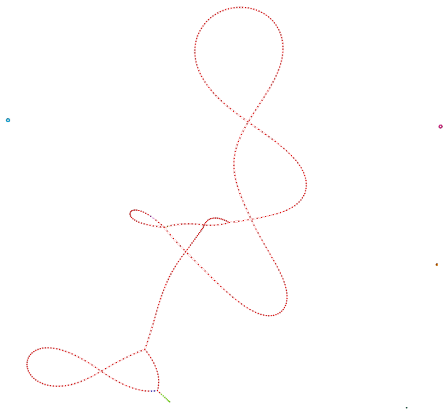	Mis-assembly/Fragmented	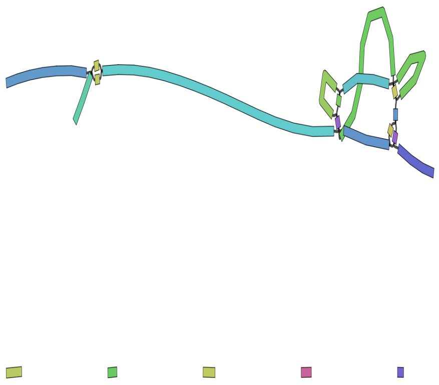
NCTC8017	*Streptococcus agalactiae*	93	Pending	Pending	Pending	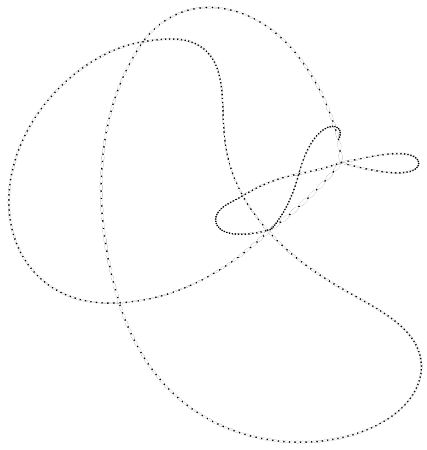	Possible mis-assembly	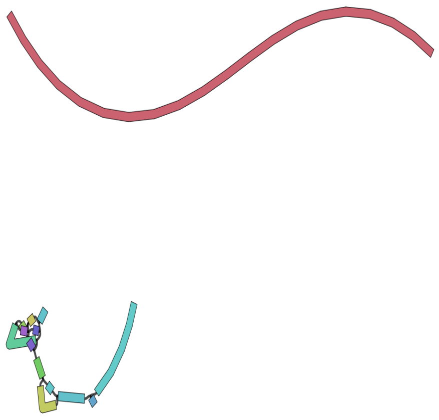
NCTC8100	*Streptococcus agalactiae*	70	Pending	Pending	Pending	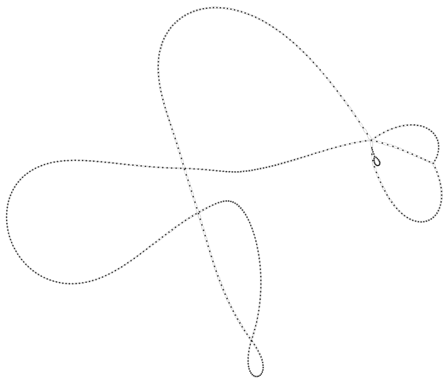	Mis-assembly/Fragmented	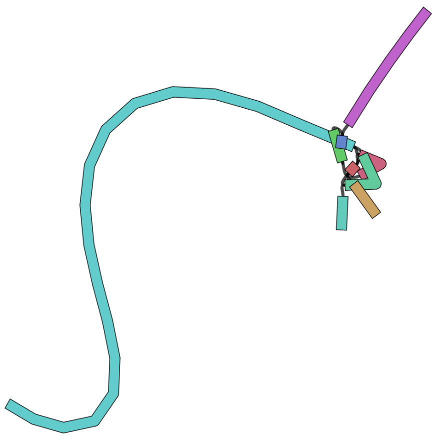
NCTC8105	*Hafnia alvei*	53	1	1	0		Finished circular assembly	

						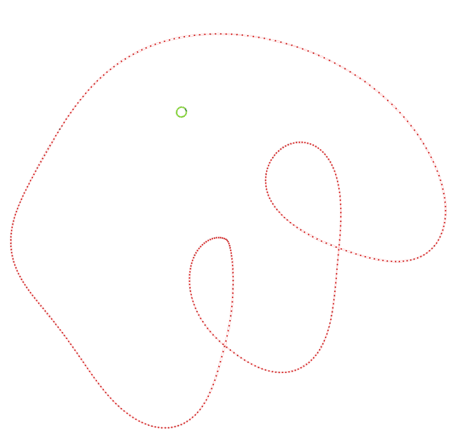		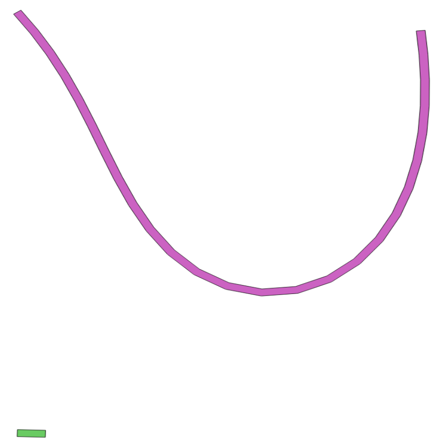
NCTC8133	*Streptococcus equinus*	33	Pending	Pending	Pending	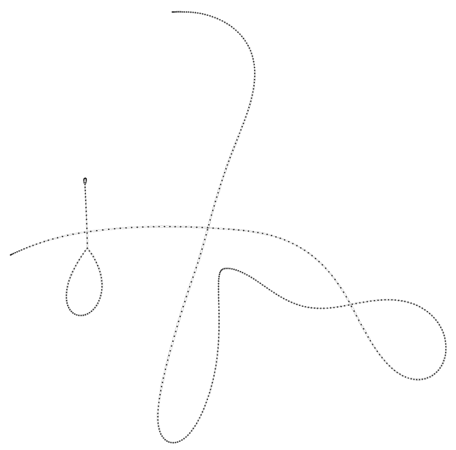	Mis-assembly/Fragmented	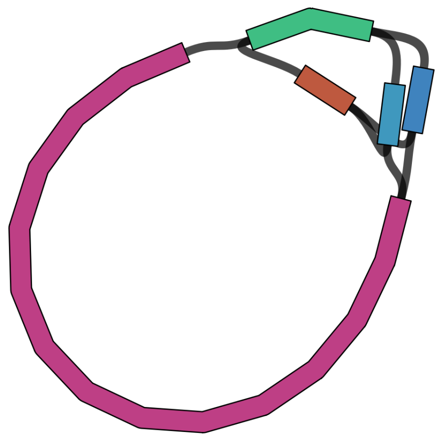
NCTC8140	*Streptococcus equinus*	45	Pending	Pending	Pending	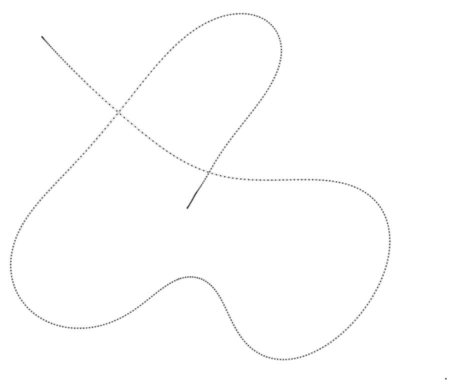	Finished assembly (lacking circularisation)	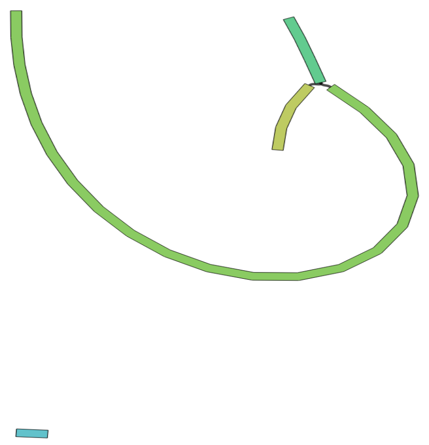
NCTC8143	*Haemophilus influenzae*	96	1	0	0	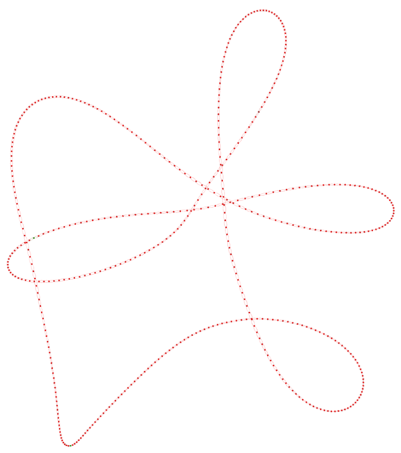	Finished circular assembly	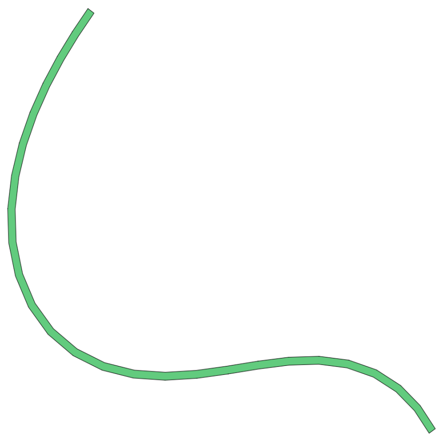
NCTC8159	*Mycobacterium smegmatis*	40	1	0	0	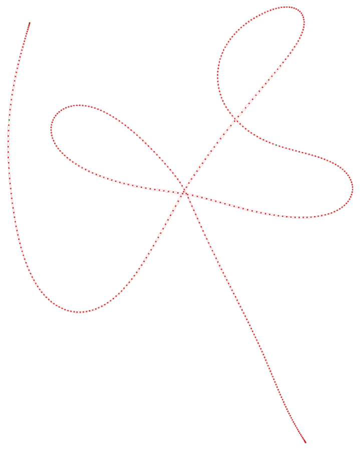	Finished assembly (lacking circularisation)	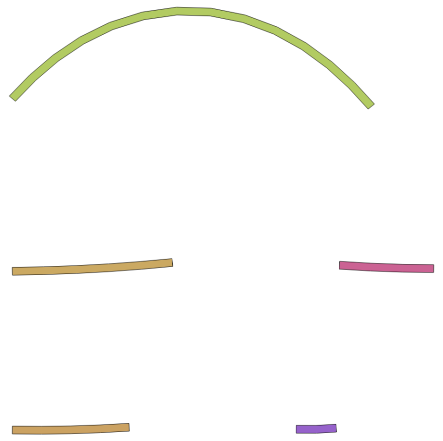
NCTC8167	*Klebsiella oxytoca*	42	0	0	3	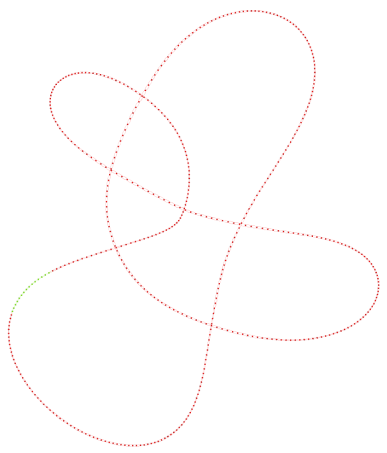	Finished circular assembly	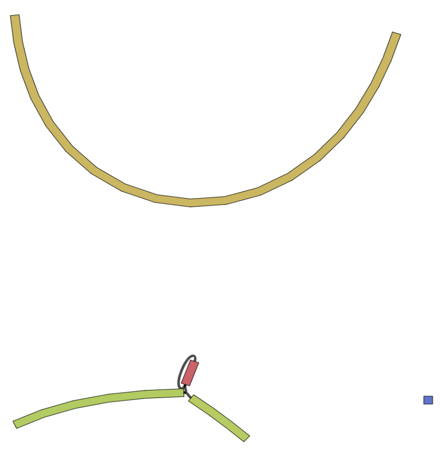
NCTC8172	*Klebsiella pneumoniae*	50	1	1	0		Finished circular assembly	

						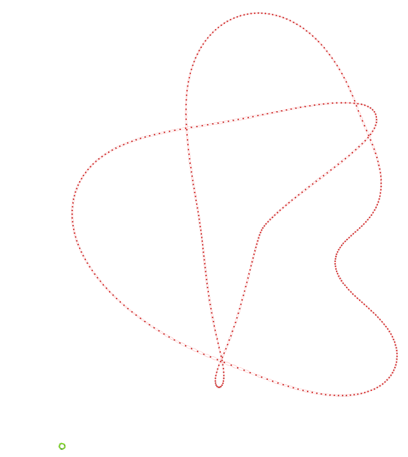		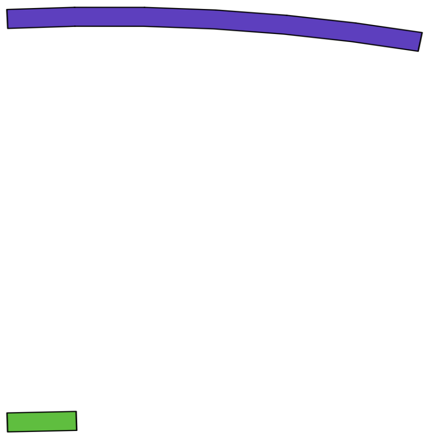
NCTC8178	*Staphylococcus aureus*	58	0	1	0	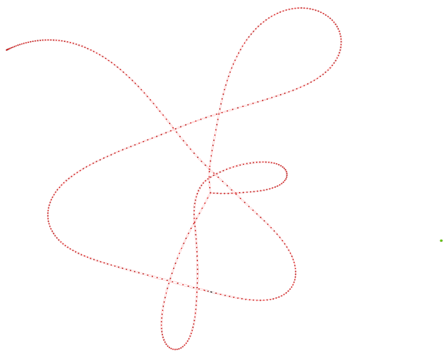	Mis-assembly/Fragmented	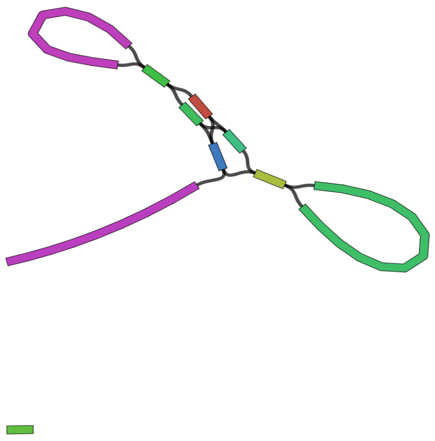
NCTC8179	*Escherichia coli*	36	1	1	3	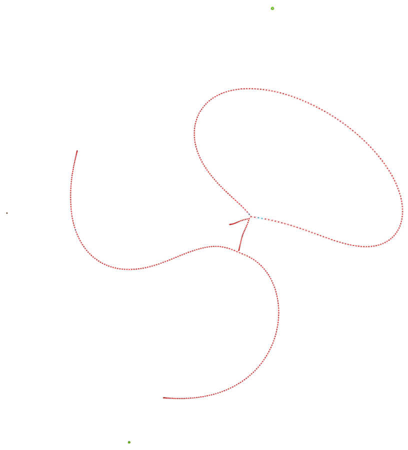	Mis-assembly/Fragmented	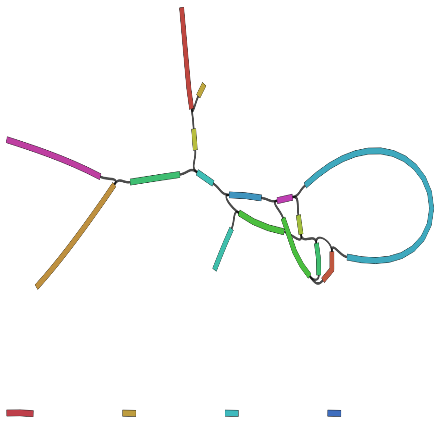
NCTC8184	*Streptococcus agalactiae*	91	0	0	1	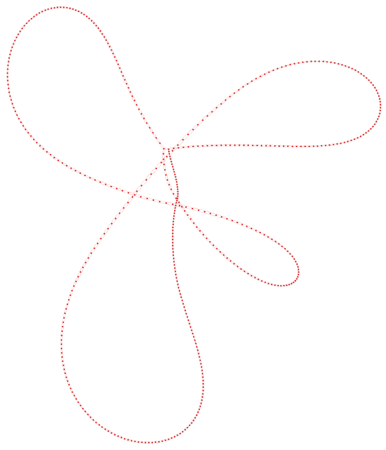	Finished assembly with multiple traversals	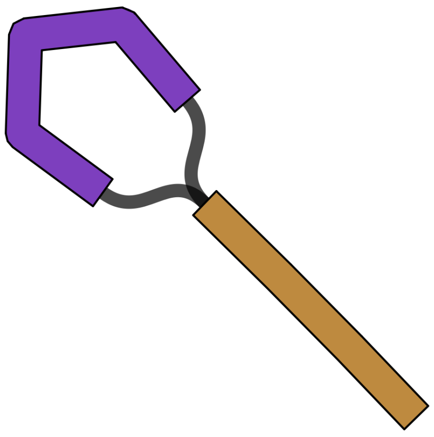
NCTC8188	*Streptococcus agalactiae*	183	1	1	3	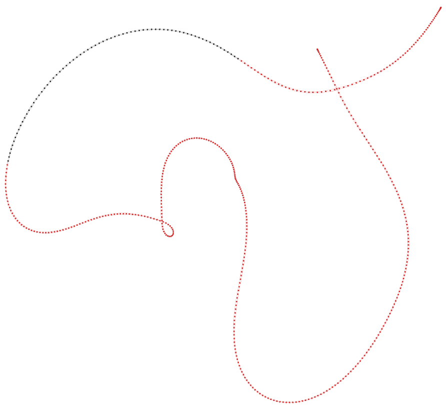	Finished assembly (lacking circularisation)	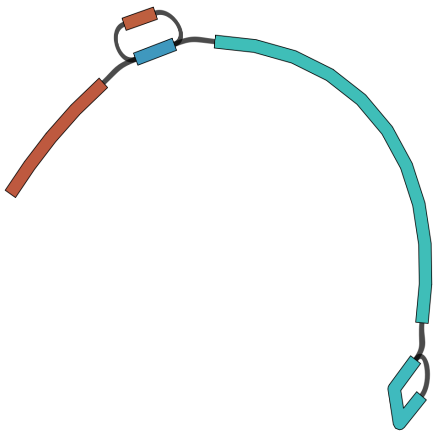
NCTC8193	*Streptococcus pyogenes*	101	0	0	1	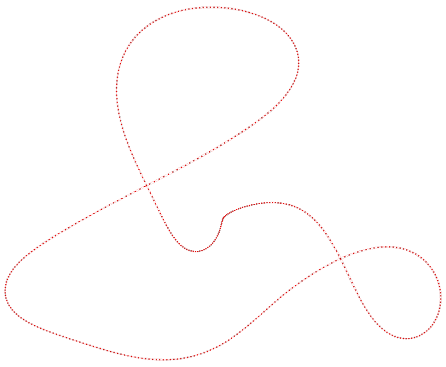	Finished circular assembly	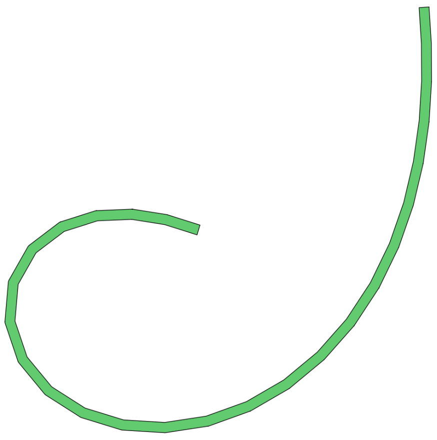
NCTC8195	*Streptococcus pyogenes*	151	Pending	Pending	Pending		Finished circular assembly	

						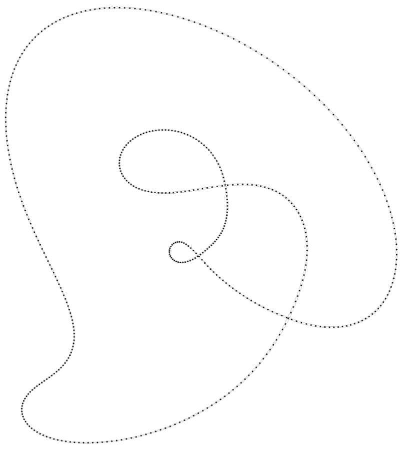		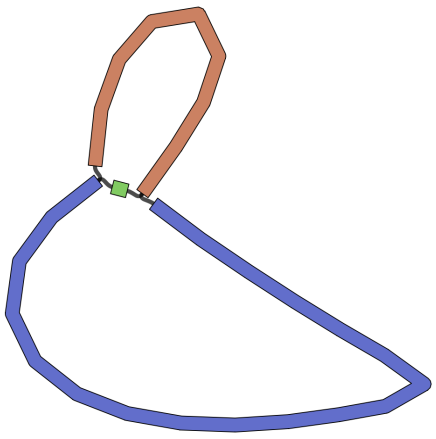
NCTC8196	*Escherichia coli*	97	1	0	0	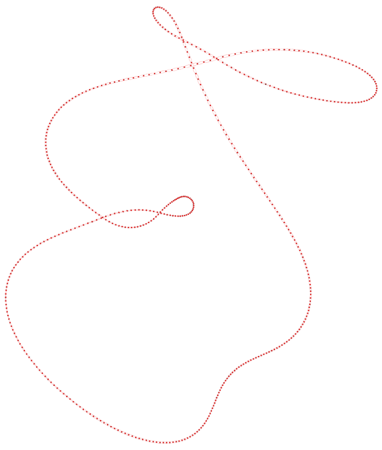	Finished circular assembly	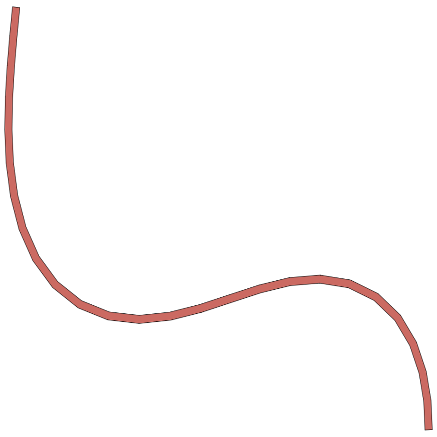
NCTC8198	*Streptococcus pyogenes*	129	1	0	0	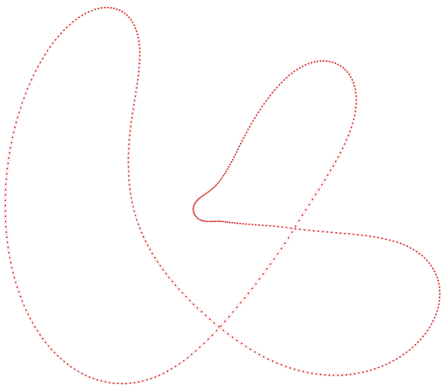	Finished circular assembly	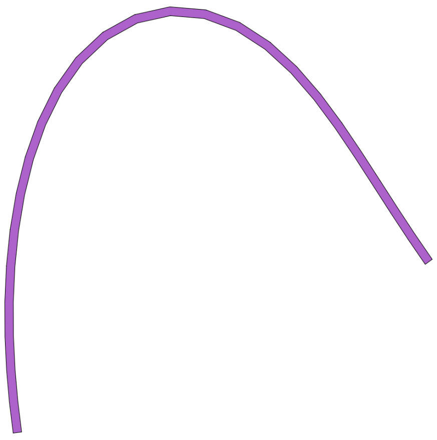
NCTC8230	*Streptococcus pyogenes*	77	Pending	Pending	Pending	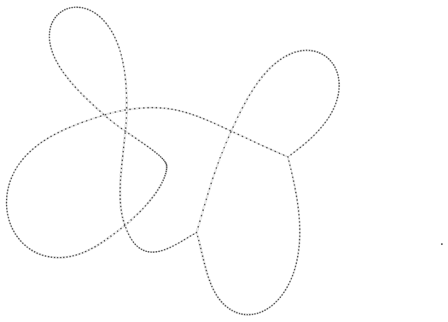	Mis-assembly/Fragmented	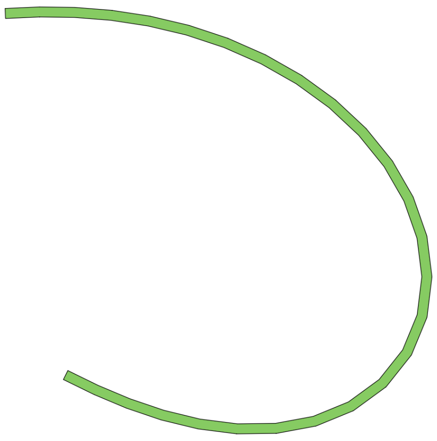
NCTC8231	*Streptococcus pyogenes*	213	1	0	0	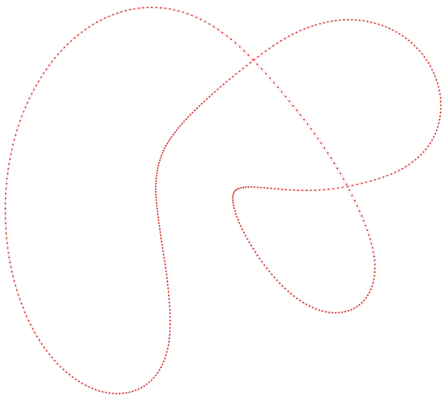	Finished circular assembly	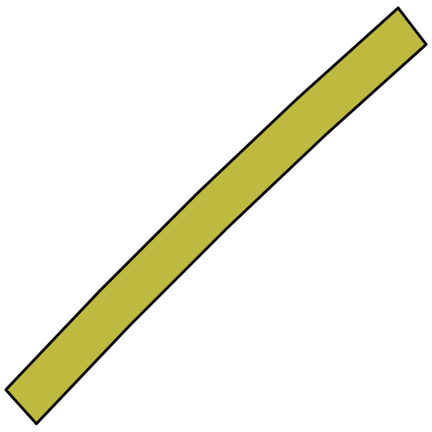
NCTC8232	*Streptococcus pyogenes*	168	Pending	Pending	Pending	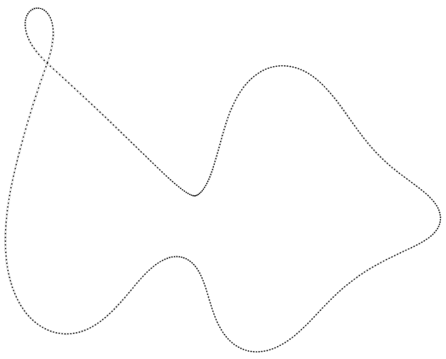	Finished circular assembly	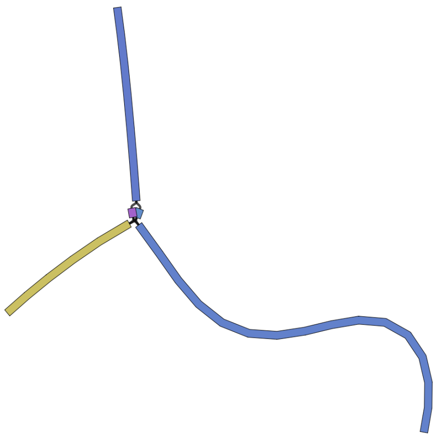
NCTC8256	*Salmonella enterica subsp. enterica*	61	1	0	1		Finished circular assembly	

						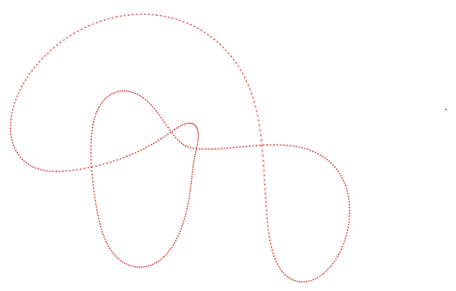		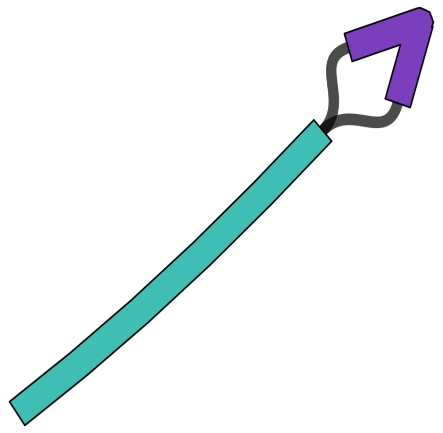
NCTC8258	*Salmonella enterica subsp. enterica*	26	1	1	6	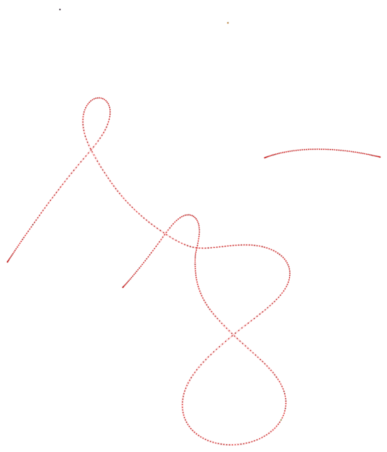	Finished assembly (lacking circularisation)	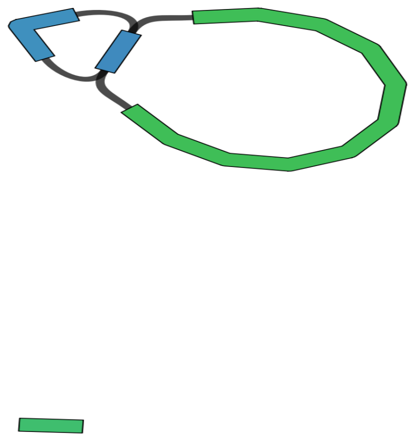
NCTC8261	*Salmonella enterica subsp. enterica*	30	1	0	0	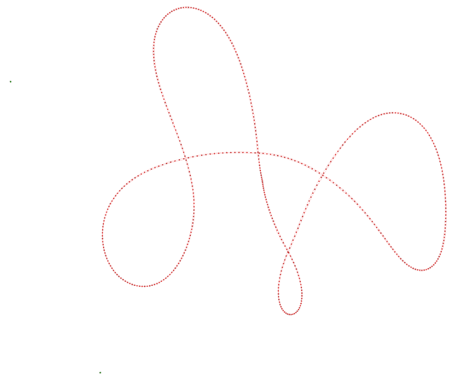	Finished circular assembly	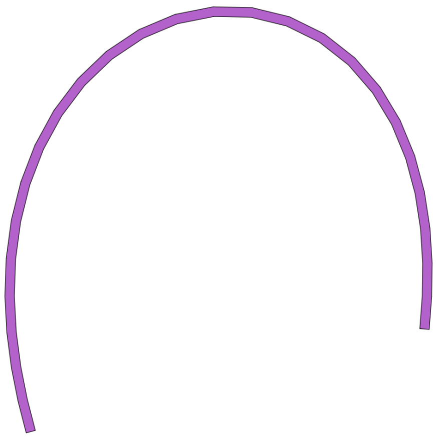
NCTC8267	*Salmonella enterica subsp. enterica*	59	1	0	0	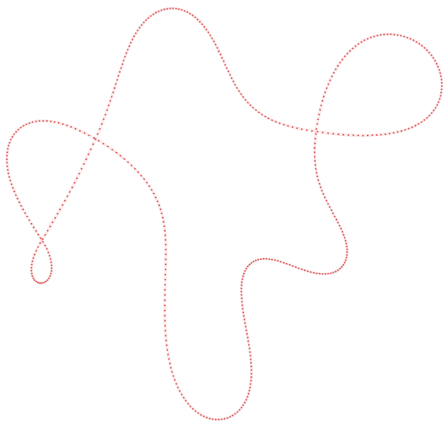	Finished circular assembly	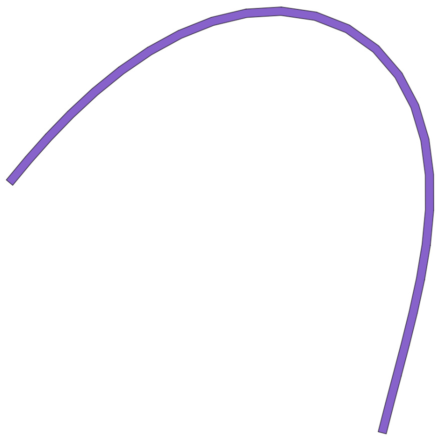
NCTC8270	*Salmonella enterica subsp. enterica*	64	0	0	3	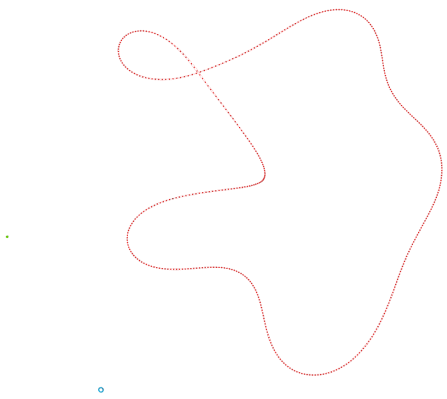	Finished circular assembly	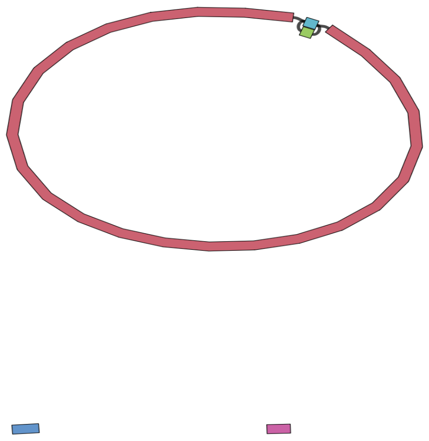
NCTC8271	*Salmonella enterica subsp. enterica*	64	1	0	0	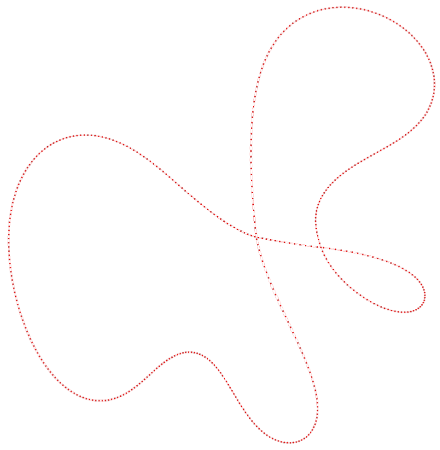	Finished circular assembly	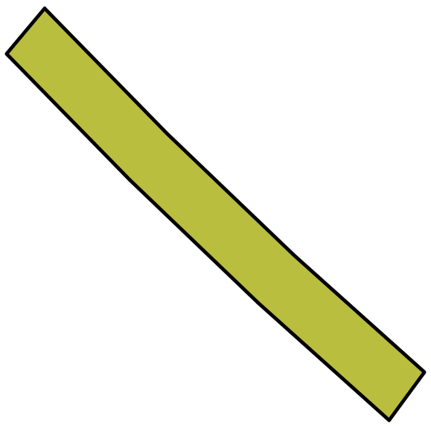
NCTC8272	*Salmonella enterica subsp. enterica*	40	0	0	1		Finished circular assembly	

						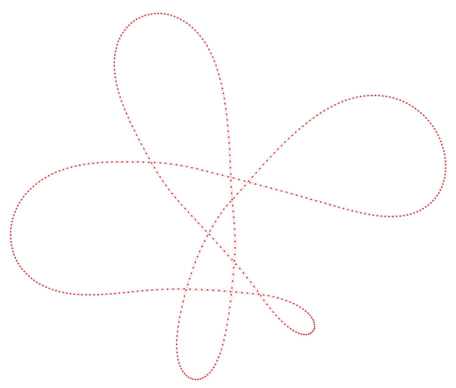		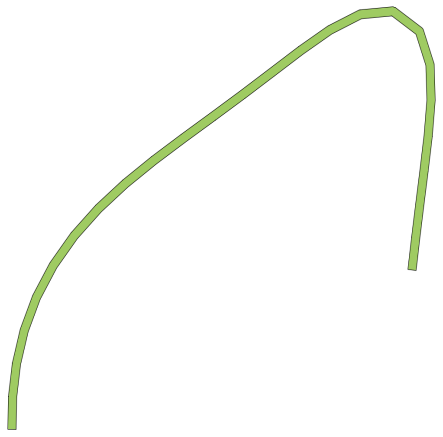
NCTC8273	*Salmonella enterica subsp. salamae*	93	1	0	0	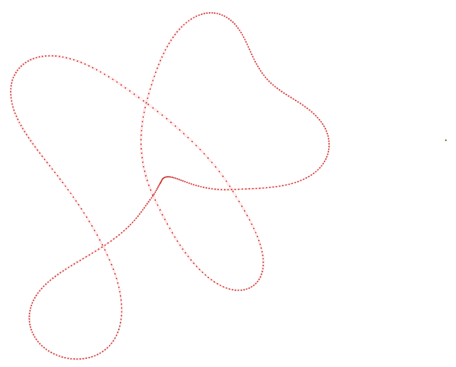	Finished circular assembly	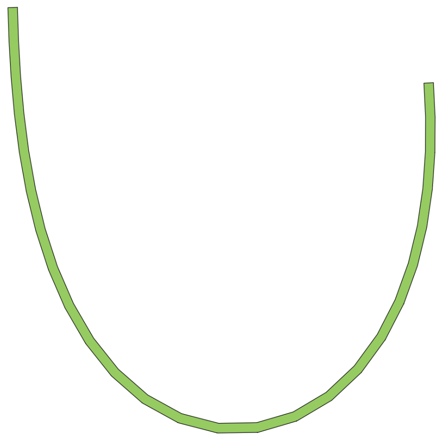
NCTC8294	*Staphylococcus sp.*	144	1	1	0	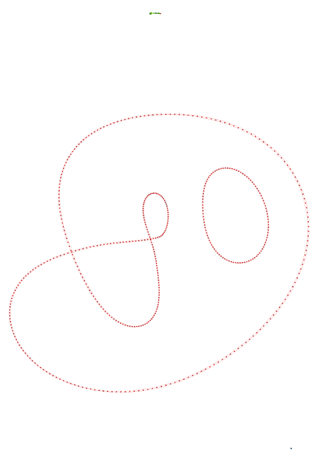	Finished circular assembly	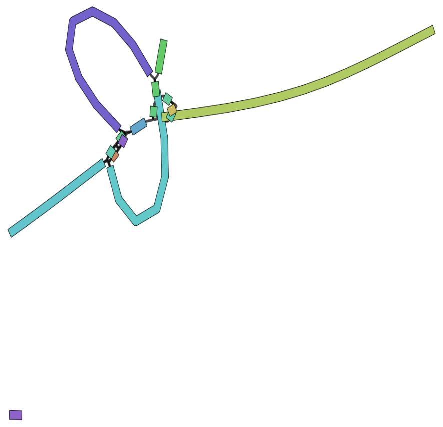
NCTC8297	*Salmonella enterica subsp. arizonae*	42	1	1	0	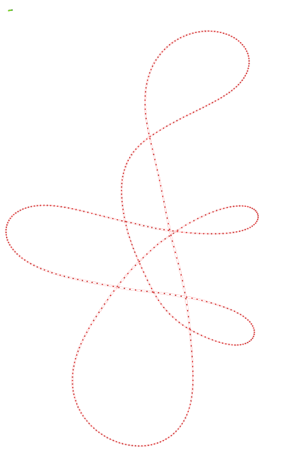	Finished circular assembly	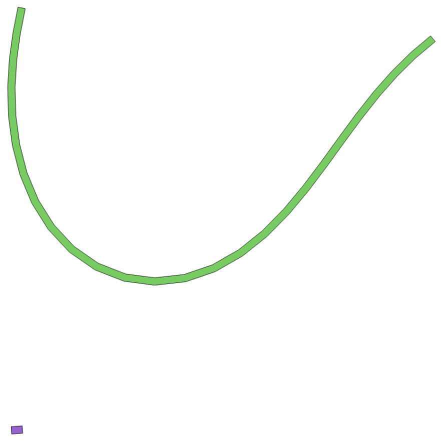
NCTC8300	*Streptococcus pyogenes*	212	1	0	0	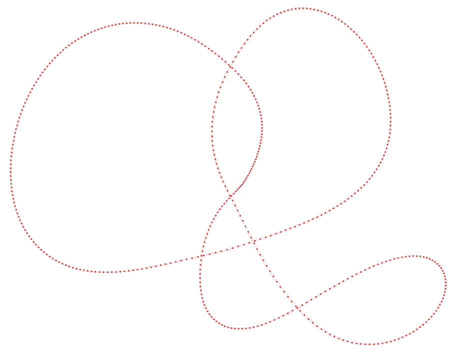	Finished circular assembly	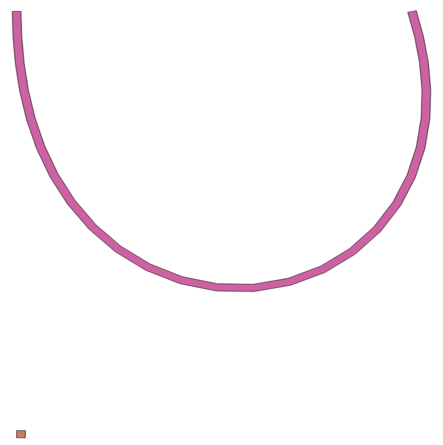
NCTC8303	*Streptococcus pyogenes*	82	Pending	Pending	Pending	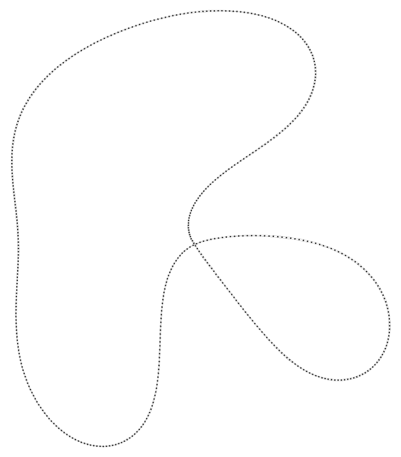	Finished circular assembly	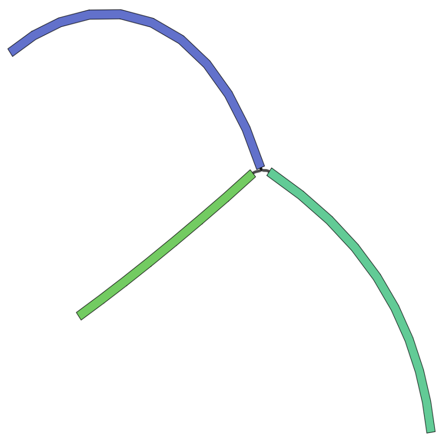
NCTC8314	*Streptococcus pyogenes*	212	1	0	0		Finished circular assembly	

						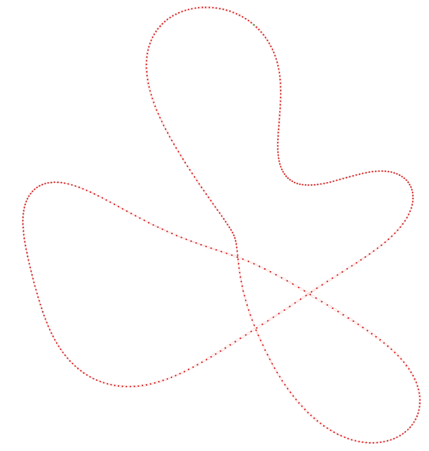		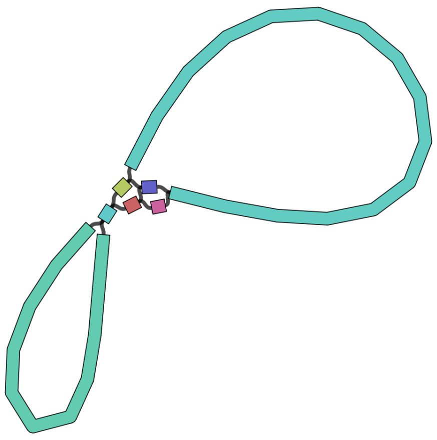
NCTC8317	*Staphylococcus aureus*	77	1	0	0	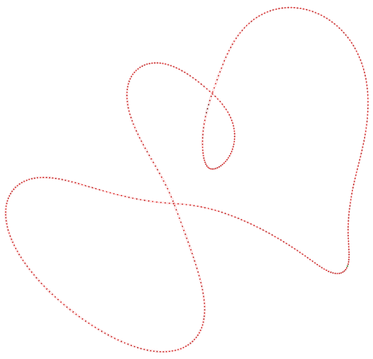	Finished circular assembly	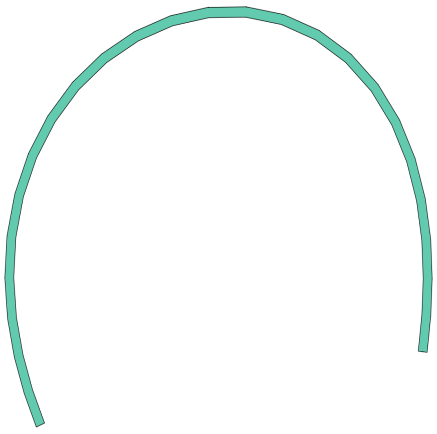
NCTC8324	*Streptococcus pyogenes*	142	Pending	Pending	Pending	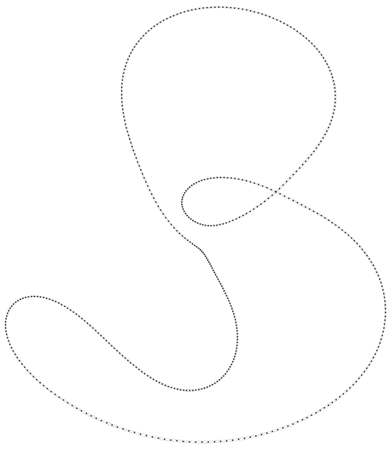	Finished circular assembly	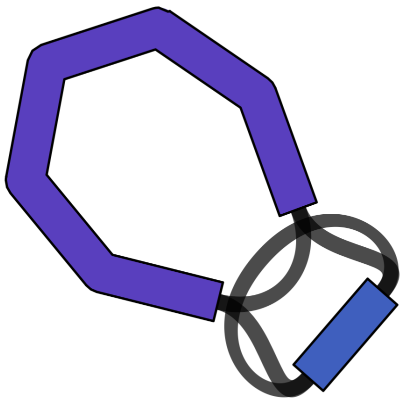
NCTC8326	*Streptococcus pyogenes*	168	Pending	Pending	Pending	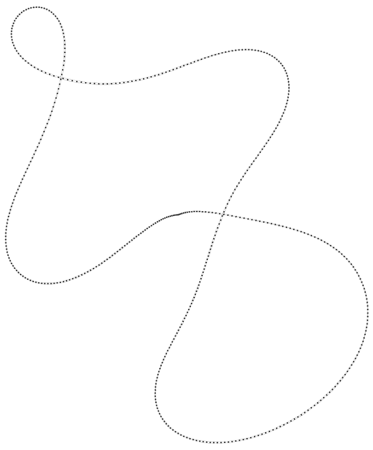	Finished circular assembly	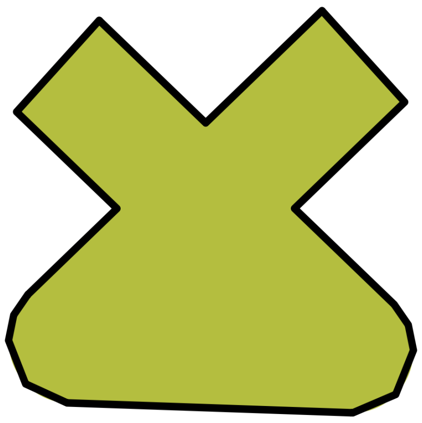
NCTC8328	*Streptococcus pyogenes*	208	1	0	0	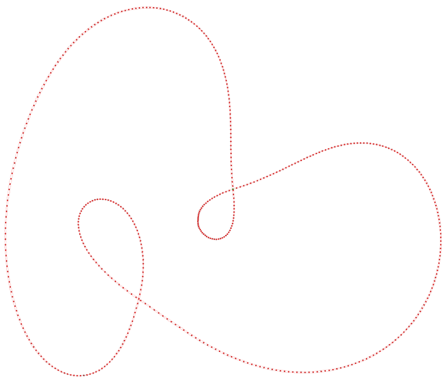	Finished circular assembly	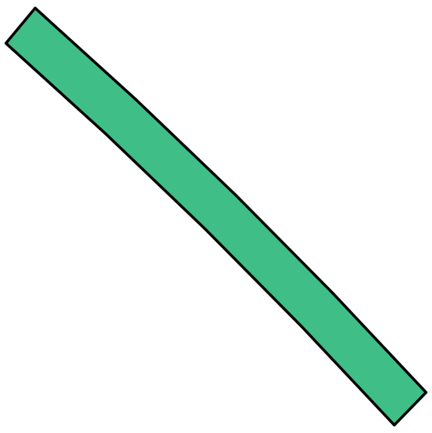
NCTC8332	*Streptococcus pyogenes*	203	1	0	0	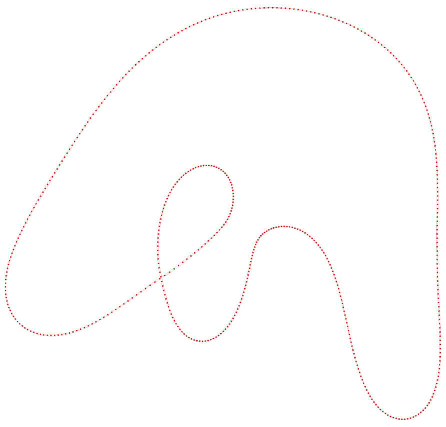	Finished circular assembly	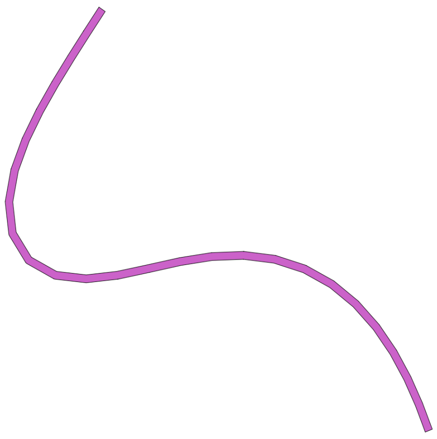
NCTC8333	*Escherichia coli*	50	1	1	5		Finished assembly with multiple traversals	

						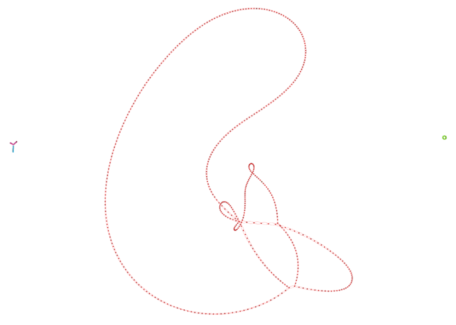		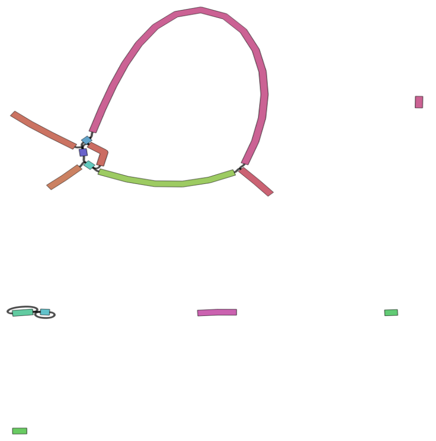
NCTC8399	*Staphylococcus aureus*	111	1	1	0	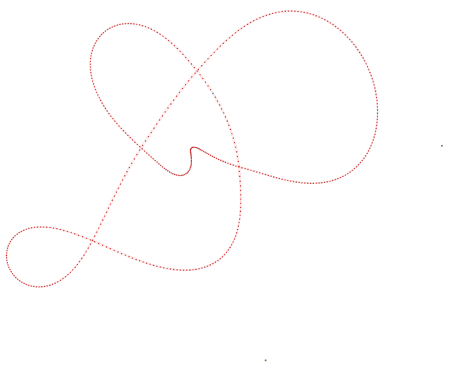	Finished circular assembly	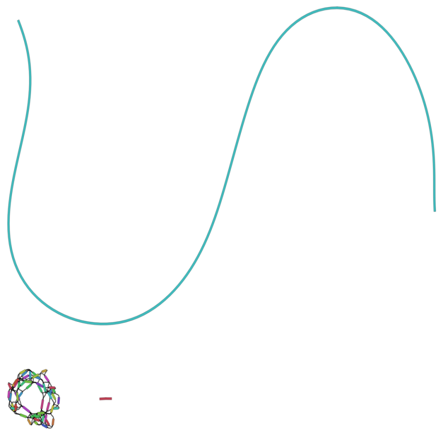
NCTC8450	*Escherichia coli*	45	0	0	3	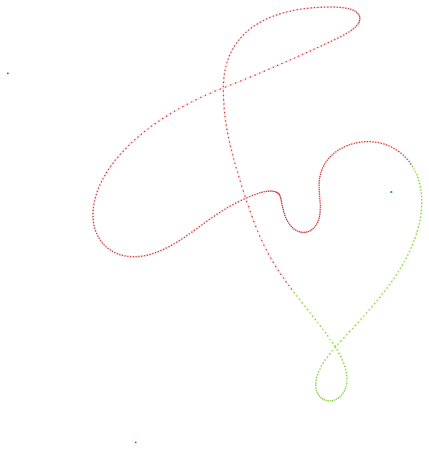	Finished circular assembly	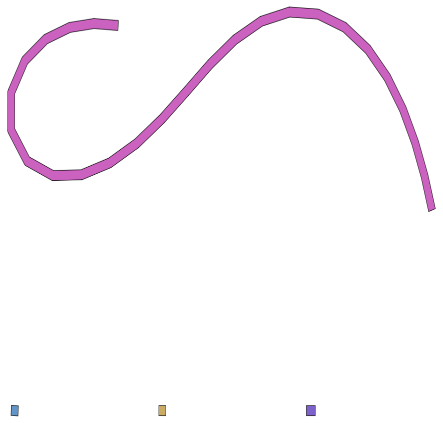
NCTC8455	*Haemophilus influenzae*	130	1	0	0	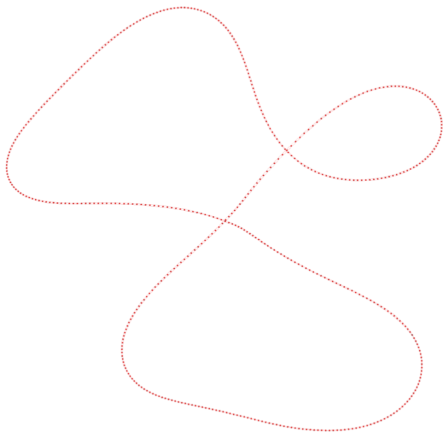	Finished circular assembly	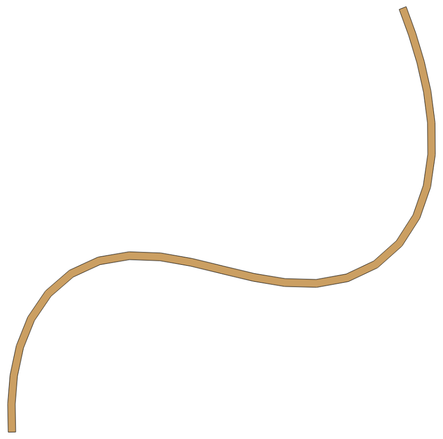
NCTC8468	*Haemophilus influenzae*	139	1	0	0	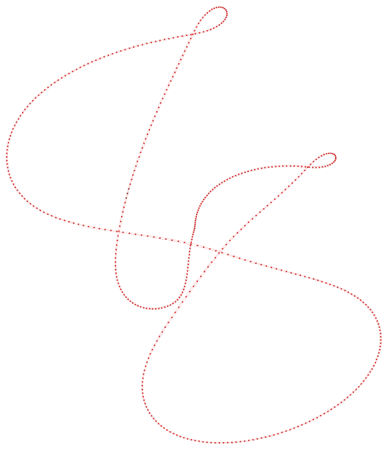	Finished circular assembly	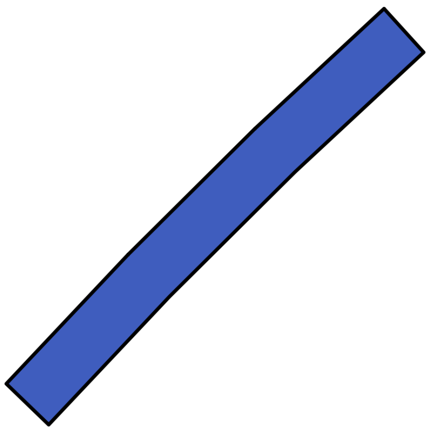
NCTC8480	*Yersinia pseudotuberculosis*	43	0	0	1	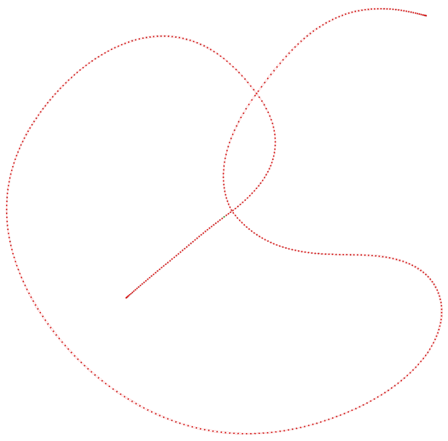	Finished assembly (lacking circularisation)	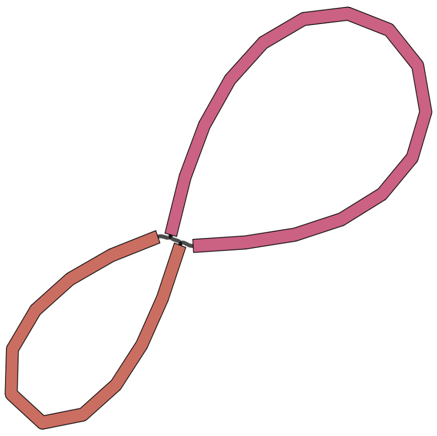
NCTC8489	*Pasteurella multocida*	109	1	0	1		Mis-assembly/Fragmented	

						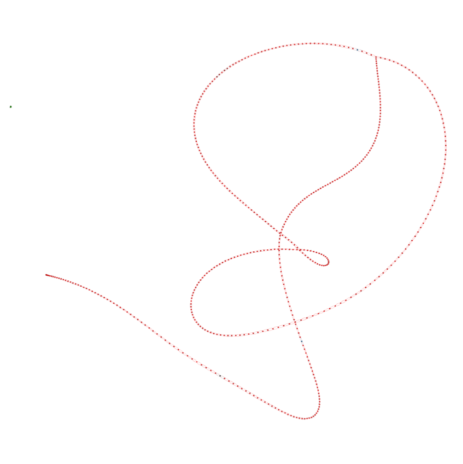		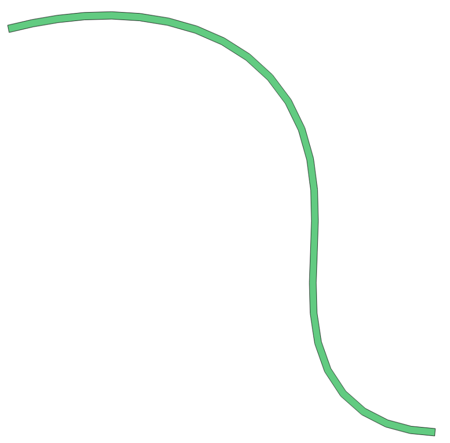
NCTC8496	*Salmonella enterica subsp. enterica*	59	1	0	0	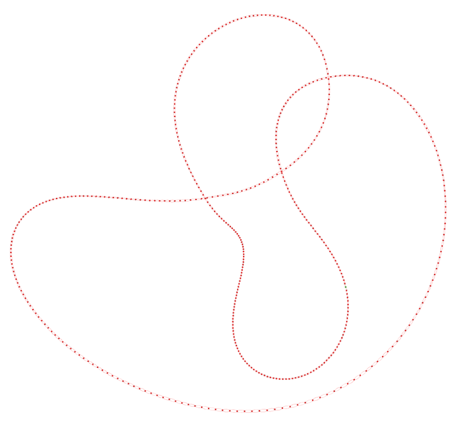	Finished circular assembly	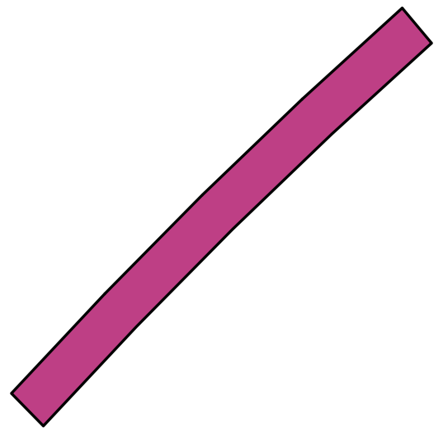
NCTC8500	*Escherichia coli*	29	1	1	0	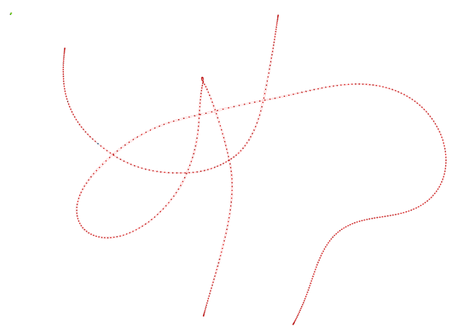	Mis-assembly/Fragmented	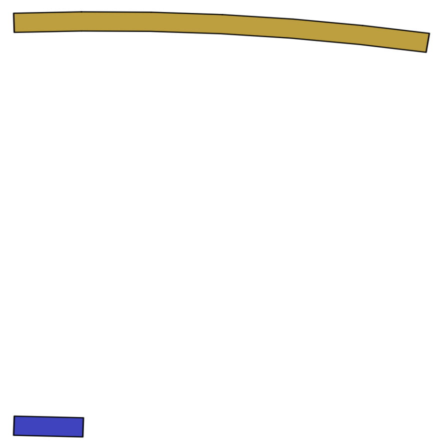
NCTC8507	*Staphylococcus aureus*	64	1	0	1	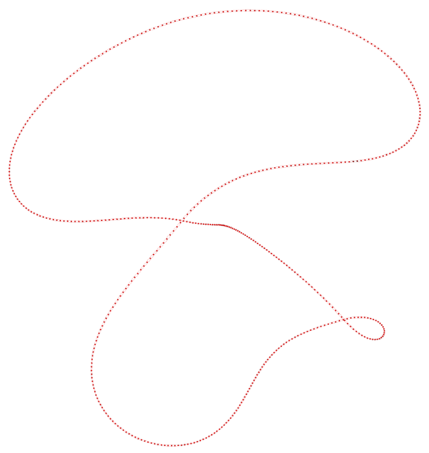	Finished circular assembly	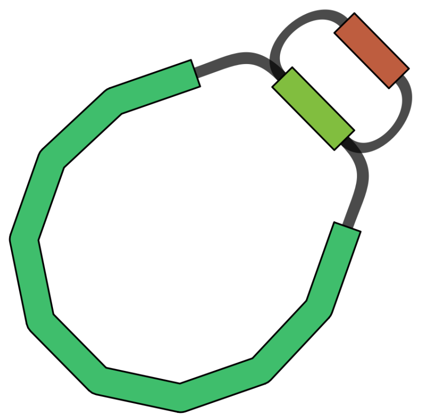
NCTC8530	*Staphylococcus aureus*	122	1	1	0	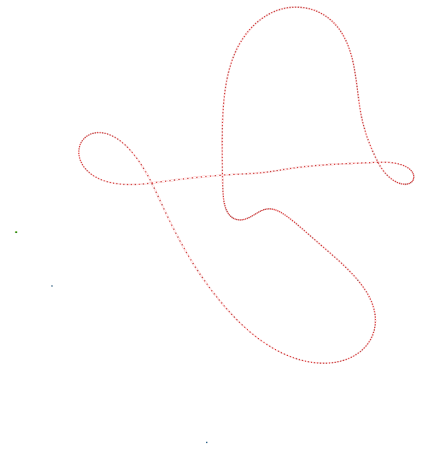	Finished circular assembly	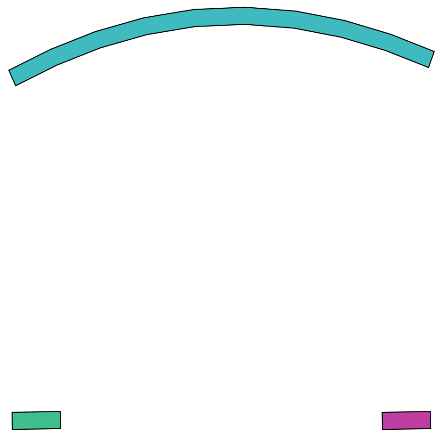
NCTC8532	*Staphylococcus aureus*	25	2	1	0	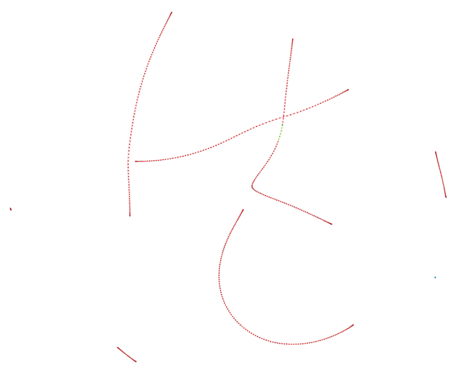	Mis-assembly/Fragmented	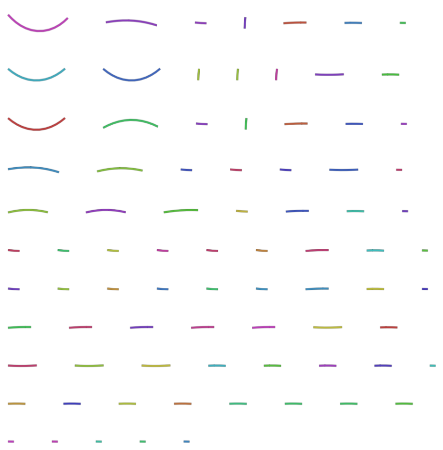
NCTC8546	*Streptococcus sp*	107	0	0	1		Finished circular assembly	

						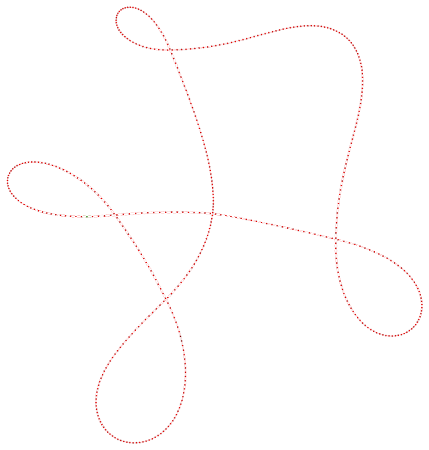		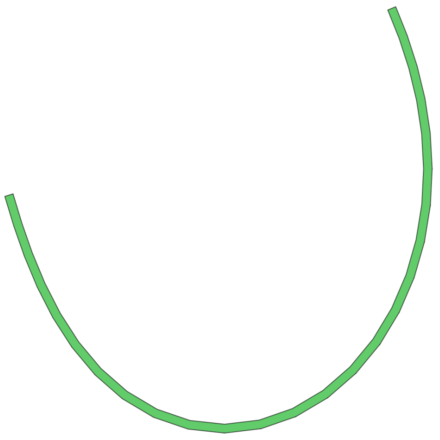
NCTC8547	*Streptococcus sp.*	112	Pending	Pending	Pending	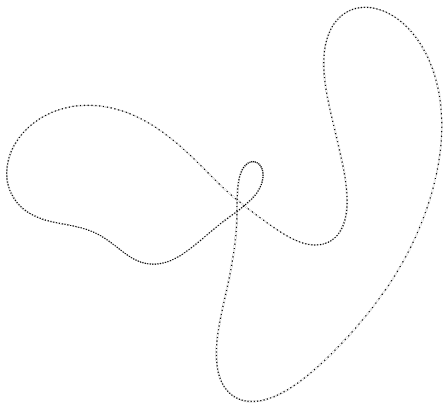	Finished circular assembly	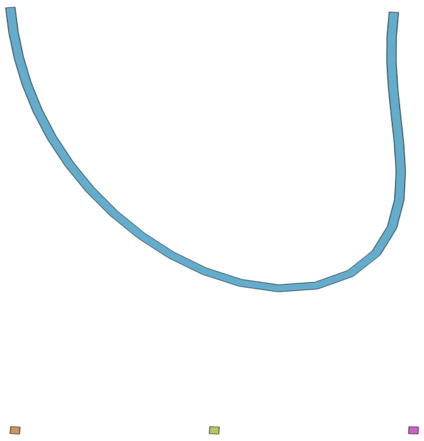
NCTC8603	*Escherichia coli*	77	0	0	3	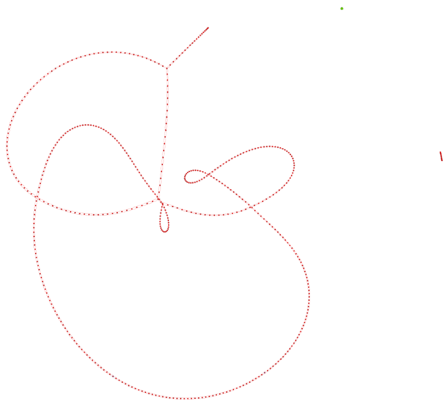	Mis-assembly/Fragmented	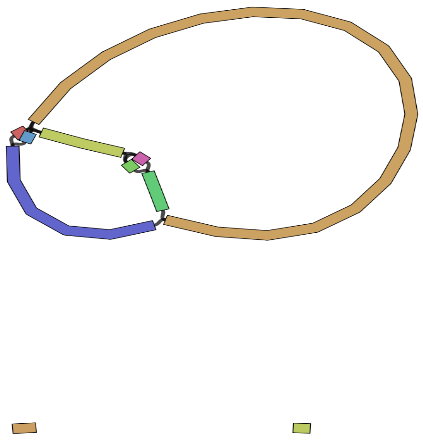
NCTC8618	*Streptococcus salivarius*	101	Pending	Pending	Pending	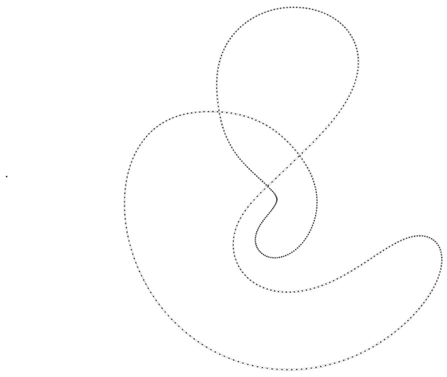	Finished circular assembly	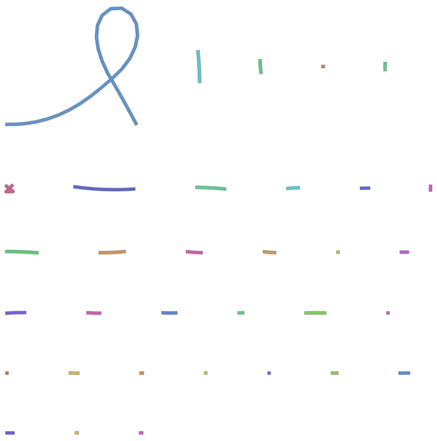
NCTC8620	*Escherichia coli*	76	0	0	5	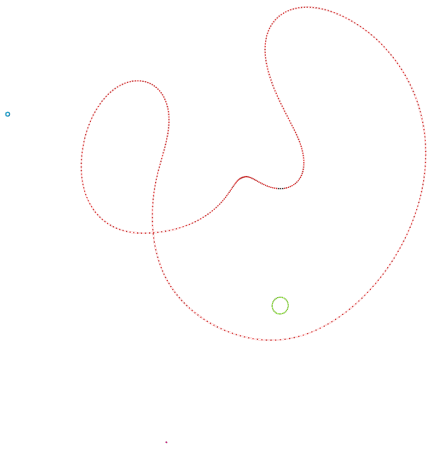	Finished circular assembly	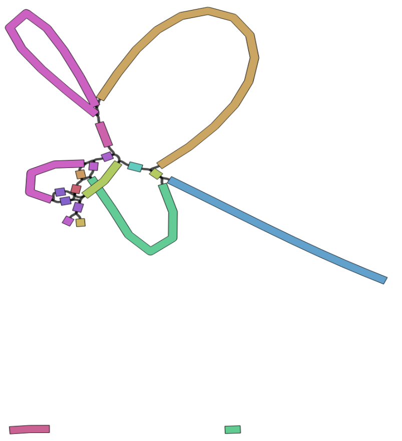
NCTC8621	*Escherichia coli*	95	0	0	3	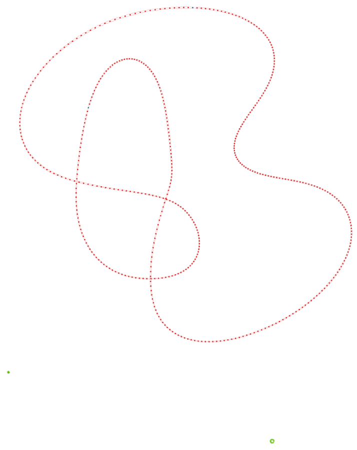	Finished circular assembly	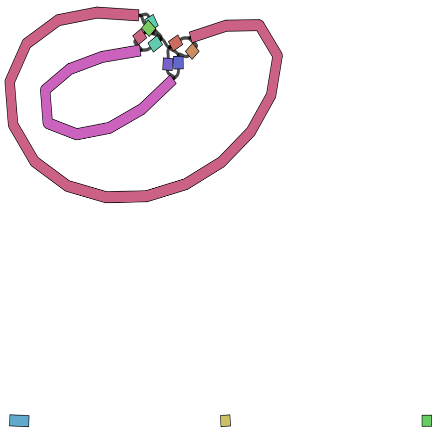
NCTC8622	*Escherichia coli*	25	0	0	8		Mis-assembly/Fragmented	

						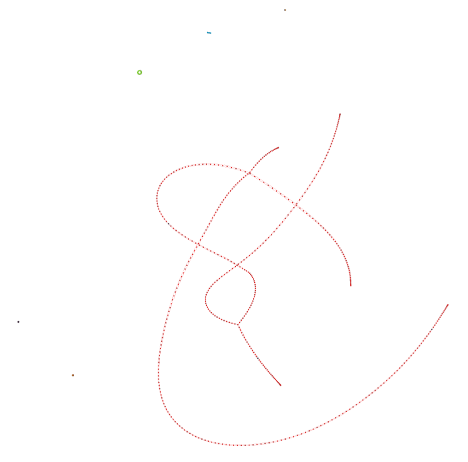		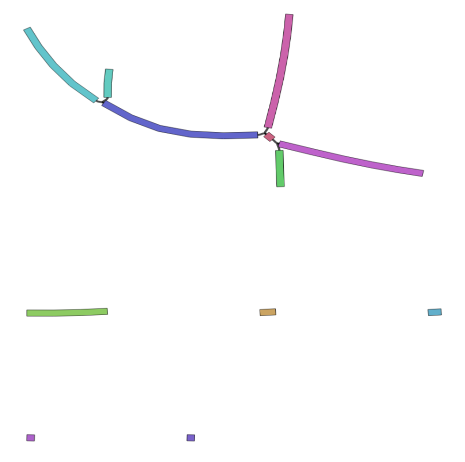
NCTC8623	*Escherichia coli*	87	0	0	1	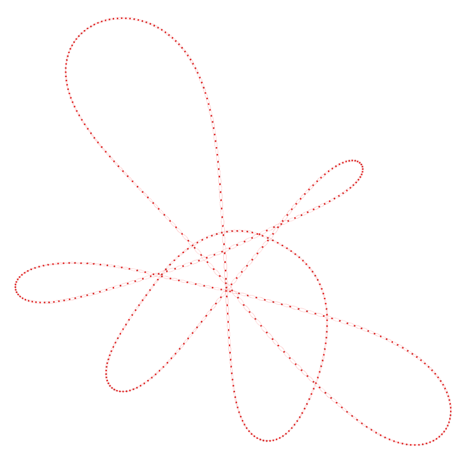	Finished circular assembly	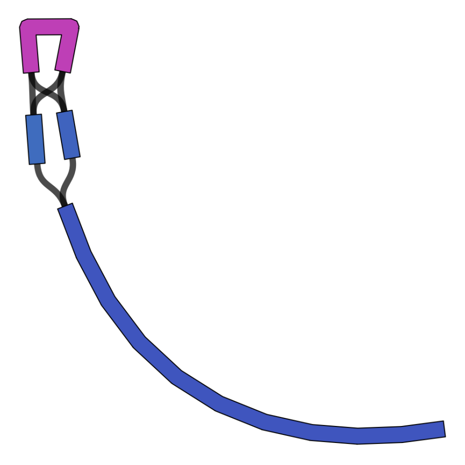
NCTC8684	*Chromobacterium violaceum*	36	0	0	3	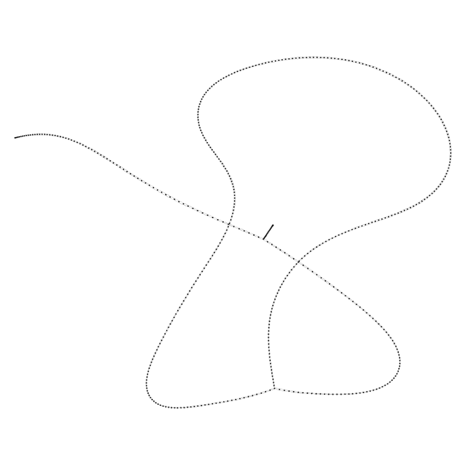	Mis-assembly/Fragmented	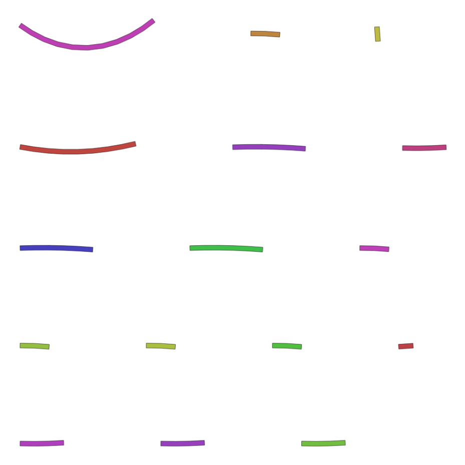
NCTC8705	*Salmonella enterica subsp. enterica*	48	1	0	1	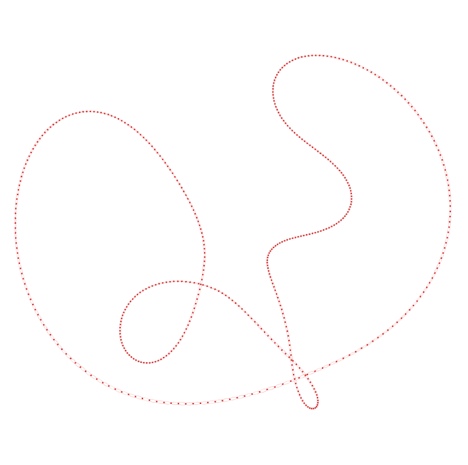	Finished circular assembly	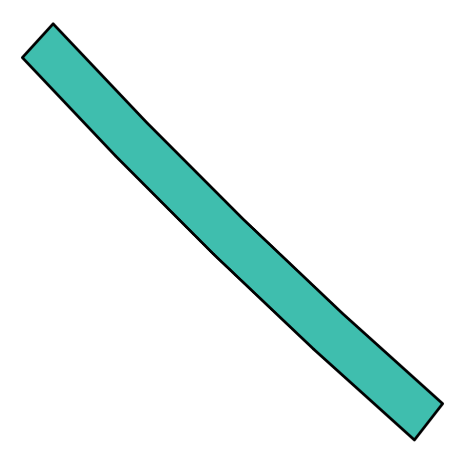
NCTC8721	*Bacillus licheniformis*	61	1	0	0	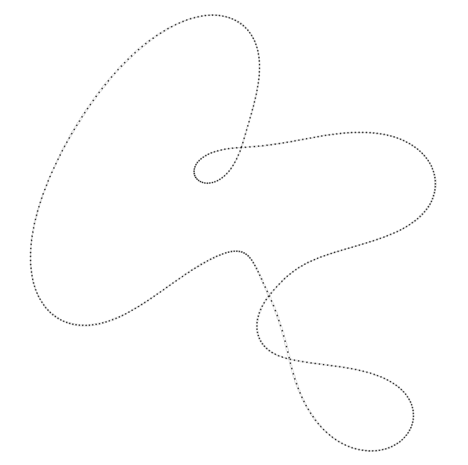	Finished circular assembly	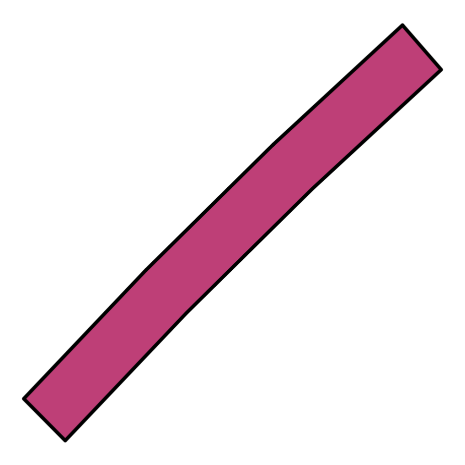
NCTC8723	*Staphylococcus aureus*	76	Pending	Pending	Pending	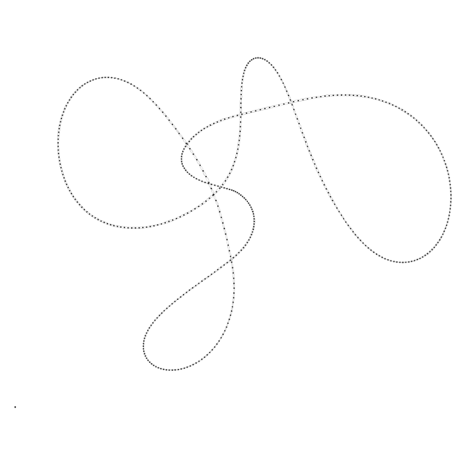	Finished circular assembly	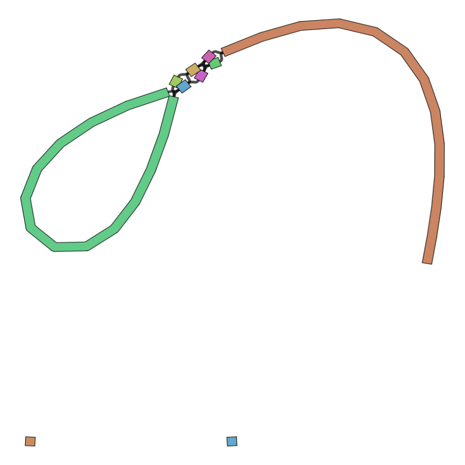
NCTC8724	*Staphylococcus aureus*	92	1	0	1		Finished circular assembly	

						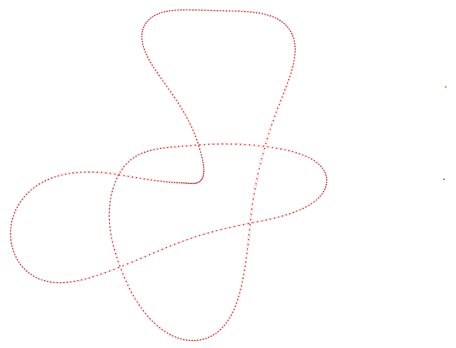		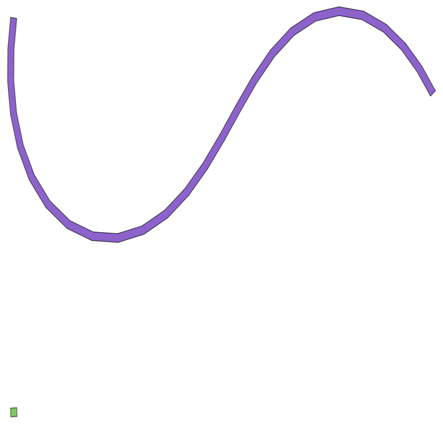
NCTC8725	*Staphylococcus aureus*	110	1	1	0	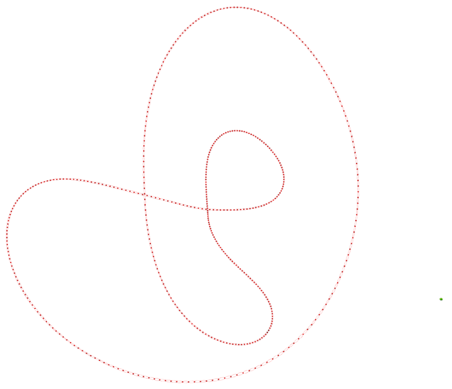	Finished circular assembly	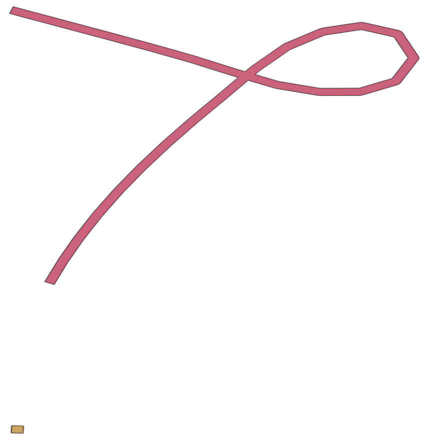
NCTC8726	*Staphylococcus aureus*	131	1	0	0	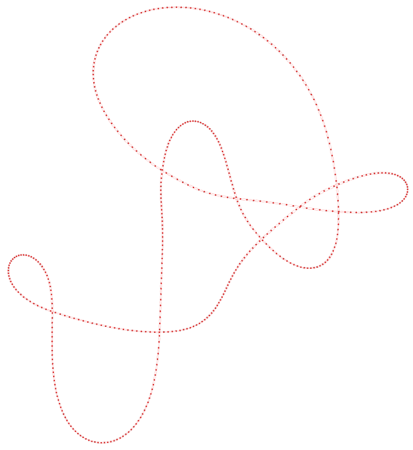	Finished circular assembly	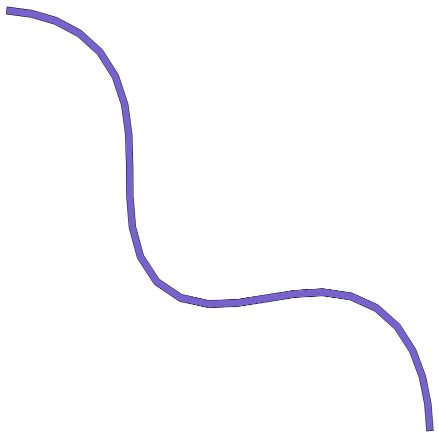
NCTC8727	*Streptococcus sp.*	76	Pending	Pending	Pending	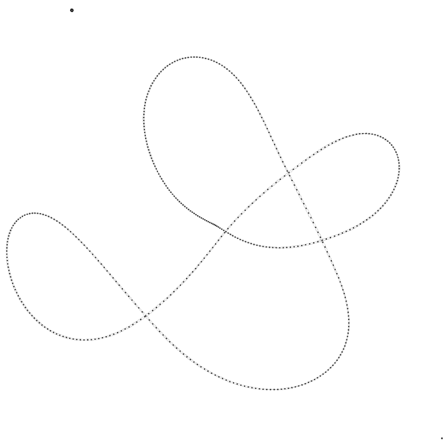	Finished circular assembly	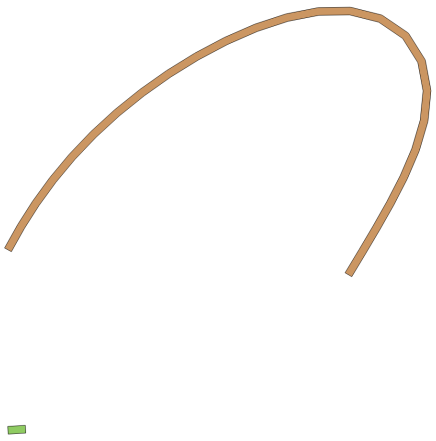
NCTC8729	*Streptococcus sp.*	81	Pending	Pending	Pending	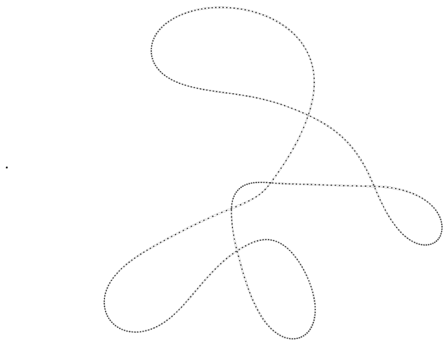	Finished circular assembly	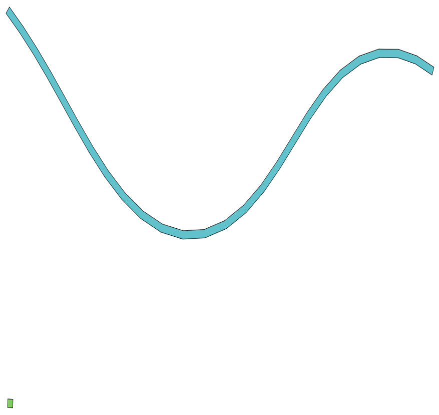
NCTC8731	*Streptococcus sp.*	51	Pending	Pending	Pending	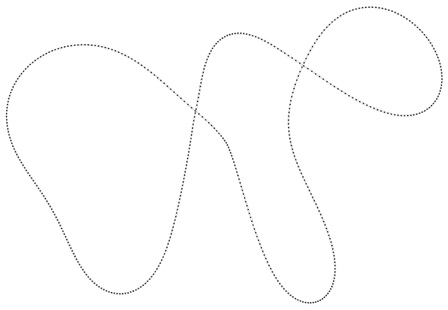	Finished circular assembly	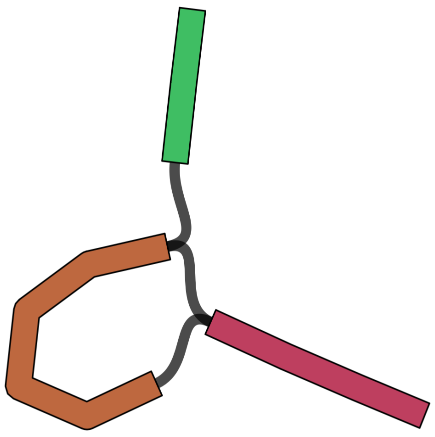
NCTC8732	*Streptococcus sp.*	76	Pending	Pending	Pending		Finished circular assembly	

						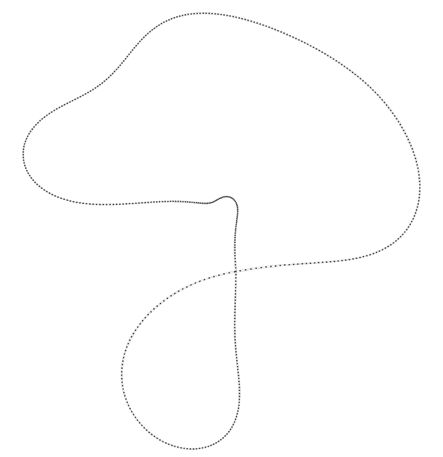		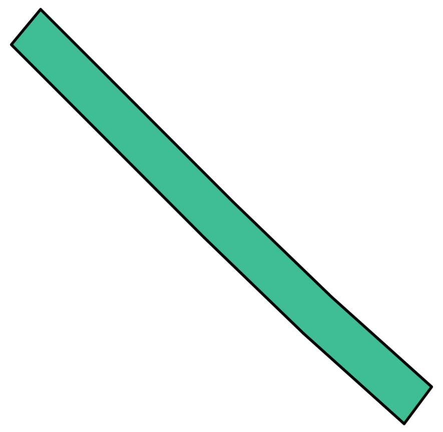
NCTC8734	*Streptococcus sp*.	75	Pending	Pending	Pending	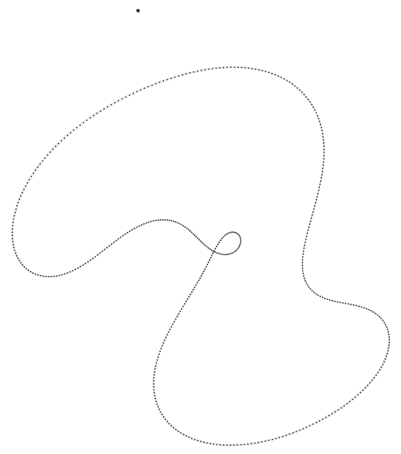	Finished circular assembly	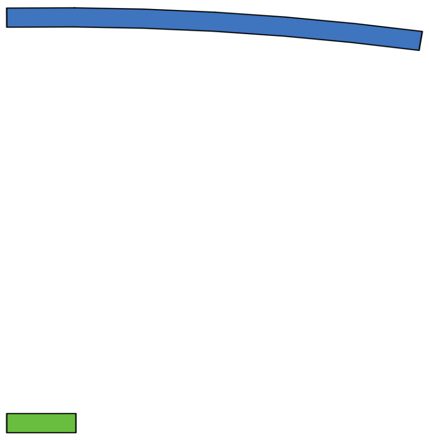
NCTC8736	*Streptococcus sp*.	79	Pending	Pending	Pending	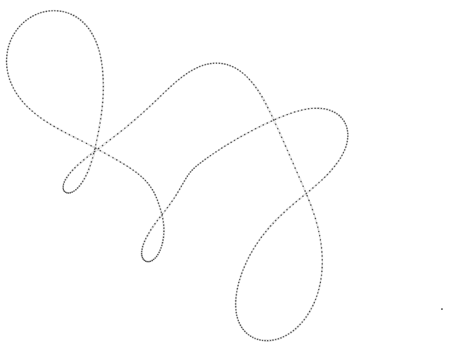	Finished circular assembly	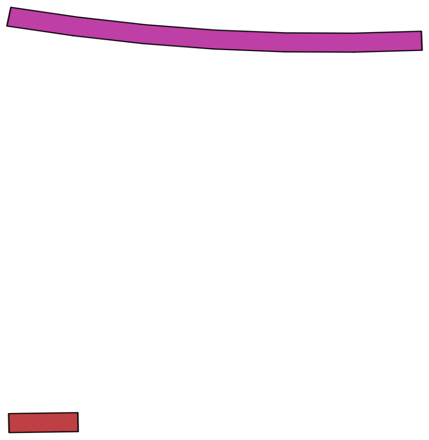
NCTC8738	*Streptococcus sp*.	125	Pending	Pending	Pending	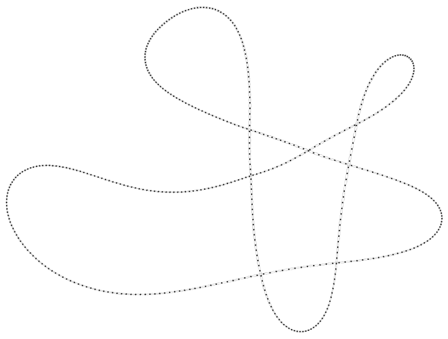	Finished circular assembly	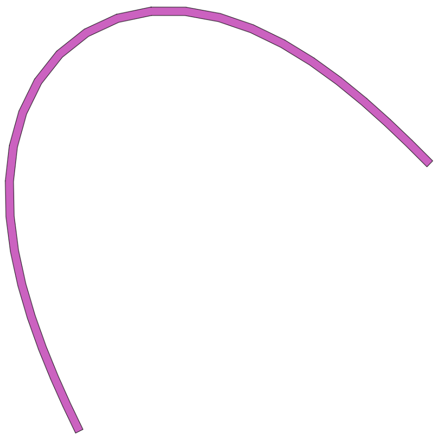
NCTC8740	*Streptococcus sp*.	117	Pending	Pending	Pending	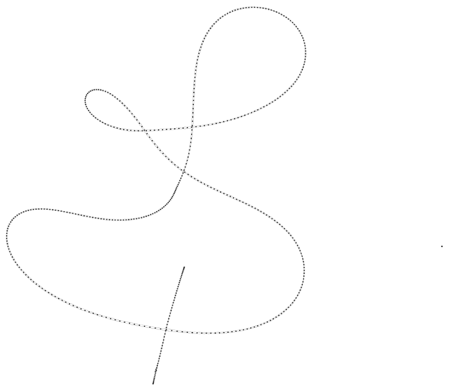	Mis-assembly/Fragmented	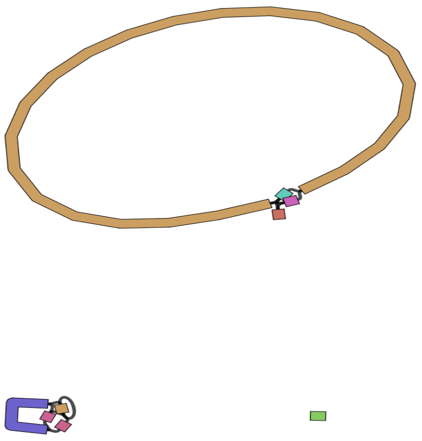
NCTC8741	*Streptococcus sp*.	52	Pending	Pending	Pending	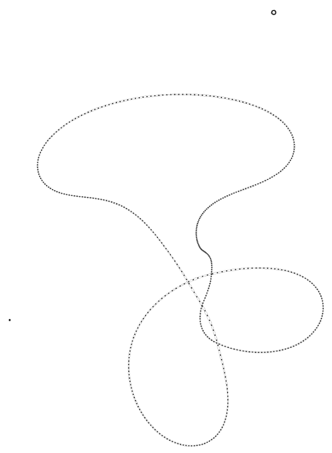	Finished circular assembly	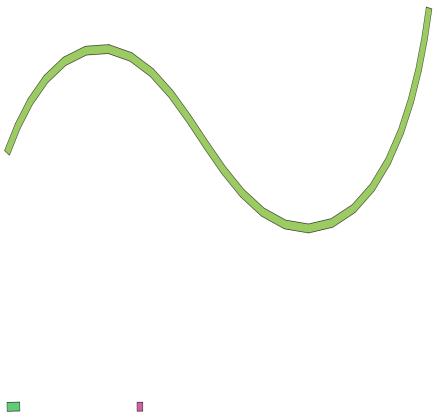
NCTC8742	*Streptococcus sp*.	49	Pending	Pending	Pending		Mis-assembly/Fragmented	

						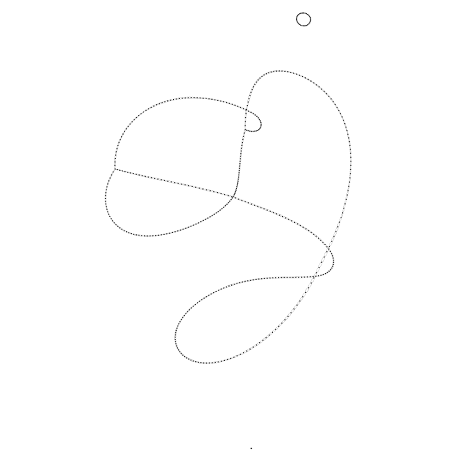		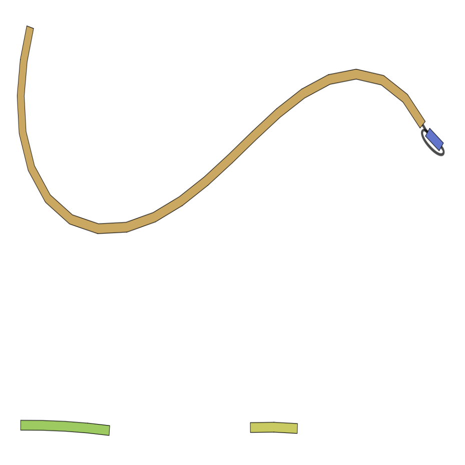
NCTC8743	*Streptococcus sp.*	118	Pending	Pending	Pending	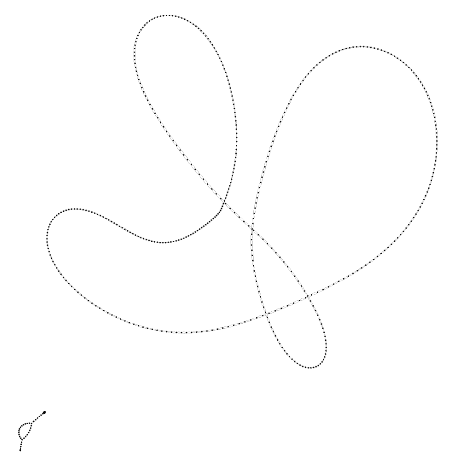	Finished circular assembly	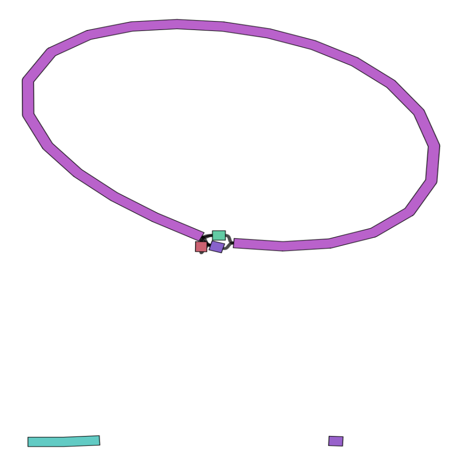
NCTC8746	*Streptococcus sp.*	126	Pending	Pending	Pending	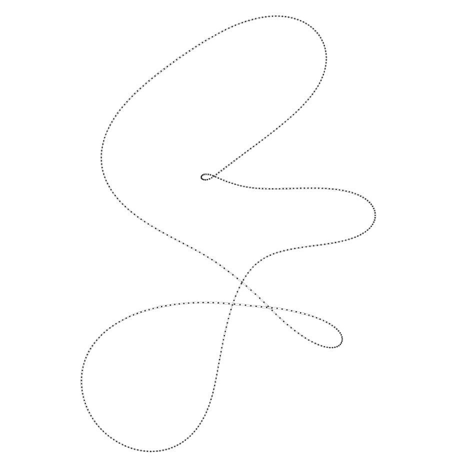	Finished circular assembly	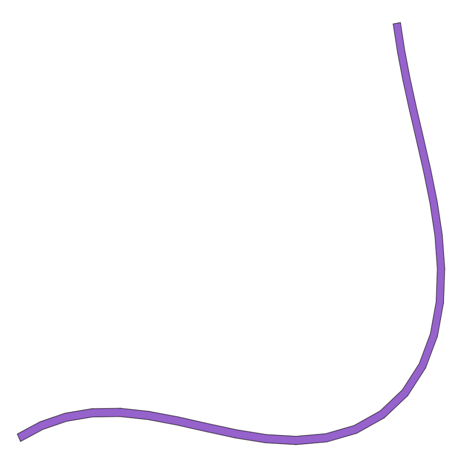
NCTC8749	*Streptococcus*	112	Pending	Pending	Pending	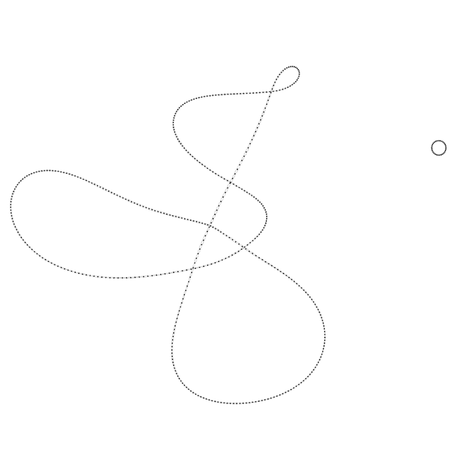	Finished circular assembly	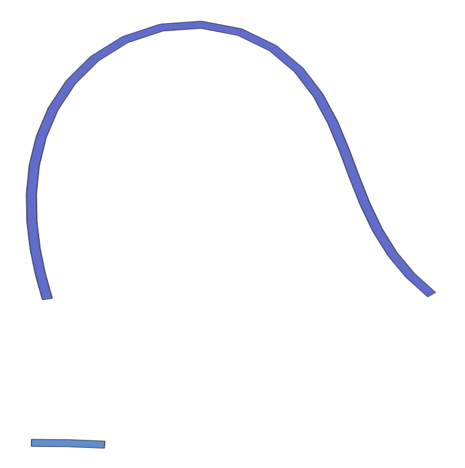
NCTC8765	*Staphylococcus aureus*	128	1	0	1	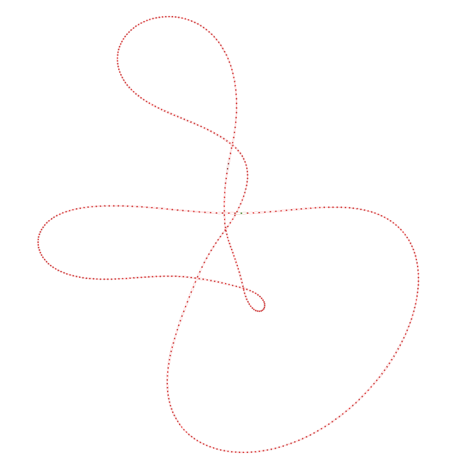	Finished circular assembly	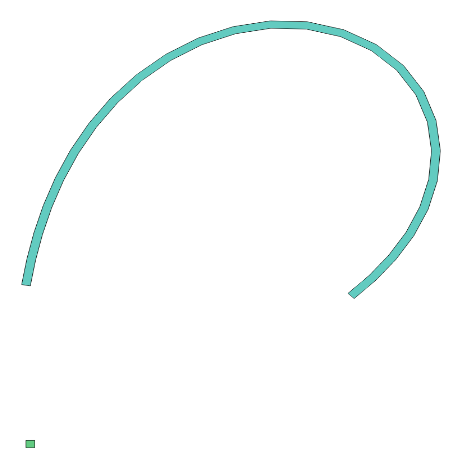
NCTC8766	*Staphylococcus aureus*	134	1	1	0	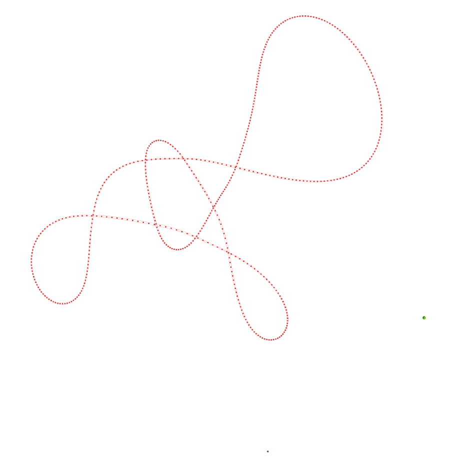	Finished circular assembly	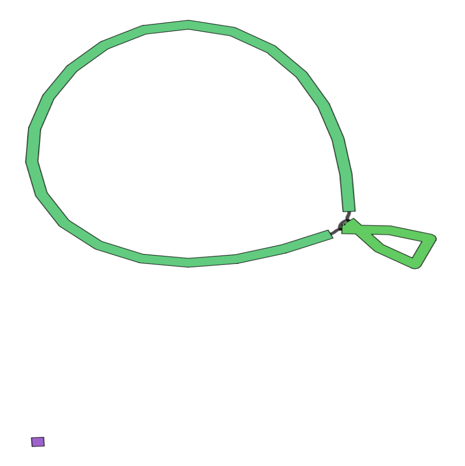
NCTC8781	*Escherichia coli*	37	0	0	3		Finished assembly with multiple traversals	

						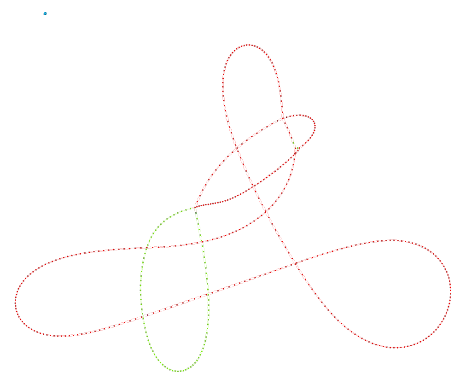		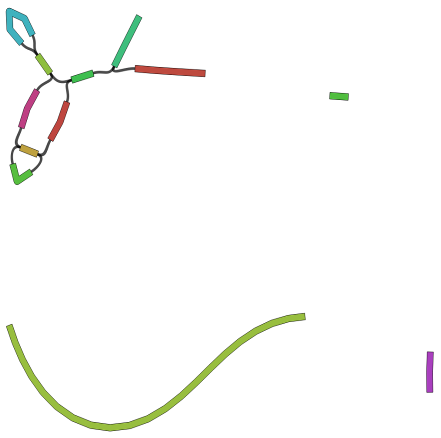
NCTC8783	*Escherichia coli*	65	0	0	4	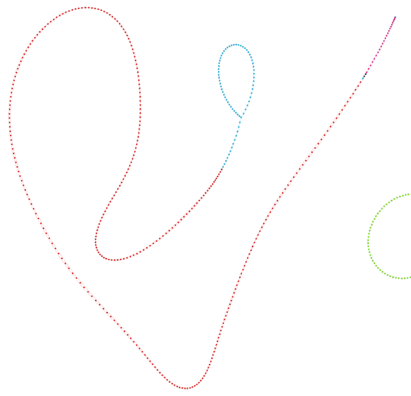	Mis-assembly/Fragmented	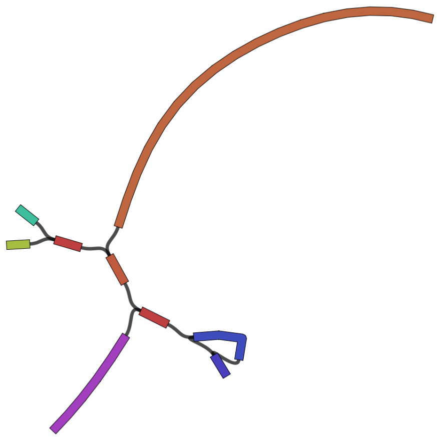
NCTC8796	*Streptococcus sp.*	122	Pending	Pending	Pending	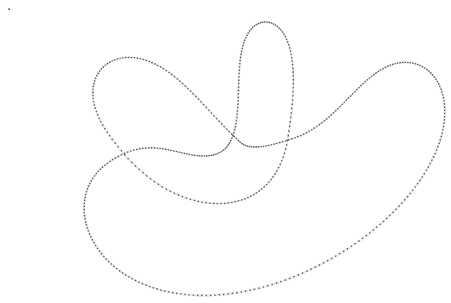	Finished circular assembly	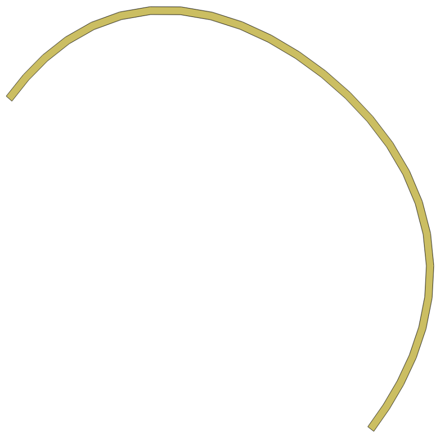
NCTC8839	*Klebsiella sp.*	65	1	1	1	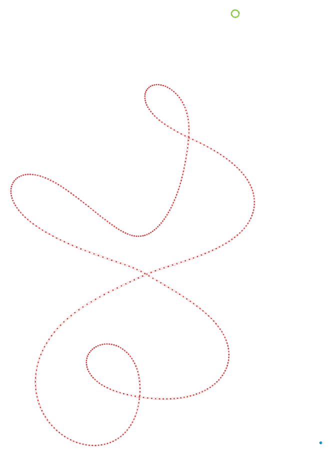	Finished circular assembly	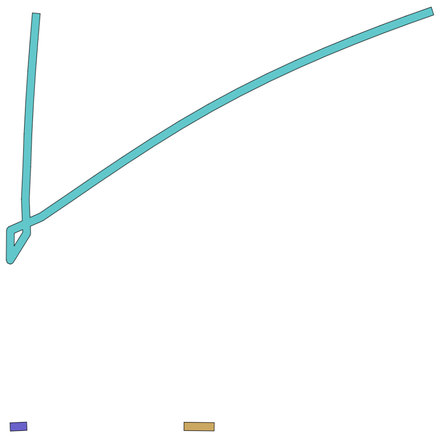
NCTC8846	*Enterobacter aerogenes*	29	Pending	Pending	Pending	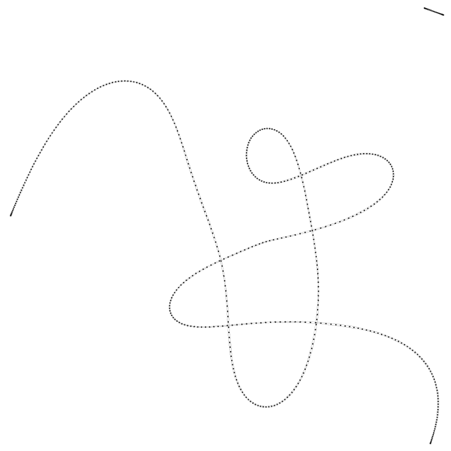	Finished assembly (lacking circularisation)	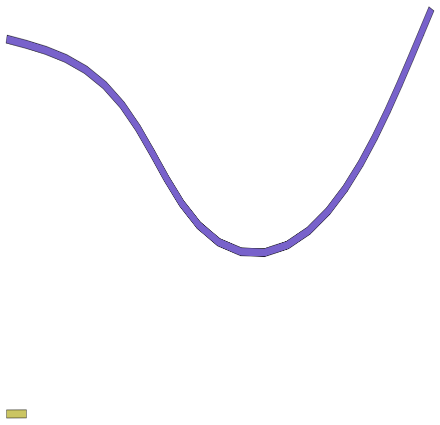
NCTC8849	*Klebsiella pneumoniae*	27	0	0	5	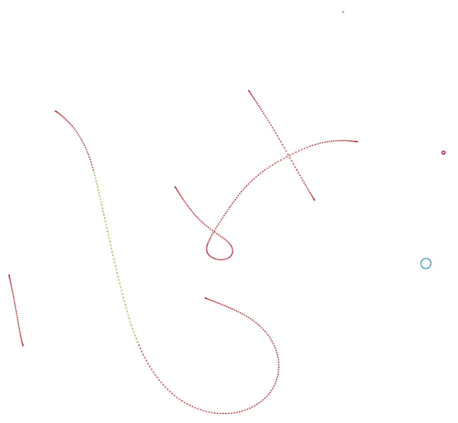	Mis-assembly/Fragmented	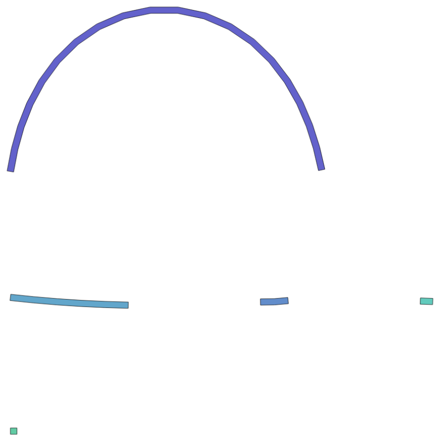
NCTC8883	*Klebsiella pneumoniae*	46	0	0	7		Mis-assembly/Fragmented	

						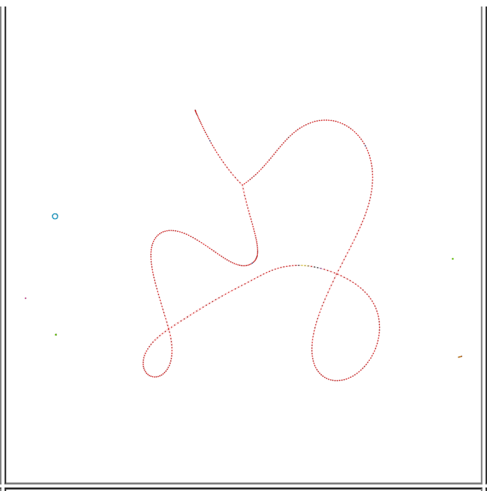		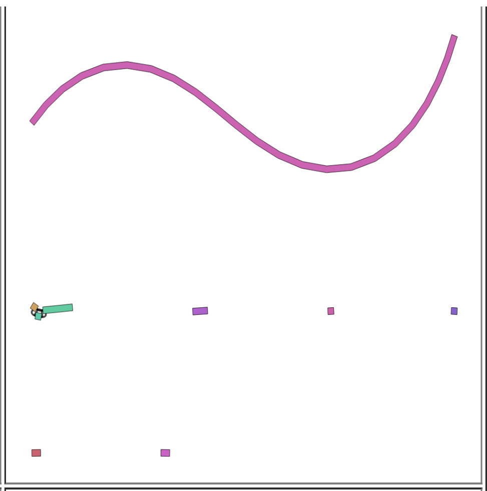
NCTC8895	*Klebsiella pneumoniae*	46	0	0	4	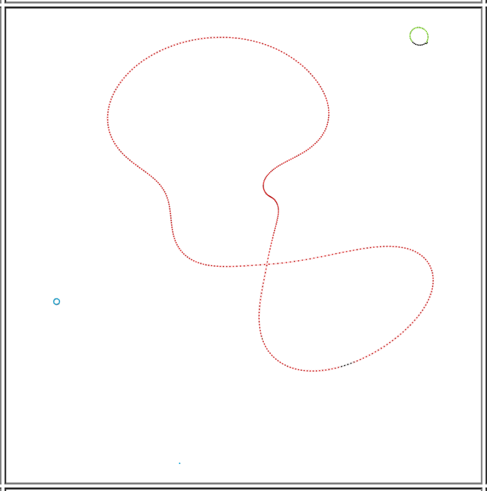	Finished circular assembly	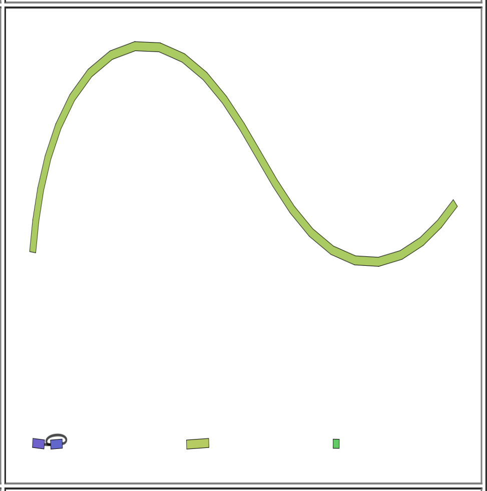
NCTC8900	*Serratia plymuthica*	55	1	0	0	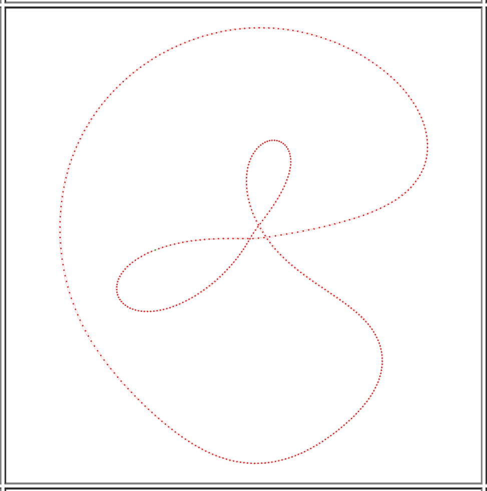	Finished circular assembly	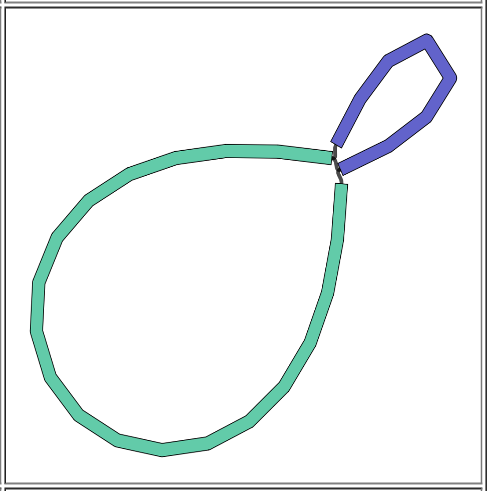
NCTC8960	*Escherichia coli*	61	0	0	7	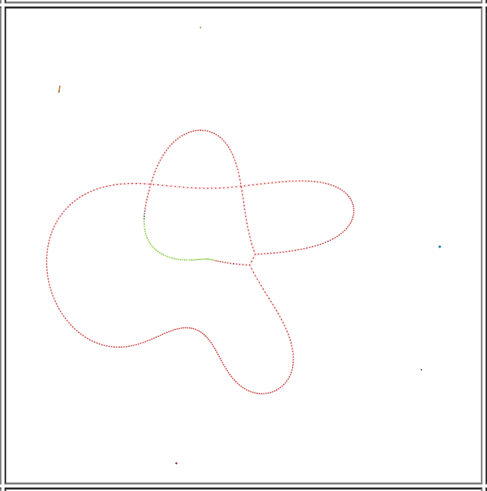	Finished assembly with multiple traversals	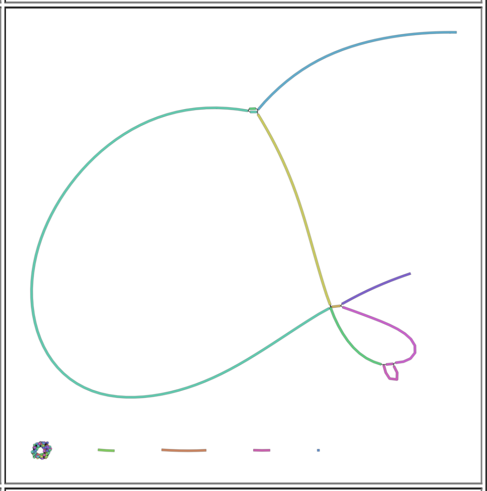
NCTC8984	*Escherichia coli*	41	1	1	2	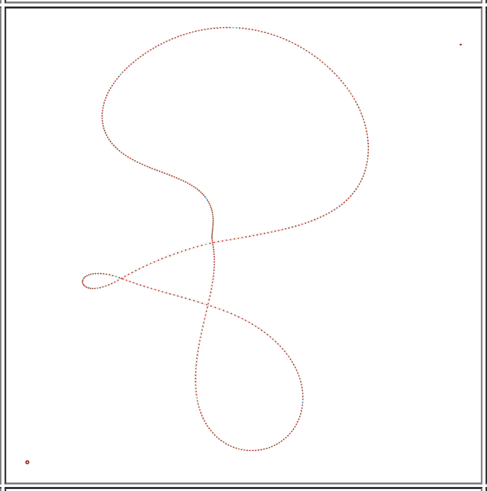	Finished circular assembly	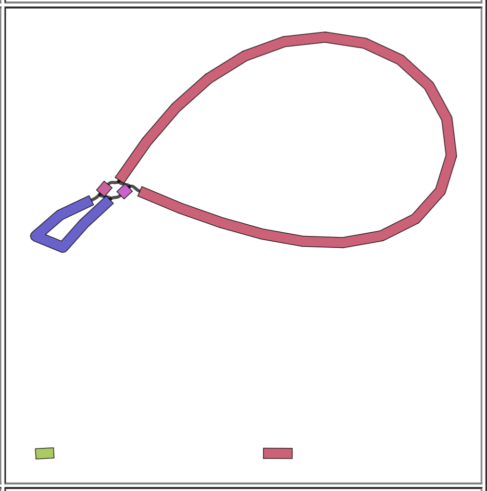
NCTC8985	*Escherichia coli*	24	0	0	3	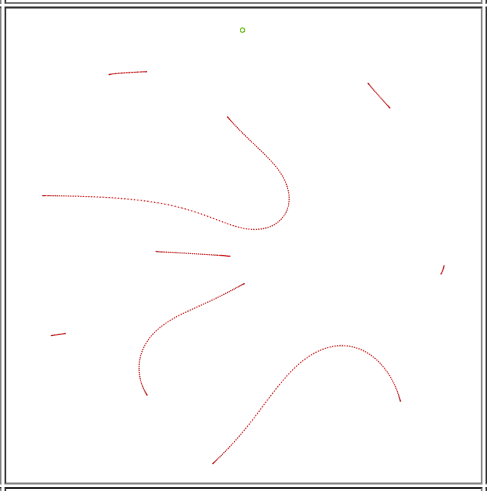	Mis-assembly/Fragmented	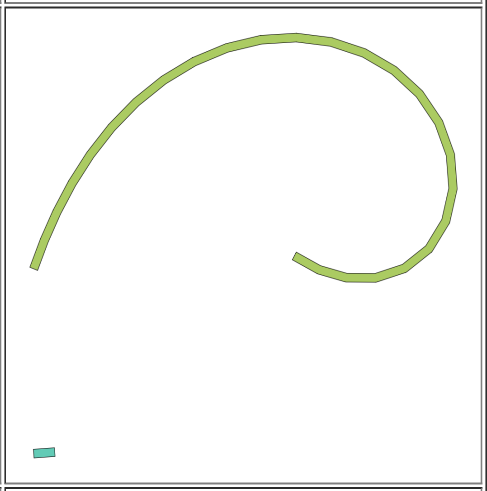
NCTC9001	*Escherichia coli*	40	6	2	0		Mis-assembly/Fragmented	

						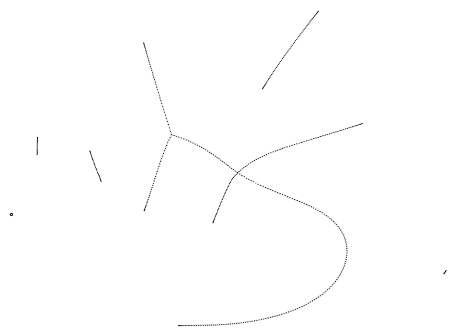		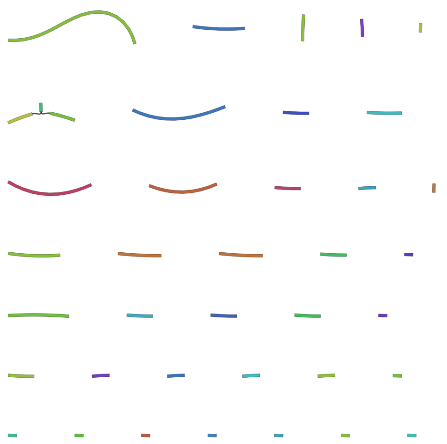
NCTC9002	*Escherichia coli*	87	0	0	11	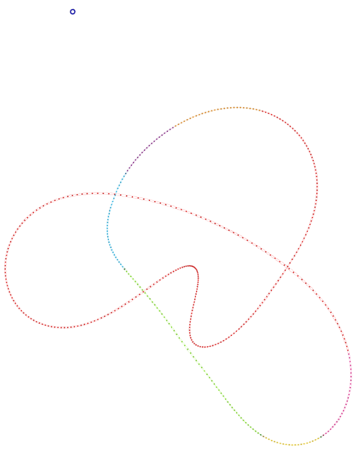	Finished circular assembly	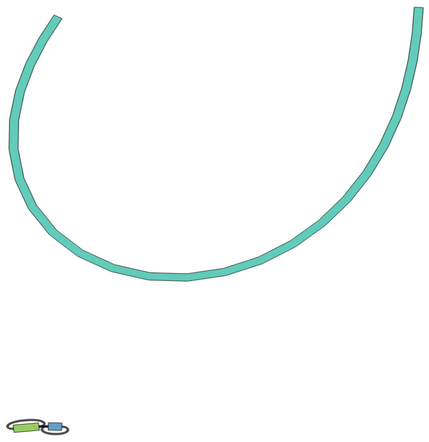
NCTC9003	*Escherichia coli*	76	1	1	0	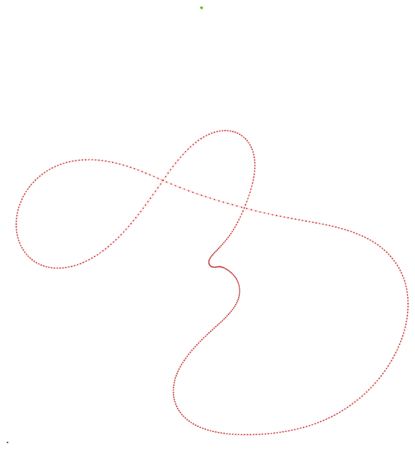	Finished circular assembly	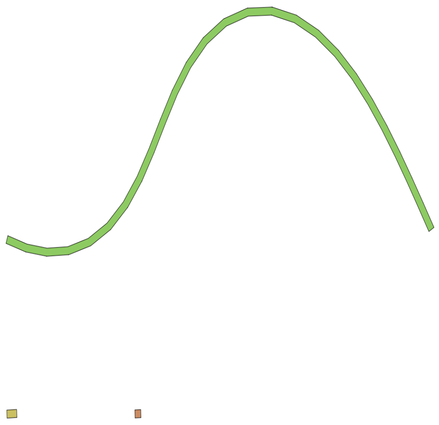
NCTC9004	*Escherichia coli*	42	1	2	0	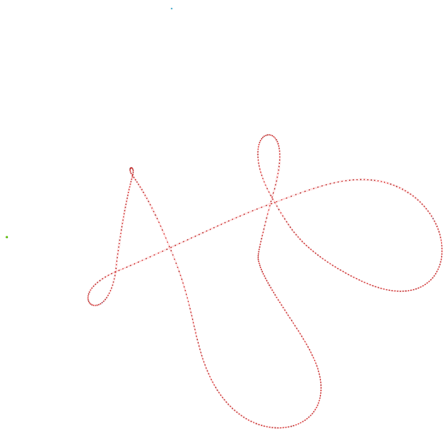	Finished circular assembly	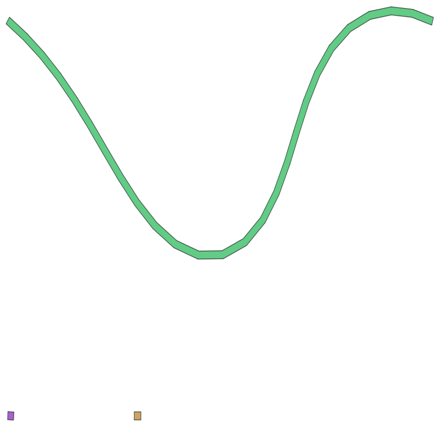
NCTC9005	*Escherichia coli*	85	1	0	1	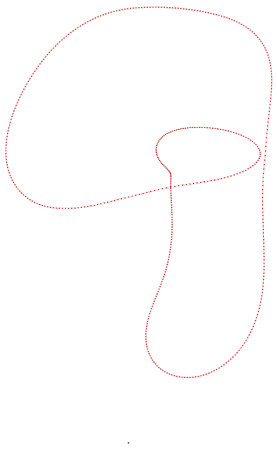	Finished circular assembly	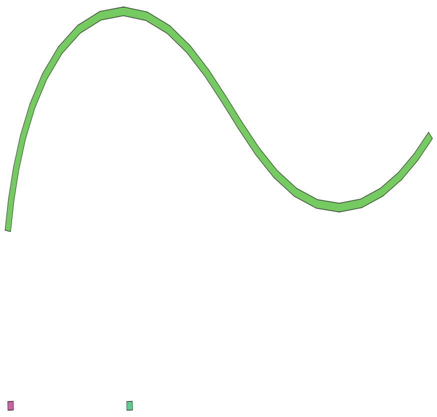
NCTC9006	*Escherichia coli*	51	0	0	6	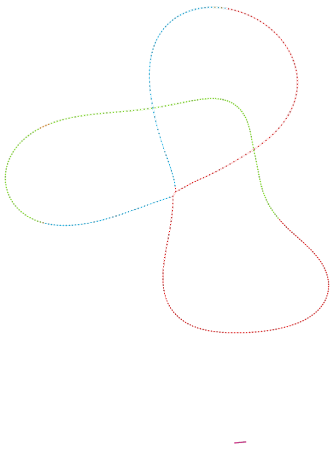	Finished assembly with multiple traversals	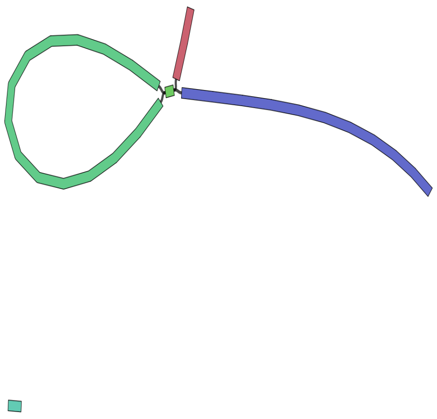
NCTC9007	*Escherichia coli*	77	0	0	4		Finished assembly with multiple traversals	

						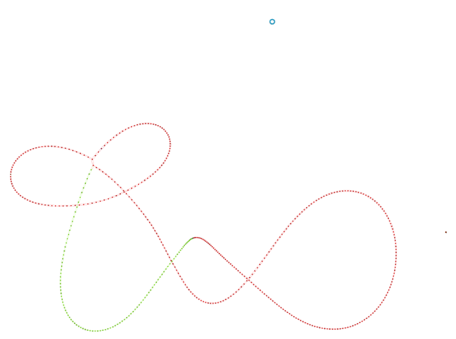		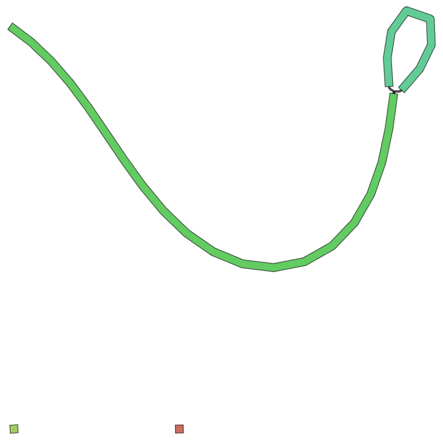
NCTC9008	*Escherichia coli*	100	1	0	0	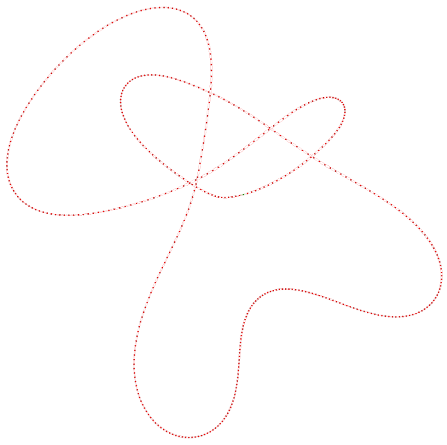	Finished circular assembly	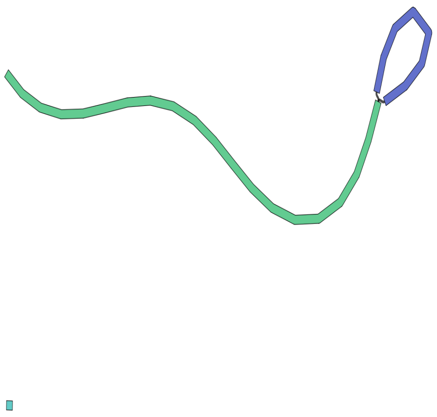
NCTC9009	*Escherichia coli*	77	1	0	1	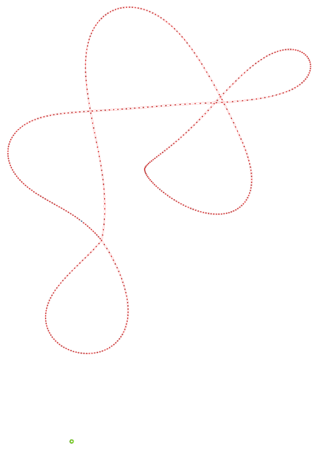	Finished assembly with multiple traversals	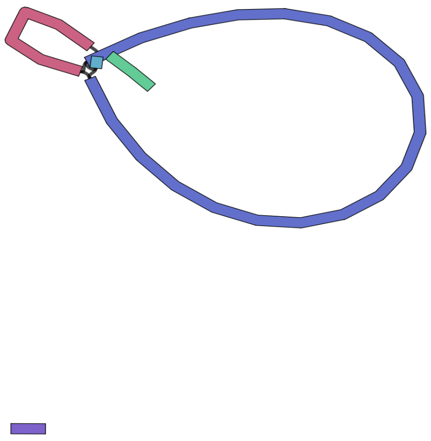
NCTC9010	*Escherichia coli*	90	0	0	5	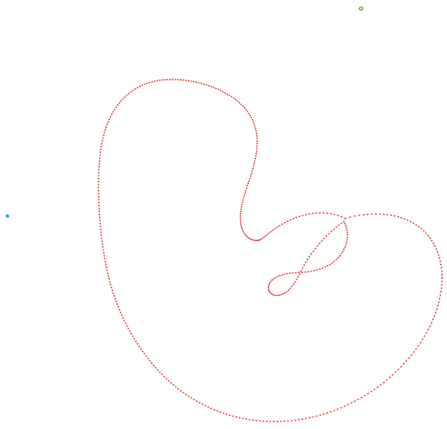	Finished assembly with multiple traversals	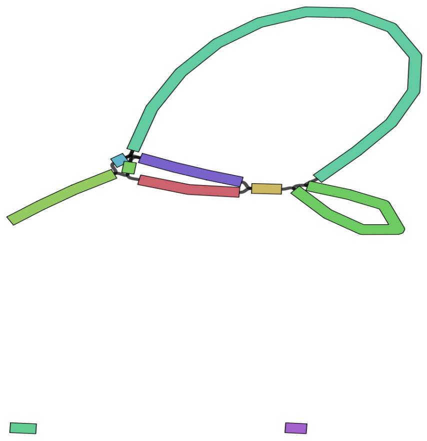
NCTC9011	*Escherichia coli*	72	0	0	5	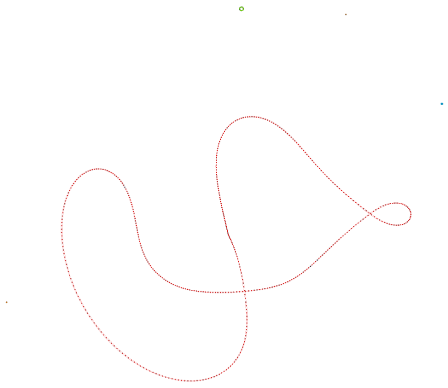	Finished circular assembly	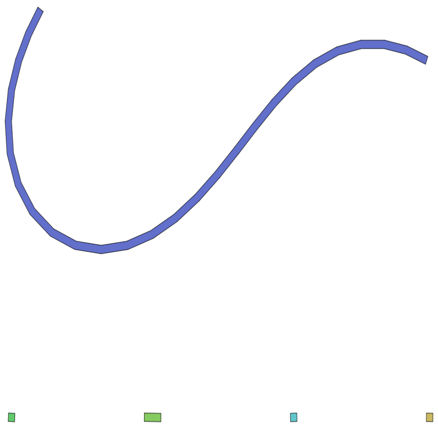
NCTC9012	*Escherichia coli*	63	1	0	3	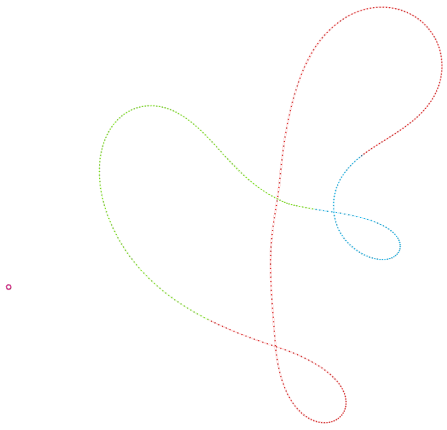	Finished circular assembly	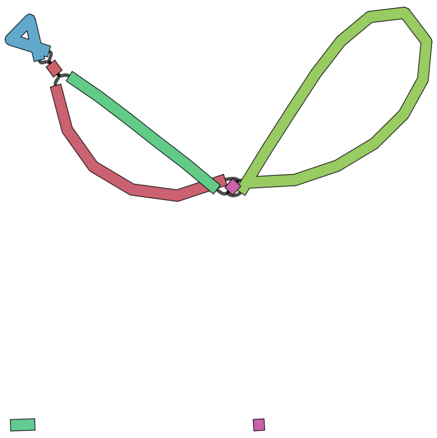
NCTC9014	*Escherichia coli*	47	1	0	1		Finished circular assembly	

						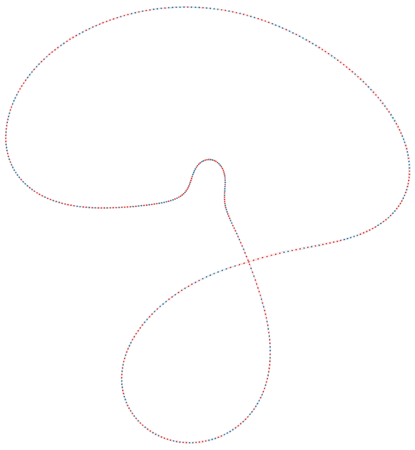		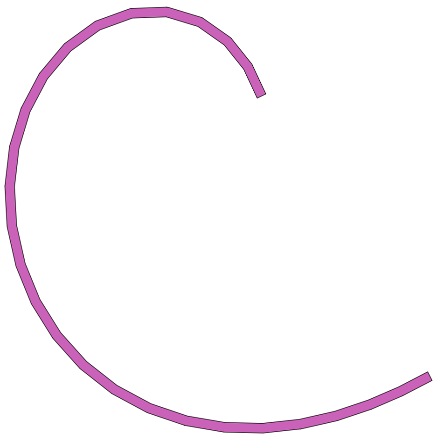
NCTC9015	*Escherichia coli*	73	1	2	1	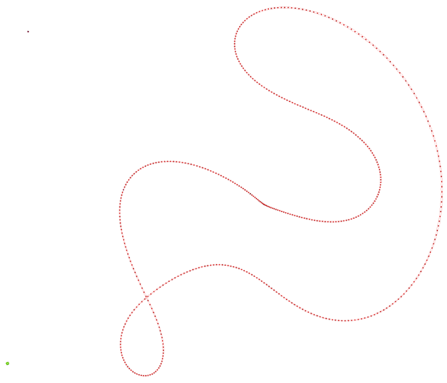	Finished circular assembly	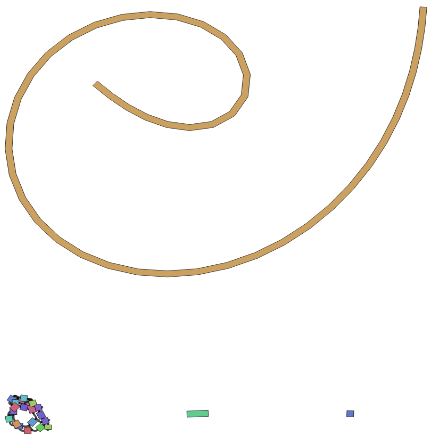
NCTC9016	*Escherichia coli*	46	0	0	118	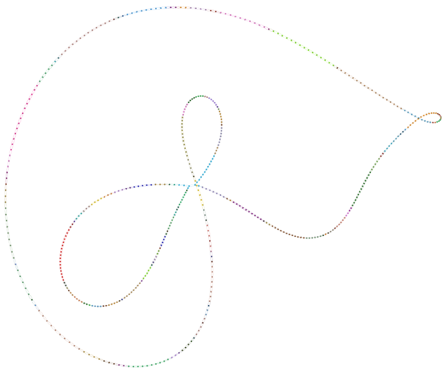	Finished assembly with multiple traversals	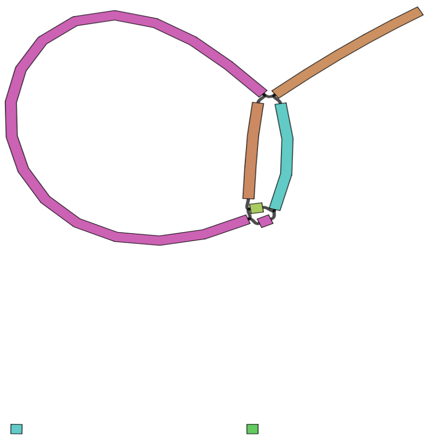
NCTC9019	*Escherichia coli*	78	Pending	Pending	Pending	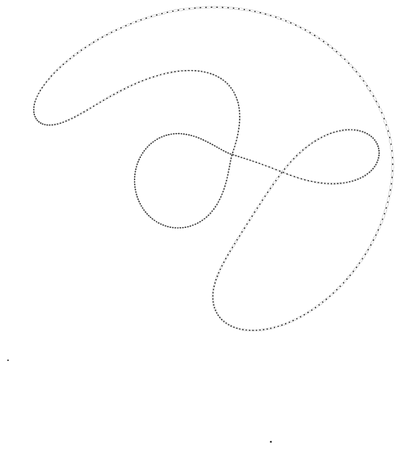	Finished circular assembly	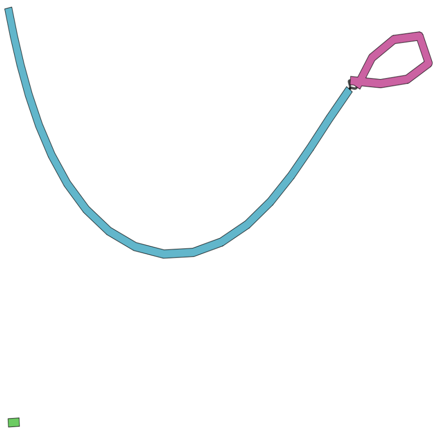
NCTC9021	*Escherichia coli*	33	1	0	1	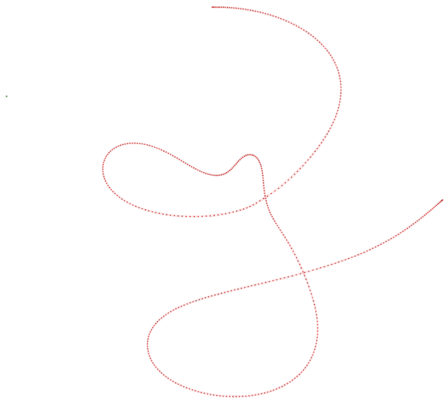	Finished assembly (lacking circularisation)	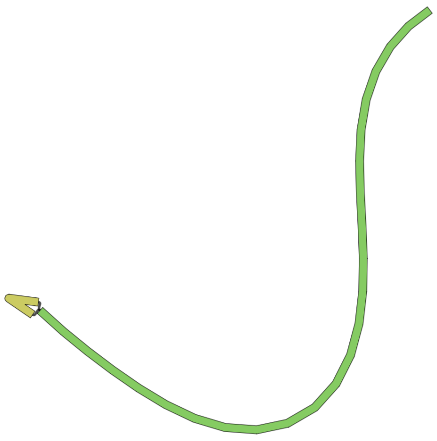
NCTC9022	*Escherichia coli*	68	1	0	0	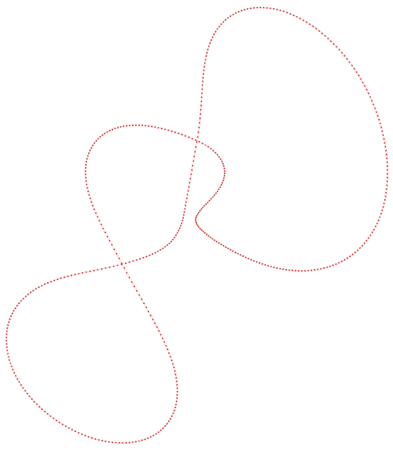	Finished circular assembly	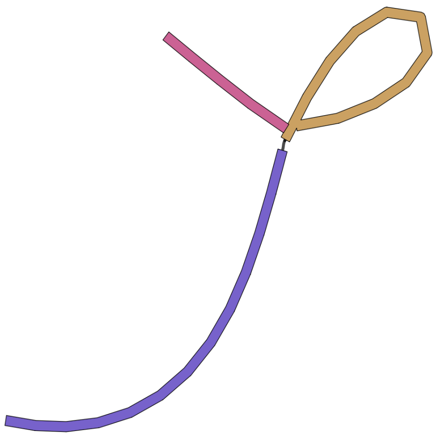
NCTC9023	*Escherichia coli*	57	0	0	2		Finished circular assembly	

						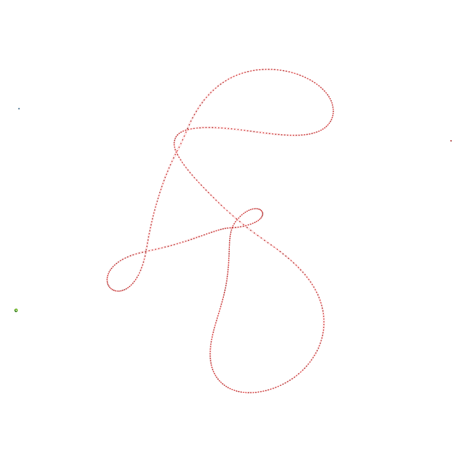		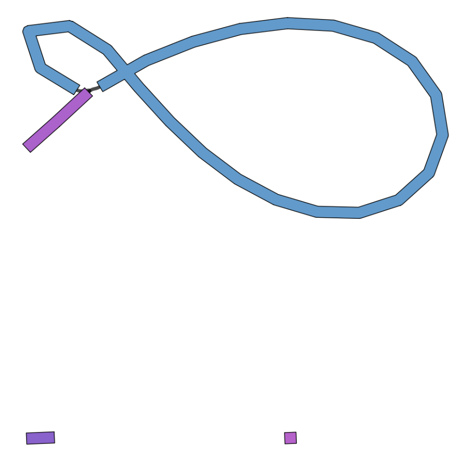
NCTC9024	*Escherichia coli*	40	0	0	3	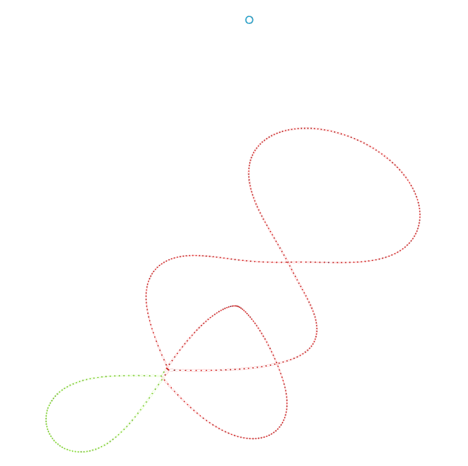	Finished assembly with multiple traversals	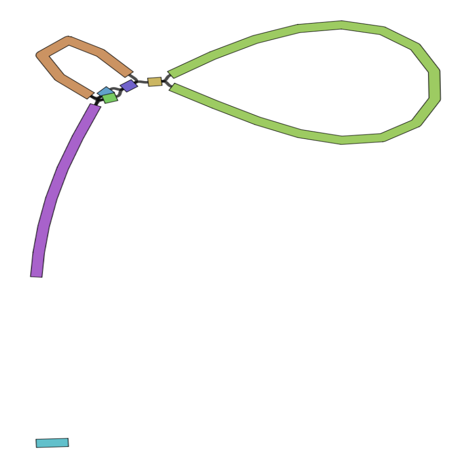
NCTC9026	*Escherichia coli*	69	1	1	0	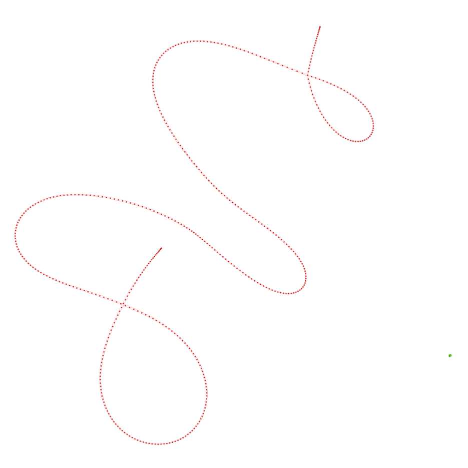	Possible mis-assembly	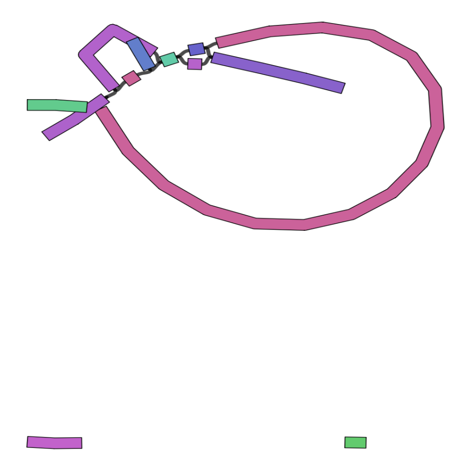
NCTC9029	*Escherichia coli*	47	1	2	4	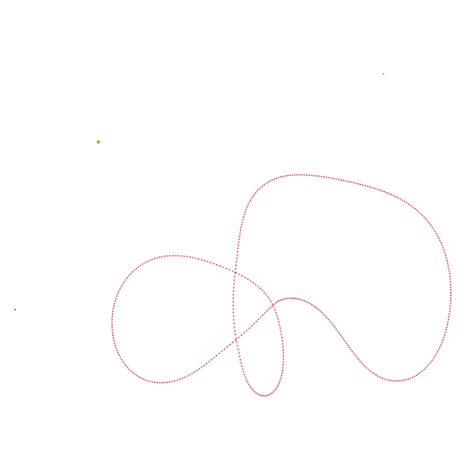	Finished circular assembly	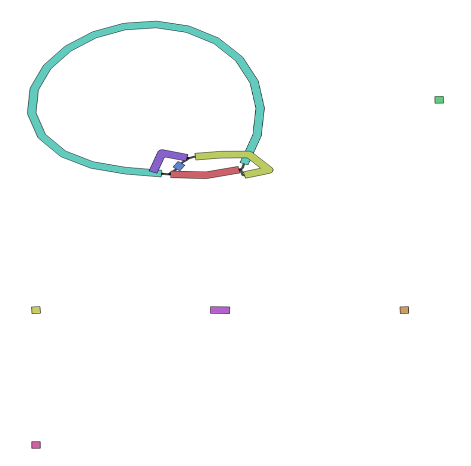
NCTC9030	*Escherichia coli*	61	1	1	1	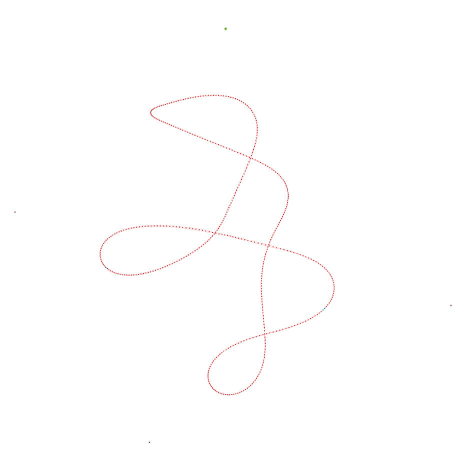	Finished circular assembly	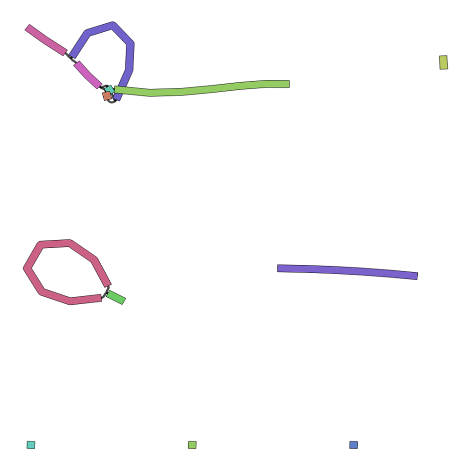
NCTC9031	*Escherichia coli*	78	1	1	0	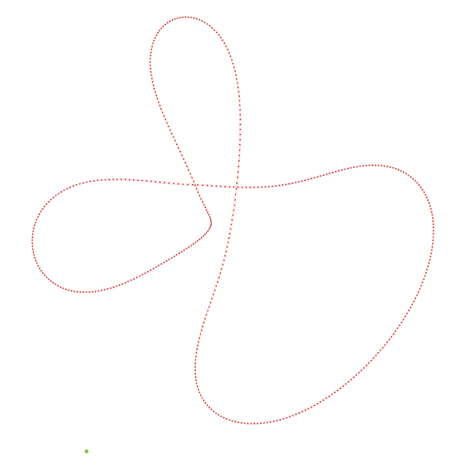	Finished circular assembly	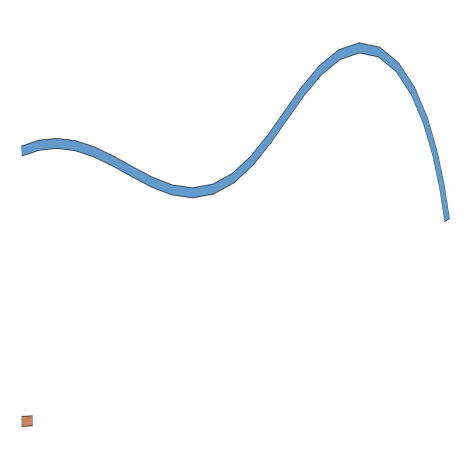
NCTC9032	*Escherichia coli*	70	0	0	1		Finished circular assembly	

						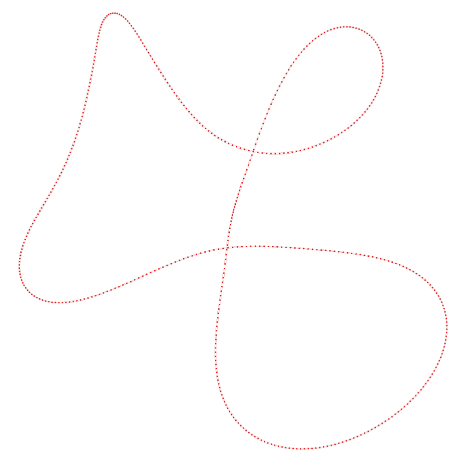		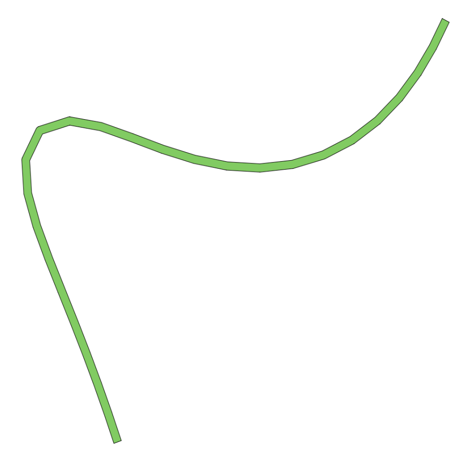
NCTC9033	*Escherichia coli*	36	1	0	0	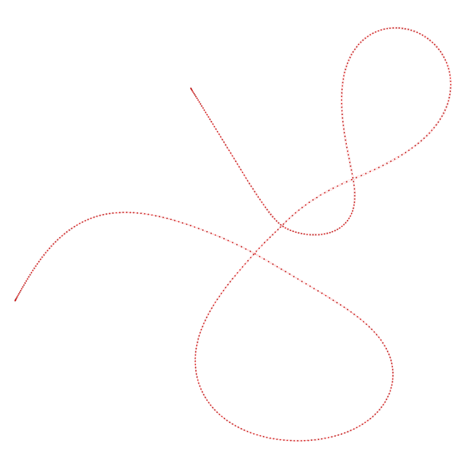	Finished assembly (lacking circularisation)	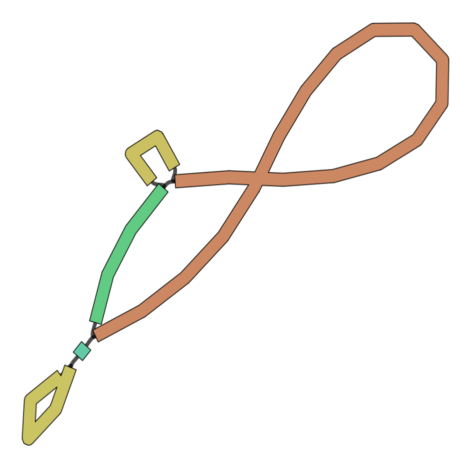
NCTC9034	*Escherichia coli*	80	0	0	3	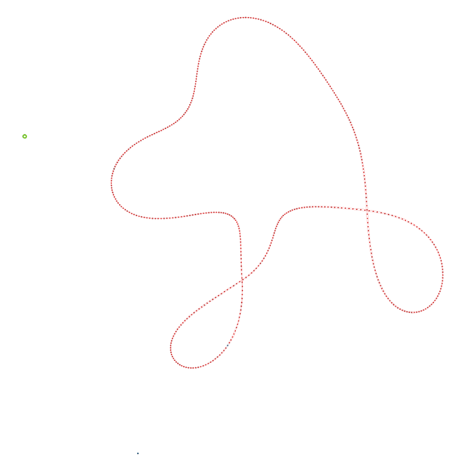	Finished circular assembly	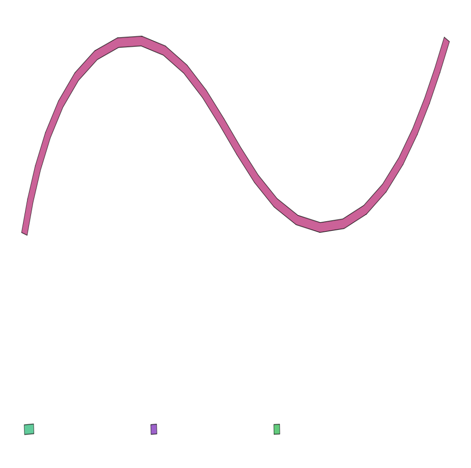
NCTC9035	*Escherichia coli*	64	1	0	1	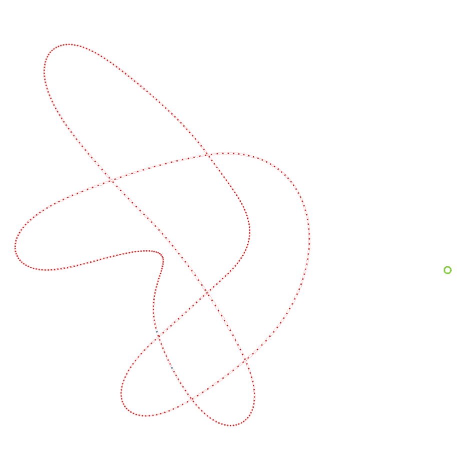	Finished circular assembly	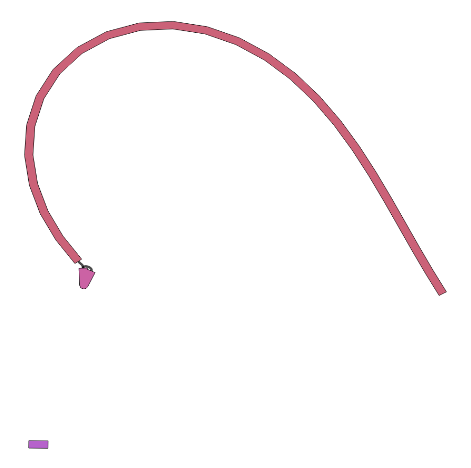
NCTC9038	*Escherichia coli*	65	1	0	0	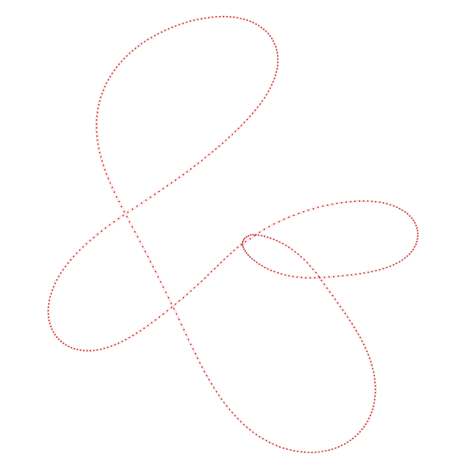	Finished circular assembly	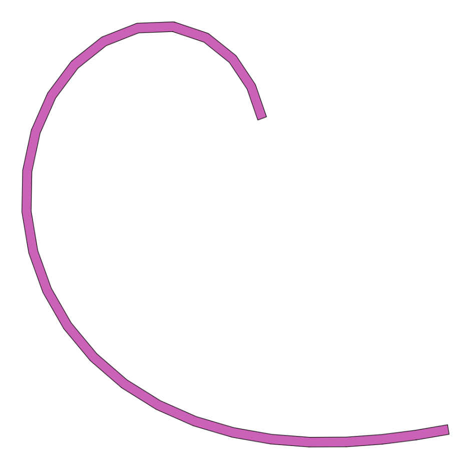
NCTC9040	*Escherichia coli*	58	1	0	0	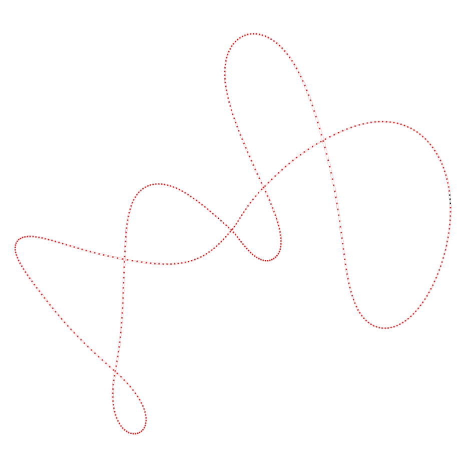	Finished circular assembly	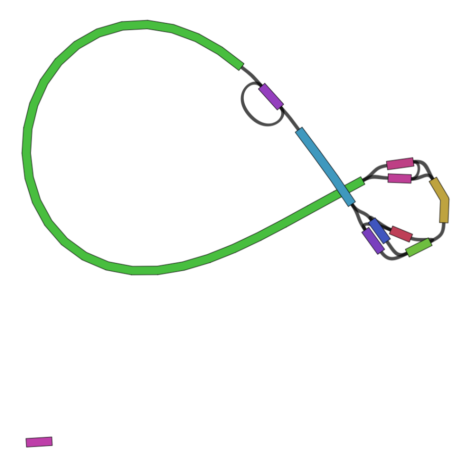
NCTC9043	*Escherichia coli*	74	1	1	0		Finished assembly with multiple traversals	

						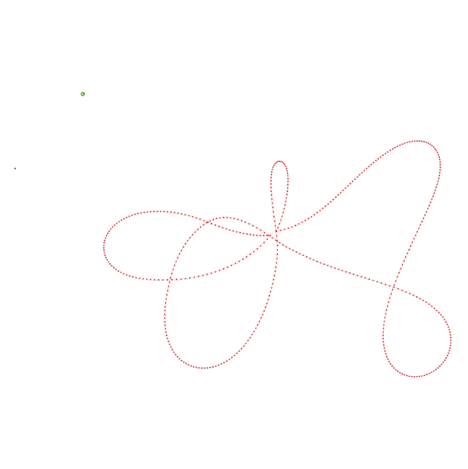		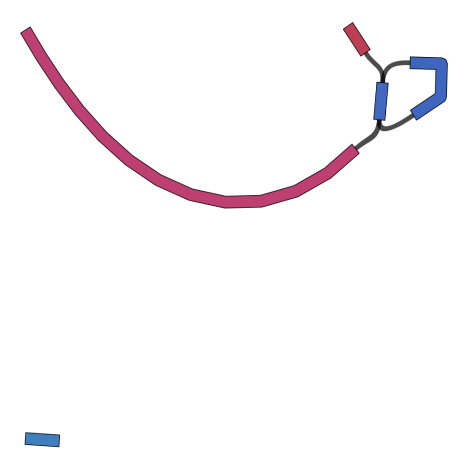
NCTC9044	*Escherichia coli*	26	Pending	Pending	Pending	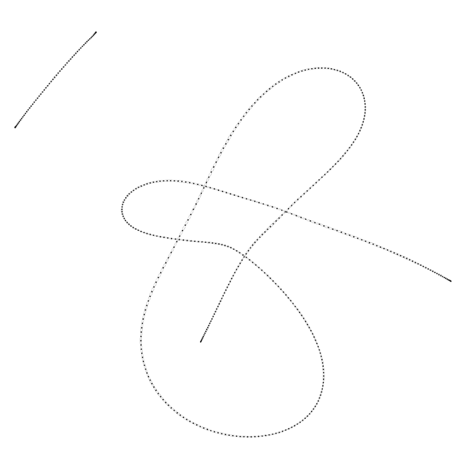	Mis-assembly/Fragmented	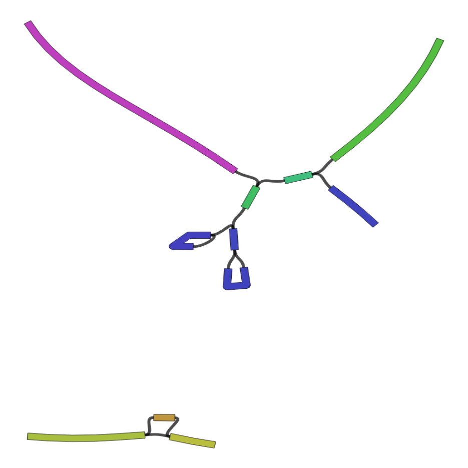
NCTC9045	*Escherichia coli*	32	1	2	1	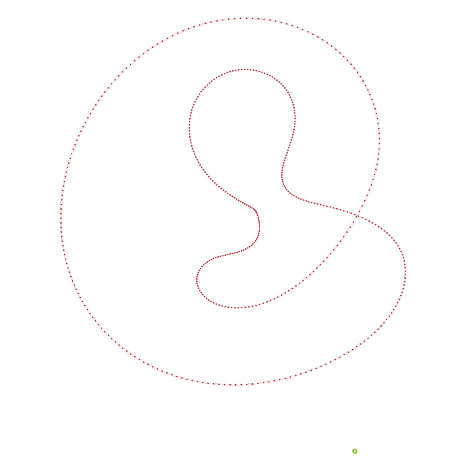	Finished circular assembly	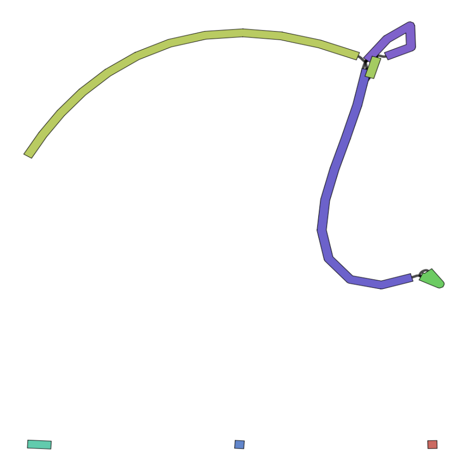
NCTC9047	*Escherichia coli*	87	1	0	4	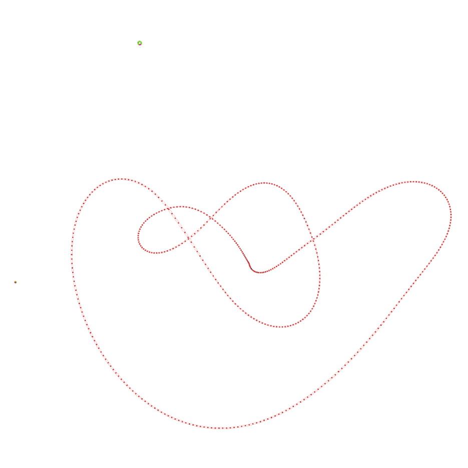	Finished circular assembly	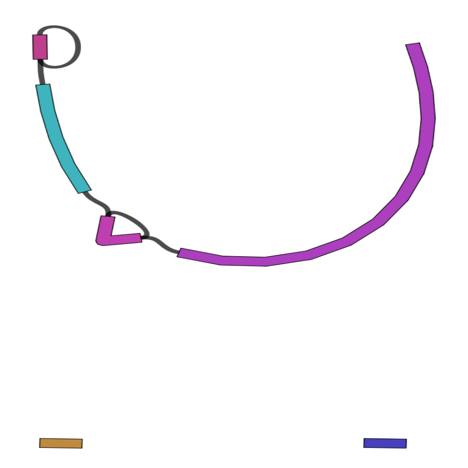
NCTC9048	*Escherichia coli*	52	1	1	1	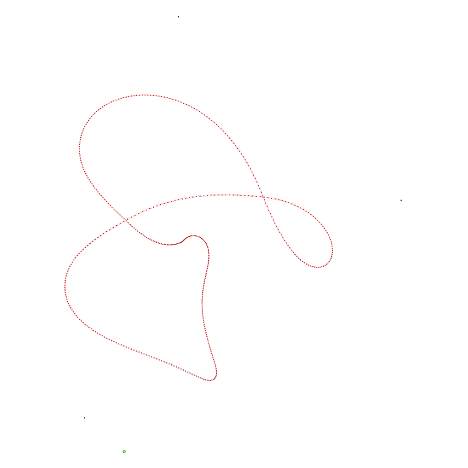	Finished circular assembly	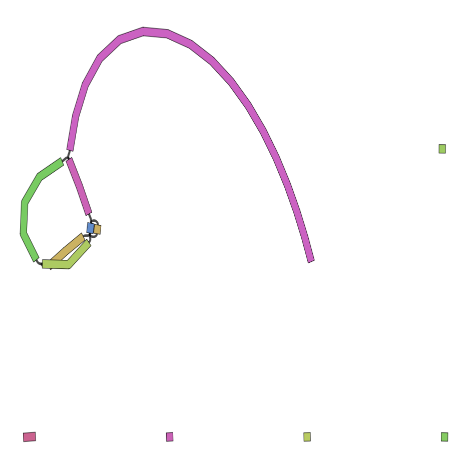
NCTC9049	*Escherichia coli*	49	1	1	0	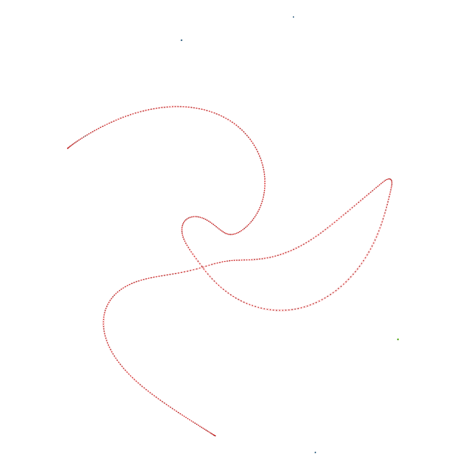	Finished assembly (lacking circularisation)	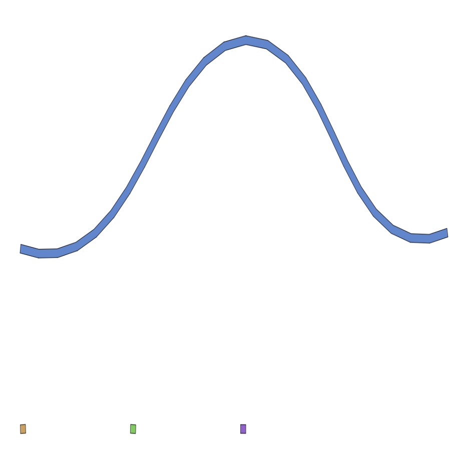
NCTC9050	*Escherichia coli*	70	0	0	4		Finished circular assembly	

						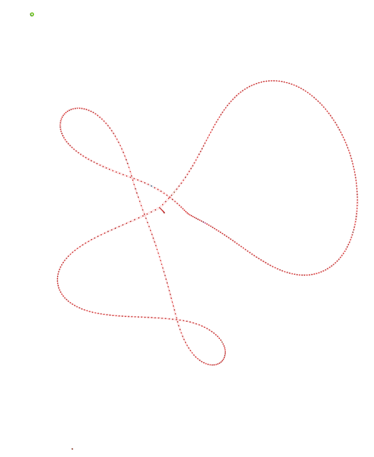		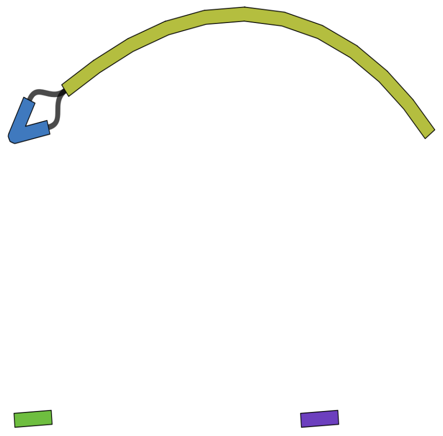
NCTC9052	*Escherichia coli*	48	1	1	0	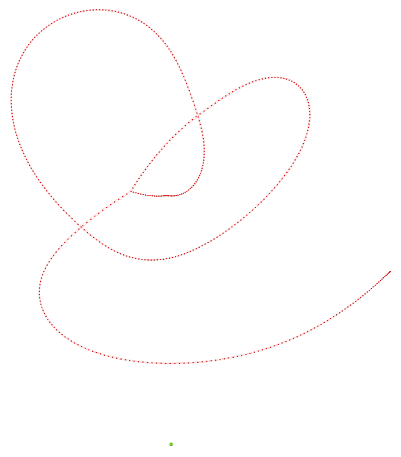	Mis-assembly/Fragmented	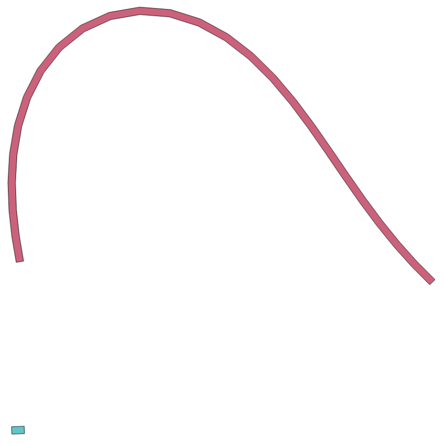
NCTC9053	*Escherichia coli*	45	1	0	1	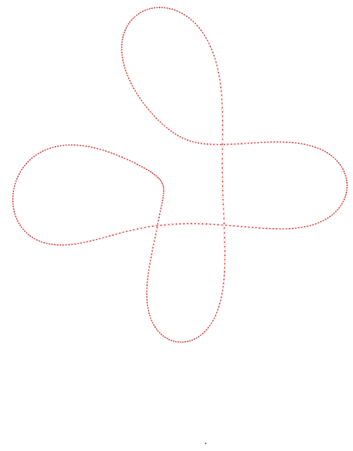	Finished circular assembly	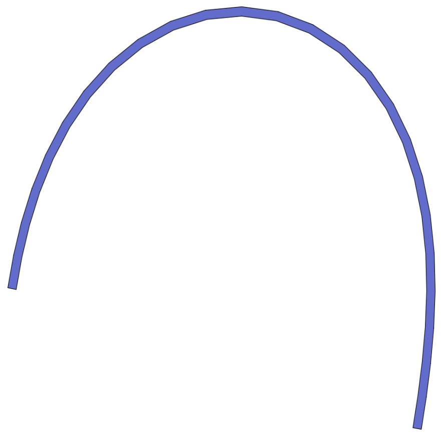
NCTC9054	*Escherichia coli*	35	1	0	0	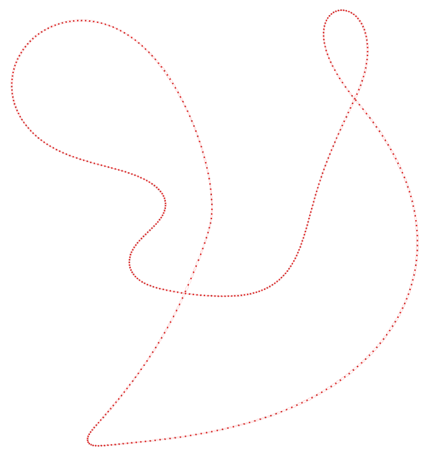	Finished circular assembly	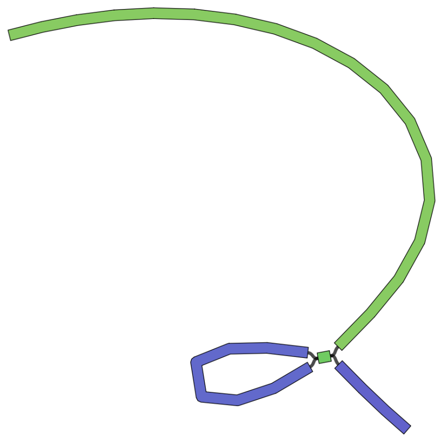
NCTC9055	*Escherichia coli*	70	0	0	4	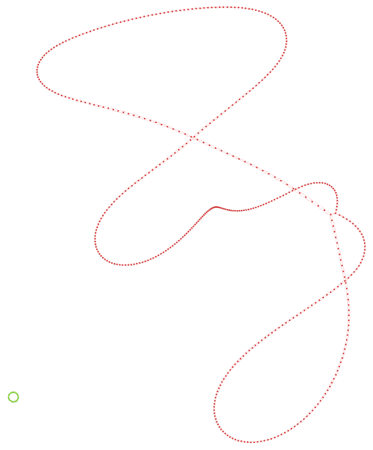	Finished assembly with multiple traversals	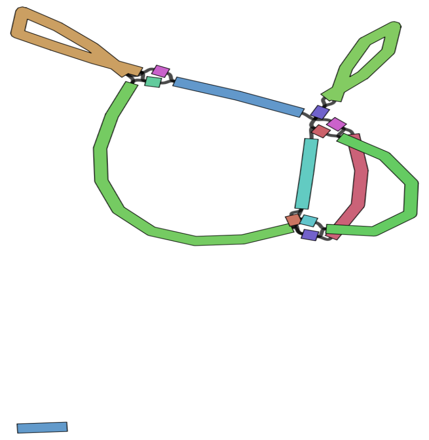
NCTC9058	*Escherichia coli*	90	0	0	4	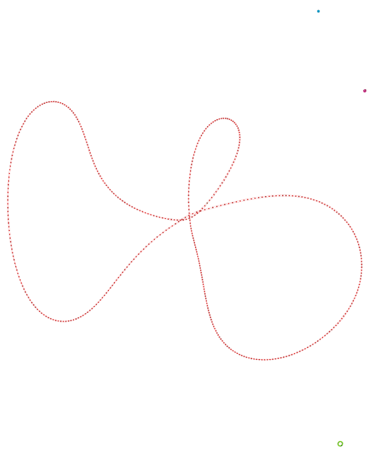	Finished circular assembly	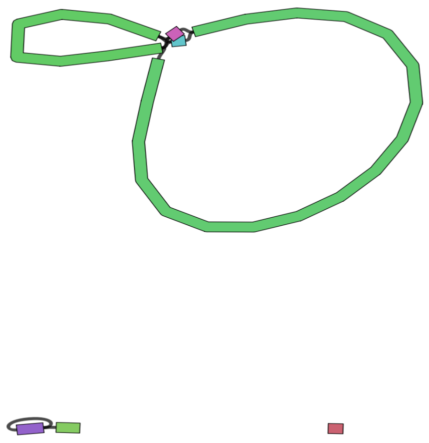
NCTC9060	*Escherichia coli*	91	0	0	2		Finished circular assembly	

						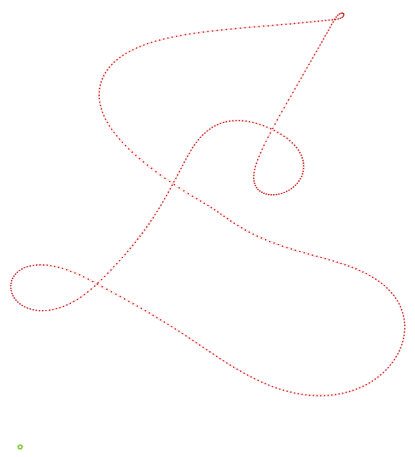		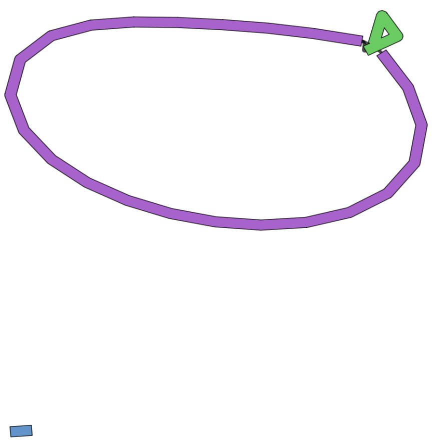
NCTC9062	*Escherichia coli*	67	0	0	5	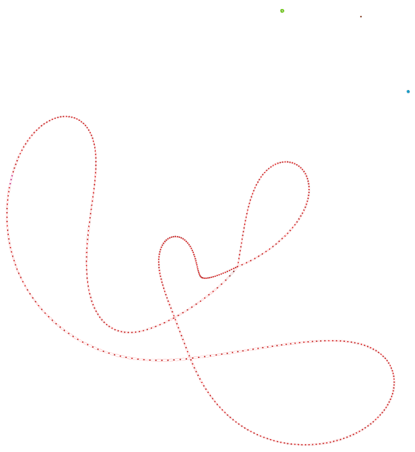	Finished assembly with multiple traversals	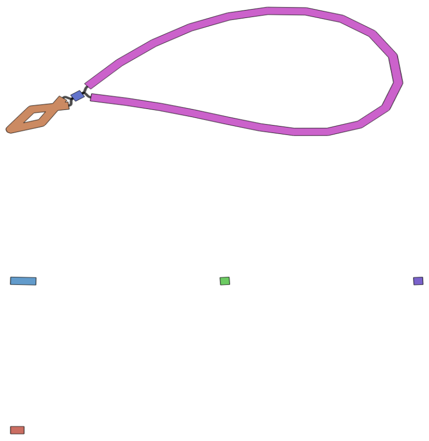
NCTC9065	*Escherichia coli*	51	1	1	0	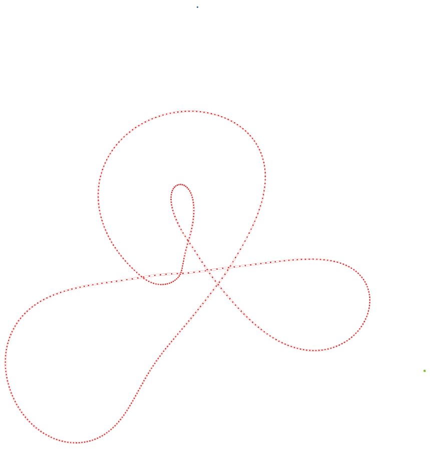	Finished circular assembly	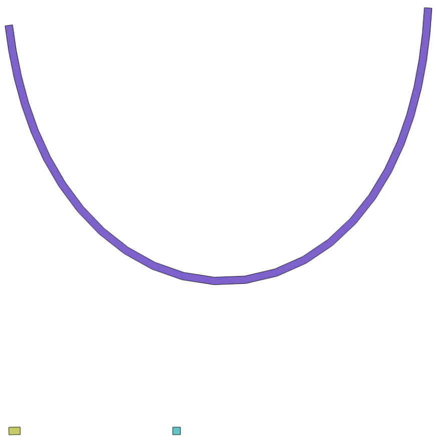
NCTC9066	*Escherichia coli*	89	1	0	0	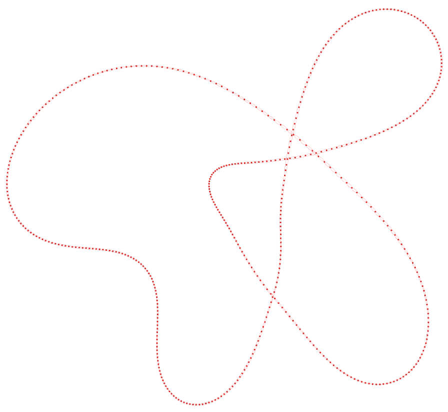	Finished circular assembly	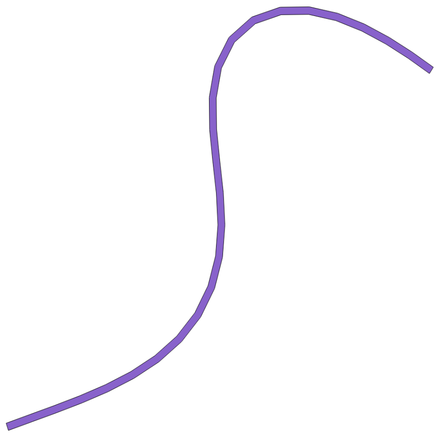
NCTC9067	*Escherichia coli*	55	Pending	Pending	Pending	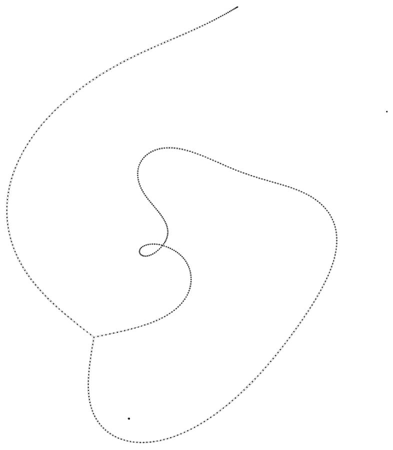	Mis-assembly/Fragmented	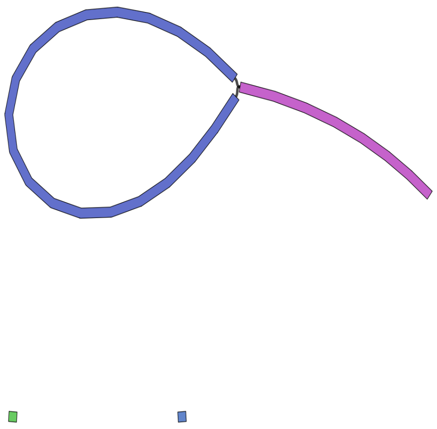
NCTC9068	*Escherichia coli*	74	1	2	1	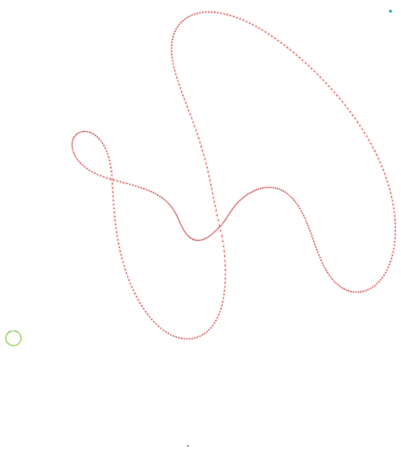	Finished circular assembly	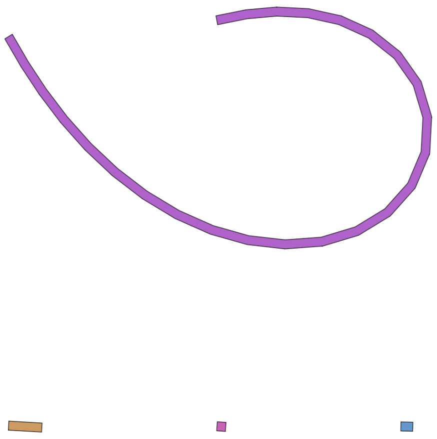
NCTC9069	*Escherichia coli*	75	1	1	0		Finished assembly (lacking circularisation)	

						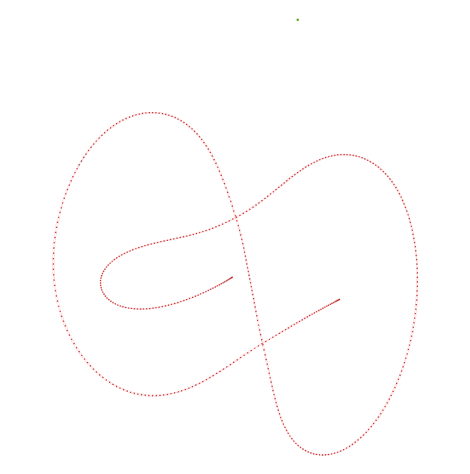		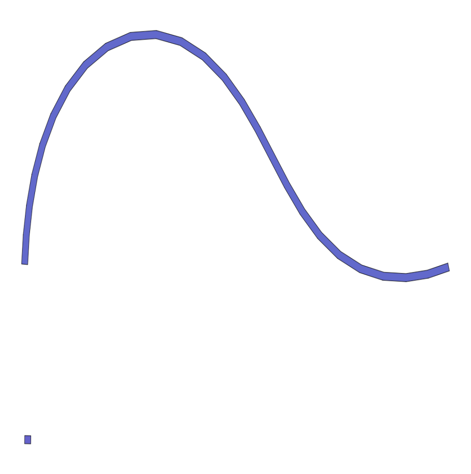
NCTC9070	*Escherichia coli*	76	1	1	2	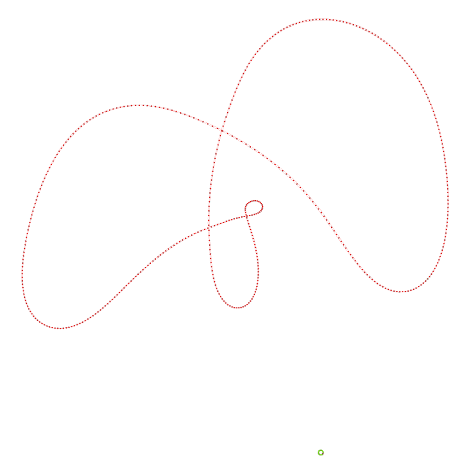	Finished circular assembly	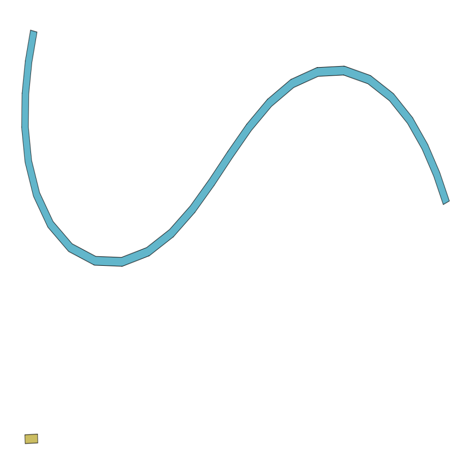
NCTC9071	*Escherichia coli*	65	1	0	2	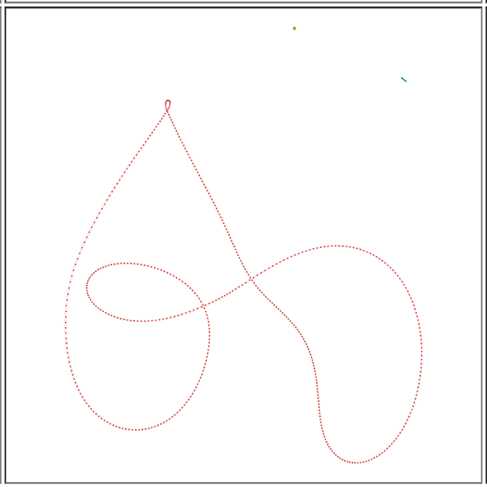	Finished circular assembly	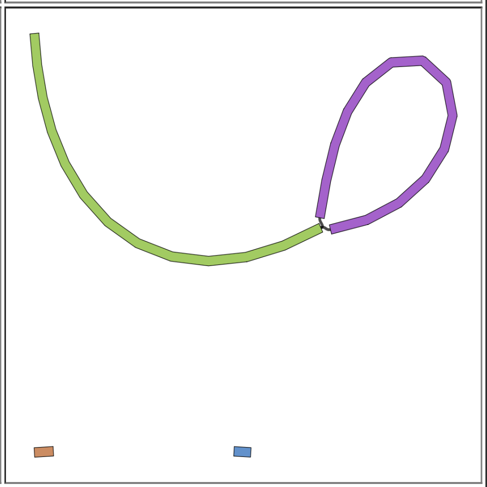
NCTC9073	*Escherichia coli*	18	0	0	12	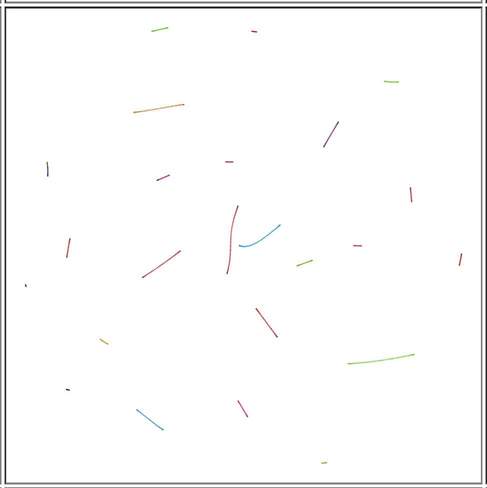	Mis-assembly/Fragmented	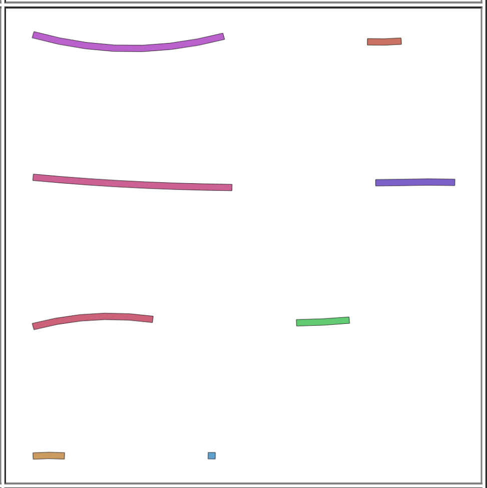
NCTC9075	*Escherichia coli*	35	1	0	3	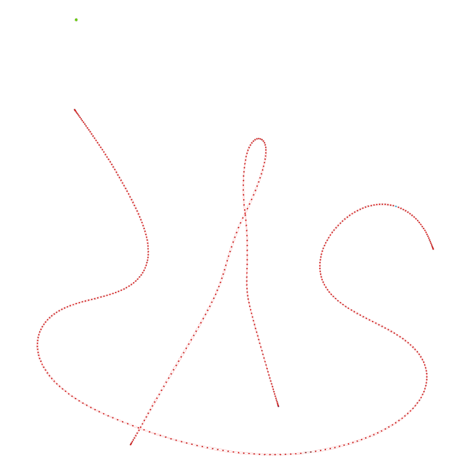	Mis-assembly/Fragmented	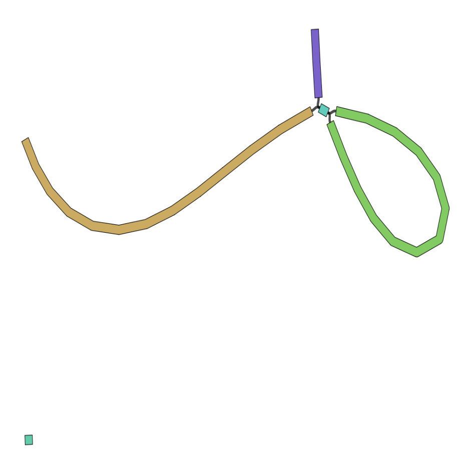
NCTC9076	*Escherichia coli*	72	0	0	2	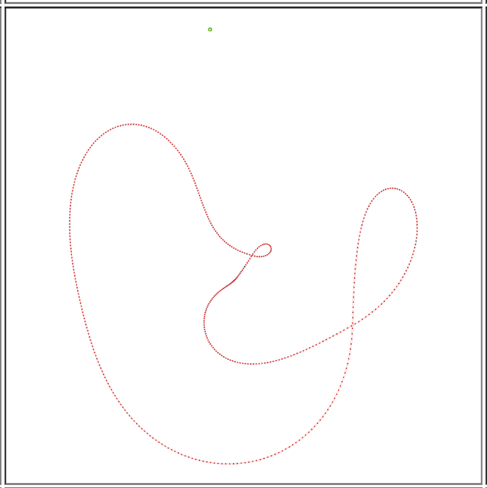	Finished circular assembly	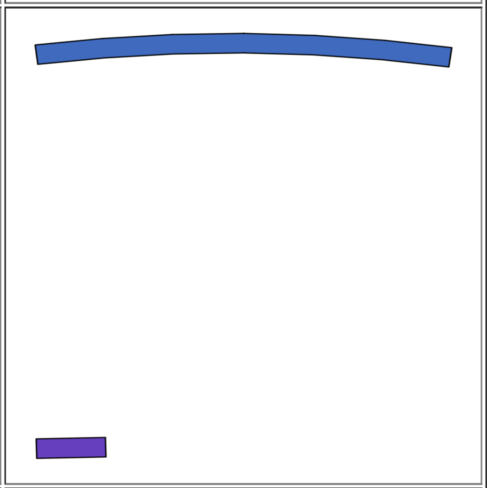
NCTC9077	*Escherichia coli*	31	1	0	2		Finished circular assembly	

						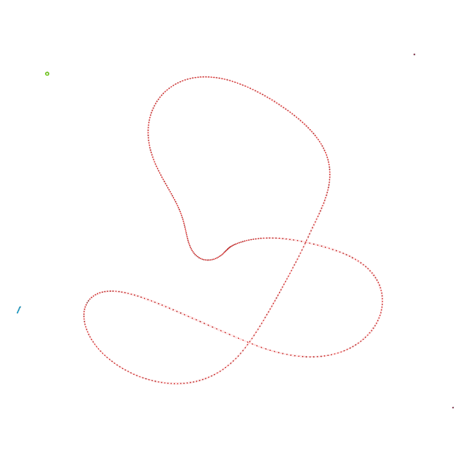		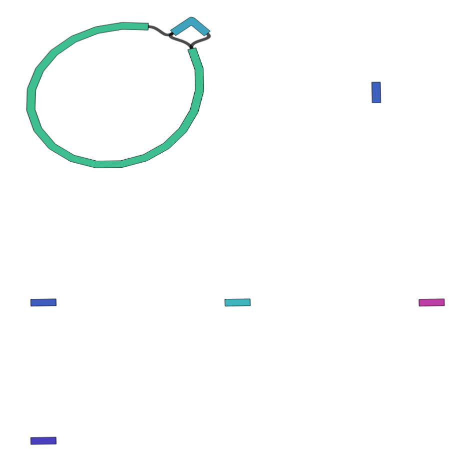
NCTC9078	*Escherichia coli*	55	1	2	2	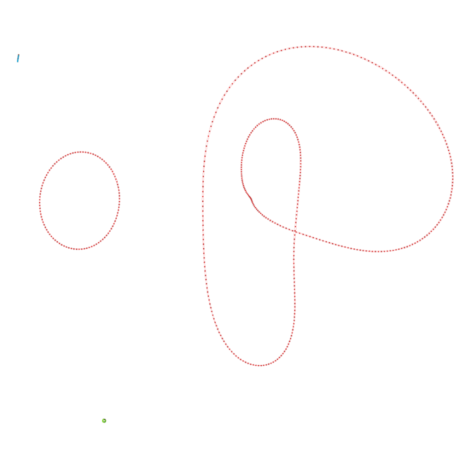	Mis-assembly	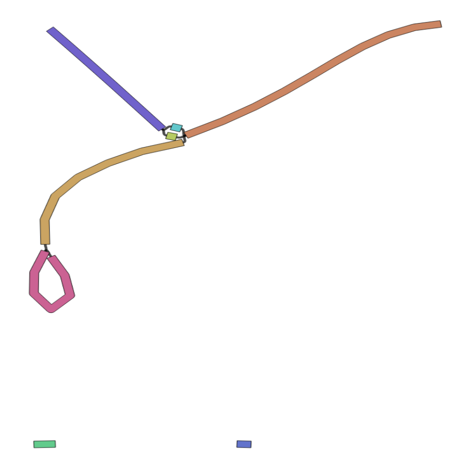
NCTC9079	*Escherichia coli*	87	1	0	1	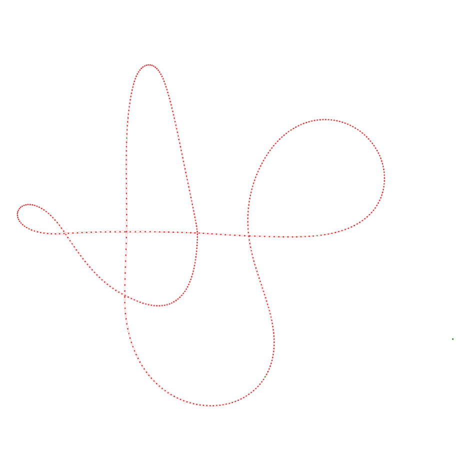	Finished circular assembly	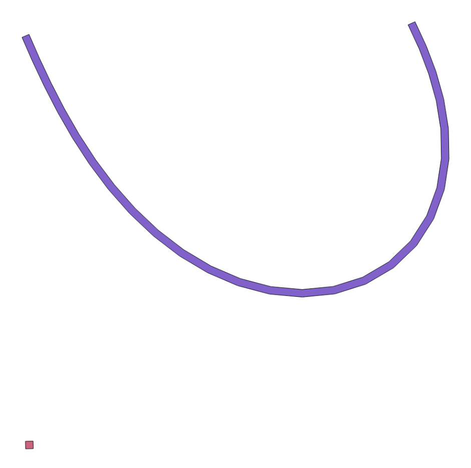
NCTC9081	*Escherichia coli*	0	1	2	5	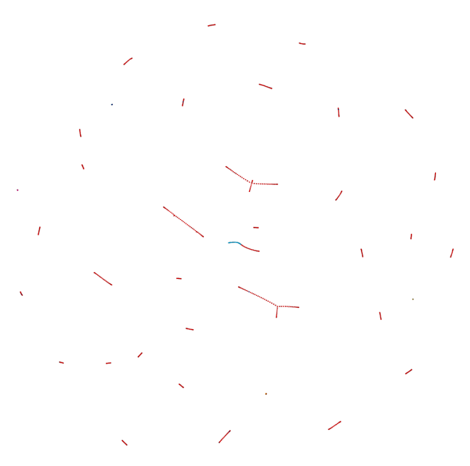	Mis-assembly/Fragmented	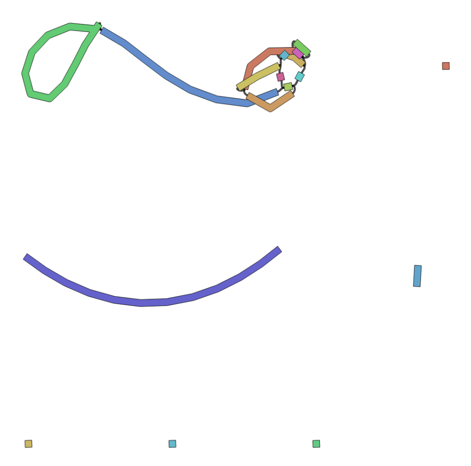
NCTC9082	*Escherichia coli*	62	1	0	0	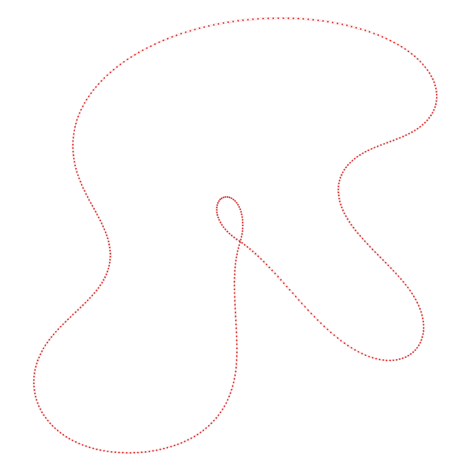	Finished circular assembly	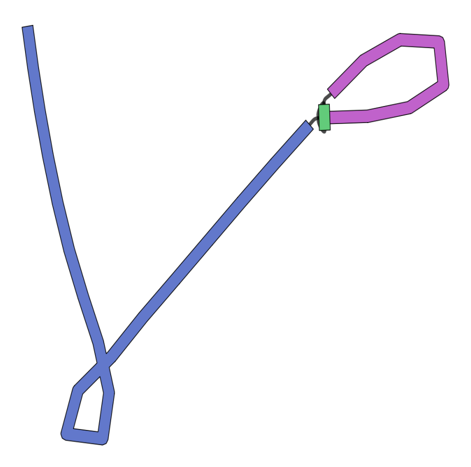
NCTC9084	*Escherichia coli*	59	1	0	0	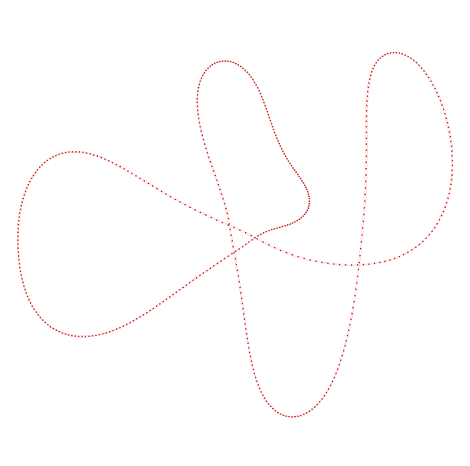	Finished circular assembly	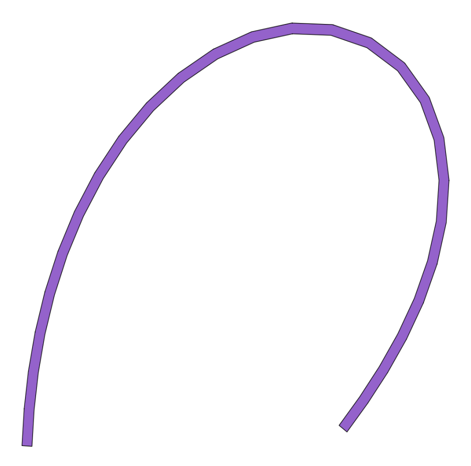
NCTC9085	*Escherichia coli*	50	1	0	0		Finished circular assembly	

						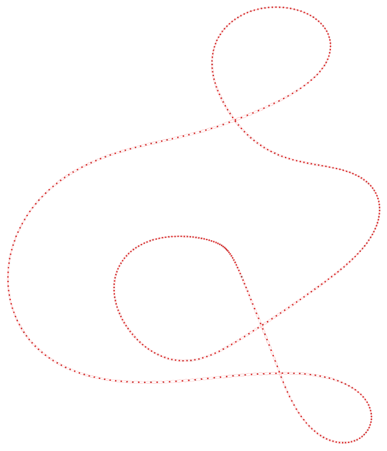		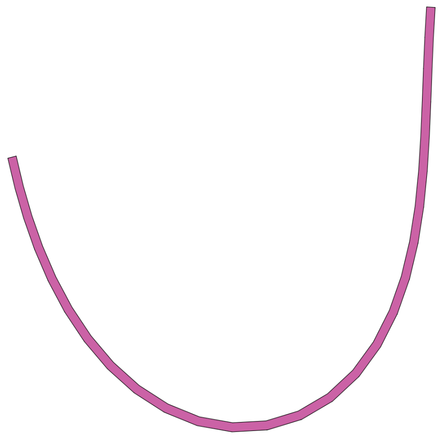
NCTC9086	*Escherichia coli*	69	1	2	0	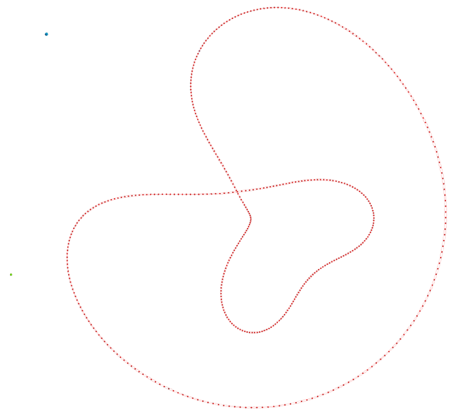	Finished circular assembly	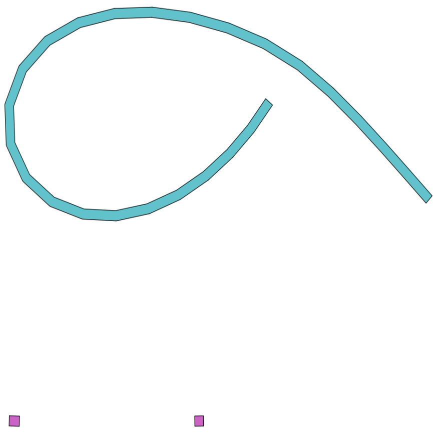
NCTC9087	*Escherichia coli*	94	1	0	0	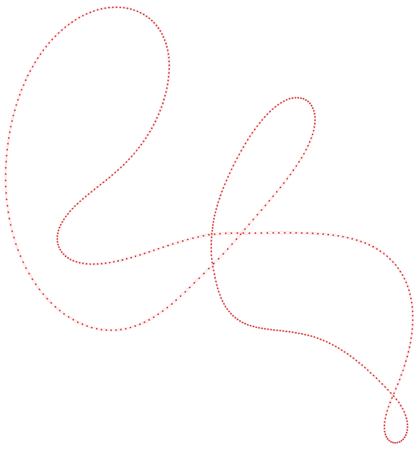	Finished circular assembly	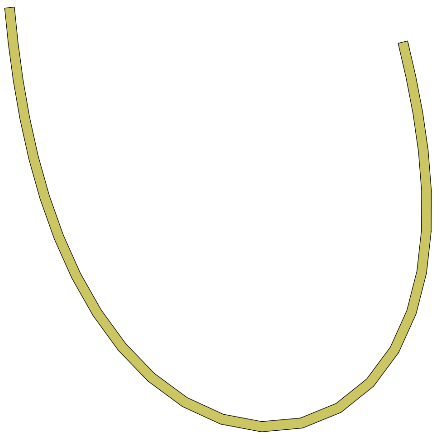
NCTC9088	*Escherichia coli*	78	1	0	0	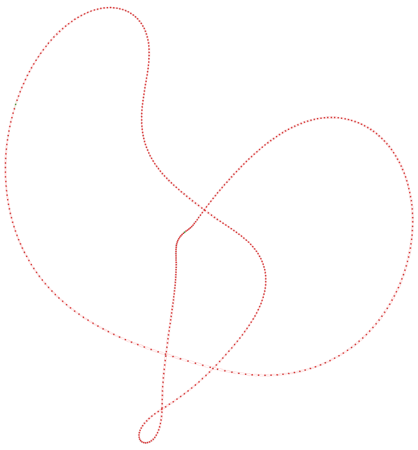	Finished circular assembly	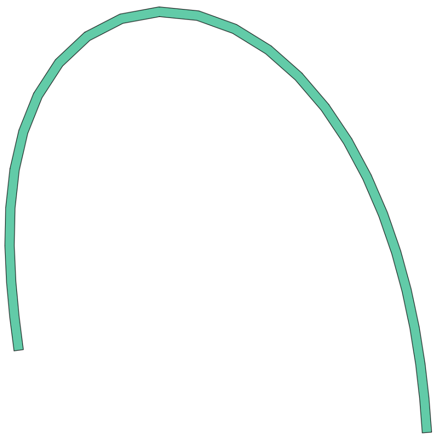
NCTC9089	*Escherichia coli*	40	1	1	0	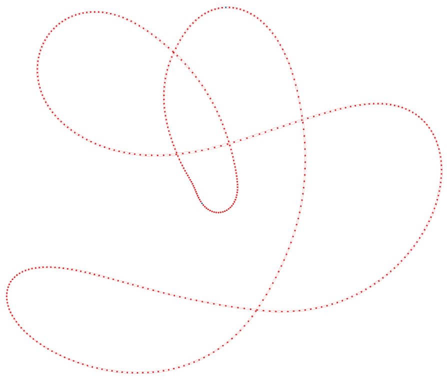	Finished circular assembly	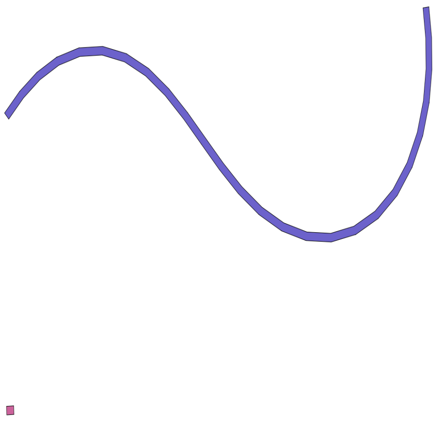
NCTC9091	*Escherichia coli*	86	1	0	1	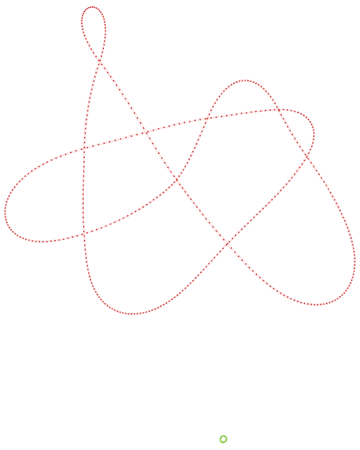	Finished circular assembly	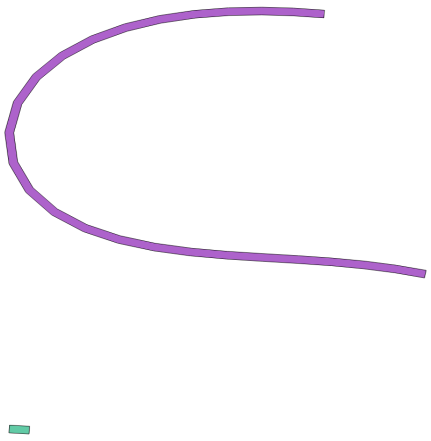
NCTC9092	*Escherichia coli*	62	0	0	4		Finished assembly with multiple traversals	

						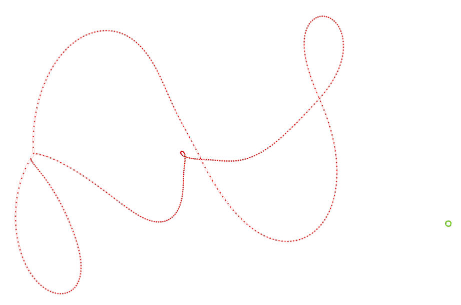		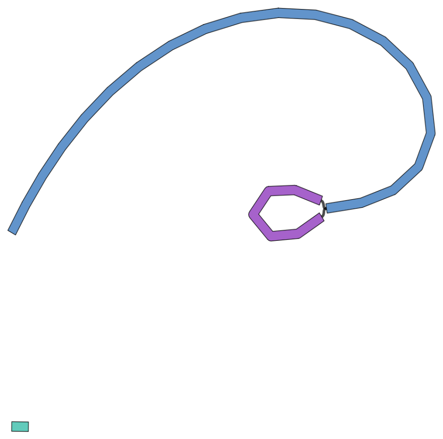
NCTC9095	*Escherichia coli*	64	Pending	Pending	Pending	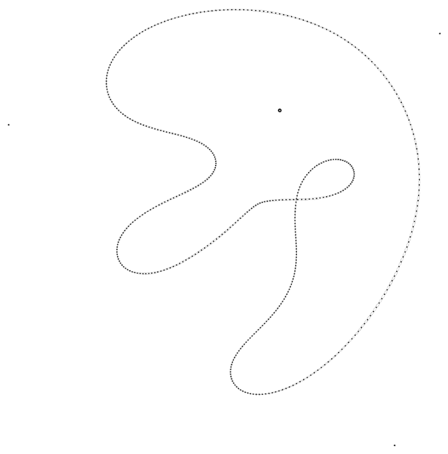	Finished circular assembly	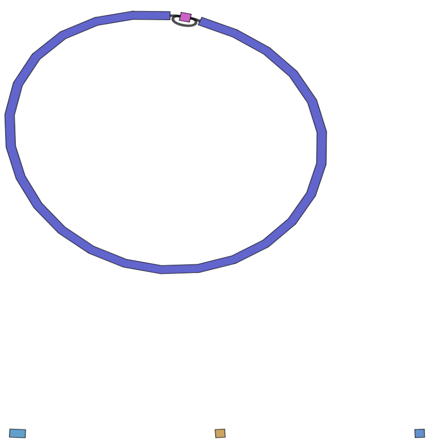
NCTC9096	*Escherichia coli*	77	1	1	1	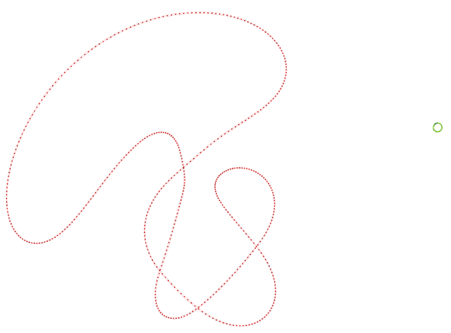	Finished circular assembly	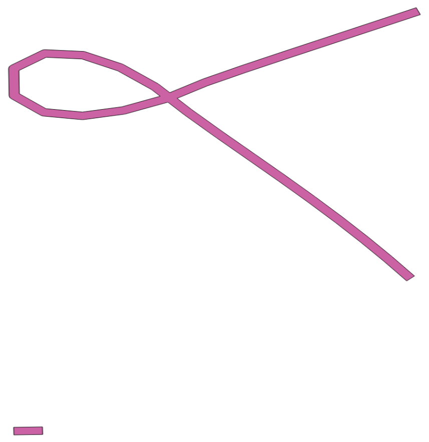
NCTC9097	*Escherichia coli*	54	1	1	0	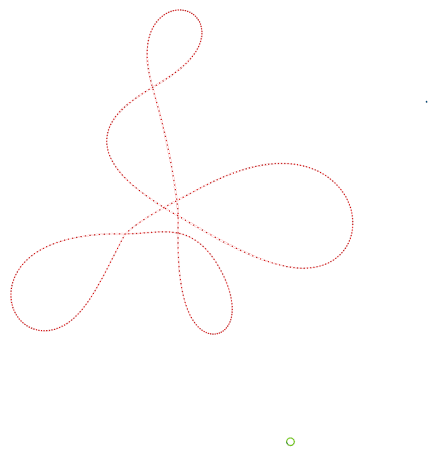	Finished circular assembly	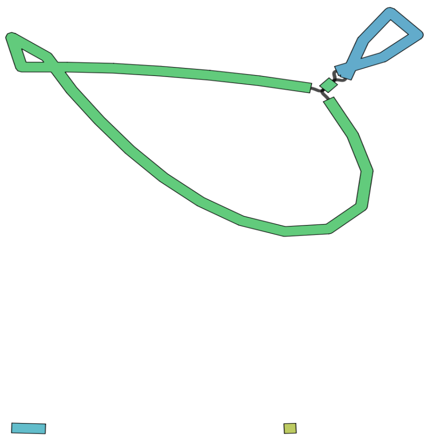
NCTC9098	*Escherichia coli*	56	1	1	2	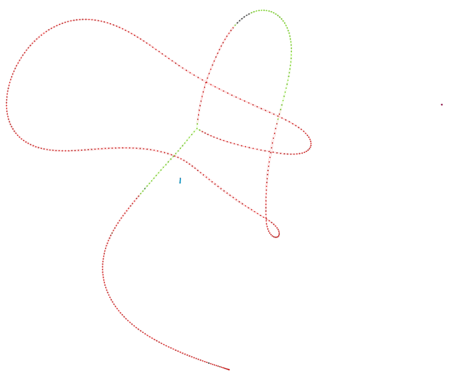	Mis-assembly/Fragmented	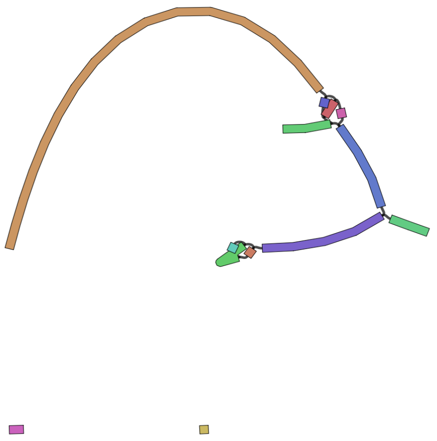
NCTC9099	*Escherichia coli*	66	1	1	4	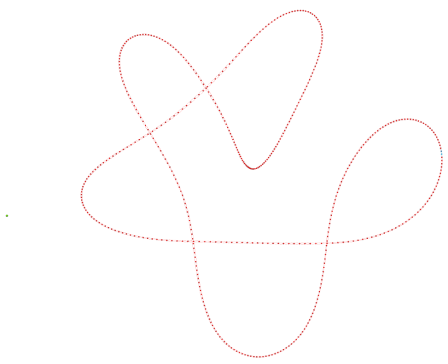	Finished circular assembly	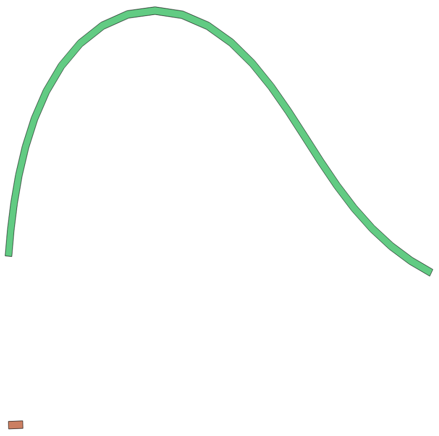
NCTC9100	*Escherichia coli*	80	1	0	0		Finished circular assembly	

						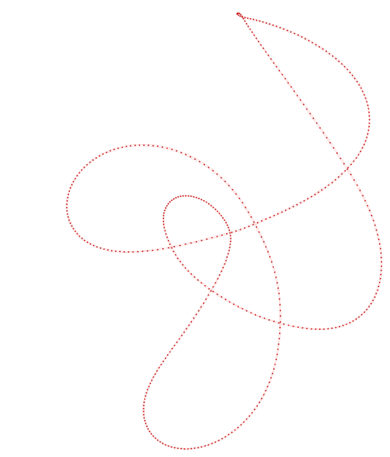		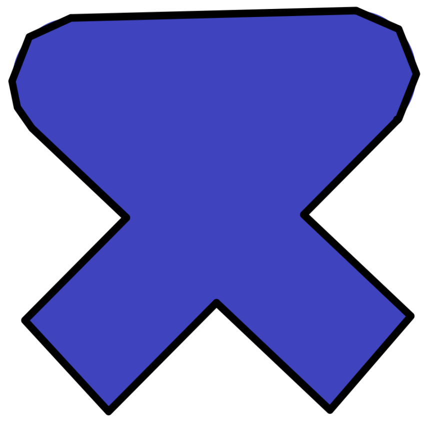
NCTC9101	*Escherichia coli*	84	1	3	0	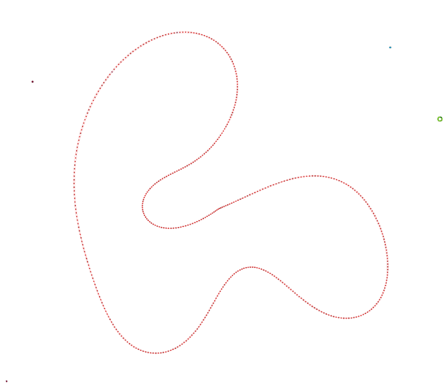	Finished circular assembly	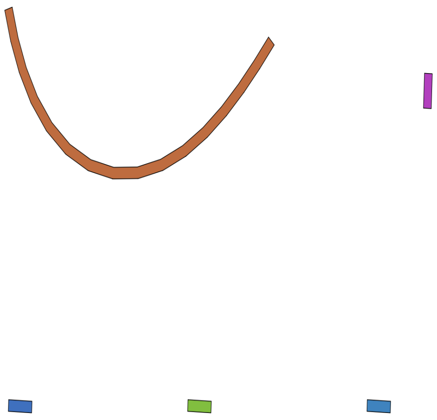
NCTC9102	*Escherichia coli*	51	1	0	0	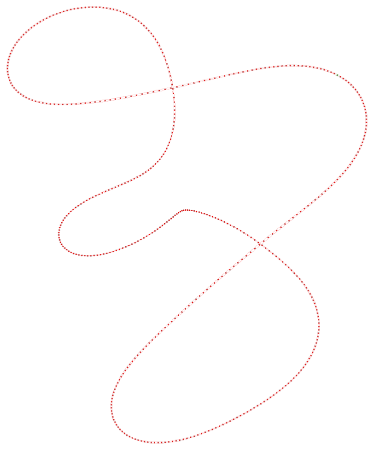	Finished circular assembly	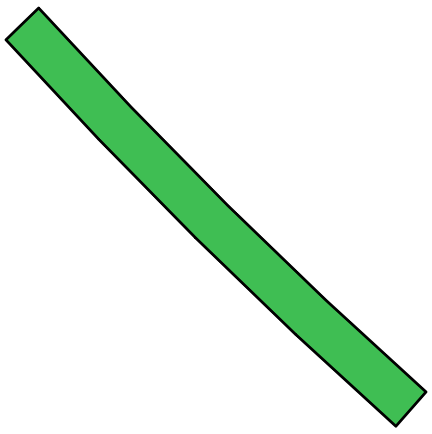
NCTC9103	*Escherichia coli*	98	0	0	18	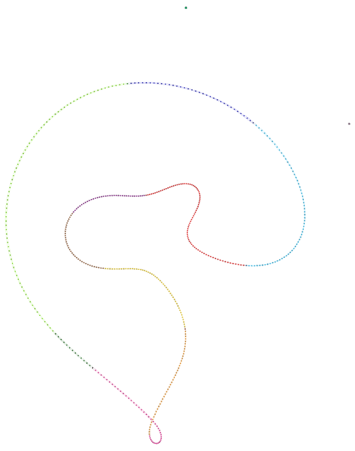	Finished circular assembly	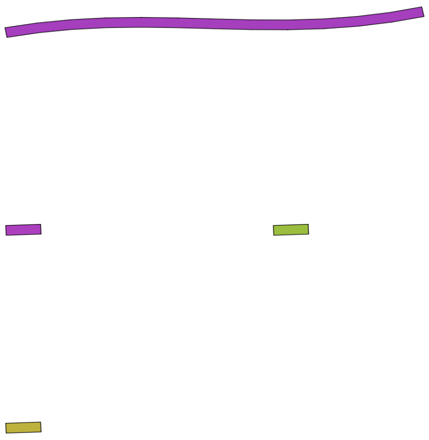
NCTC9104	*Escherichia coli*	65	1	0	0	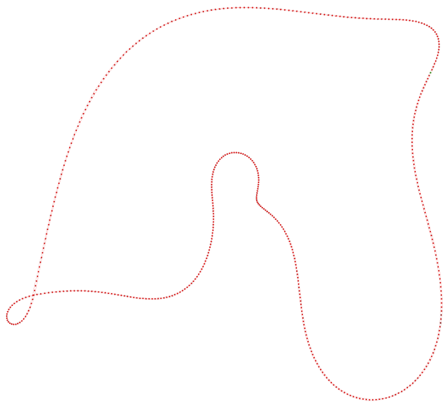	Finished circular assembly	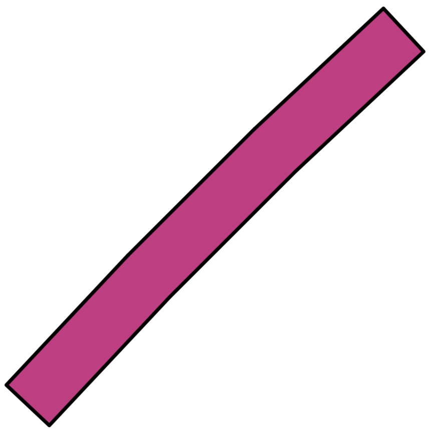
NCTC9107	*Escherichia coli*	57	1	0	0	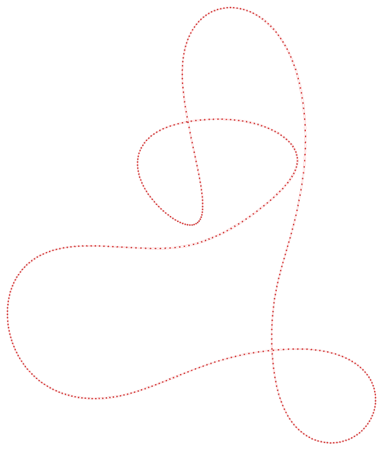	Finished circular assembly	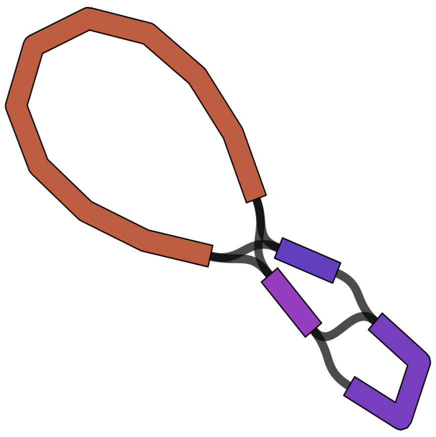
NCTC9108	*Escherichia coli*	49	1	2	0		Finished circular assembly	

						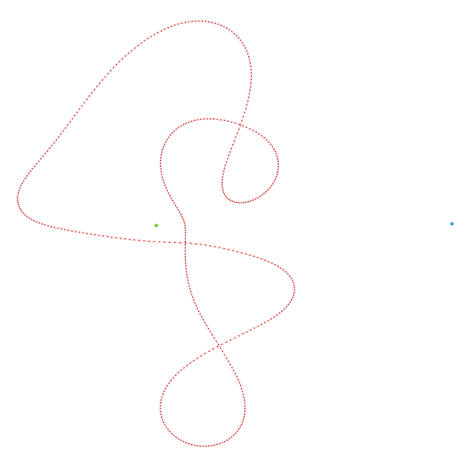		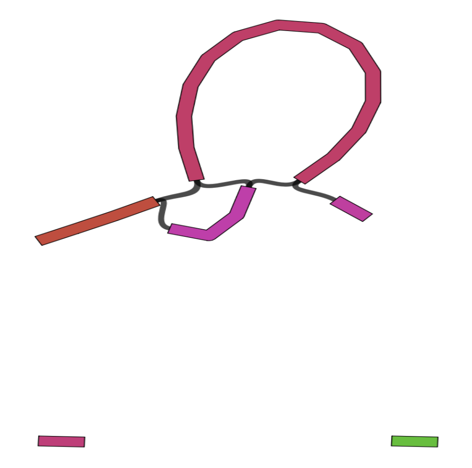
NCTC9109	*Escherichia coli*	61	1	1	1	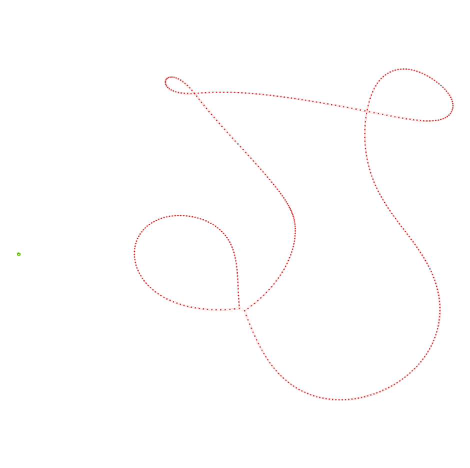	Finished assembly with multiple traversals	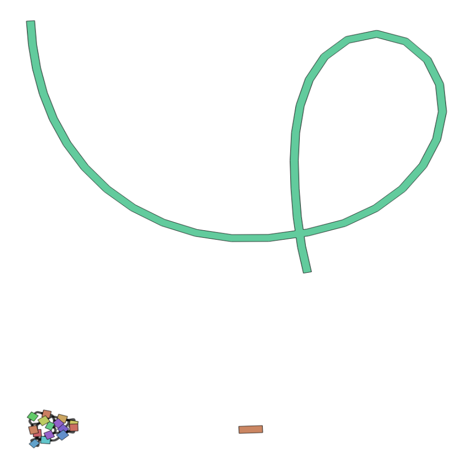
NCTC9110	*Escherichia coli*	73	1	0	1	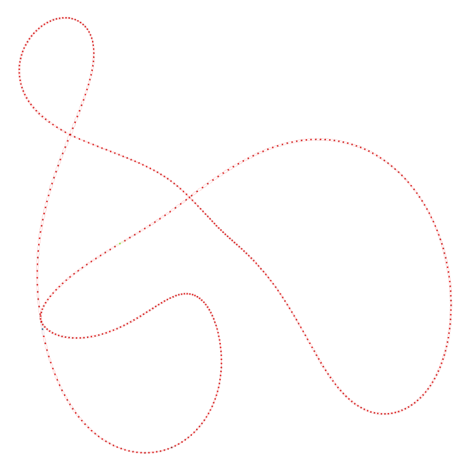	Finished circular assembly	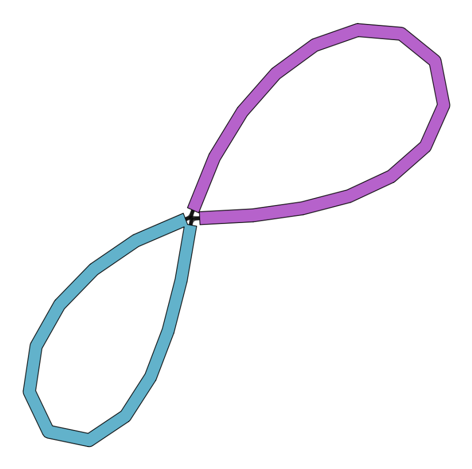
NCTC9111	*Escherichia coli*	62	1	1	9	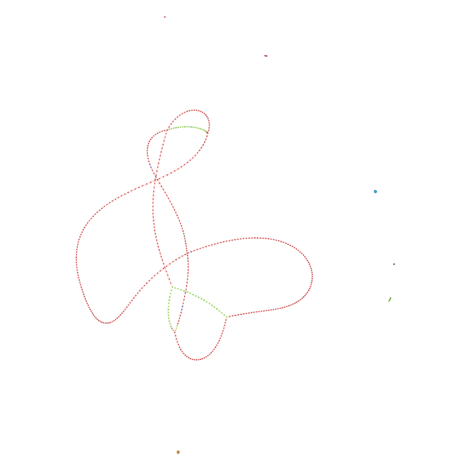	Mis-assembly/Fragmented	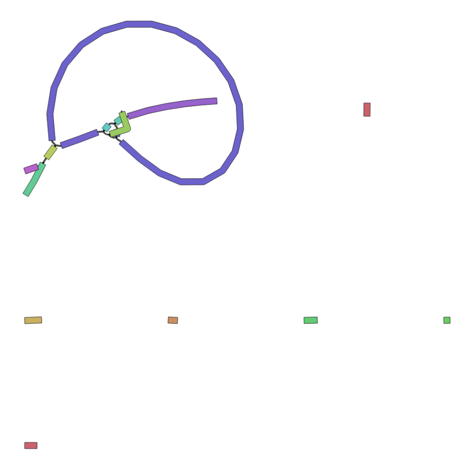
NCTC9112	*Escherichia coli*	69	1	0	0	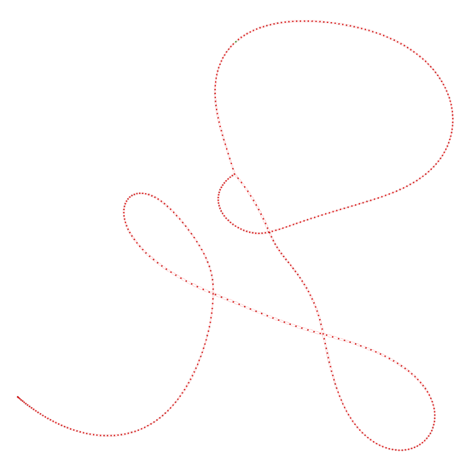	Mis-assembly/Fragmented	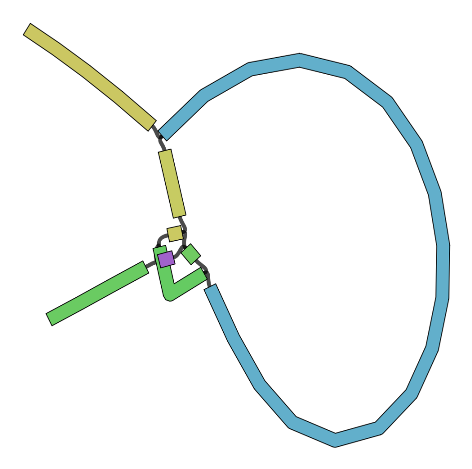
NCTC9114	*Escherichia coli*	78	1	0	2	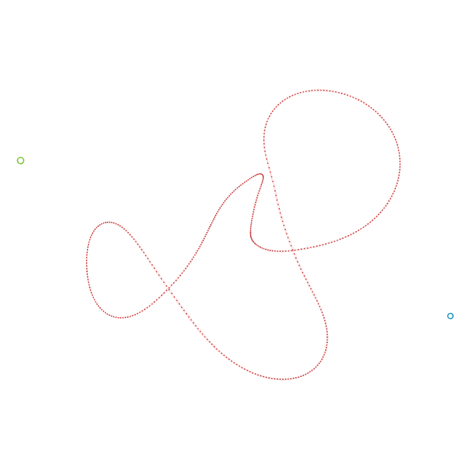	Finished circular assembly	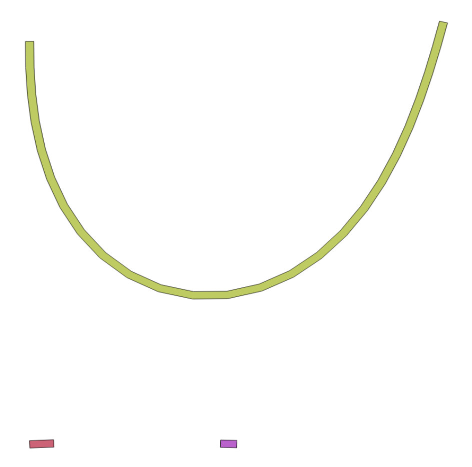
NCTC9116	*Escherichia coli*	49	1	0	2		Finished circular assembly	

						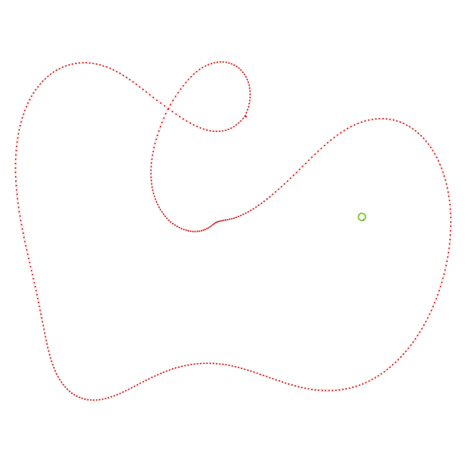		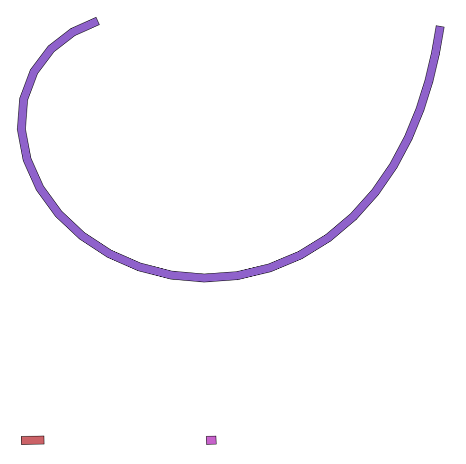	
NCTC9117	*Escherichia coli*	36	1	0	5	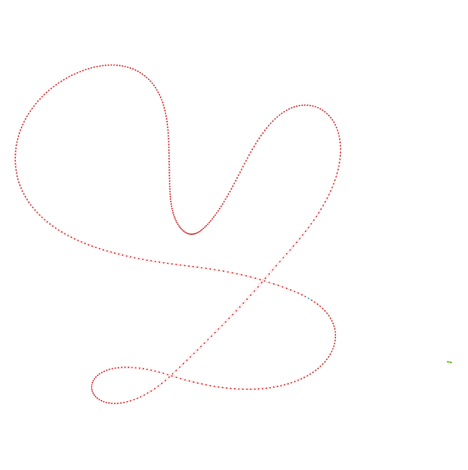	Finished circular assembly	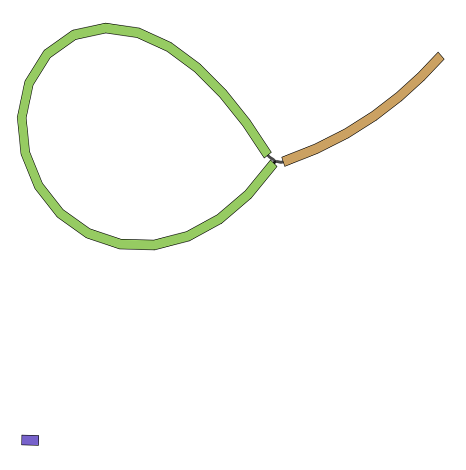	
NCTC9120	*Escherichia coli*	66	1	1	3	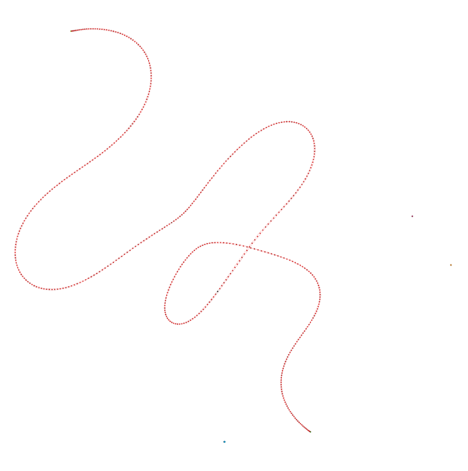	Finished assembly (lacking circularisation)	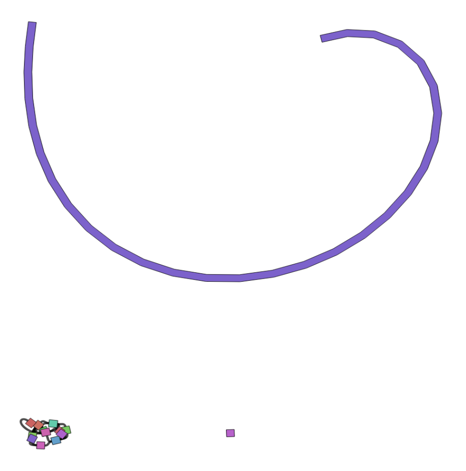	
NCTC9121	*Escherichia coli*	51	1	1	2	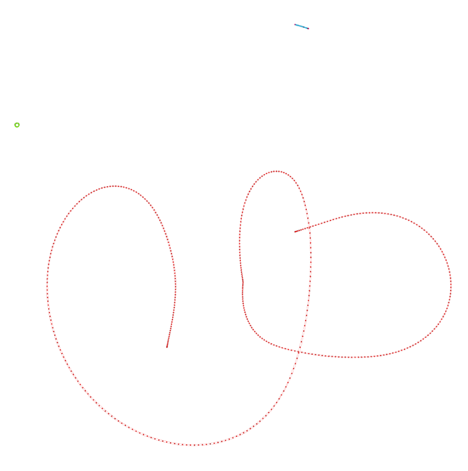	Finished assembly (lacking circularisation)	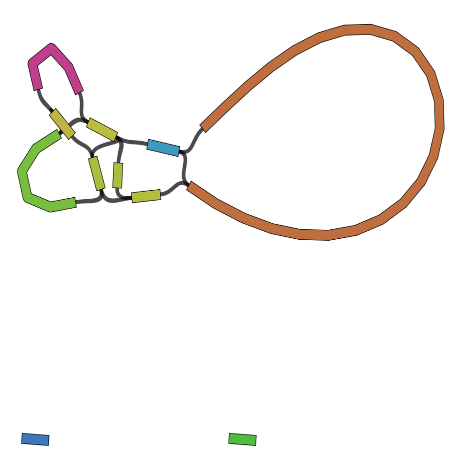	
NCTC9123	*Escherichia coli*	59	1	0	3	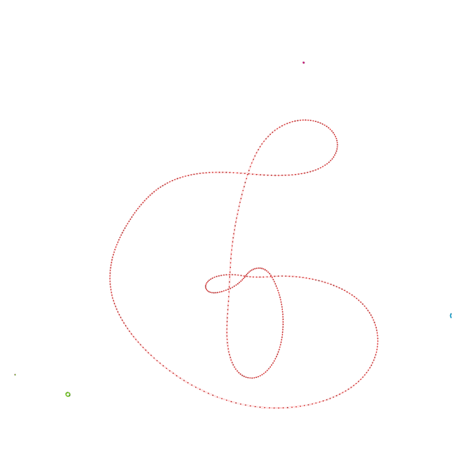	Finished circular assembly	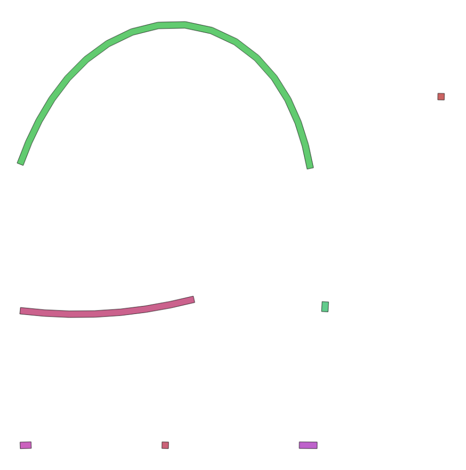	
NCTC9124	*Streptococcus sp.*	133	Pending	Pending	Pending	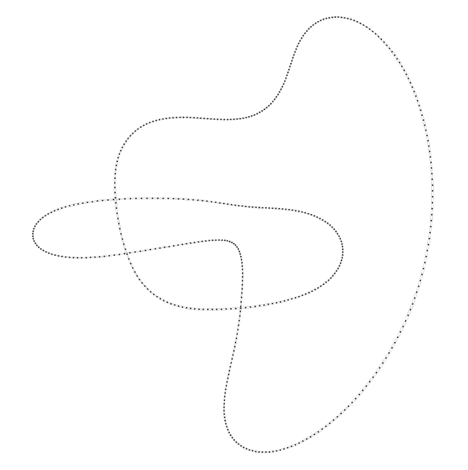	Finished circular assembly	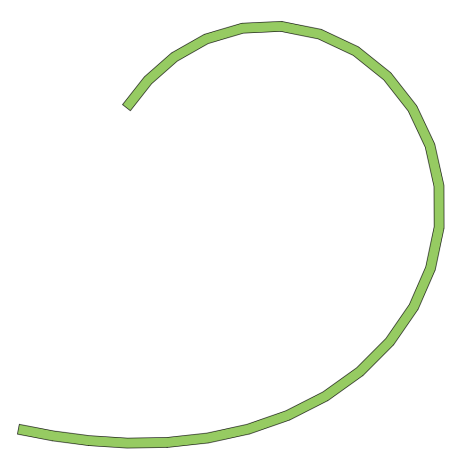	
NCTC9127	*Klebsiella sp.*	43	1	1	0		Finished circular assembly		

						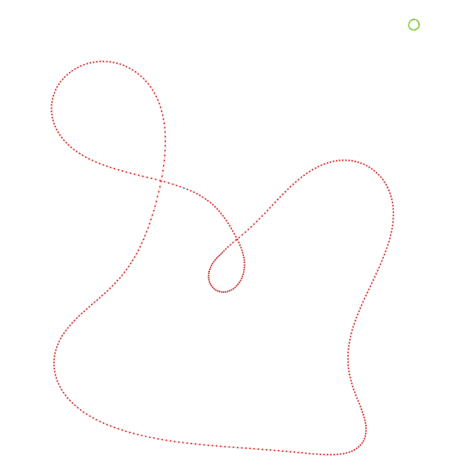		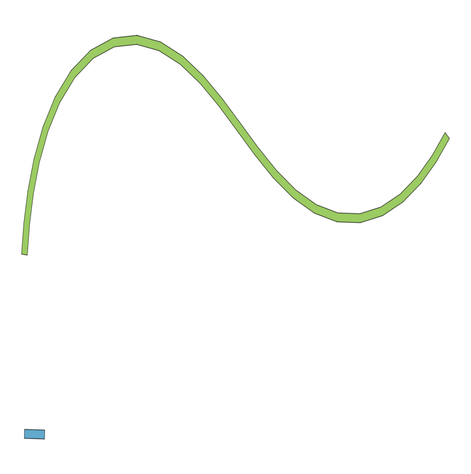
NCTC9128	*Klebsiella pneumoniae*	38	0	0	2	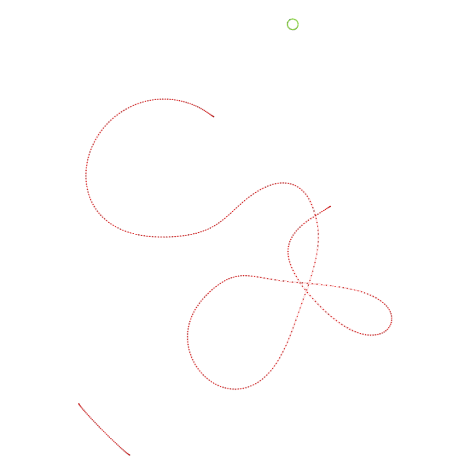	Mis-assembly/Fragmented	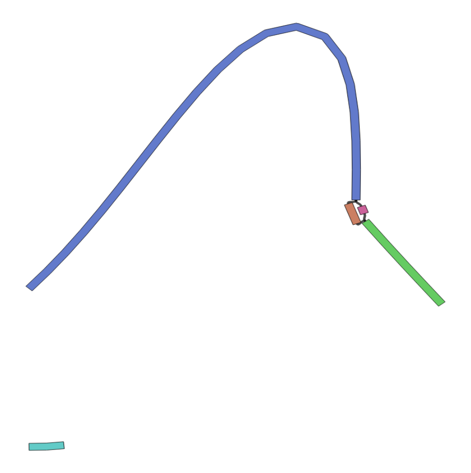
NCTC9129	*Klebsiella sp.*	43	0	0	4	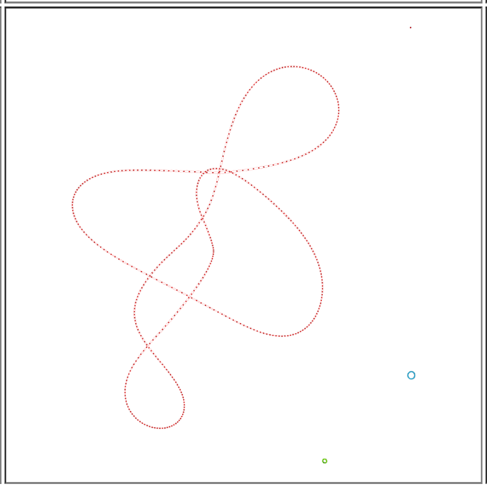	Finished circular assembly	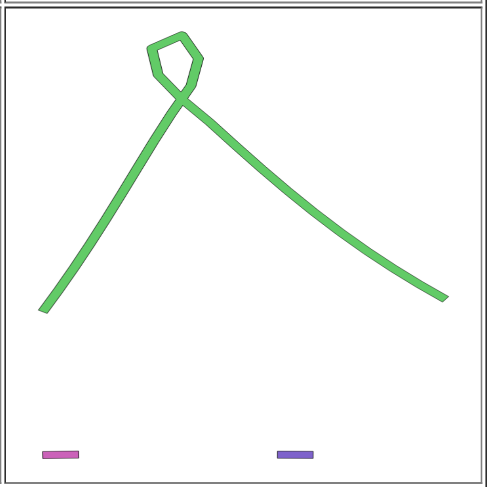
NCTC9130	*Klebsiella sp.*	0	1	1	1	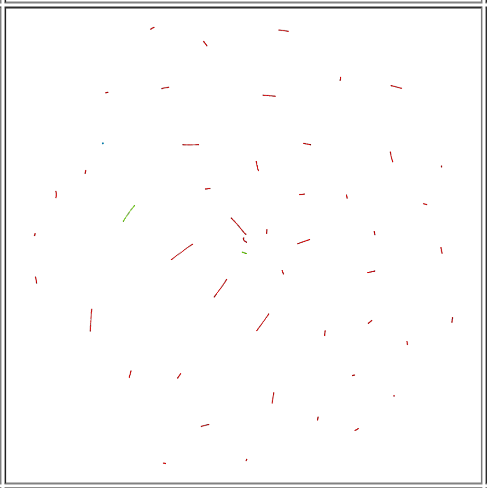	Mis-assembly/Fragmented	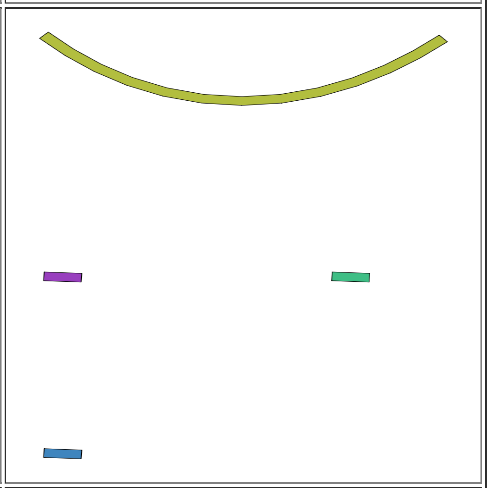
NCTC9132	*Klebsiella sp.*	69	0	0	2	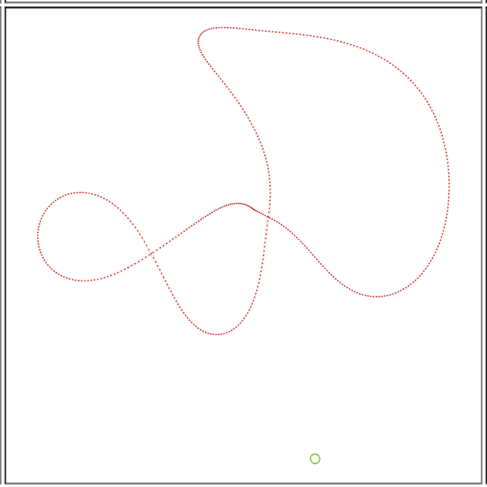	Finished circular assembly	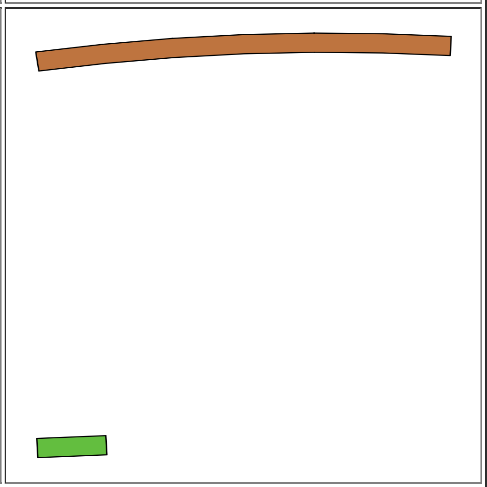
NCTC9133	*Klebsiella sp.*	71	1	0	1	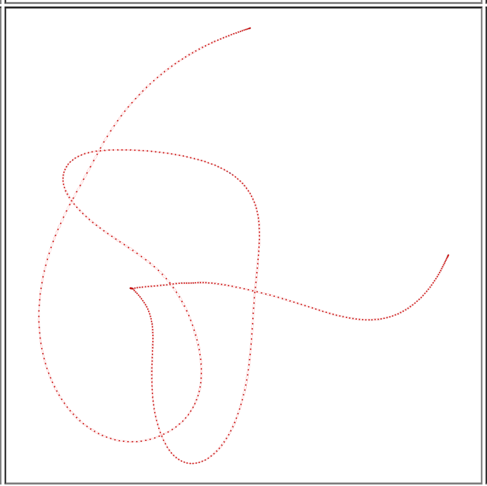	Finished assembly (lacking circularisation)	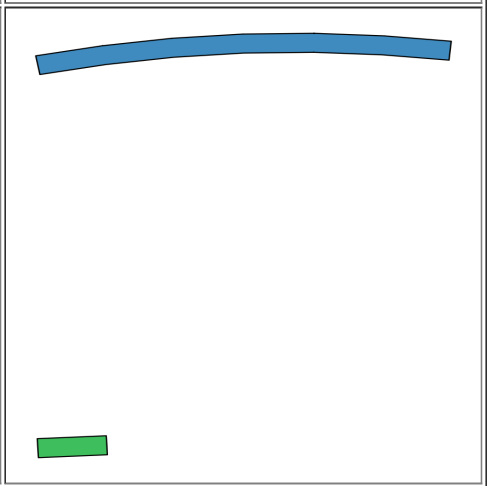
NCTC9134	*Klebsiella pneumoniae*	0	0	0	2		Mis-assembly/Fragmented	

						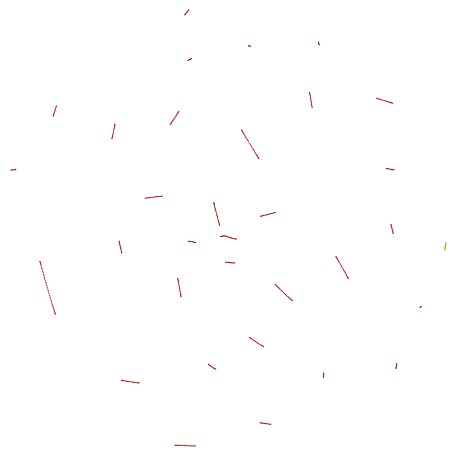		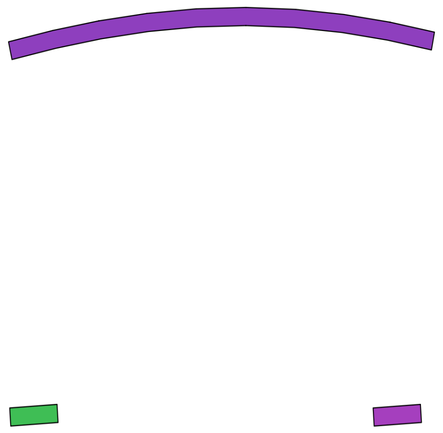
NCTC9135	*Klebsiella sp.*	35	0	0	6	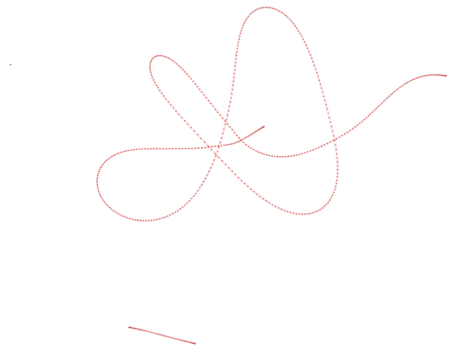	Finished assembly (lacking circularisation)	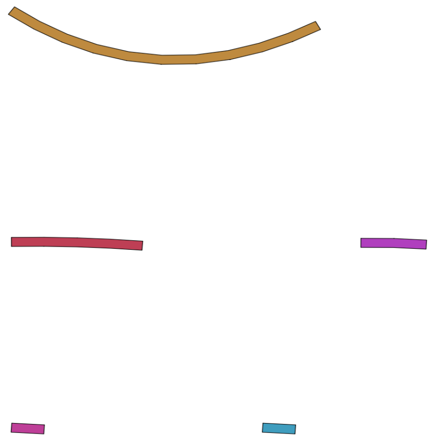
NCTC9136	*Klebsiella sp.*	68	1	1	0	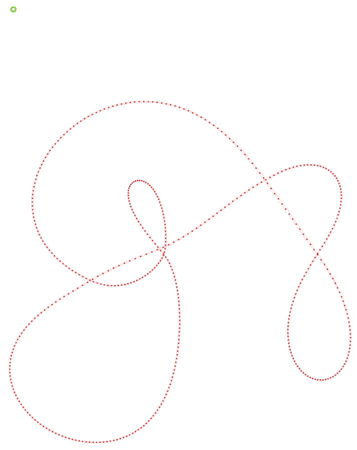	Finished circular assembly	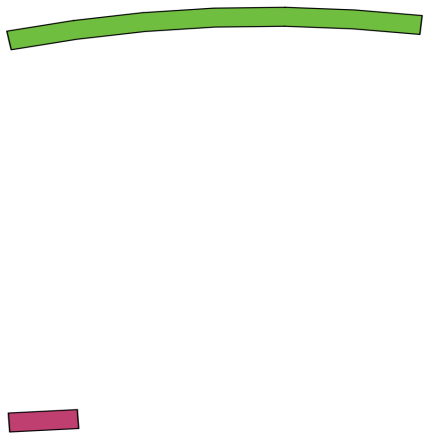
NCTC9137	*Klebsiella sp.*	64	1	3	0	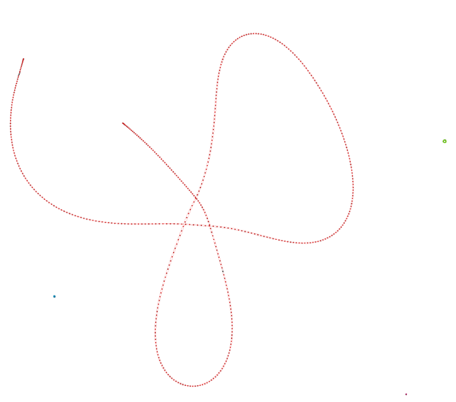	Finished assembly (lacking circularisation)	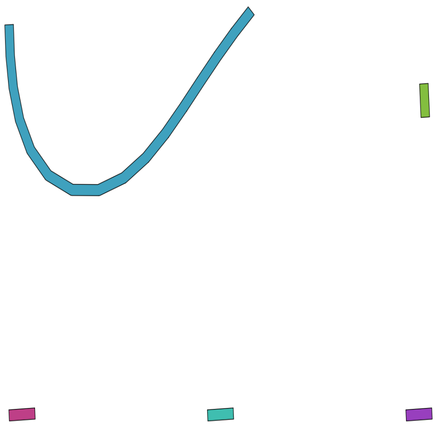
NCTC9138	*Klebsiella sp.*	52	1	1	0	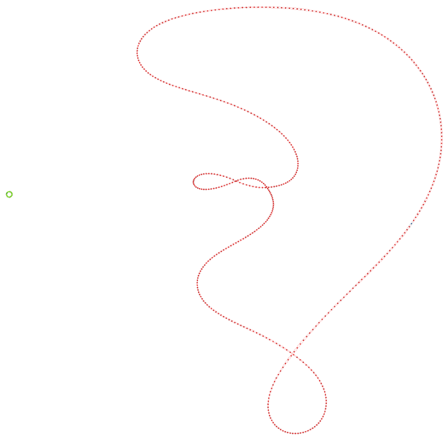	Finished circular assembly	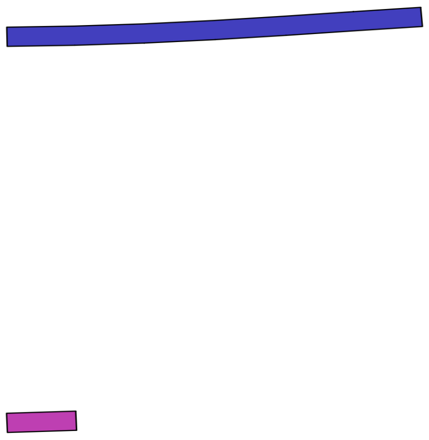
NCTC9139	*Klebsiella sp.*	37	1	1	0	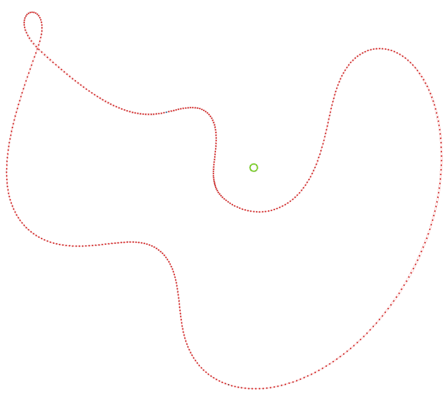	Finished circular assembly	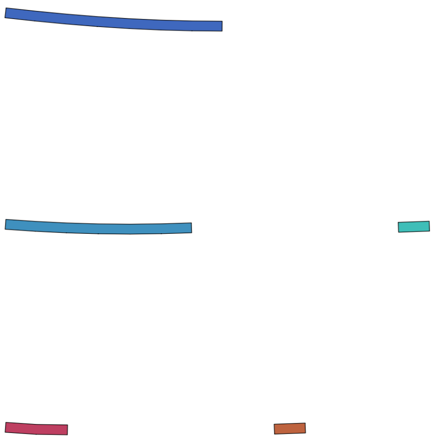
NCTC9140	*Klebsiella pneumoniae*	59	1	3	3		Finished circular assembly	

						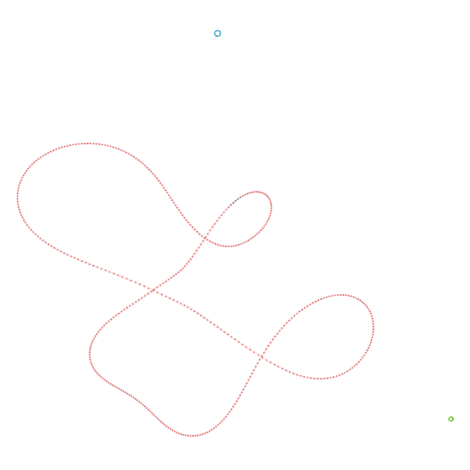		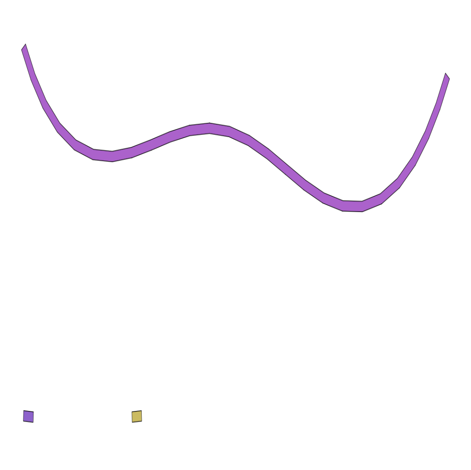
NCTC9142	*Klebsiella sp.*	70	1	1	0	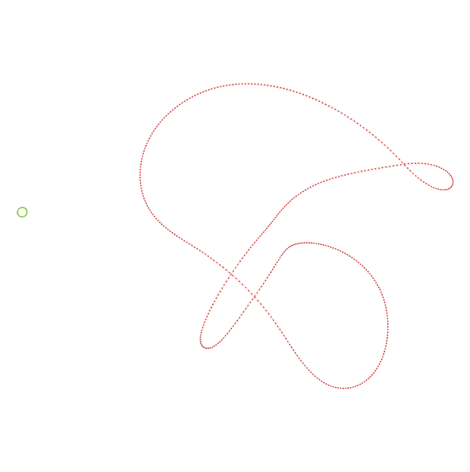	Finished circular assembly	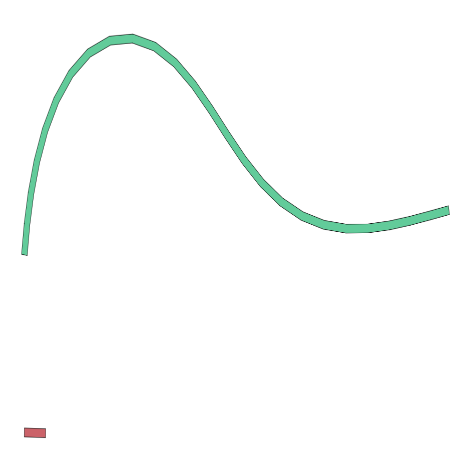
NCTC9143	*Klebsiella pneumoniae*	24	1	0	4	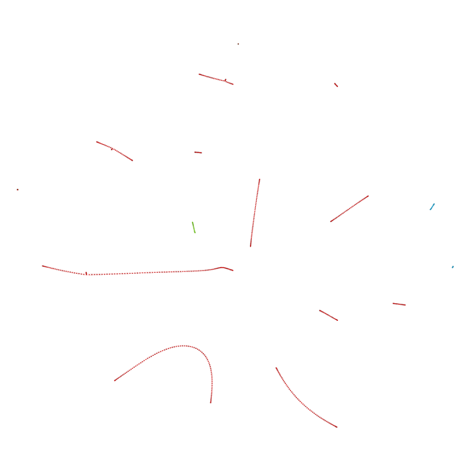	Mis-assembly/Fragmented	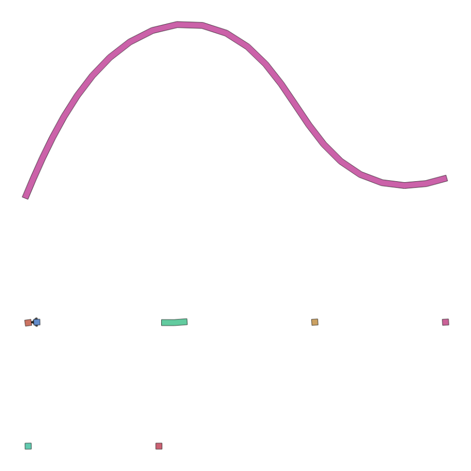
NCTC9144	*Klebsiella sp.*	56	1	1	1	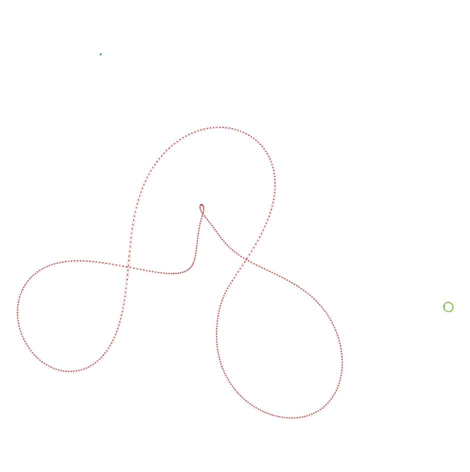	Finished circular assembly	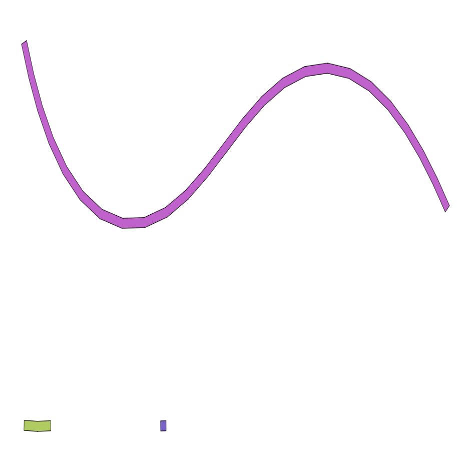
NCTC9145	*Klebsiella sp.*	42	1	1	2	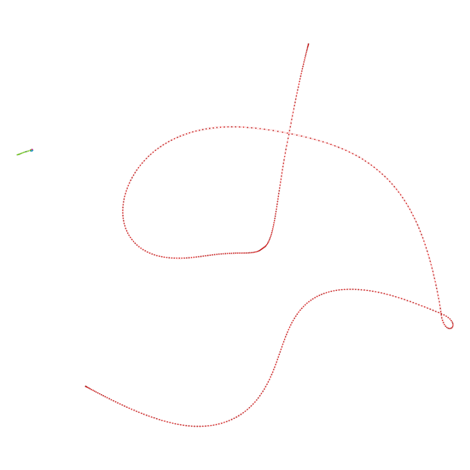	Finished assembly (lacking circularisation)	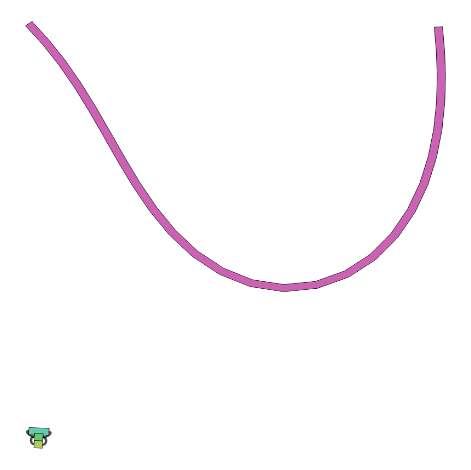
NCTC9146	*Klebsiella sp.*	64	1	0	0	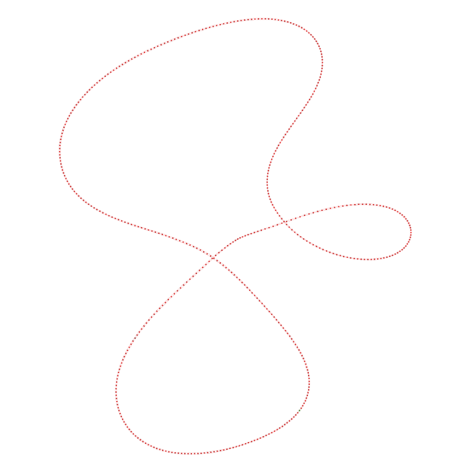	Finished circular assembly	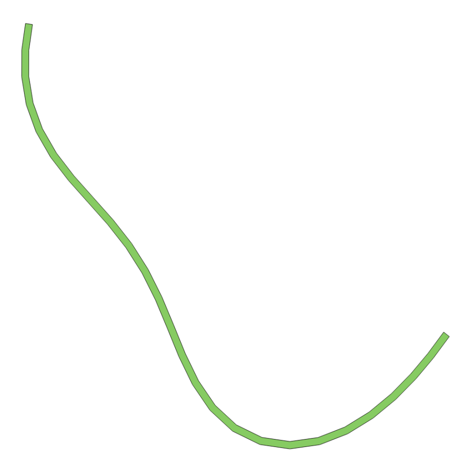
NCTC9149	*Klebsiella sp.*	33	1	0	1		Mis-assembly/Fragmented	

						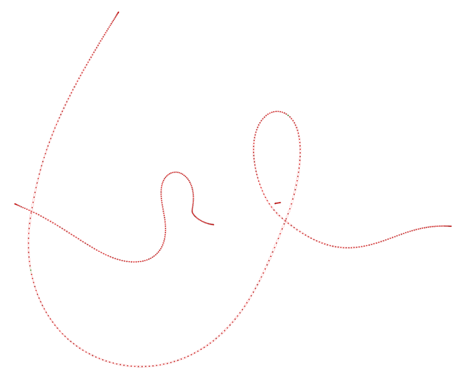		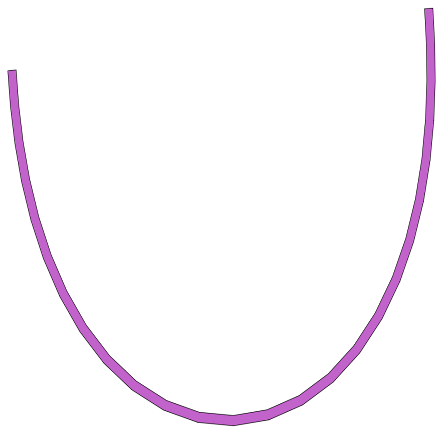
NCTC9150	*Klebsiella sp.*	59	1	1	0	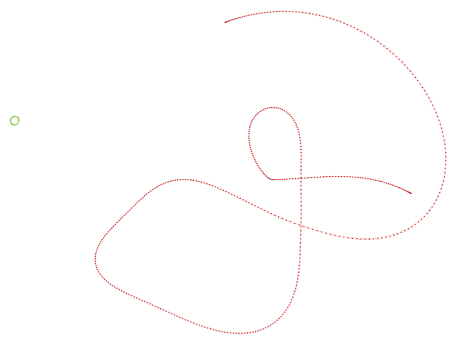	Finished circular assembly	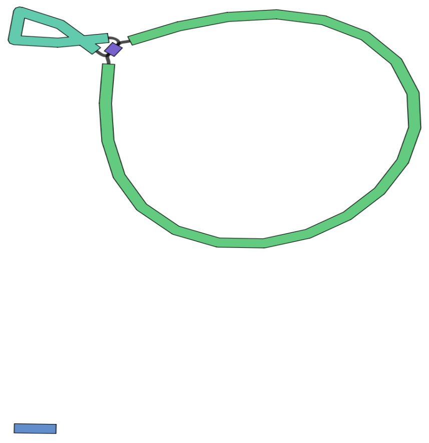
NCTC9151	*Klebsiella sp.*	45	1	0	4	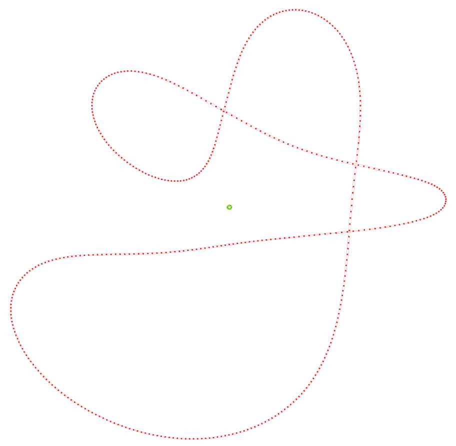	Finished circular assembly	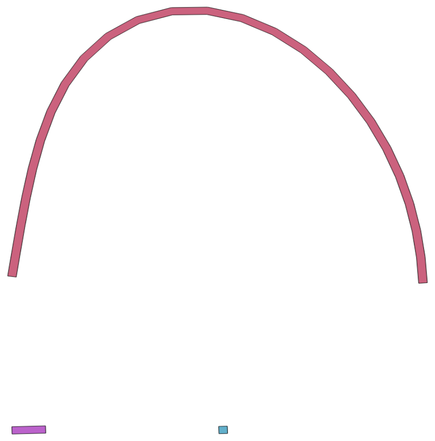
NCTC9152	*Klebsiella sp.*	40	1	0	1	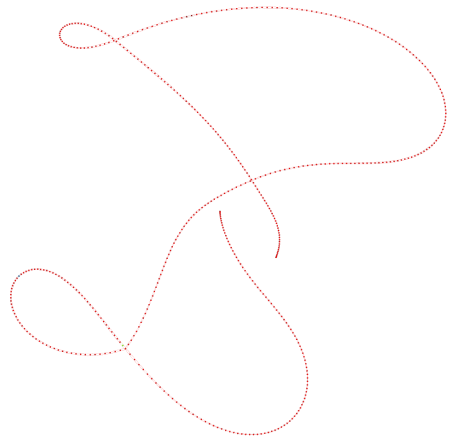	Possible mis-assembly	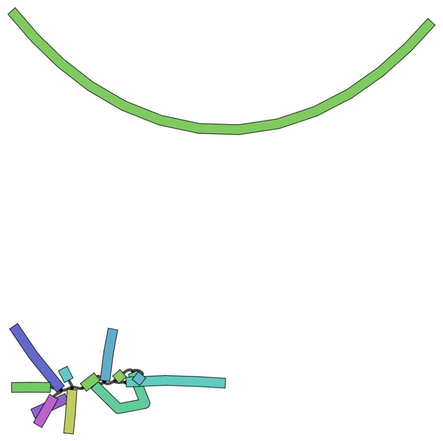
NCTC9153	*Klebsiella sp.*	47	0	0	3	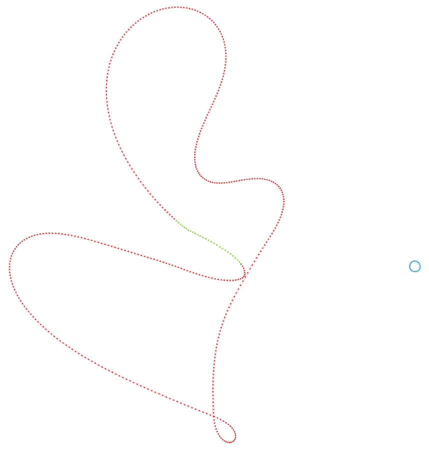	Finished circular assembly	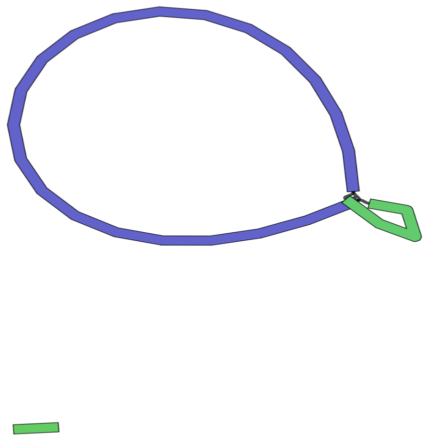
NCTC9154	*Klebsiella sp.*	32	1	1	0	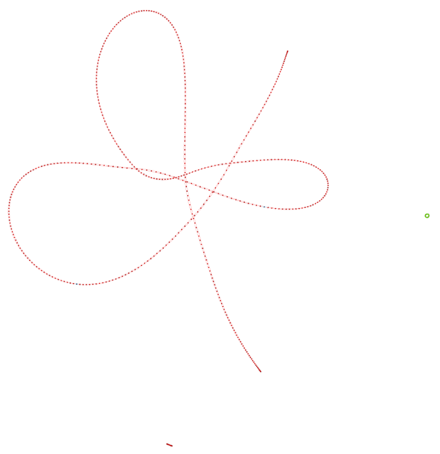	Finished assembly (lacking circularisation)	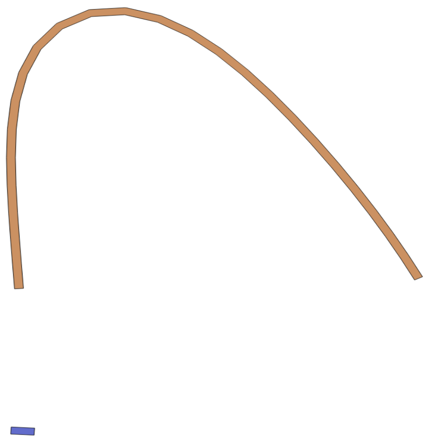
NCTC9155	*Klebsiella sp.*	39	0	0	3		Finished circular assembly	

						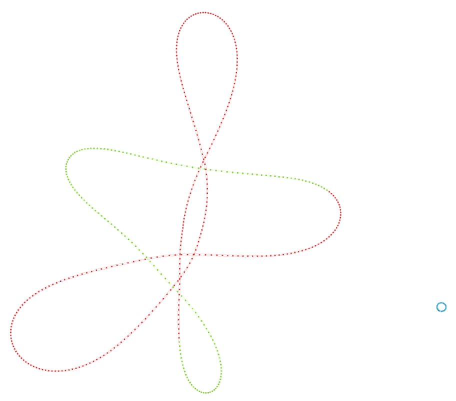		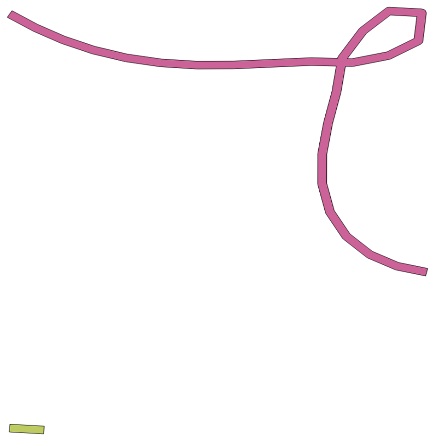
NCTC9156	*Klebsiella sp.*	51	1	1	0	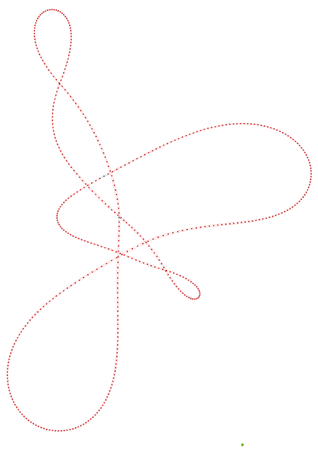	Finished circular assembly	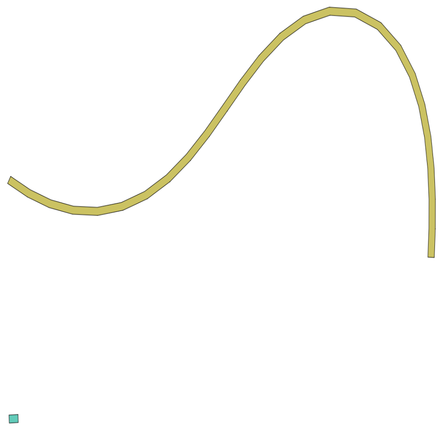
NCTC9157	*Klebsiella sp.*	68	1	0	0	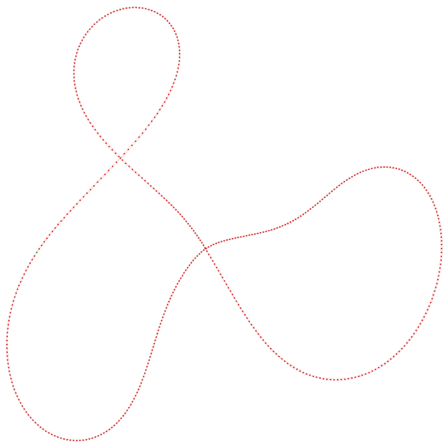	Finished circular assembly	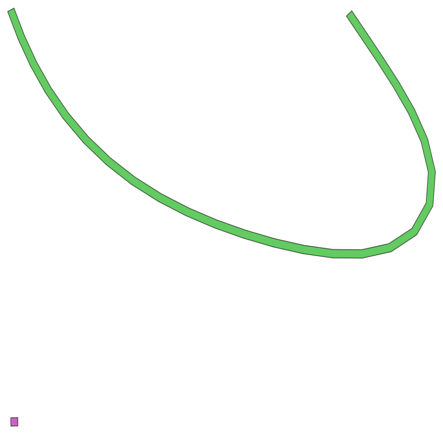
NCTC9158	*Klebsiella sp.*	44	1	1	1	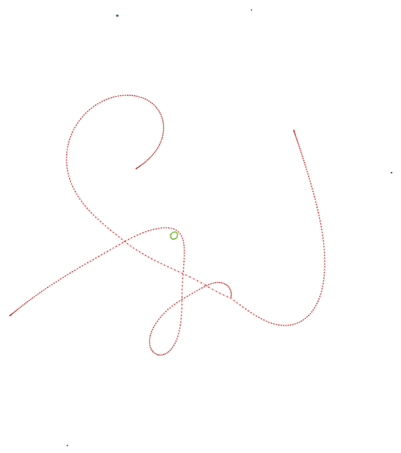	Mis-assembly/Fragmented	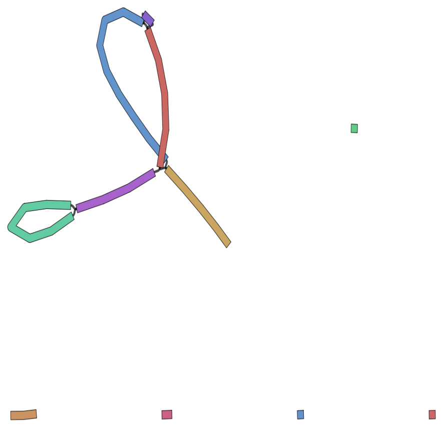
NCTC9159	*Klebsiella sp.*	46	0	0	4	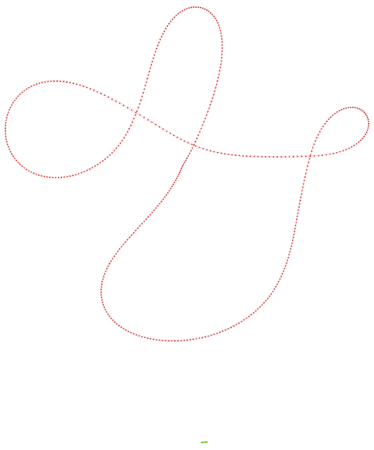	Finished circular assembly	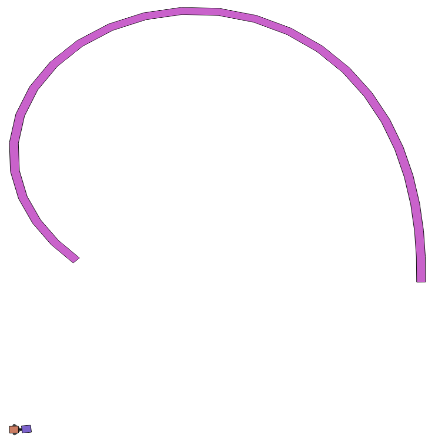
NCTC9161	*Klebsiella sp.*	41	1	1	0	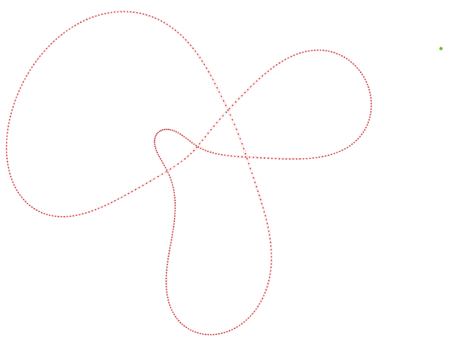	Finished circular assembly	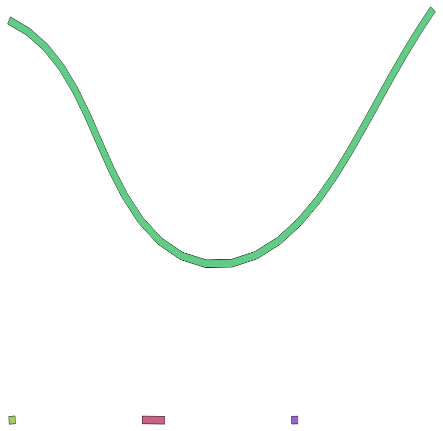
NCTC9162	*Klebsiella sp.*	64	1	0	6		Finished assembly (lacking circularisation)	

						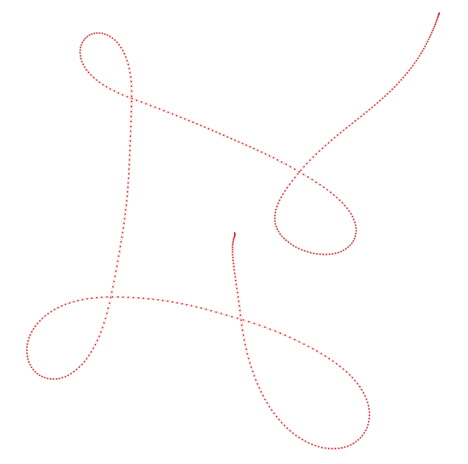		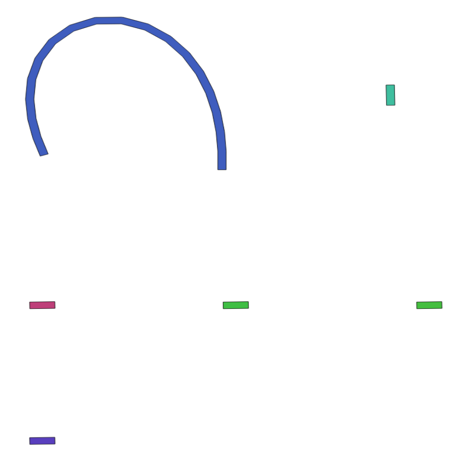
NCTC9163	*Klebsiella sp.*	0	1	2	1	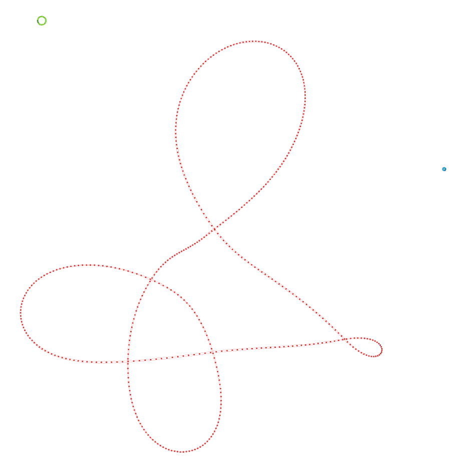	Finished circular assembly	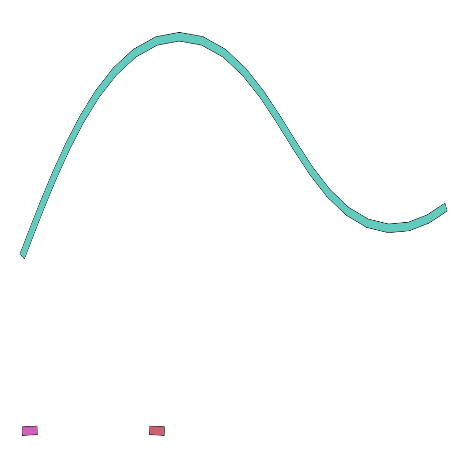
NCTC9164	*Klebsiella sp.*	75	1	1	0	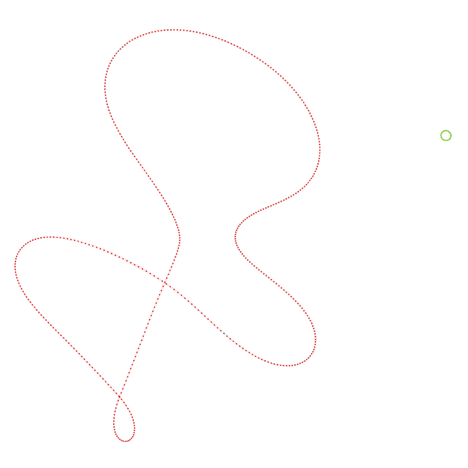	Finished circular assembly	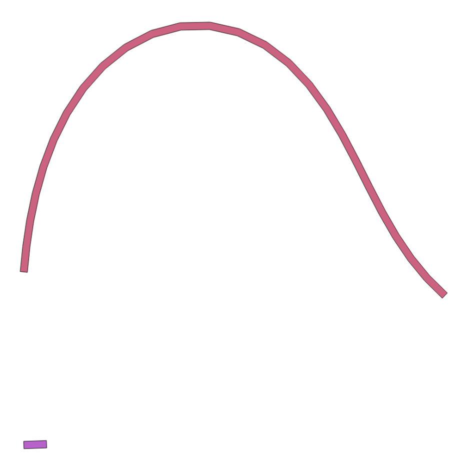
NCTC9166	*Klebsiella sp.*	61	1	0	1	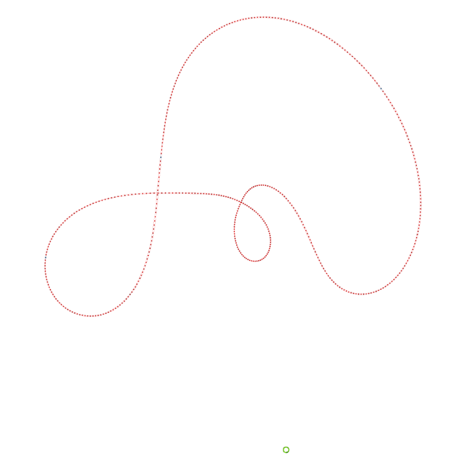	Finished circular assembly	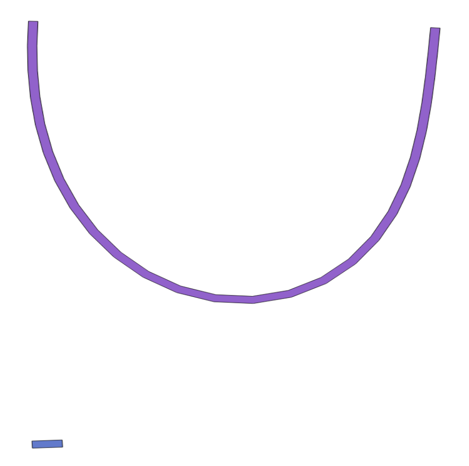
NCTC9167	*Klebsiella sp.*	56	1	0	1	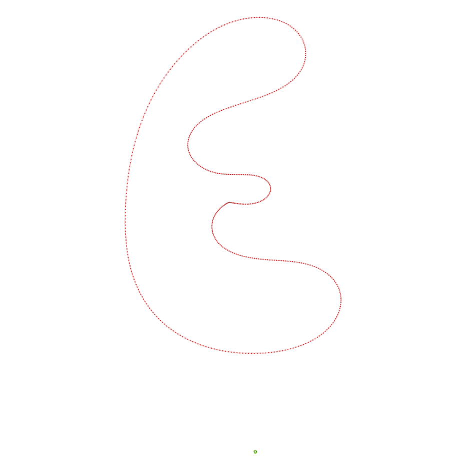	Finished circular assembly	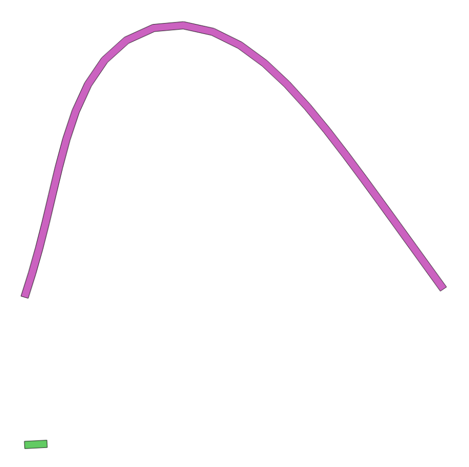
NCTC9169	*Klebsiella sp.*	74	1	0	1	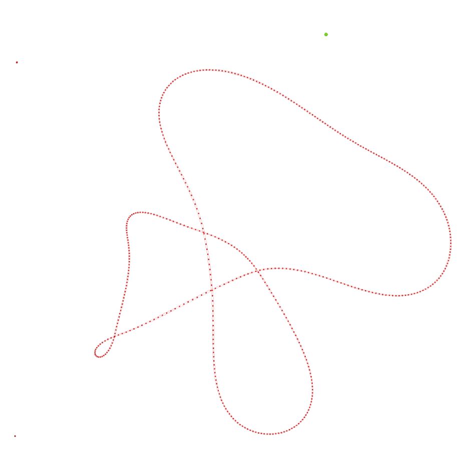	Finished circular assembly	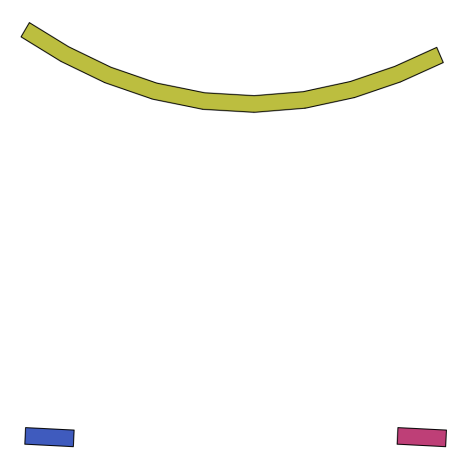
NCTC9170	*Klebsiella sp.*	85	0	0	1		Finished circular assembly	

						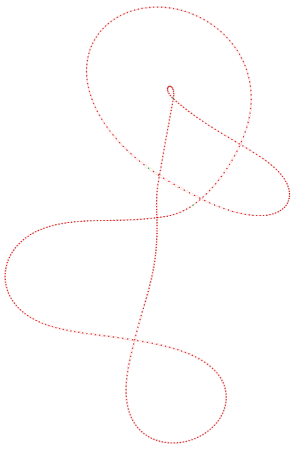		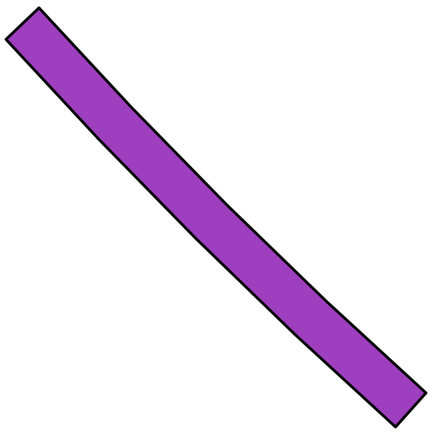
NCTC9171	*Klebsiella sp.*	117	1	0	0	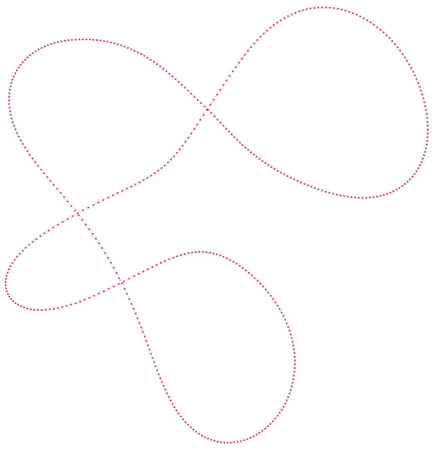	Finished circular assembly	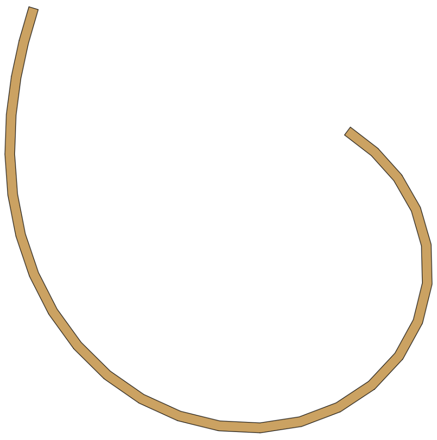
NCTC9174	*Klebsiella sp.*	9	0	0	4	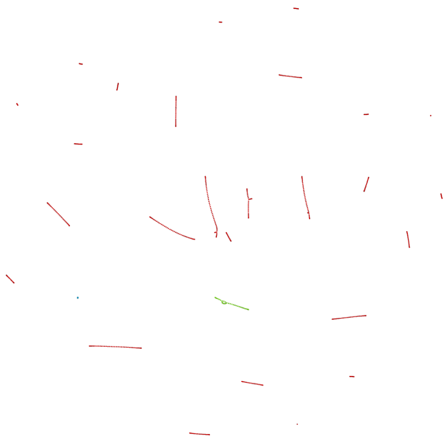	Mis-assembly/Fragmented	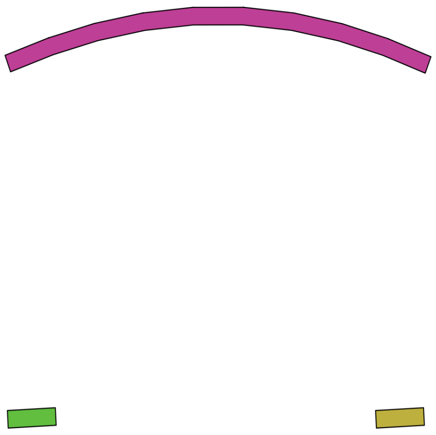
NCTC9175	*Klebsiella sp.*	31	1	3	0	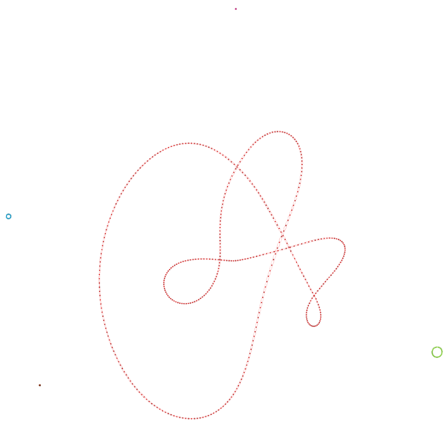	Finished circular assembly	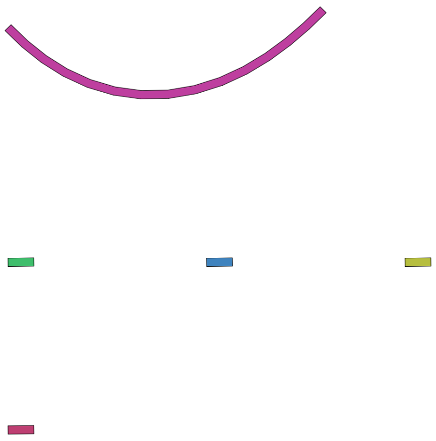
NCTC9176	*Klebsiella sp.*	42	1	0	1	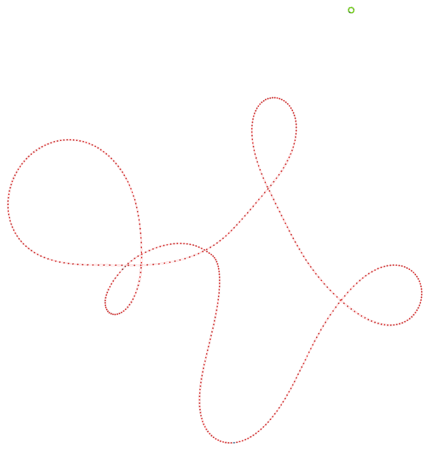	Finished circular assembly	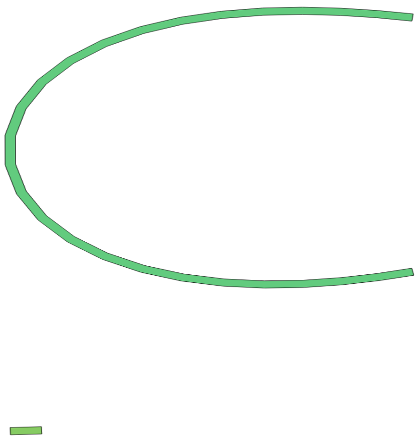
NCTC9177	*Klebsiella pneumoniae*	65	0	0	4	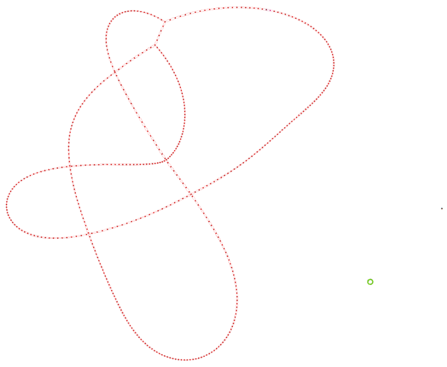	Mis-assembly/Fragmented	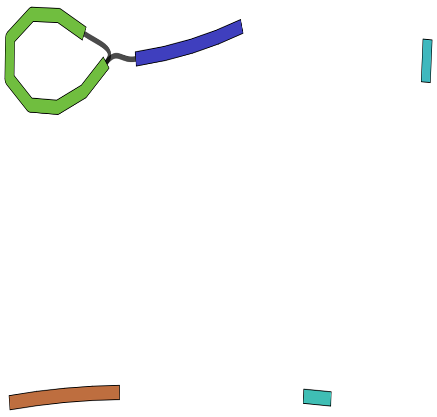
NCTC9178	*Klebsiella sp.*	29	0	0	1		Mis-assembly/Fragmented	

						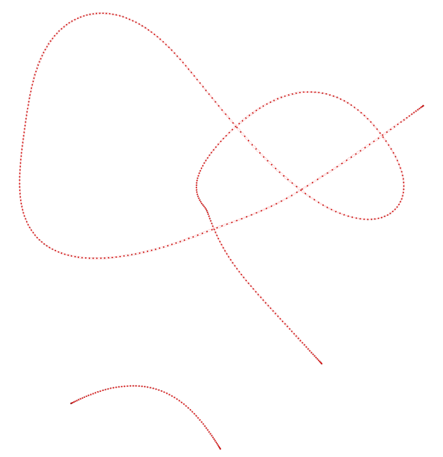		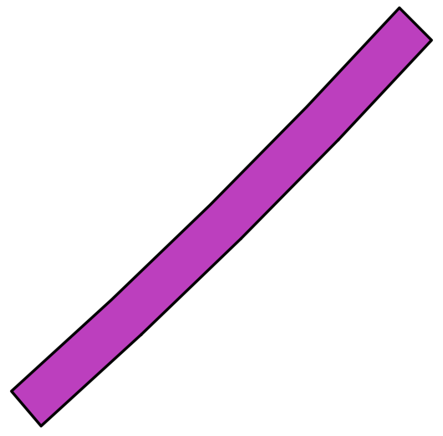
NCTC9179	*Klebsiella sp.*	72	1	2	0	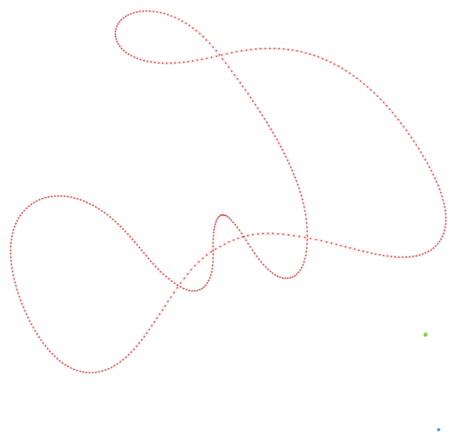	Finished circular assembly	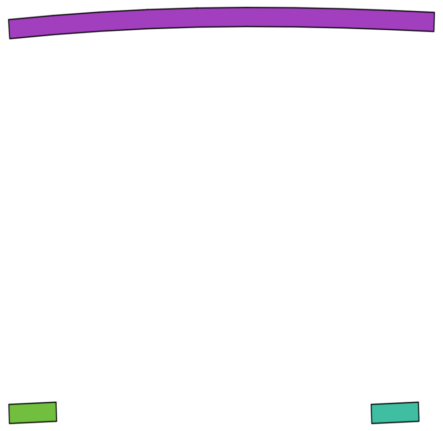
NCTC9180	*Klebsiella sp.*	0	0	0	1	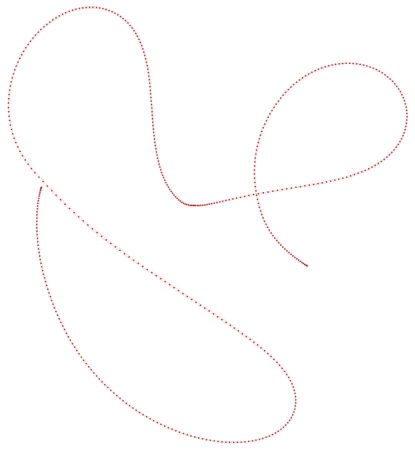	Finished assembly (lacking circularisation)	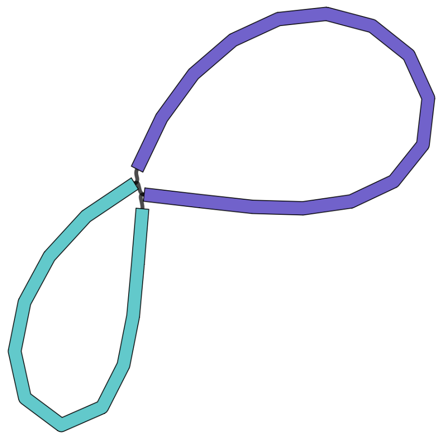
NCTC9181	*Klebsiella sp.*	53	0	0	3	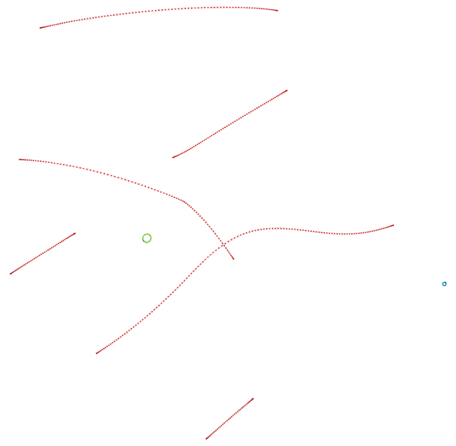	Mis-assembly/Fragmented	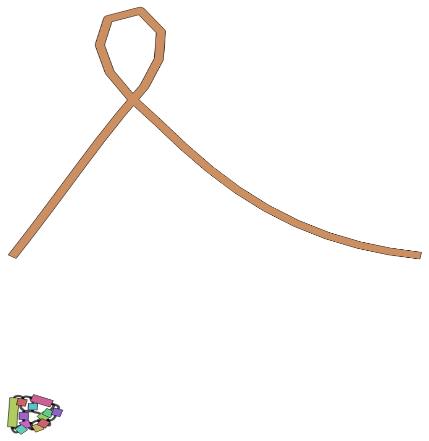
NCTC9182	*Klebsiella sp.*	72	1	1	0	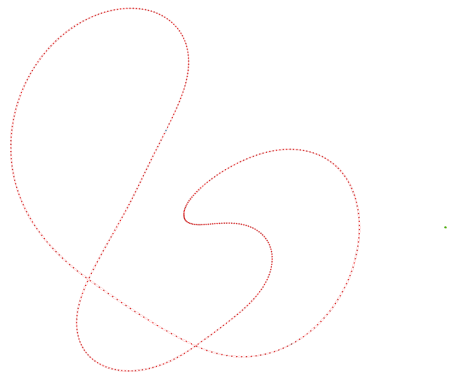	Finished circular assembly	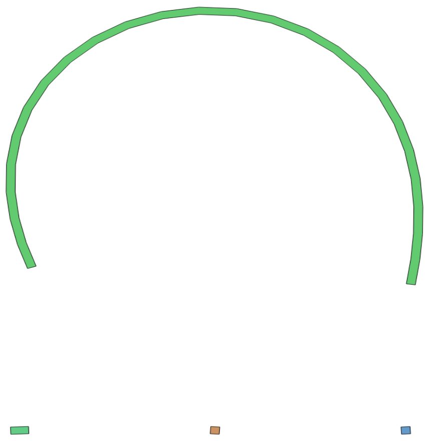
NCTC9183	*Klebsiella sp.*	24	0	0	3	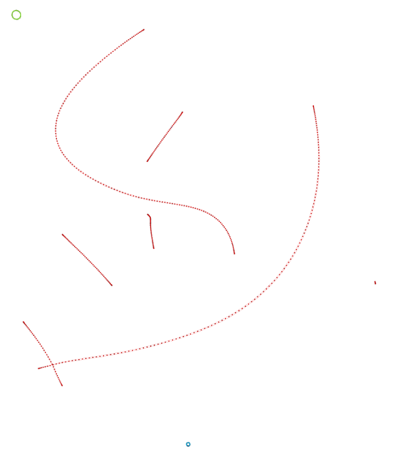	Mis-assembly/Fragmented	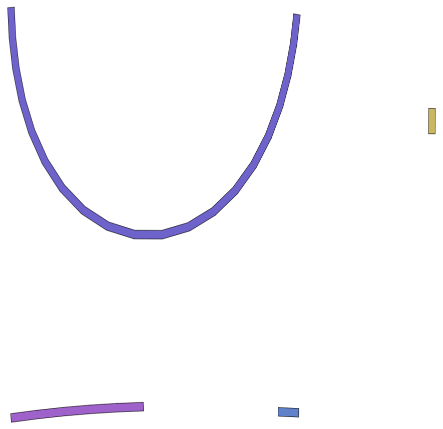
NCTC9184	*Klebsiella sp.*	6	0	0	179		Mis-assembly/Fragmented	

								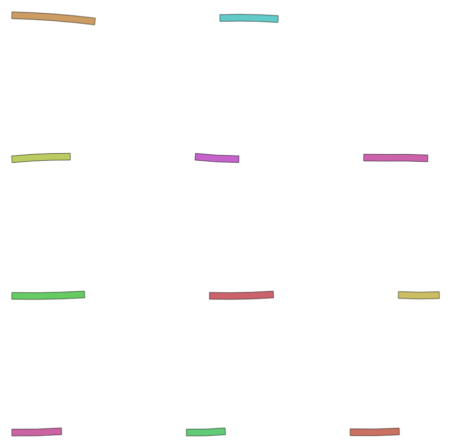
NCTC9185	*Klebsiella sp.*	24	0	0	2	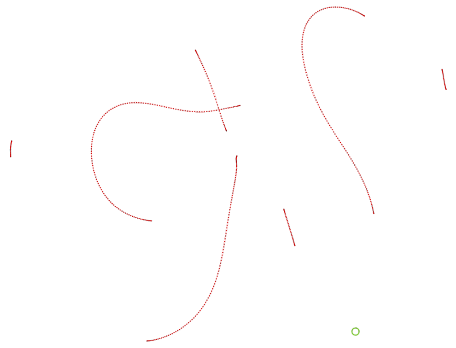	Mis-assembly/Fragmented	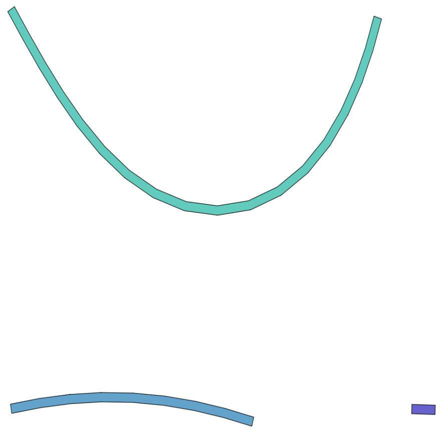
NCTC9186	*Klebsiella sp.*	37	1	2	1	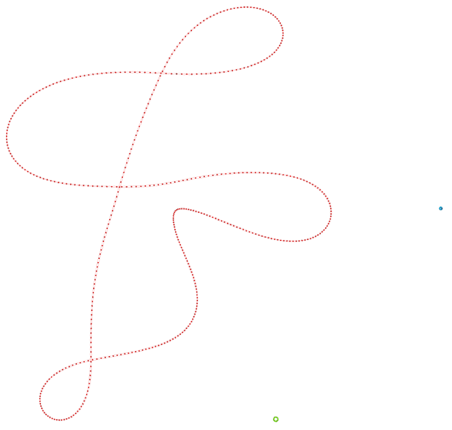	Finished circular assembly	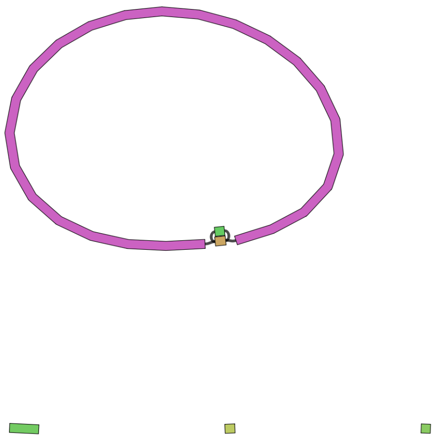
NCTC9187	*Klebsiella sp.*	24	Pending	Pending	Pending	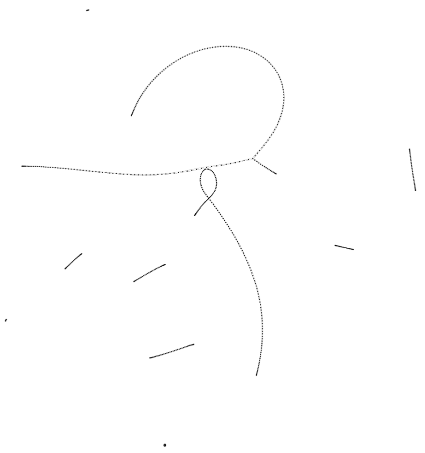	Mis-assembly/Fragmented	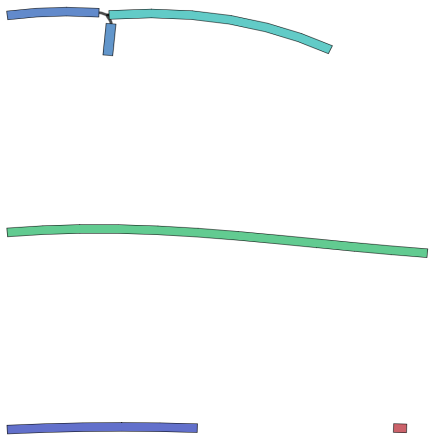
NCTC9188	*Klebsiella sp.*	63	0	0	2	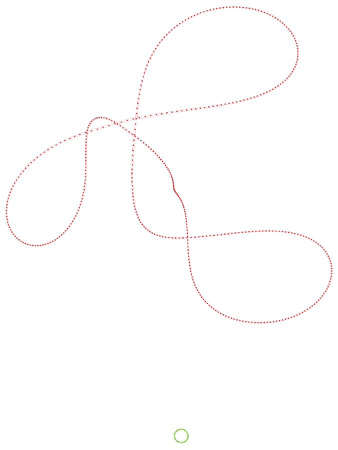	Finished circular assembly	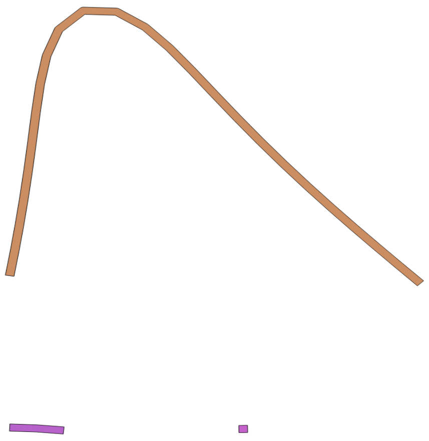
NCTC9189	*Klebsiella sp.*	70	1	0	0	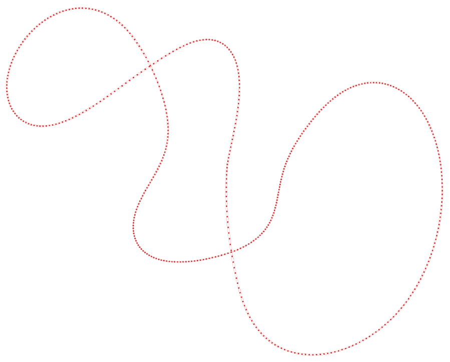	Finished circular assembly	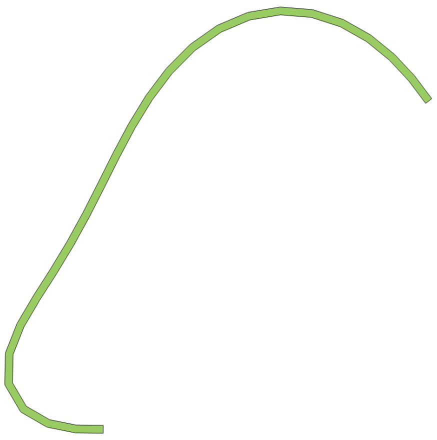
NCTC9239	*Vibrio*	38	Pending	Pending	Pending		Finished circular assembly	

						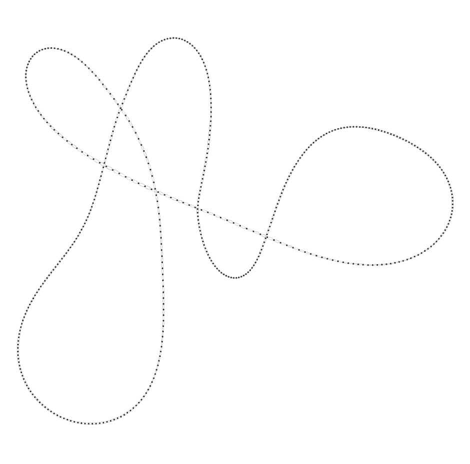		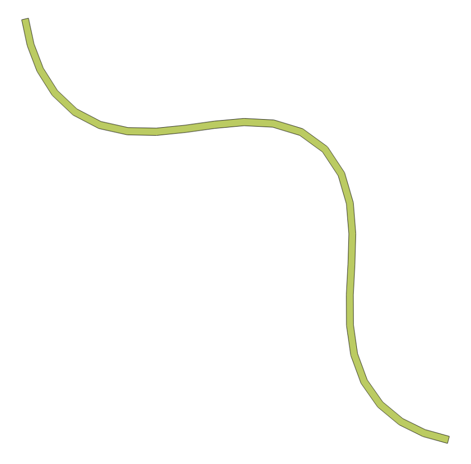
NCTC9309	*Staphylococcus aureus*	50	1	0	2	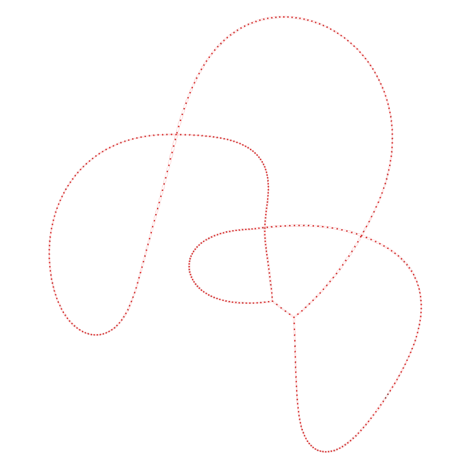	Mis-assembly/Fragmented	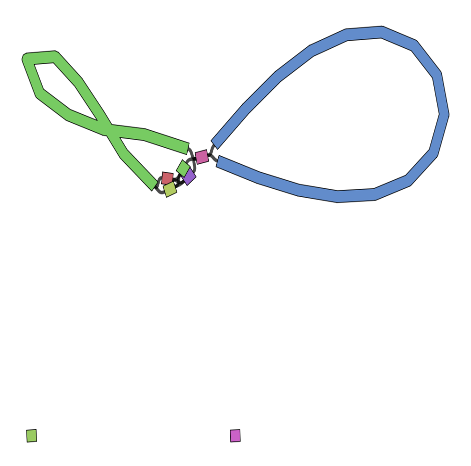
NCTC9369	*Staphylococcus aureus*	119	1	1	0	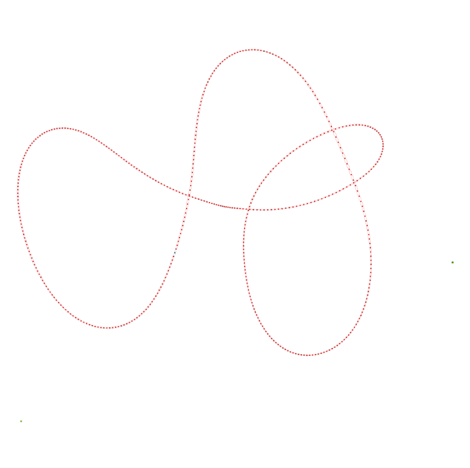	Finished circular assembly	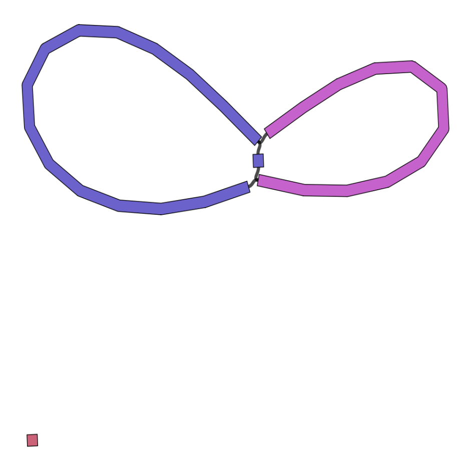
NCTC9381	*Pantoea agglomerans*	41	1	2	0	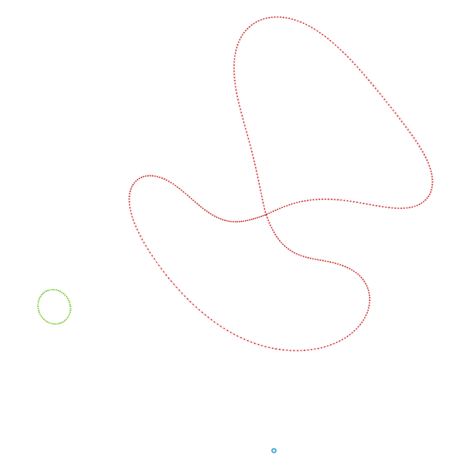	Finished circular assembly	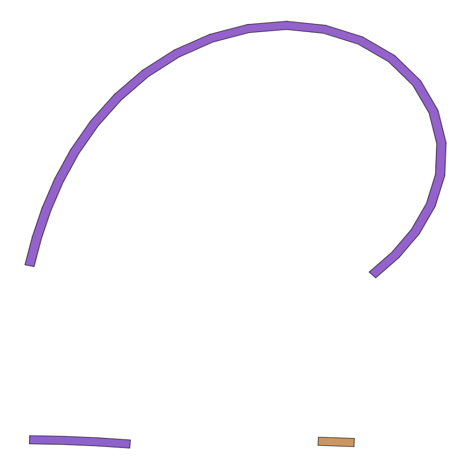
NCTC9393	*Staphylococcus aureus*	78	1	1	0	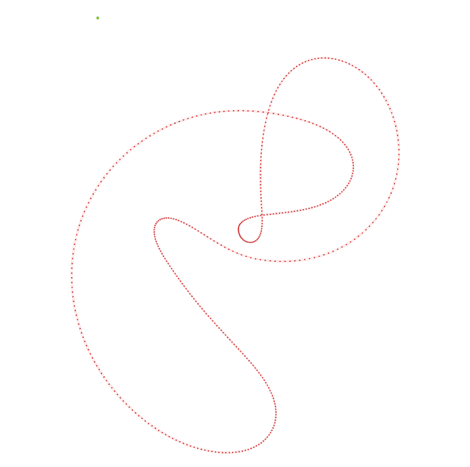	Finished circular assembly	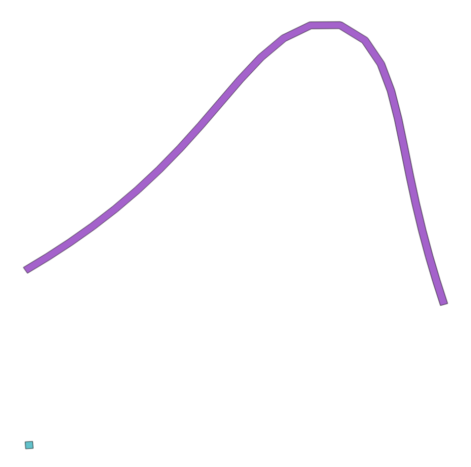
NCTC9413	*Streptococcus sp.*	104	Pending	Pending	Pending	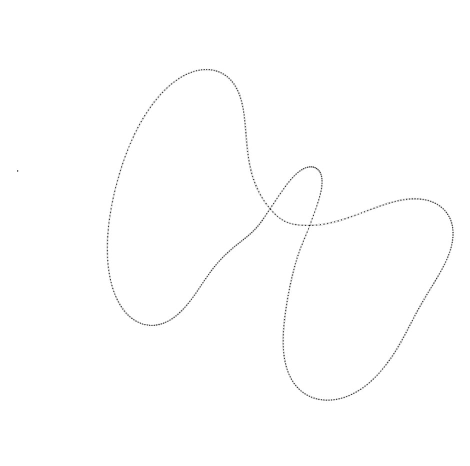	Finished circular assembly	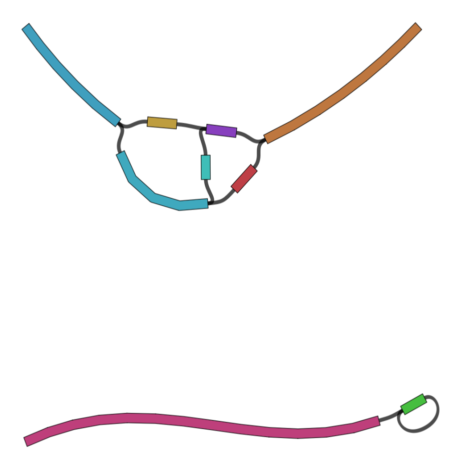
NCTC9414	*Streptococcus sp.*	51	Pending	Pending	Pending		Mis-assembly/Fragmented	

						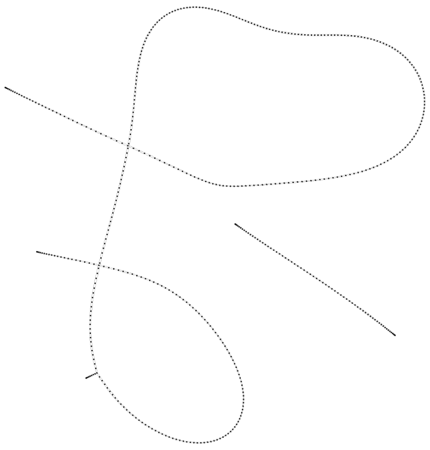		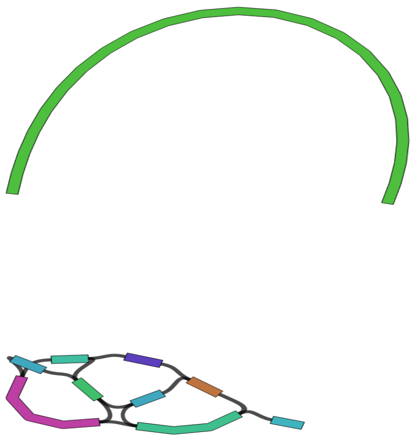
NCTC9419	*Serratia rubidaea*	47	0	0	1	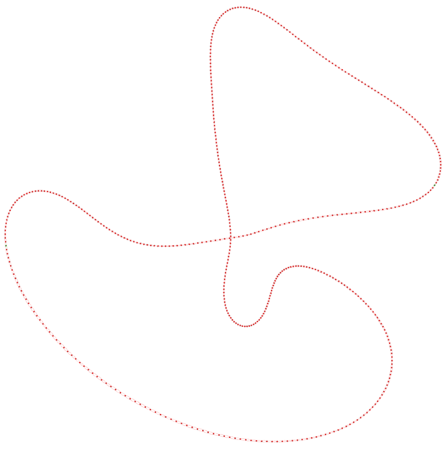	Finished circular assembly	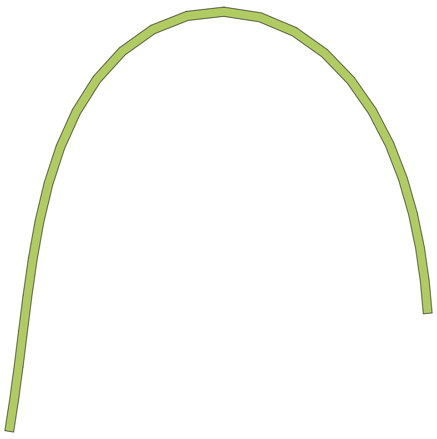
NCTC9433	*Pseudomonas aeruginosa*	53	1	0	0	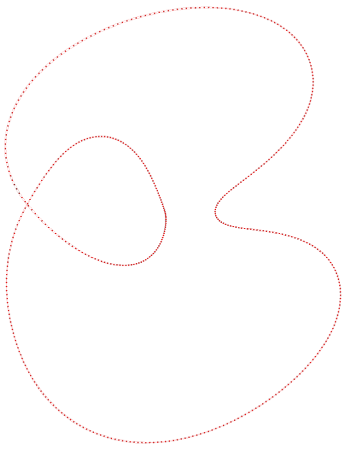	Finished circular assembly	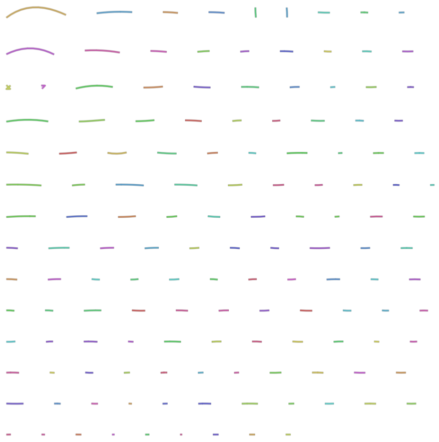
NCTC9434	*Escherichia coli*	83	1	1	0	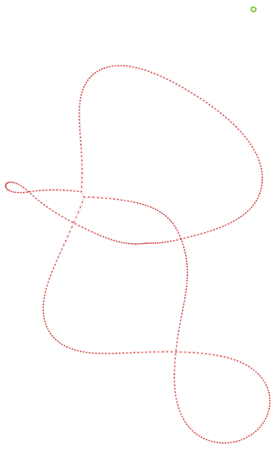	Finished assembly with multiple traversals	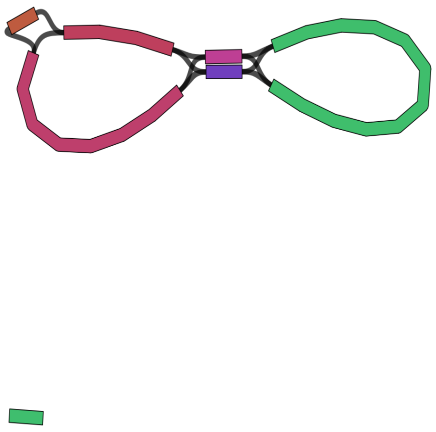
NCTC9494	*Klebsiella pneumoniae subsp. pneumoniae*	60	1	1	1	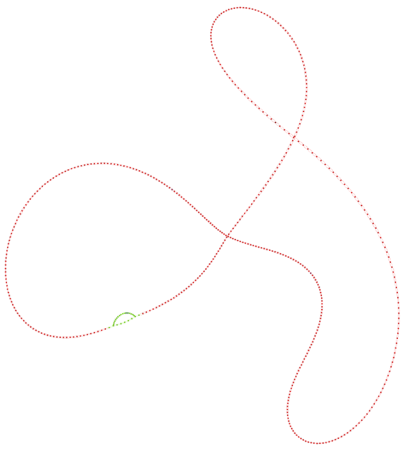	Finished circular assembly	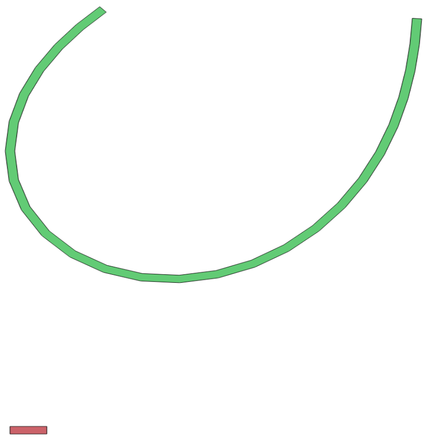
NCTC9495	*Klebsiella sp.*	75	1	1	2	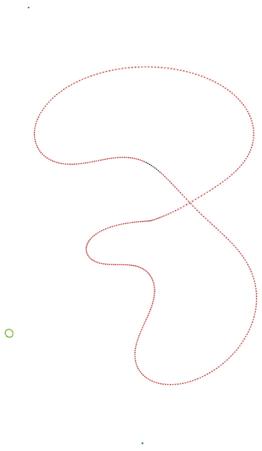	Finished circular assembly	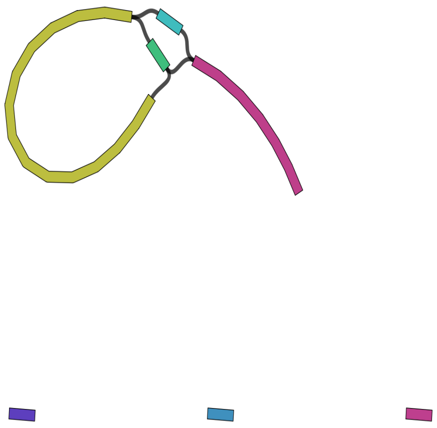
NCTC9498	*Klebsiella sp.*	51	0	0	4		Finished circular assembly	

						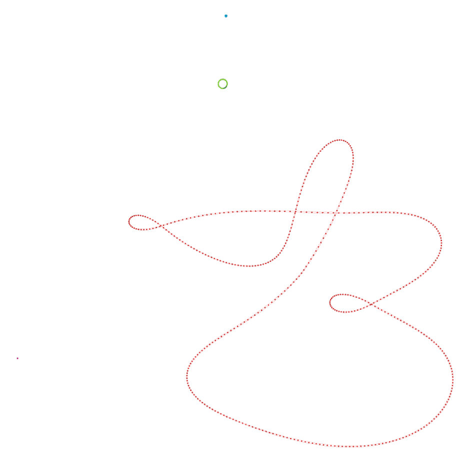		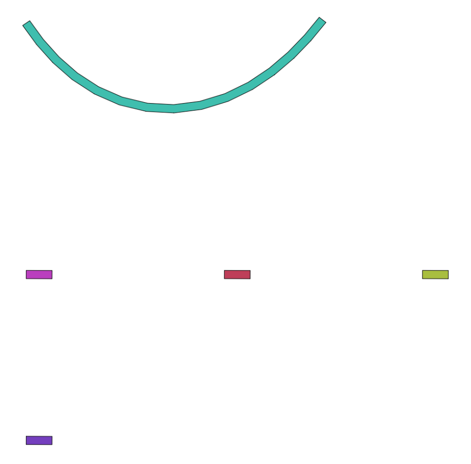
NCTC9504	*Klebsiella pneumoniae subsp. pneumoniae*	45	1	1	3	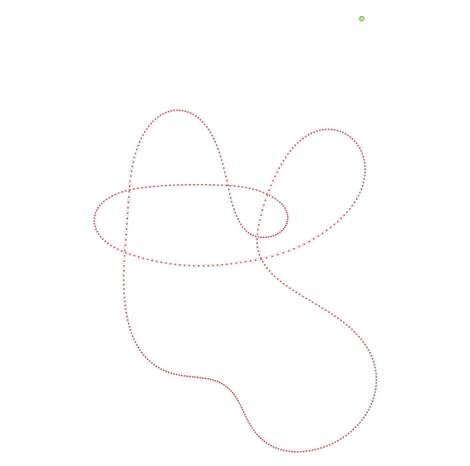	Finished circular assembly	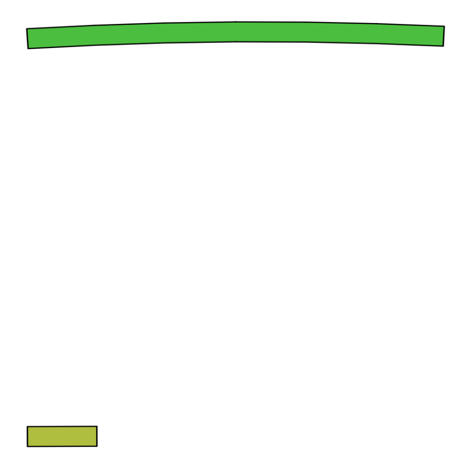
NCTC9527	*Klebsiella sp.*	85	1	0	3	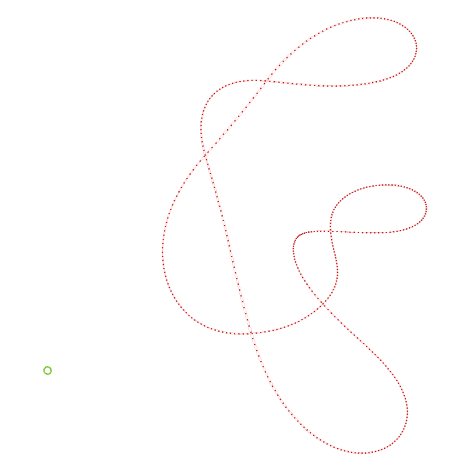	Finished circular assembly	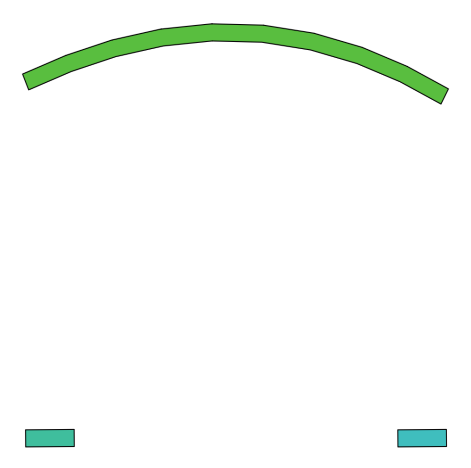
NCTC9529	*Cronobacter universalis*	88	1	1	0	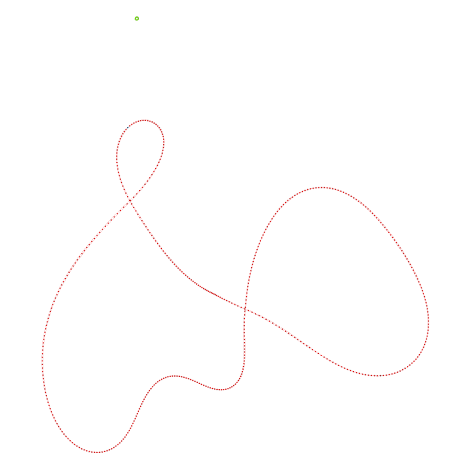	Finished circular assembly	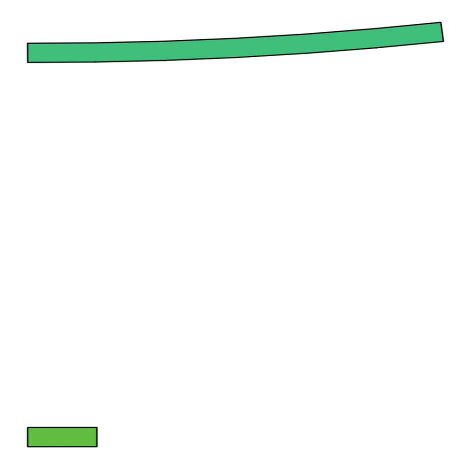
NCTC9546	*Staphylococcus aureus*	83	1	0	2	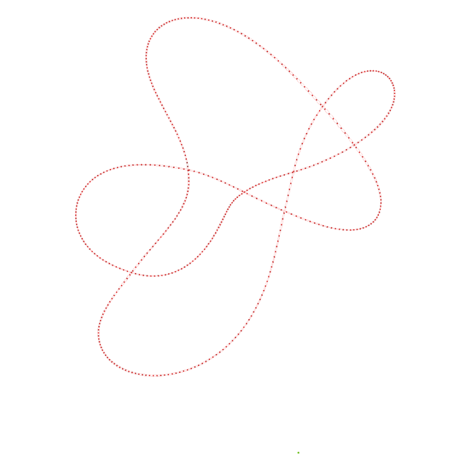	Finished circular assembly	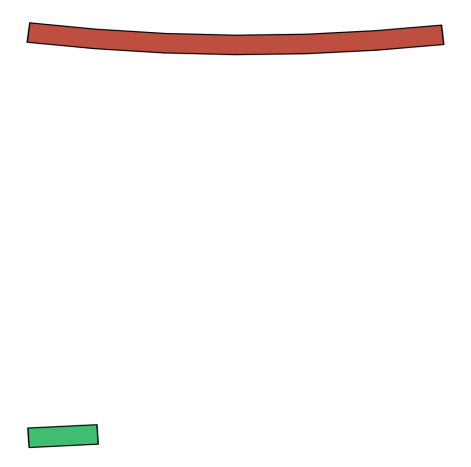
NCTC9547	*Staphylococcus aureus*	165	1	0	1	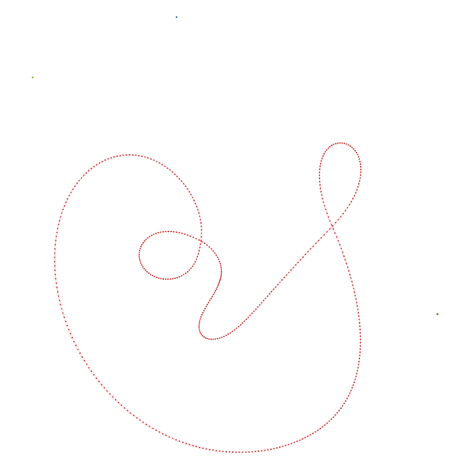	Finished circular assembly	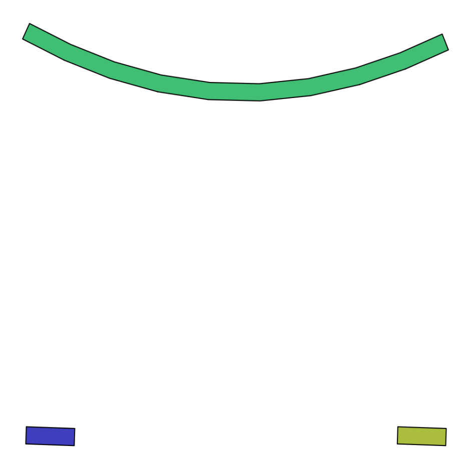
NCTC9551	*Staphylococcus aureus*	45	1	0	2		Finished circular assembly	

						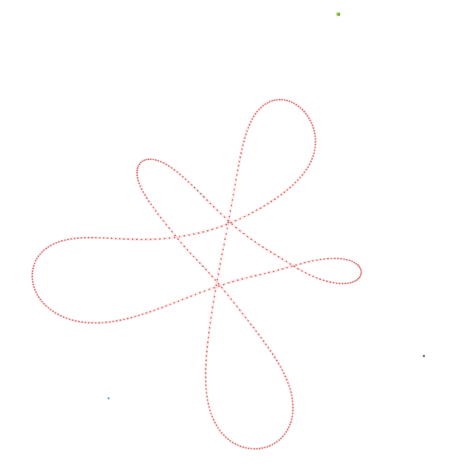		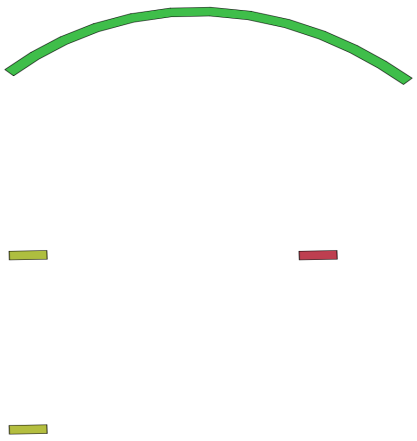
NCTC9552	*Staphylococcus aureus*	27	Pending	Pending	Pending	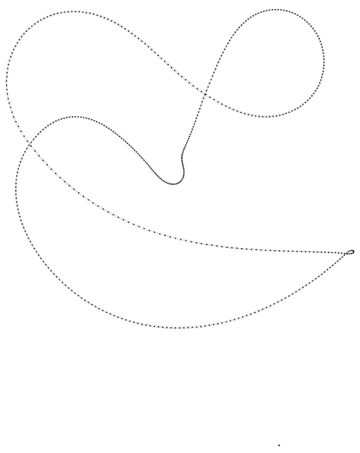	Finished circular assembly	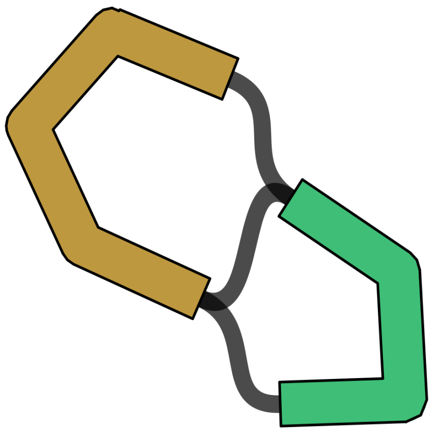
NCTC9553	*Staphylococcus aureus*	159	1	1	0	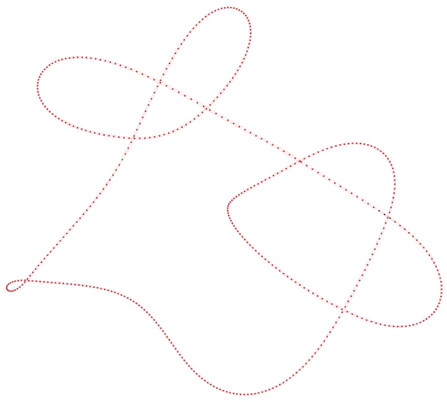	Finished circular assembly	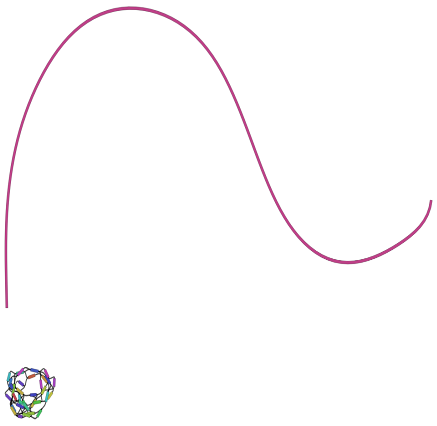
NCTC9555	*Staphylococcus aureus*	72	1	0	0	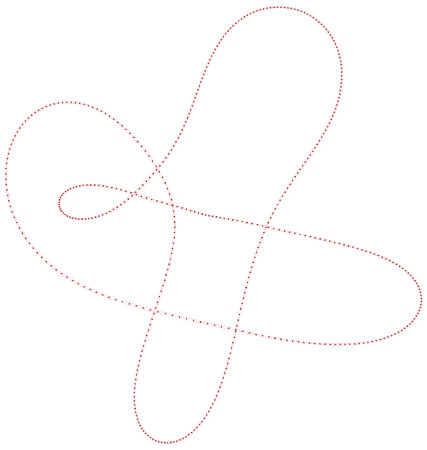	Finished circular assembly	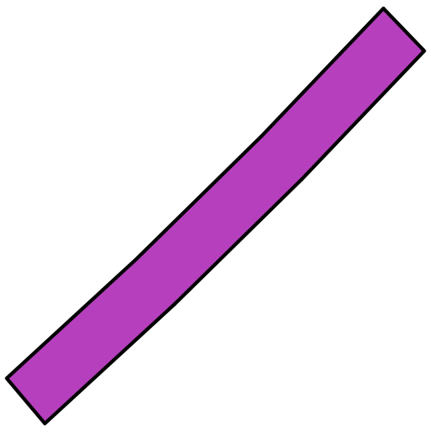
NCTC9556	*Staphylococcus aureus*	94	Pending	Pending	Pending	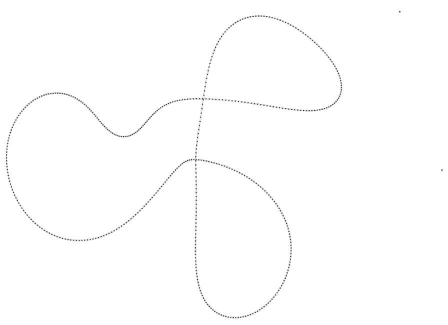	Finished circular assembly	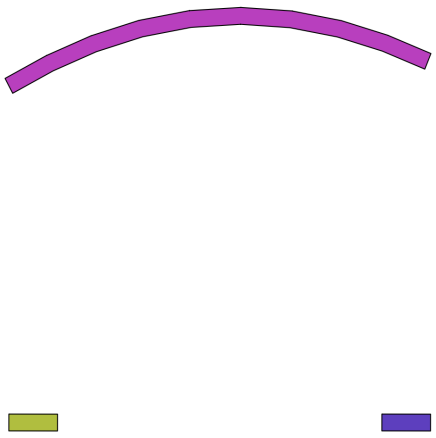
NCTC9601	*Klebsiella pneumoniae*	25	0	0	4	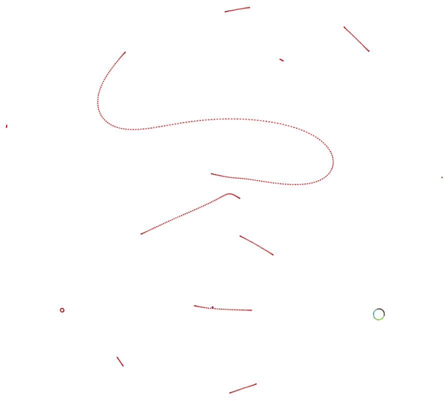	Mis-assembly/Fragmented	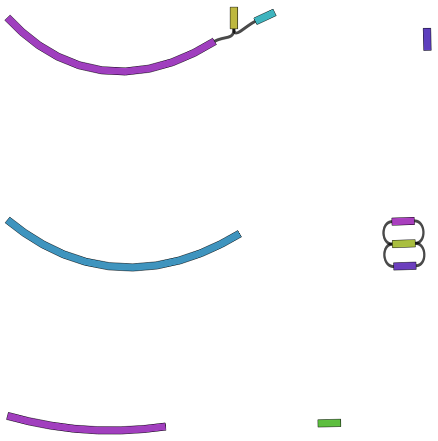
NCTC9603	*Streptococcus sp.*	125	Pending	Pending	Pending		Finished circular assembly	

						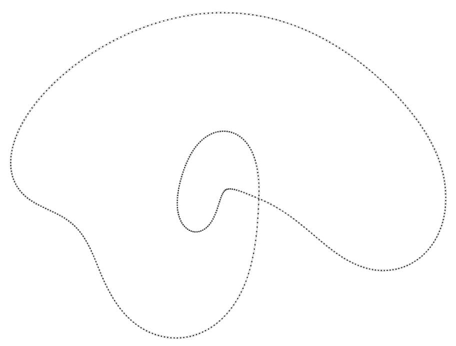		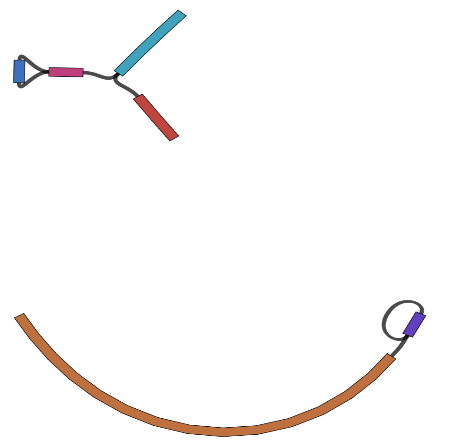
NCTC9606	*Salmonella sp*	45	0	0	3	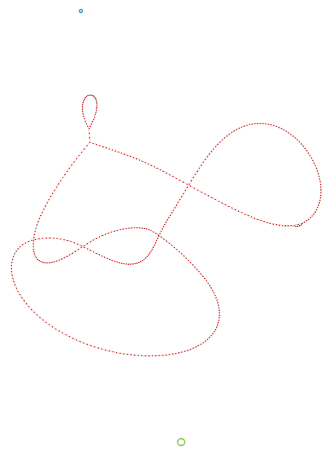	Finished assembly with multiple traversals	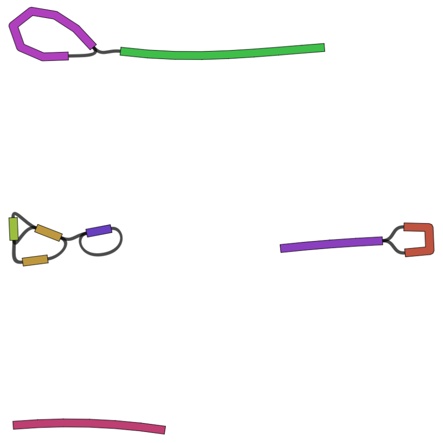
NCTC9611	*Staphylococcus aureus*	75	Pending	Pending	Pending	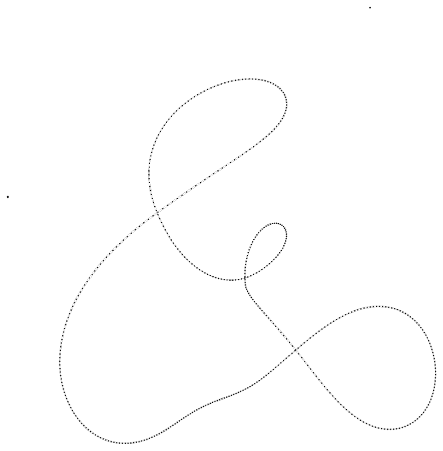	Finished circular assembly	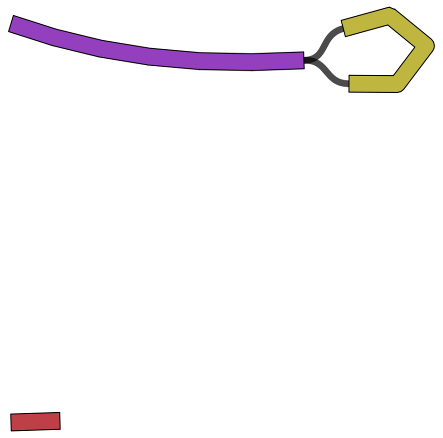
NCTC9612	*Staphylococcus aureus*	130	1	1	0	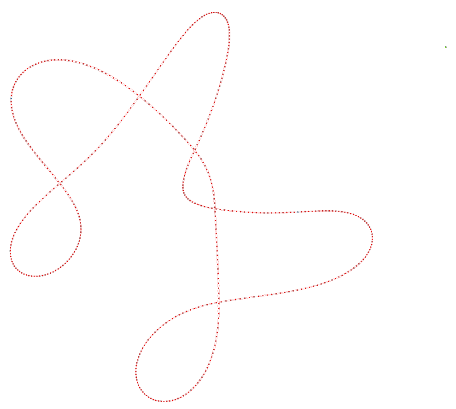	Finished circular assembly	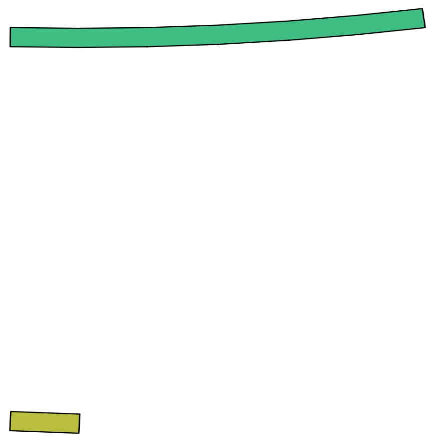
NCTC9613	*Staphylococcus aureus*	66	1	1	1	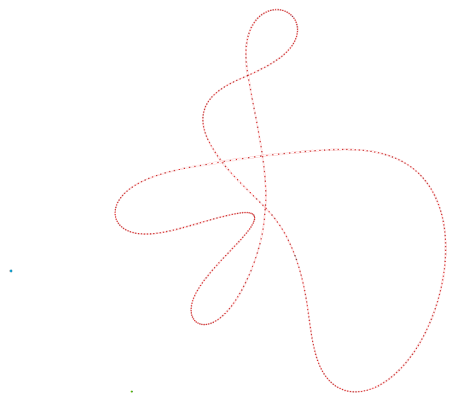	Finished circular assembly	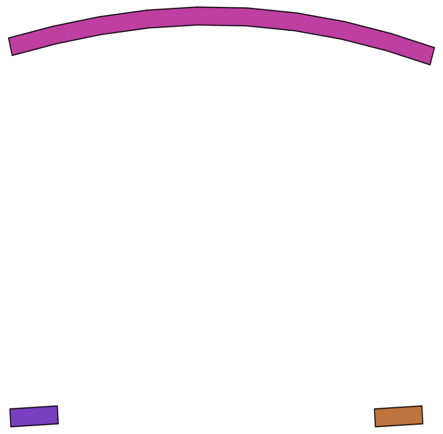
NCTC9614	*Staphylococcus aureus*	125	1	1	0	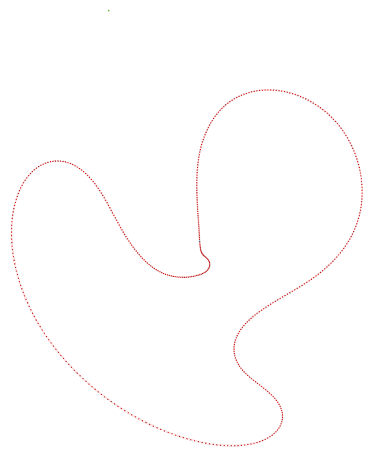	Finished circular assembly	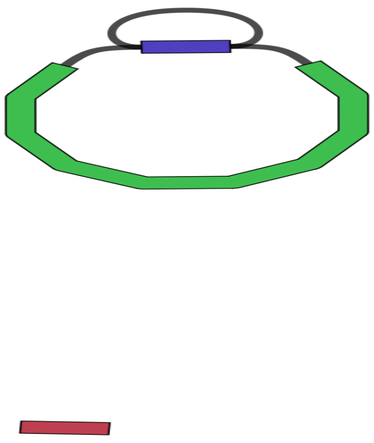
NCTC9616	*Klebsiella sp.*	45	0	0	4		Finished circular assembly	

						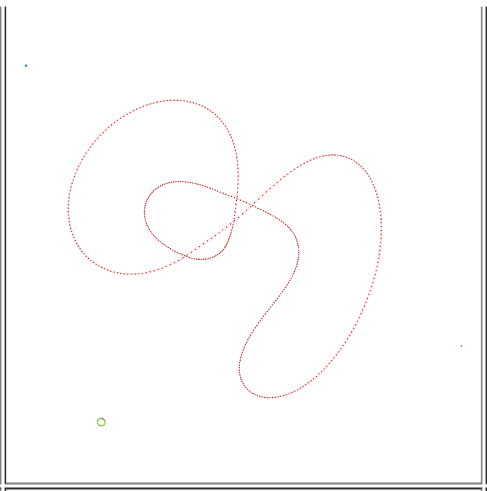		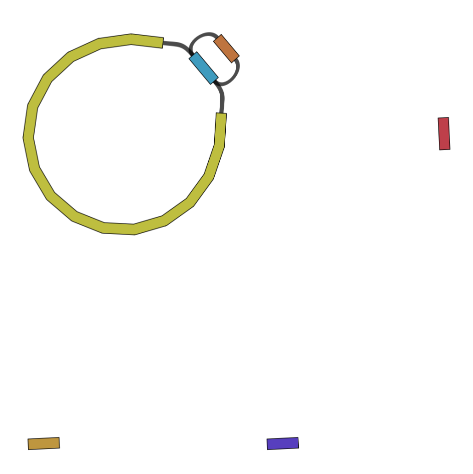
NCTC9617	*Klebsiella sp.*	22	0	0	9	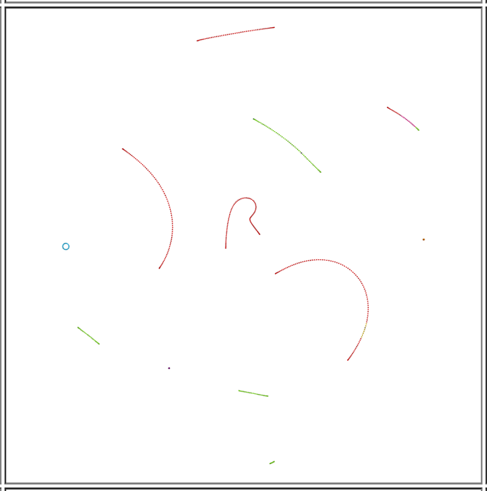	Mis-assembly/Fragmented	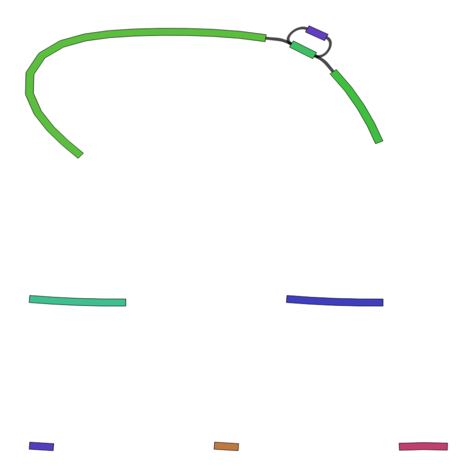
NCTC9632	*Klebsiella pneumoniae*	67	1	2	3	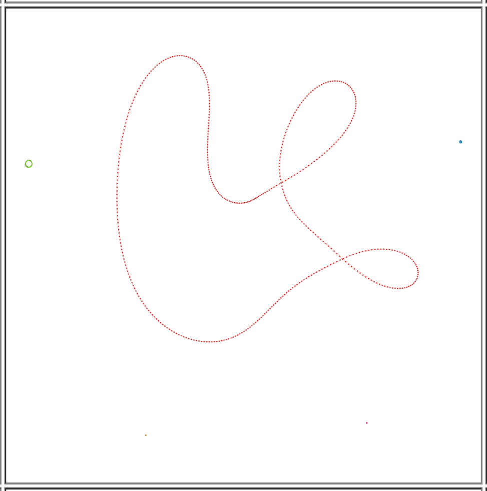	Finished circular assembly	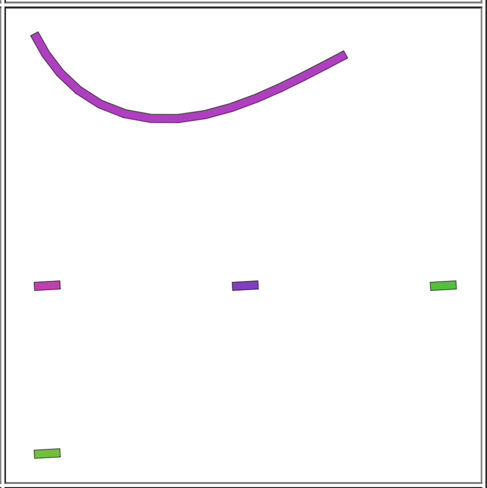
NCTC9635	*Klebsiella pneumoniae*	58	1	2	2	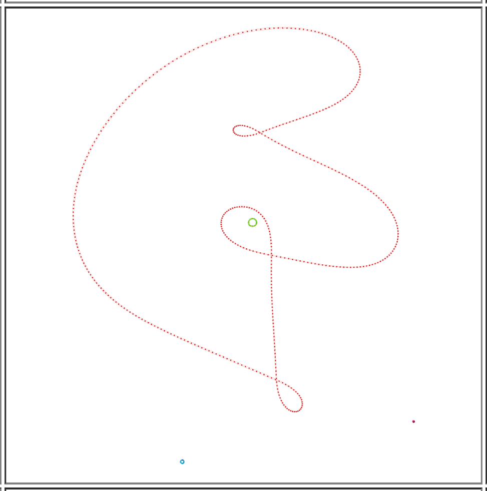	Finished circular assembly	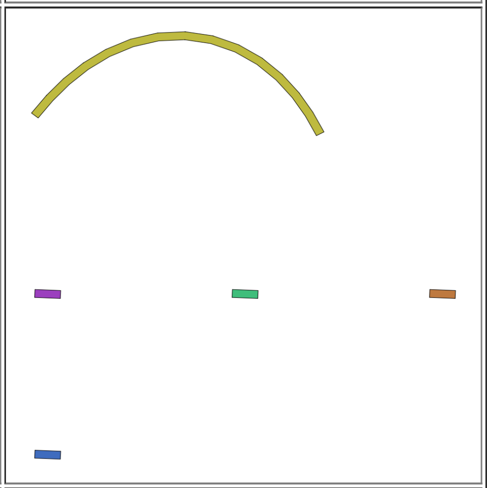
NCTC9637	*Klebsiella pneumoniae*	40	1	1	3	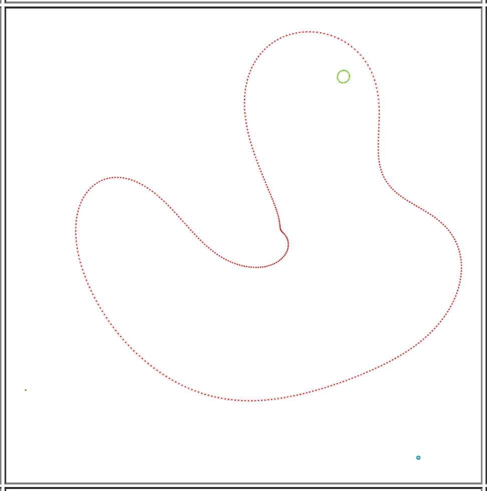	Finished circular assembly	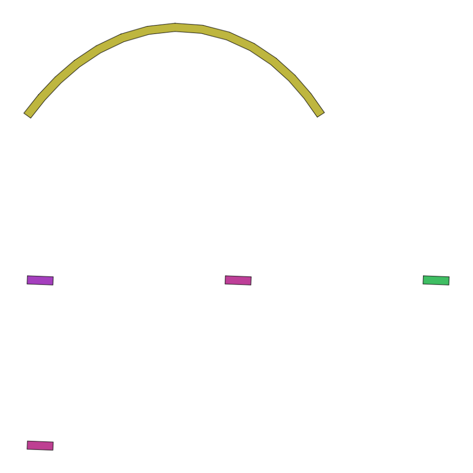
NCTC9644	*Enterobacter aerogenes*	66	Pending	Pending	Pending	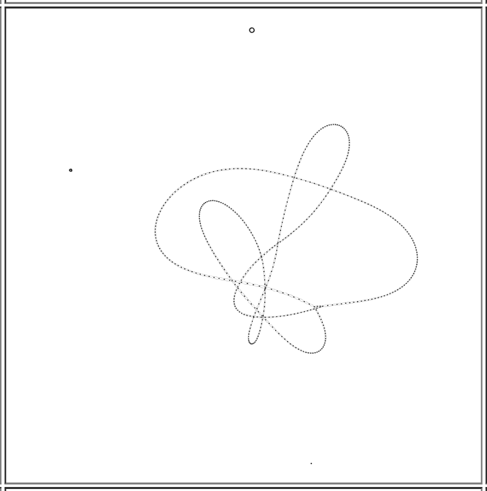	Finished assembly with multiple traversals	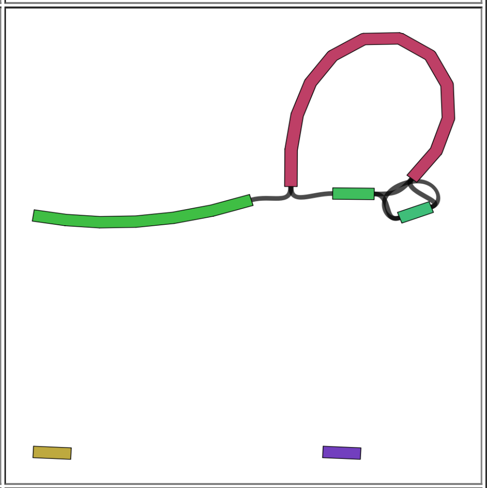
NCTC9645	*Klebsiella pneumoniae*	17	0	0	16		Mis-assembly/Fragmented	

						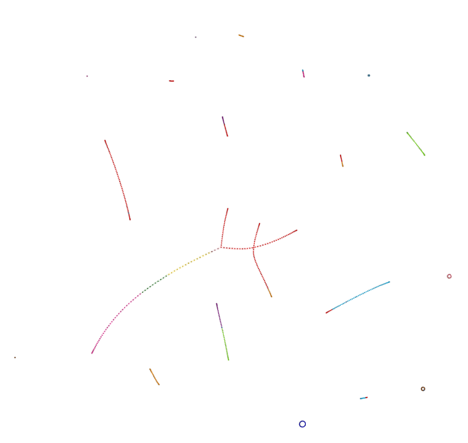		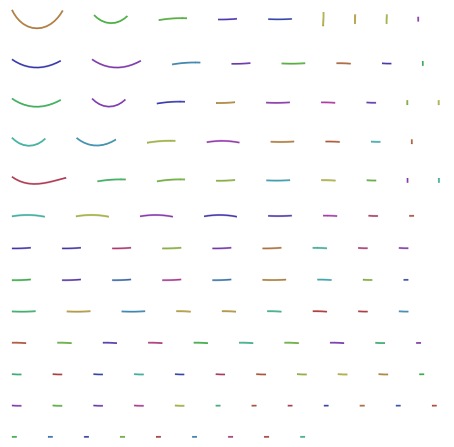
NCTC9646	*Klebsiella aerogenes*	24	Pending	Pending	Pending	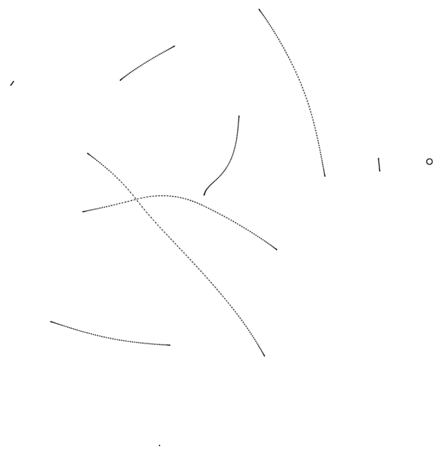	Mis-assembly/Fragmented	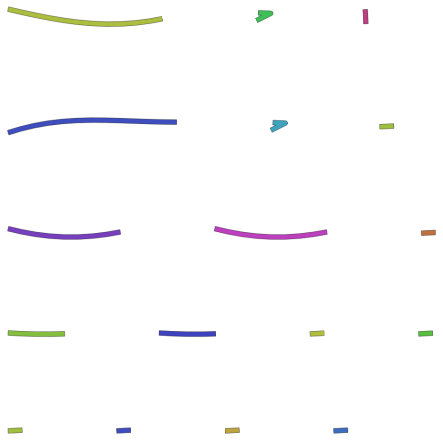
NCTC9652	*Enterobacter aerogenes*	34	Pending	Pending	Pending	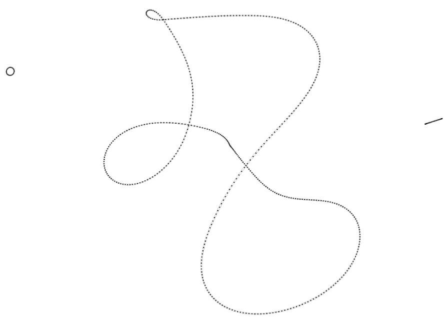	Finished circular assembly	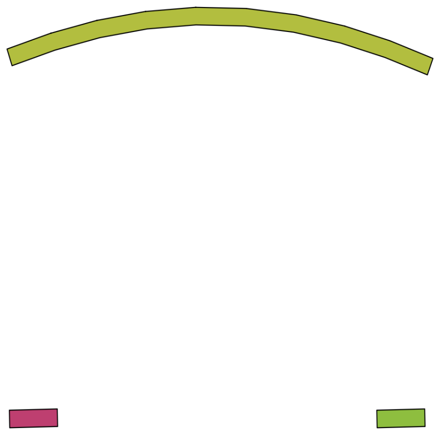
NCTC9656	*Klebsiella pneumoniae*	46	0	0	7	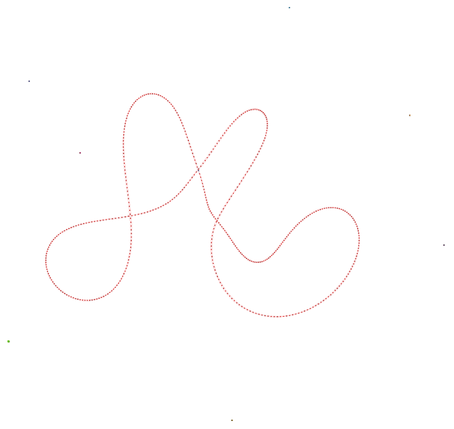	Finished circular assembly	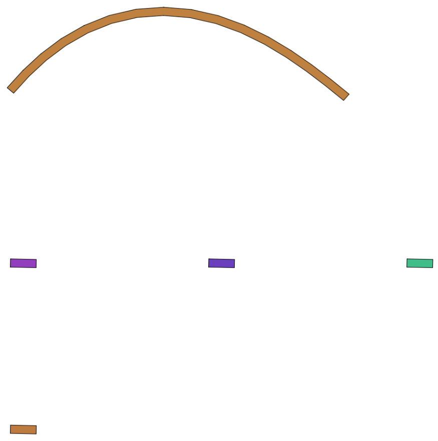
NCTC9657	*Klebsiella pneumoniae*	43	0	0	7	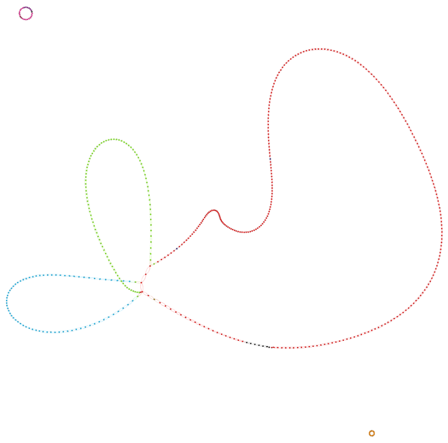	Finished assembly with multiple traversals	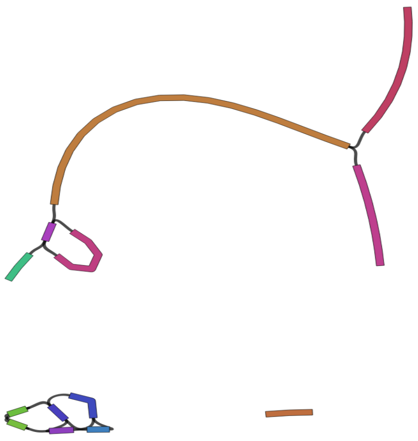
NCTC9660	*Klebsiella pneumoniae*	73	0	0	5	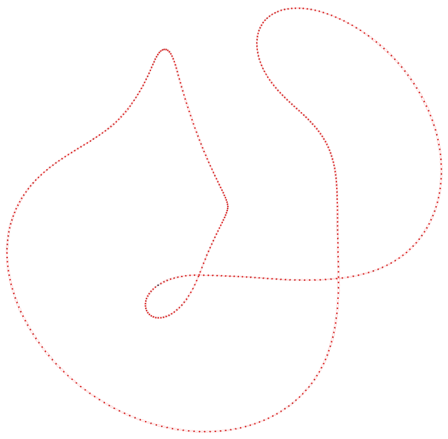	Finished circular assembly	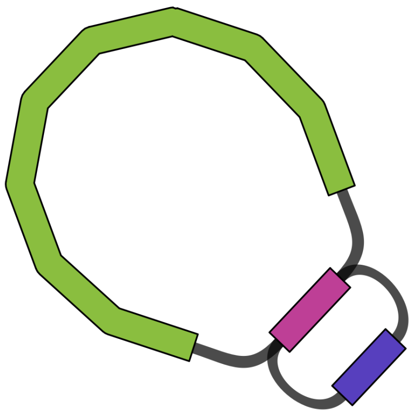
NCTC9661	*Klebsiella pneumoniae*	54	0	0	6		Finished circular assembly	

						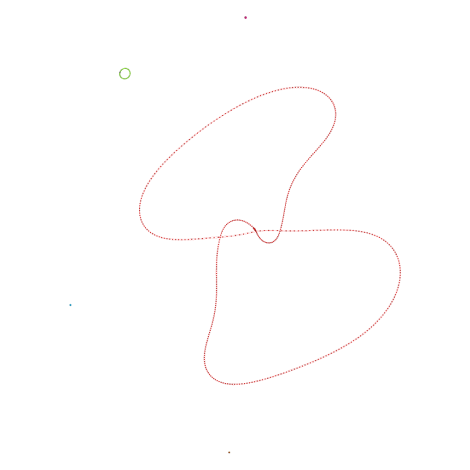		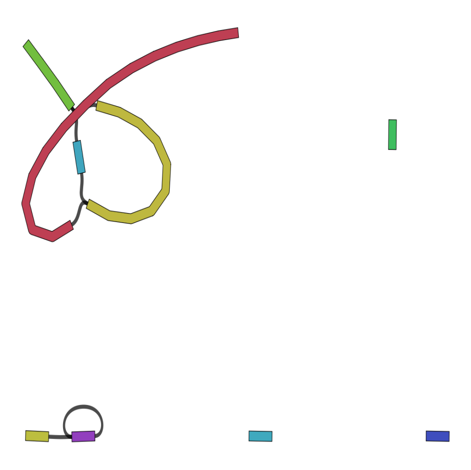
NCTC9662	*Klebsiella pneumoniae*	52	0	0	9	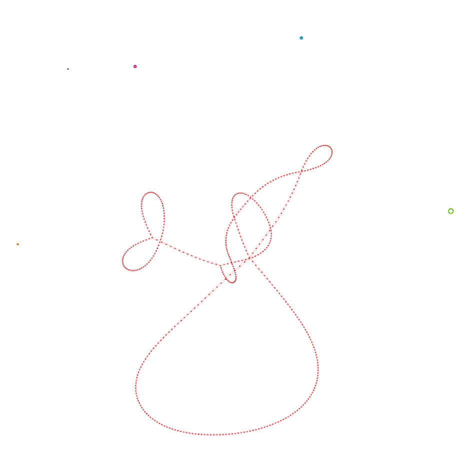	Mis-assembly/Fragmented	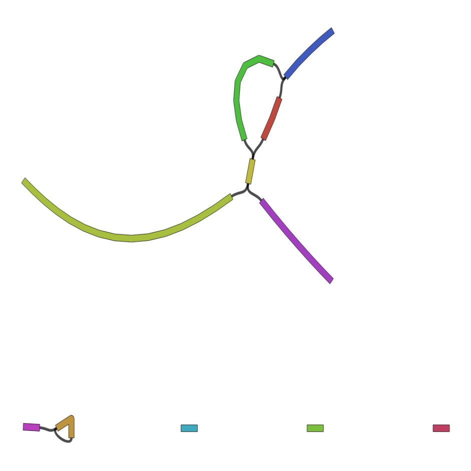
NCTC9667	*Enterobacter aerogenes*	39	0	0	2	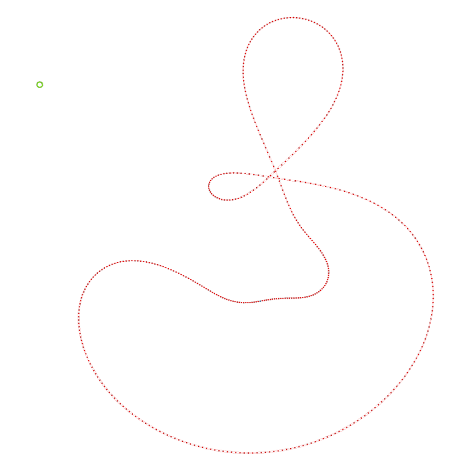	Finished circular assembly	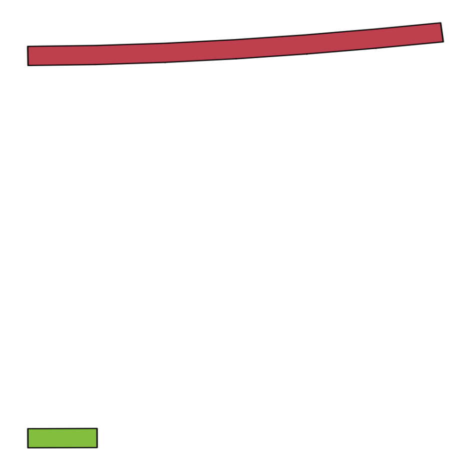
NCTC9668	*Enterobacter aerogenes*	58	0	0	1	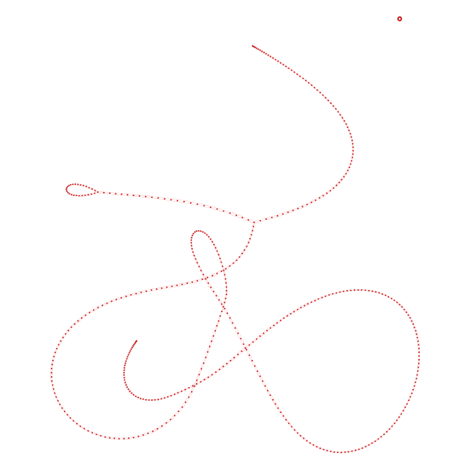	Mis-assembly/Fragmented	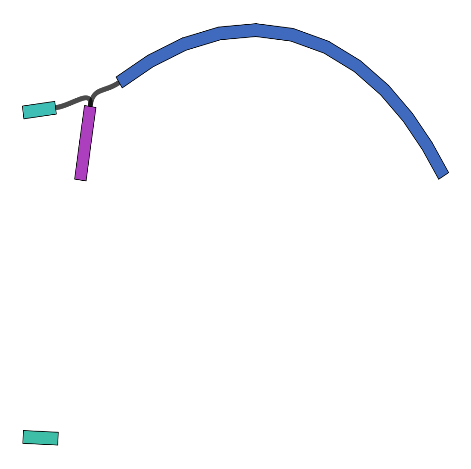
NCTC9684	*Salmonella enterica subsp. enterica*	31	1	0	0	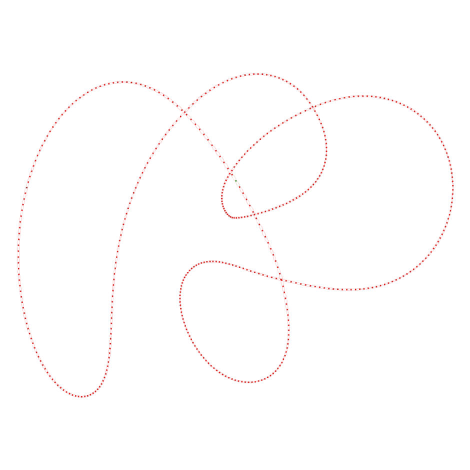	Finished circular assembly	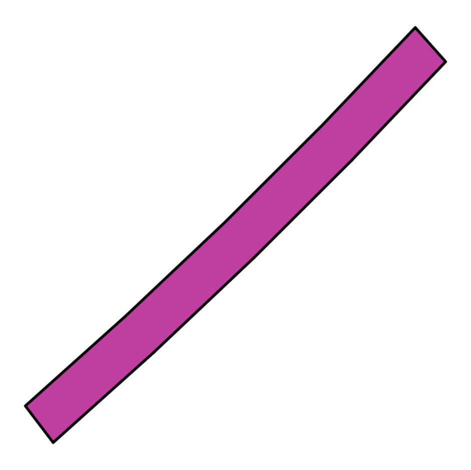
NCTC9695	*Chromobacterium violaceum*	34	1	0	0	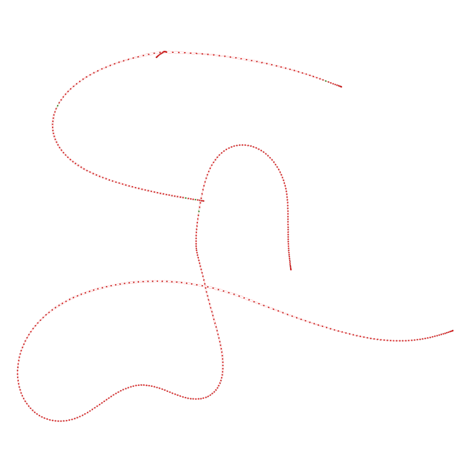	Mis-assembly/Fragmented	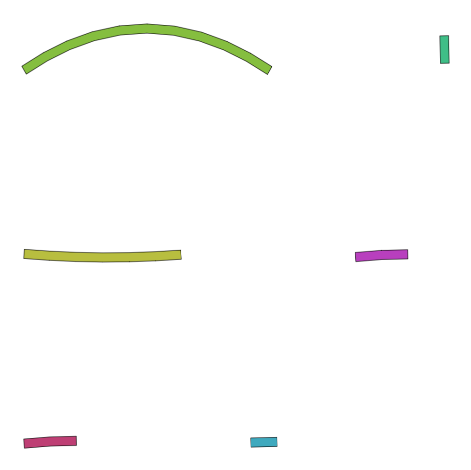
NCTC9699	*Escherichia coli*	77	1	0	0		Finished circular assembly	

						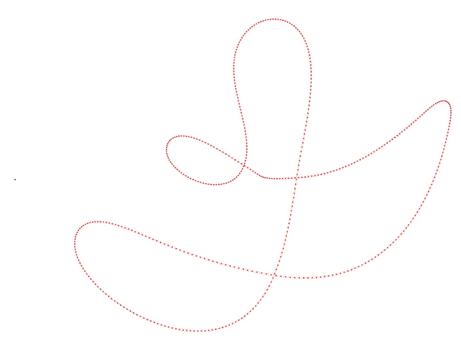		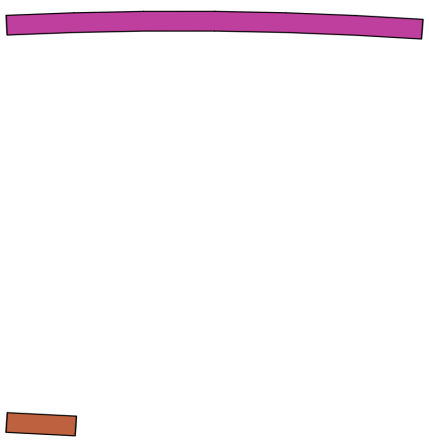
NCTC9701	*Escherichia coli*	85	1	1	0	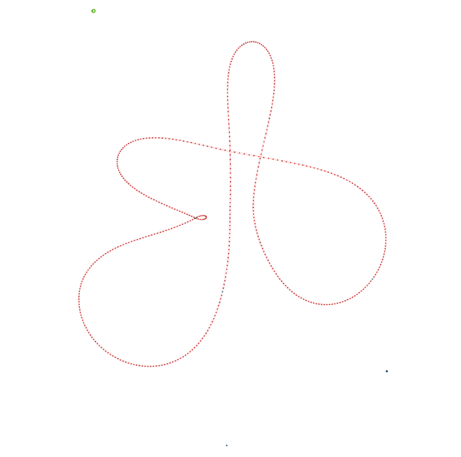	Finished circular assembly	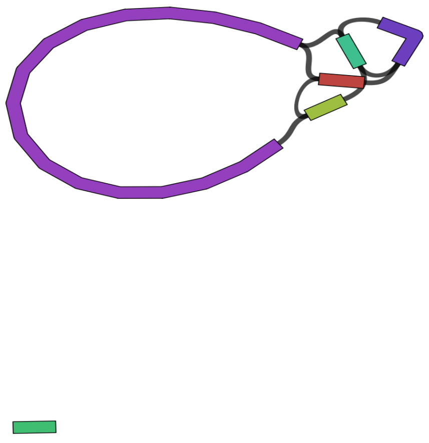
NCTC9702	*Escherichia coli*	36	1	0	0	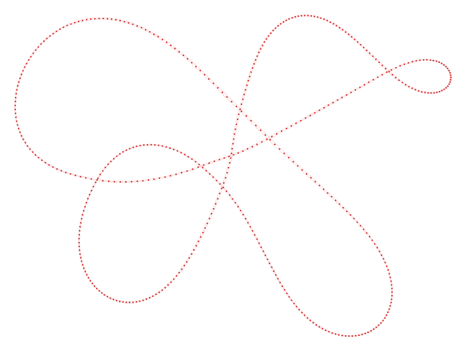	Finished circular assembly	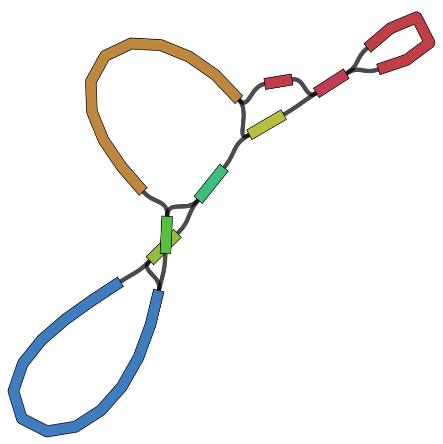
NCTC9703	*Escherichia coli*	57	Pending	Pending	Pending	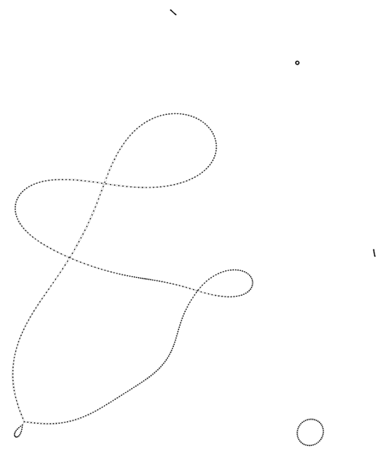	Finished assembly with multiple traversals	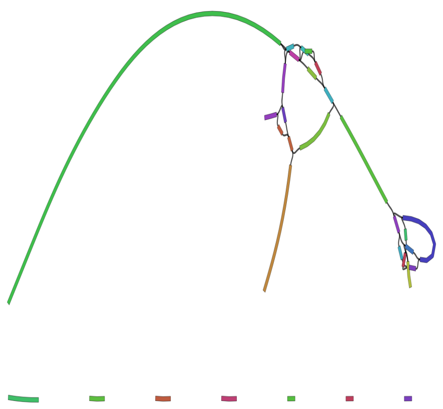
NCTC9705	*Escherichia coli*	102	Pending	Pending	Pending	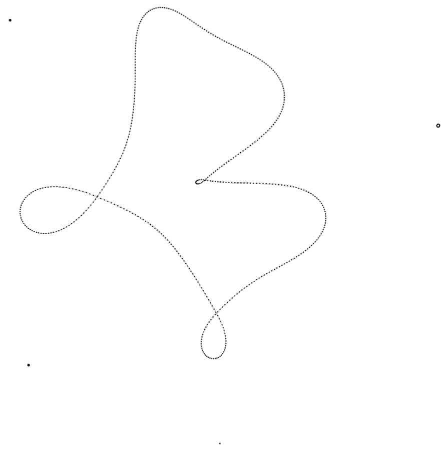	Finished circular assembly	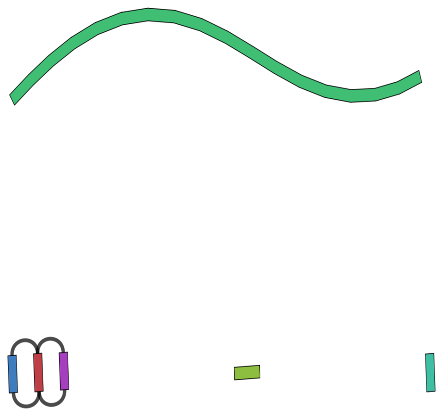
NCTC9707	*Escherichia coli*	71	0	0	6	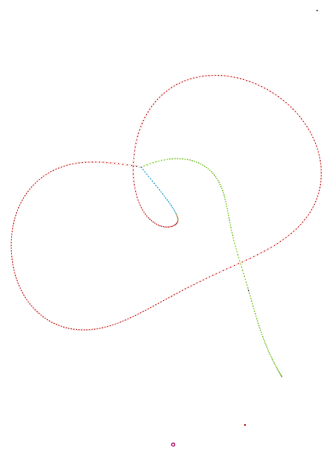	Mis-assembly/Fragmented	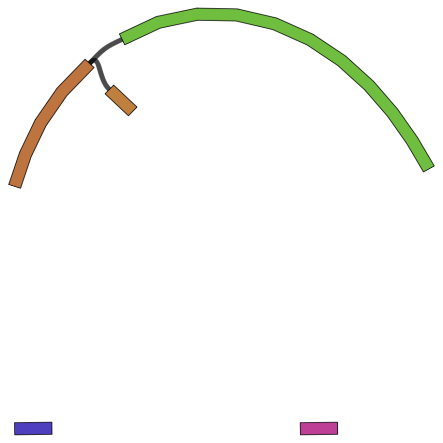
NCTC9708	*Escherichia coli*	50	1	1	4		Possible mis-assembly	

						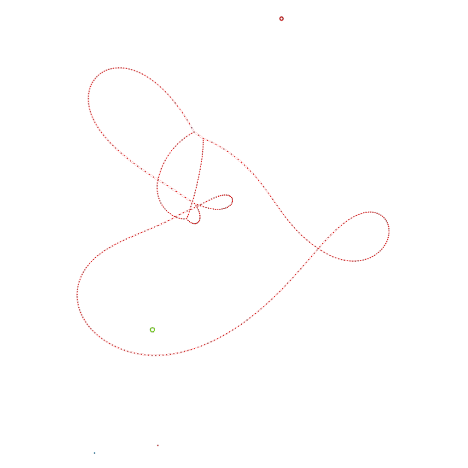		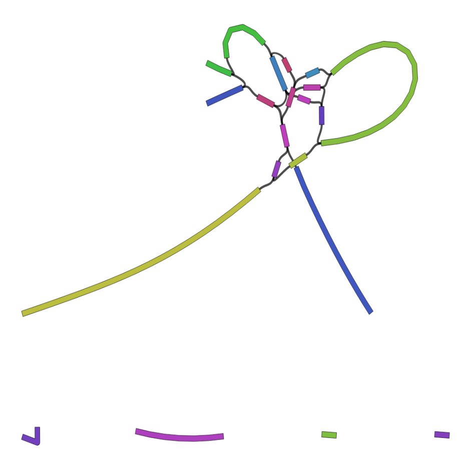
NCTC9750	*Citrobacter freundii*	78	1	0	0	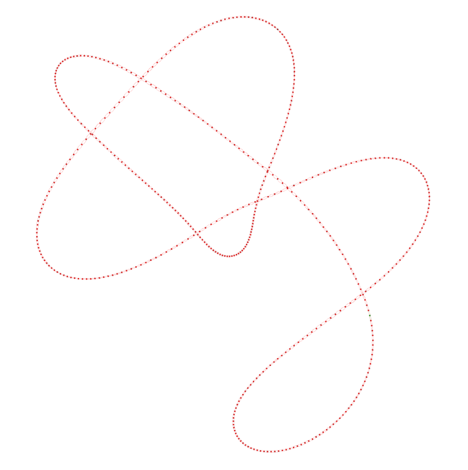	Finished circular assembly	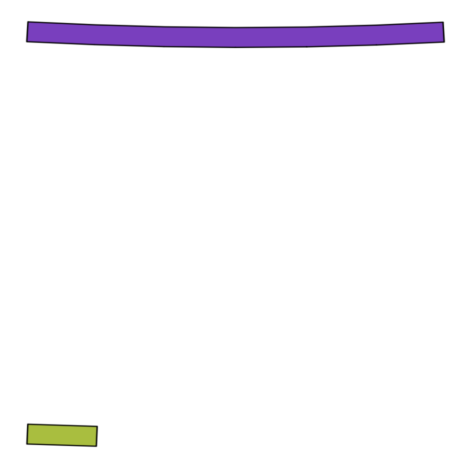
NCTC9752	*Staphylococcus aureus*	112	1	0	0	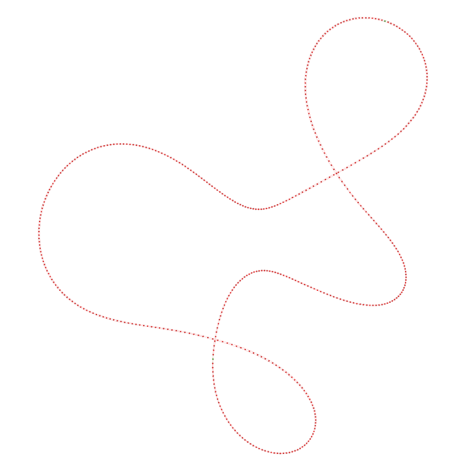	Finished circular assembly	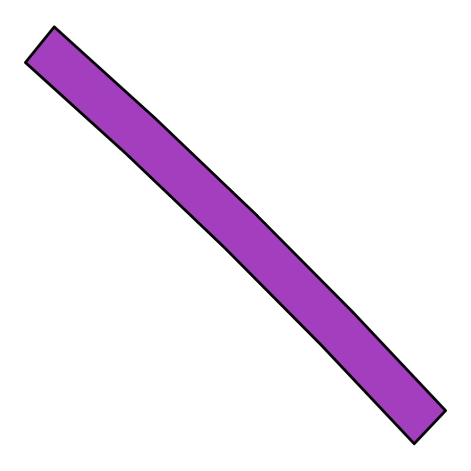
NCTC9757	*Chromobacterium violaceum*	27	Pending	Pending	Pending	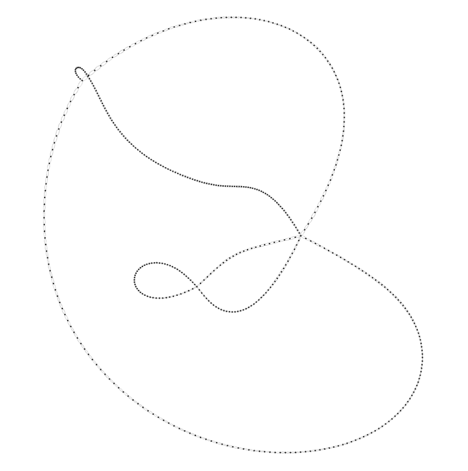	Mis-assembly/Fragmented	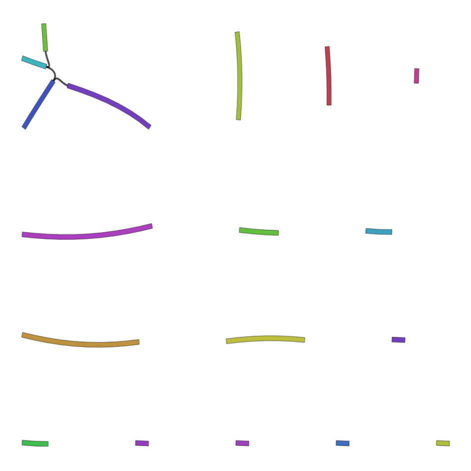
NCTC9775	*Escherichia coli*	43	0	0	7	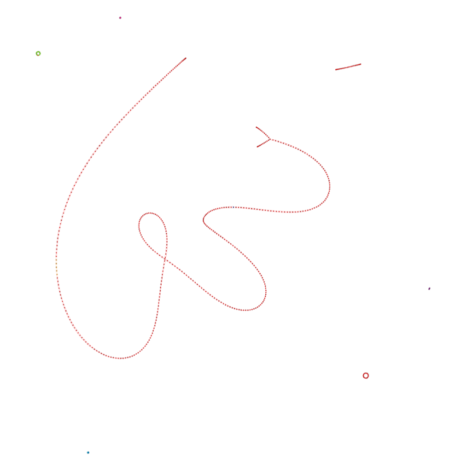	Finished assembly (lacking circularisation)	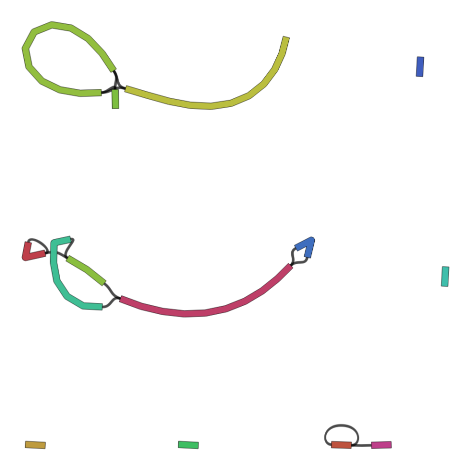
NCTC9776	*Escherichia coli*	61	1	1	0	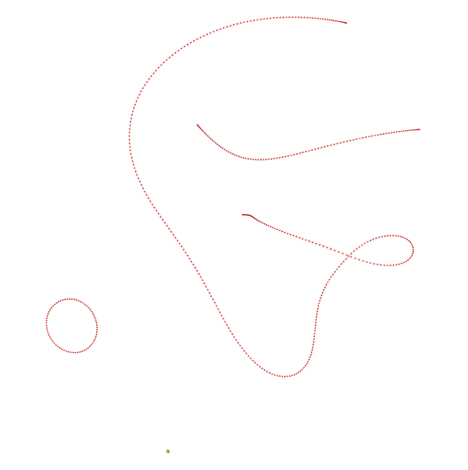	Mis-assembly/Fragmented	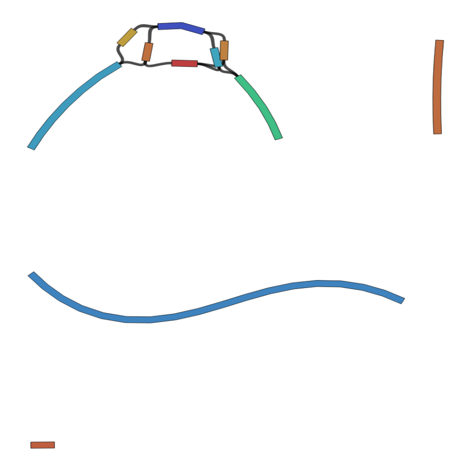
NCTC9777	*Escherichia coli*	59	0	0	11		Mis-assembly/Fragmented	

						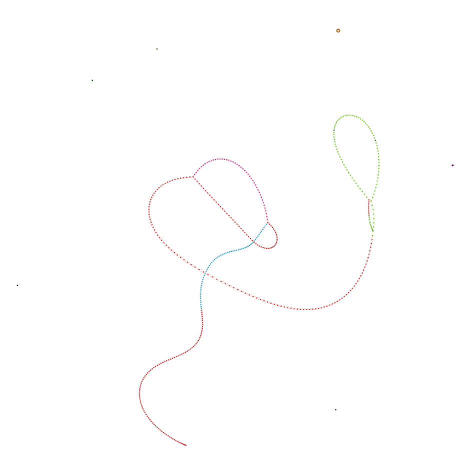		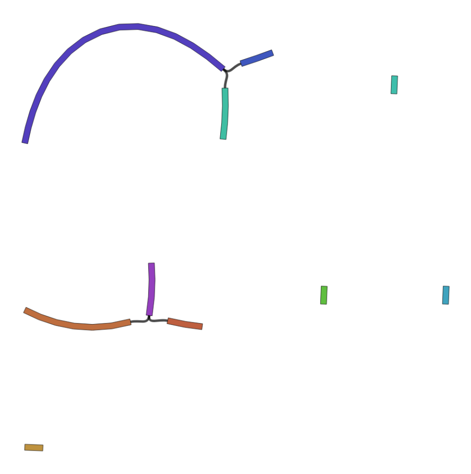
NCTC9778	*Escherichia coli*	35	Pending	Pending	Pending	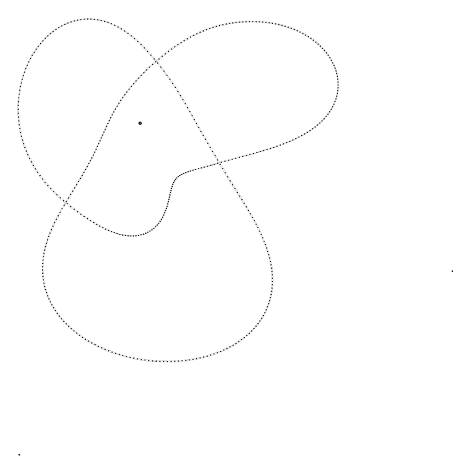	Finished circular assembly	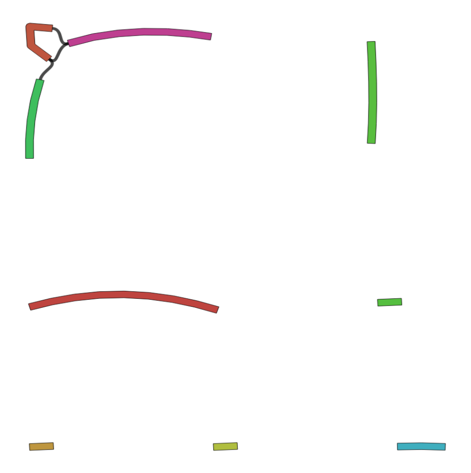
NCTC9793	*Enterobacter aerogenes*	57	Pending	Pending	Pending	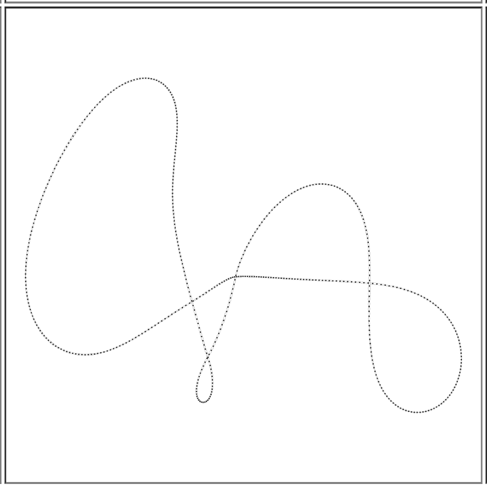	Finished circular assembly	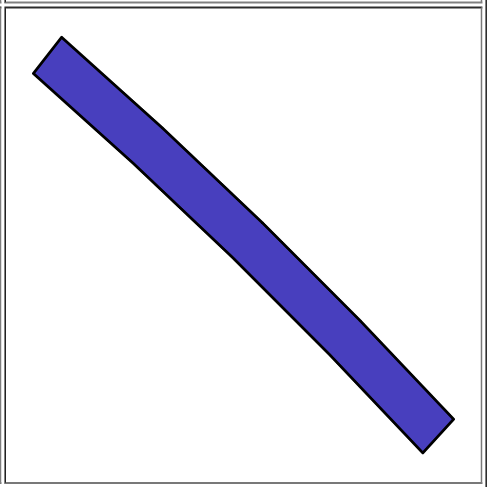
NCTC9828	*Streptococcus agalactiae*	24	Pending	Pending	Pending	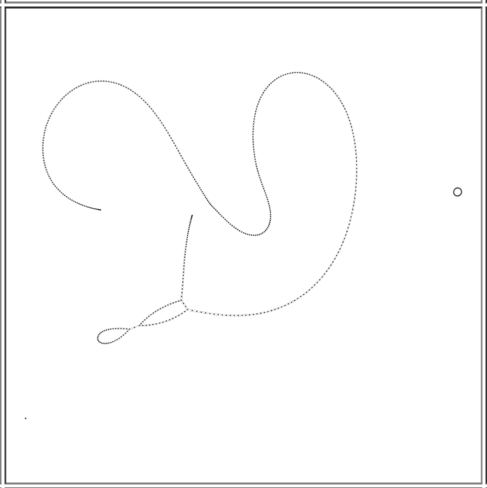	Mis-assembly/Fragmented	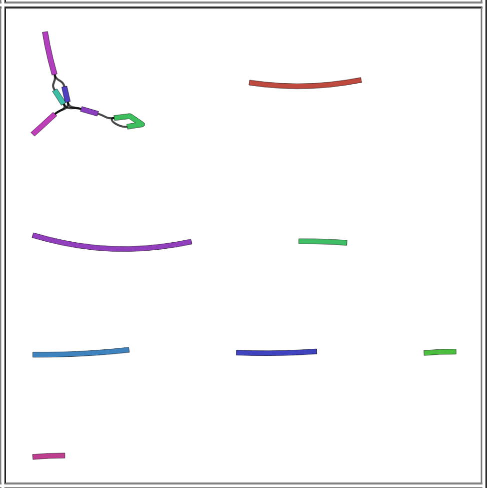
NCTC9855	*Escherichia coli*	54	1	2	1	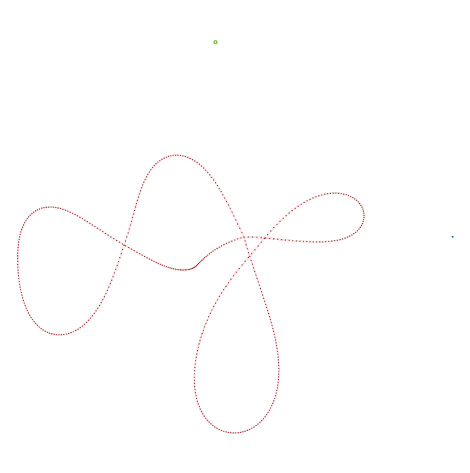	Finished circular assembly	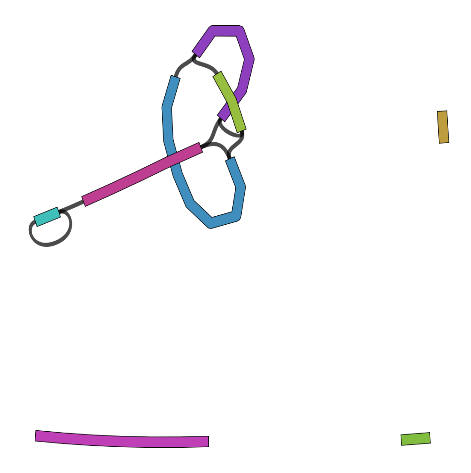
NCTC9868	*Salmonella enterica subsp. enterica*	57	1	1	0	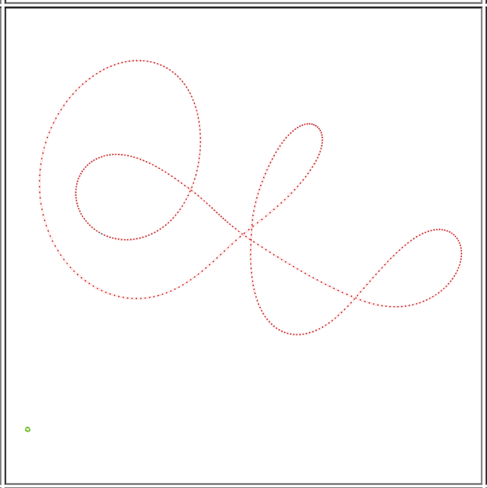	Finished circular assembly	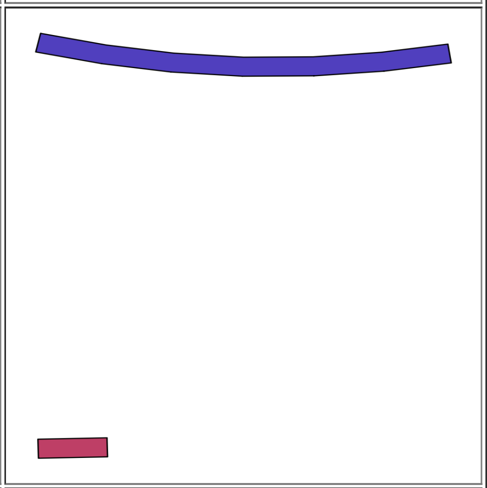
NCTC9944	*Staphylococcus aureus*	79	1	0	0		Finished circular assembly	

						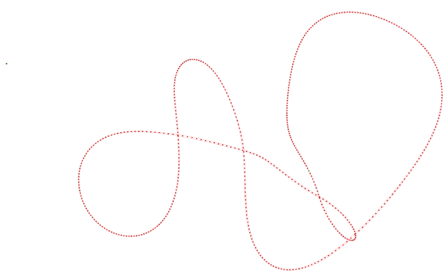		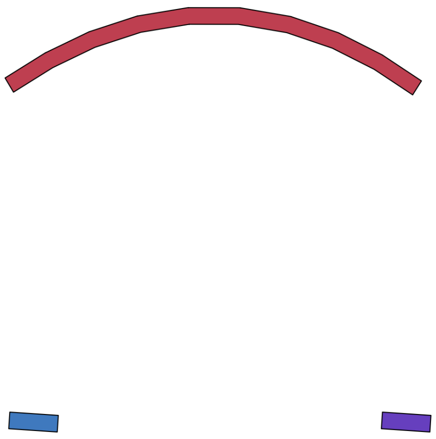
NCTC9948	*Salmonella enterica*	56	1	0	0	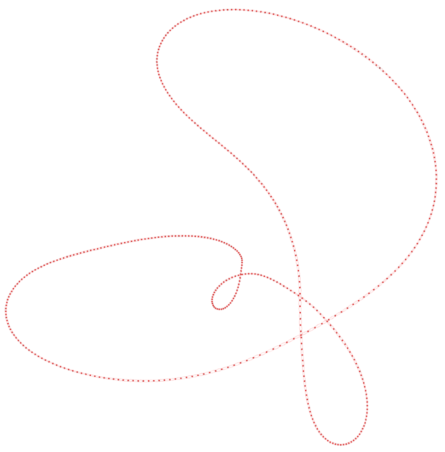	Finished circular assembly	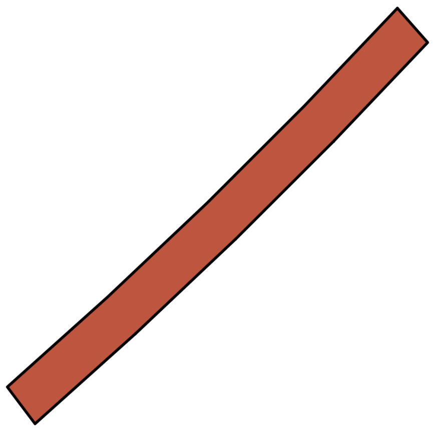
NCTC9961	*Escherichia coli*	0	0	0	2	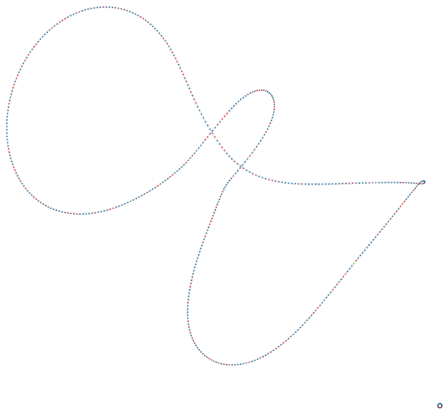	Finished circular assembly	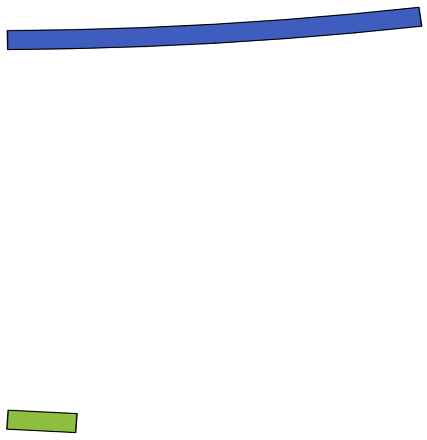
NCTC9962	*Escherichia coli*	26	0	0	23	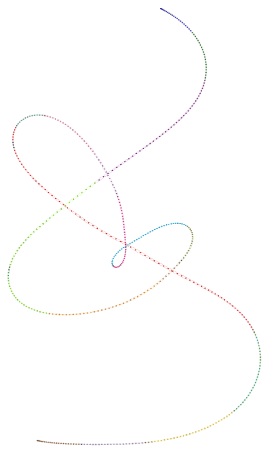	Finished assembly (lacking circularisation)	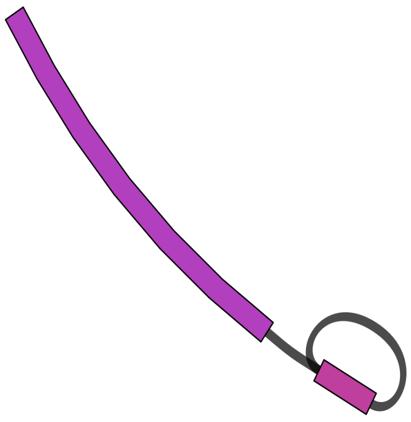
NCTC9963	*Escherichia coli*	45	1	0	2	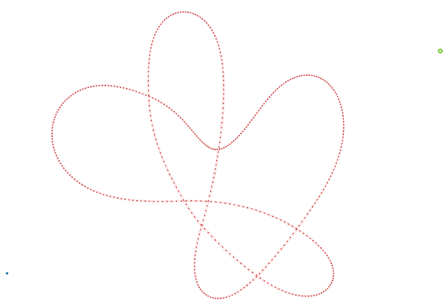	Finished circular assembly	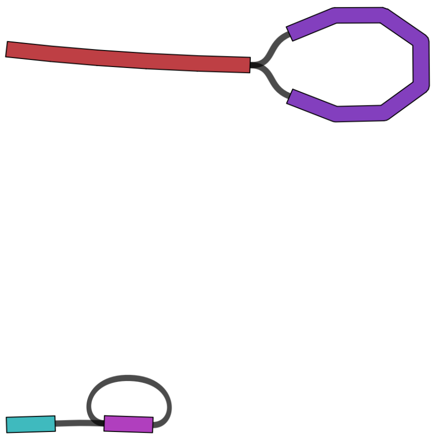
NCTC9964	*Escherichia coli*	63	1	0	1	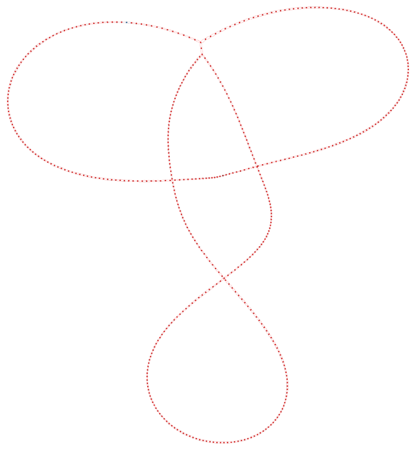	Finished assembly with multiple traversals	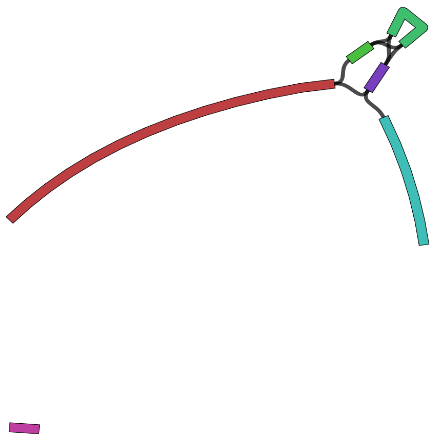
NCTC9965	*Escherichia coli*	35	1	1	2		Finished circular assembly	

						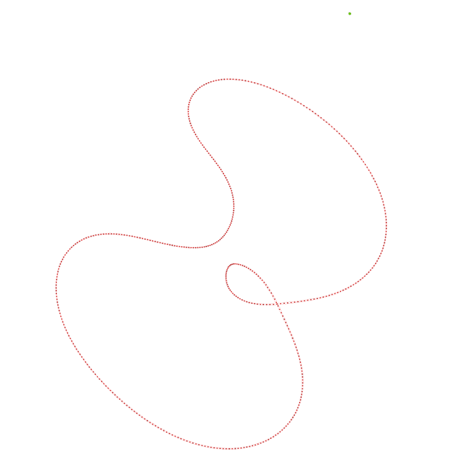		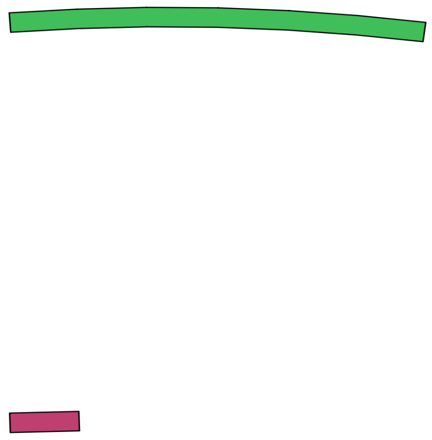
NCTC9966	*Escherichia coli*	54	1	0	0	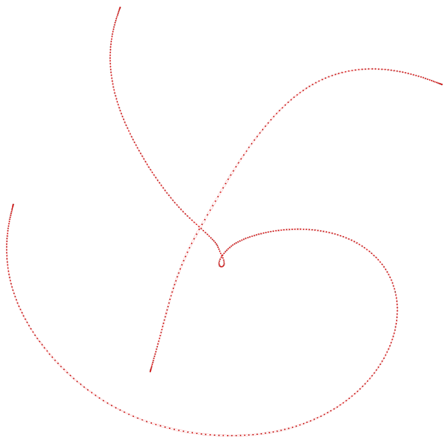	Mis-assembly/Fragmented	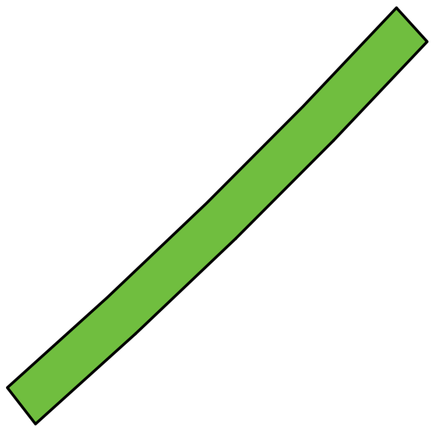
NCTC9967	*Escherichia coli*	82	1	0	0	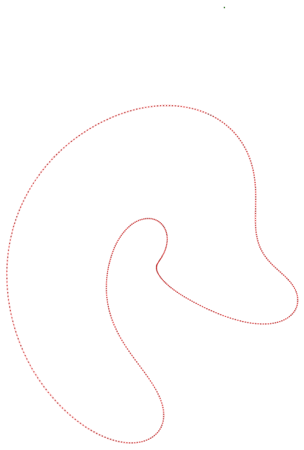	Finished circular assembly	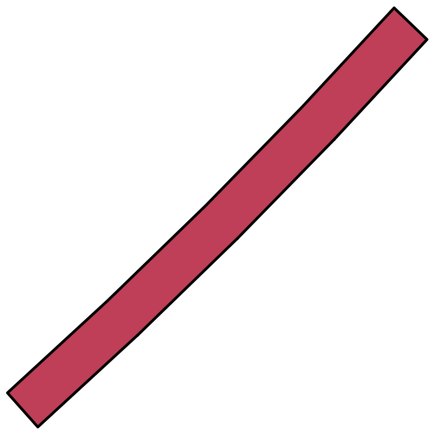
NCTC9969	*Escherichia coli*	59	1	2	4	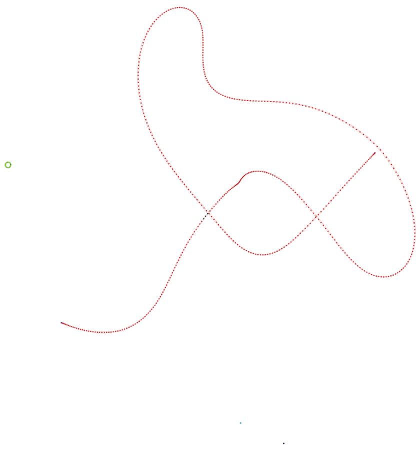	Finished assembly (lacking circularisation)	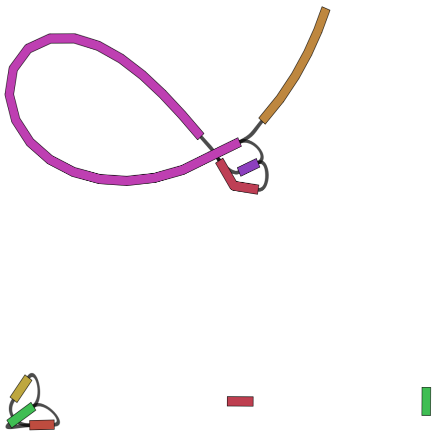
NCTC9997	*Enterobacter aerogenes*	42	1	0	2	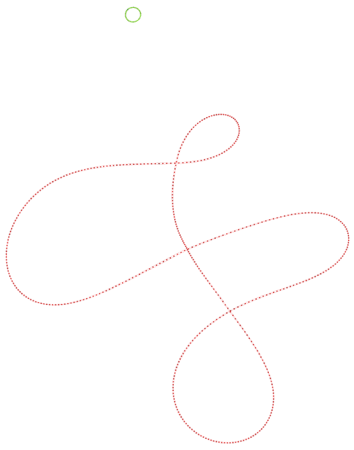	Finished circular assembly	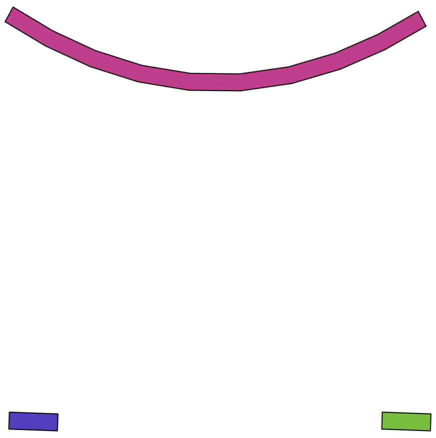
NCTC10002	*Escherichia coli*	63	0	0	5	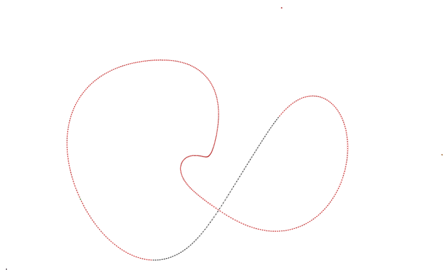	Finished circular assembly	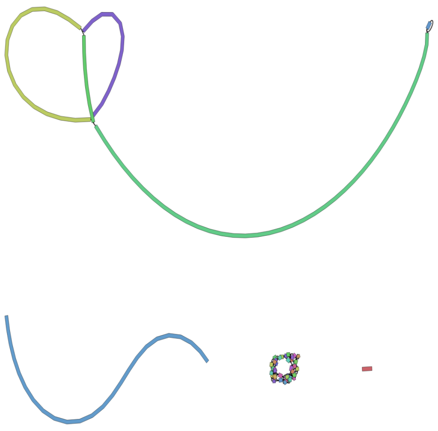
NCTC10004	*Klebsiella aerogenes*	58	0	0	4		Finished circular assembly	

						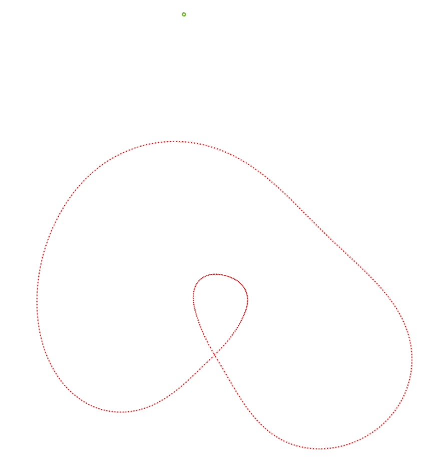		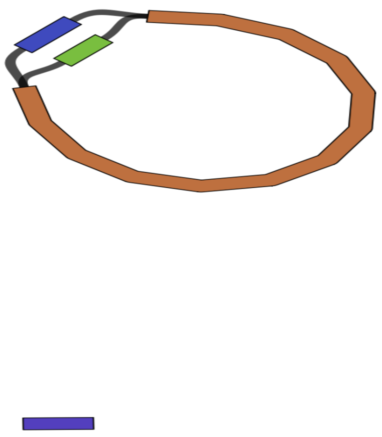
NCTC10005	*Enterobacter cloacae*	67	1	0	6	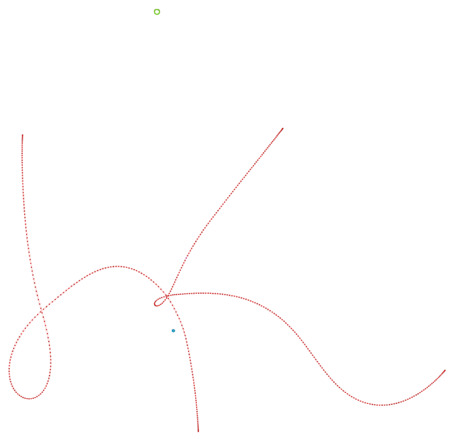	Mis-assembly/Fragmented	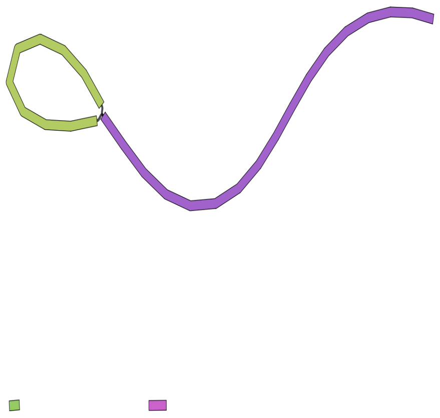
NCTC10006	*Enterobacter aerogenes*	56	1	0	0	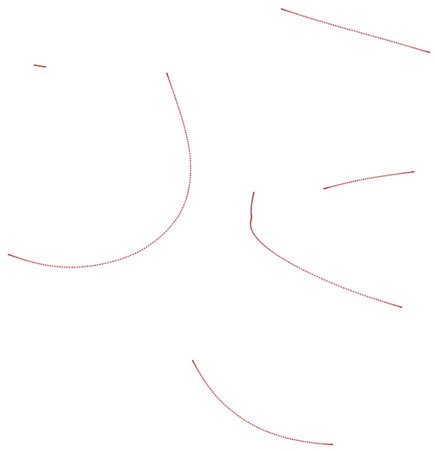	Mis-assembly/Fragmented	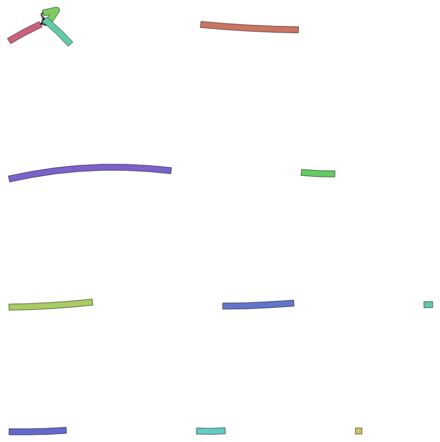
NCTC10016	*Elizabethkingia meningoseptica*	63	Pending	Pending	Pending	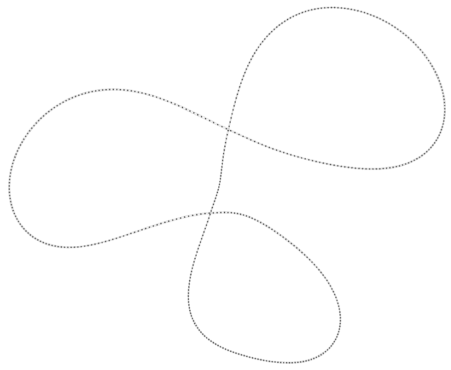	Finished circular assembly	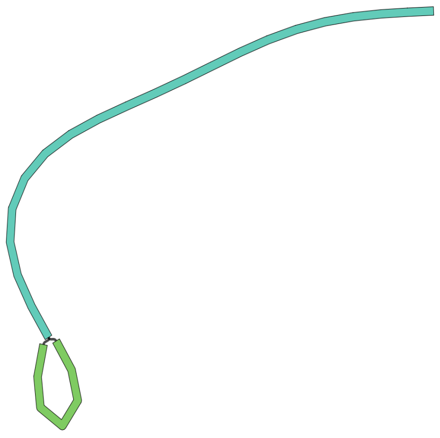
NCTC10035	*Staphylococcus aureus*	126	1	2	1	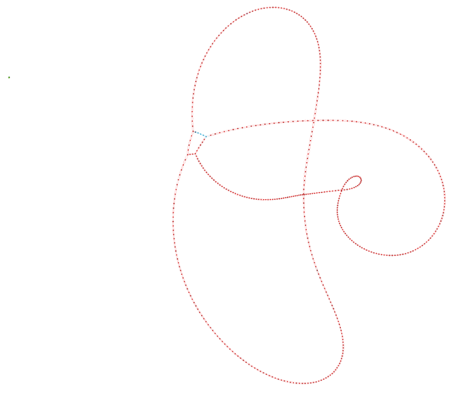	Finished assembly with multiple traversals	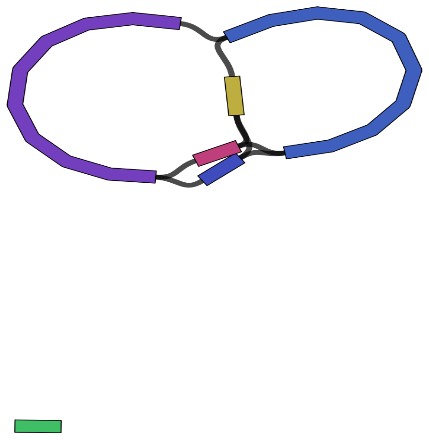
NCTC10038	*Pseudomonas fluorescens*	41	Pending	Pending	Pending	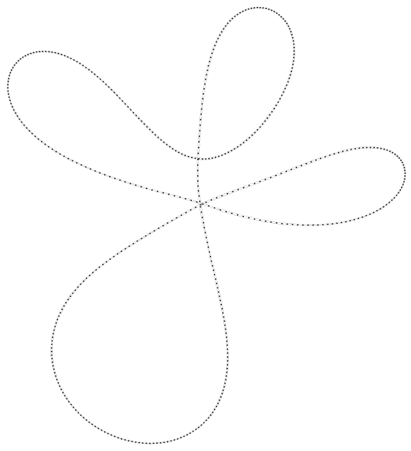	Finished circular assembly	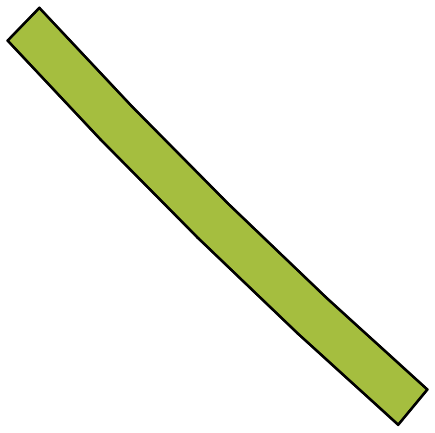
NCTC10047	*Salmonella enterica subsp. arizonae*	52	1	0	0		Finished circular assembly	

						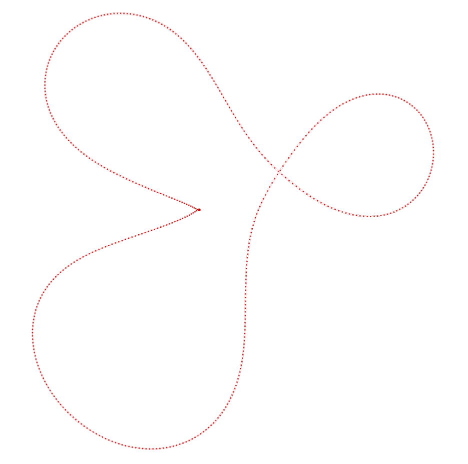		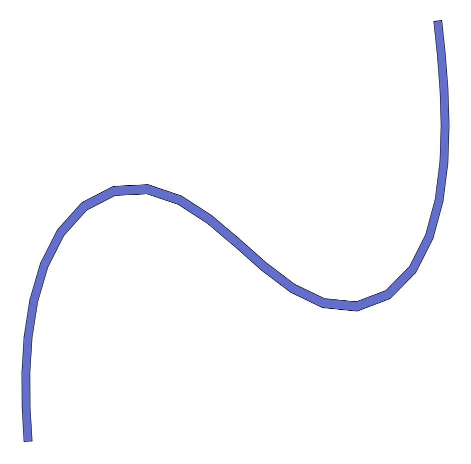
NCTC10060	*Salmonella enterica subsp. diarizonae*	41	0	0	5	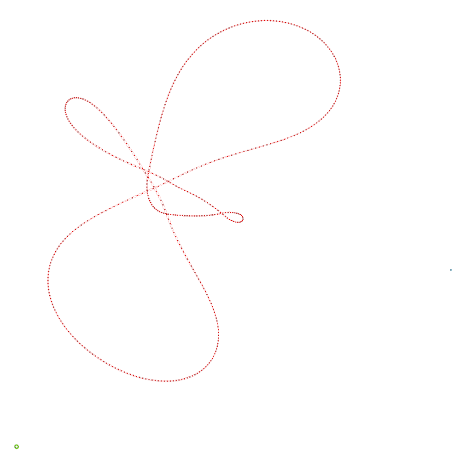	Finished circular assembly	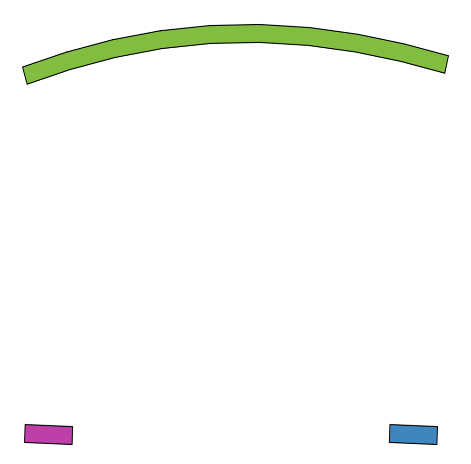
NCTC10081	*Salmonella enterica subsp. enterica*	55	1	0	2	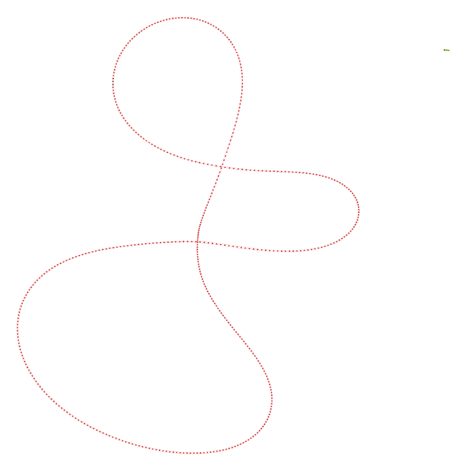	Finished circular assembly	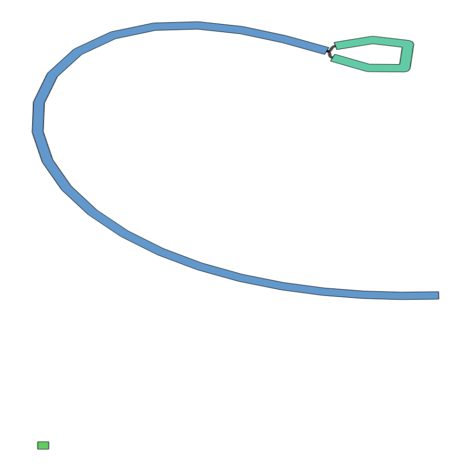
NCTC10086	*Escherichia coli*	62	1	0	6	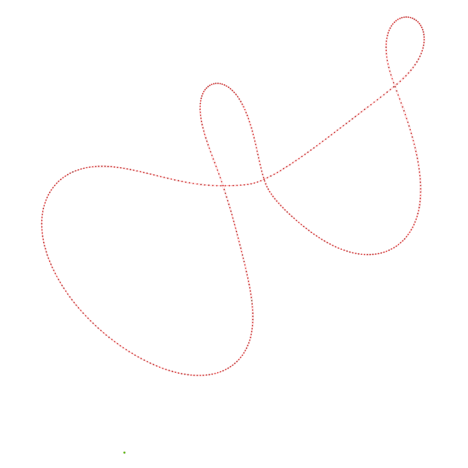	Finished circular assembly	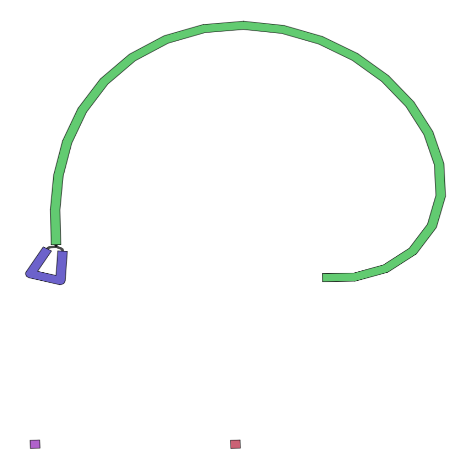
NCTC10087	*Escherichia coli*	58	1	1	0	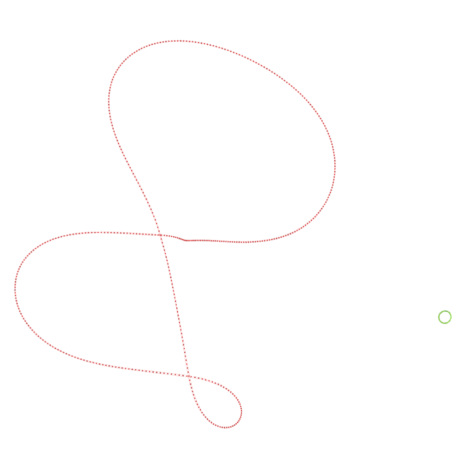	Finished circular assembly	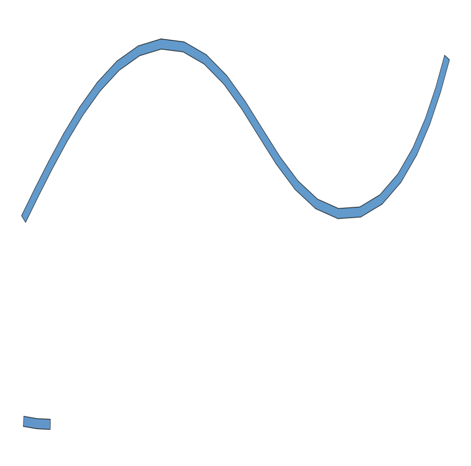
NCTC10089	*Escherichia coli*	70	1	3	3	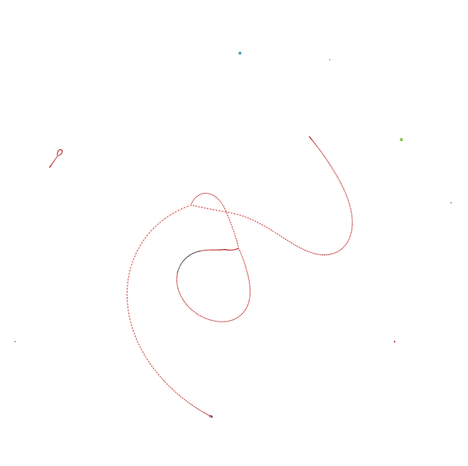	Mis-assembly/Fragmented	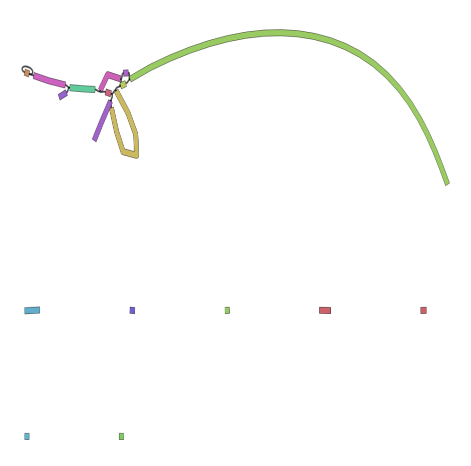
NCTC10090	*Escherichia coli*	79	Pending	Pending	Pending		Mis-assembly/Fragmented	

						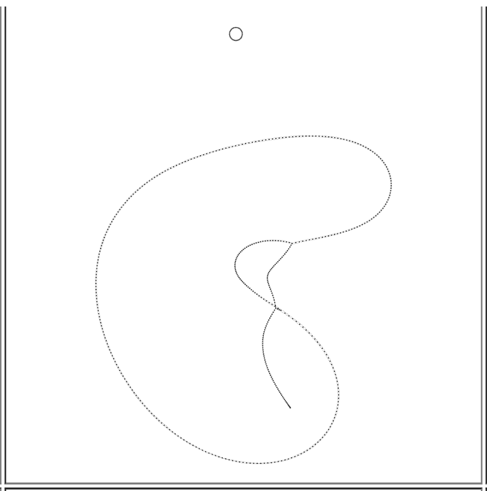		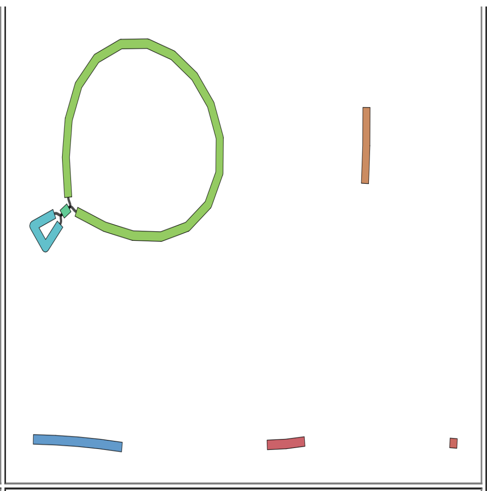
NCTC10211	*Serratia marcescens*	67	0	0	3	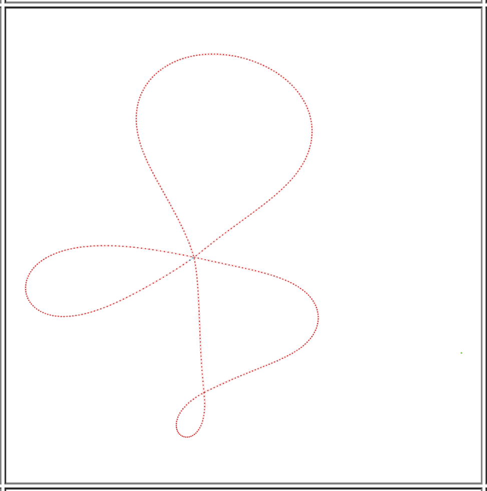	Finished circular assembly	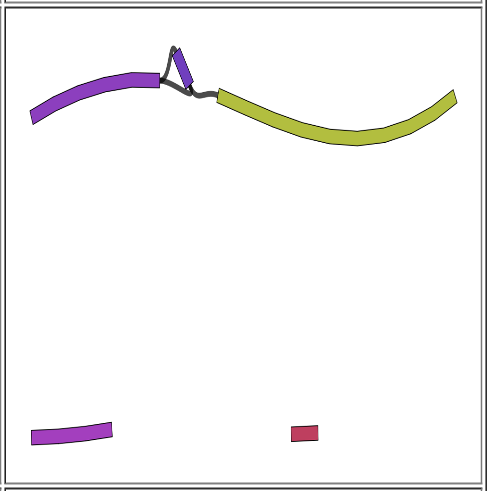
NCTC10212	*Neisseria animalis*	106	Pending	Pending	Pending	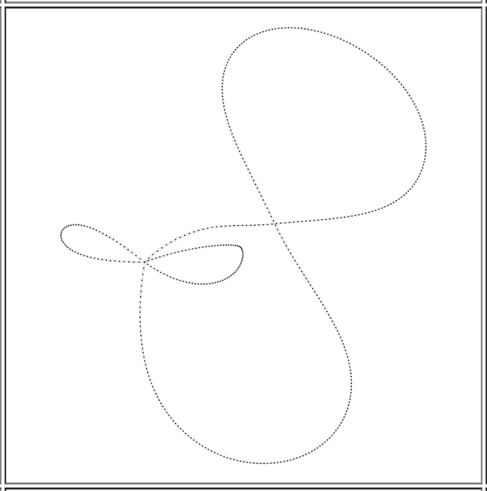	Finished assembly with multiple traversals	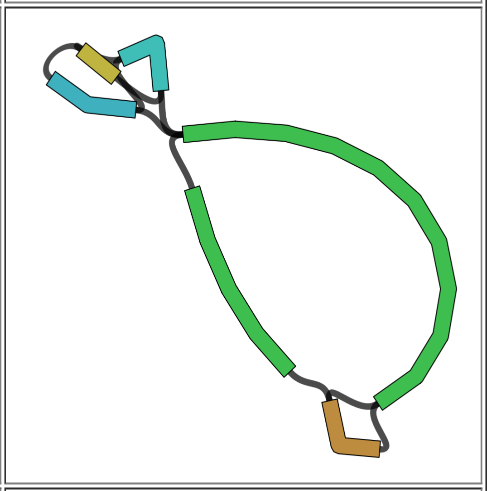
NCTC10213	*Klebsiella edwardsii var. atlantae*	55	1	1	5	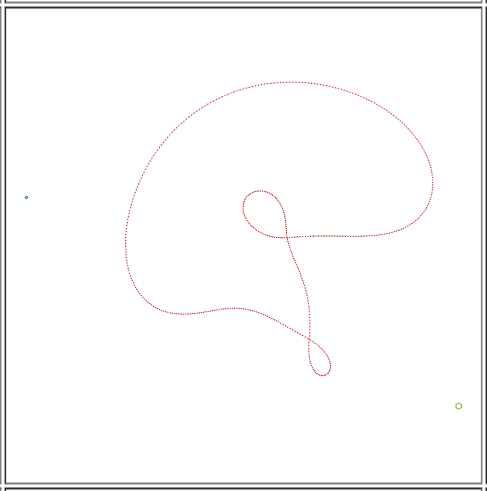	Finished circular assembly	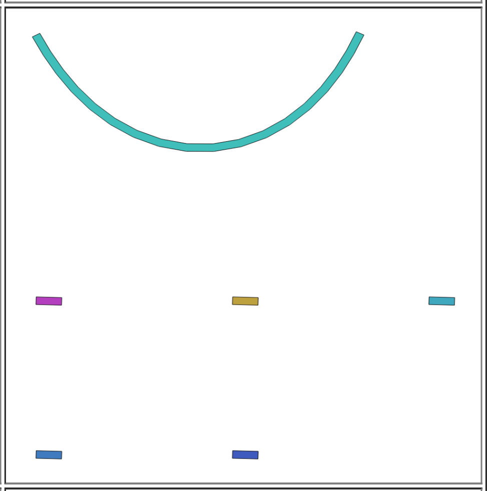
NCTC10217	*Yersinia pseudotuberculosis*	66	1	0	0	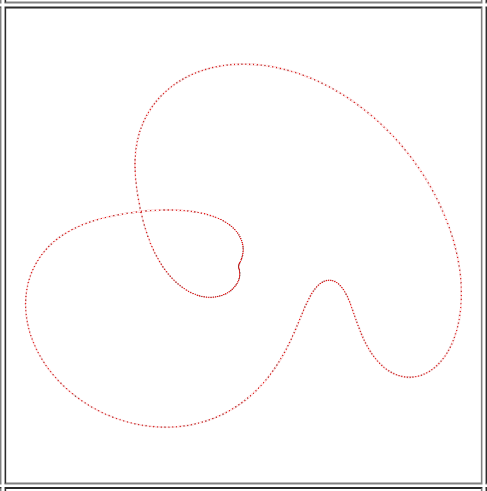	Finished circular assembly	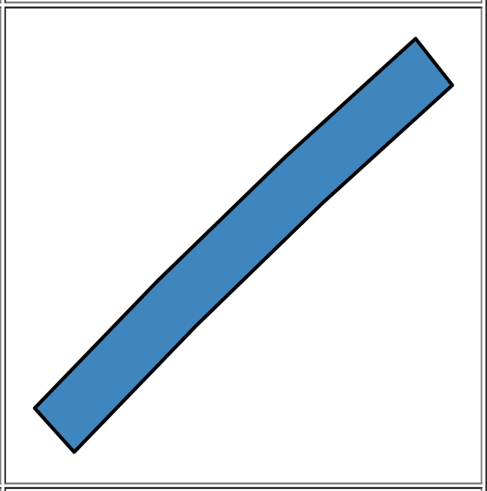
NCTC10221	*Actinobacillus ureae*	98	Pending	Pending	Pending	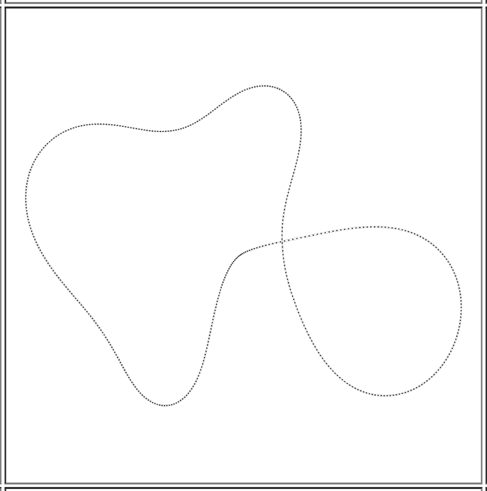	Finished circular assembly	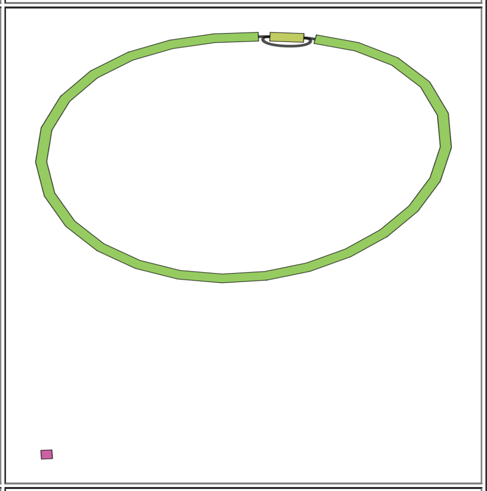
NCTC10232	*Streptococcus sp.*	95	Pending	Pending	Pending		Finished circular assembly	

						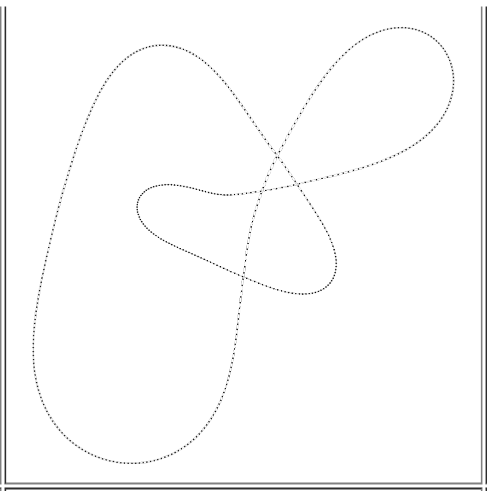		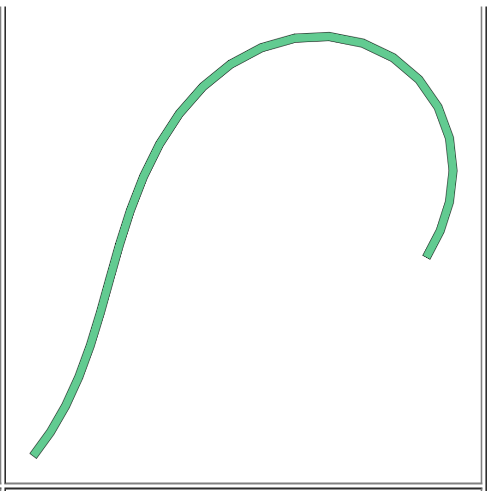
NCTC10237	*Streptococcus sp*.	33	Pending	Pending	Pending	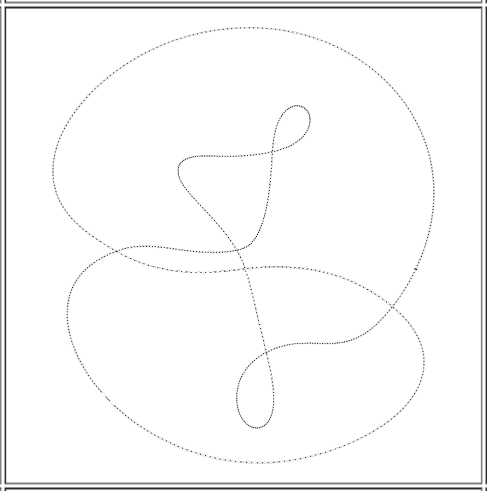	Finished circular assembly	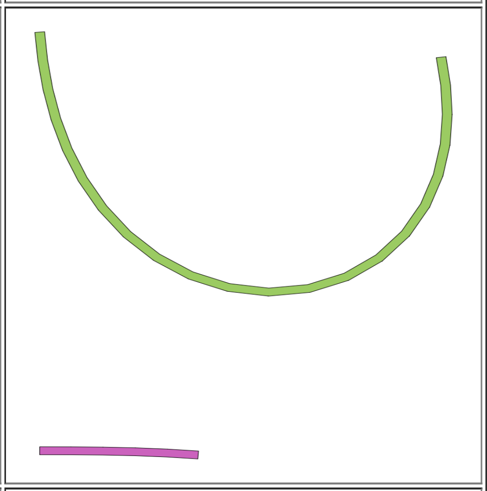
NCTC10238	*Streptococcus sp*.	31	Pending	Pending	Pending	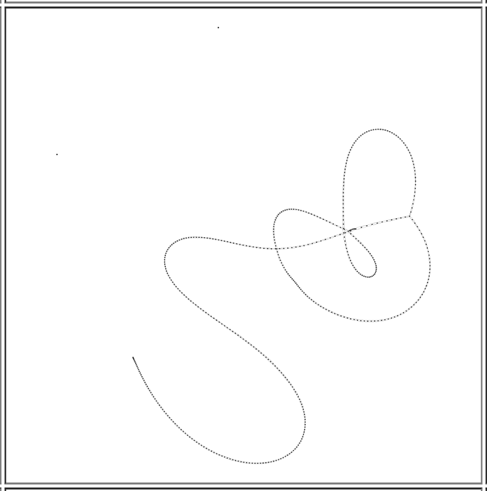	Mis-assembly/Fragmented	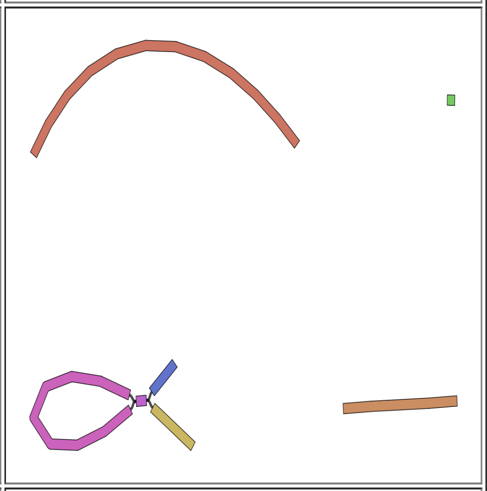
NCTC10246	*Klebsiella pneumoniae*	47	1	1	3	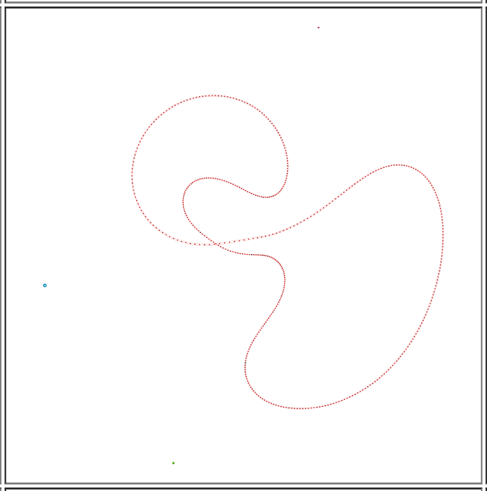	Finished circular assembly	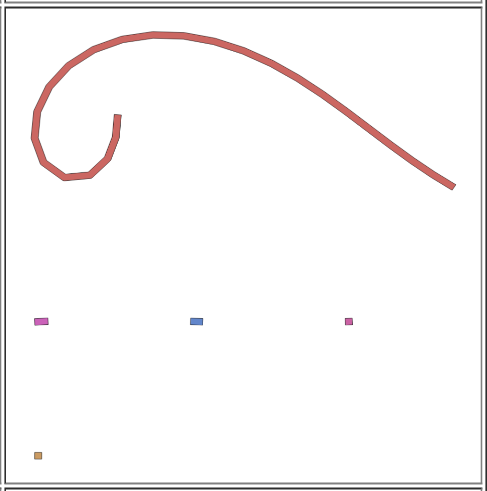
NCTC10261	*Klebsiella sp*.	36	Pending	Pending	Pending	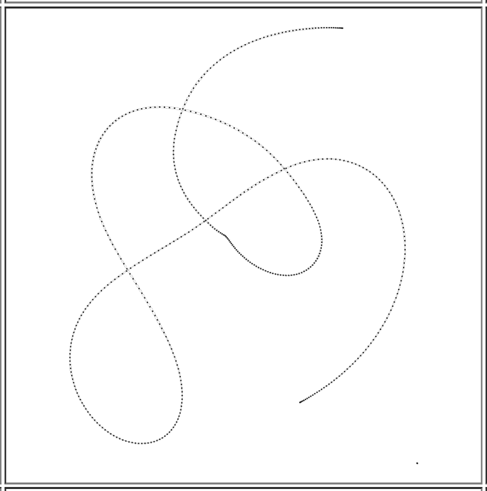	Finished assembly (lacking circularisation)	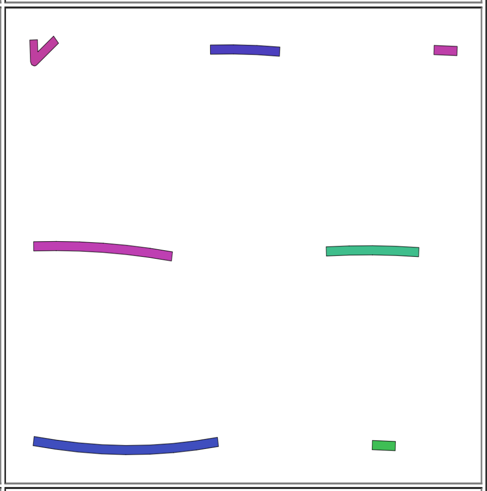
NCTC10262	*Klebsiella sp*.	80	1	2	0	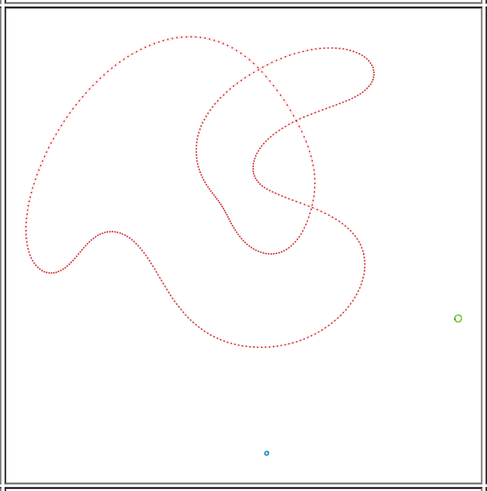	Finished circular assembly	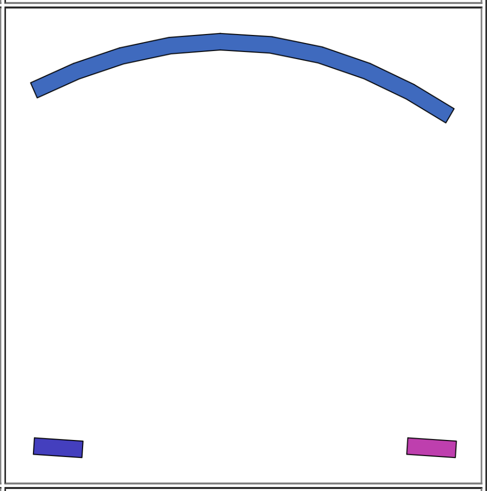
NCTC10263	*Klebsiella sp*.	54	0	0	7		Finished circular assembly	

						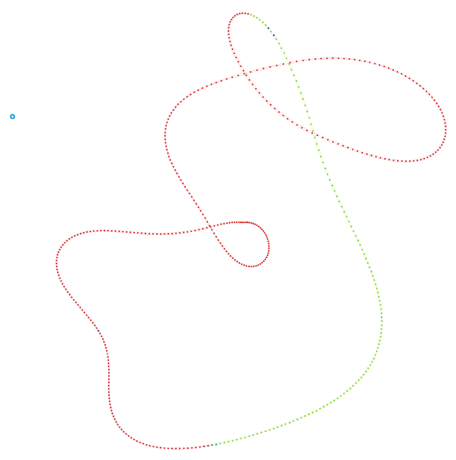		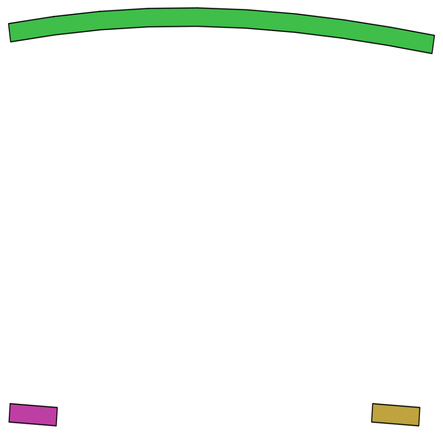
NCTC10286	*Providencia alcalifaciens*	41	1	0	0	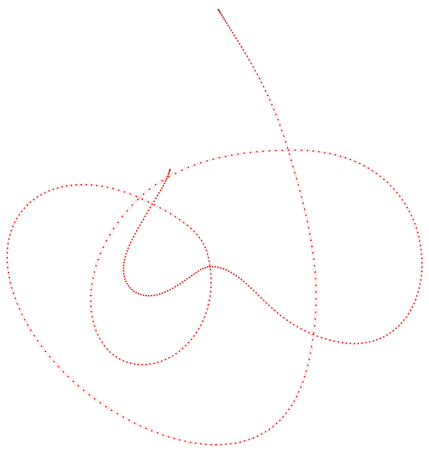	Finished assembly (lacking circularisation)	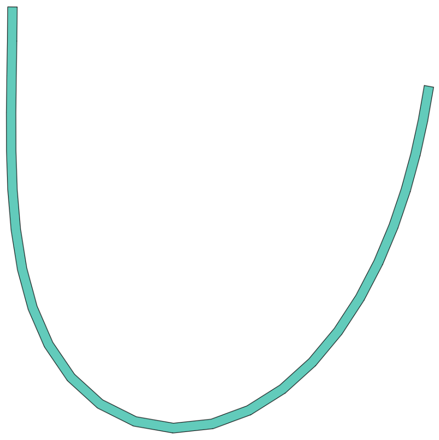
NCTC10313	*Klebsiella pneumoniae subsp. ozaenae*	40	1	3	2	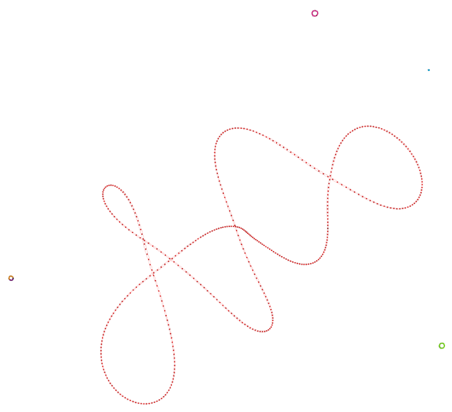	Finished circular assembly	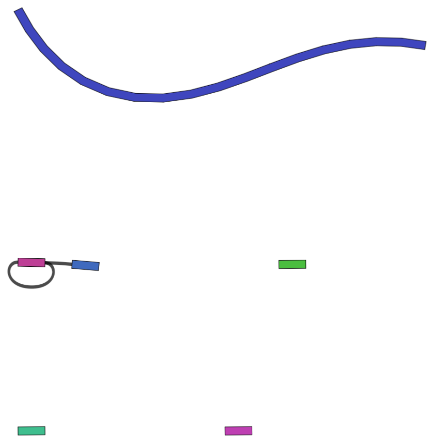
NCTC10314	*Klebsiella pneumoniae*	48	1	0	1	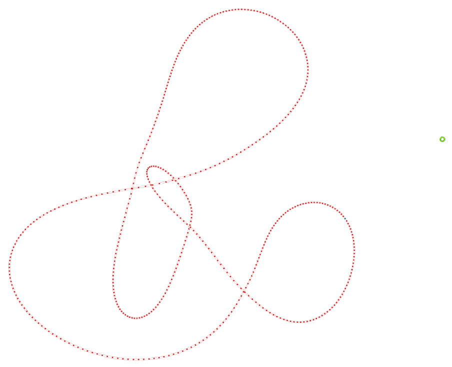	Finished circular assembly	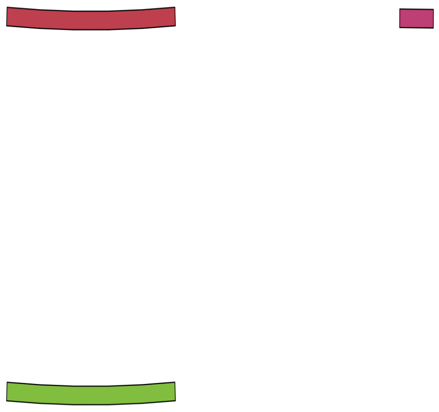
NCTC10317	*Enterobacter aerogenes*	58	Pending	Pending	Pending	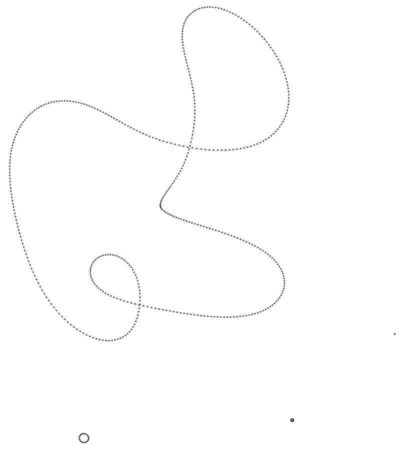	Finished circular assembly	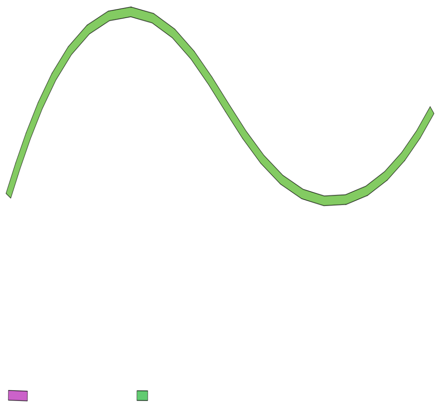
NCTC10321	*Streptococcus sp.*	70	0	0	2	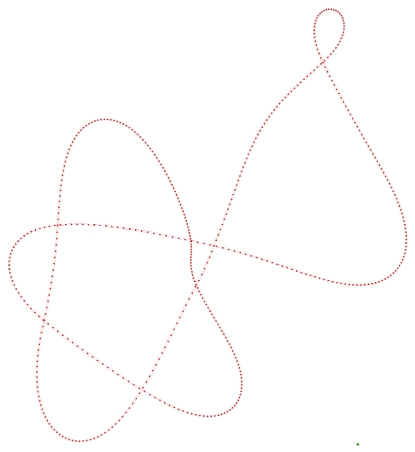	Finished circular assembly	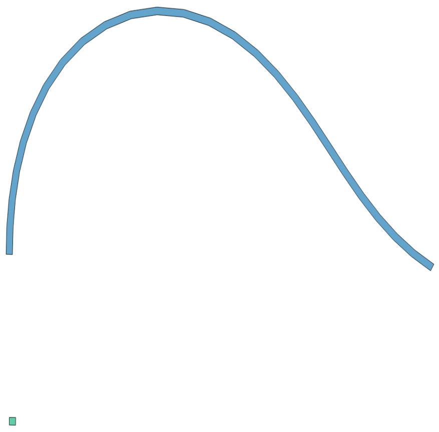
NCTC10332	*Pseudomonas aeruginosa*	36	1	0	0		Mis-assembly/Fragmented	

						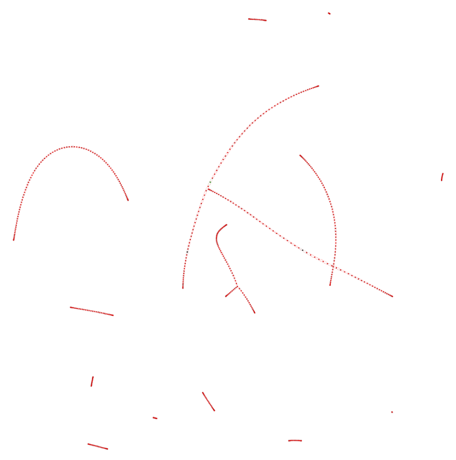		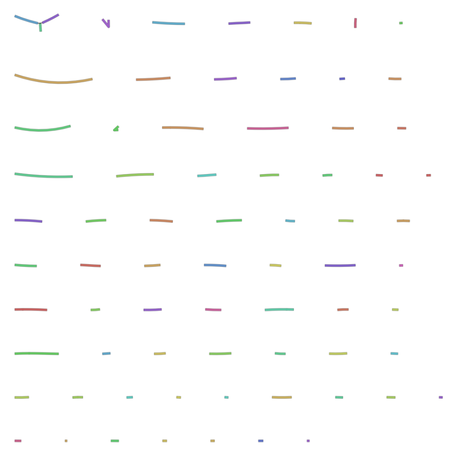
NCTC10344	*Staphylococcus aureus*	117	1	0	0	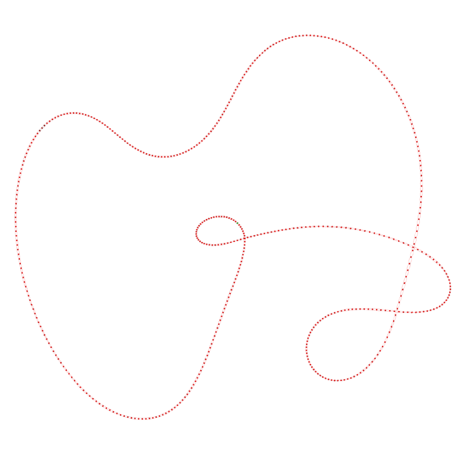	Finished circular assembly	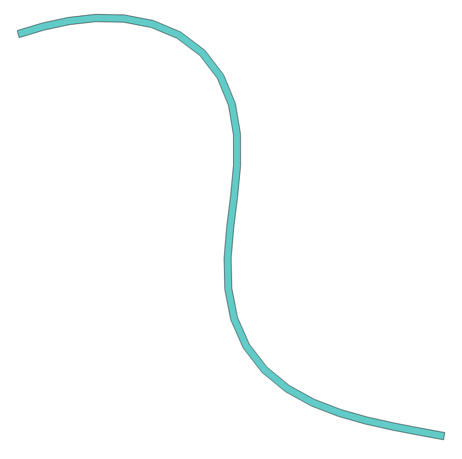
NCTC10345	*Staphylococcus aureus*	136	1	0	1	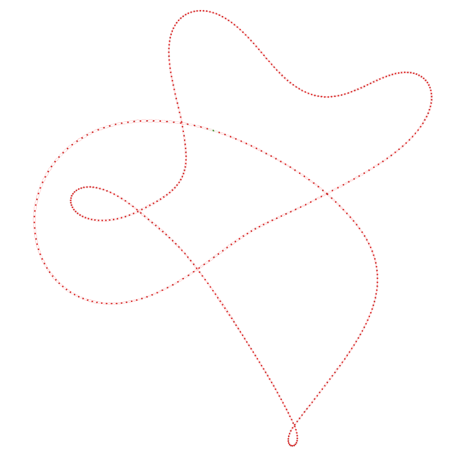	Finished circular assembly	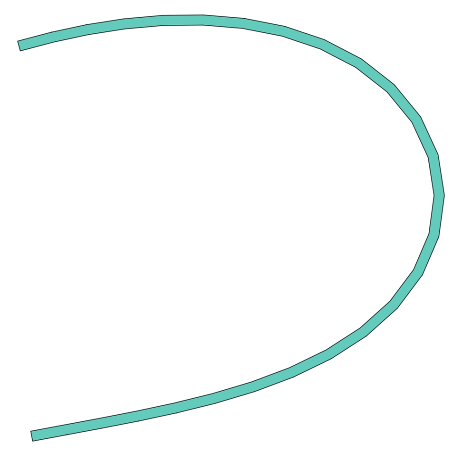
NCTC10350	*Staphylococcus hyicus*	121	Pending	Pending	Pending	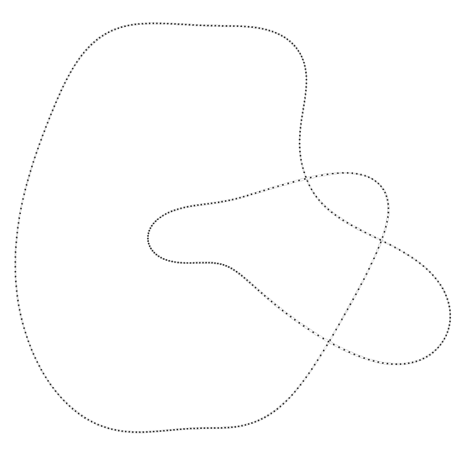	Finished circular assembly	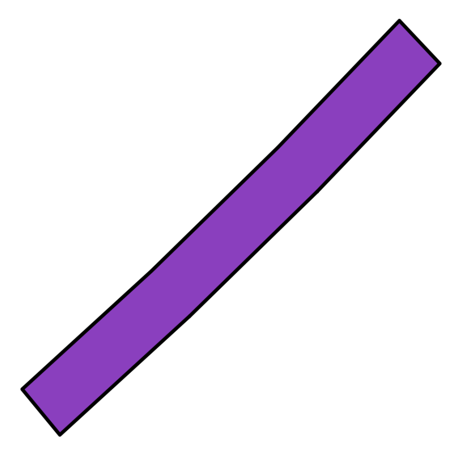
NCTC10353	*Streptococcus thermophilus*	160	Pending	Pending	Pending	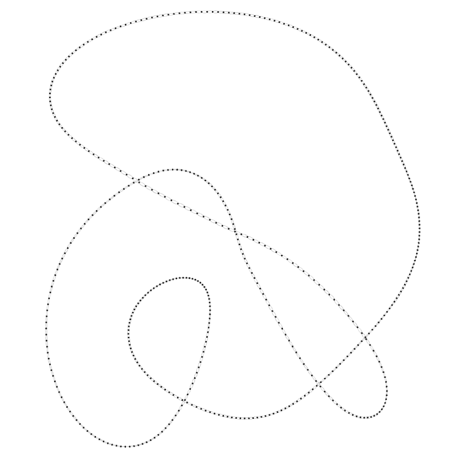	Finished circular assembly	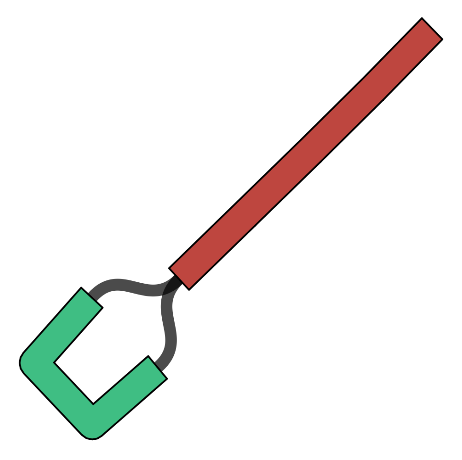
NCTC10360	*Plesiomonas shigelloides*	60	1	0	0	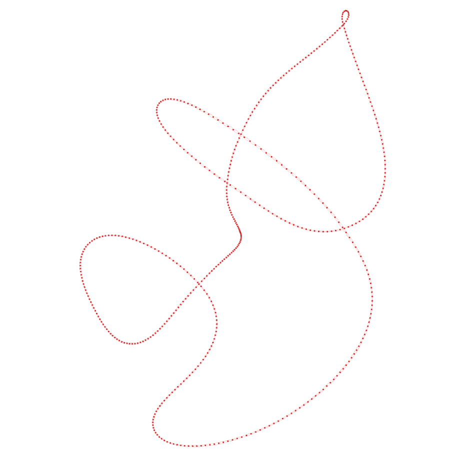	Finished circular assembly	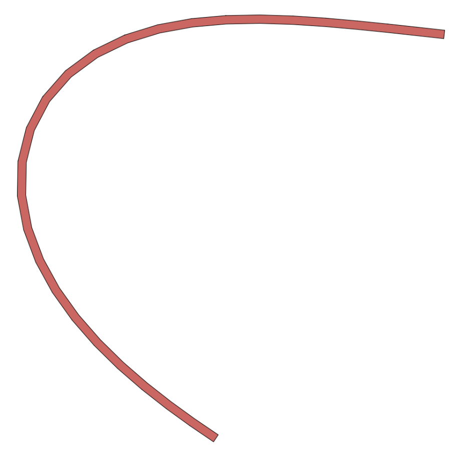
NCTC10361	*Escherichia coli*	65	0	0	5		Finished circular assembly	

						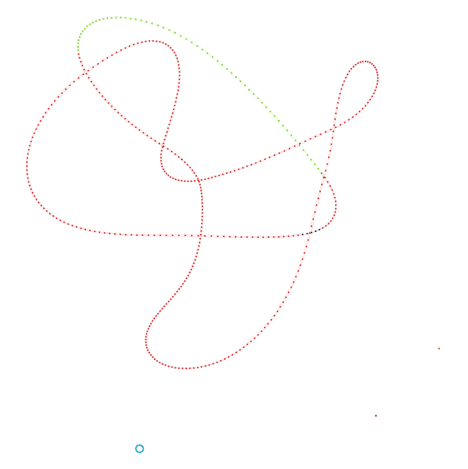		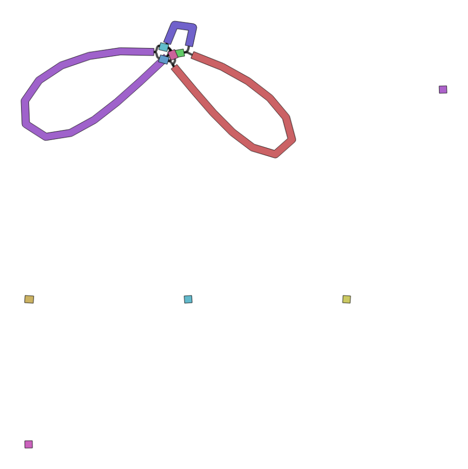
NCTC10382	*Pasteurella multocida*	95	1	0	0	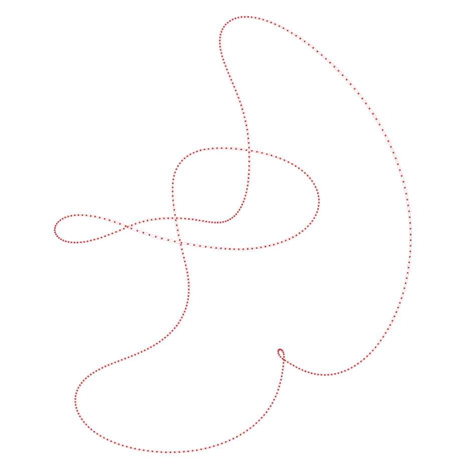	Finished circular assembly	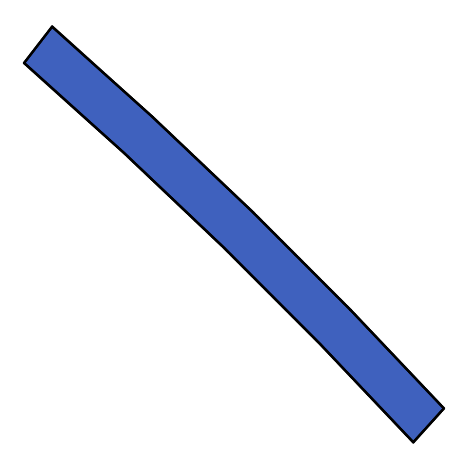
NCTC10384	*Salmonella enterica subsp. enterica serovar Senftenberg*	38	4	0	0	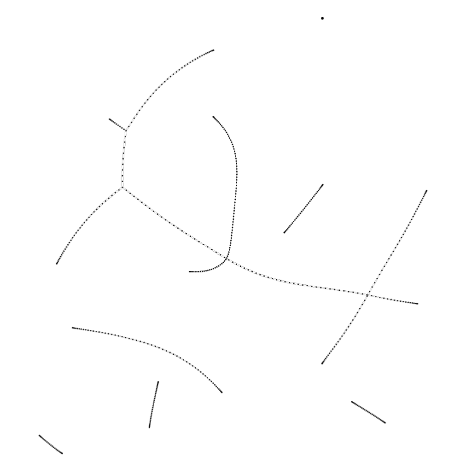	Mis-assembly/Fragmented	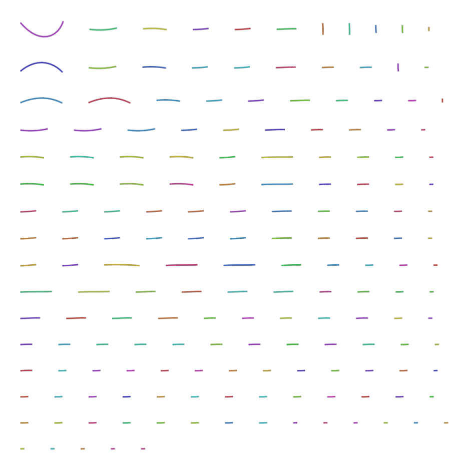
NCTC10396	*Edwardsiella tarda*	47	0	0	2	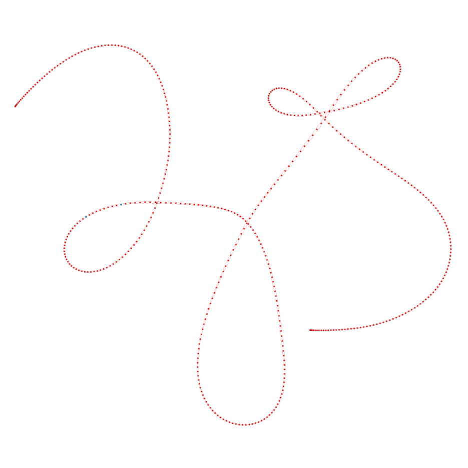	Finished assembly (lacking circularisation)	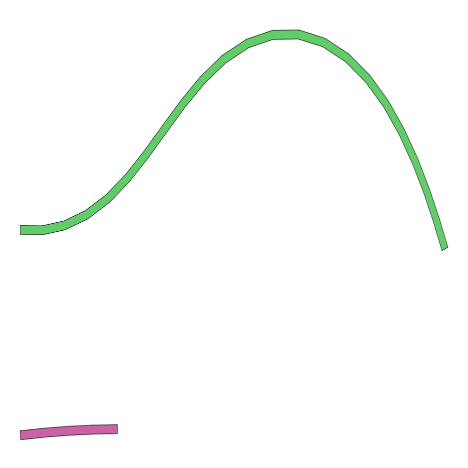
NCTC10399	*Staphylococcus aureus*	83	Pending	Pending	Pending	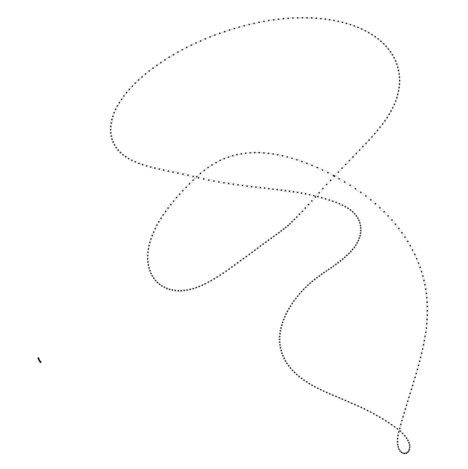	Finished circular assembly	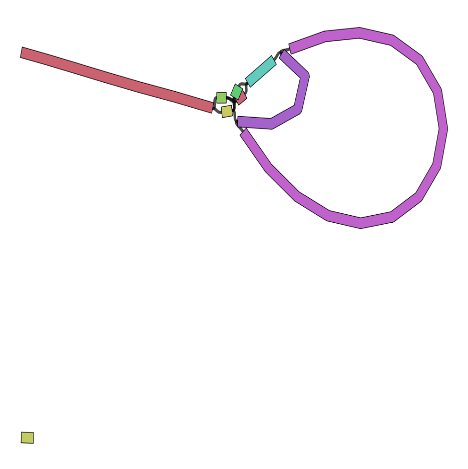
NCTC10418	*Escherichia coli*	45	1	1	0	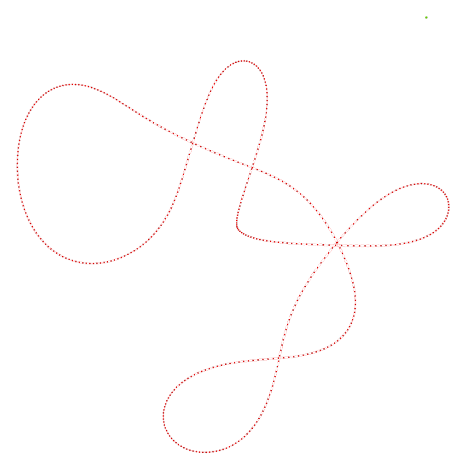	Finished circular assembly	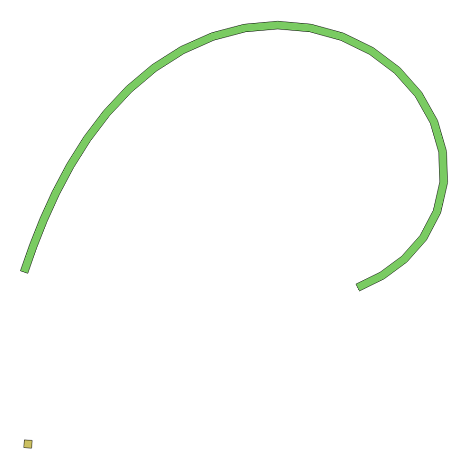
NCTC10429	*Escherichia coli*	21	0	0	7		Mis-assembly/Fragmented	

						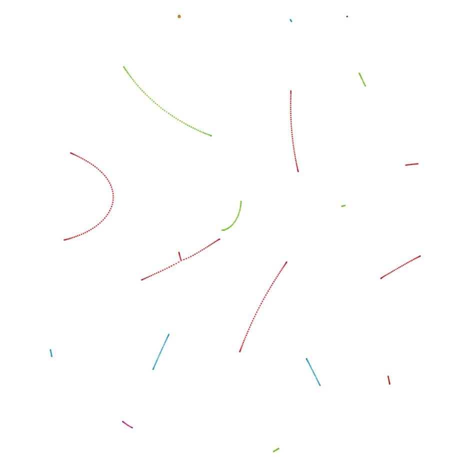		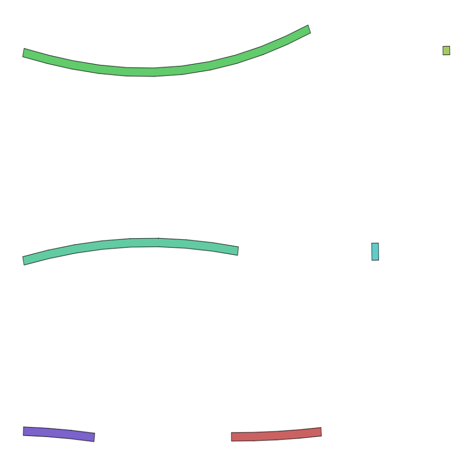
NCTC10430	*Escherichia coli*	39	0	0	1	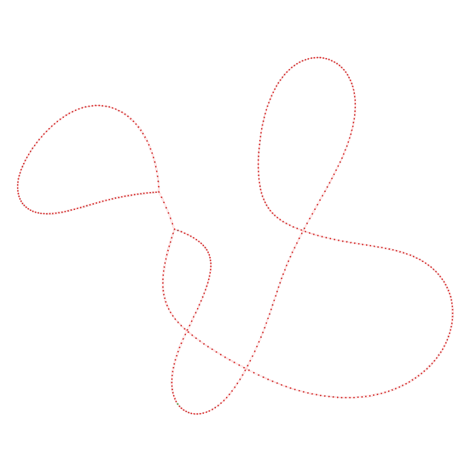	Mis-assembly/Fragmented	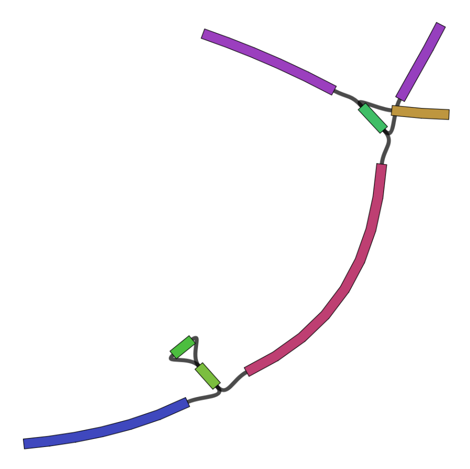
NCTC10442	*Staphylococcus aureus*	170	1	0	2	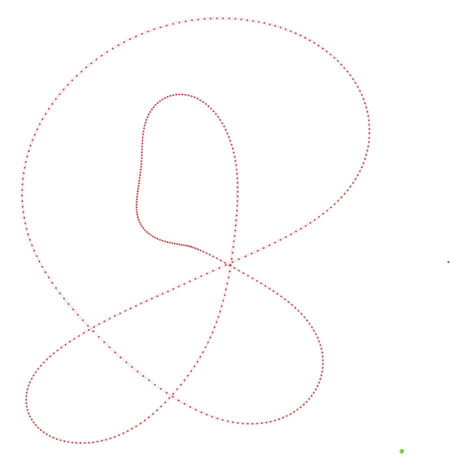	Finished circular assembly	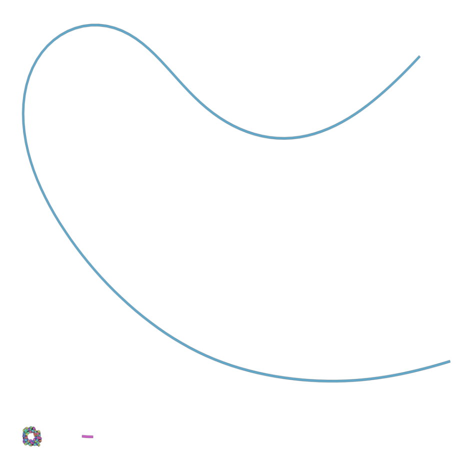
NCTC10443	*Staphylococcus aureus*	140	1	0	1	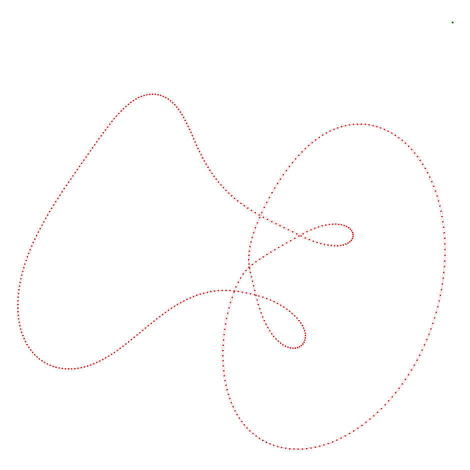	Finished circular assembly	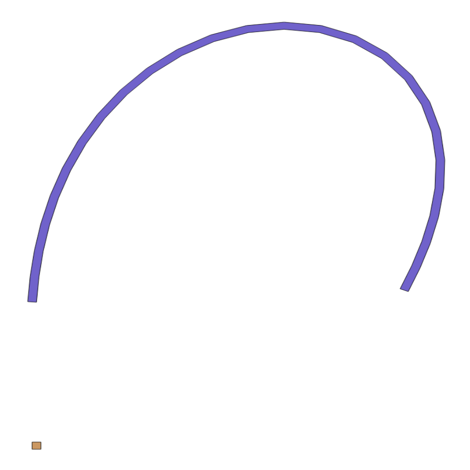
NCTC10444	*Escherichia coli*	61	1	0	0	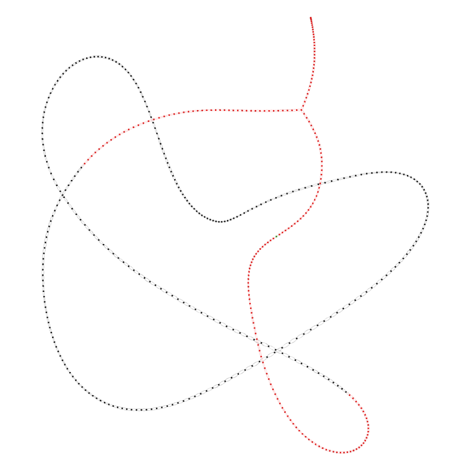	Mis-assembly/Fragmented	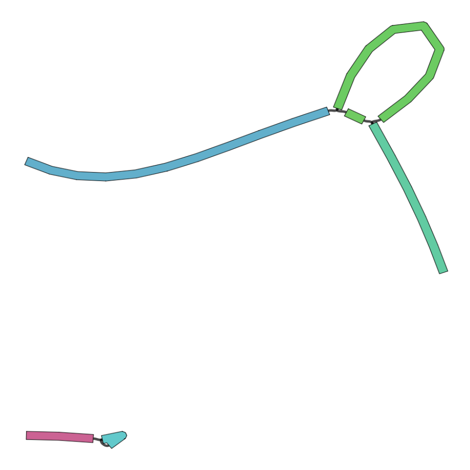
NCTC10449	*Streptococcus mutans*	160	Pending	Pending	Pending	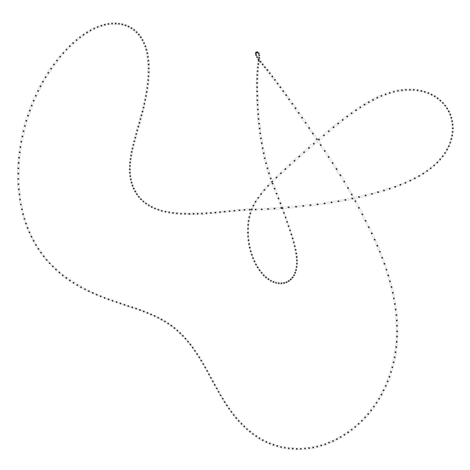	Finished circular assembly	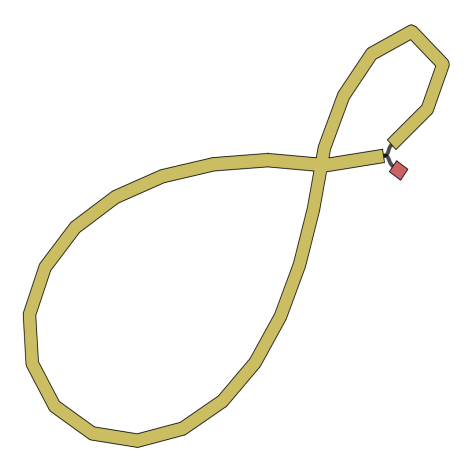
NCTC10460	*Yersinia enterocolitica*	0	Pending	Pending	Pending		Mis-assembly/Fragmented	

						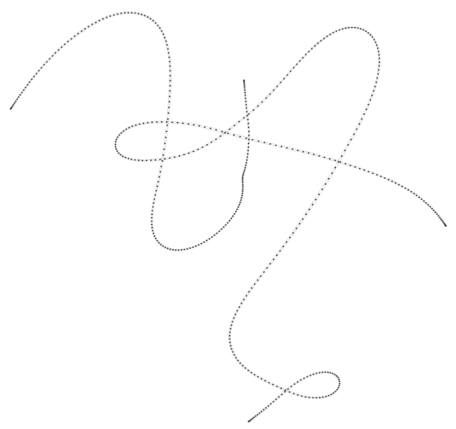		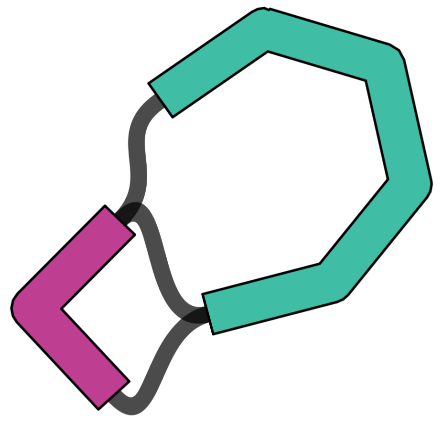	
NCTC10487	*Escherichia coli*	58	1	4	1	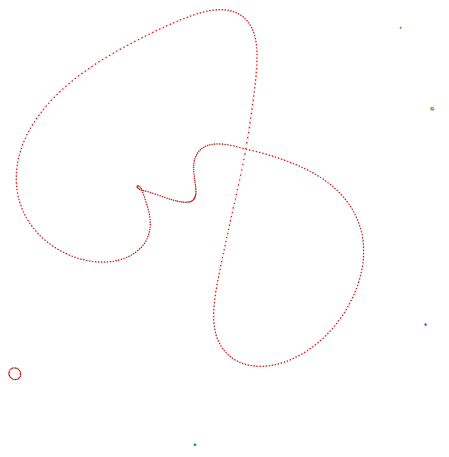	Finished circular assembly	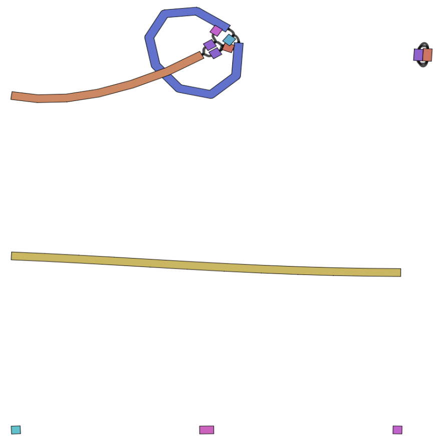	
NCTC10517	*Staphylococcus xylosus*	100	0	0	89	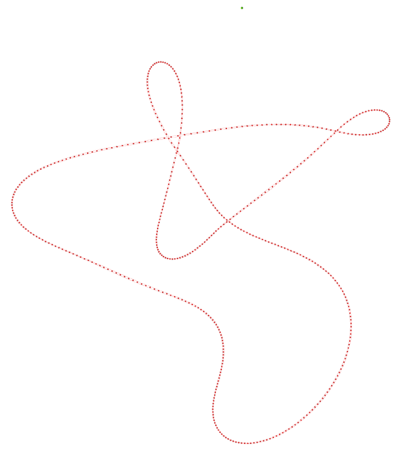	Finished circular assembly	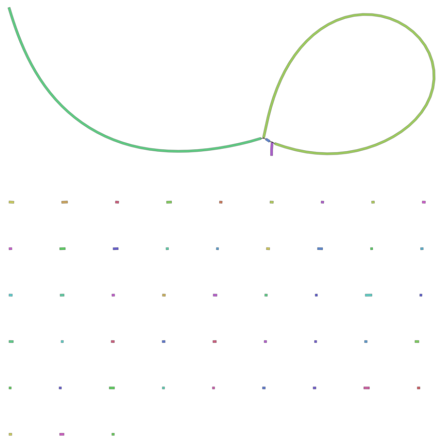	
NCTC10518	*Staphylococcus warneri*	119	1	0	1	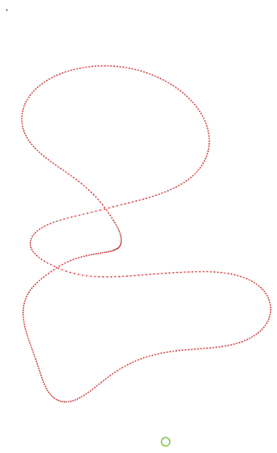	Finished circular assembly	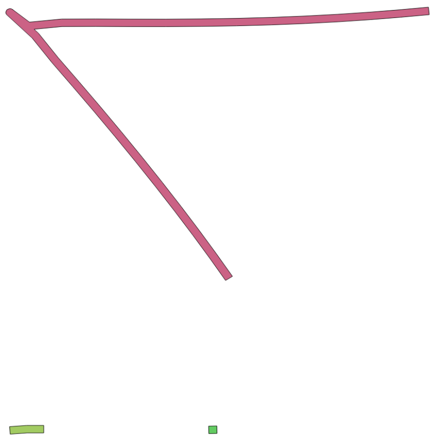	
NCTC10530	*Staphylococcus chromogenes*	121	Pending	Pending	Pending	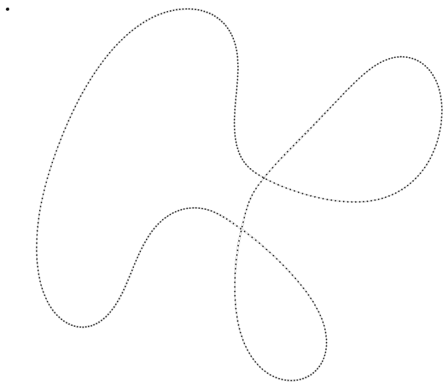	Finished circular assembly	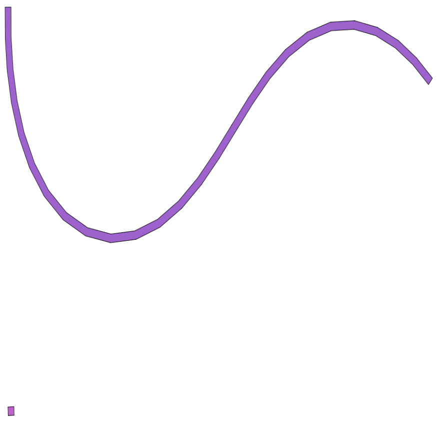	
NCTC10535	*Pasteurella bettyae*	80	0	0	2	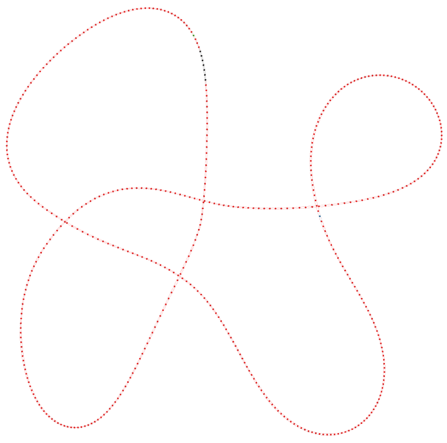	Finished circular assembly	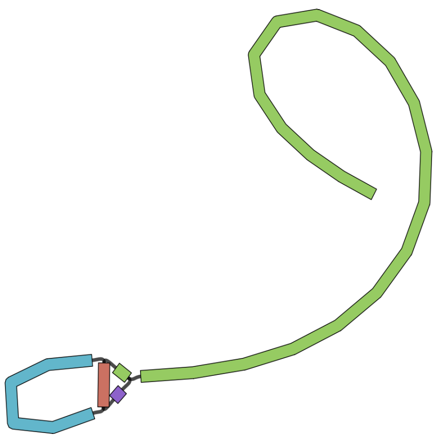	
NCTC10537	*Escherichia coli*	46	1	0	0		Finished assembly (lacking circularisation)		

						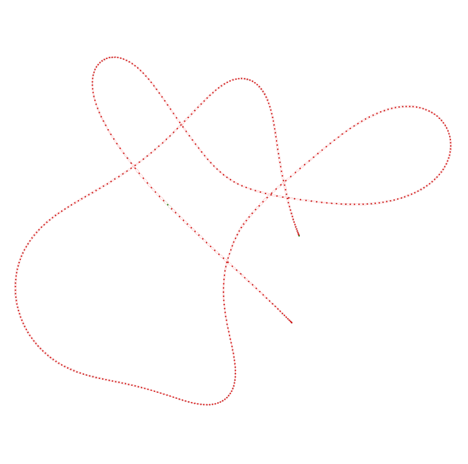		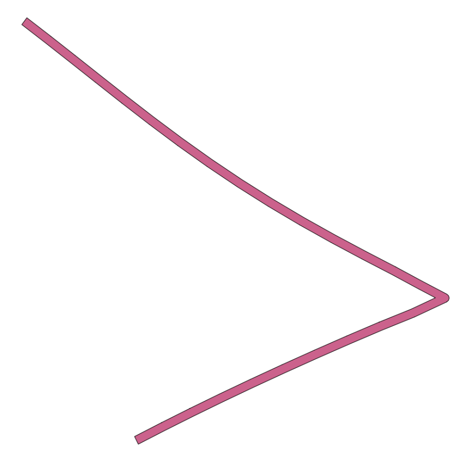
NCTC10538	*Escherichia coli*	58	1	0	1	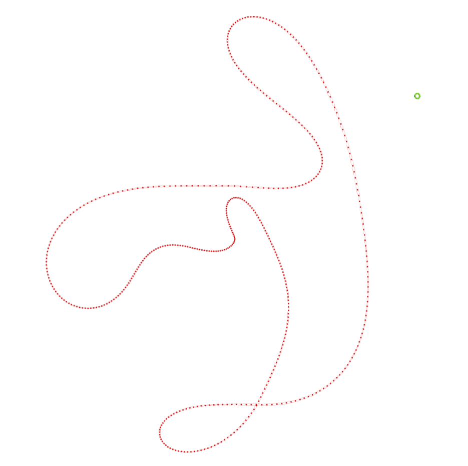	Finished circular assembly	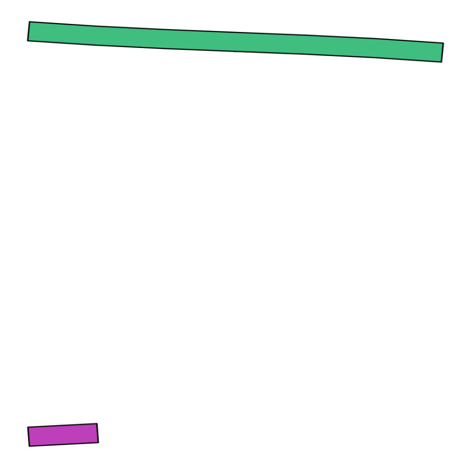
NCTC10562	*Fusobacterium nucleatum subsp. polymorphum*	64	1	1	0	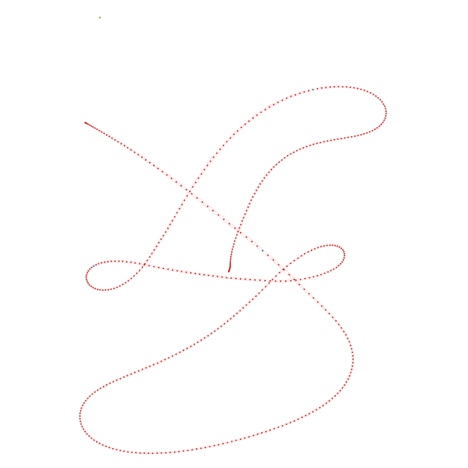	Finished assembly (lacking circularisation)	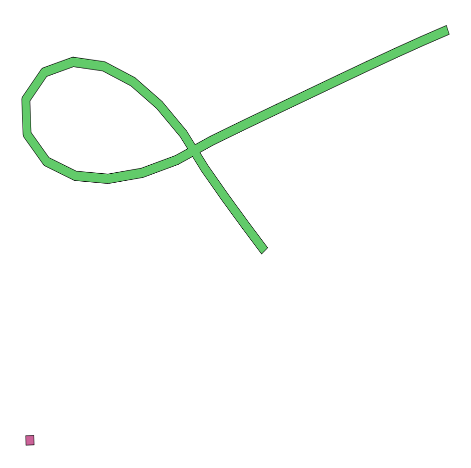
NCTC10568	*Actinobacillus lignieresii*	69	1	0	0	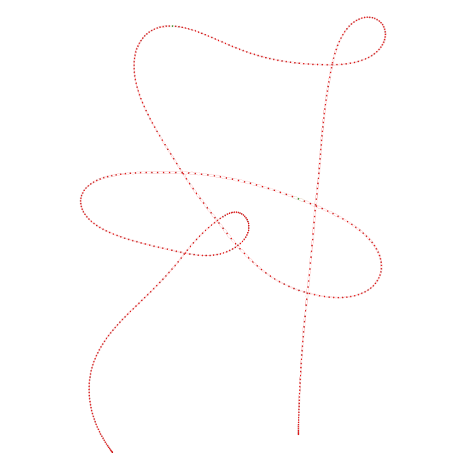	Finished assembly (lacking circularisation)	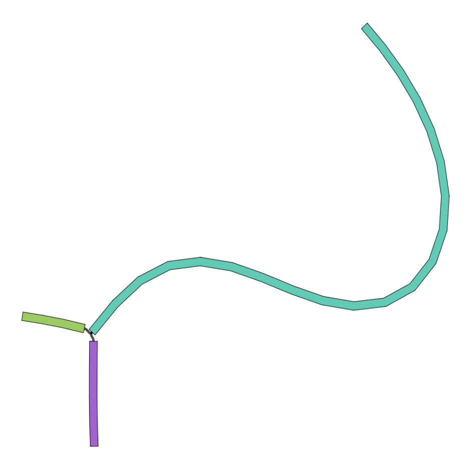
NCTC10588	*Elizabethkingia meningoseptica*	52	1	0	2	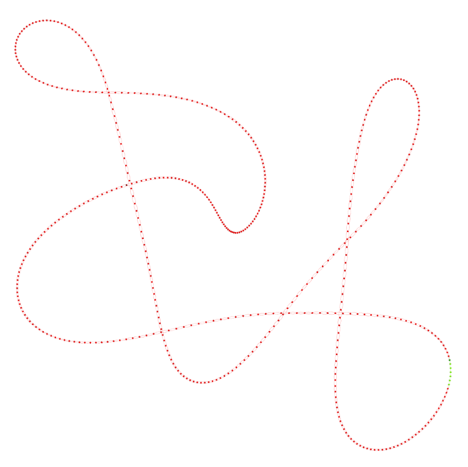	Finished circular assembly	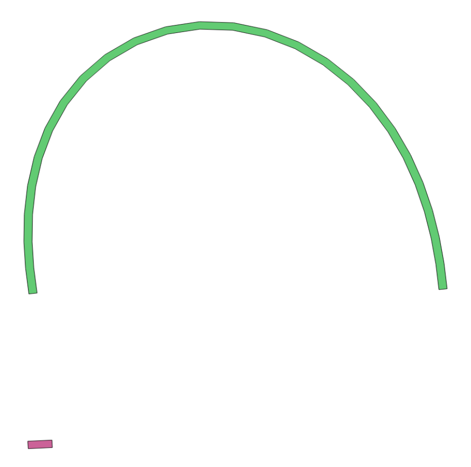
NCTC10609	*Mannheimia haemolytica*	34	1	0	0	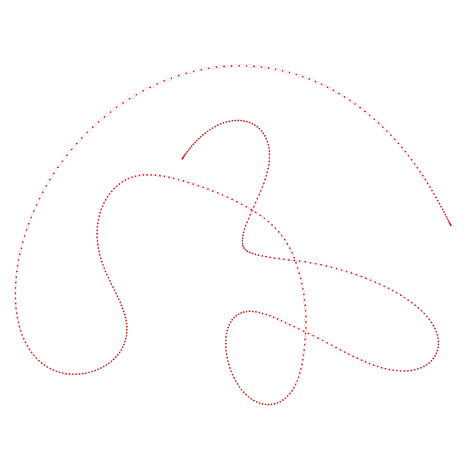	Finished assembly (lacking circularisation)	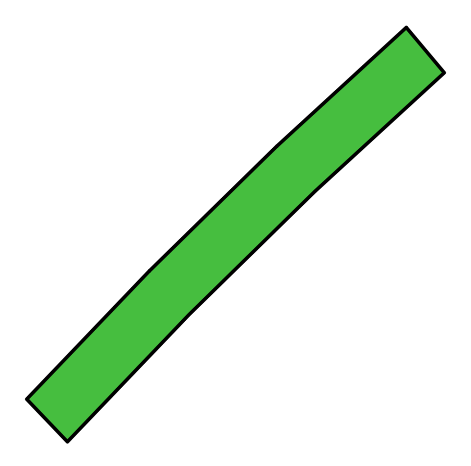
NCTC10618	*Neisseria lactamica*	141	1	0	1		Finished circular assembly	

						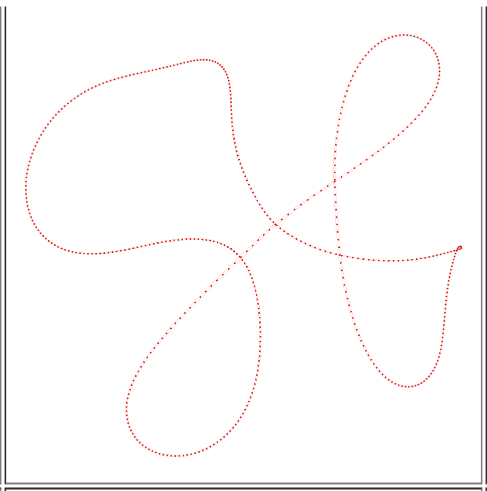		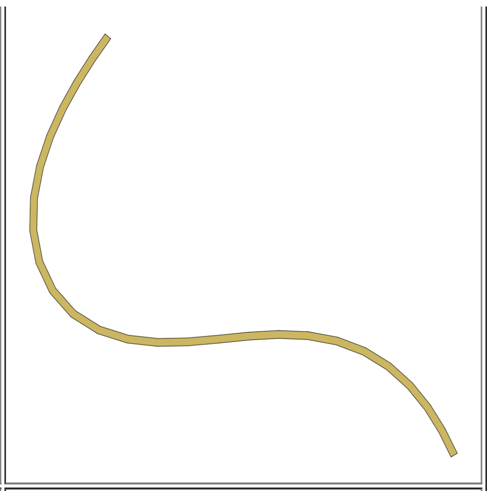
NCTC10638	*Mannheimia haemolytica*	41	1	0	1	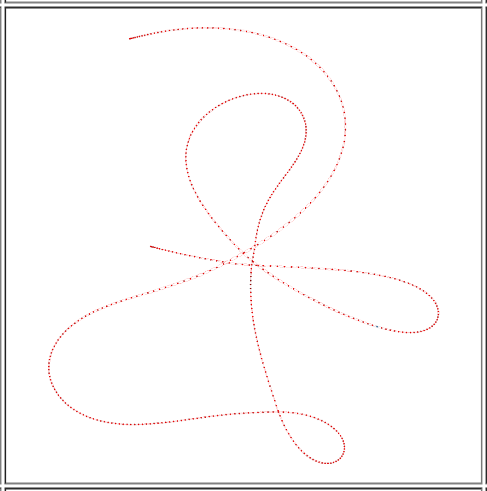	Finished assembly (lacking circularisation)	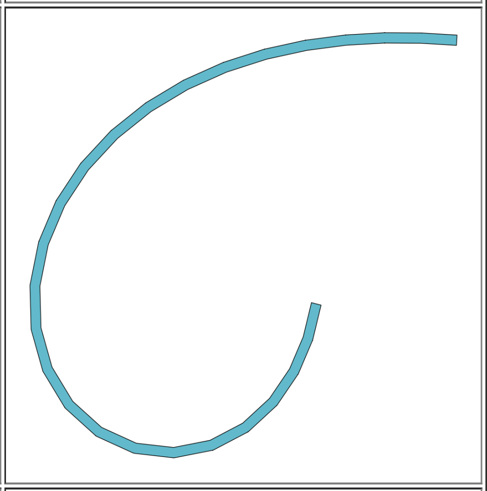
NCTC10650	*Escherichia coli*	107	0	0	11	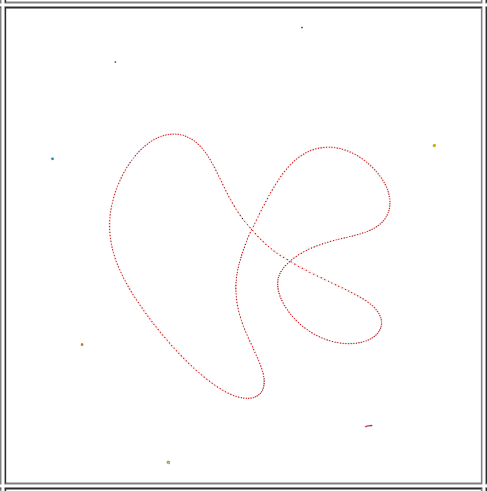	Finished circular assembly	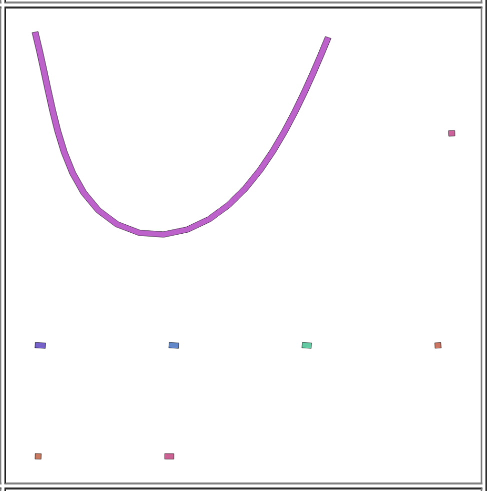
NCTC10652	*Staphylococcus aureus*	96	1	1	0	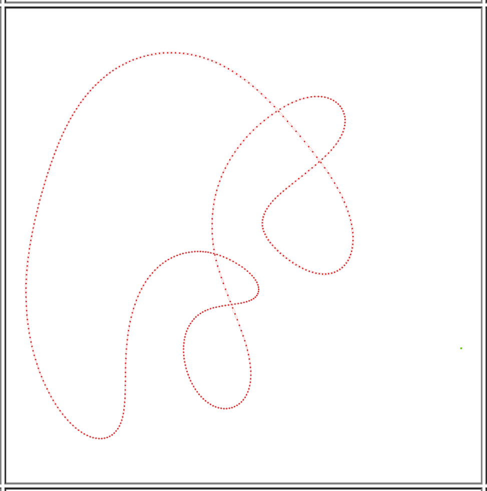	Finished circular assembly	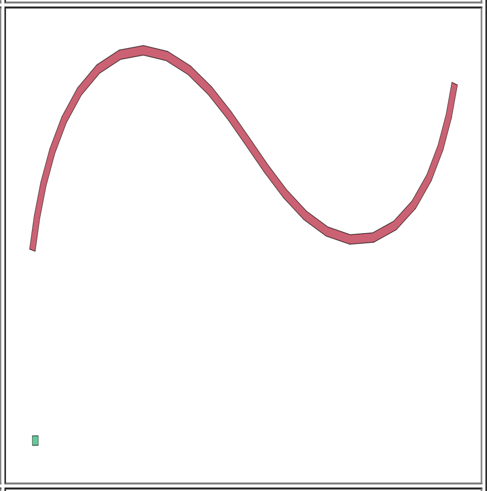
NCTC10654	*Staphylococcus aureus*	96	1	1	2	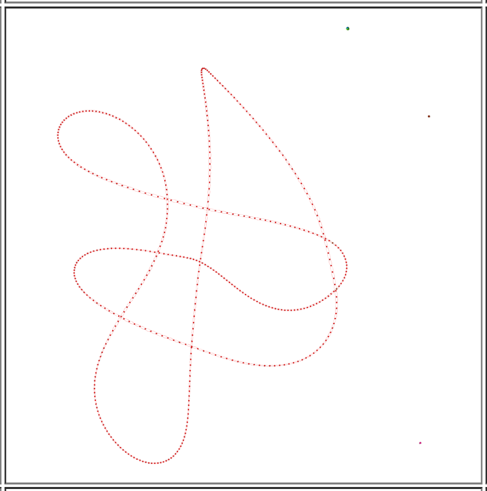	Finished circular assembly	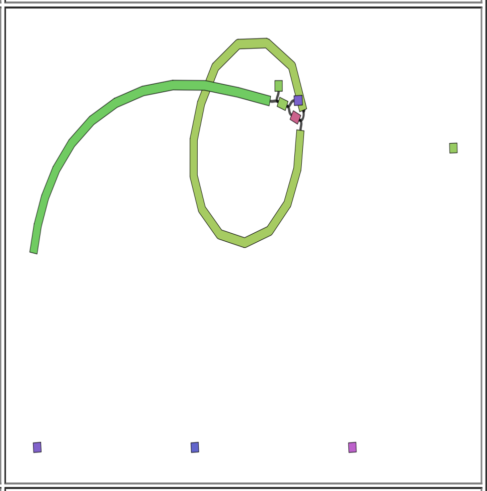
NCTC10655	*Staphylococcus aureus*	203	Pending	Pending	Pending	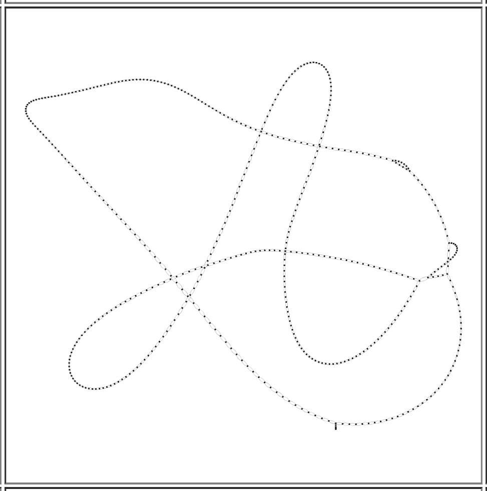	Mis-assembly/Fragmented	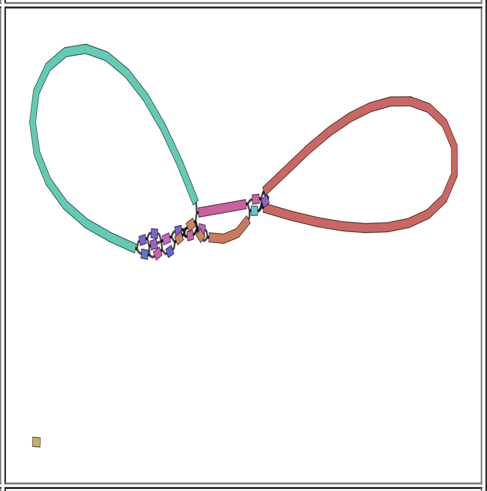
NCTC10656	*Staphylococcus aureus*	48	1	1	0		Finished circular assembly	

						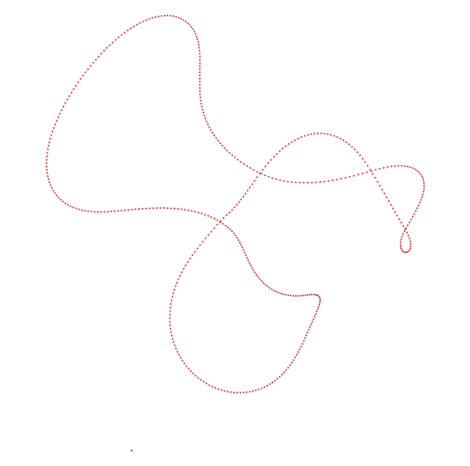		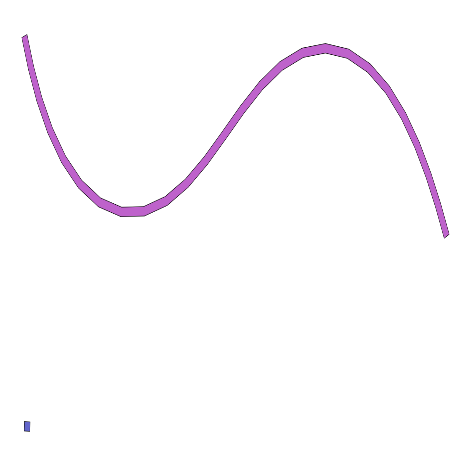
NCTC10657	*Staphylococcus aureus*	132	1	1	0	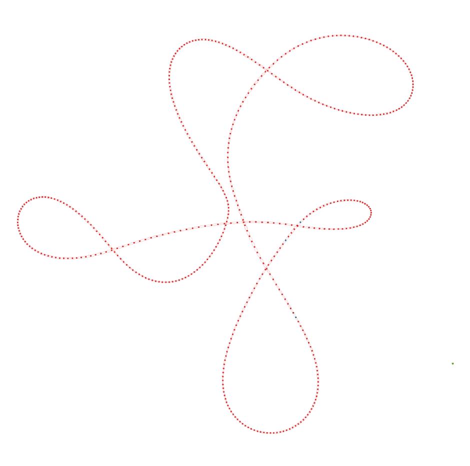	Finished circular assembly	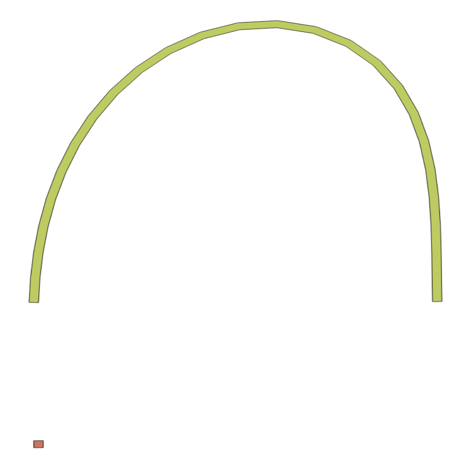
NCTC10662	*Pseudomonas aeruginosa*	37	0	0	3	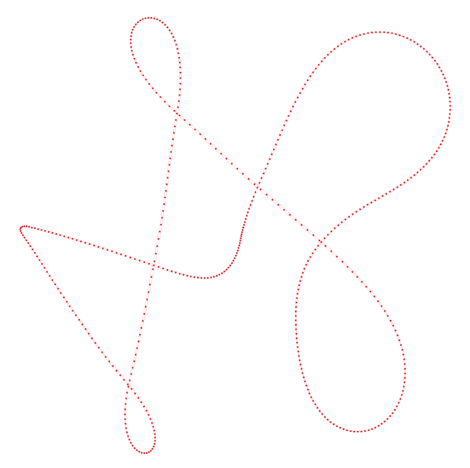	Finished circular assembly	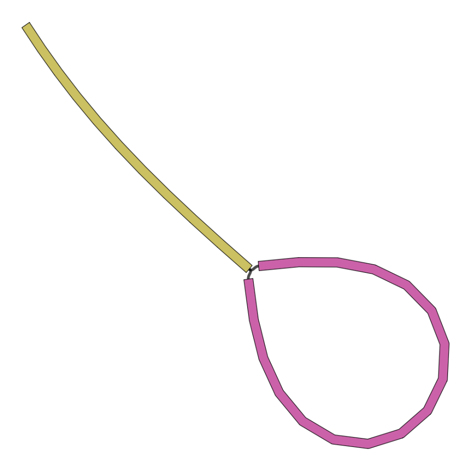
NCTC10664	*Escherichia coli*	71	0	0	5	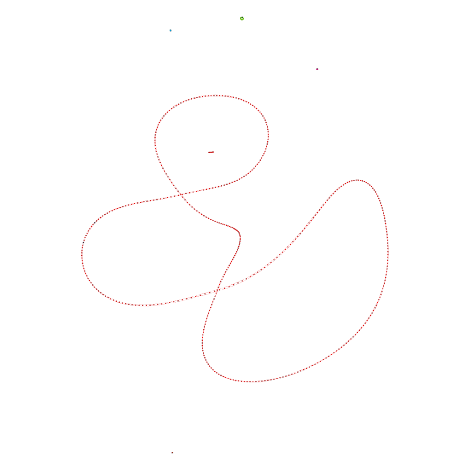	Finished circular assembly	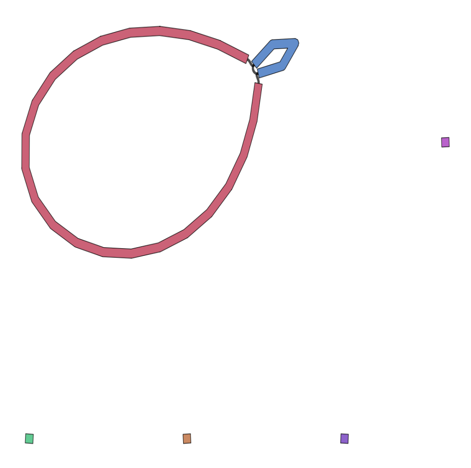
NCTC10672	*Haemophilus parainfluenzae*	124	0	0	2	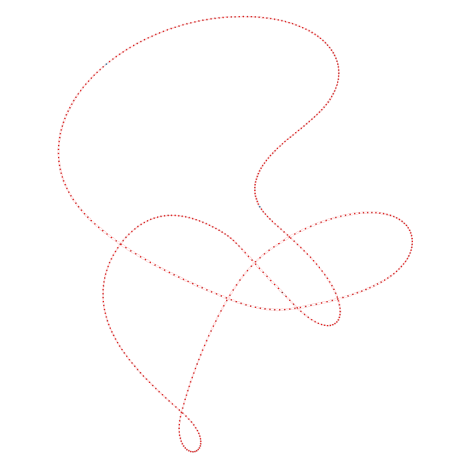	Finished circular assembly	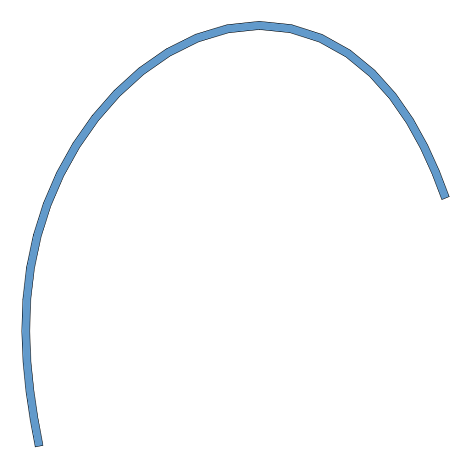
NCTC10674	*Escherichia coli*	78	1	1	3	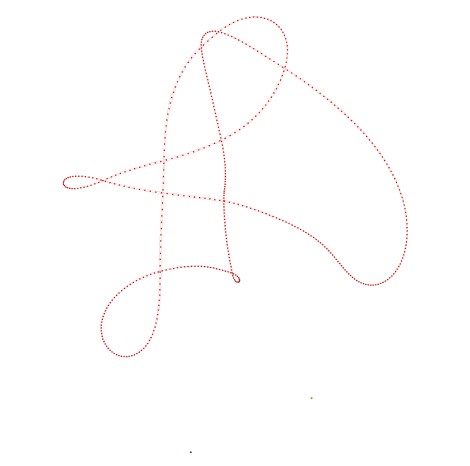	Finished circular assembly	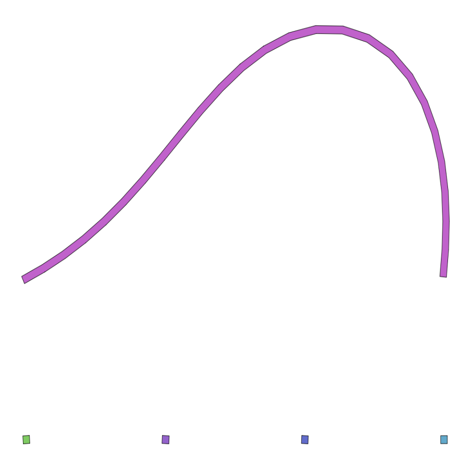
NCTC10702	*Staphylococcus aureus*	24	1	1	1		Mis-assembly/Fragmented	

						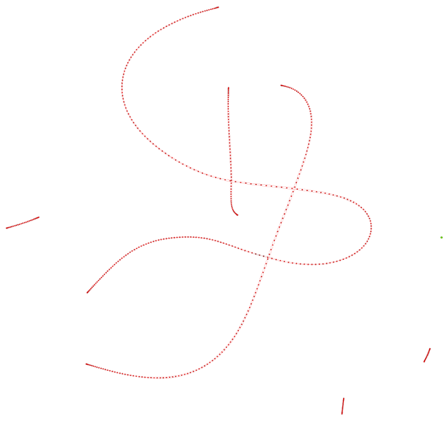		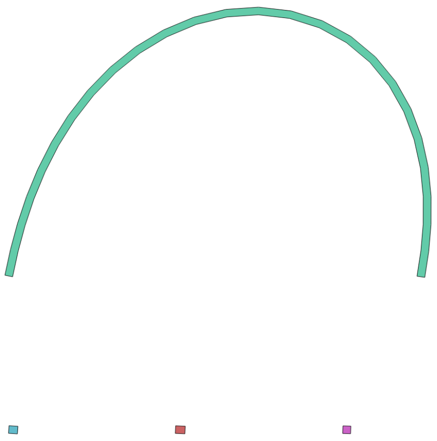
NCTC10703	*Staphylococcus aureus*	95	1	1	1	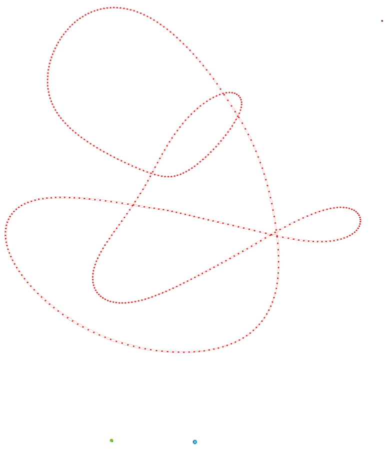	Finished circular assembly	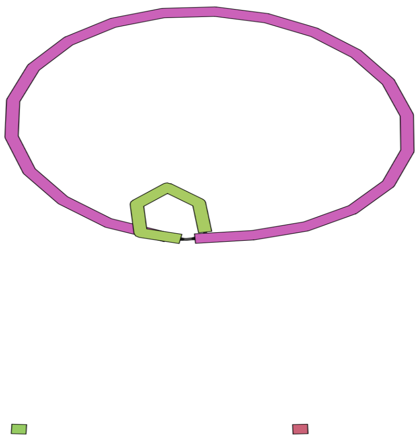
NCTC10705	*Salmonella enterica*	41	1	2	1	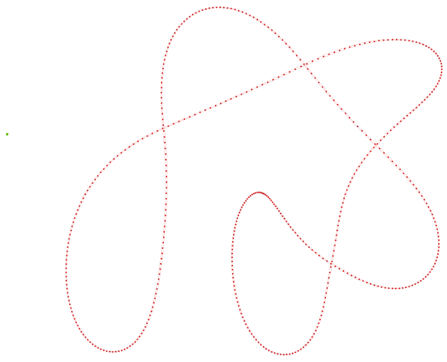	Finished circular assembly	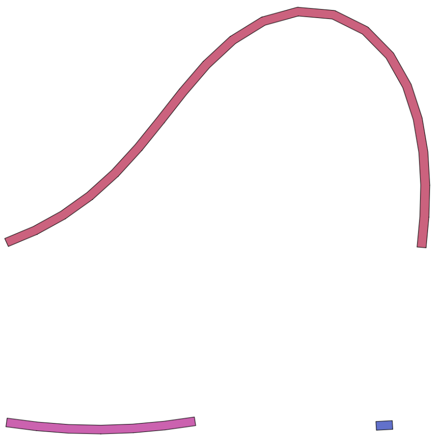
NCTC10707	*Streptococcus sp*.	67	Pending	Pending	Pending	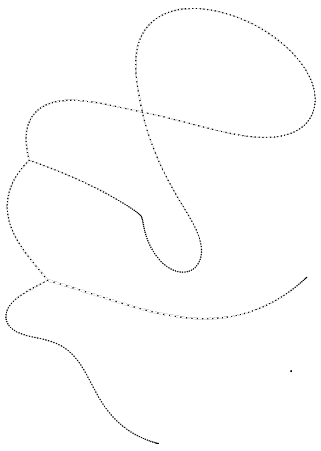	Mis-assembly/Fragmented	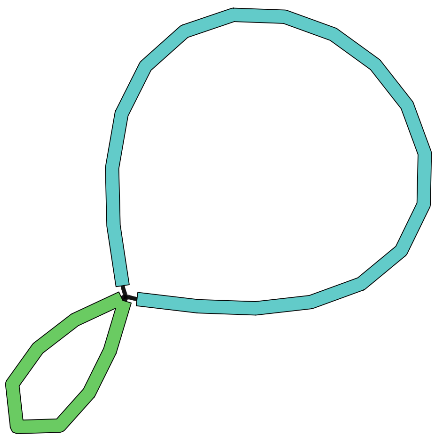
NCTC10710	*Streptococcus sp*	190	1	0	0	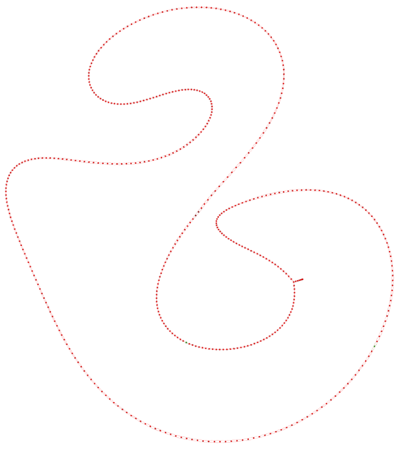	Finished circular assembly	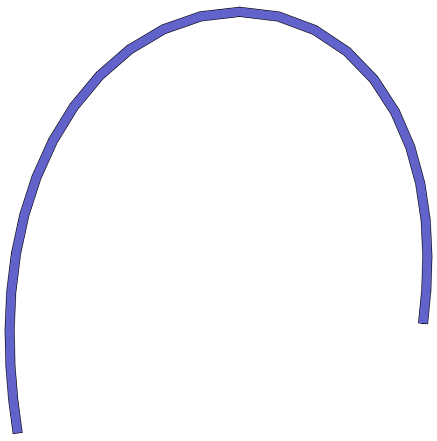
NCTC10713	*Streptococcus anginosus*	177	Pending	Pending	Pending	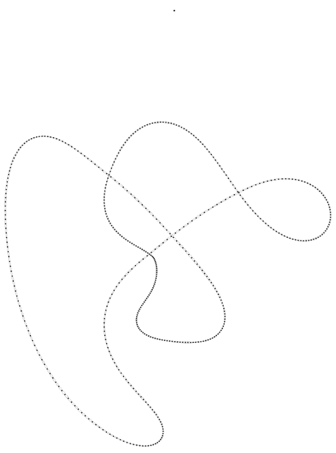	Finished circular assembly	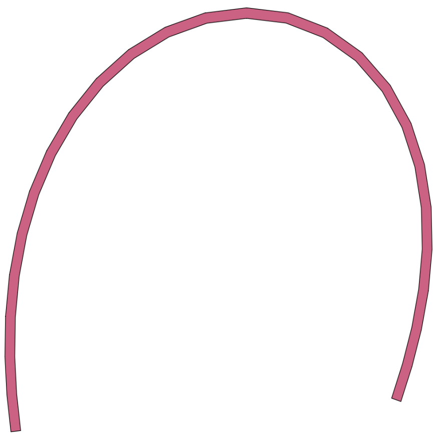
NCTC10722	*Pasteurella multocida*	129	1	1	0		Finished circular assembly	

						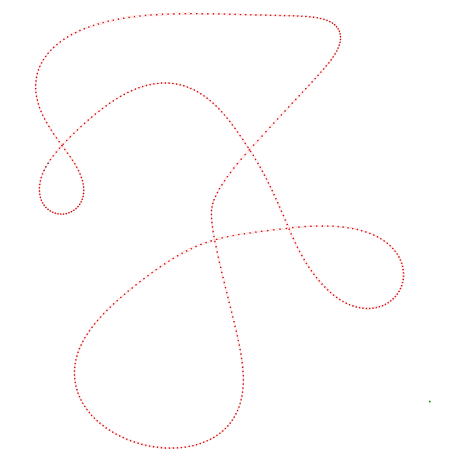		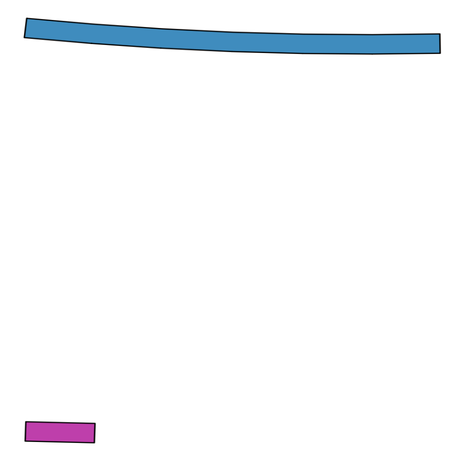
NCTC10723	*Fusobacterium necrogenes*	92	Pending	Pending	Pending	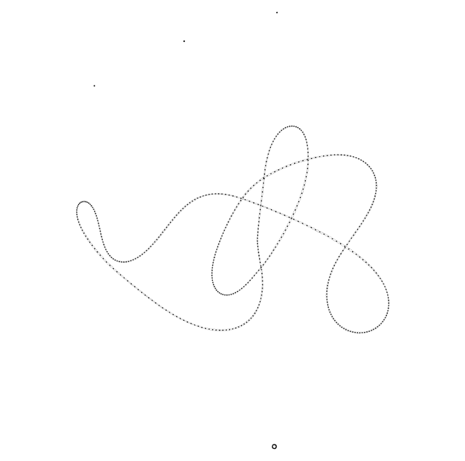	Finished circular assembly	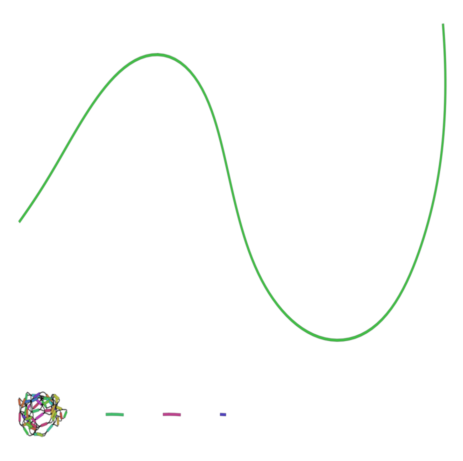
NCTC10724	*Staphylococcus aureus*	34	1	1	4	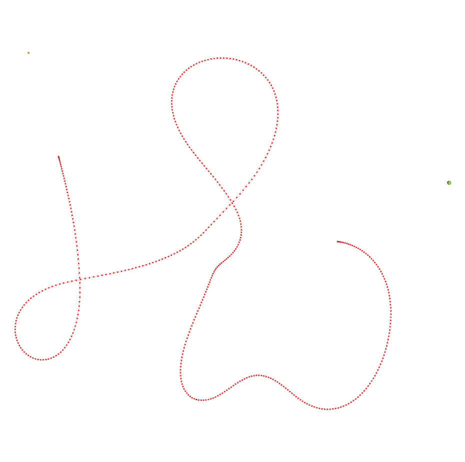	Finished assembly (lacking circularisation)	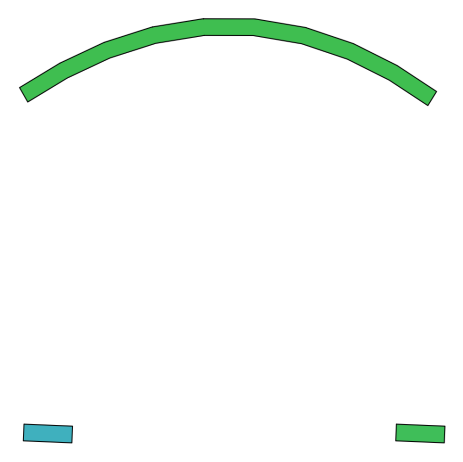
NCTC10757	*Escherichia coli*	27	0	0	6	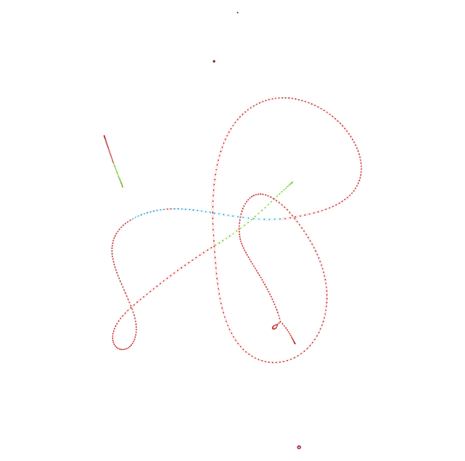	Mis-assembly/Fragmented	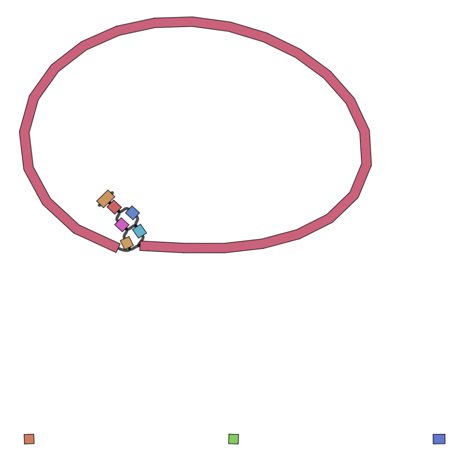
NCTC10758	*Escherichia coli*	35	1	0	5	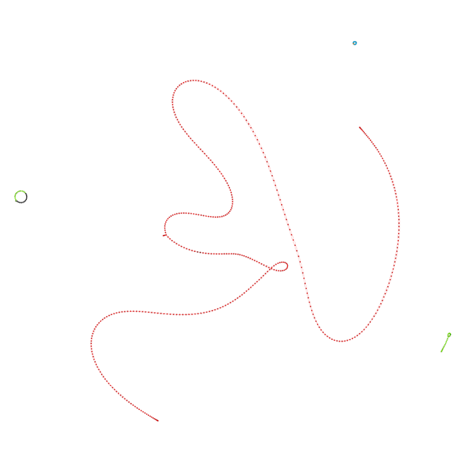	Finished assembly (lacking circularisation)	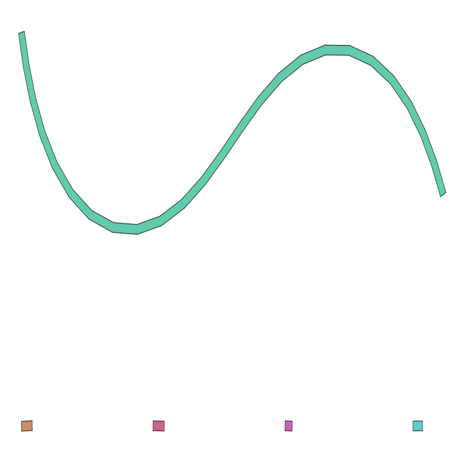
NCTC10764	*Escherichia coli*	47	0	0	8	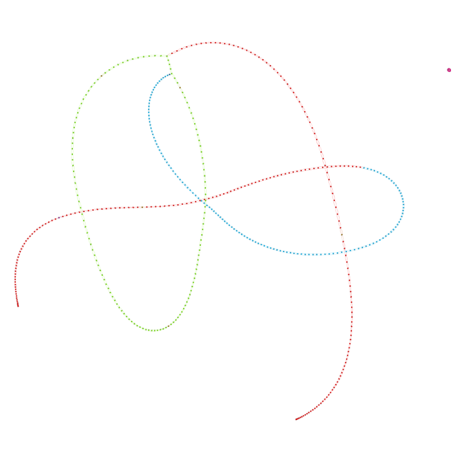	Possible mis-assembly	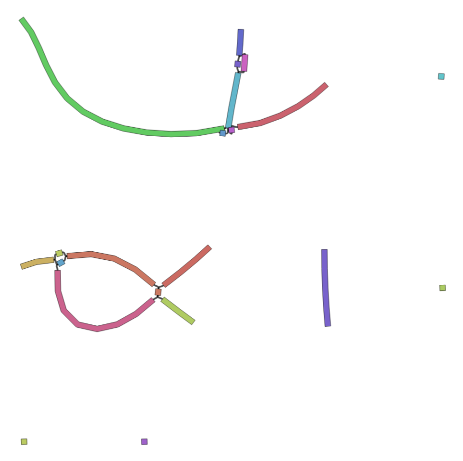
NCTC10766	*Escherichia alkalescens*	37	0	0	11		Possible mis-assembly	

						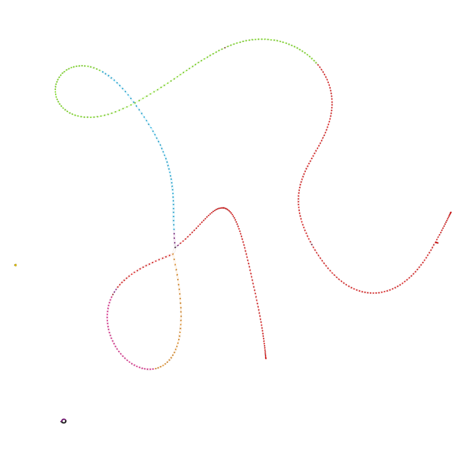		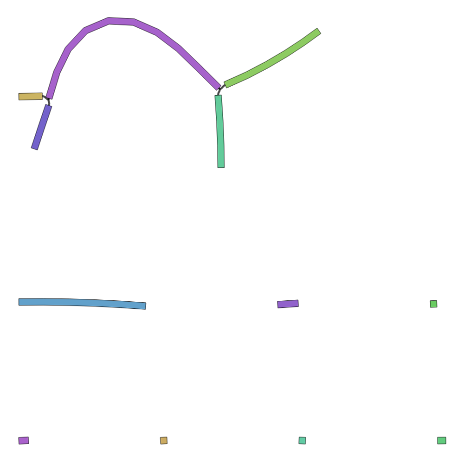
NCTC10767	*Escherichia alkalescens*	50	Pending	Pending	Pending	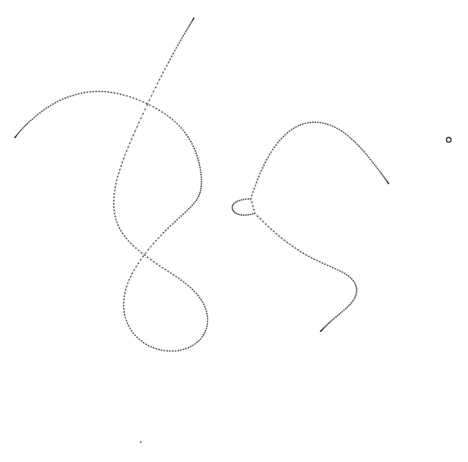	Mis-assembly/Fragmented	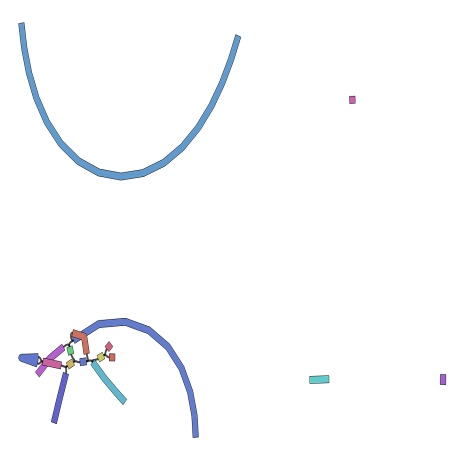
NCTC10768	*Citrobacter koseri*	52	1	0	5	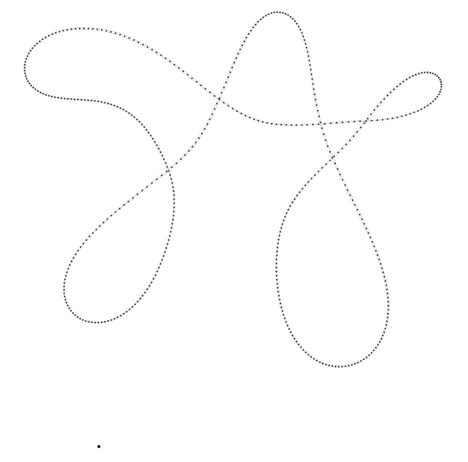	Finished circular assembly	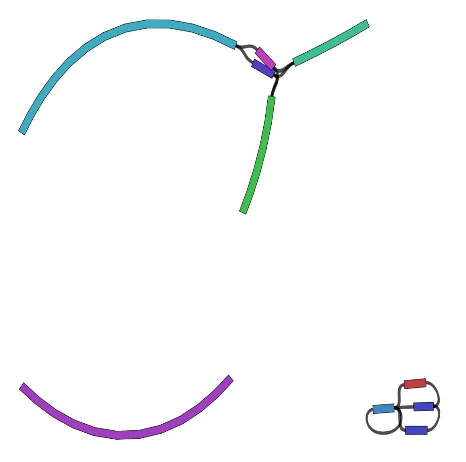
NCTC10786	*Citrobacter koseri*	42	1	1	0	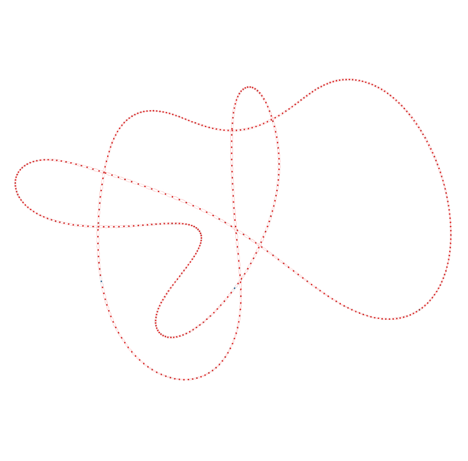	Finished circular assembly	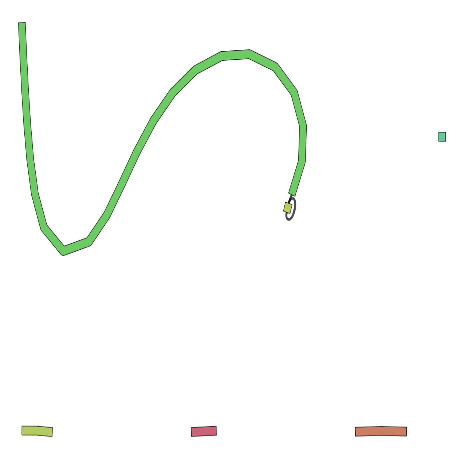
NCTC10788	*Staphylococcus aureus*	70	0	0	3	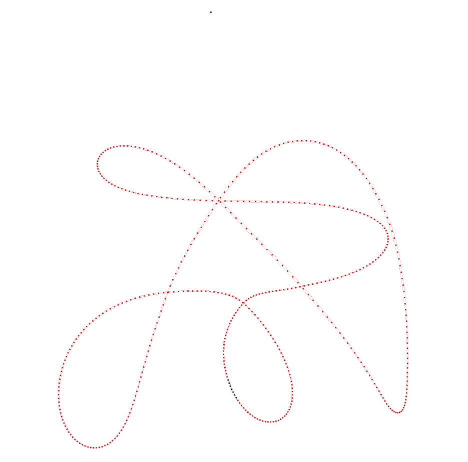	Finished circular assembly	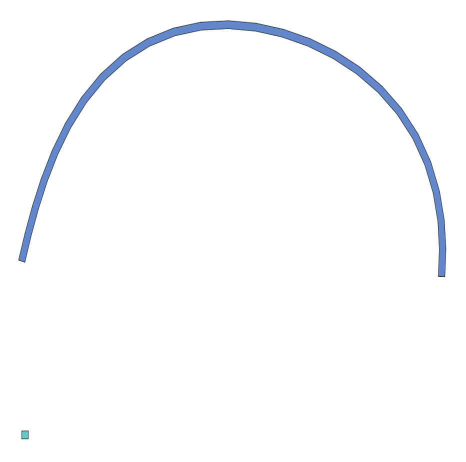
NCTC10794	*Haemophilus parahaemolyticus*	26	0	0	3	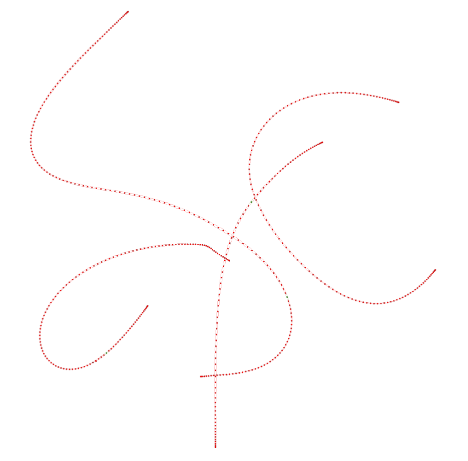	Mis-assembly/Fragmented	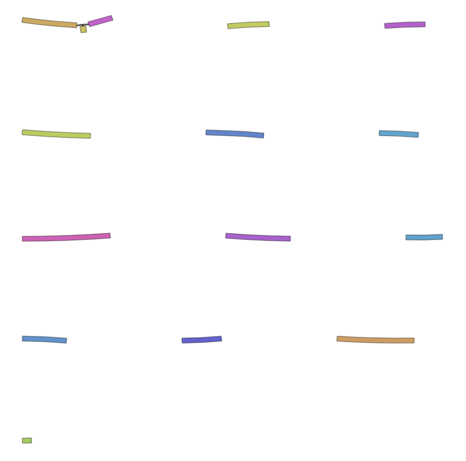
NCTC10796	*Chryseobacterium indologenes*	32	Pending	Pending	Pending		Finished circular assembly	

						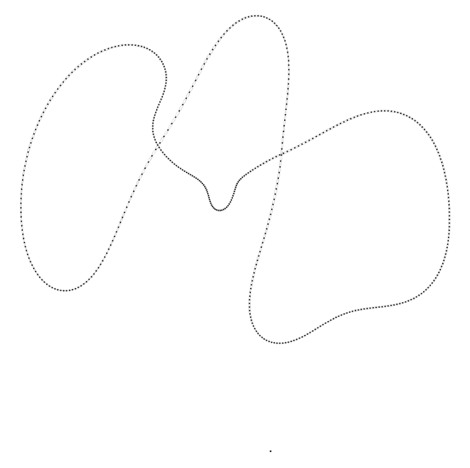		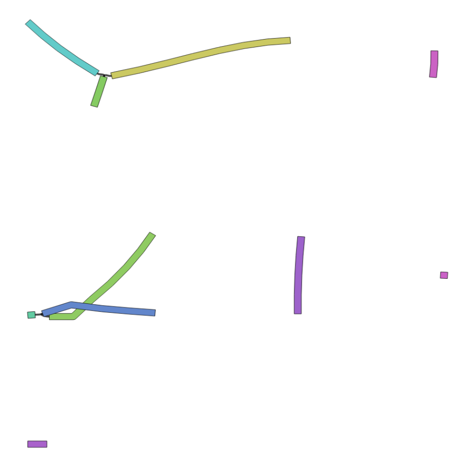
NCTC10804	*Staphylococcus aureus*	61	1	1	0	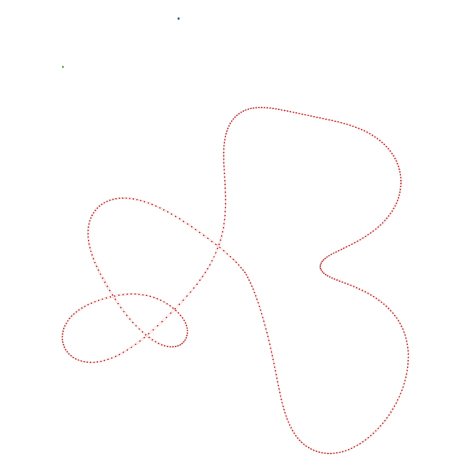	Finished circular assembly	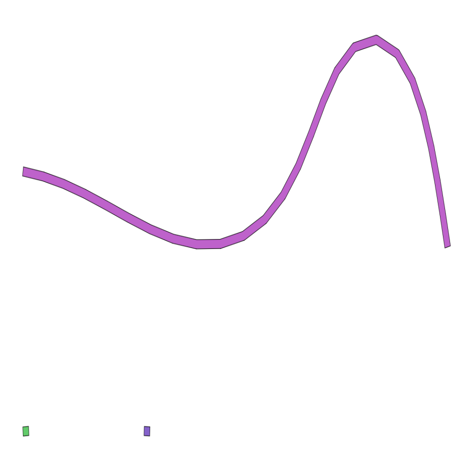
NCTC10805	*Citrobacter amalonaticus*	68	1	2	0	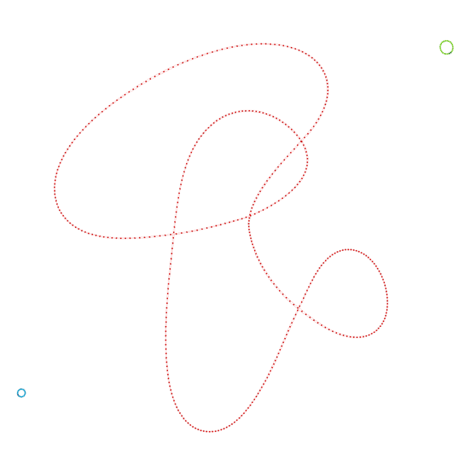	Finished circular assembly	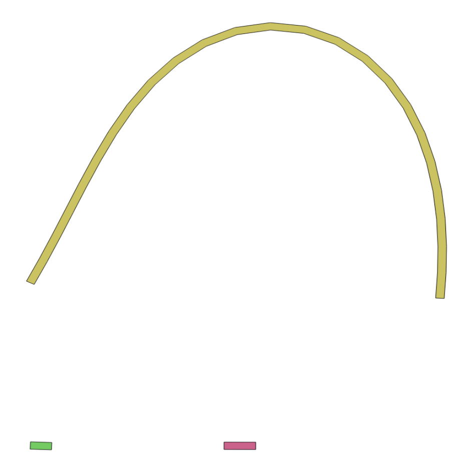
NCTC10807	*Achromobacter xylosoxidans*	65	1	0	0	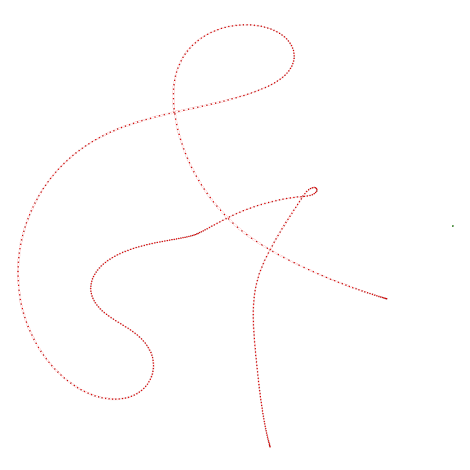	Finished assembly (lacking circularisation)	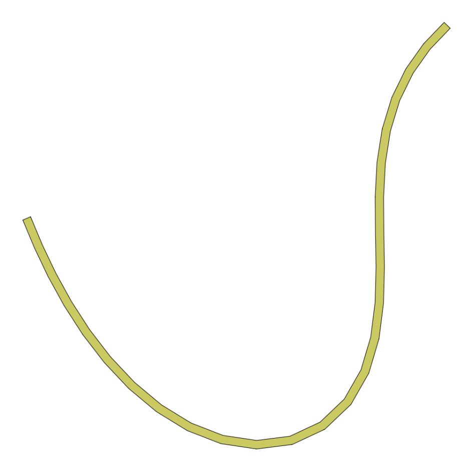
NCTC10810	*Citrobacter koseri*	67	1	2	0	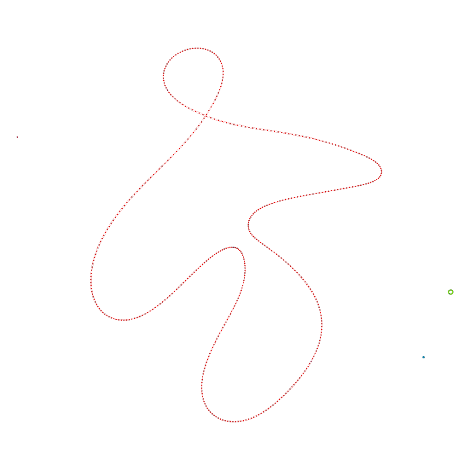	Finished circular assembly	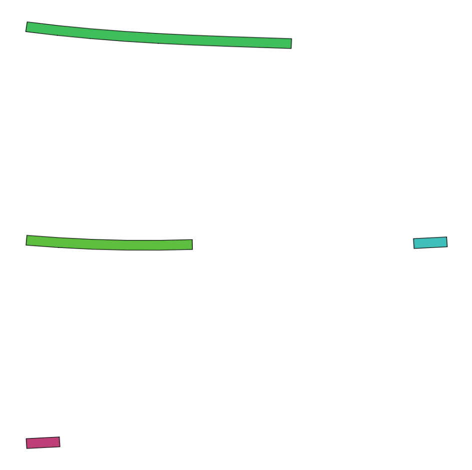
NCTC10833	*Staphylococcus aureus*	102	1	1	1	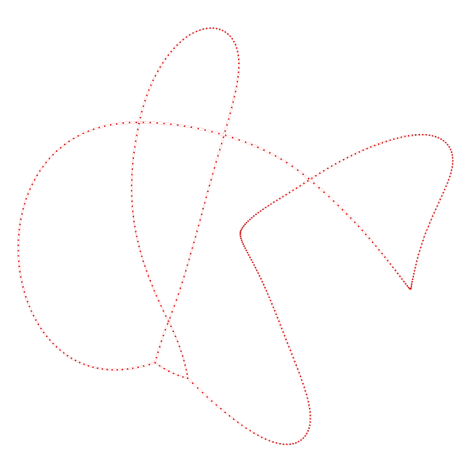	Mis-assembly/Fragmented	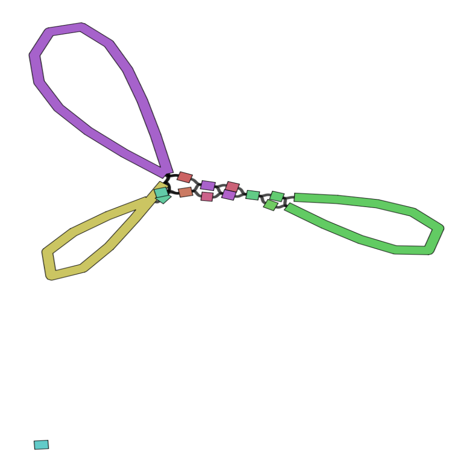
NCTC10848	*Serratia rubidaea*	56	1	0	0		Finished circular assembly	

						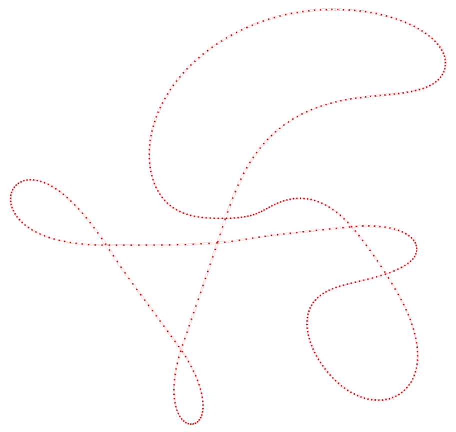		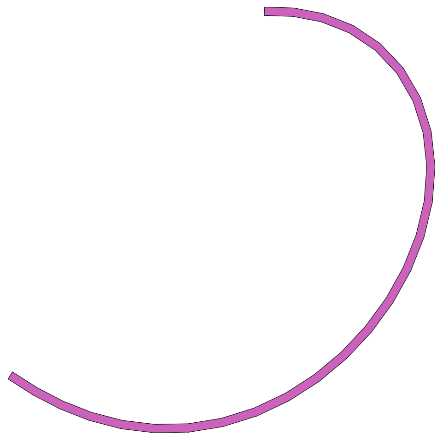
NCTC10849	*Citrobacter koseri*	47	1	1	0	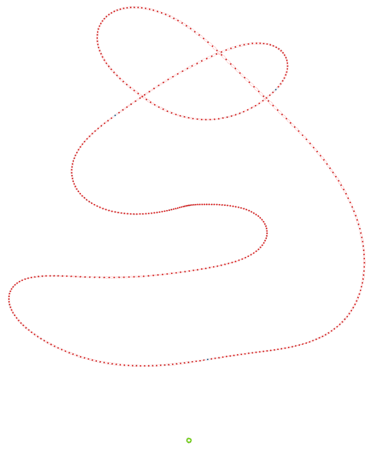	Finished circular assembly	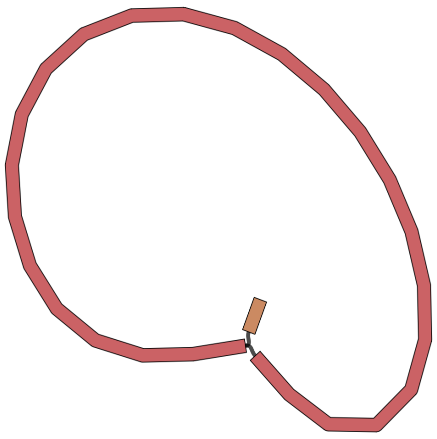
NCTC10850	*Escherichia coli*	60	0	0	2	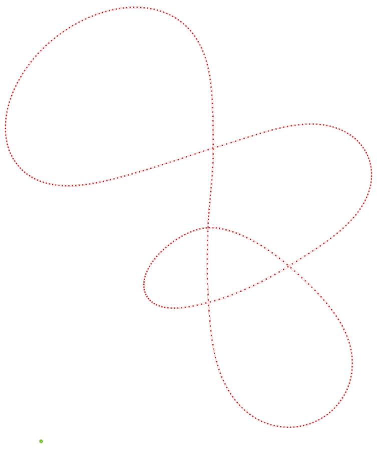	Finished circular assembly	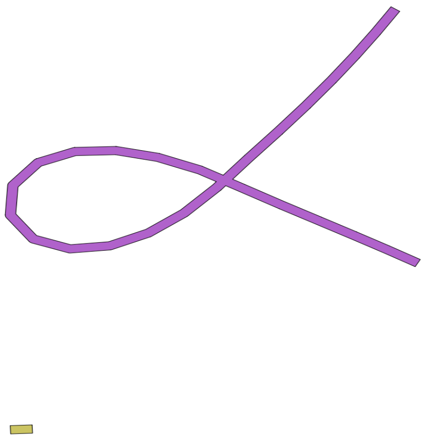
NCTC10855	*Escherichia aurescens*	44	0	0	5	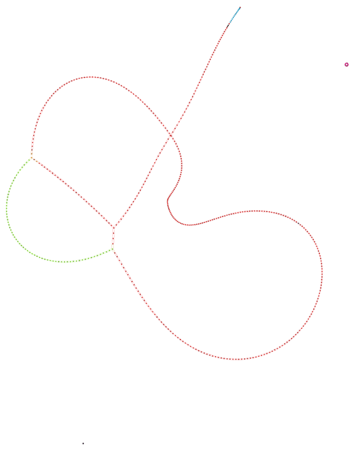	Mis-assembly/Fragmented	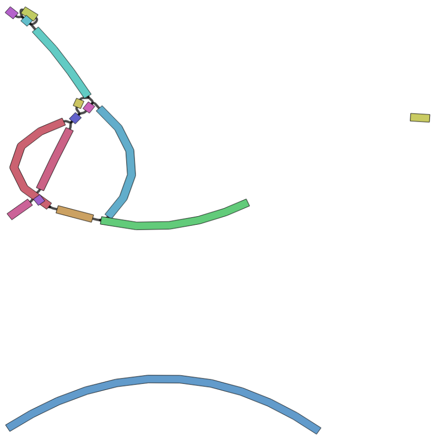
NCTC10863	*Escherichia coli*	49	1	1	0	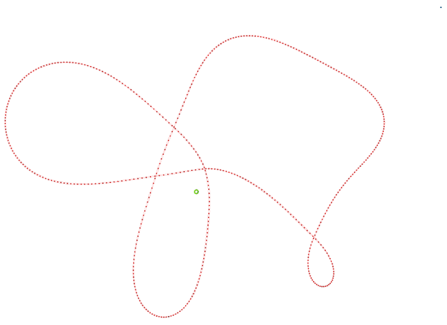	Finished circular assembly	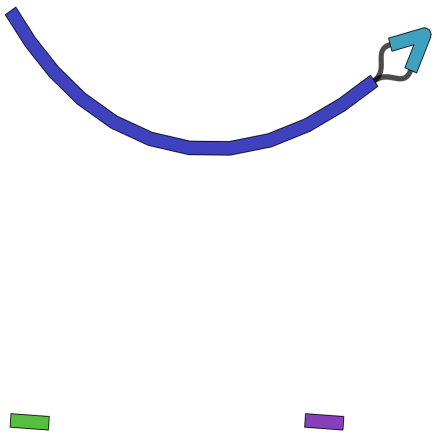
NCTC10864	*Escherichia coli*	46	0	0	6	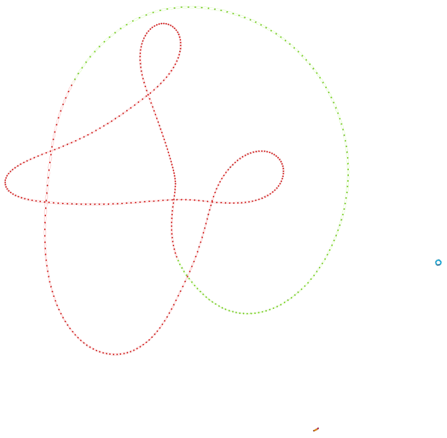	Finished circular assembly	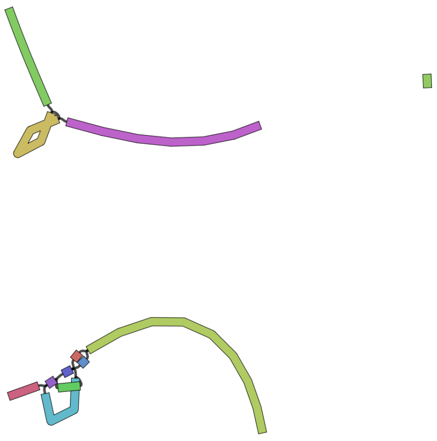
NCTC10865	*Escherichia coli*	32	1	0	5		Finished circular assembly	

						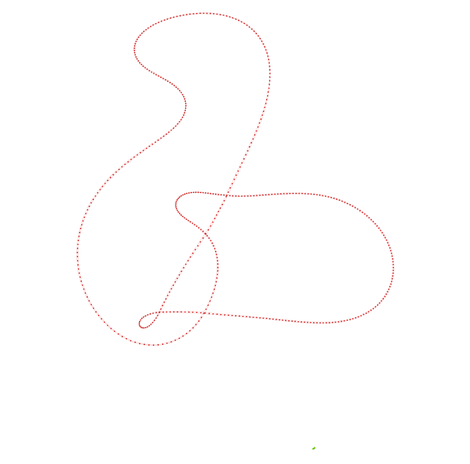		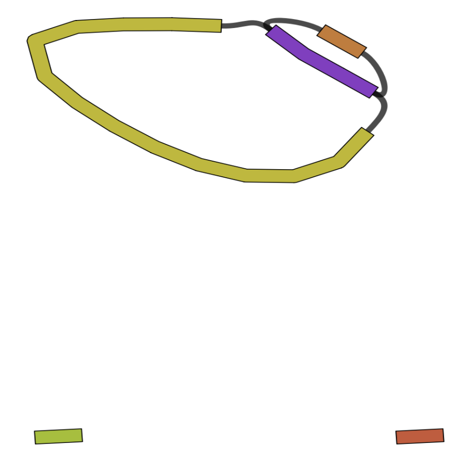
NCTC10866	*Streptococcus pyogenes*	151	Pending	Pending	Pending	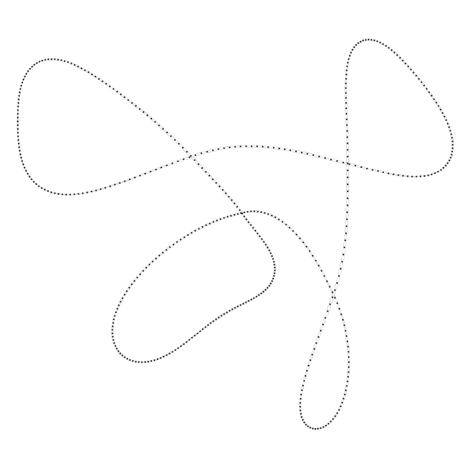	Finished circular assembly	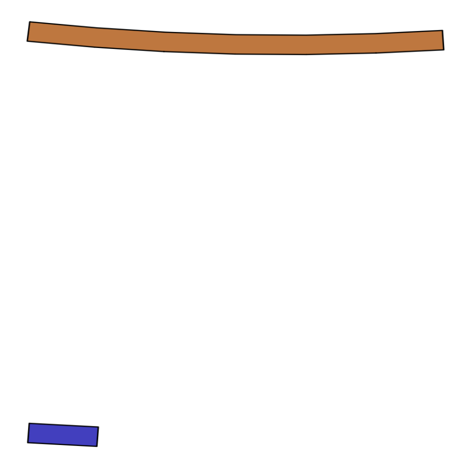
NCTC10872	*Streptococcus pyogenes*	129	Pending	Pending	Pending	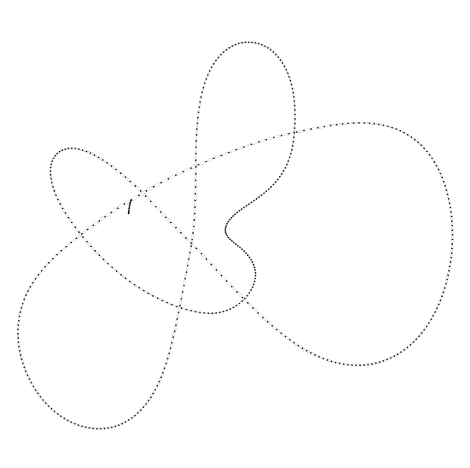	Finished circular assembly	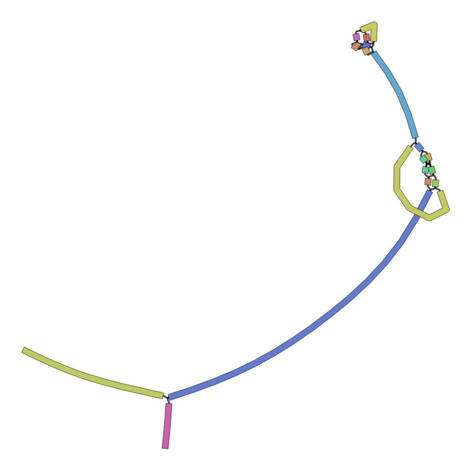
NCTC10884	*Vibrio parahaemolyticus*	82	0	0	2	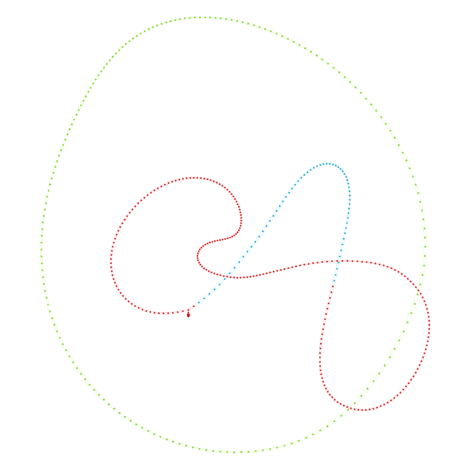	Finished circular assembly	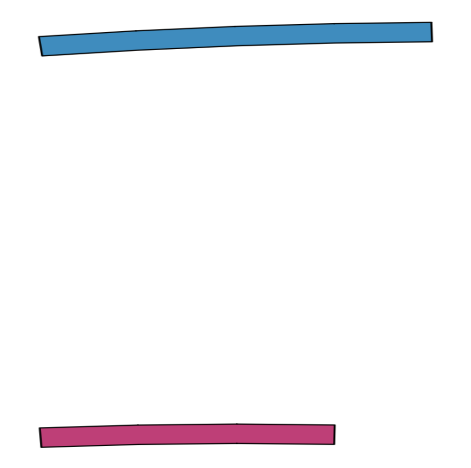
NCTC10897	*Pseudomonas mendocina*	52	Pending	Pending	Pending	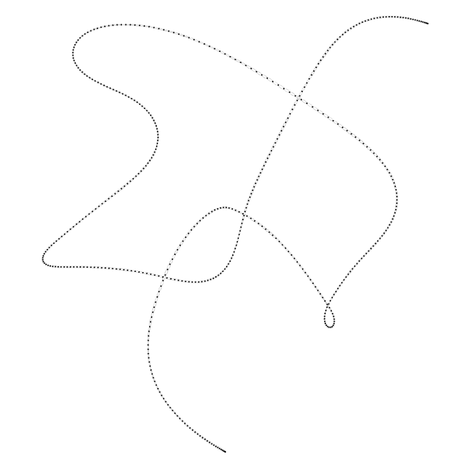	Finished assembly (lacking circularisation)	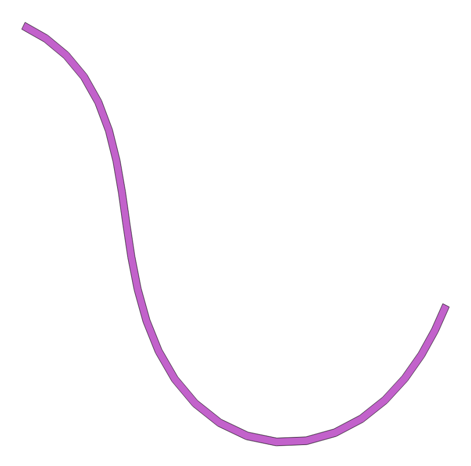
NCTC10903	*Vibrio parahaemolyticus*	48	Pending	Pending	Pending	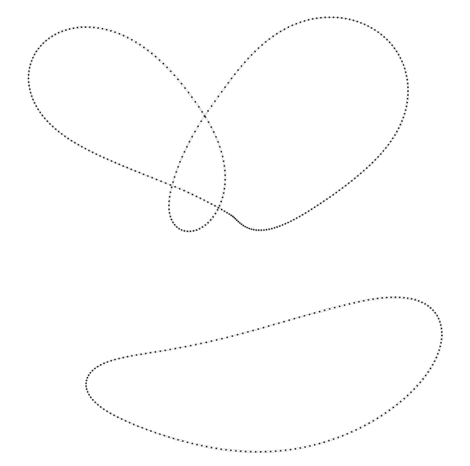	Finished circular assembly	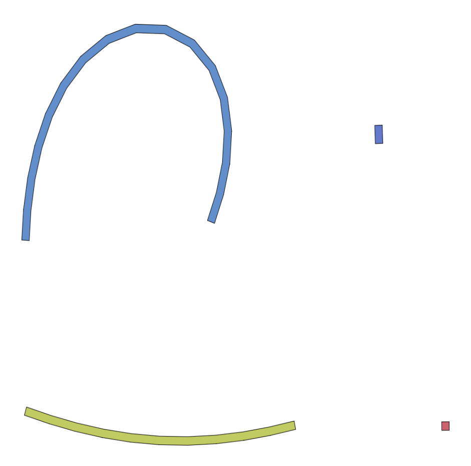
NCTC10904	*Streptococcus sanguinis*	97	1	0	0		Finished assembly (lacking circularisation)	

						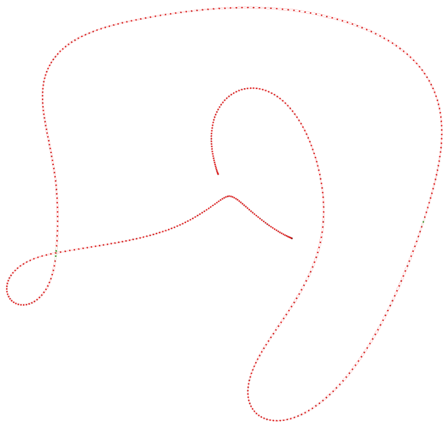		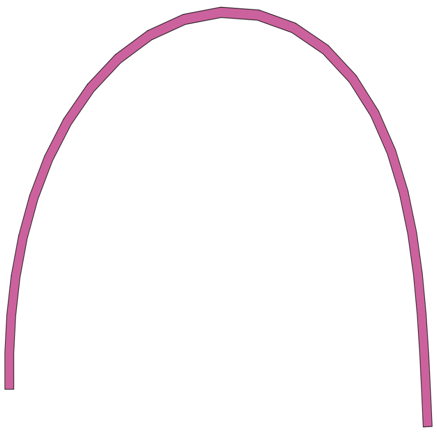
NCTC10907	*Bordetella pertussis*	44	0	0	2	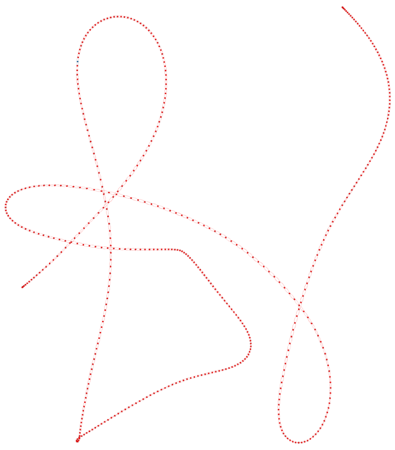	Finished assembly (lacking circularisation)	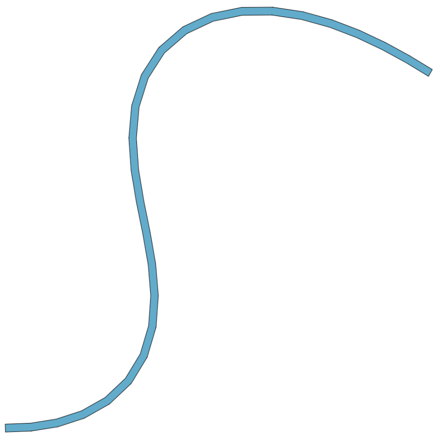
NCTC10923	*Streptococcus mutans*	73	0	0	2	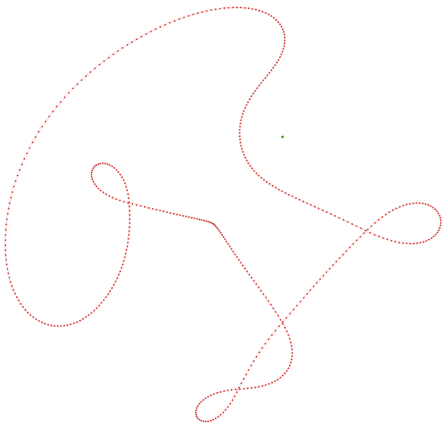	Finished circular assembly	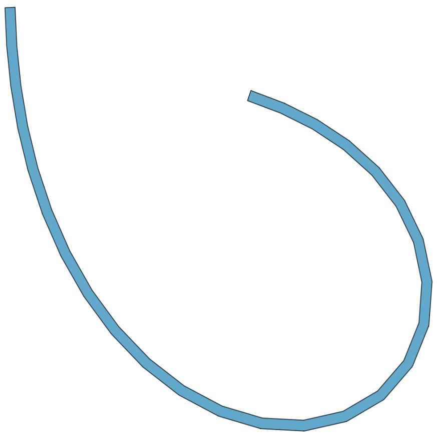
NCTC10924	*Streptococcus sp*	219	1	0	0	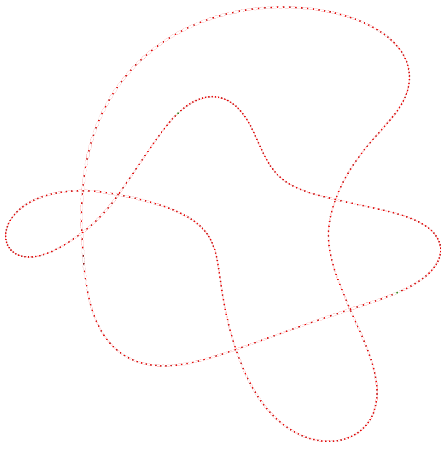	Finished circular assembly	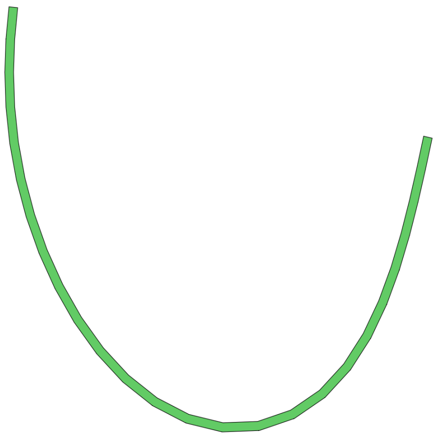
NCTC10925	*Streptococcus sp.*	146	1	0	0	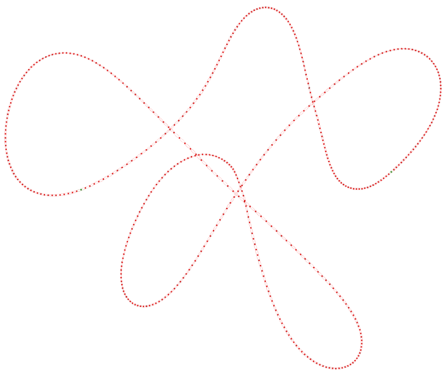	Finished circular assembly	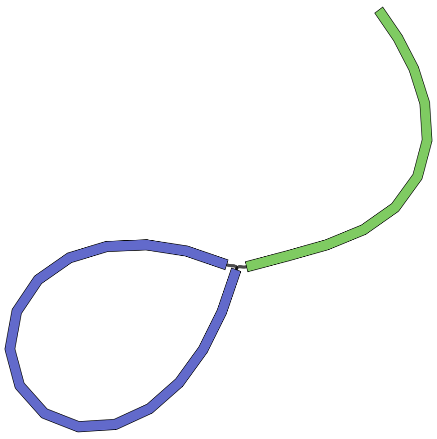
NCTC10928	*Neisseria gonorrhoeae*	50	1	1	1	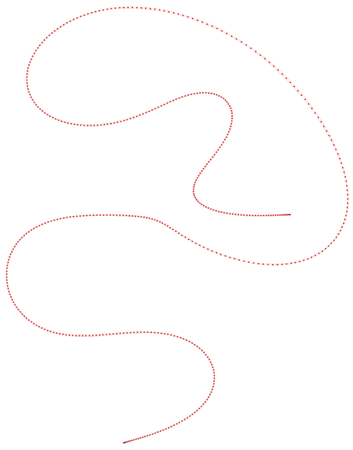	Finished assembly (lacking circularisation)	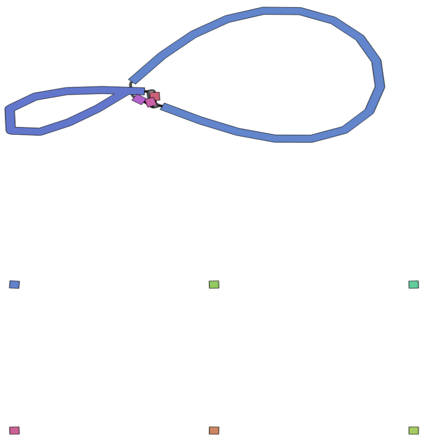
NCTC10931	*Neisseria gonorrhoeae*	133	0	0	4		Finished assembly with multiple traversals	

						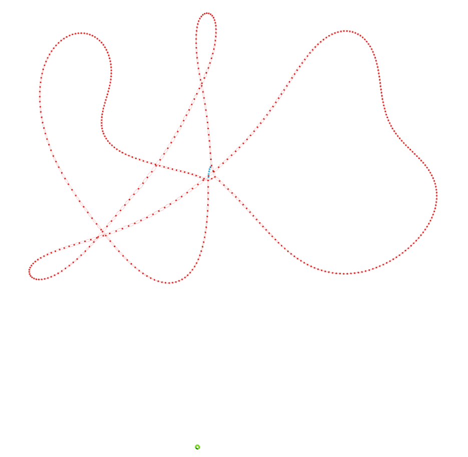		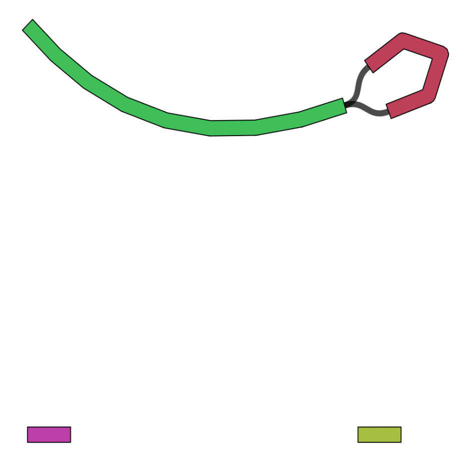
NCTC10957	*Escherichia coli*	58	1	2	0	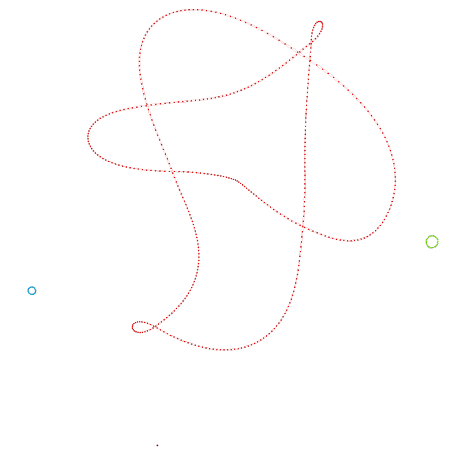	Finished circular assembly	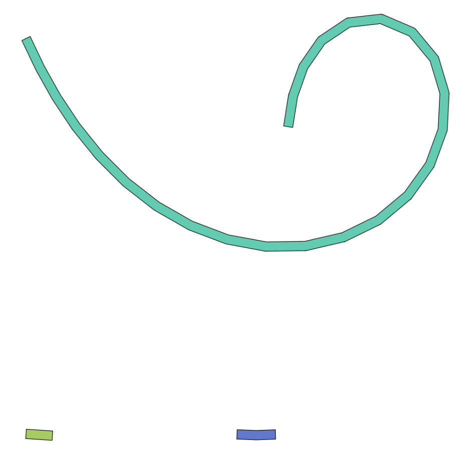
NCTC10958	*Escherichia coli*	60	1	1	0	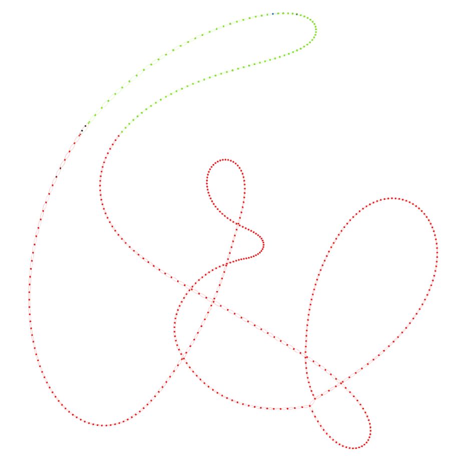	Finished assembly with multiple traversals	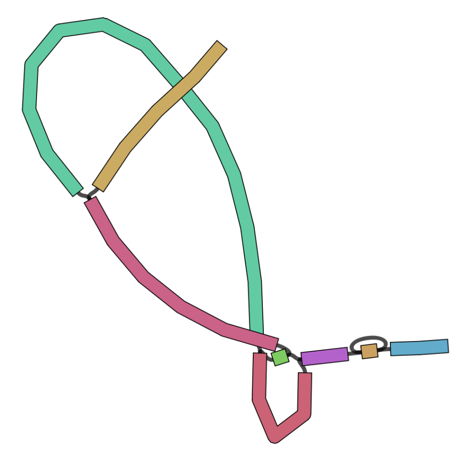
NCTC10959	*Escherichia coli*	42	0	0	6	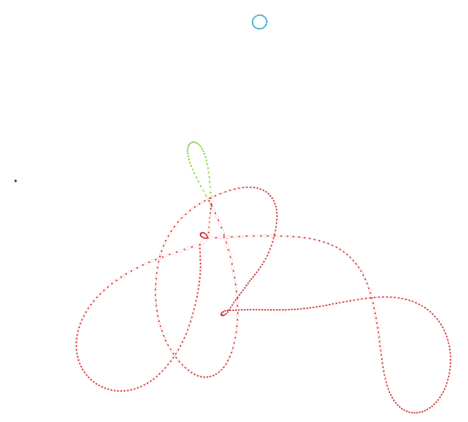	Mis-assembly	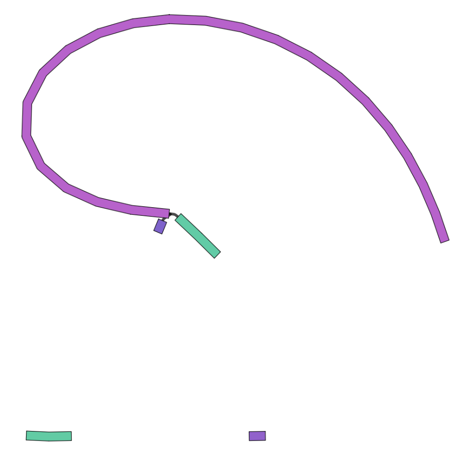
NCTC10960	*Escherichia coli*	63	0	0	5	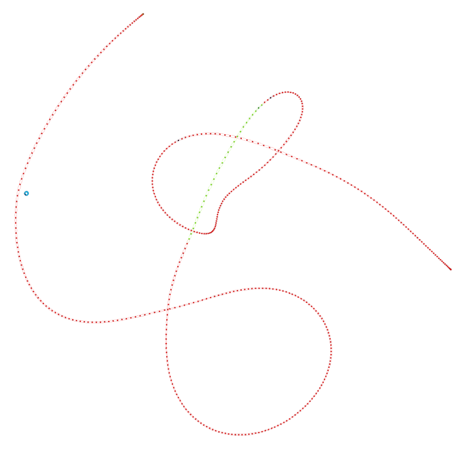	Finished assembly (lacking circularisation)	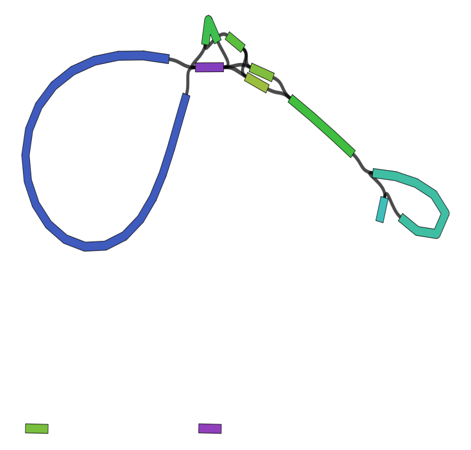
NCTC10961	*Escherichia coli*	60	0	0	3	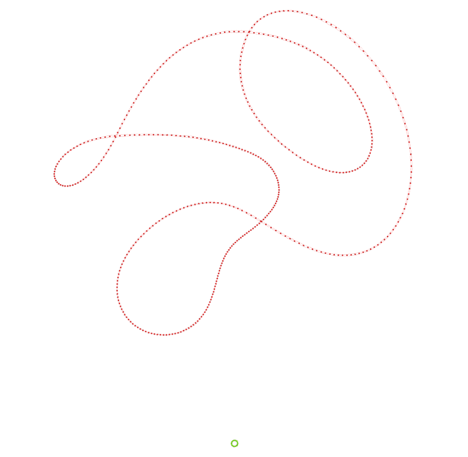	Finished circular assembly	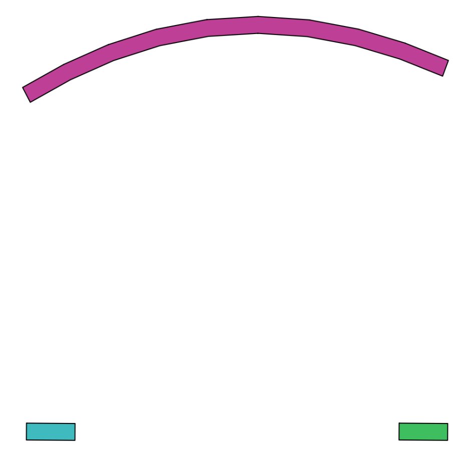
NCTC10962	*Escherichia coli*	42	1	2	0		Finished circular assembly	

						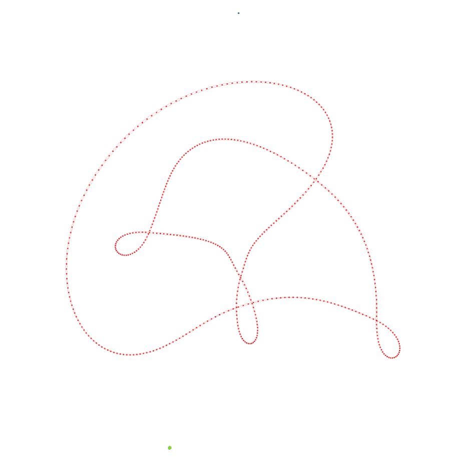		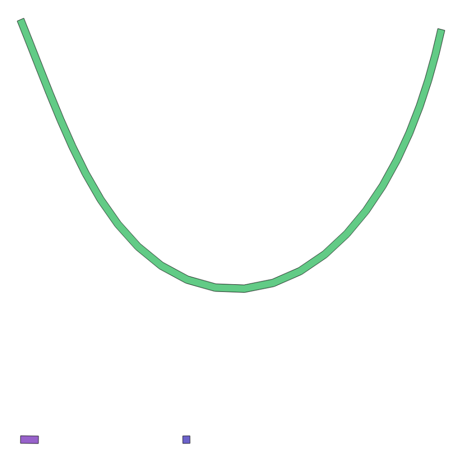
NCTC10965	*Shimwellia blattae*	58	1	0	0	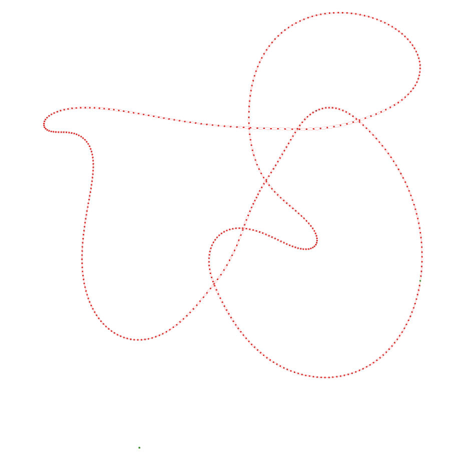	Finished circular assembly	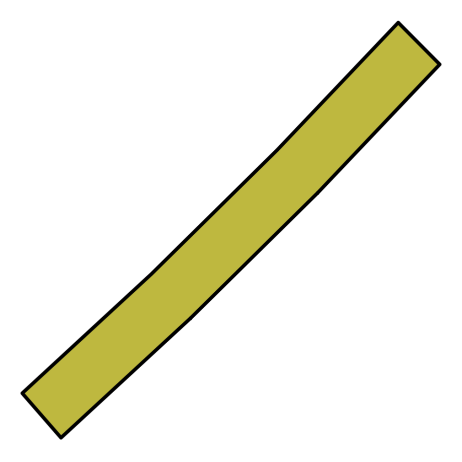
NCTC10973	*Escherichia coli*	79	1	0	0	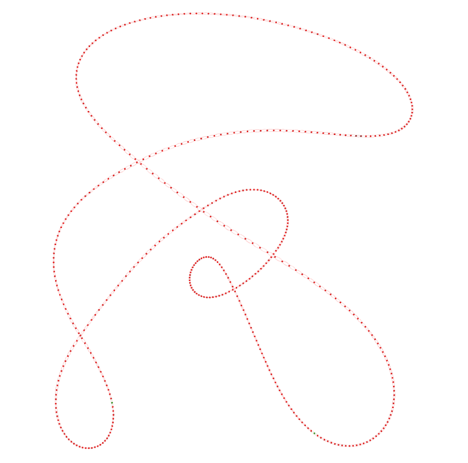	Finished circular assembly	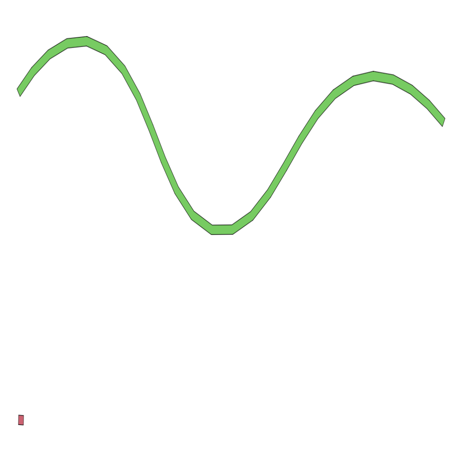
NCTC10974	*Escherichia coli*	30	1	0	1	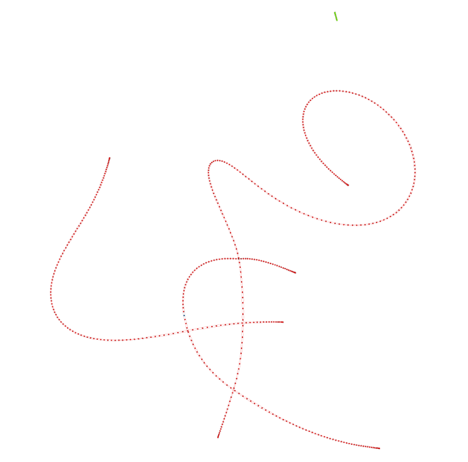	Mis-assembly/Fragmented	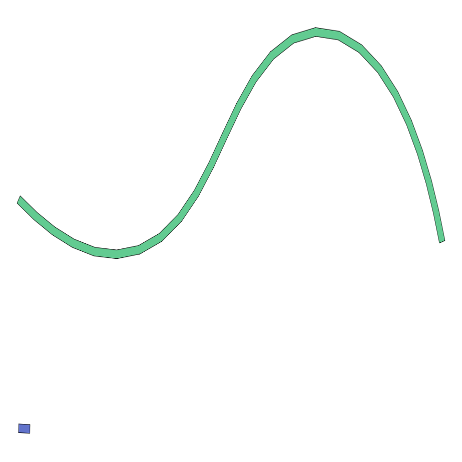
NCTC10975	*Proteus mirabilis*	39	Pending	Pending	Pending	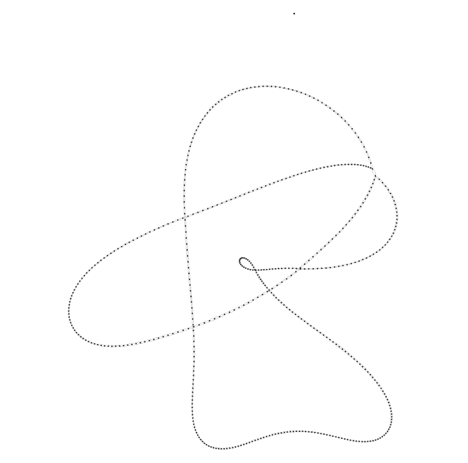	Finished assembly with multiple traversals	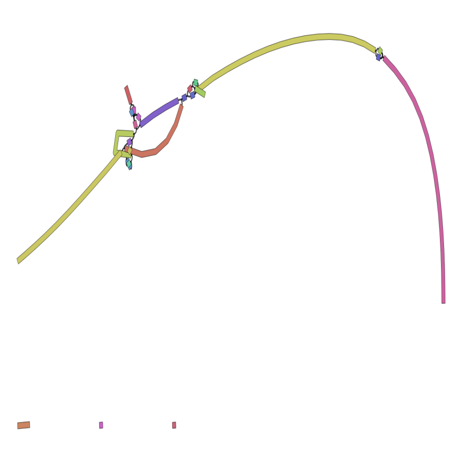
NCTC10988	*Staphylococcus aureus*	87	0	0	13	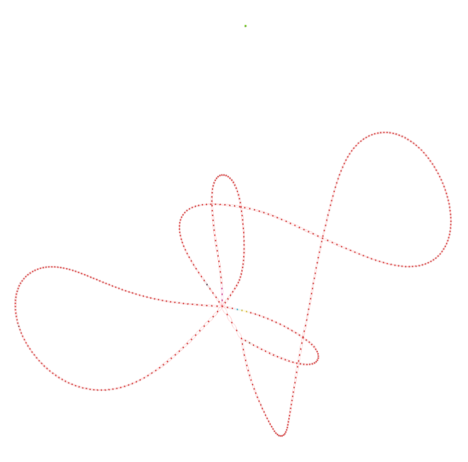	Mis-assembly	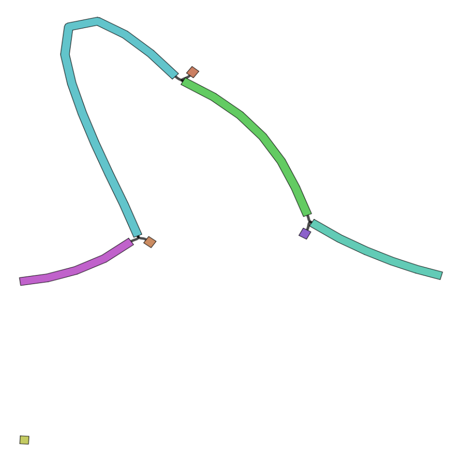
NCTC10999	*Streptococcus porcinus*	120	Pending	Pending	Pending		Finished circular assembly	

						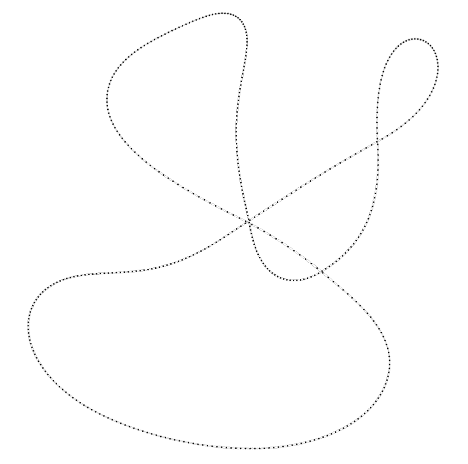		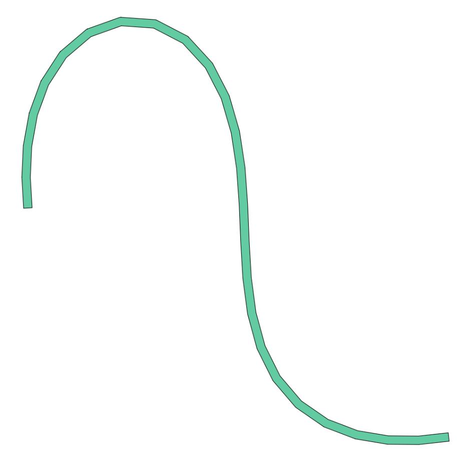
NCTC11022	*Escherichia coli*	42	0	0	3	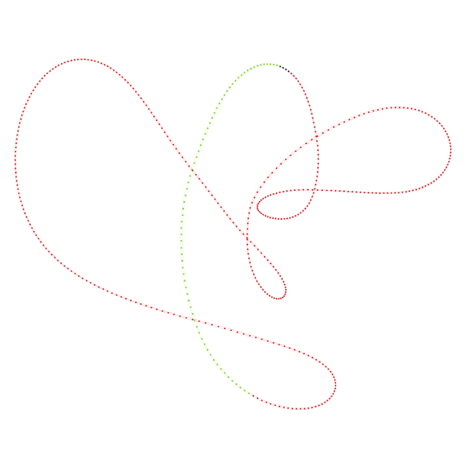	Finished circular assembly	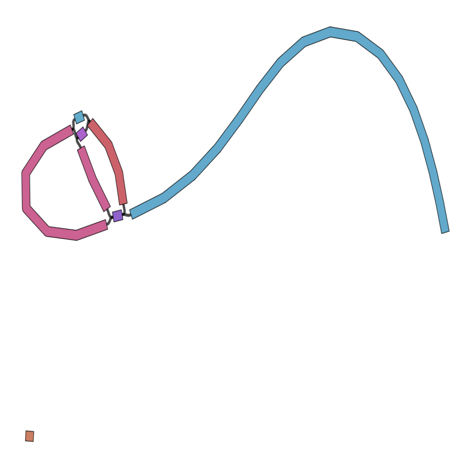
NCTC11023	*Escherichia coli*	82	1	0	0	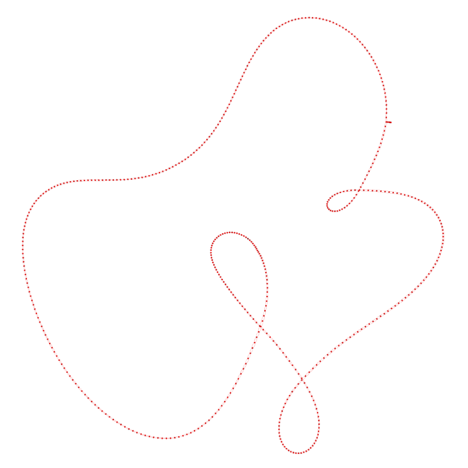	Finished circular assembly	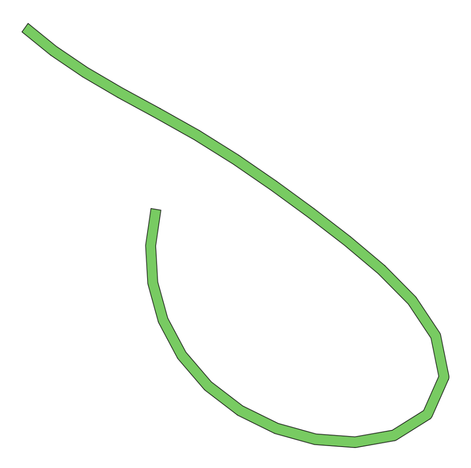
NCTC11030	*Sphingomonas paucimobilis*	32	Pending	Pending	Pending	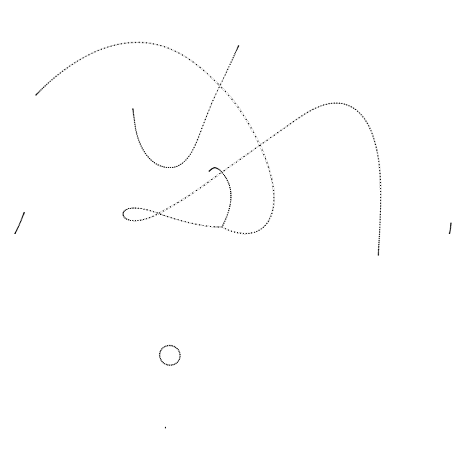	Mis-assembly/Fragmented	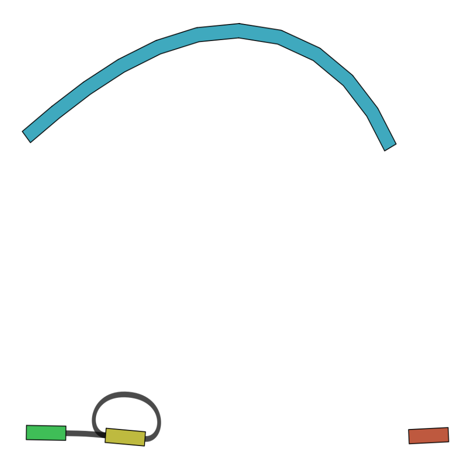
NCTC11042	*Staphylococcus haemolyticus*	114	1	0	2	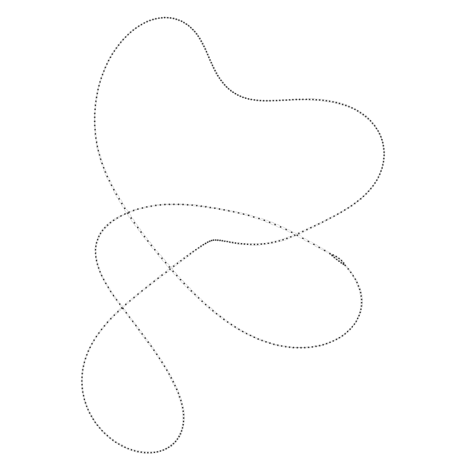	Finished circular assembly	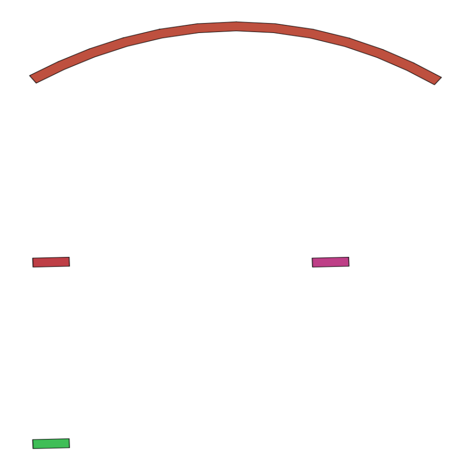
NCTC11043	*Staphylococcus xylosus*	96	Pending	Pending	Pending	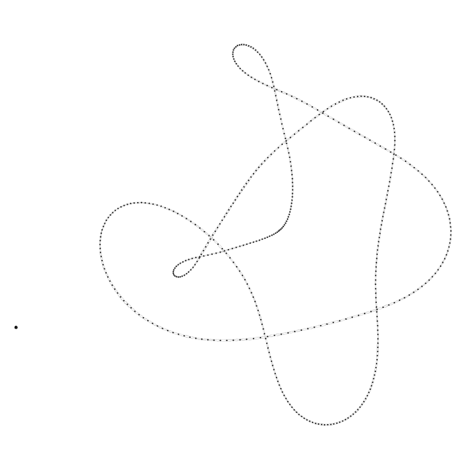	Finished circular assembly	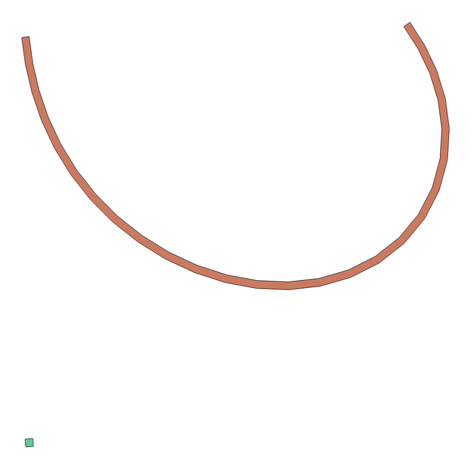
NCTC11044	*Staphylococcus warneri*	57	1	0	0		Finished circular assembly	

						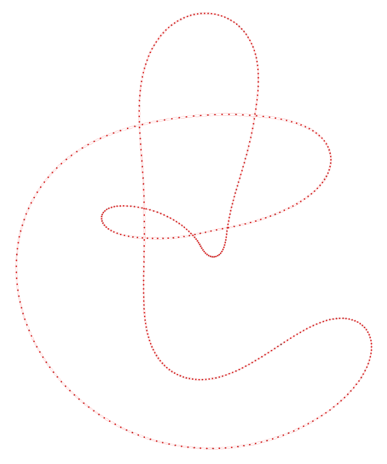		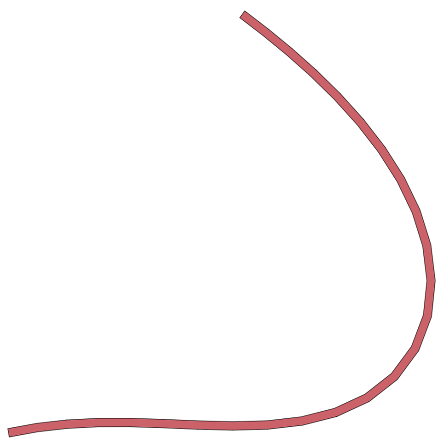	
NCTC11045	*Staphylococcus capitis*	81	Pending	Pending	Pending	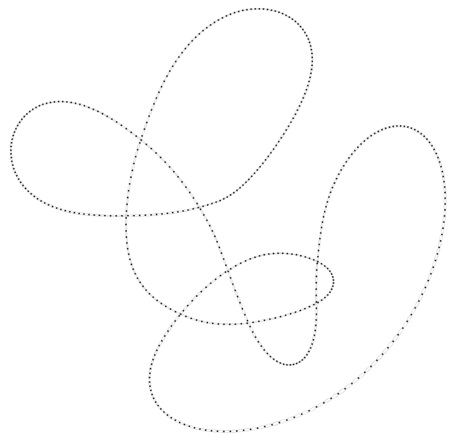	Finished circular assembly	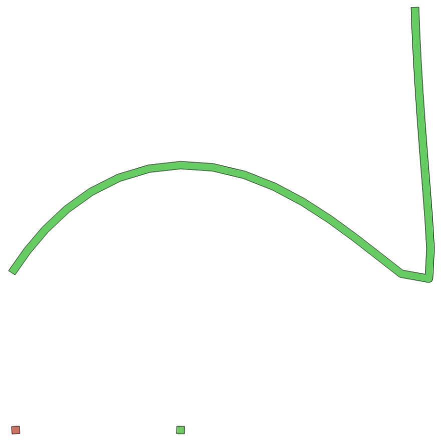	
NCTC11046	*Staphylococcus simulans*	161	Pending	Pending	Pending	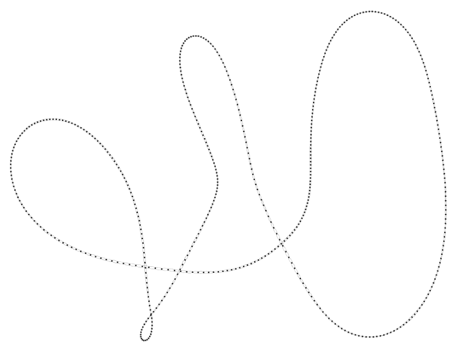	Finished circular assembly	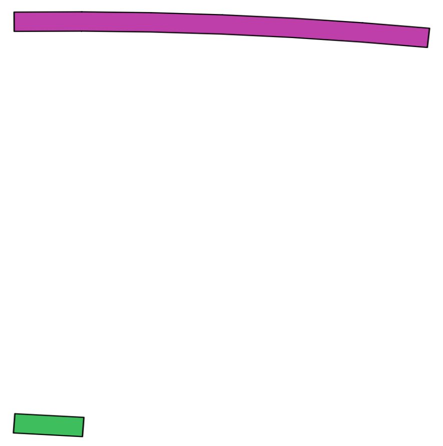	
NCTC11047	*Staphylococcus epidermidis*	63	1	0	4	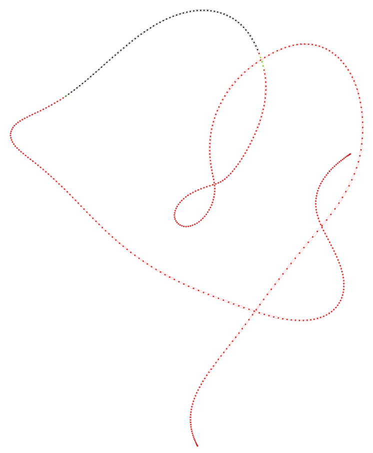	Finished assembly (lacking circularisation)	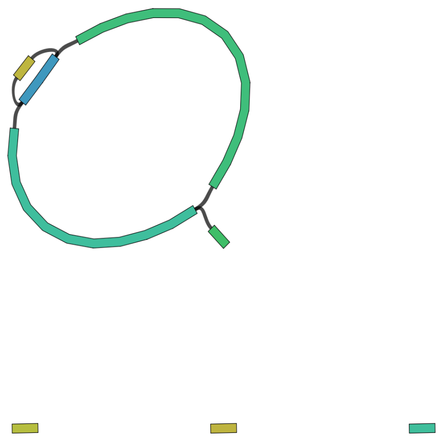	
NCTC11048	*Staphylococcus intermedius*	79	Pending	Pending	Pending	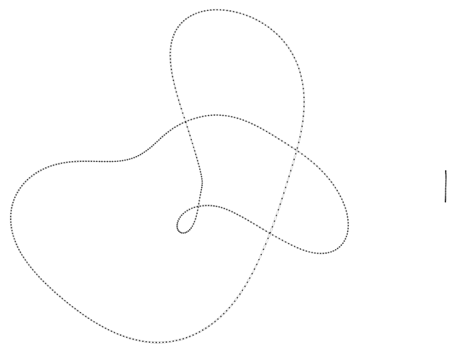	Finished circular assembly	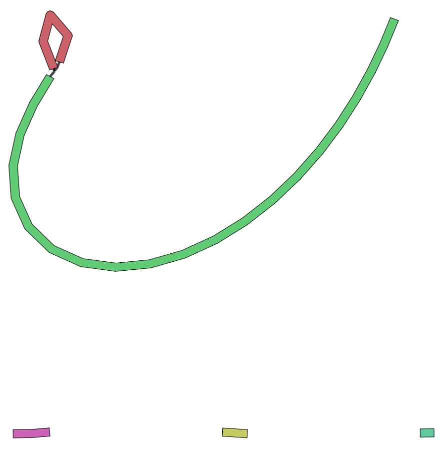	
NCTC11063	*Streptococcus milleri*	187	Pending	Pending	Pending	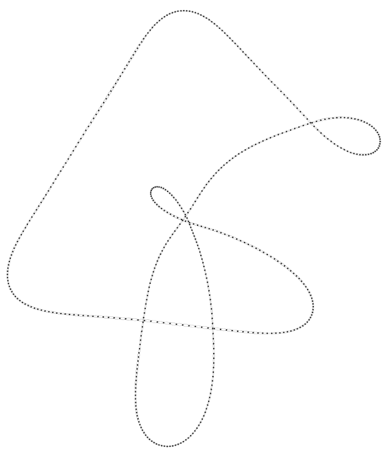	Finished circular assembly	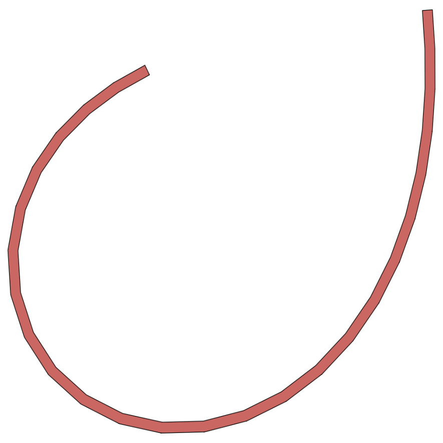	
NCTC11064	*Streptococcus milleri*	168	Pending	Pending	Pending		Finished circular assembly		

						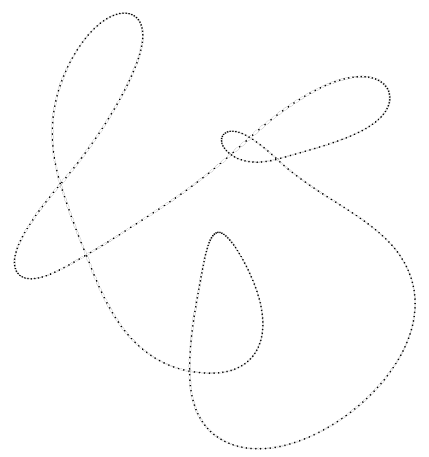		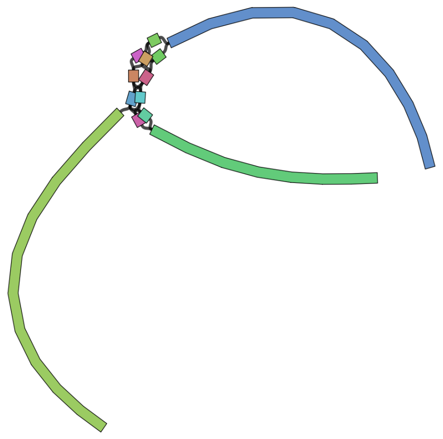
NCTC11067	*Vibrio vulnificus*	55	2	0	1	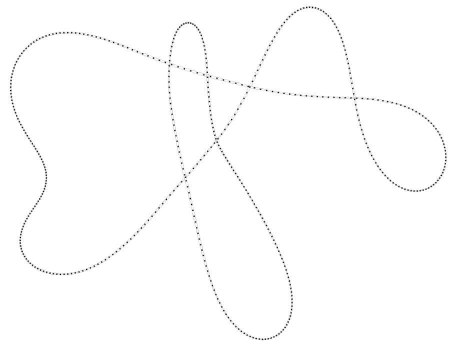	Finished circular assembly	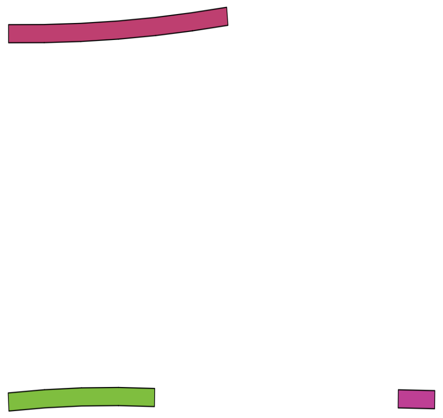
NCTC11074	*Citrobacter koseri*	71	1	0	2	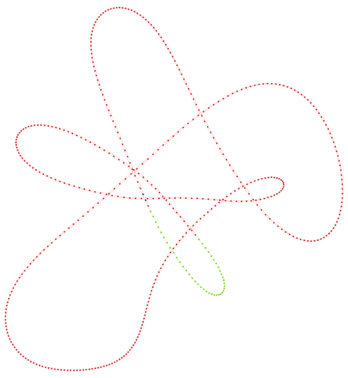	Finished circular assembly	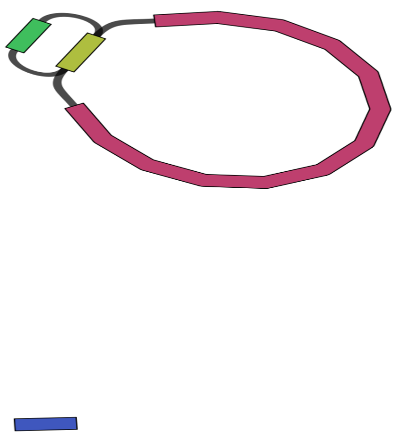
NCTC11075	*Citrobacter koseri*	30	0	0	1	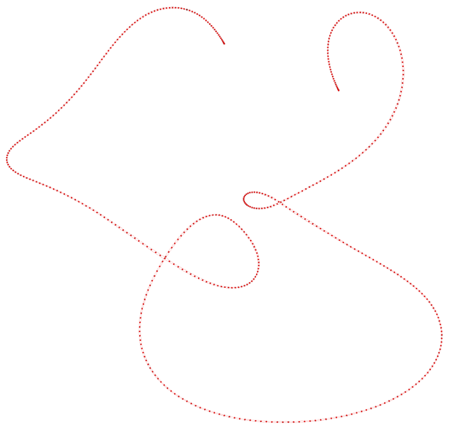	Finished assembly (lacking circularisation)	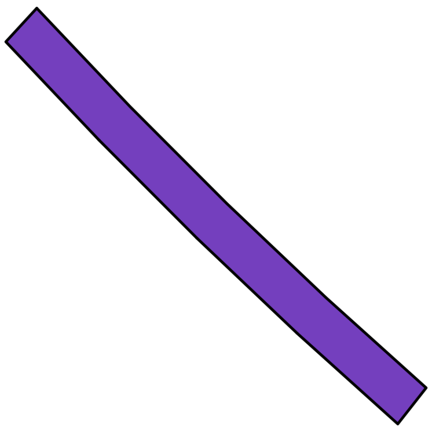
NCTC11076	*Citrobacter koseri*	64	0	0	2	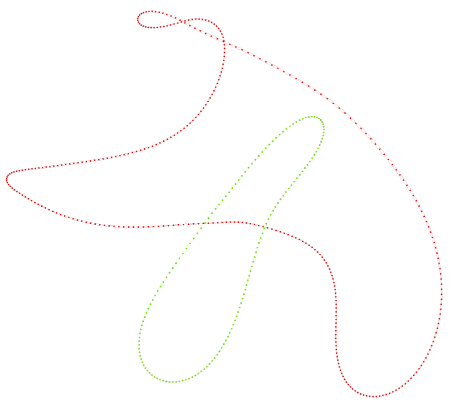	Finished circular assembly	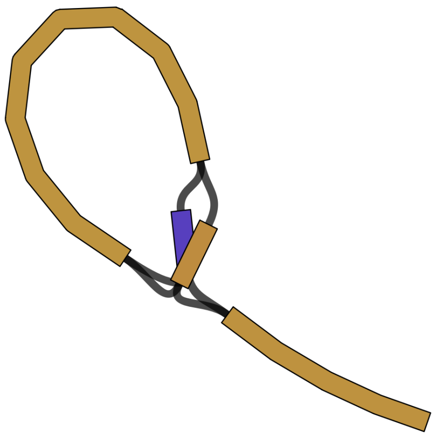
NCTC11077	*Citrobacter koseri*	73	0	0	2	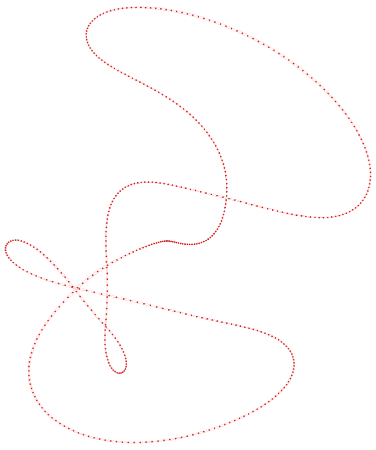	Finished circular assembly	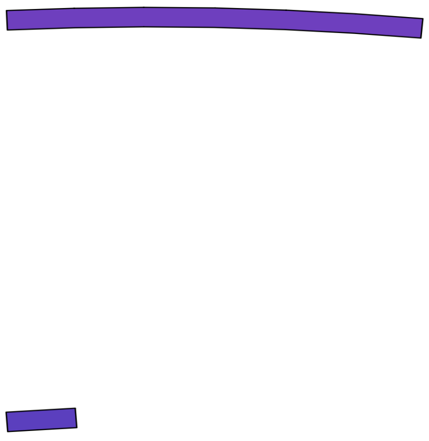
NCTC11079	*Streptococcus agalactiae*	81	Pending	Pending	Pending		Finished circular assembly	

						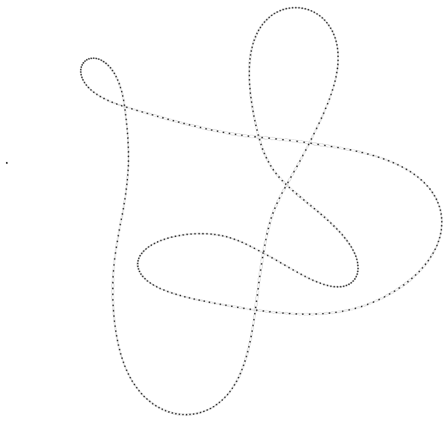		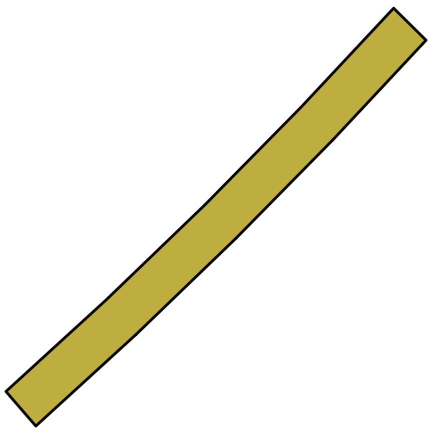
NCTC11080	*Streptococcus agalactiae*	44	Pending	Pending	Pending	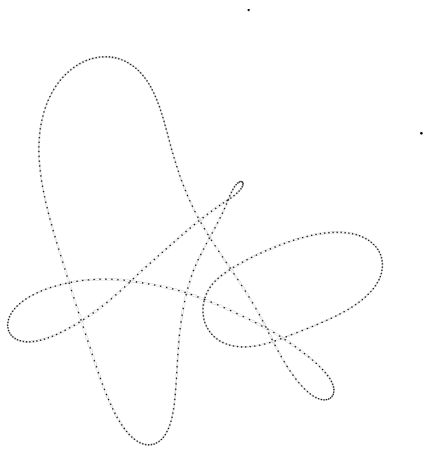	Finished circular assembly	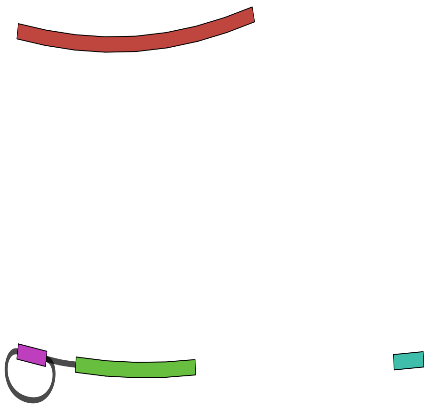
NCTC11085	*Streptococcus sanguinis*	88	Pending	Pending	Pending	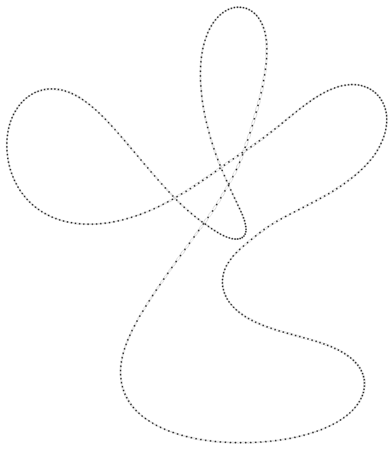	Finished circular assembly	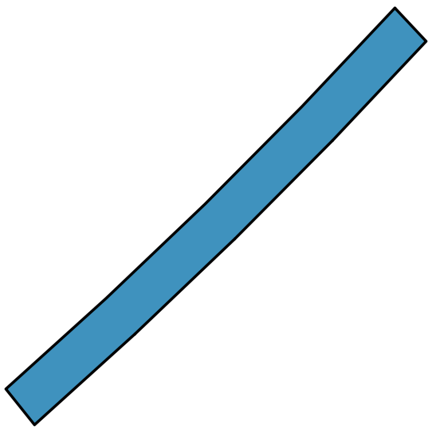
NCTC11086	*Streptococcus sanguinis*	34	Pending	Pending	Pending	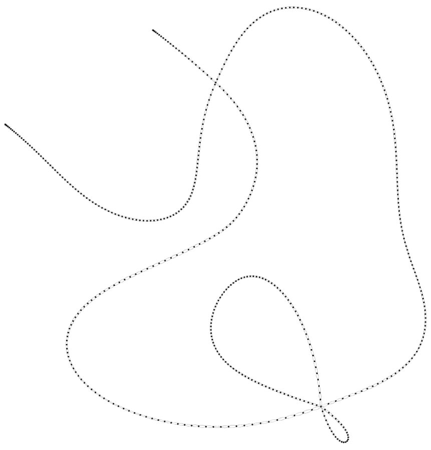	Finished assembly (lacking circularisation)	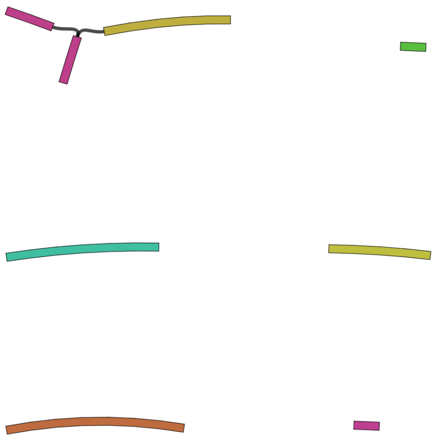
NCTC11096	*Aggregatibacter aphrophilus*	93	1	0	0	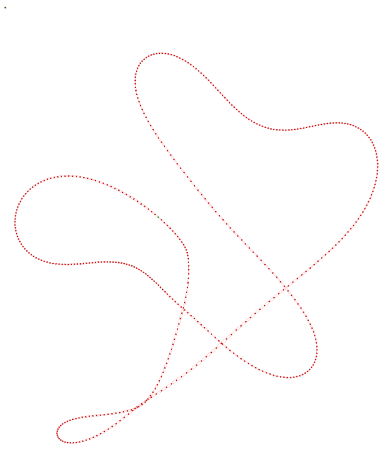	Mis-assembly	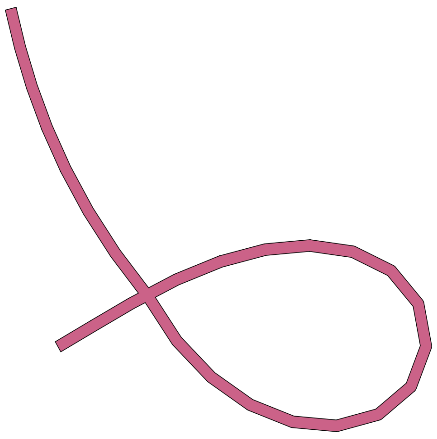
NCTC11100	*Escherichia coli*	59	1	0	1	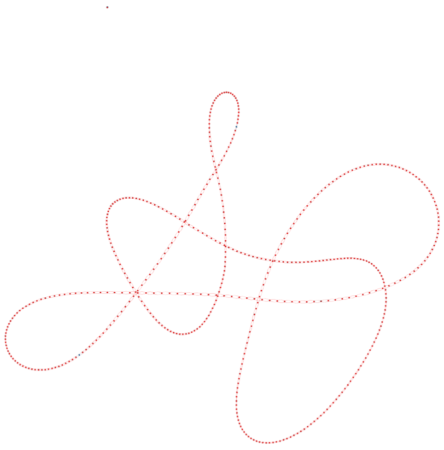	Finished circular assembly	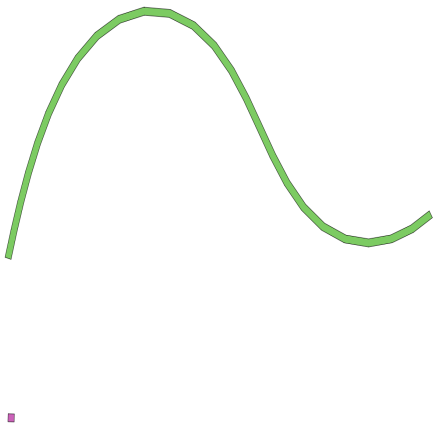
NCTC11101	*Escherichia coli*	81	1	1	0		Finished circular assembly	

						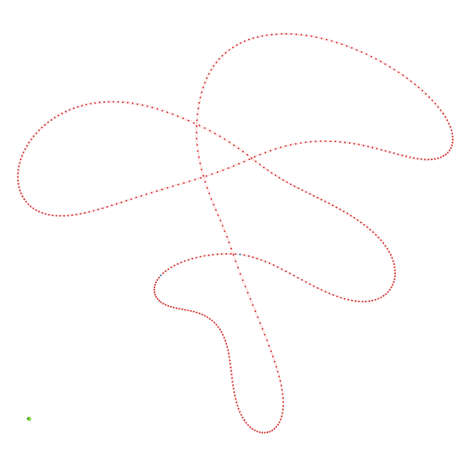		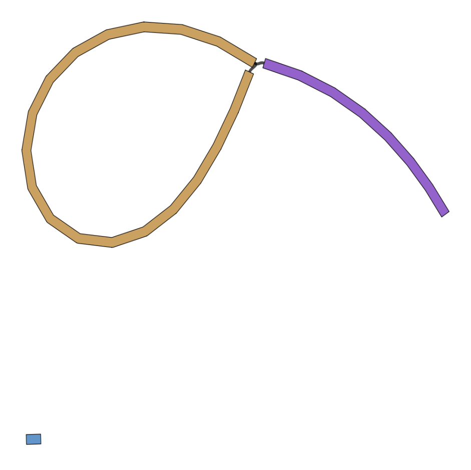
NCTC11102	*Escherichia coli*	73	1	1	0	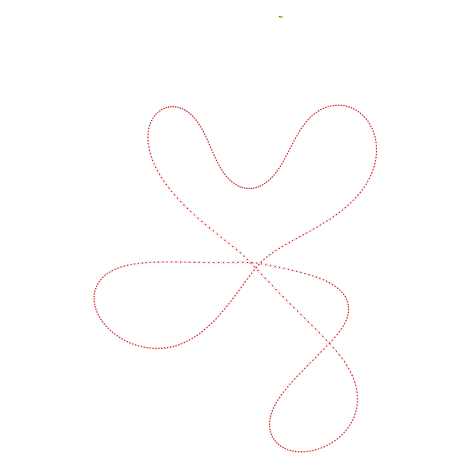	Finished circular assembly	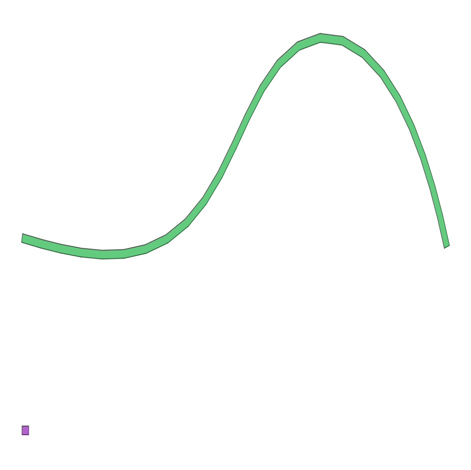
NCTC11103	*Escherichia coli*	48	1	2	0	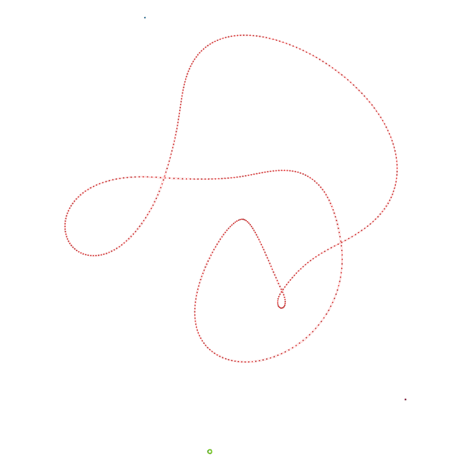	Finished circular assembly	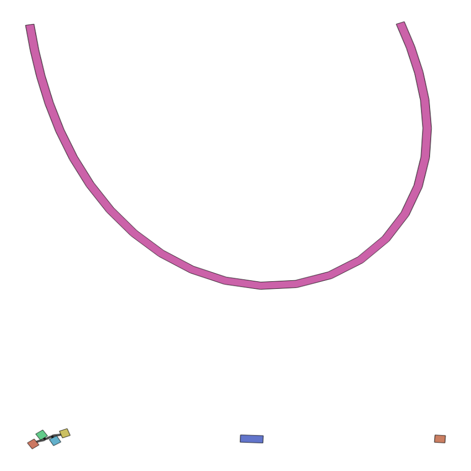
NCTC11104	*Escherichia coli*	45	Pending	Pending	Pending	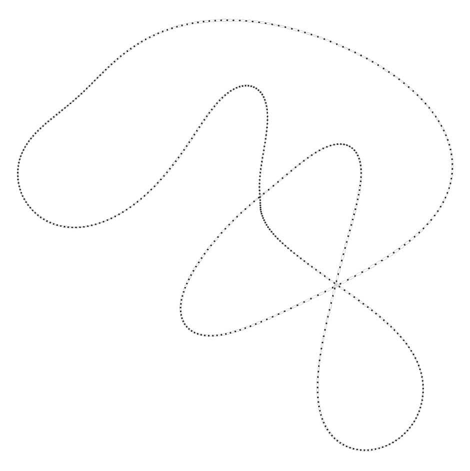	Finished circular assembly	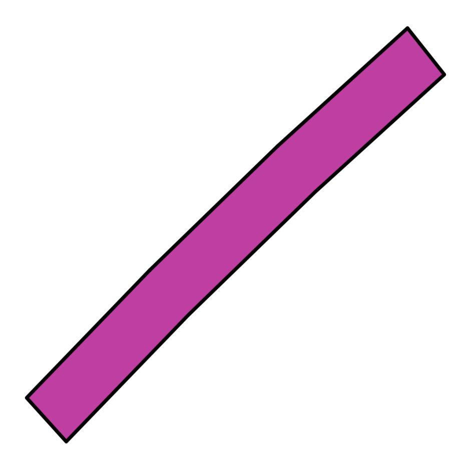
NCTC11105	*Escherichia coli*	49	0	0	1	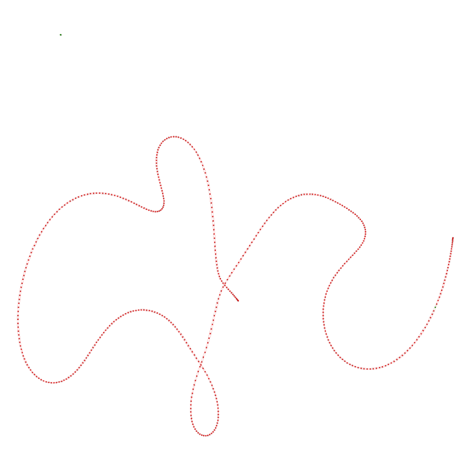	Finished assembly (lacking circularisation)	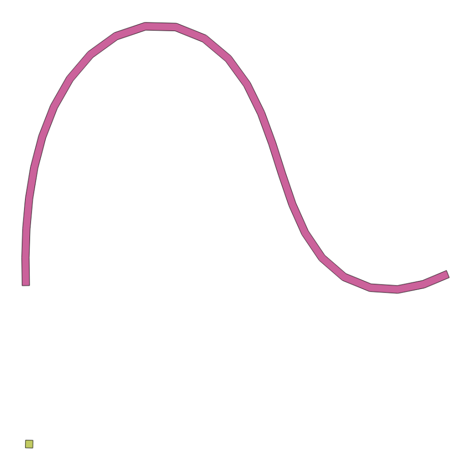
NCTC11106	*Escherichia coli*	52	1	0	4	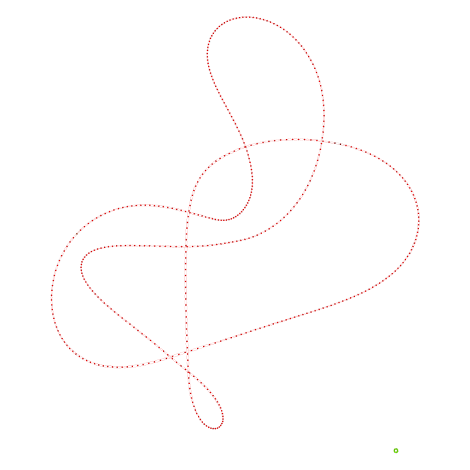	Finished circular assembly	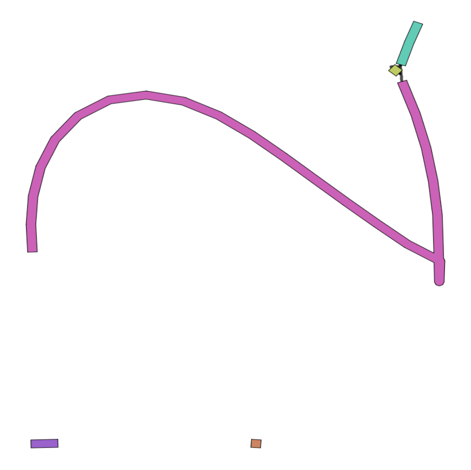
NCTC11107	*Escherichia coli*	34	0	0	3		Mis-assembly/Fragmented	

						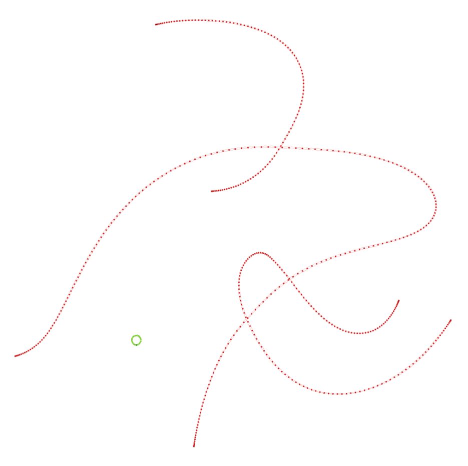		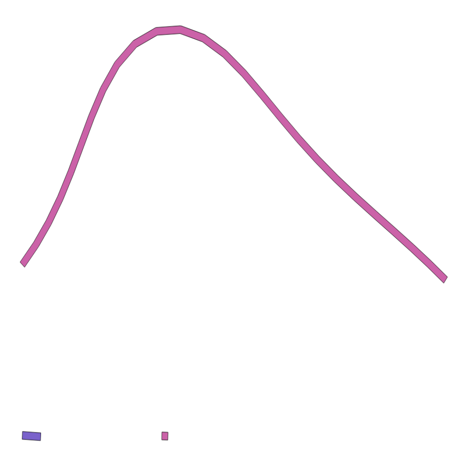
NCTC11108	*Escherichia coli*	77	1	1	0	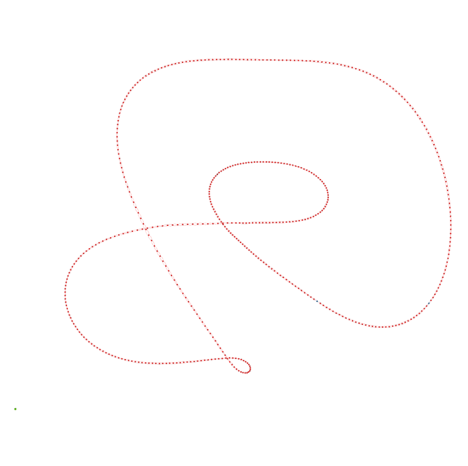	Finished circular assembly	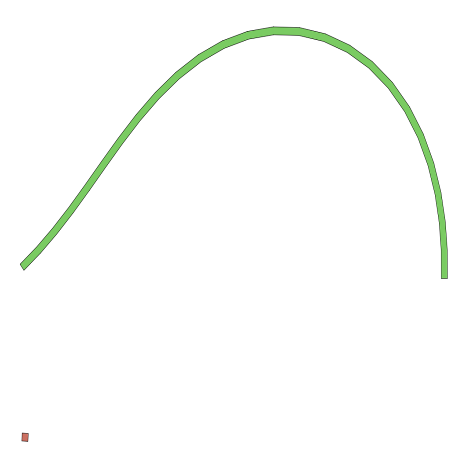
NCTC11109	*Escherichia coli*	72	1	0	1	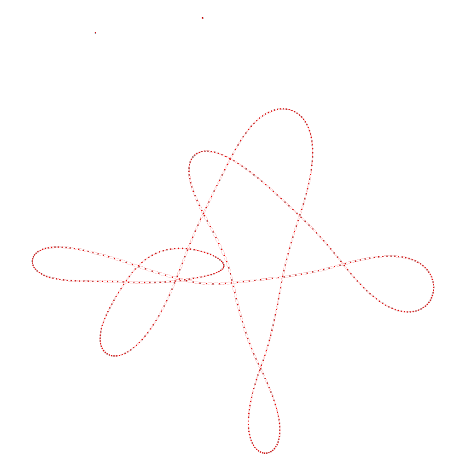	Finished circular assembly	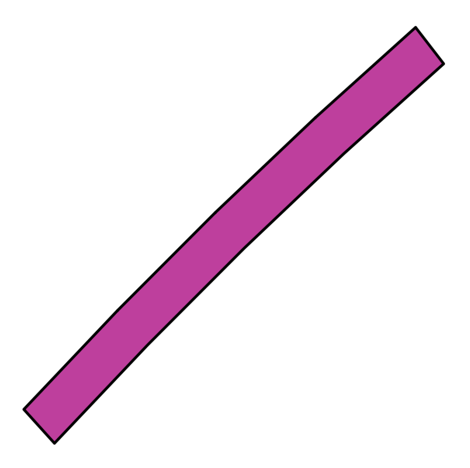
NCTC11111	*Escherichia coli*	58	1	2	0	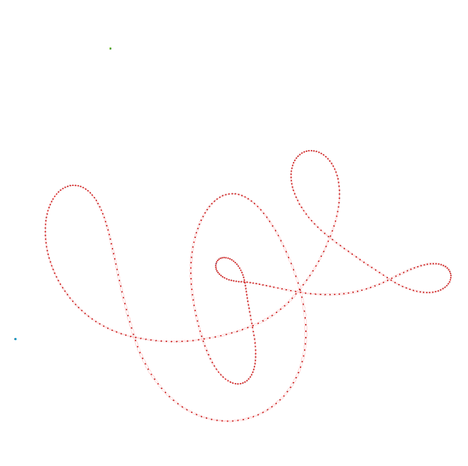	Finished circular assembly	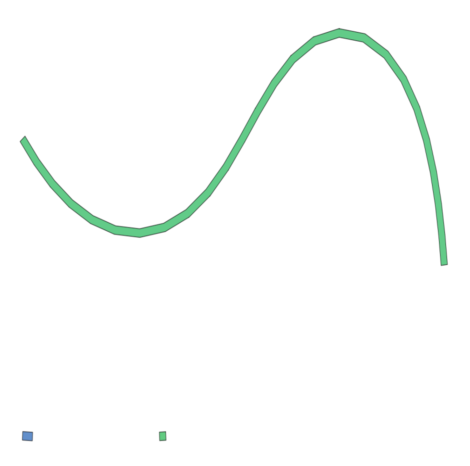
NCTC11112	*Escherichia coli*	0	0	0	2	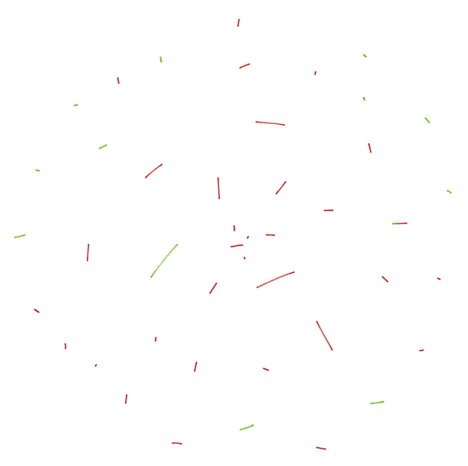	Mis-assembly/Fragmented	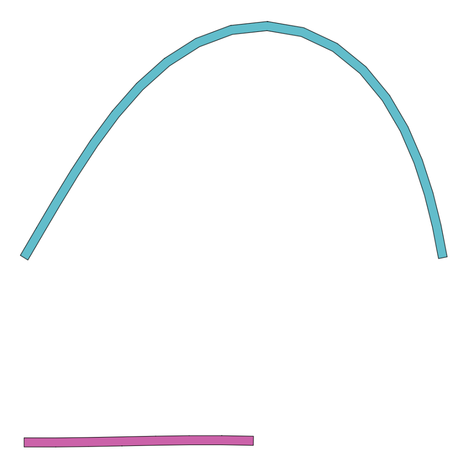
NCTC11113	*Escherichia coli*	55	1	0	0	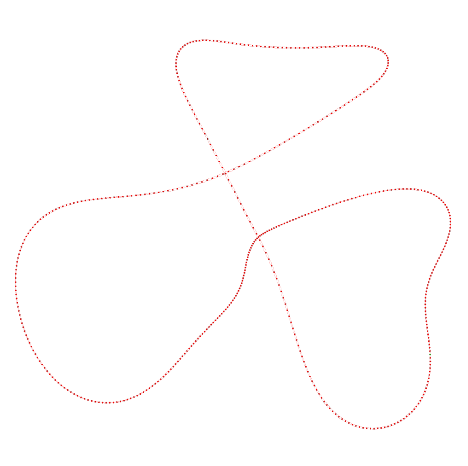	Finished circular assembly	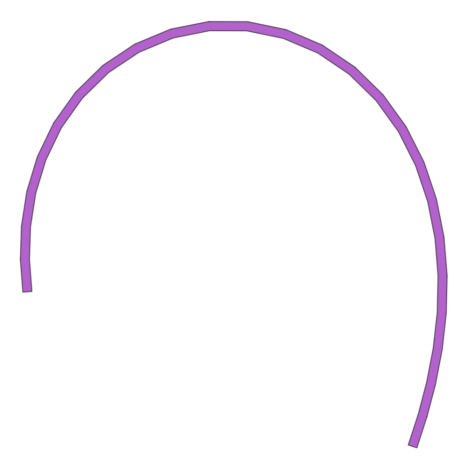
NCTC11114	*Escherichia coli*	71	1	1	4		Finished assembly (lacking circularisation)	

						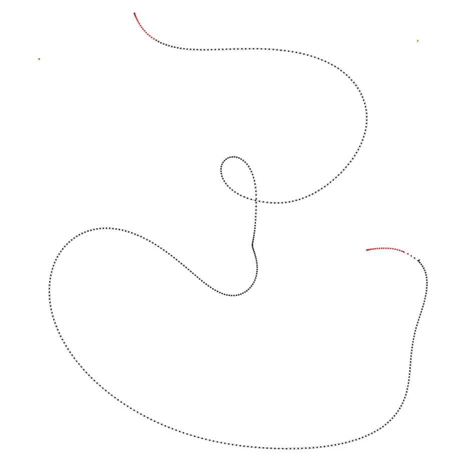		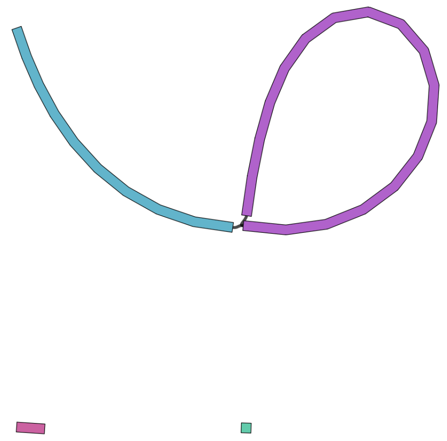
NCTC11115	*Escherichia coli*	42	1	0	1	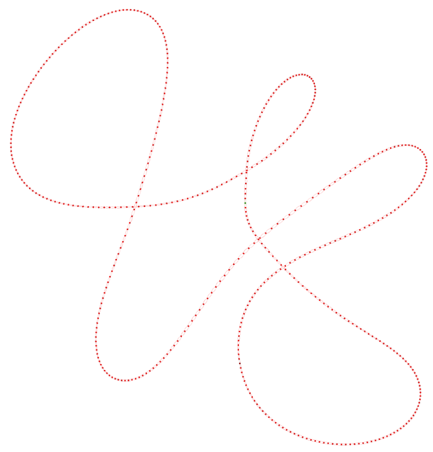	Finished circular assembly	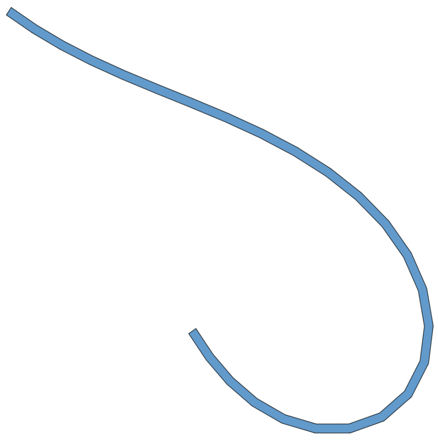
NCTC11116	*Escherichia coli*	56	0	0	4	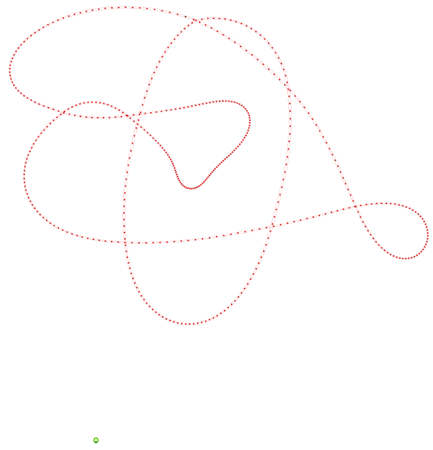	Finished circular assembly	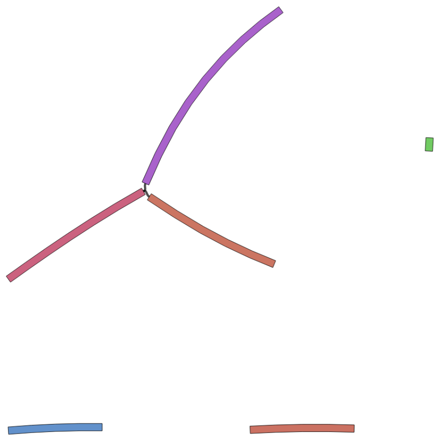
NCTC11117	*Escherichia coli*	46	1	0	1	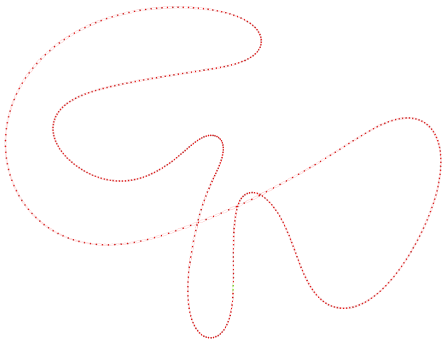	Finished circular assembly	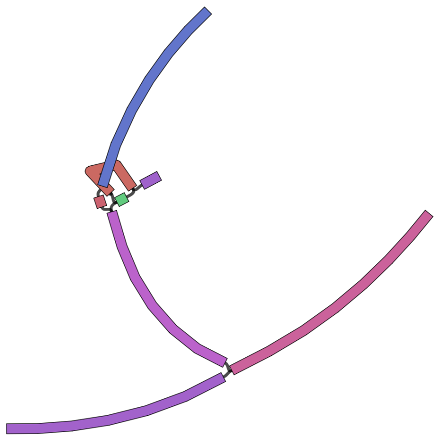
NCTC11118	*Escherichia coli*	59	1	1	0	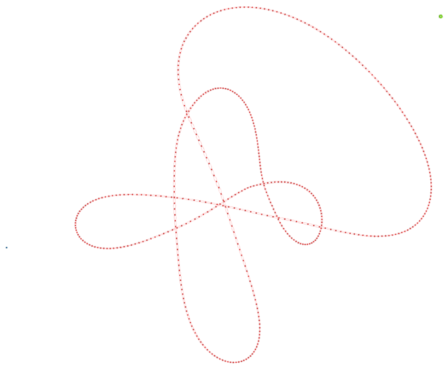	Finished circular assembly	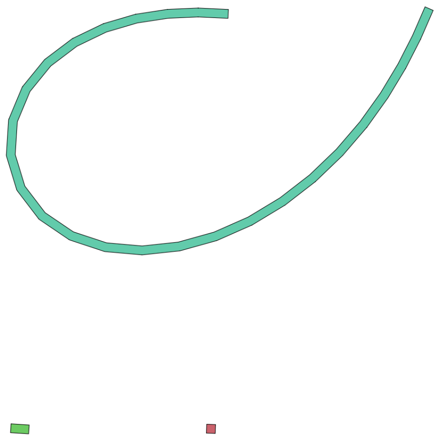
NCTC11119	*Escherichia coli*	61	1	1	3	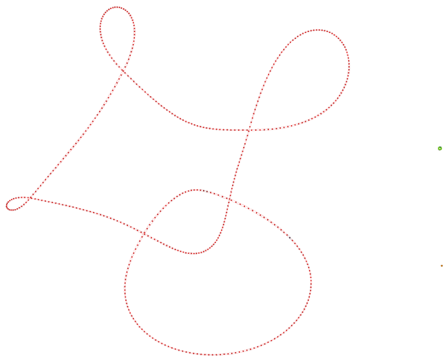	Finished circular assembly	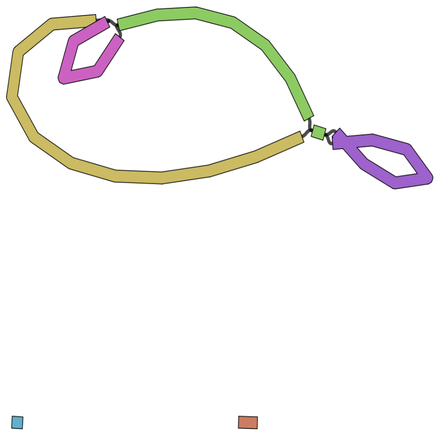
NCTC11120	*Escherichia coli*	35	1	3	5		Finished circular assembly	

						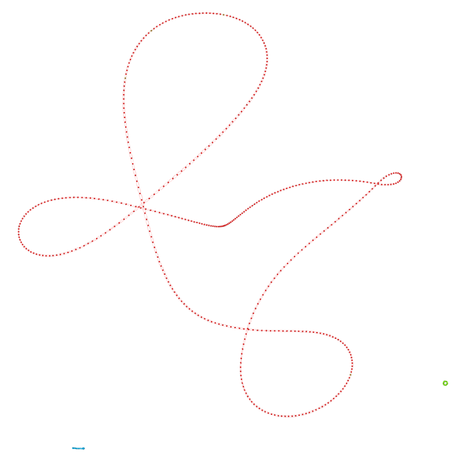		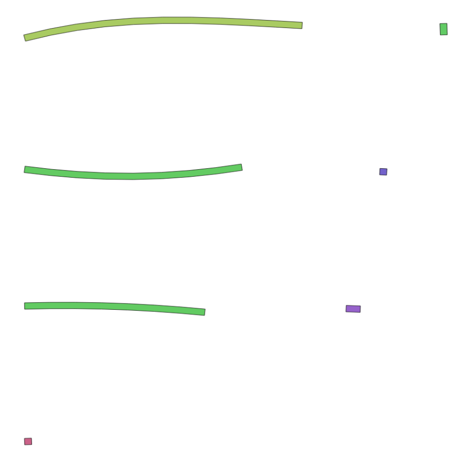
NCTC11121	*Escherichia coli*	53	1	0	1	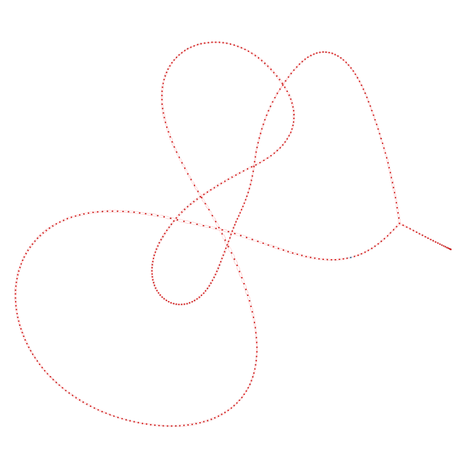	Mis-assembly/Fragmented	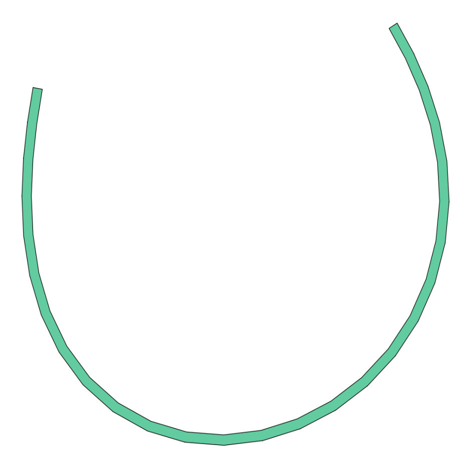
NCTC11122	*Escherichia coli*	80	1	0	1	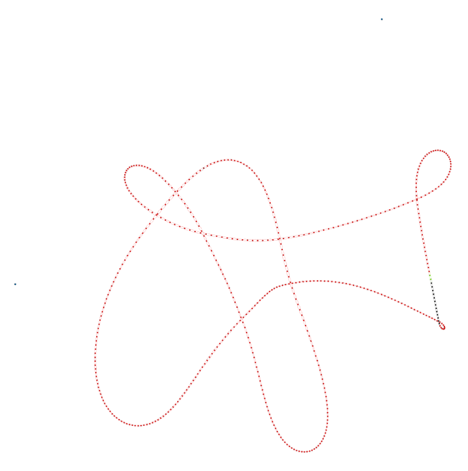	Finished circular assembly	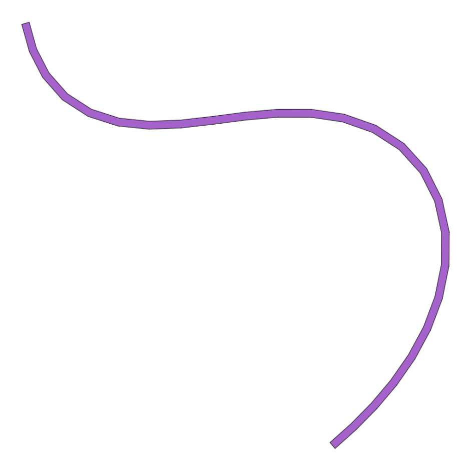
NCTC11123	*Escherichia coli*	83	0	0	10	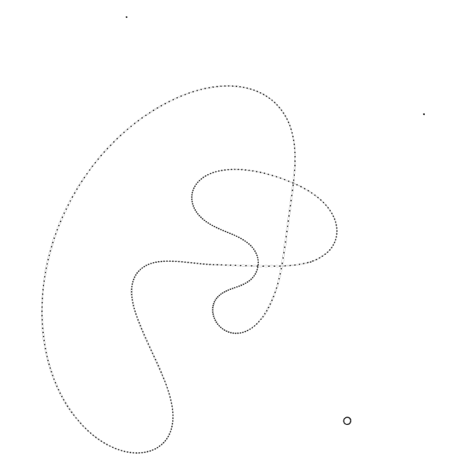	Finished circular assembly	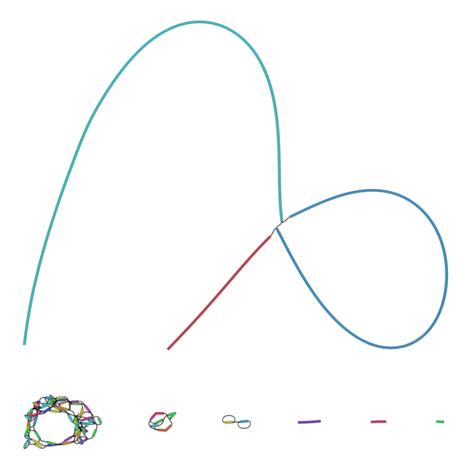
NCTC11124	*Escherichia coli*	39	1	1	0	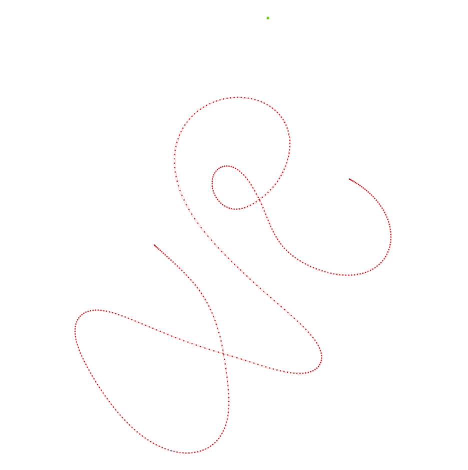	Finished assembly (lacking circularisation)	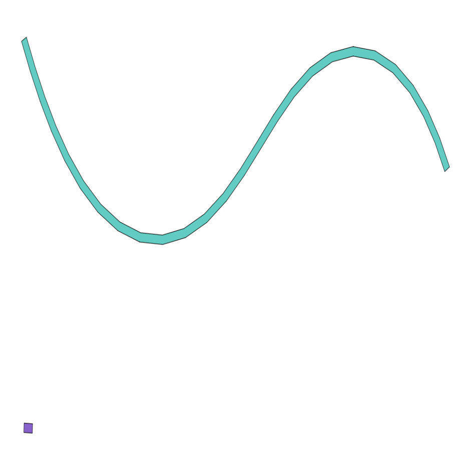
NCTC11125	*Escherichia coli*	64	1	0	2	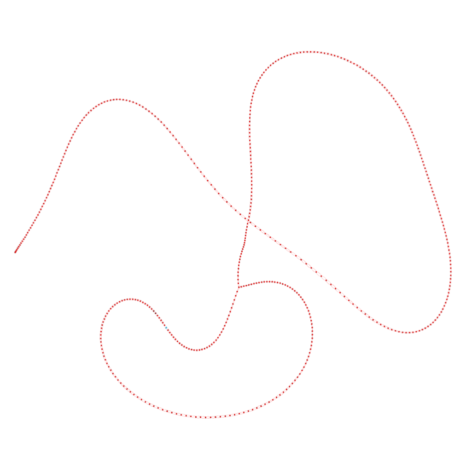	Mis-assembly/Fragmented	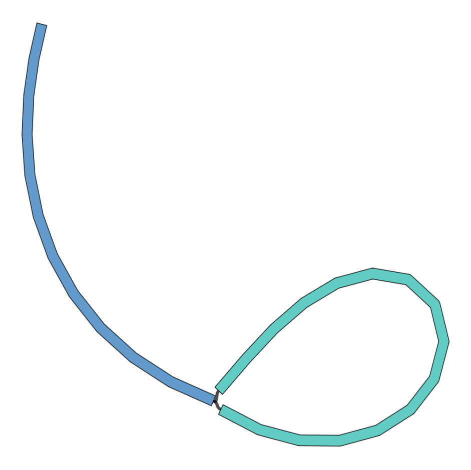
NCTC11126	*Escherichia coli*	50	2	0	0		Finished assembly (lacking circularisation)	

						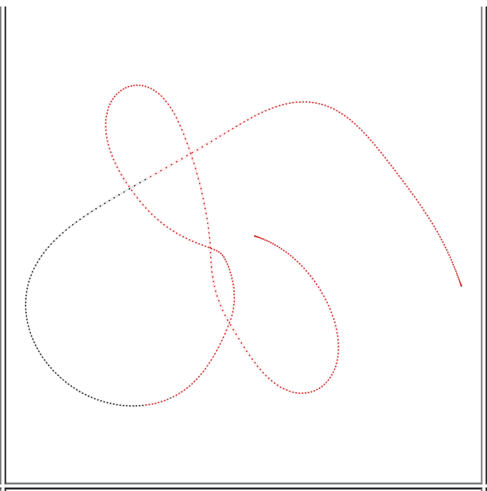		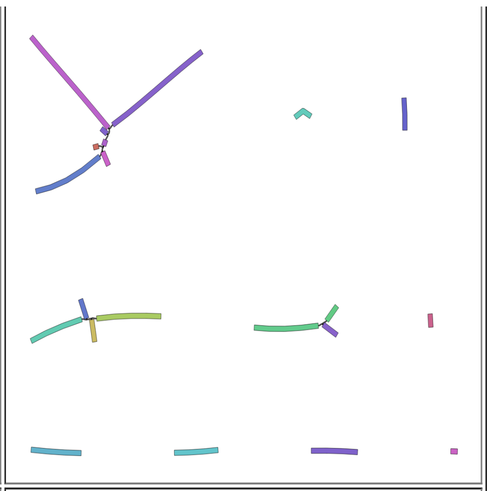
NCTC11127	*Escherichia coli*	64	0	0	4	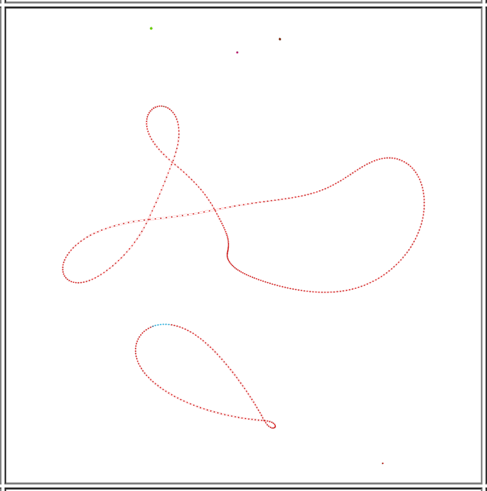	Mis-assembly/Fragmented	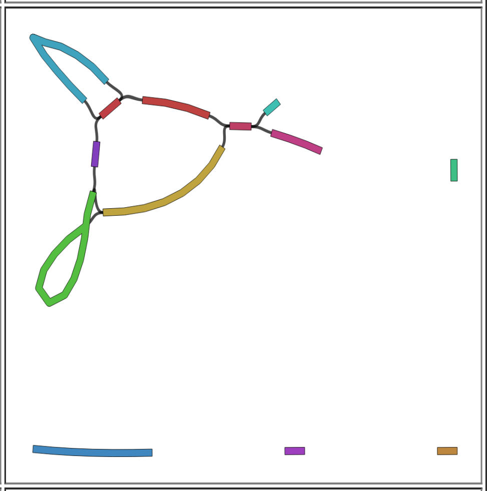
NCTC11128	*Escherichia coli*	63	1	1	2	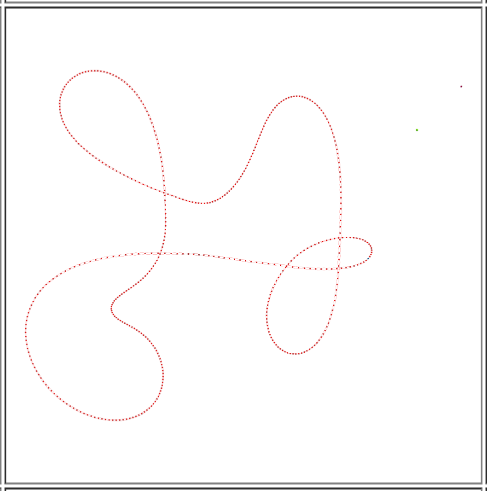	Finished circular assembly	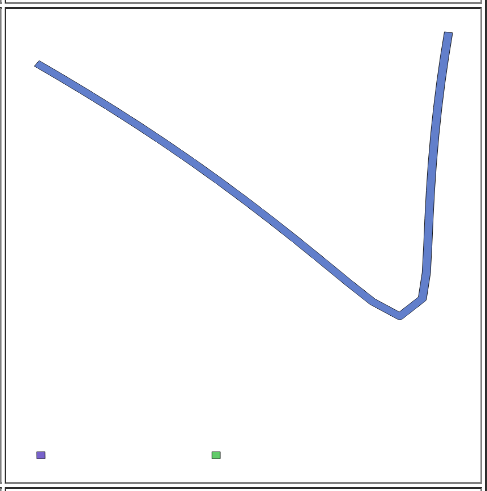
NCTC11129	*Escherichia coli*	70	1	0	0	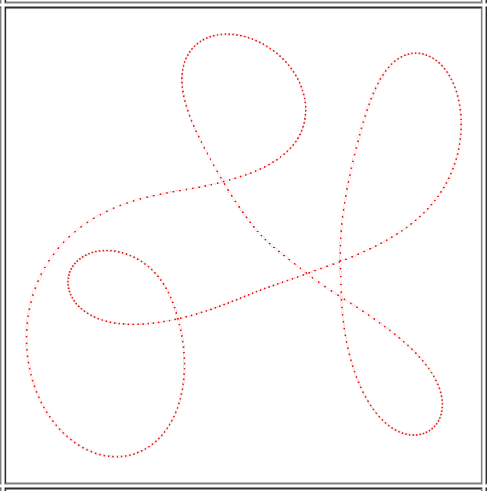	Finished circular assembly	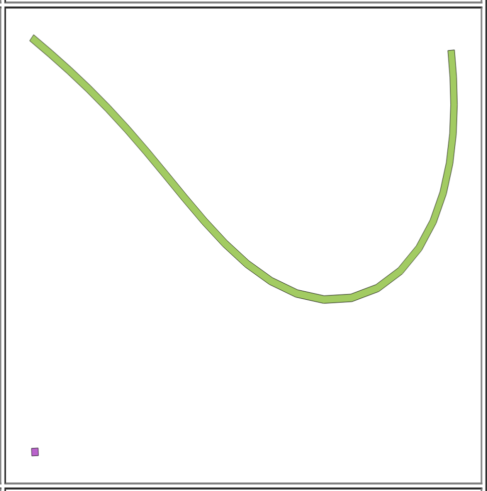
NCTC11130	*Escherichia coli*	87	0	0	2	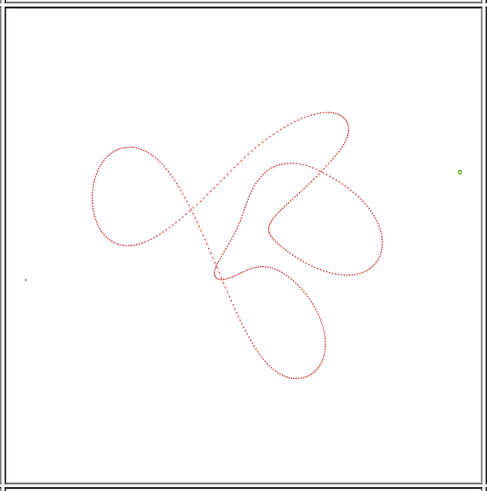	Finished circular assembly	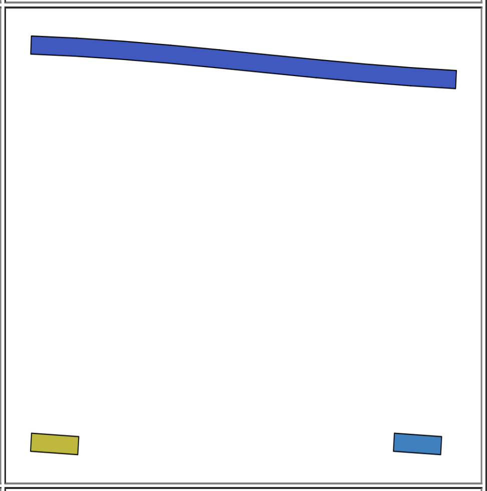
NCTC11131	*Escherichia coli*	63	1	0	2	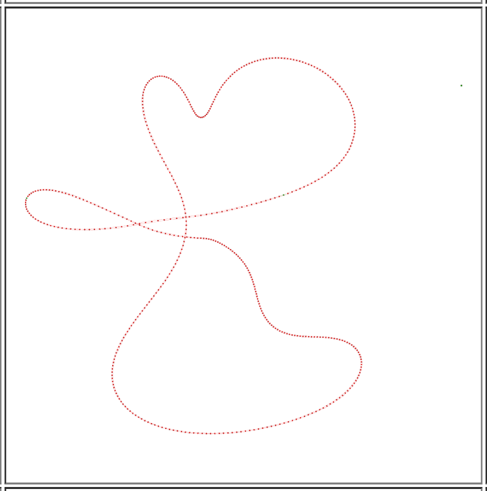	Finished circular assembly	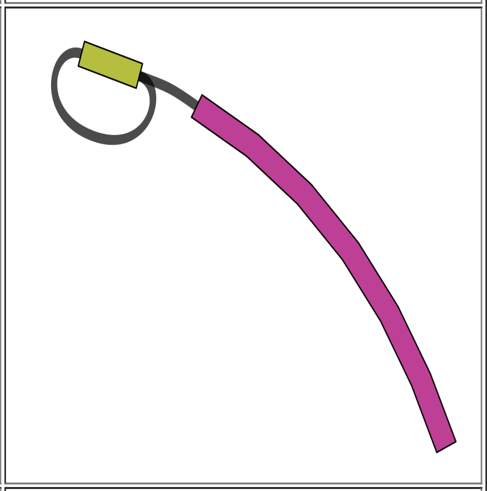
NCTC11132	*Escherichia coli*	65	0	0	4		Mis-assembly/Fragmented	

						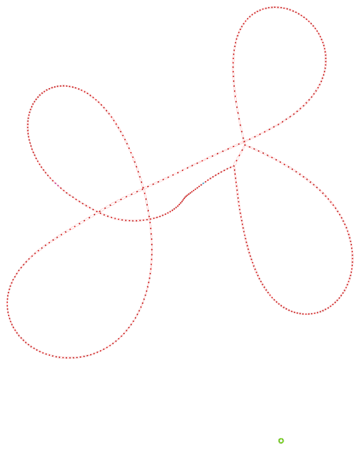		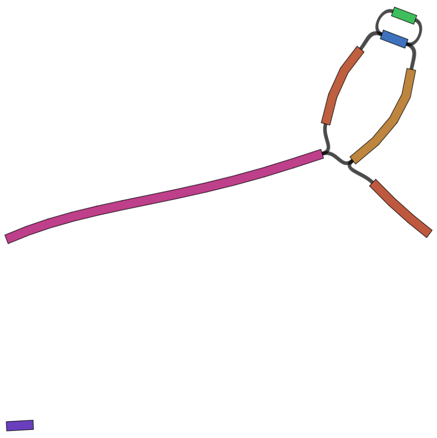
NCTC11134	*Nocardia farcinica*	52	3	2	0	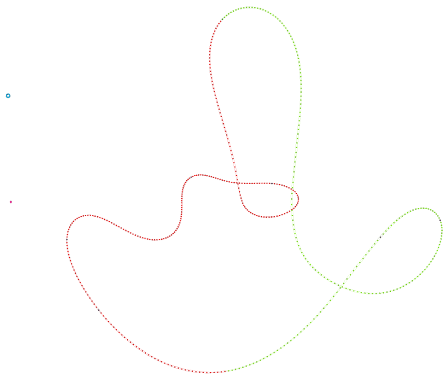	Finished circular assembly	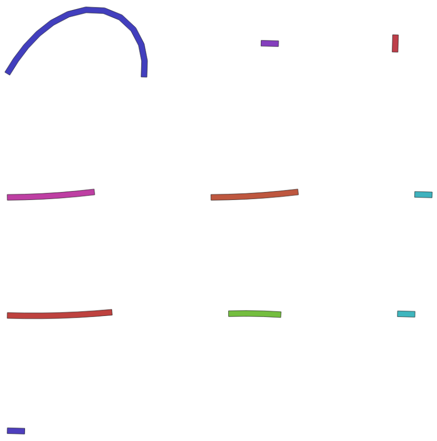
NCTC11150	*Staphylococcus aureus*	31	1	1	1	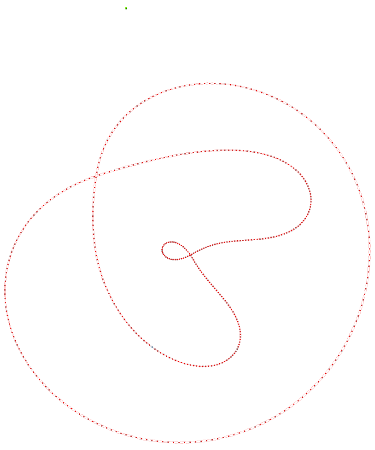	Finished circular assembly	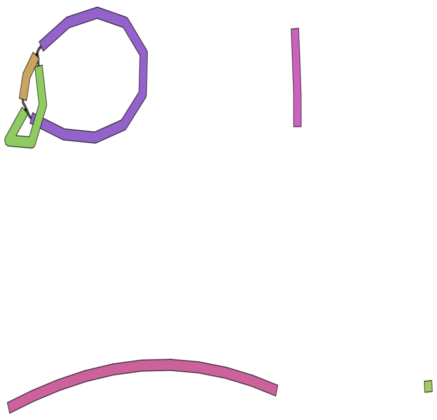
NCTC11151	*Escherichia coli*	72	1	0	0	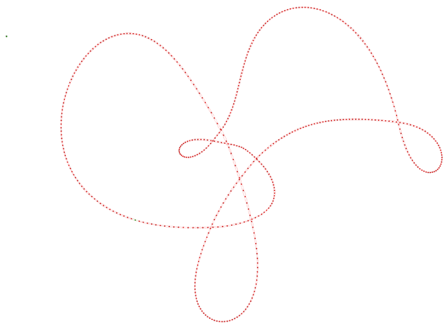	Finished circular assembly	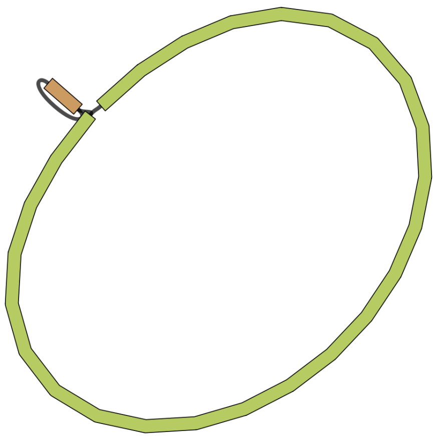
NCTC11169	*Streptococcus milleri*	100	Pending	Pending	Pending	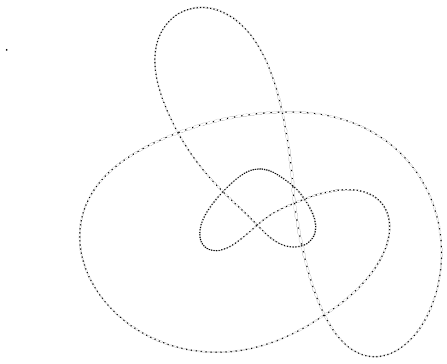	Finished circular assembly	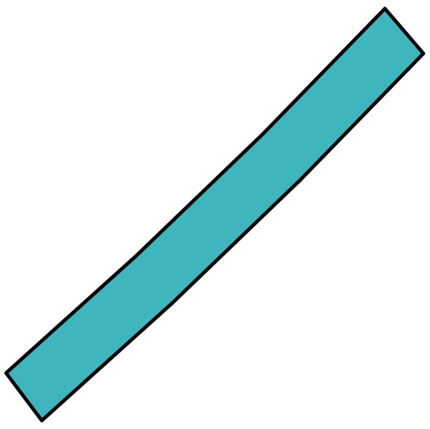
NCTC11186	*Escherichia coli*	85	1	1	3	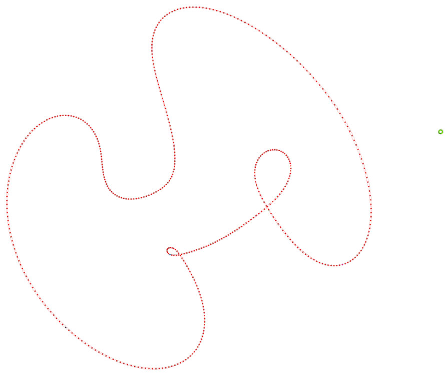	Finished circular assembly	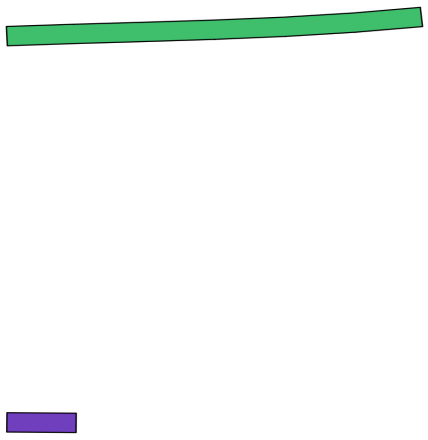
NCTC11189	*Streptococcus viridans*	79	0	0	4		Finished assembly (lacking circularisation)	

						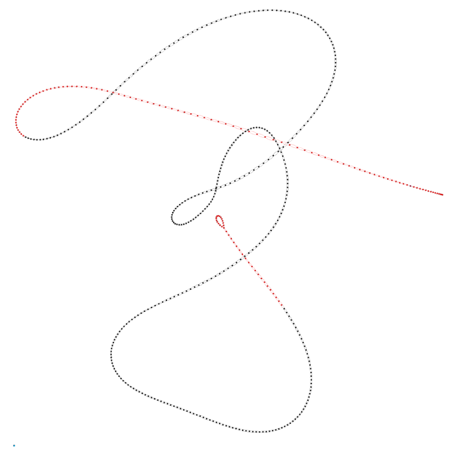		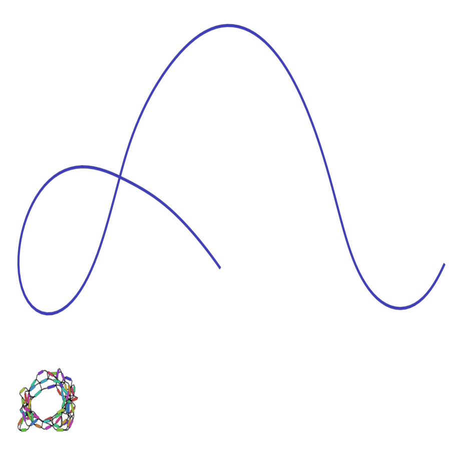
NCTC11192	*Legionella pneumophila*	38	1	0	1	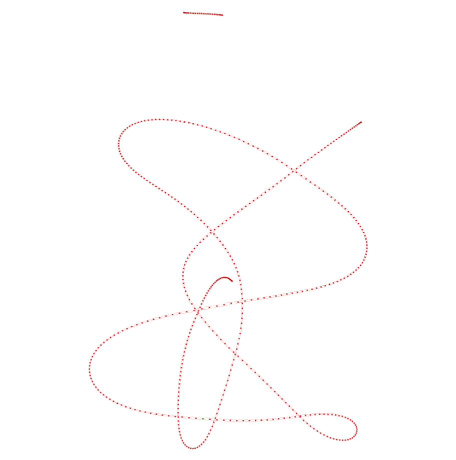	Finished assembly (lacking circularisation)	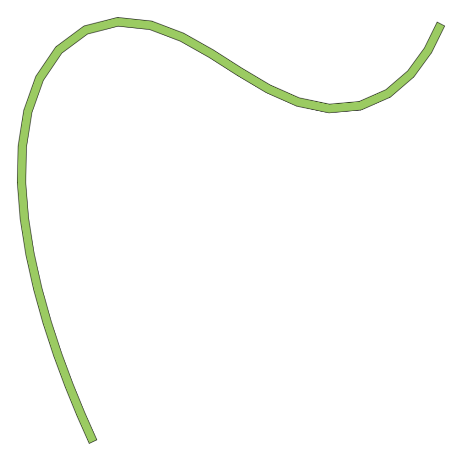
NCTC11214	*Serratia odorifera*	37	1	0	0	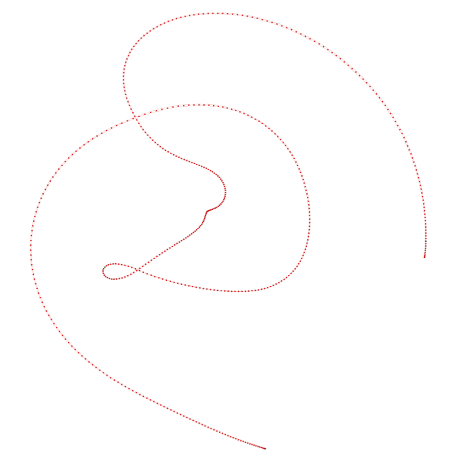	Finished assembly (lacking circularisation)	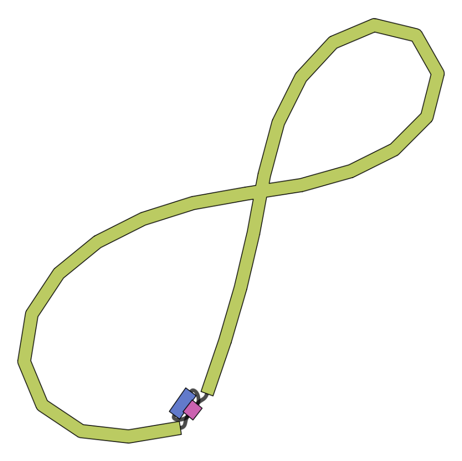
NCTC11228	*Klebsiella pneumoniae*	76	0	0	4	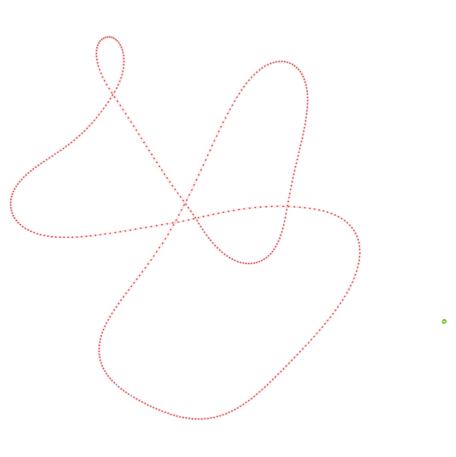	Finished circular assembly	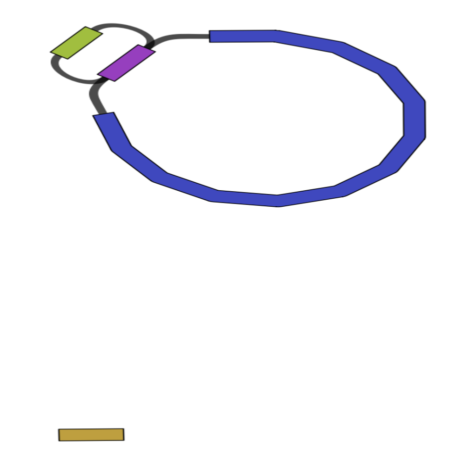
NCTC11242	*Streptococcus agalactiae*	10	Pending	Pending	Pending	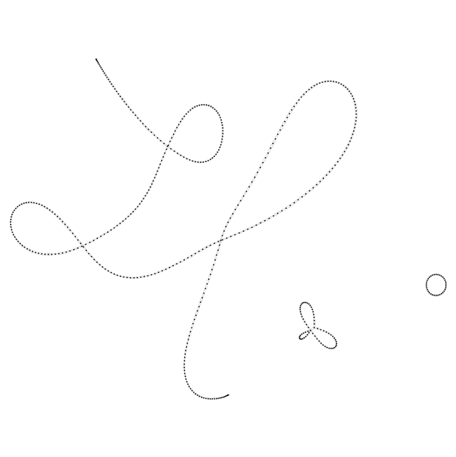	Finished assembly (lacking circularisation)	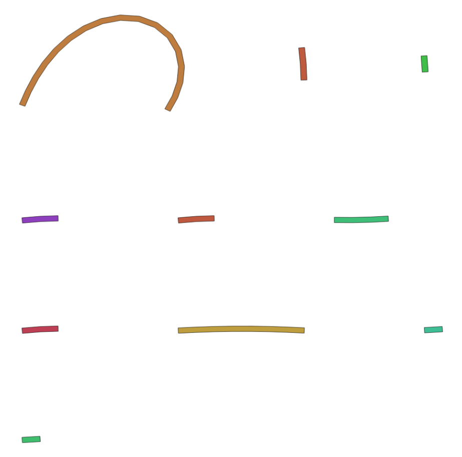
NCTC11320	*Staphylococcus hominis*	90	0	1	1	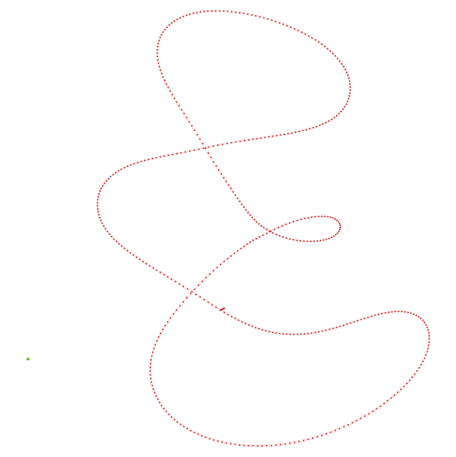	Finished circular assembly	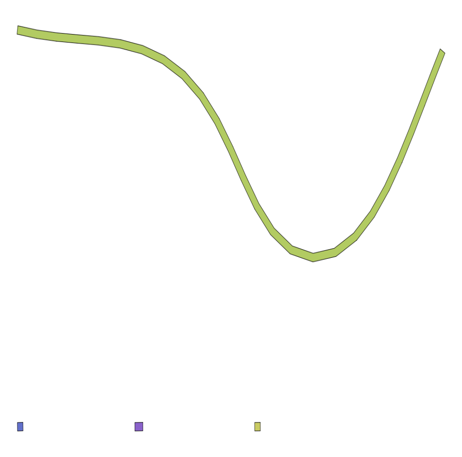
NCTC11327	*Vibrio fluvialis*	83	1	1	0		Finished circular assembly	

						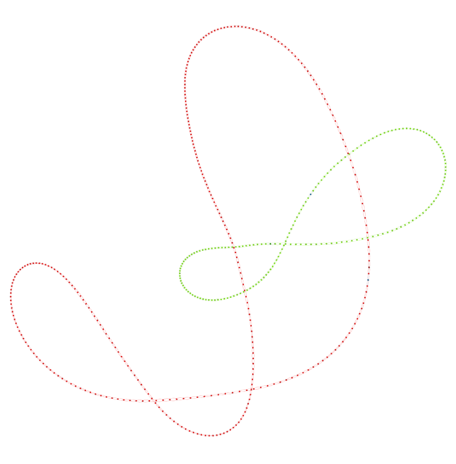		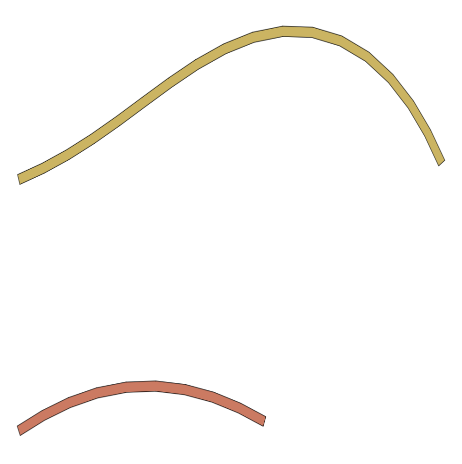
NCTC11341	*Escherichia coli*	40	0	0	4	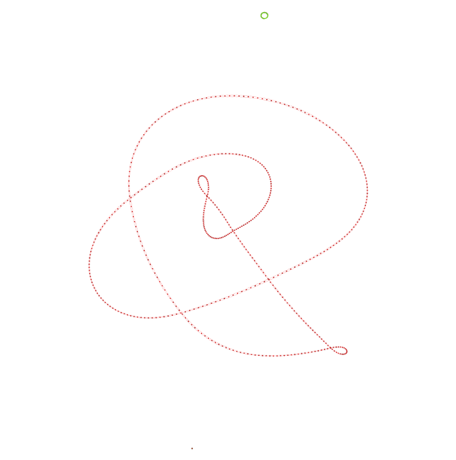	Finished circular assembly	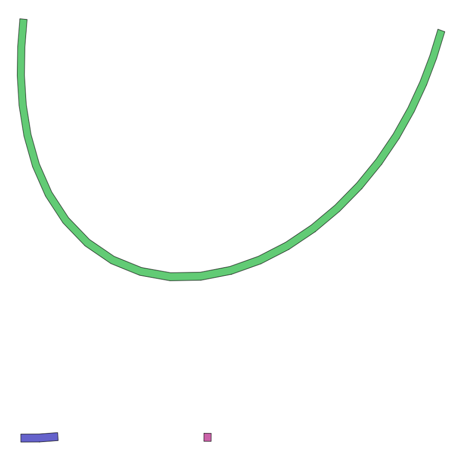
NCTC11343	*Sphingobacterium multivorum*	22	Pending	Pending	Pending	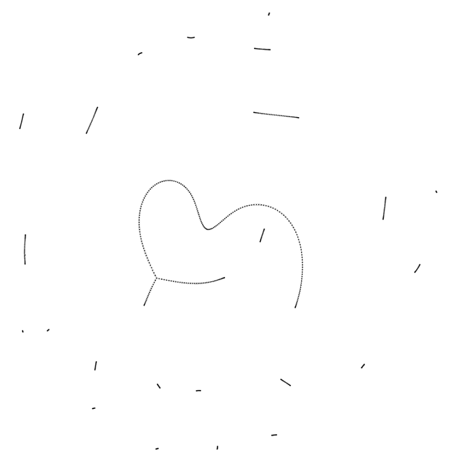	Mis-assembly/Fragmented	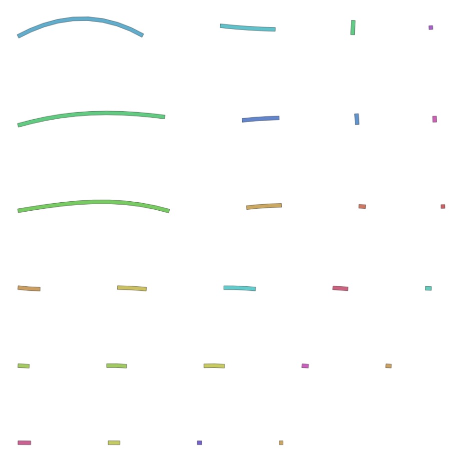
NCTC11351	*Campylobacter jejuni*	61	1	0	0	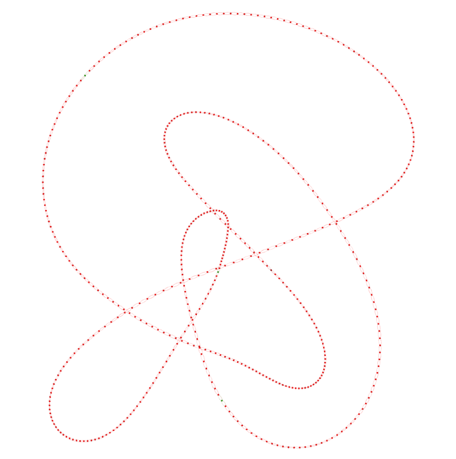	Finished circular assembly	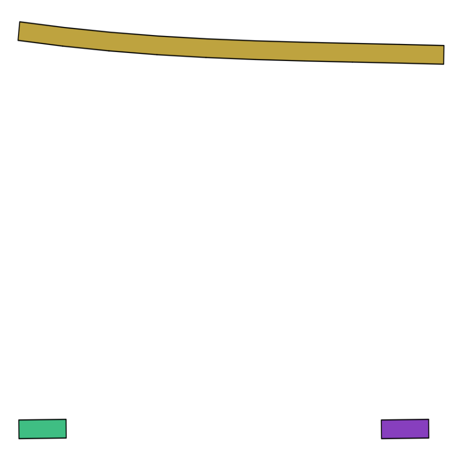
NCTC11352	*Campylobacter lari*	30	Pending	Pending	Pending	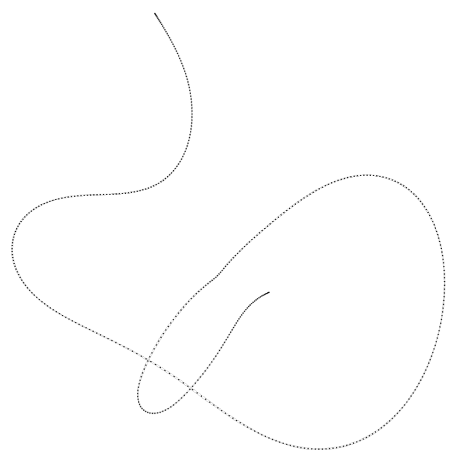	Finished assembly (lacking circularisation)	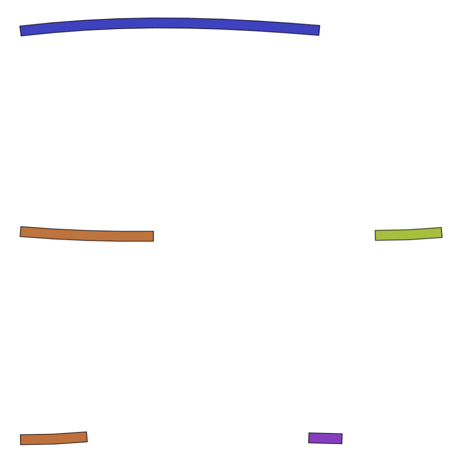
NCTC11355	*Klebsiella sp*	42	0	0	1	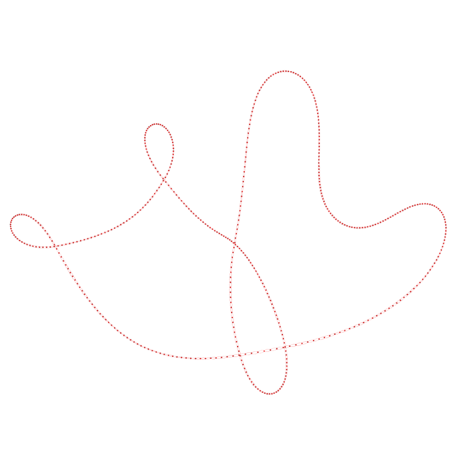	Finished circular assembly	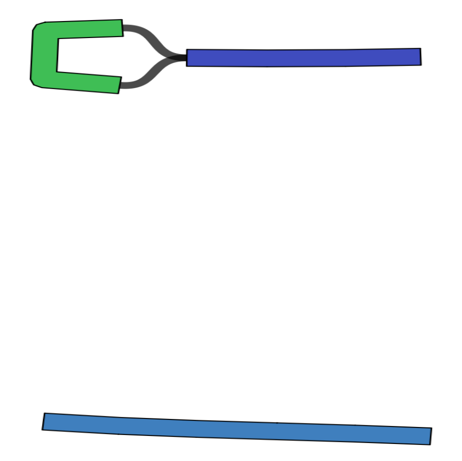
NCTC11356	*Klebsiella sp*	70	1	0	0		Finished assembly (lacking circularisation)	

						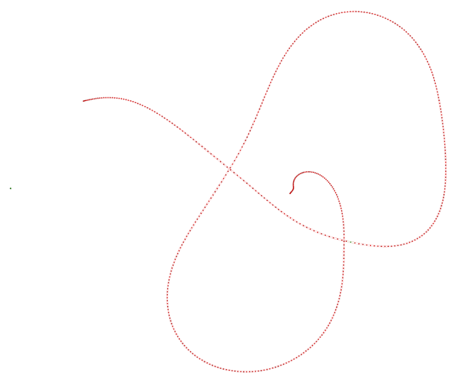		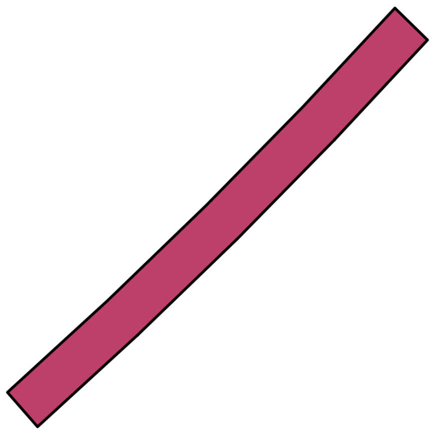
NCTC11357	*Klebsiella sp*	77	1	0	0	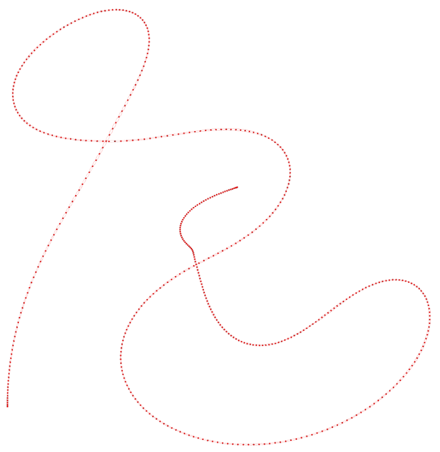	Finished assembly (lacking circularisation)	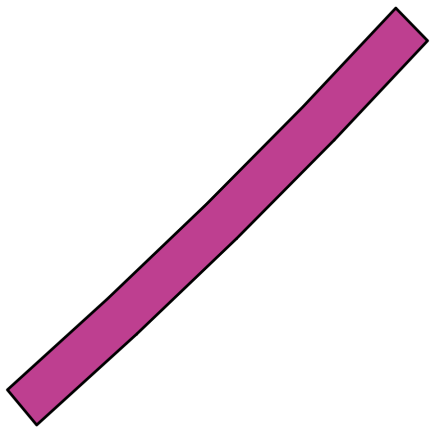
NCTC11358	*Klebsiella sp*	45	1	1	1	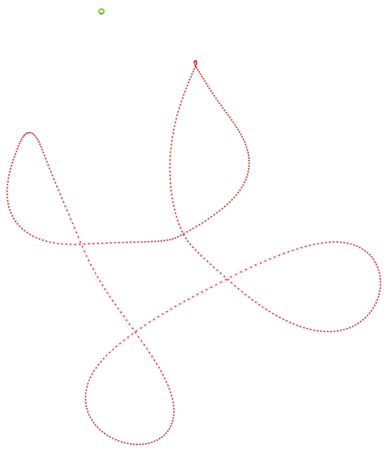	Finished circular assembly	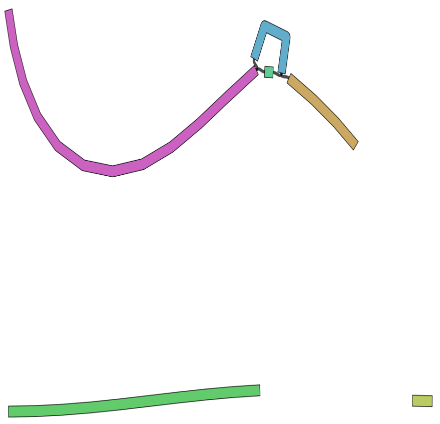
NCTC11359	*Klebsiella sp*	61	1	0	0	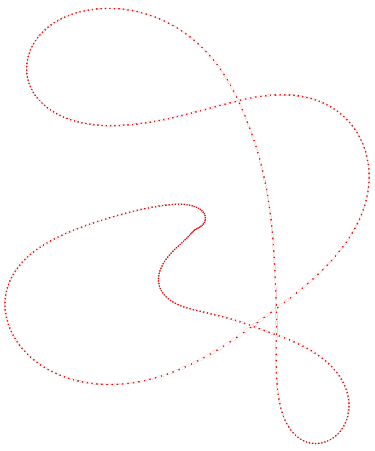	Finished circular assembly	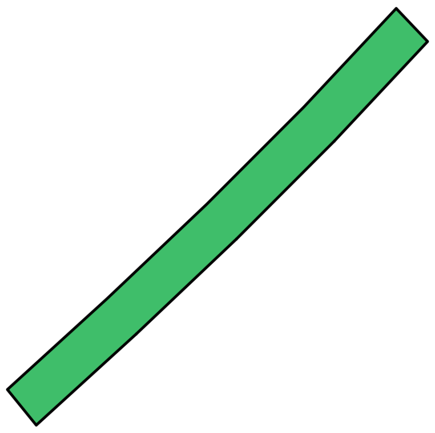
NCTC11360	*Streptococcus agalactiae*	3	Pending	Pending	Pending	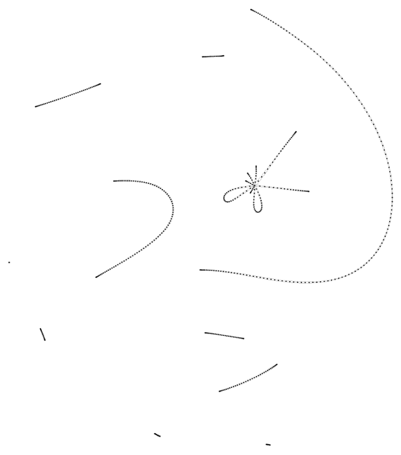	Mis-assembly/Fragmented	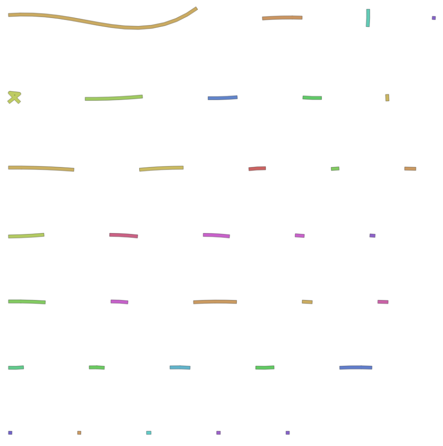
NCTC11366	*Campylobacter coli*	135	Pending	Pending	Pending	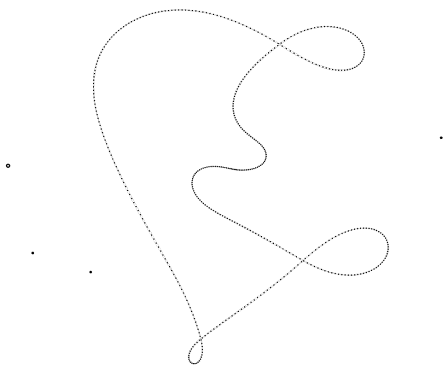	Finished circular assembly	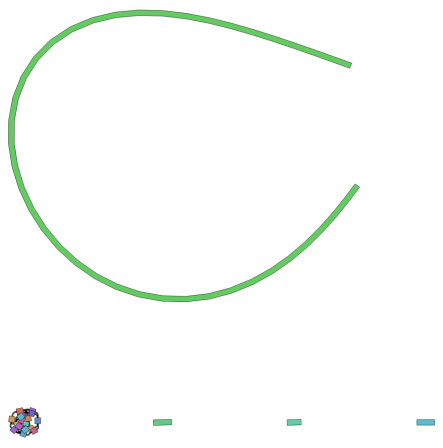
NCTC11383	*Actinobacillus pleuropneumoniae*	6	Pending	Pending	Pending		Mis-assembly/Fragmented	

						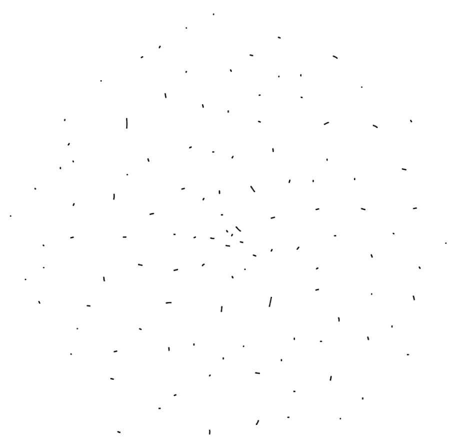		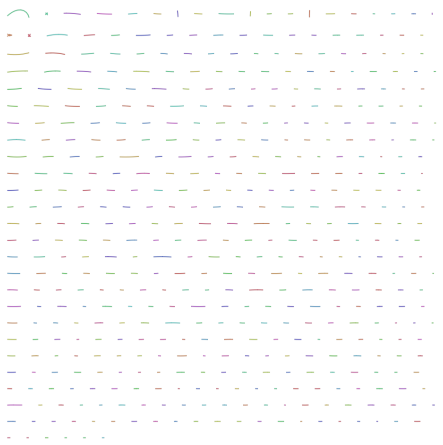
NCTC11384	*Actinobacillus pleuropneumoniae*	69	Pending	Pending	Pending	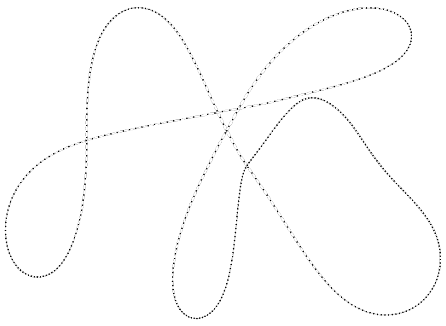	Finished circular assembly	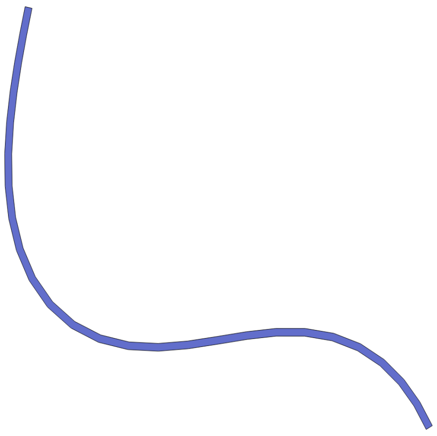
NCTC11388	*Sphingobacterium spiritivorum*	39	0	0	2	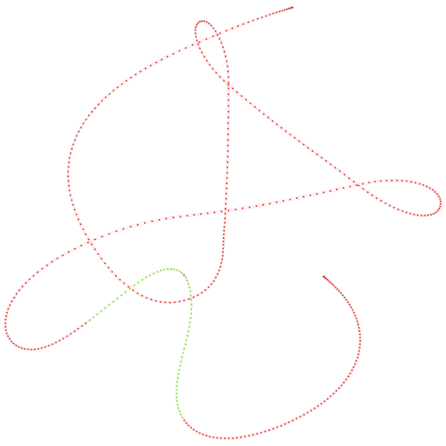	Finished assembly (lacking circularisation)	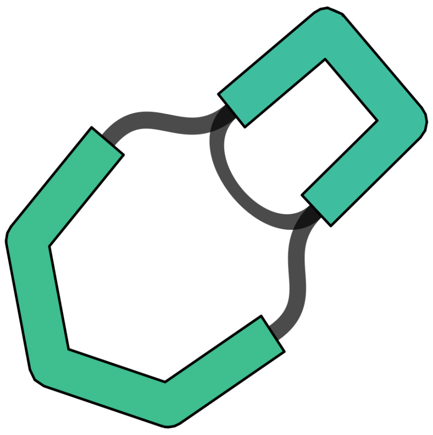
NCTC11394	*Haemophilus influenzae*	85	1	0	0	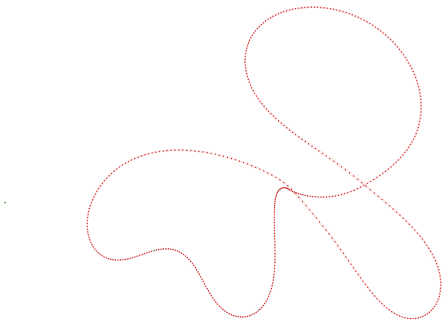	Finished circular assembly	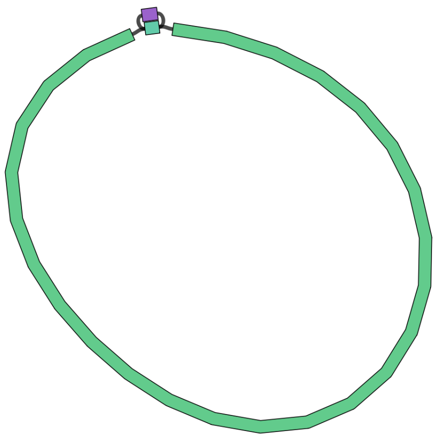
NCTC11397	*Corynebacterium diphtheriae*	59	1	0	0	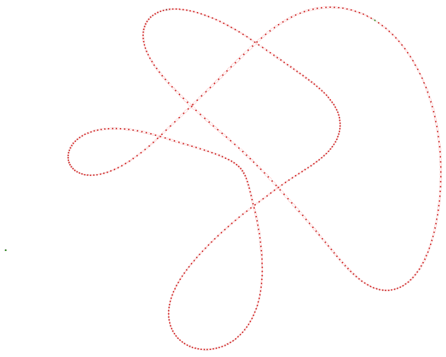	Finished circular assembly	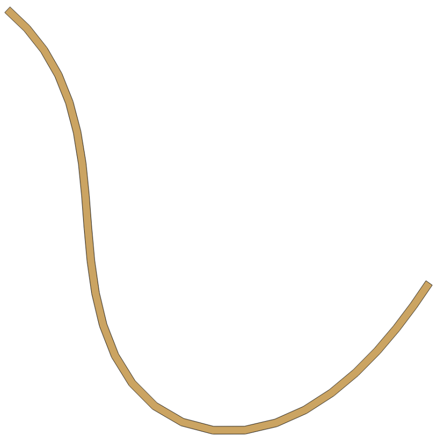
NCTC11407	*Actinobacillus pleuropneumoniae*	97	Pending	Pending	Pending	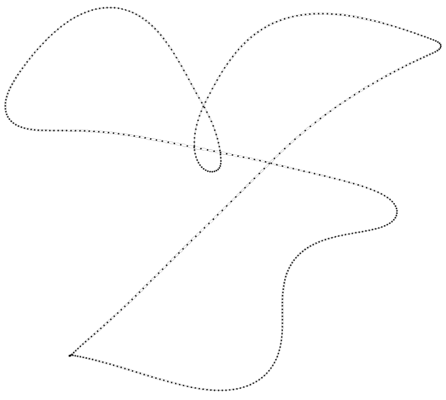	Finished circular assembly	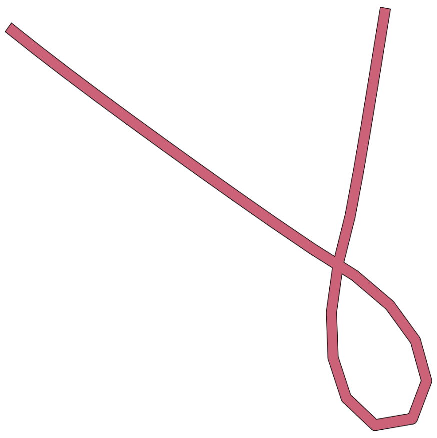
NCTC11429	*Sphingobacterium thalpophilum*	0	Pending	Pending	Pending		Mis-assembly/Fragmented	

NCTC11432	*Chryseobacterium gleum*	41	Pending	Pending	Pending	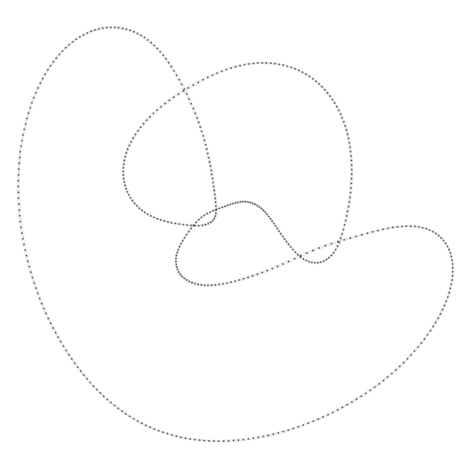	Finished circular assembly	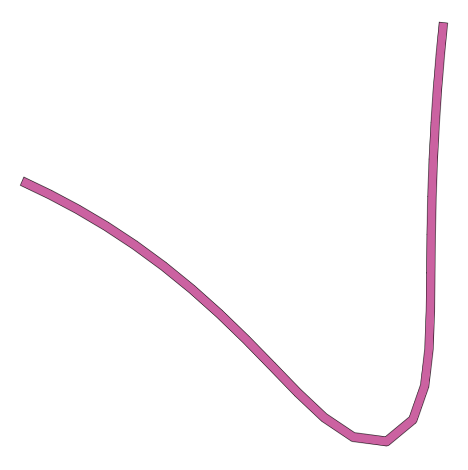
NCTC11434	*Pluralibacter gergoviae*	46	1	1	2	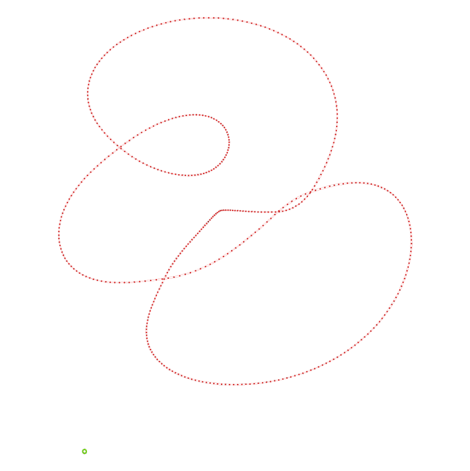	Finished circular assembly	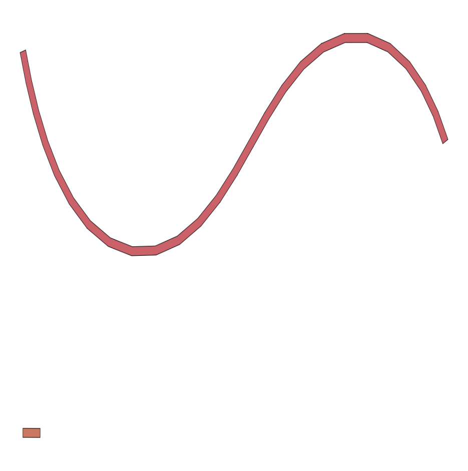
NCTC11435	*Vibrio mimicus*	60	Pending	Pending	Pending	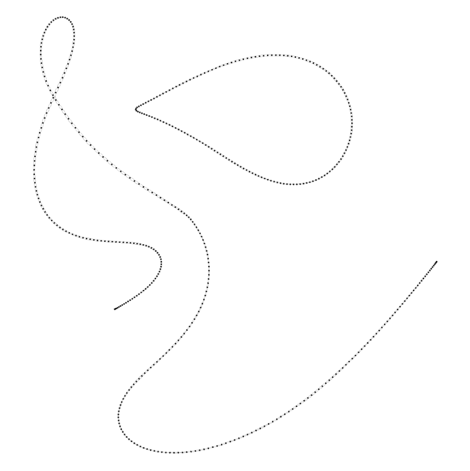	Mis-assembly	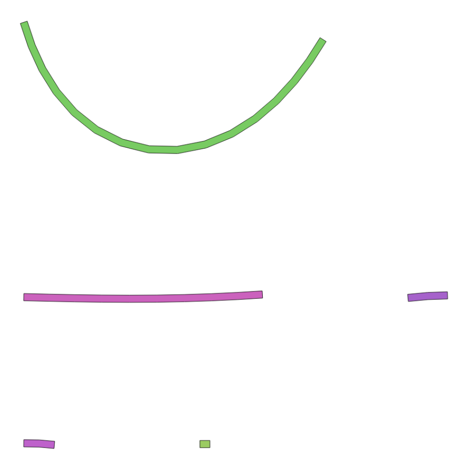
NCTC11436	*Streptococcus equinus*	137	0	0	2	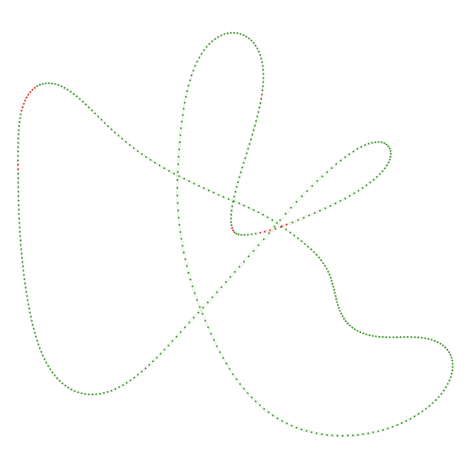	Finished circular assembly	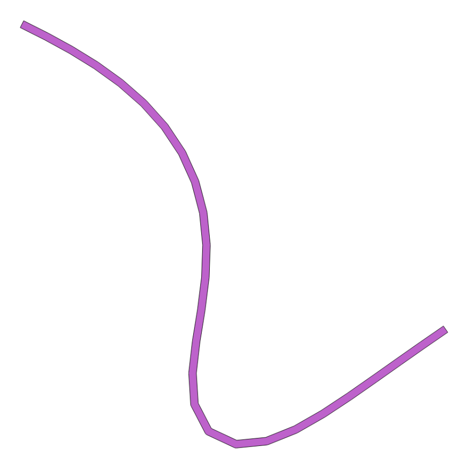
NCTC11437	*Campylobacter coli*	226	1	1	0	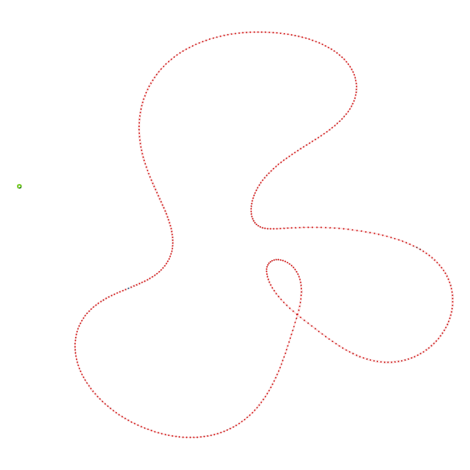	Finished circular assembly	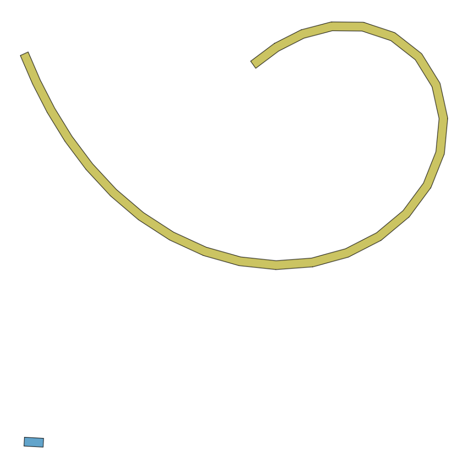
NCTC11440	*Pseudomonas aeruginosa*	44	0	0	4		Finished assembly (lacking circularisation)	

						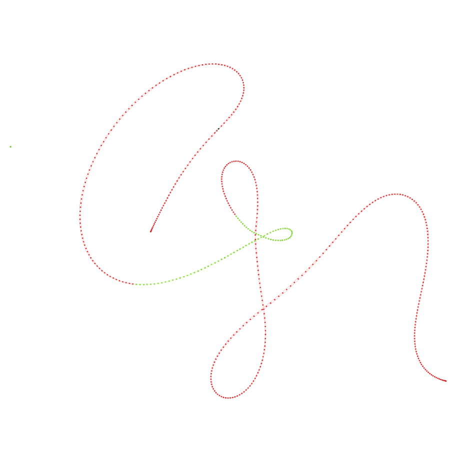		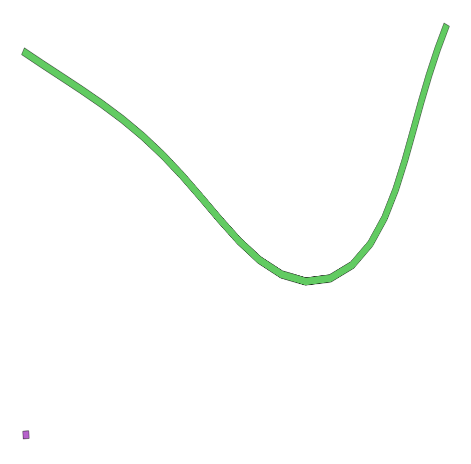
NCTC11466	*Cedecea lapagei*	64	1	0	0	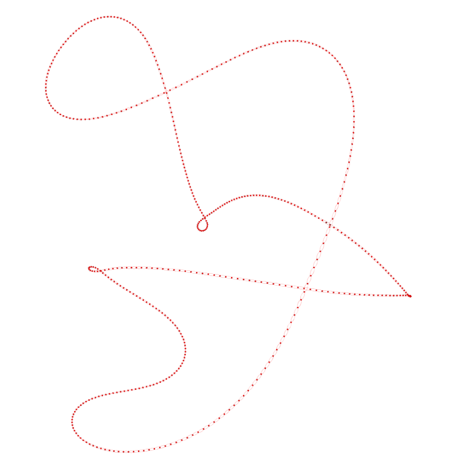	Finished circular assembly	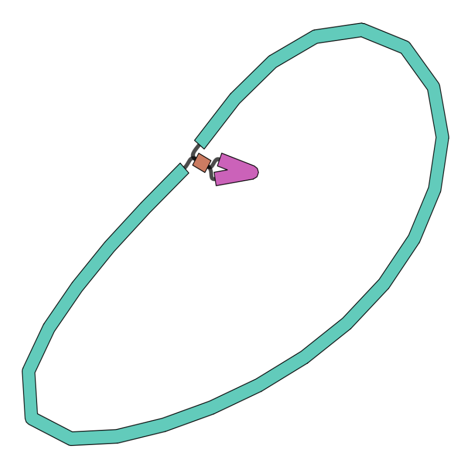
NCTC11467	*Cronobacter sakazakii*	48	4	3	0	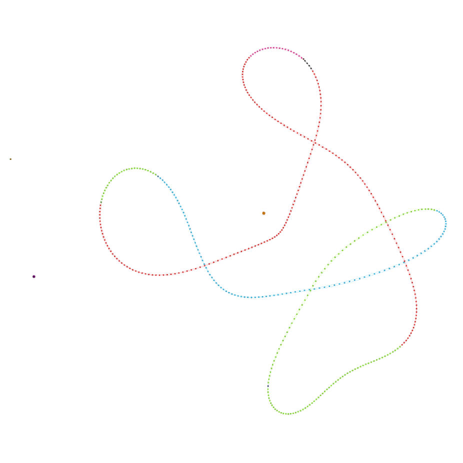	Finished circular assembly	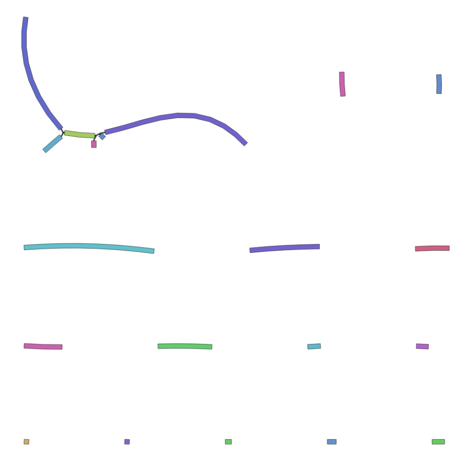
NCTC11468	*Tatumella ptyseos*	78	1	0	0	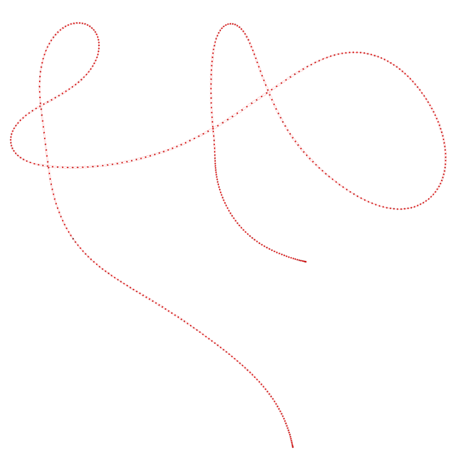	Finished assembly (lacking circularisation)	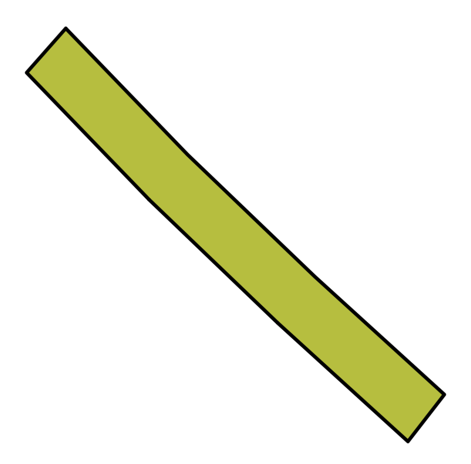
NCTC11469	*Yersinia intermedia*	44	1	0	0	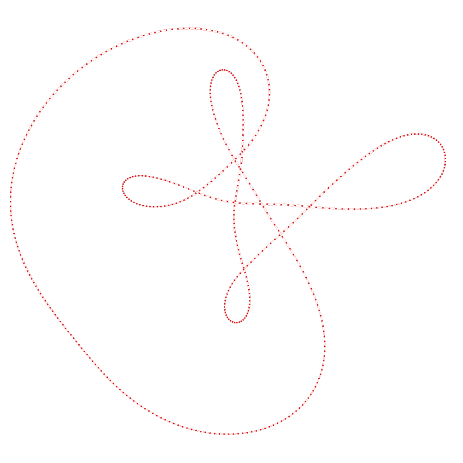	Finished circular assembly	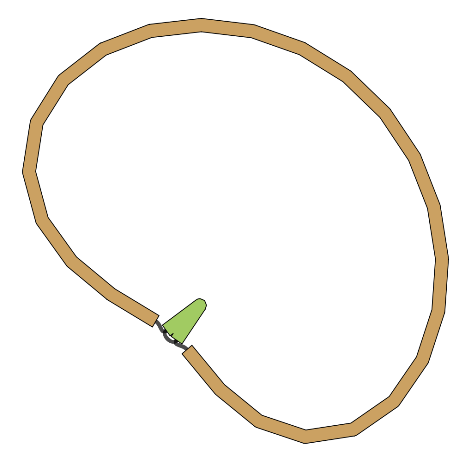
NCTC11470	*Yersinia frederiksenii*	45	1	0	0	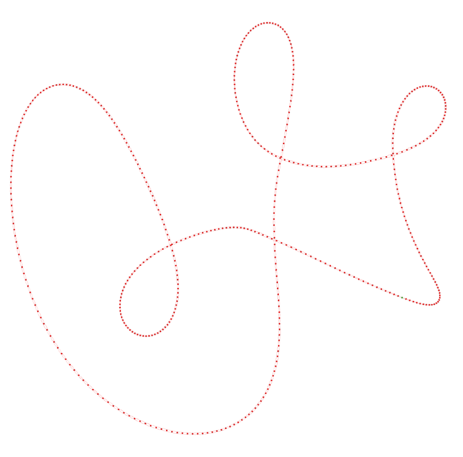	Finished circular assembly	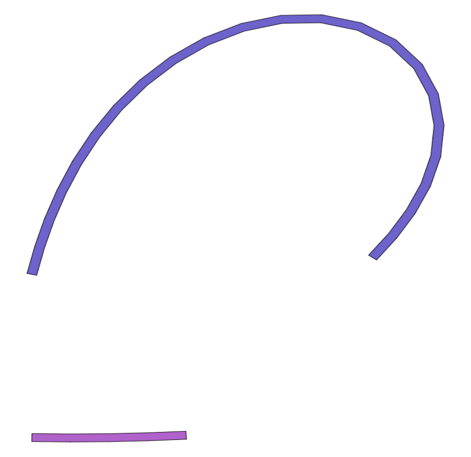
NCTC11471	*Yersinia kristensenii*	73	1	0	0		Finished circular assembly	

						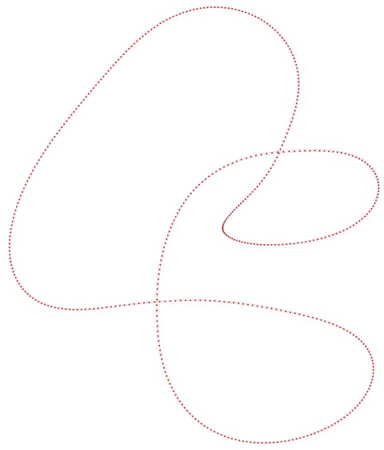		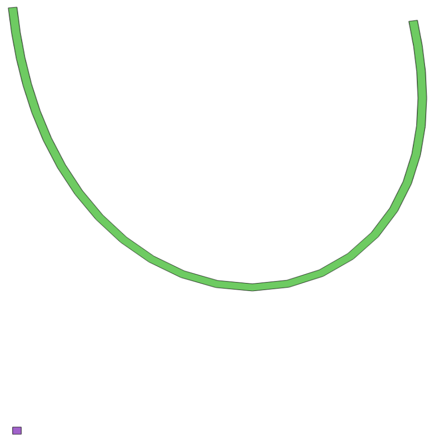
NCTC11472	*Escherichia coli*	56	1	1	2	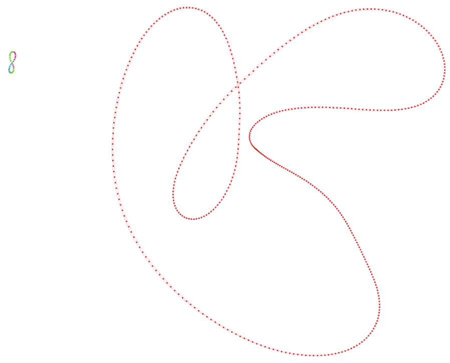	Finished circular assembly	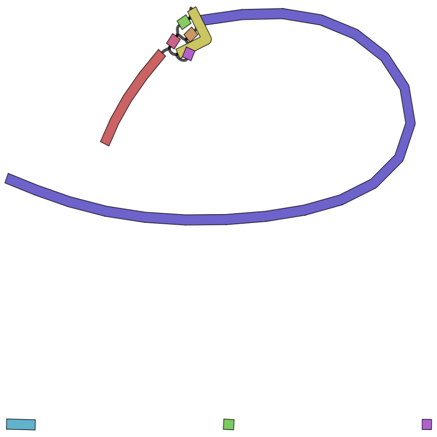
NCTC11473	*Escherichia coli*	0	1	1	1	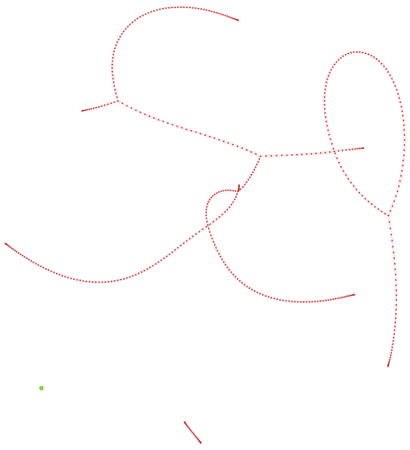	Mis-assembly/Fragmented	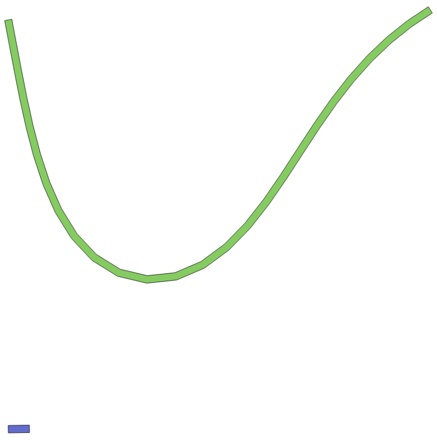
NCTC11474	*Escherichia coli*	73	1	0	3	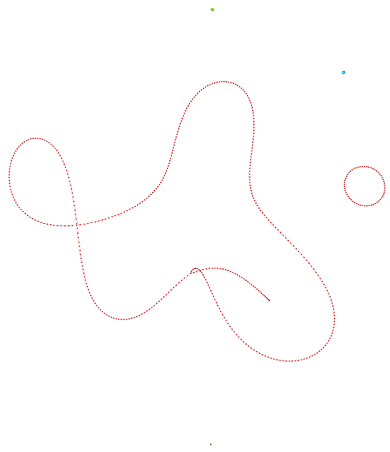	Mis-assembly/Fragmented	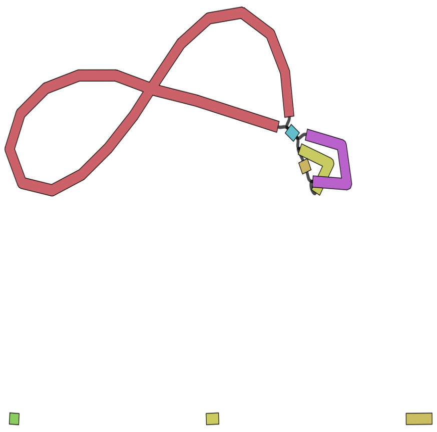
NCTC11475	*Escherichia coli*	62	1	0	3	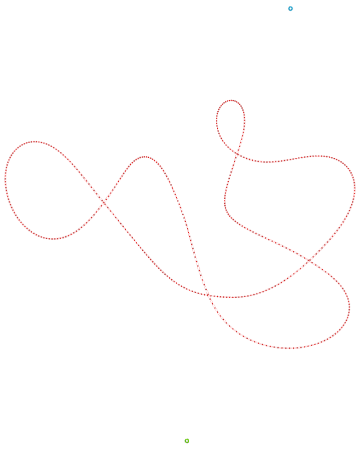	Finished circular assembly	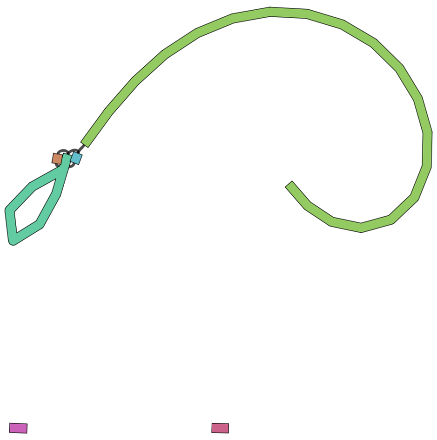
NCTC11476	*Escherichia coli*	82	1	0	0	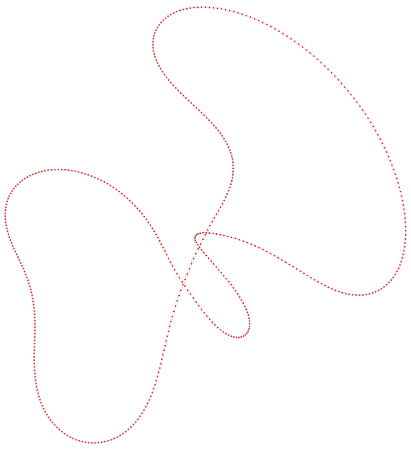	Finished circular assembly	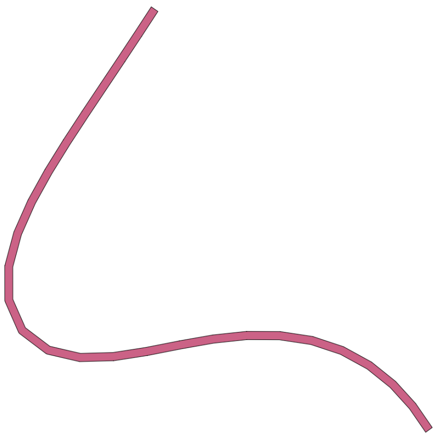
NCTC11477	*Legionella longbeachae*	96	Pending	Pending	Pending		Finished circular assembly	

						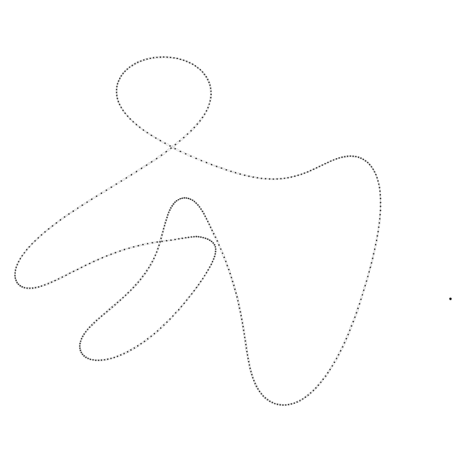		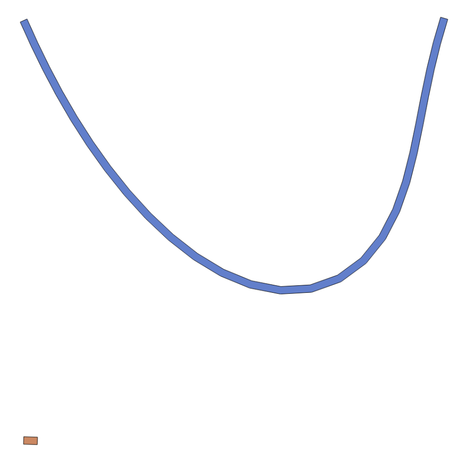
NCTC11531	*Legionella oakridgensis*	75	Pending	Pending	Pending	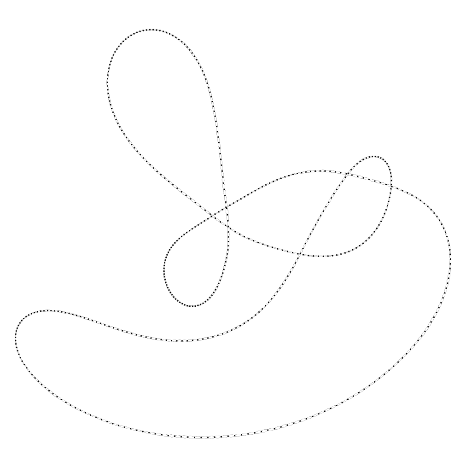	Finished circular assembly	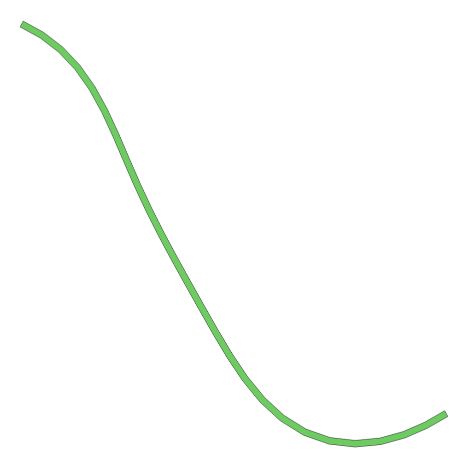
NCTC11532	*Legionella wadsworthii*	64	Pending	Pending	Pending	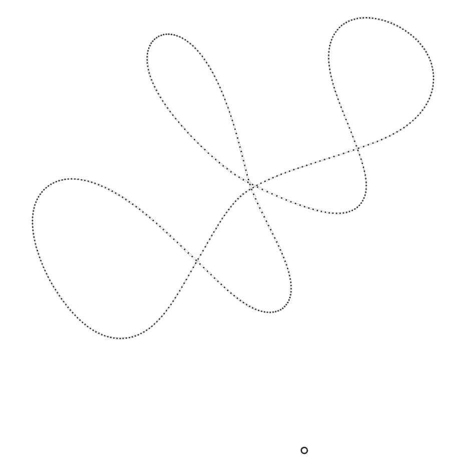	Finished circular assembly	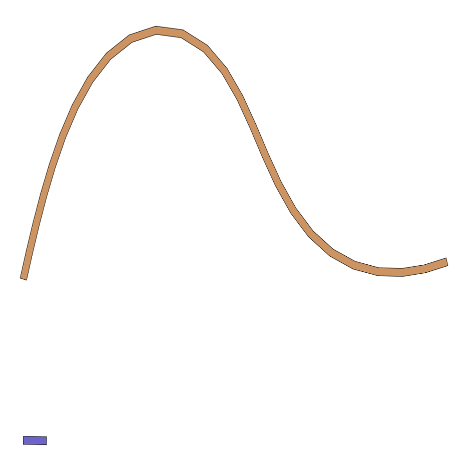
NCTC11543	*Serratia grimesii*	63	1	0	0	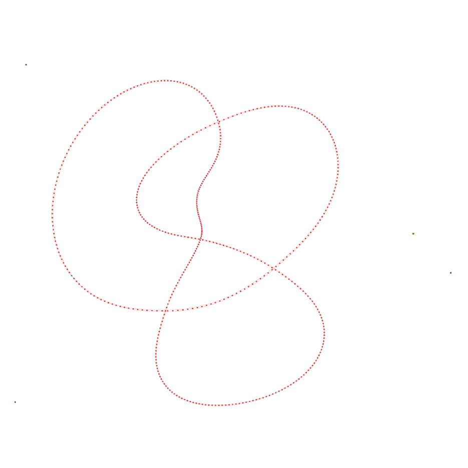	Finished circular assembly	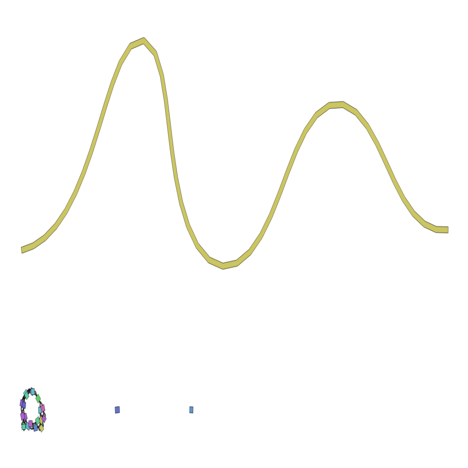
NCTC11544	*Serratia quinivorans*	38	0	0	3	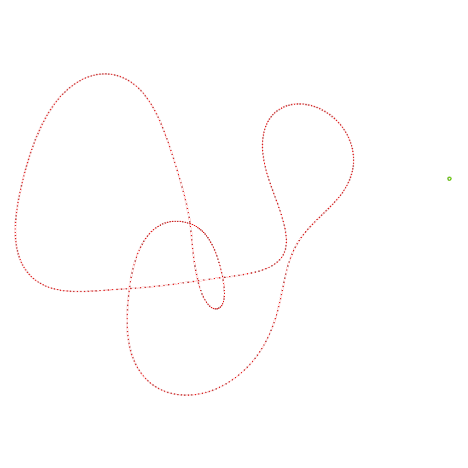	Finished circular assembly	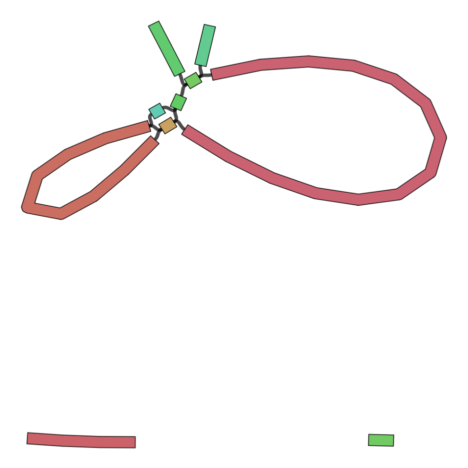
NCTC11561	*Staphylococcus aureus*	57	1	1	1	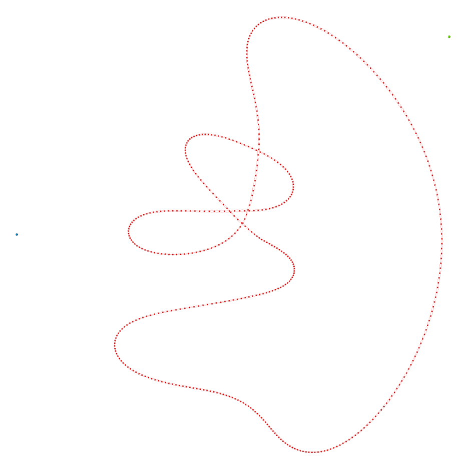	Finished circular assembly	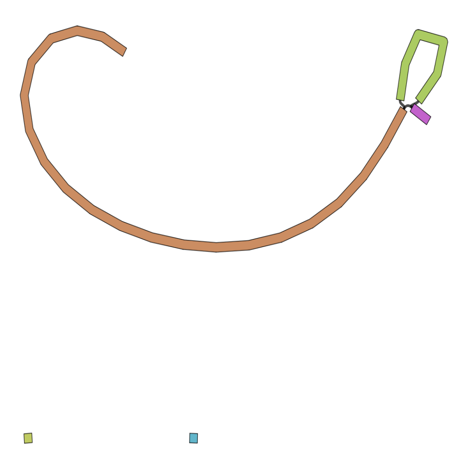
NCTC11567	*Streptococcus sp.*	217	Pending	Pending	Pending		Finished circular assembly	

						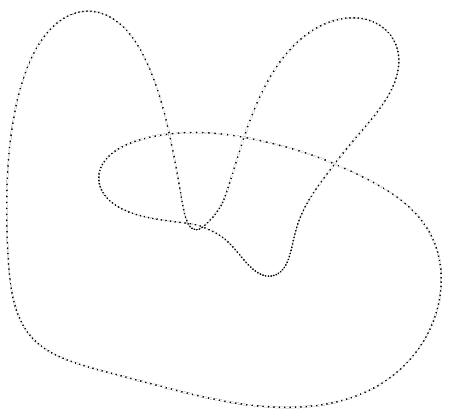		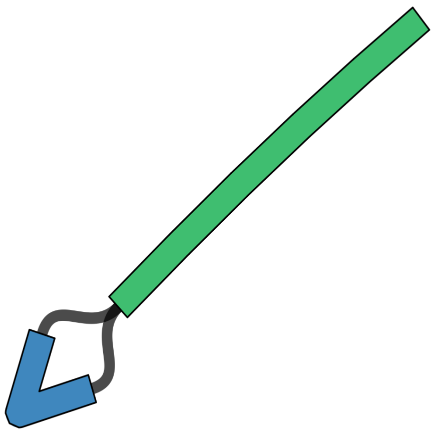
NCTC11571	*Enterobacter cloacae*	77	1	0	0	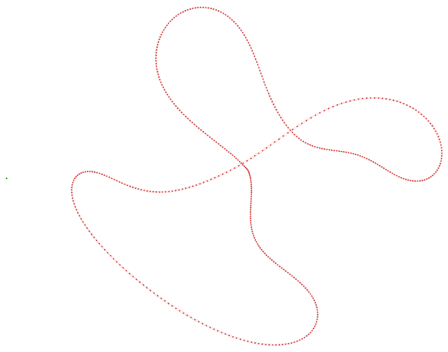	Finished circular assembly	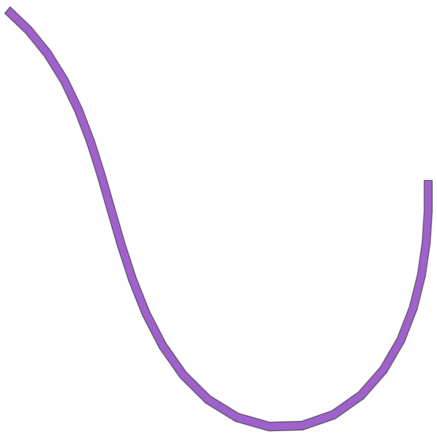
NCTC11579	*Enterobacter cloacae*	76	1	0	1	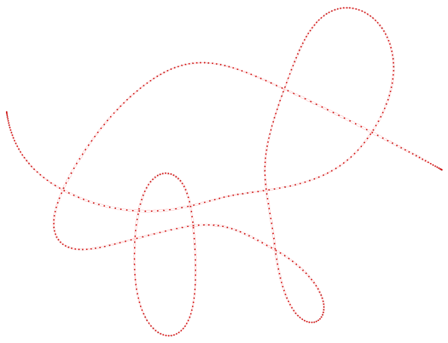	Mis-assembly	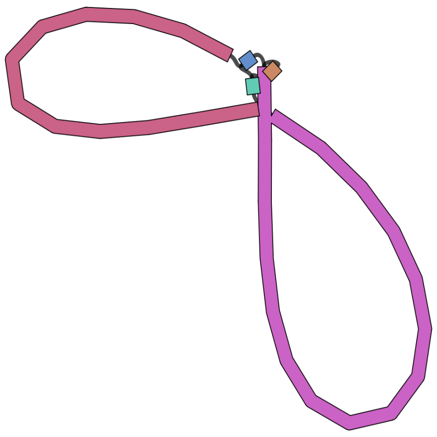
NCTC11588	*Enterobacter cloacae*	72	1	0	1	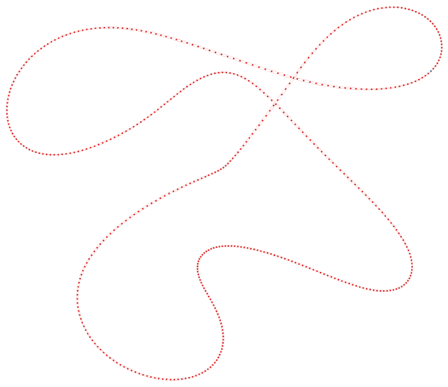	Finished circular assembly	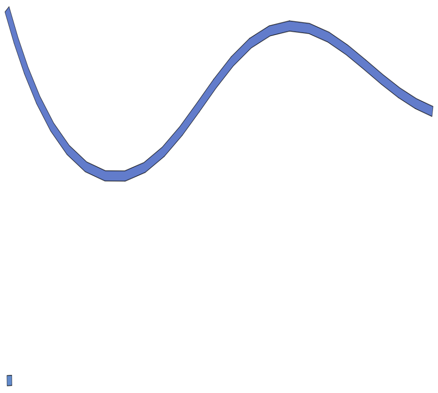
NCTC11606	*Streptococcus equi*	68	Pending	Pending	Pending	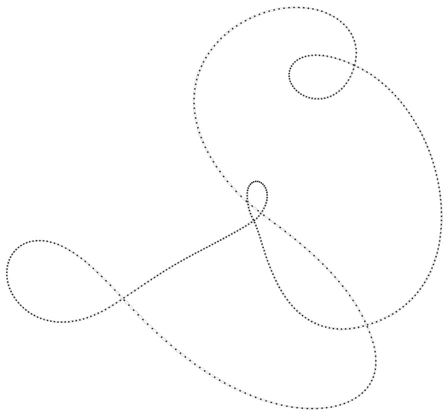	Finished circular assembly	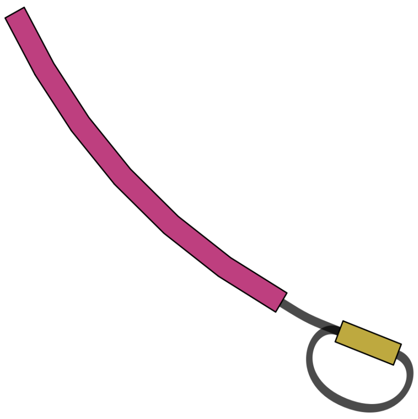
NCTC11607	*Aggregatibacter sp.*	102	1	0	1	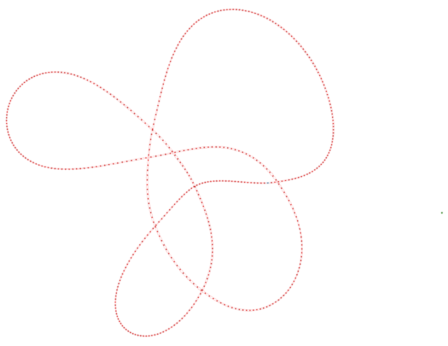	Finished circular assembly	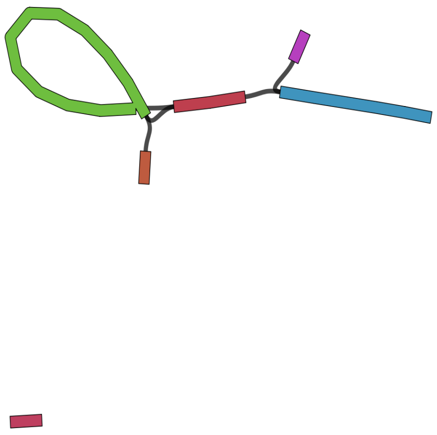
NCTC11637	*Helicobacter pylori*	126	1	0	0		Finished circular assembly	

						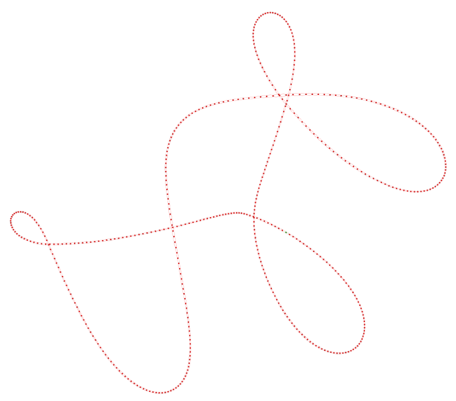		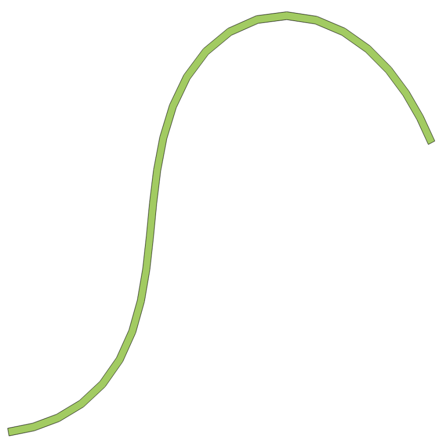
NCTC11667	*Providencia rustigianii*	58	Pending	Pending	Pending	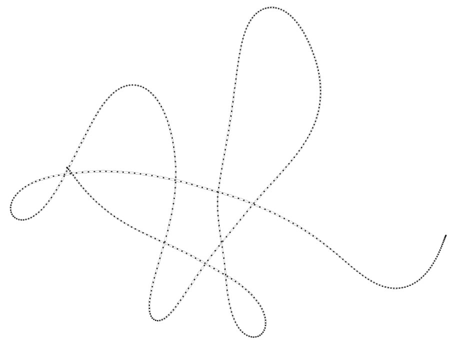	Finished assembly (lacking circularisation)	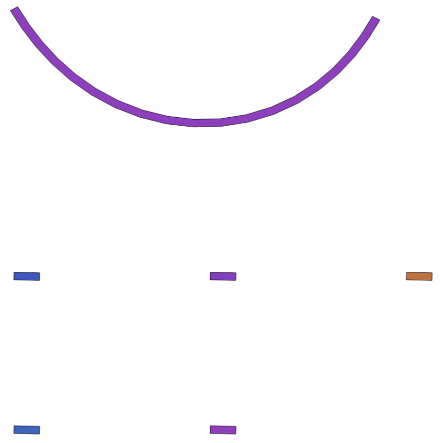
NCTC11674	*Klebsiella sp*	54	1	1	1	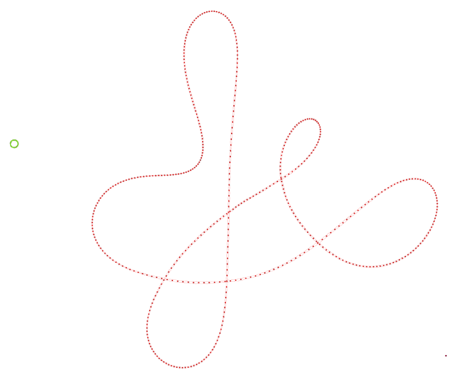	Finished circular assembly	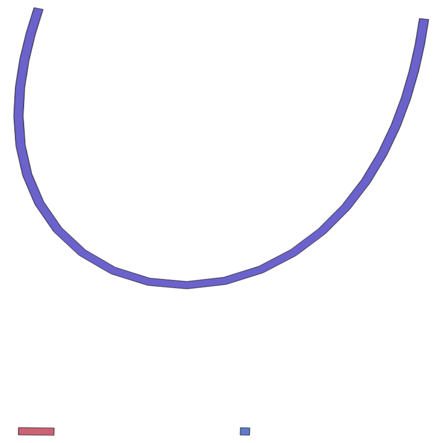
NCTC11675	*Klebsiella sp*.	40	1	1	1	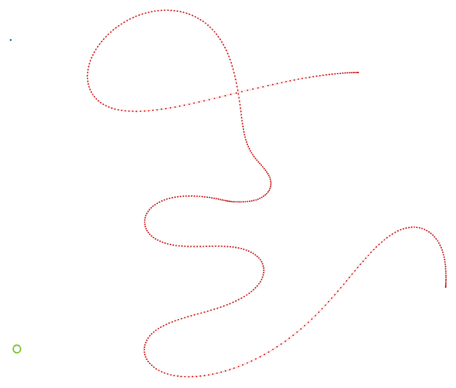	Finished assembly (lacking circularisation)	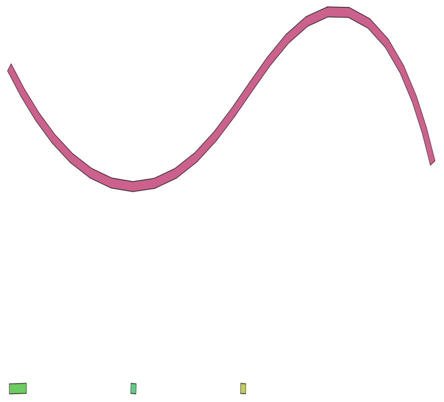
NCTC11676	*Klebsiella sp*	56	0	0	2	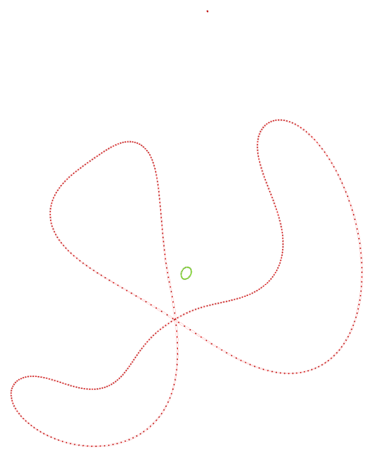	Finished circular assembly	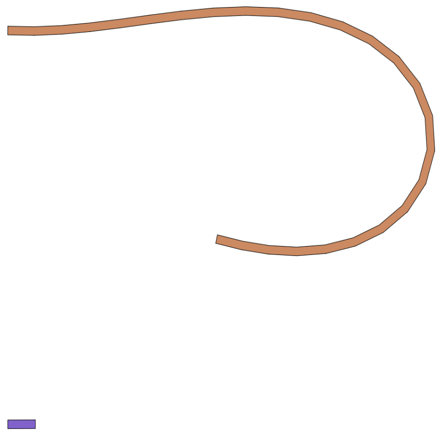
NCTC11678	*Klebsiella sp*	57	1	1	2	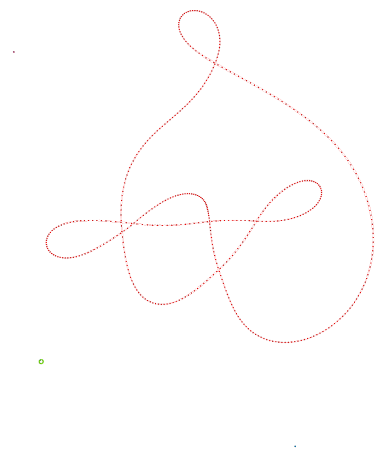	Finished circular assembly	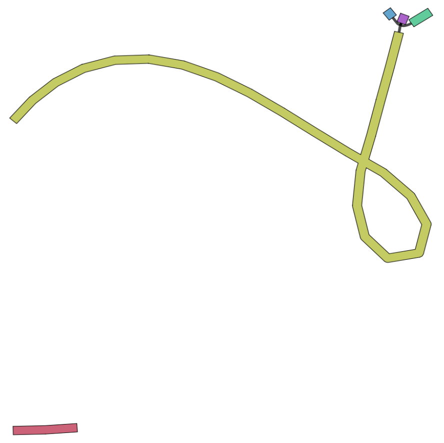
NCTC11679	*Klebsiella sp*.	46	1	0	6		Finished circular assembly	

						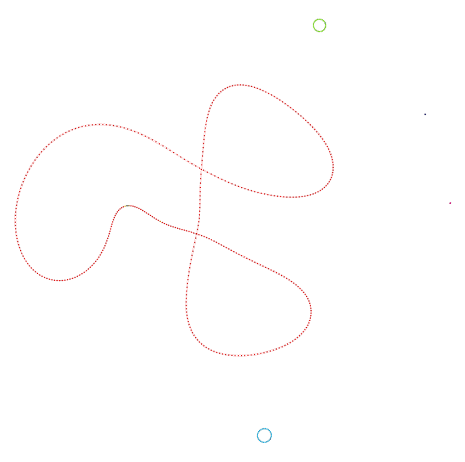		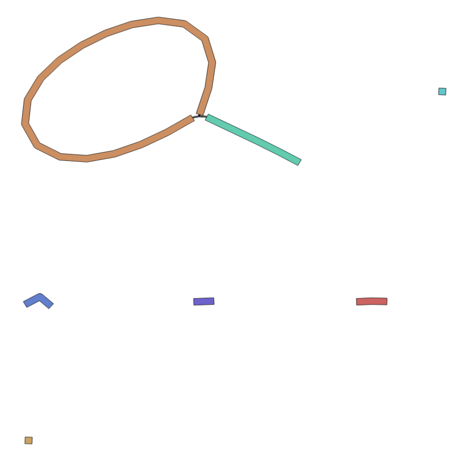
NCTC11680	*Klebsiella sp*	50	0	0	3	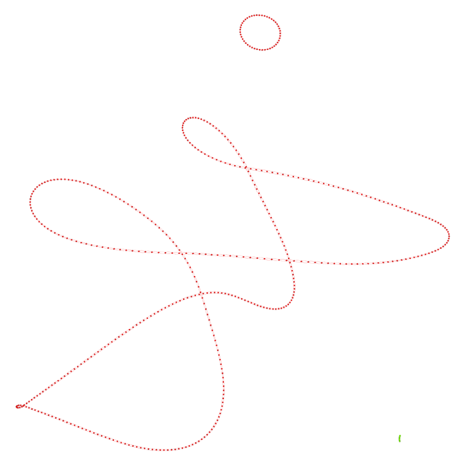	Finished circular assembly	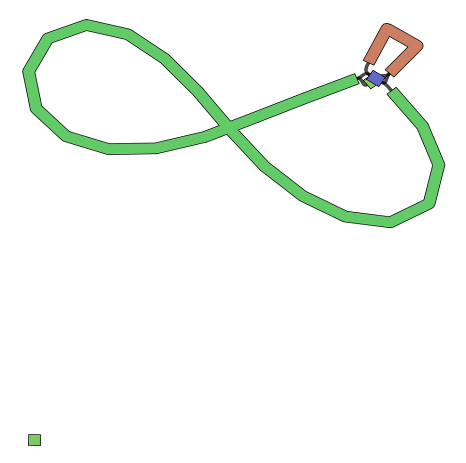
NCTC11681	*Klebsiella sp*.	41	Pending	Pending	Pending	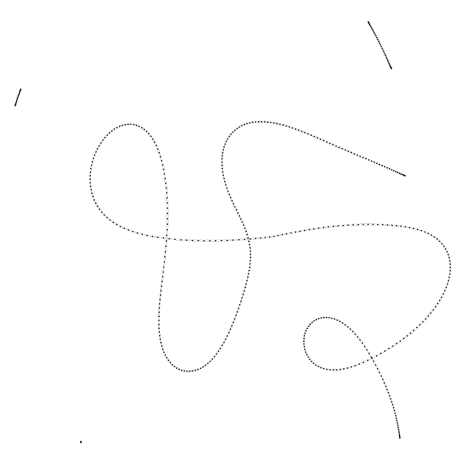	Finished assembly (lacking circularisation)	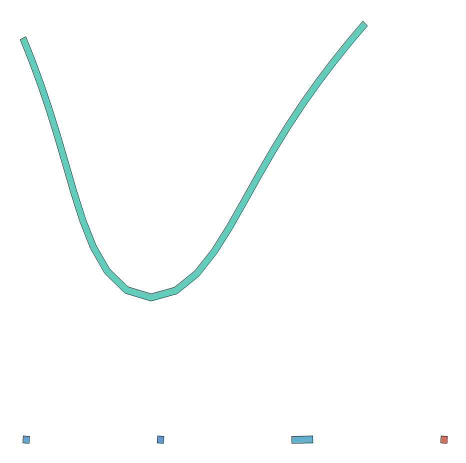
NCTC11682	*Klebsiella sp*.	39	1	1	3	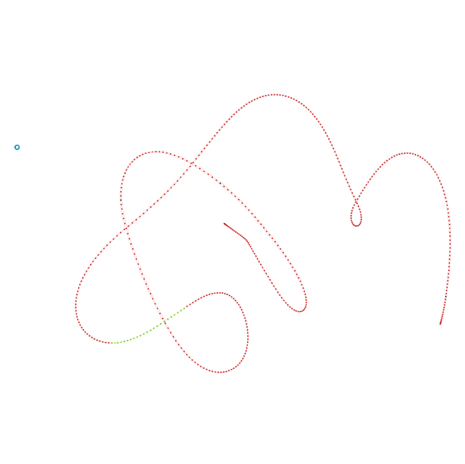	Finished assembly (lacking circularisation)	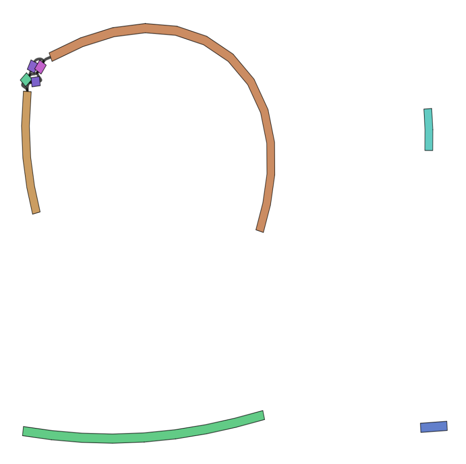
NCTC11683	*Klebsiella oxytoca*	54	1	0	5	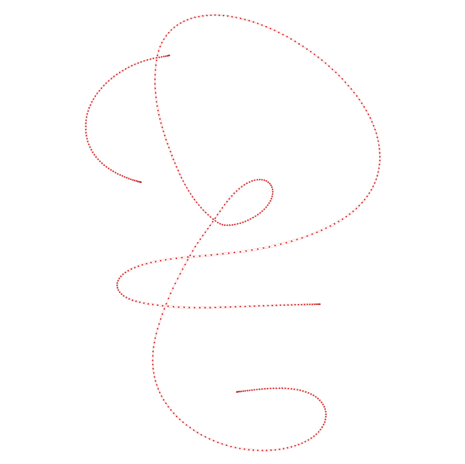	Mis-assembly/Fragmented	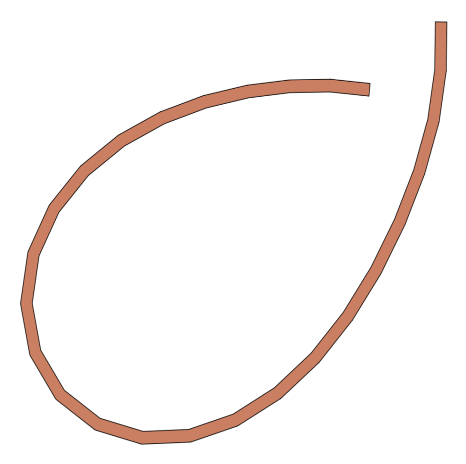
NCTC11684	*Klebsiella sp*.	35	0	0	5	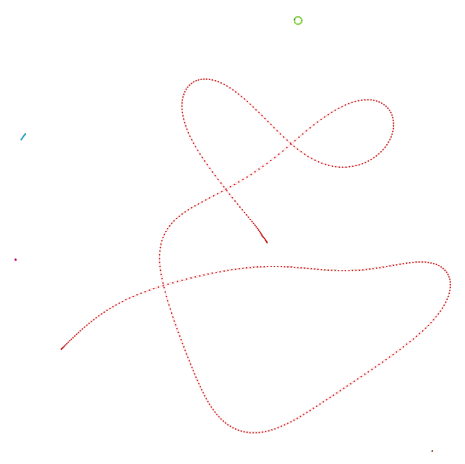	Finished assembly (lacking circularisation)	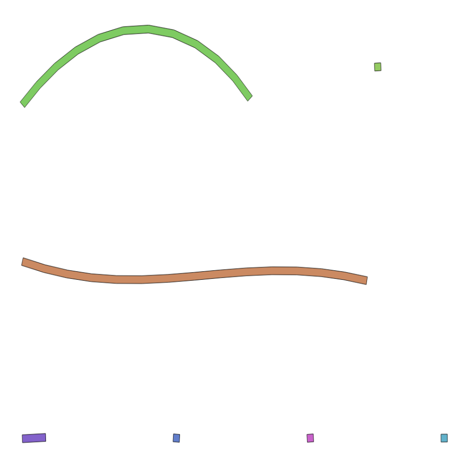
NCTC11685	*Klebsiella sp*.	28	0	0	8		Mis-assembly/Fragmented	

						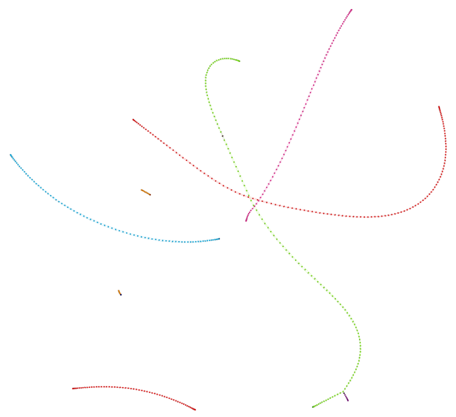		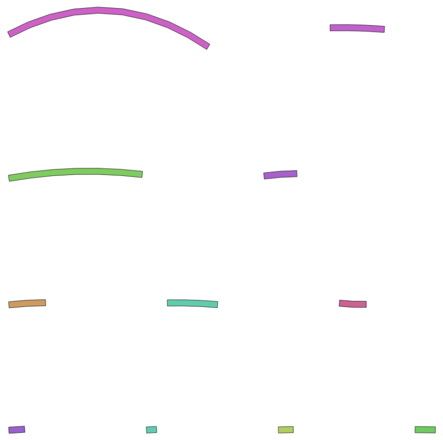
NCTC11686	*Klebsiella oxytoca*	52	0	0	4	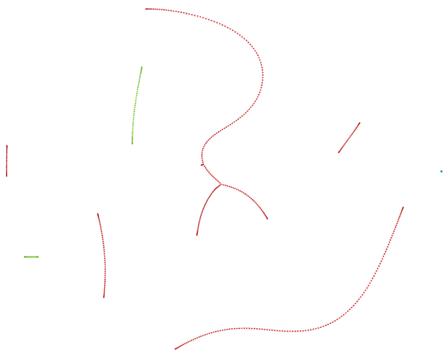	Mis-assembly/Fragmented	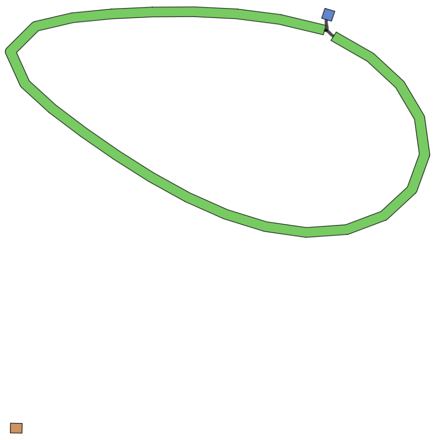
NCTC11687	*Klebsiella sp*	41	0	0	4	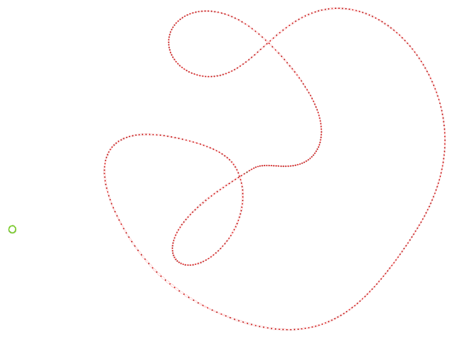	Finished circular assembly	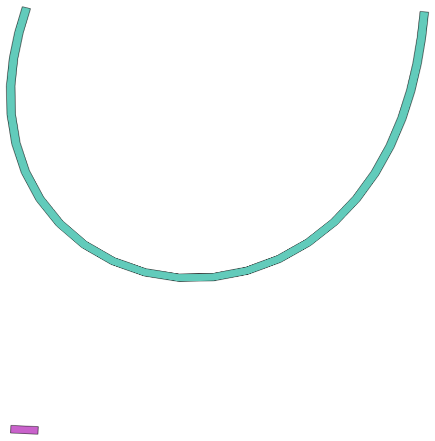
NCTC11688	*Klebsiella sp*	49	0	0	9	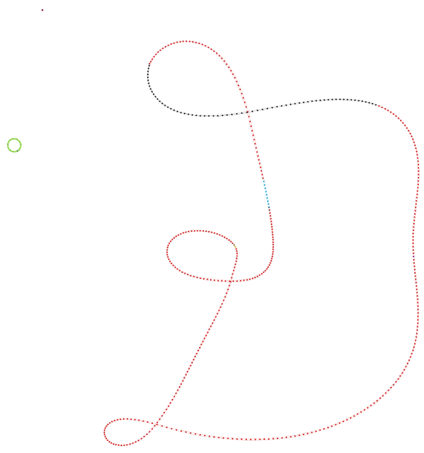	Finished circular assembly	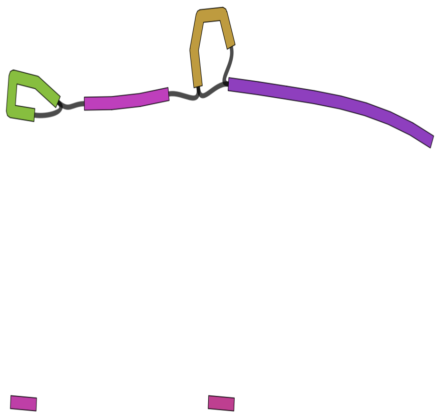
NCTC11689	*Klebsiella sp.*	54	0	0	13	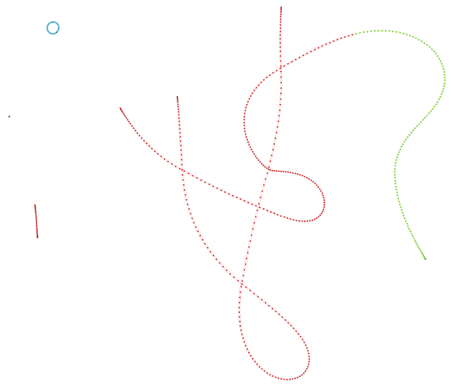	Mis-assembly/Fragmented	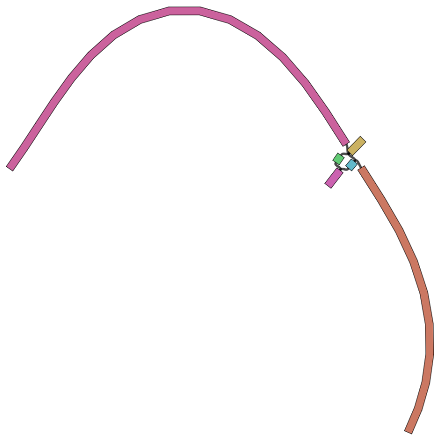
NCTC11691	*Klebsiella oxytoca*	50	Pending	Pending	Pending	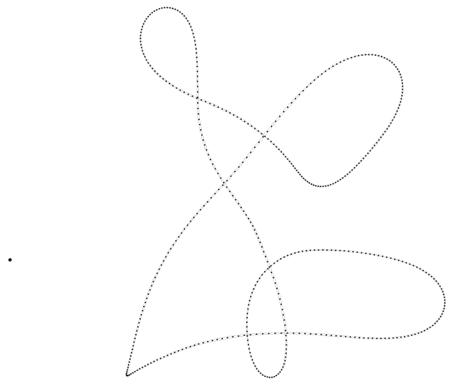	Finished circular assembly	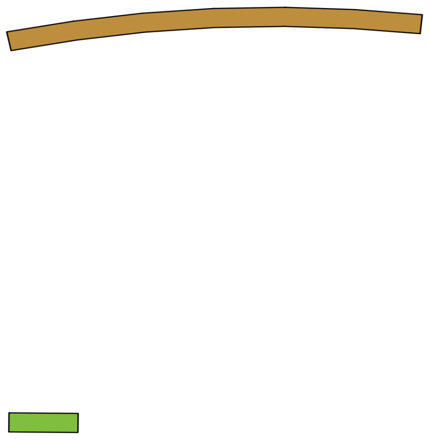
NCTC11692	*Klebsiella sp*	34	0	0	12		Finished circular assembly	

						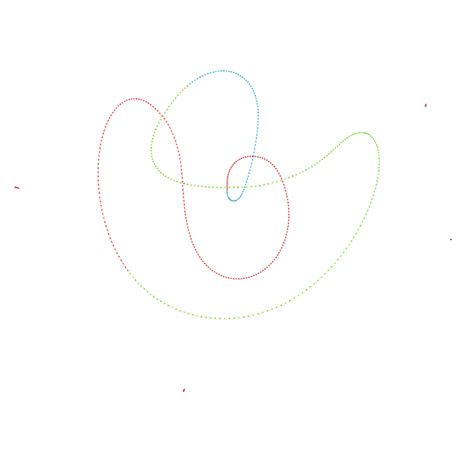		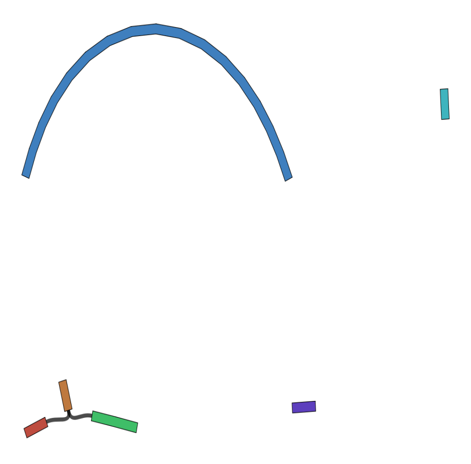
NCTC11693	*Klebsiella sp*	31	0	0	9	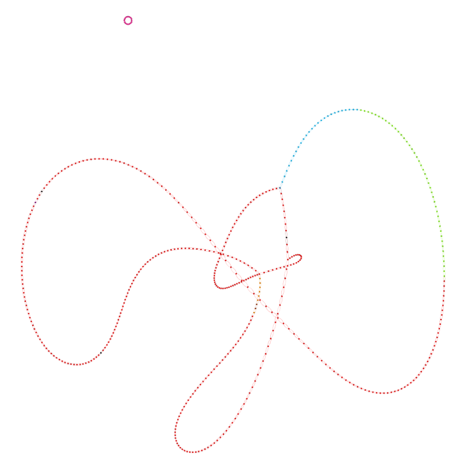	Mis-assembly/Fragmented	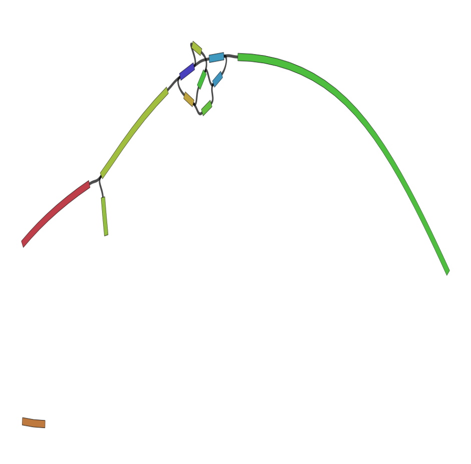
NCTC11694	*Klebsiella oxytoca*	34	0	0	7	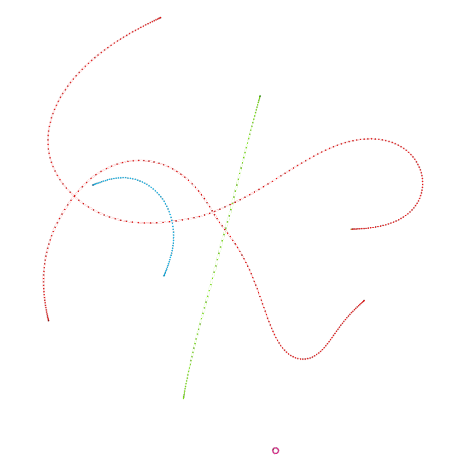	Mis-assembly/Fragmented	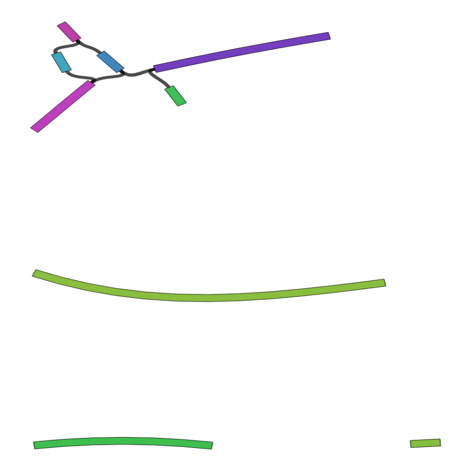
NCTC11695	*Klebsiella pneumoniae subsp. pneumoniae*	49	1	1	0	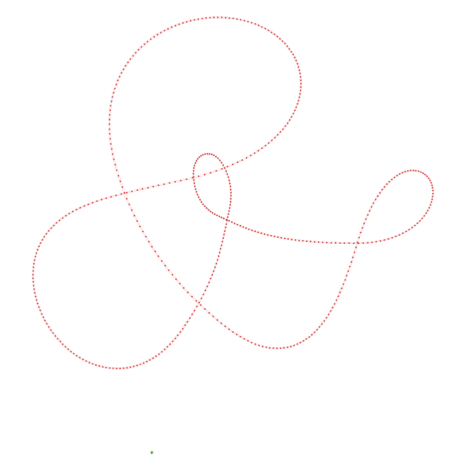	Finished circular assembly	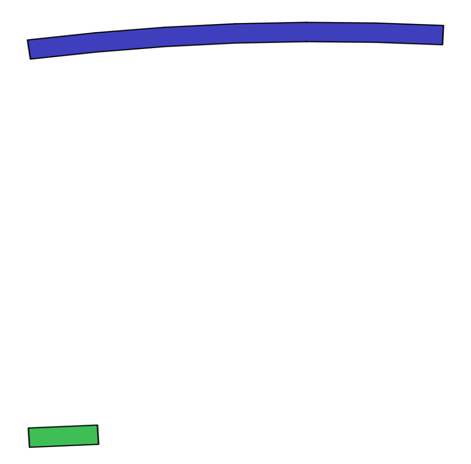
NCTC11696	*Klebsiella oxytoca*	73	1	2	1	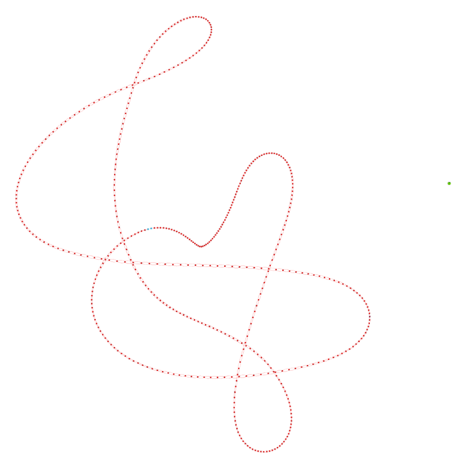	Finished circular assembly	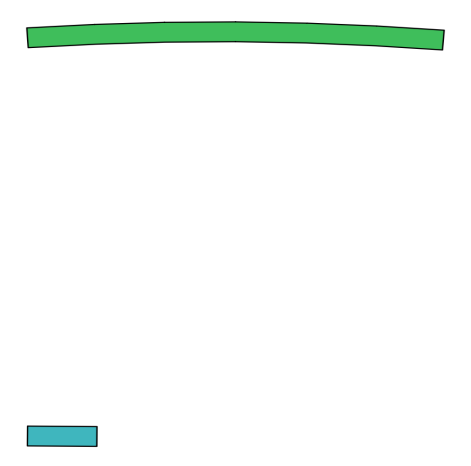
NCTC11697	*Klebsiella sp*	30	0	0	4	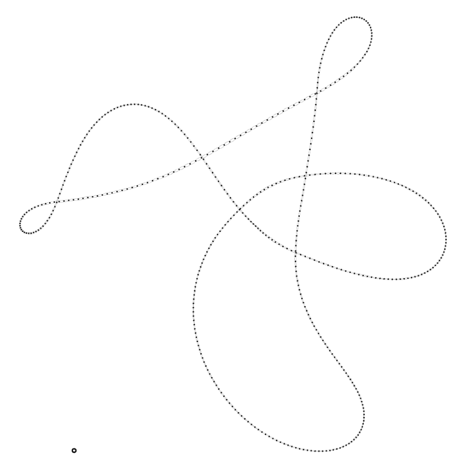	Finished circular assembly	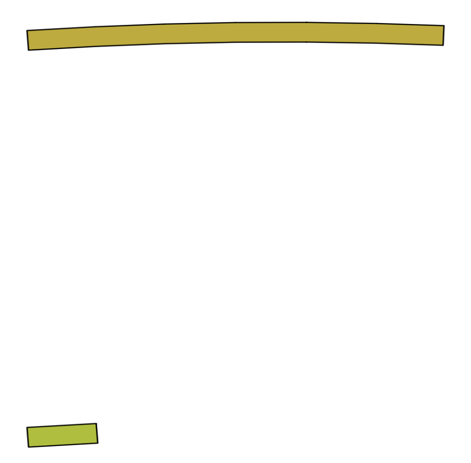
NCTC11698	*Klebsiella sp.*	24	1	0	0		Mis-assembly/Fragmented	

						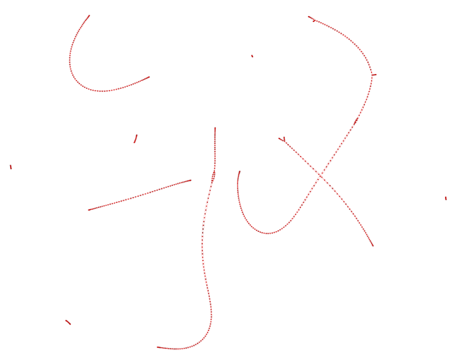		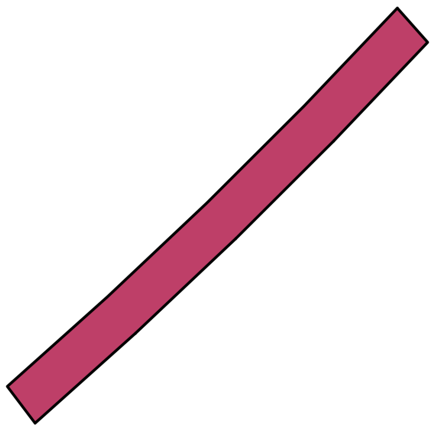
NCTC11800	*Providencia stuartii*	32	0	0	4	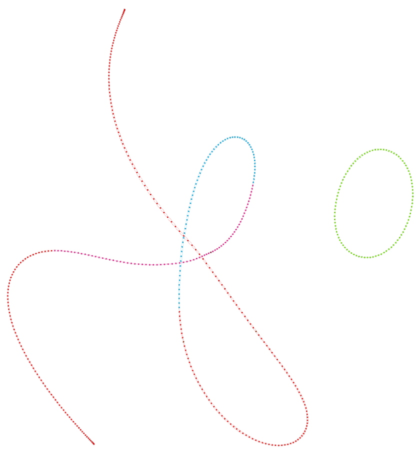	Mis-assembly	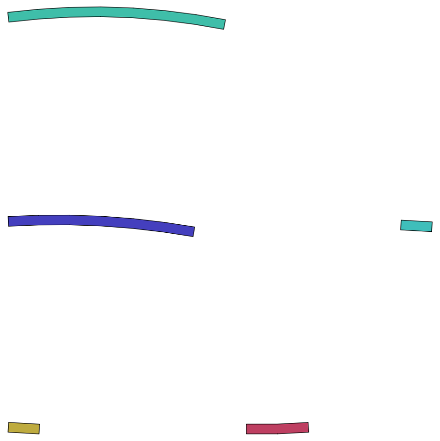
NCTC11801	*Providencia rettgeri*	57	0	0	5	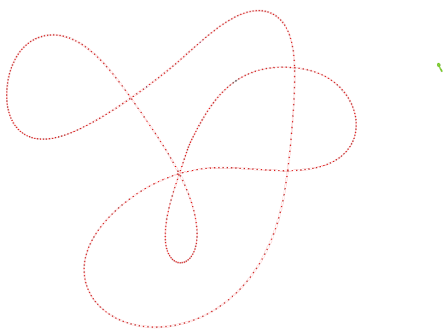	Finished circular assembly	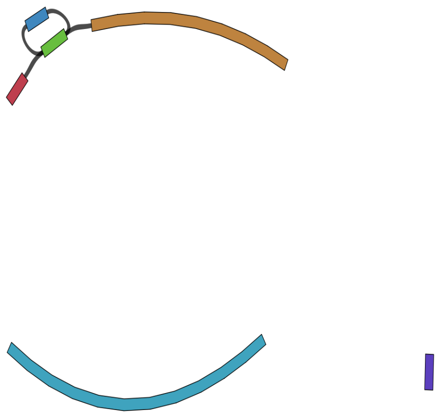
NCTC11802	*Providencia rustigianii*	39	1	2	0	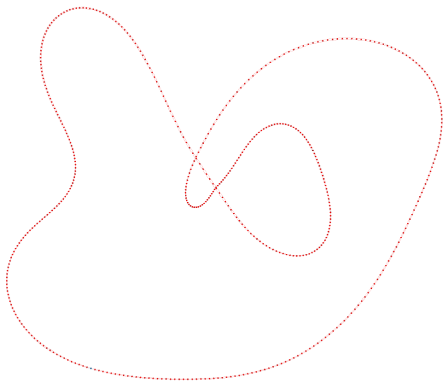	Finished circular assembly	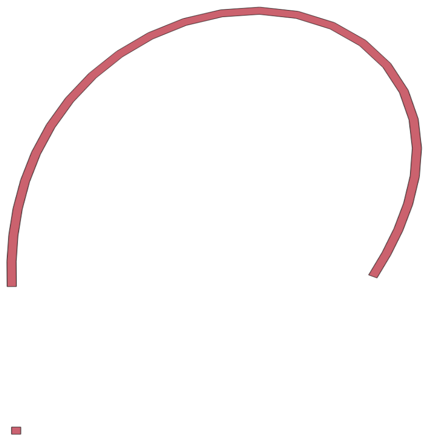
NCTC11854	*Streptococcus equi subsp. zooepidemicus*	122	0	0	1	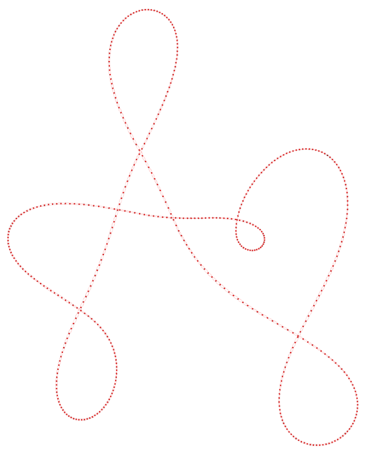	Finished circular assembly	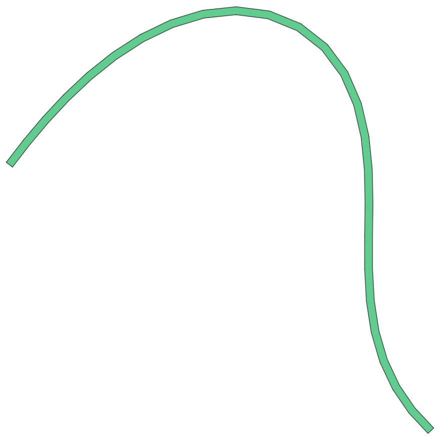
NCTC11872	*Haemophilus influenzae*	27	0	0	11	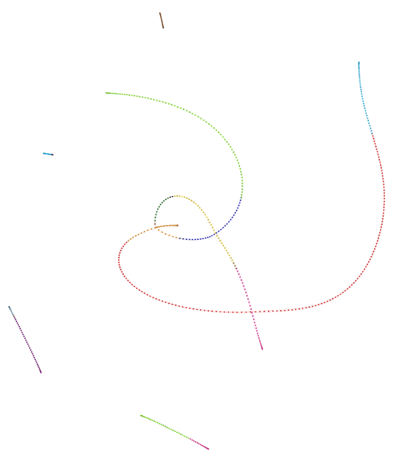	Mis-assembly/Fragmented	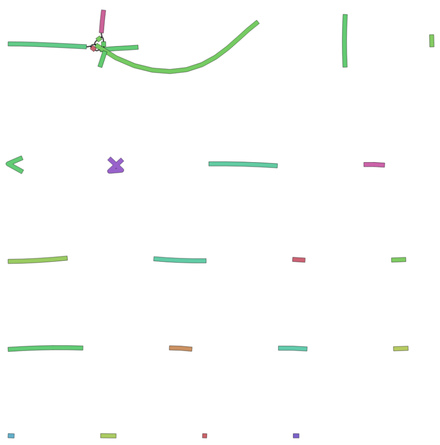
NCTC11881	*Salmonella sp*	40	1	1	1		Mis-assembly/Fragmented	

						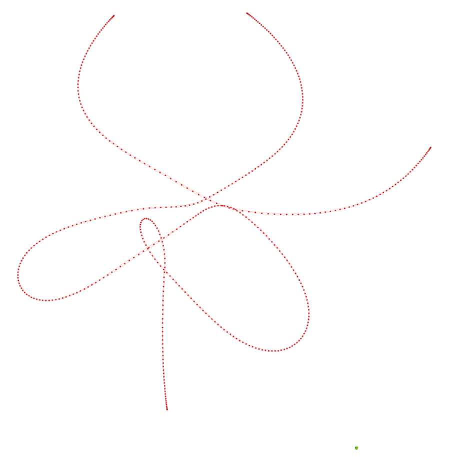		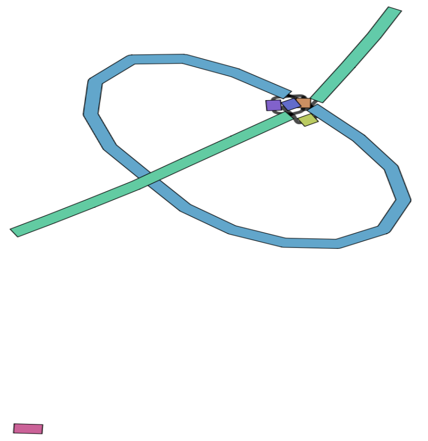
NCTC11925	*Campylobacter jejuni*	167	0	0	1	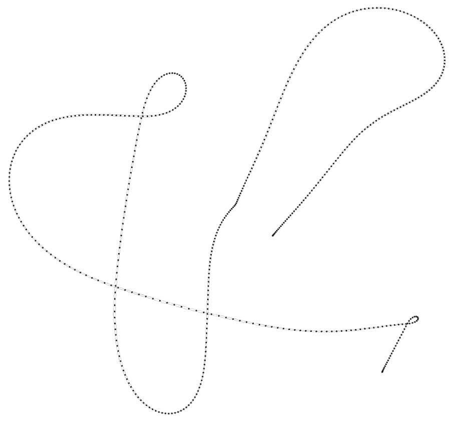	Finished circular assembly	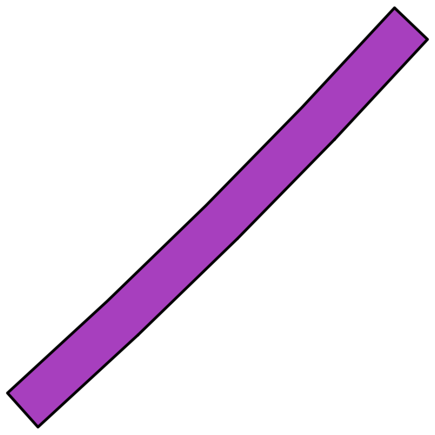
NCTC11930	*Streptococcus agalactiae*	179	Pending	Pending	Pending	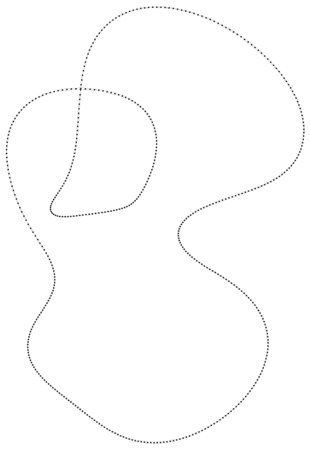	Finished circular assembly	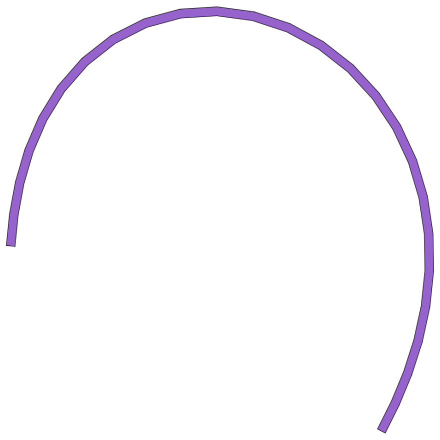
NCTC11938	*Proteus mirabilis*	46	0	0	1	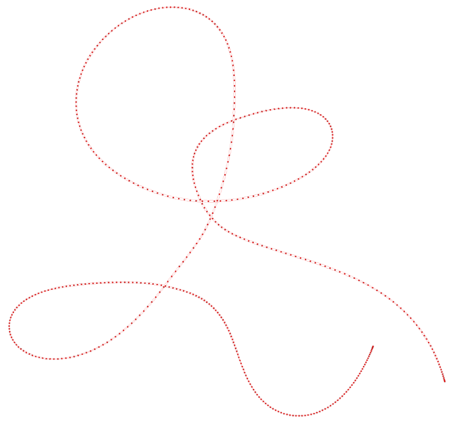	Finished assembly (lacking circularisation)	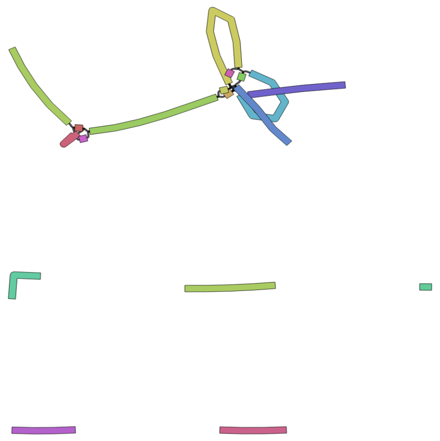
NCTC11939	*Staphylococcus aureus*	73	0	0	7	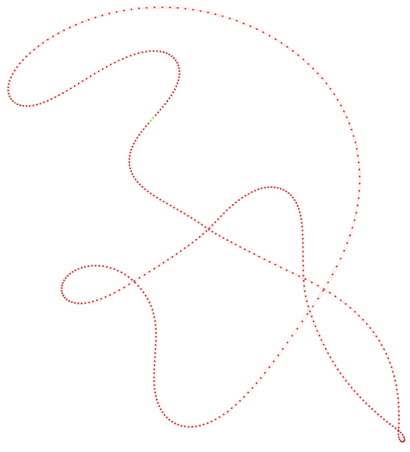	Finished circular assembly	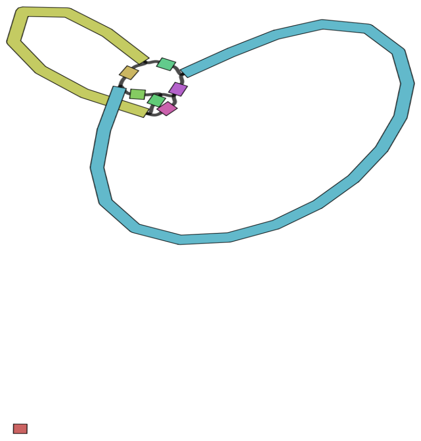
NCTC11940	*Staphylococcus aureus*	79	Pending	Pending	Pending	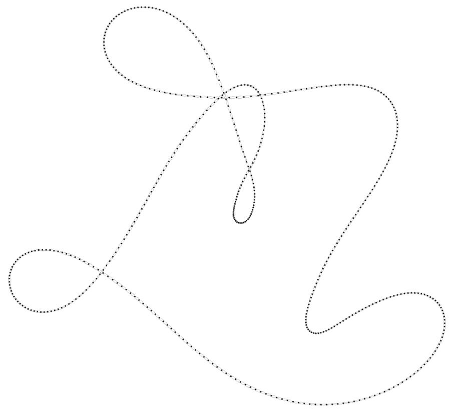	Finished circular assembly	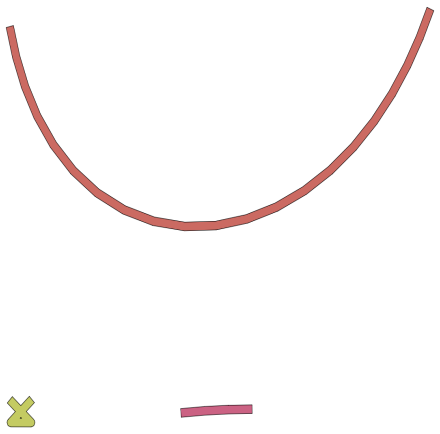
NCTC11962	*Staphylococcus aureus*	71	0	0	4		Finished assembly with multiple traversals	

						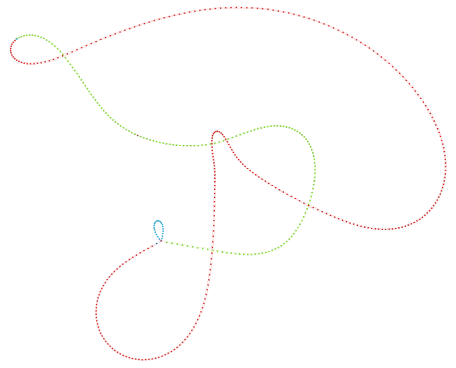		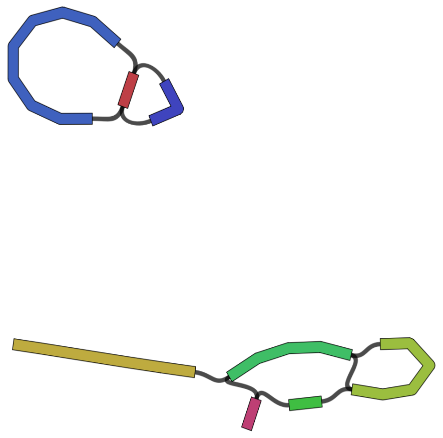
NCTC11963	*Staphylococcus aureus*	108	1	0	8	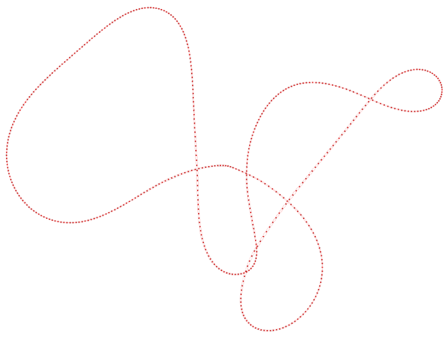	Finished circular assembly	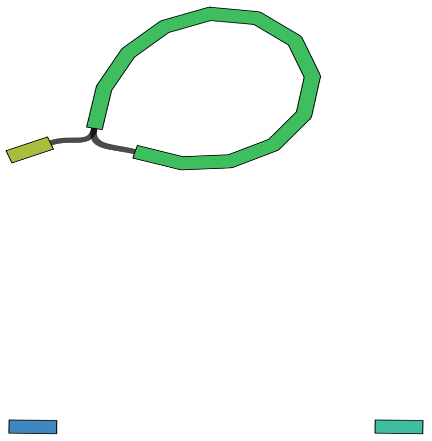
NCTC11964	*Staphylococcus epidermidis*	158	1	1	3	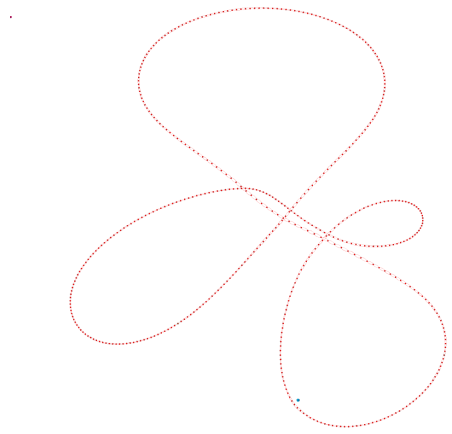	Finished circular assembly	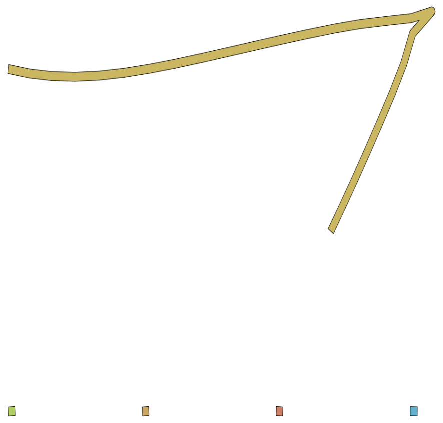
NCTC11965	*Staphylococcus aureus*	96	1	0	0	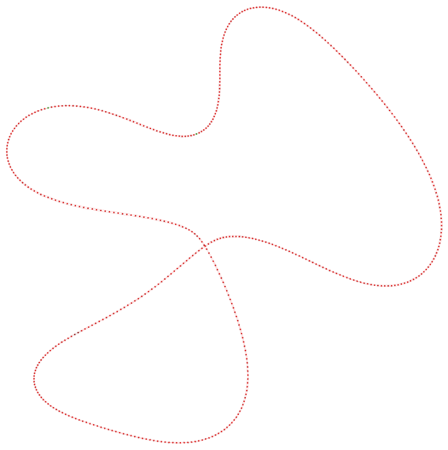	Finished circular assembly	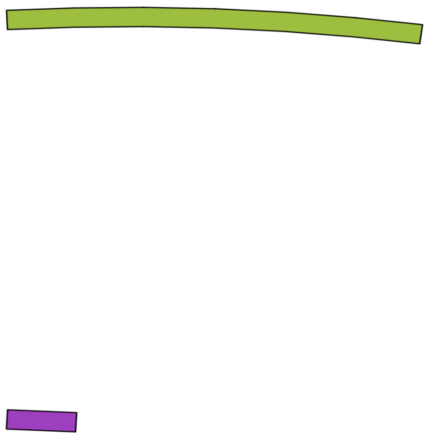
NCTC11976	*Legionella cherrii*	37	0	0	1	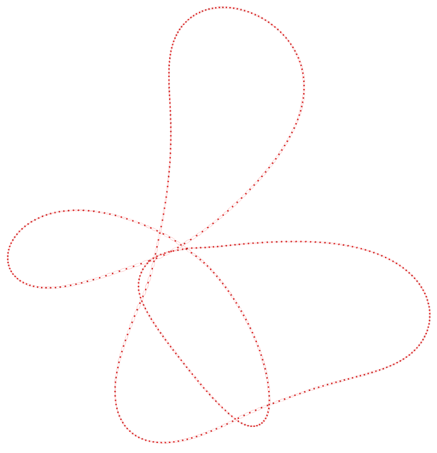	Finished circular assembly	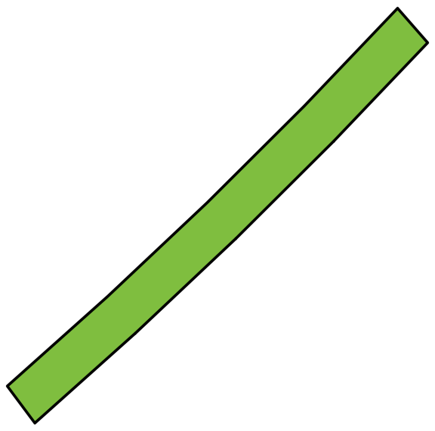
NCTC11978	*Legionella feeleii*	58	1	1	0	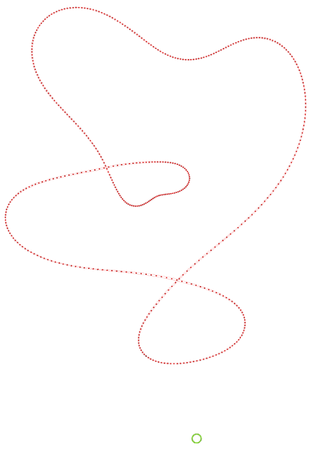	Finished circular assembly	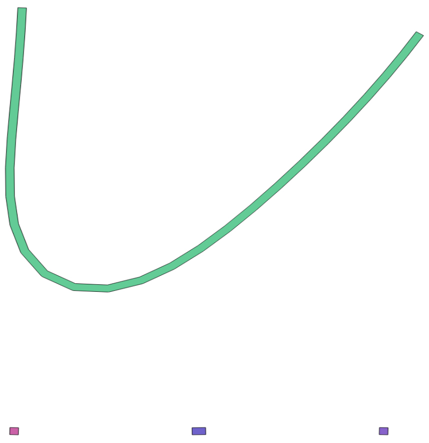
NCTC11979	*Legionella hackeliae*	44	1	1	0		Finished circular assembly	

						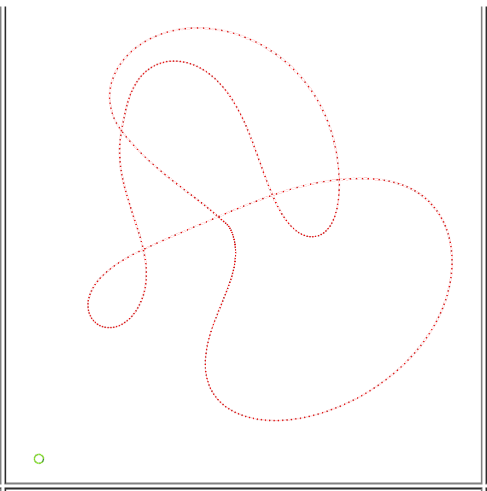		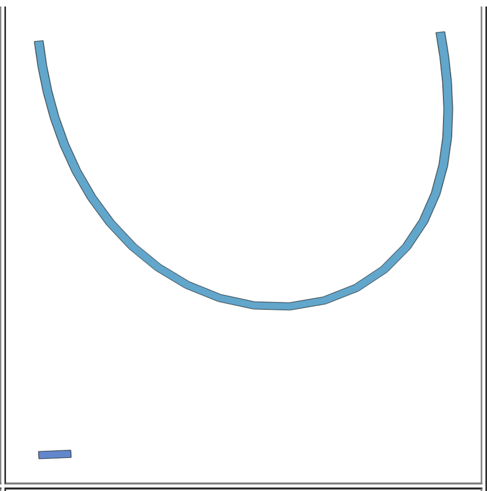
NCTC12007	*Legionella pneumophila*	52	1	0	1	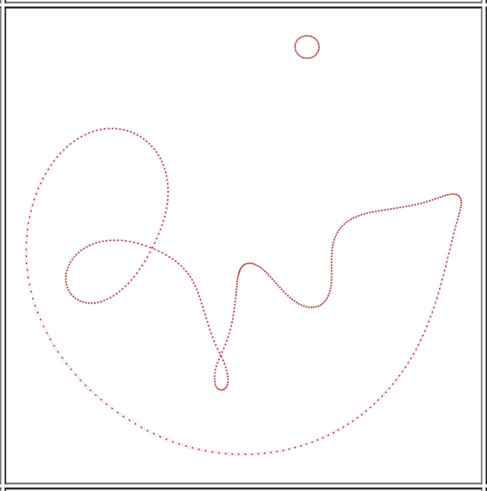	Finished circular assembly	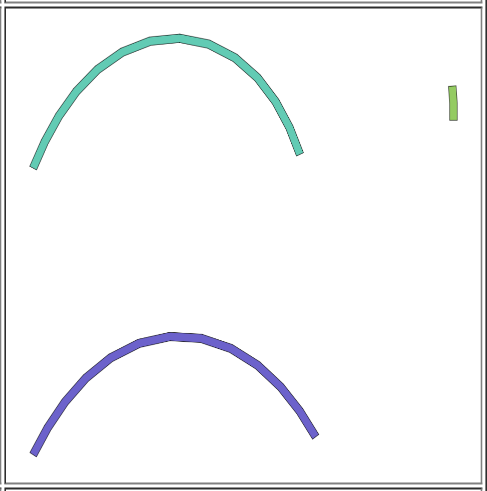
NCTC12012	*Vibrio cincinnatiensis*	48	2	0	1	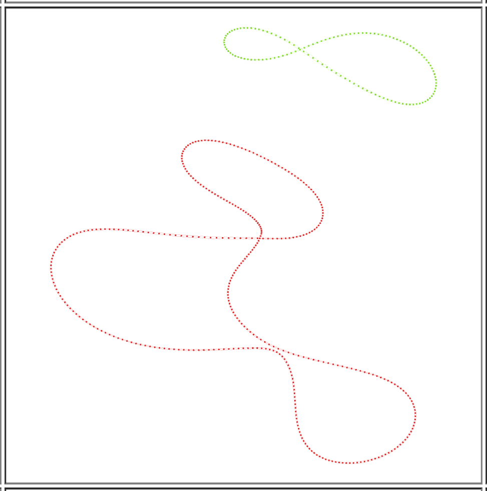	Finished circular assembly	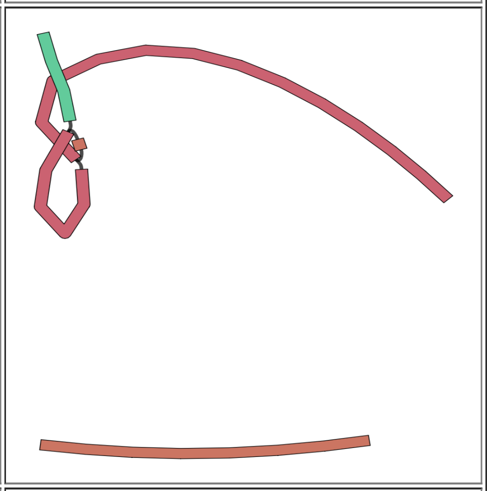
NCTC12023	*Salmonella enterica subsp. enterica serovar Typhimurium*	44	0	0	2	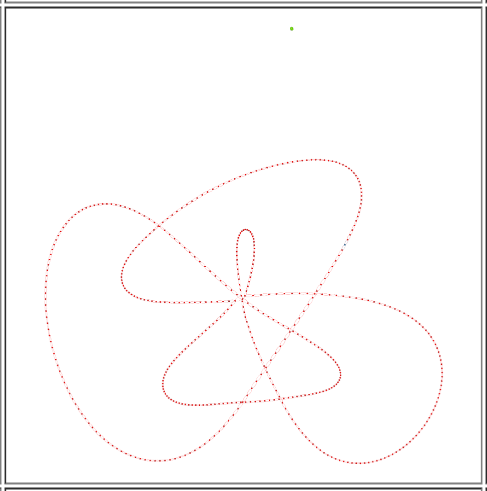	Finished assembly with multiple traversals	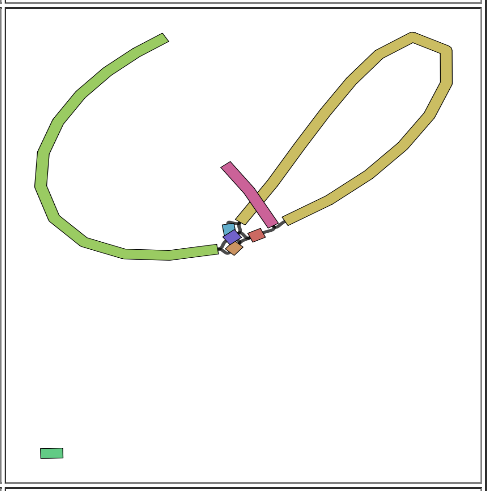
NCTC12026	*Providencia rustigianii*	133	1	1	2	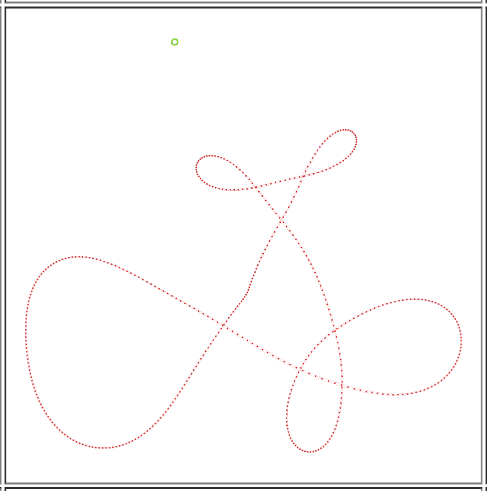	Finished circular assembly	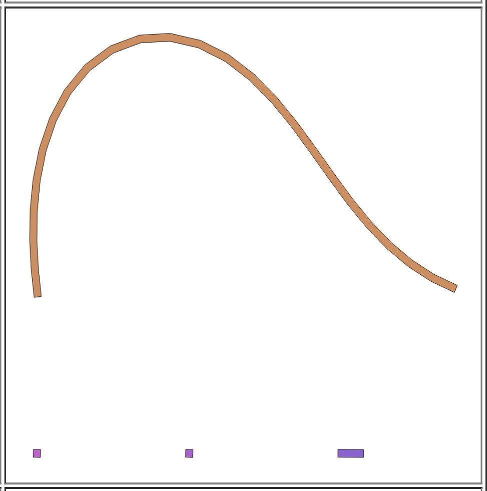
NCTC12028	*Morganella morganii*	80	1	0	0	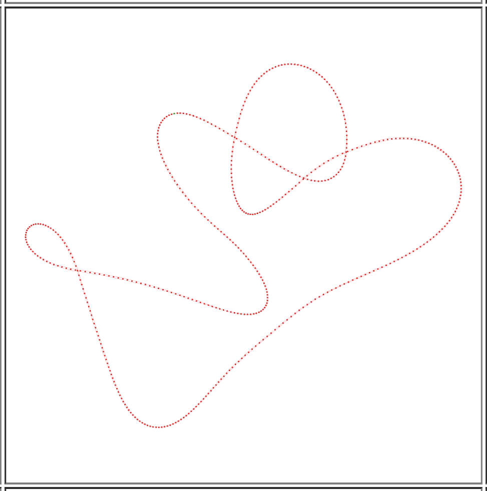	Finished circular assembly	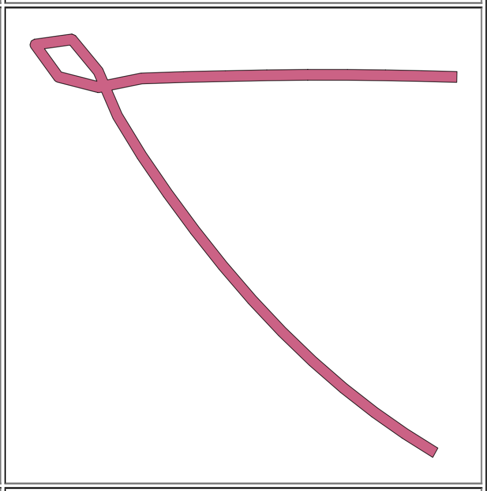
NCTC12035	*Staphylococcus aureus*	42	1	1	0		Finished circular assembly	

						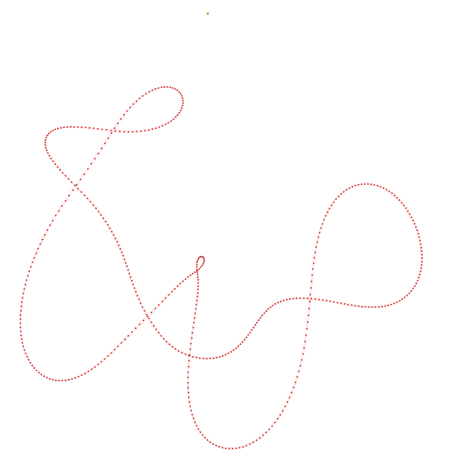		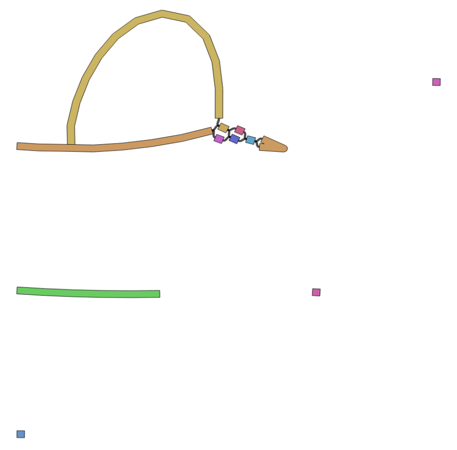
NCTC12045	*Streptococcus pyogenes*	118	1	0	0	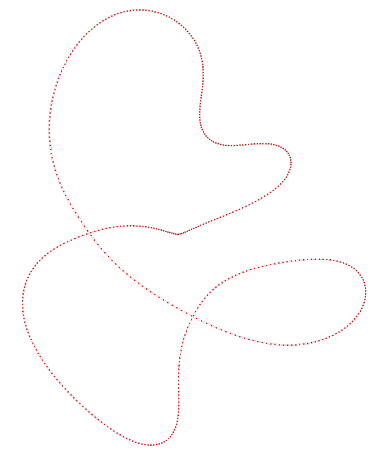	Finished circular assembly	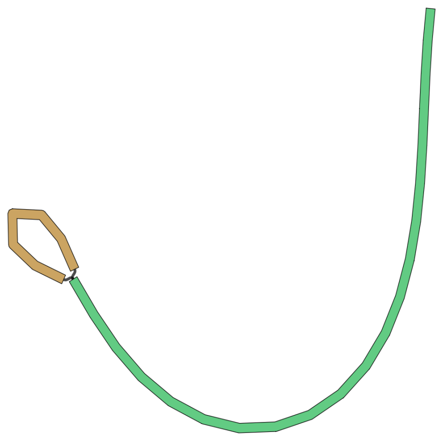
NCTC12046	*Streptococcus pyogenes*	167	1	0	0	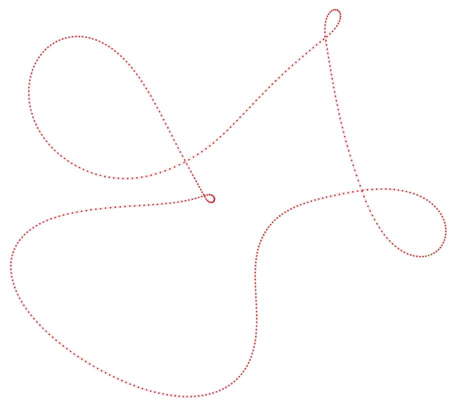	Finished circular assembly	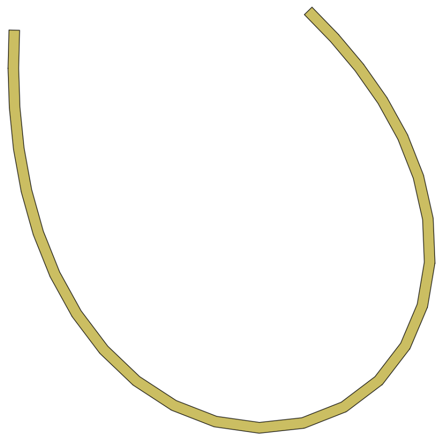
NCTC12047	*Streptococcus pyogenes*	187	1	0	0	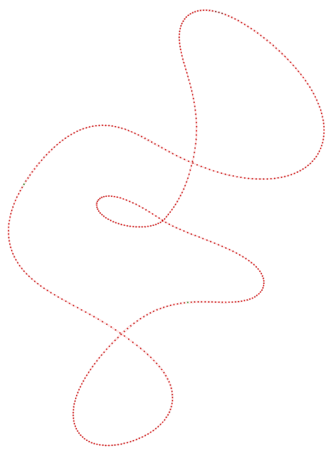	Finished circular assembly	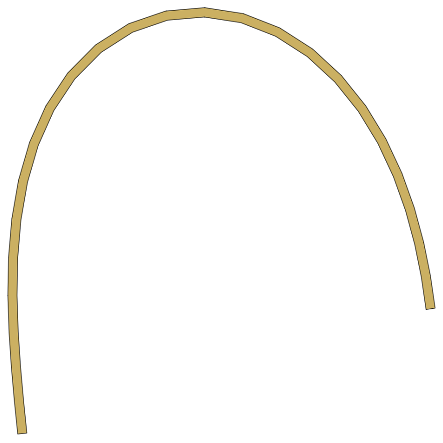
NCTC12048	*Streptococcus pyogenes*	144	1	0	0	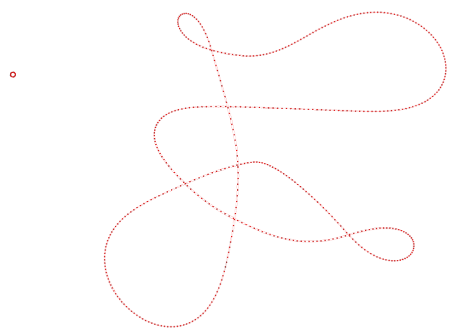	Finished circular assembly	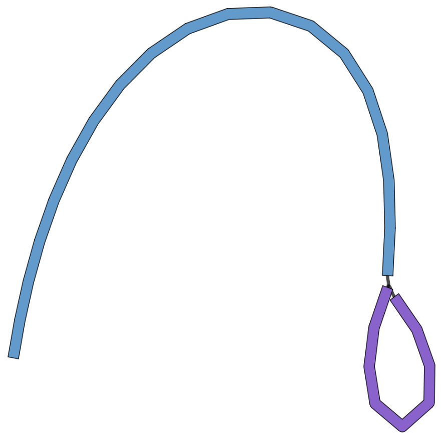
NCTC12049	*Streptococcus pyogenes*	183	1	0	1	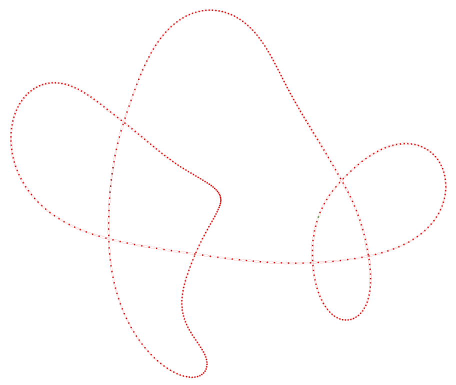	Finished circular assembly	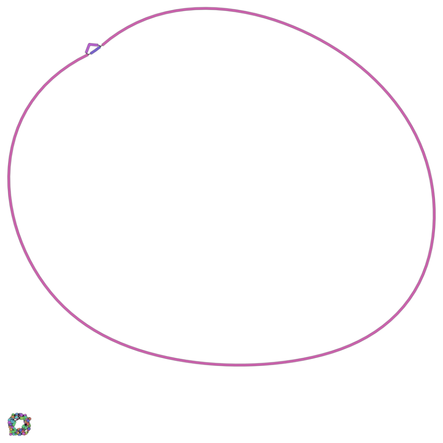
NCTC12050	*Streptococcus pyogenes*	200	1	0	0		Finished circular assembly	

						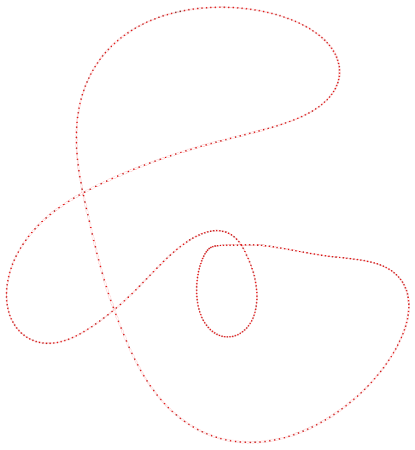		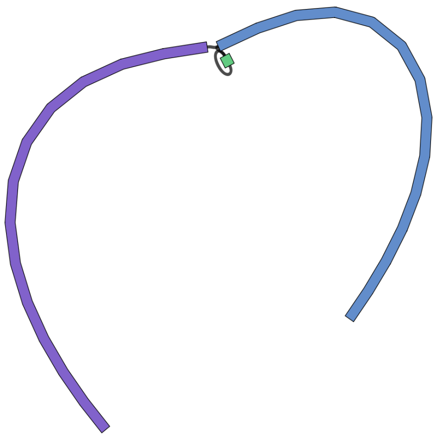
NCTC12051	*Streptococcus pyogenes*	76	1	0	1	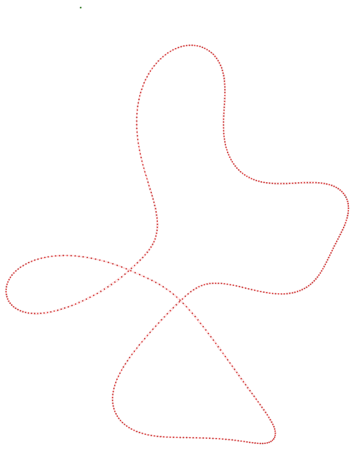	Finished circular assembly	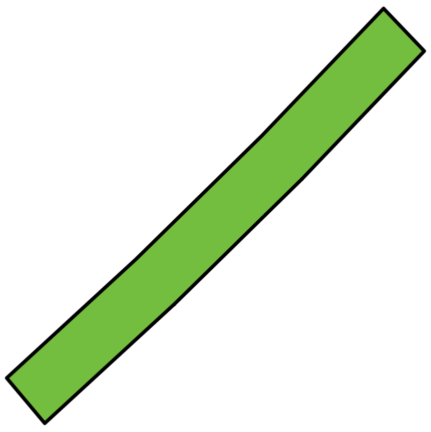
NCTC12052	*Streptococcus pyogenes*	100	1	0	0	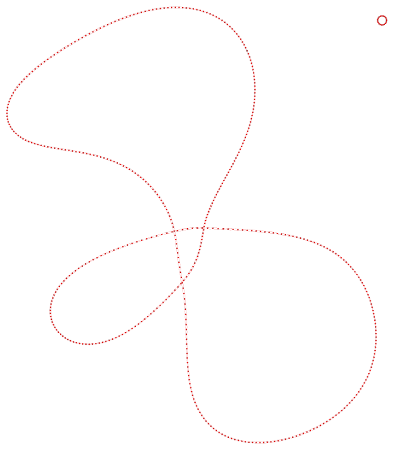	Finished circular assembly	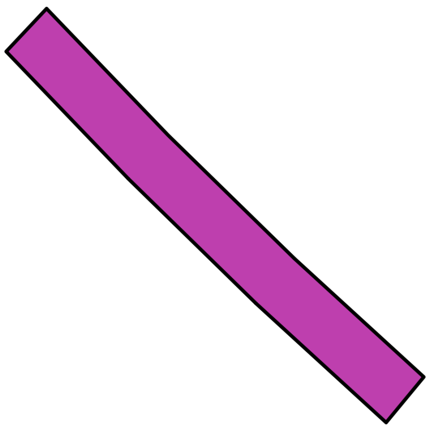
NCTC12054	*Streptococcus pyogenes*	134	1	0	1	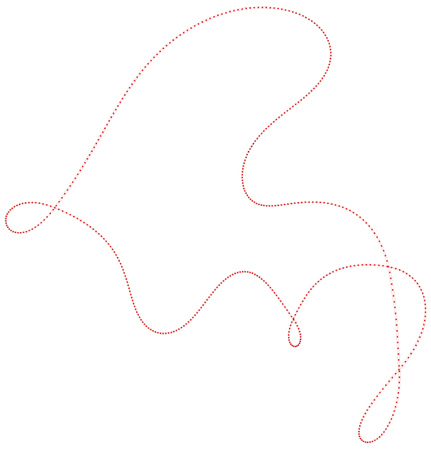	Finished circular assembly	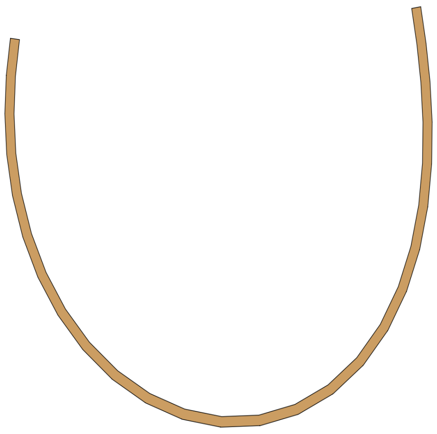
NCTC12057	*Streptococcus pyogenes*	218	1	0	0	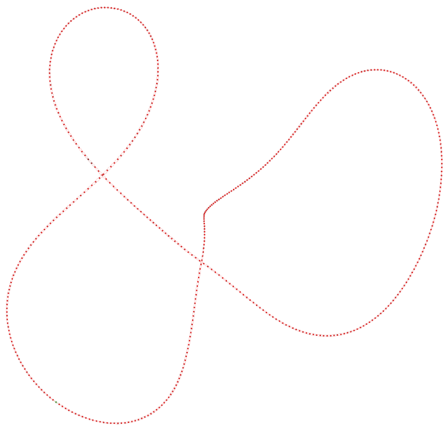	Finished circular assembly	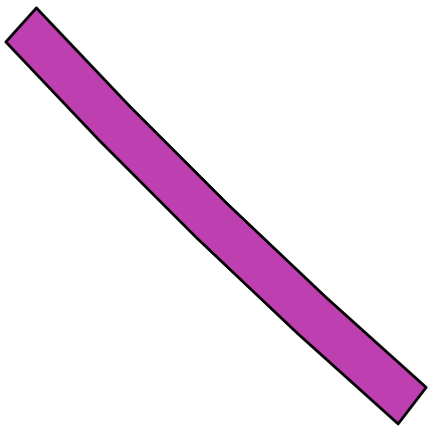
NCTC12058	*Streptococcus pyogenes*	173	1	0	0	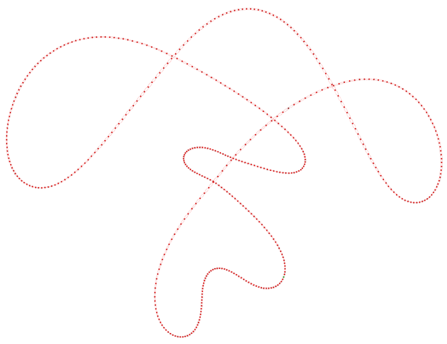	Finished circular assembly	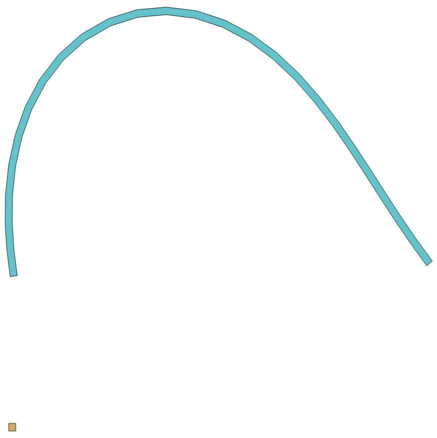
NCTC12059	*Streptococcus pyogenes*	86	1	0	0		Finished circular assembly	

						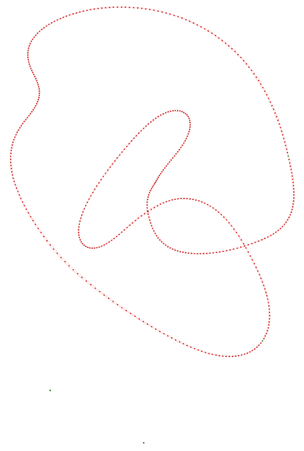		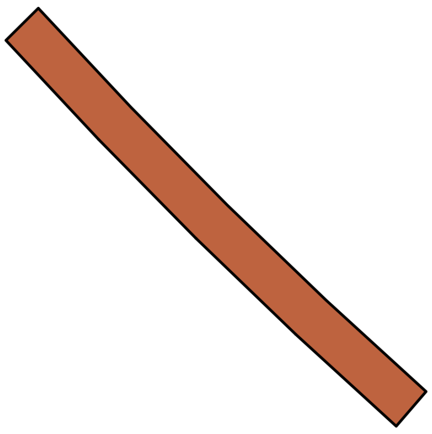
NCTC12060	*Streptococcus pyogenes*	60	0	0	1	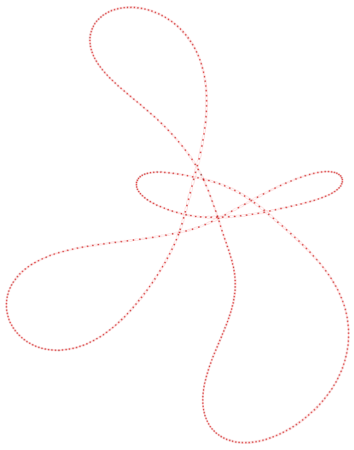	Finished circular assembly	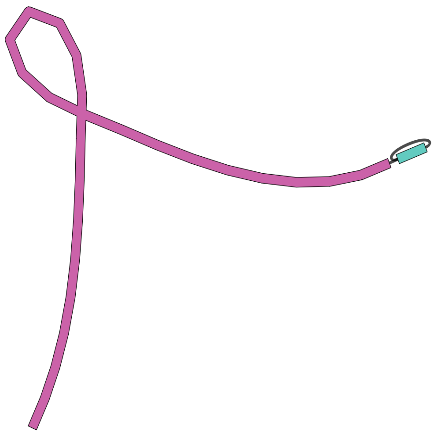
NCTC12061	*Streptococcus pyogenes*	192	1	0	1	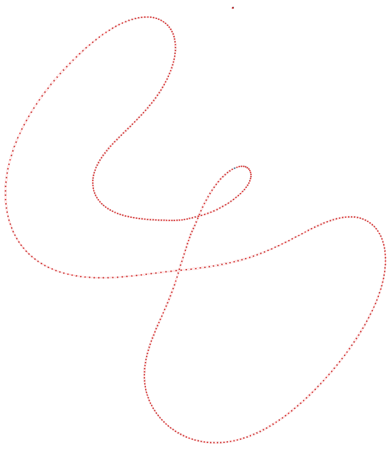	Finished circular assembly	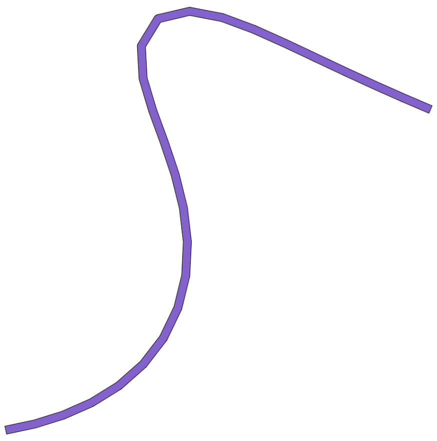
NCTC12062	*Streptococcus pyogenes*	161	1	0	0	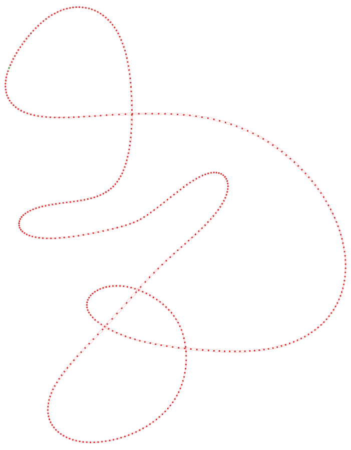	Finished circular assembly	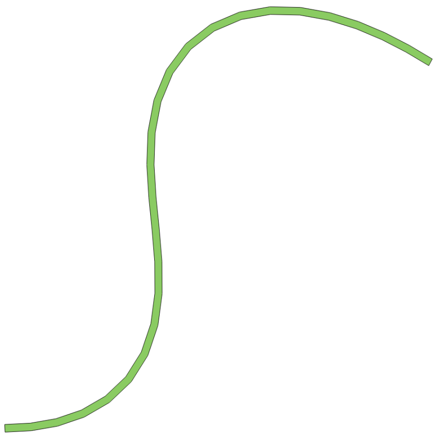
NCTC12064	*Streptococcus pyogenes*	127	1	0	0	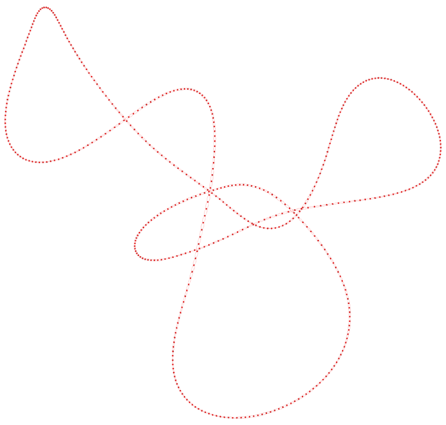	Finished circular assembly	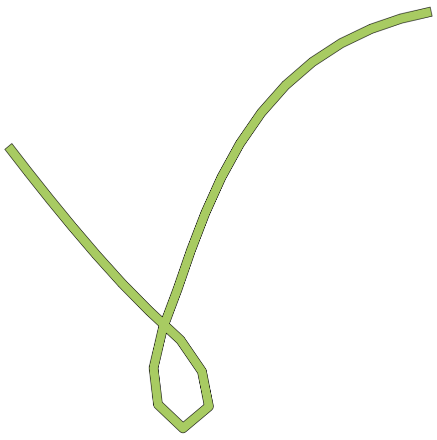
NCTC12065	*Streptococcus pyogenes*	203	1	0	1	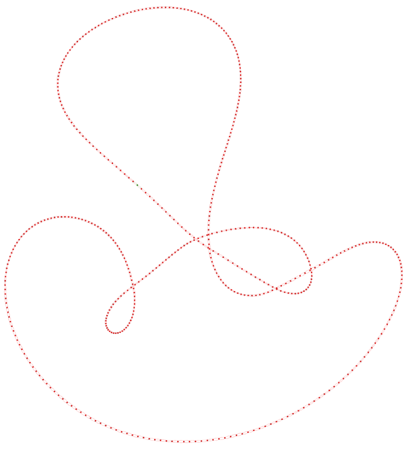	Finished circular assembly	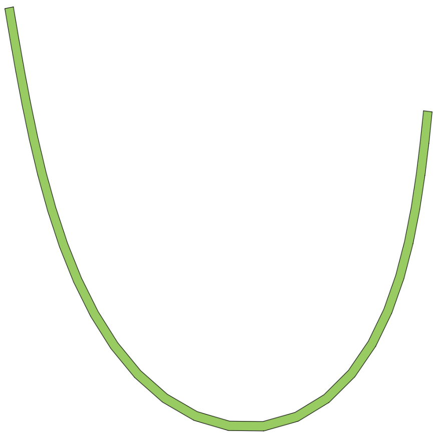
NCTC12066	*Streptococcus pyogenes*	128	1	0	0		Finished circular assembly	

						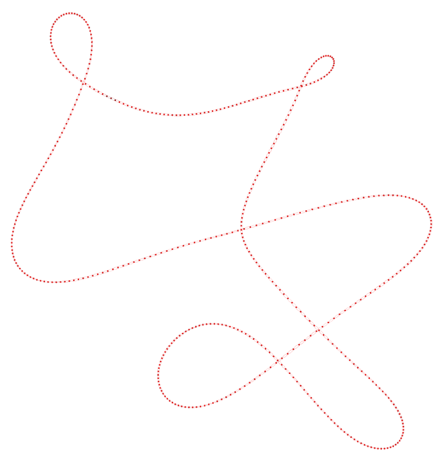		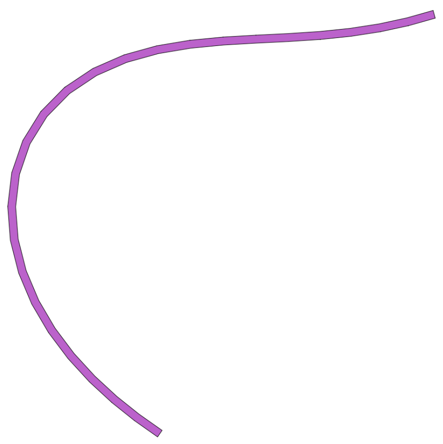	
NCTC12067	*Streptococcus pyogenes*	188	1	0	0	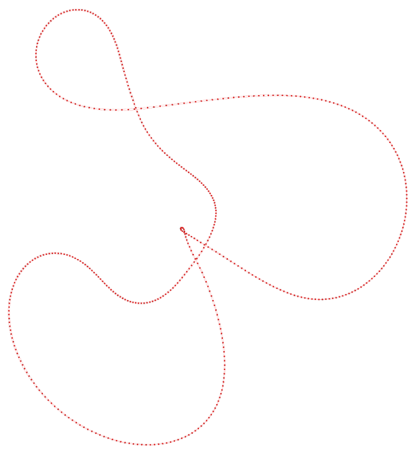	Finished circular assembly	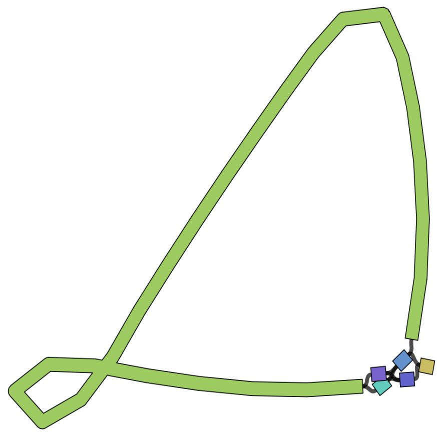	
NCTC12068	*Streptococcus pyogenes*	220	1	0	0	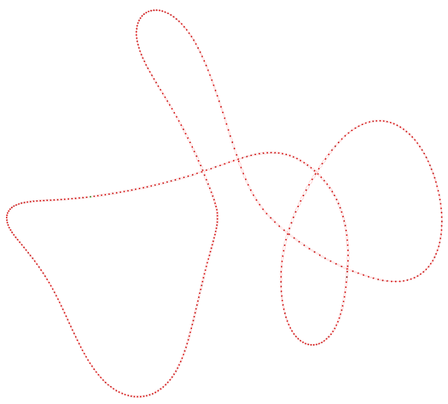	Finished circular assembly	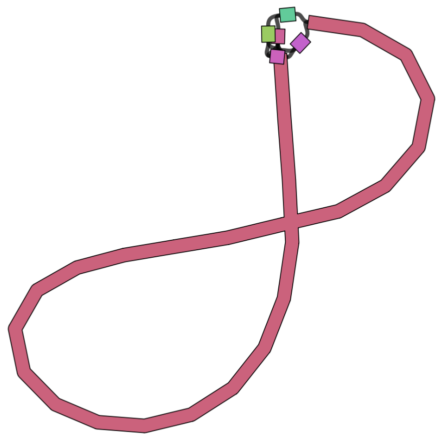	
NCTC12069	*Streptococcus pyogenes*	185	1	0	0	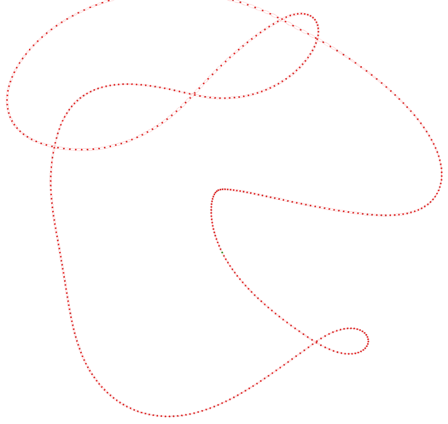	Finished circular assembly	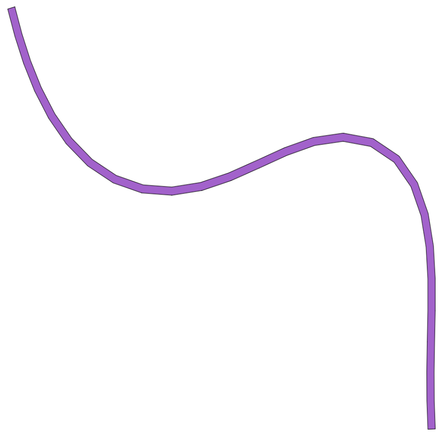	
NCTC12090	*Streptococcus equi*	0	1	0	0	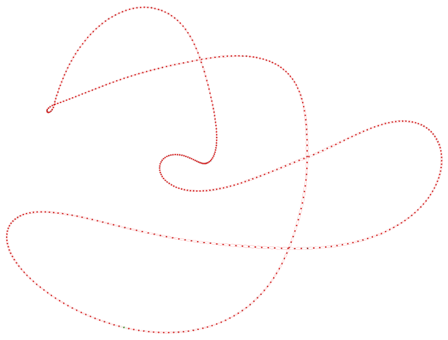	Finished circular assembly	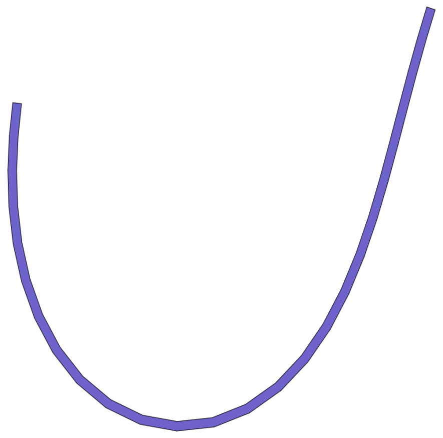	
NCTC12091	*Streptococcus equi*	111	1	0	0	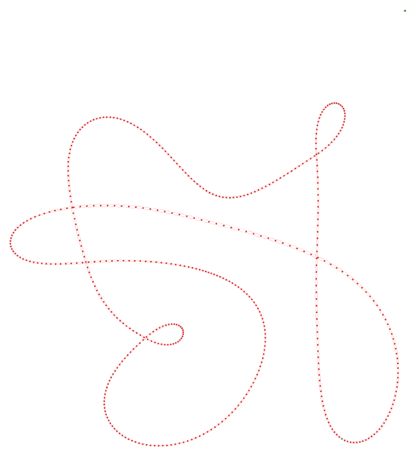	Finished circular assembly	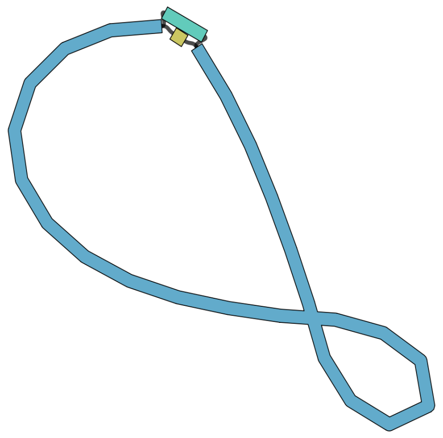	
NCTC12092	*Streptococcus equi*	85	Pending	Pending	Pending		Finished circular assembly		

						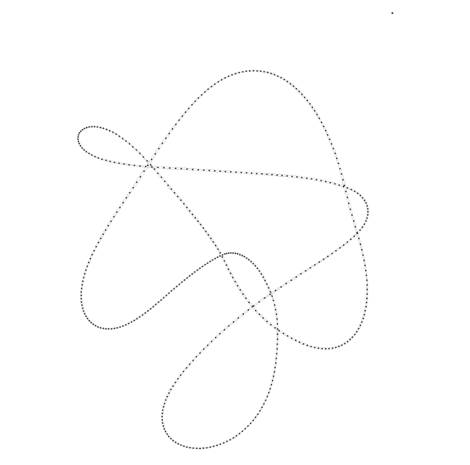		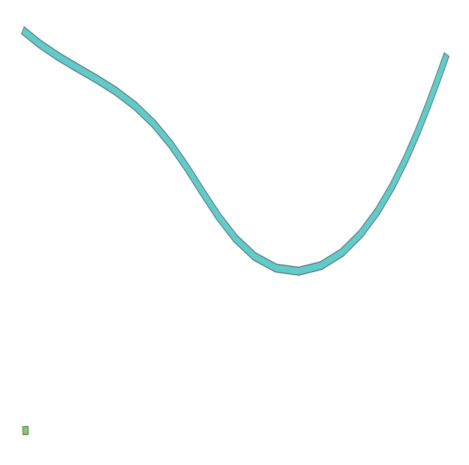
NCTC12100	*Staphylococcus epidermidis*	88	Pending	Pending	Pending	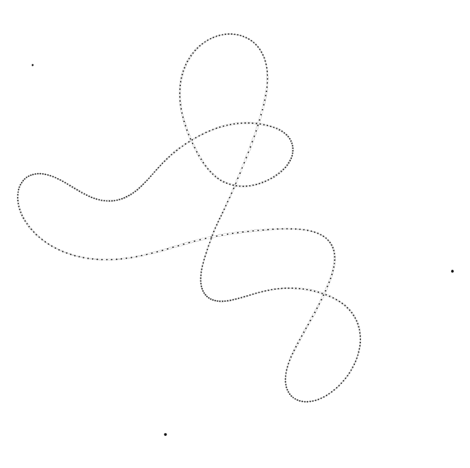	Finished circular assembly	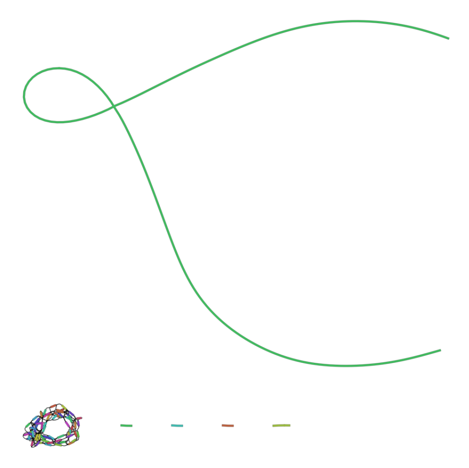
NCTC12101	*Staphylococcus auricularis*	101	1	0	0	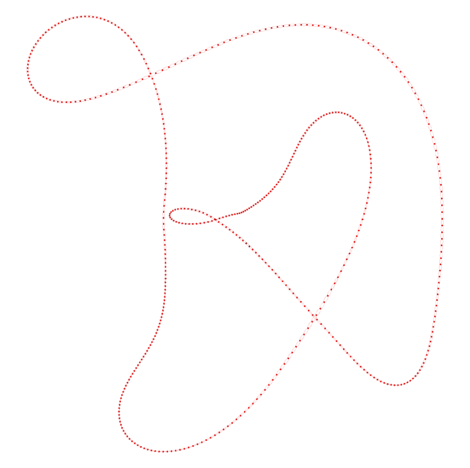	Finished circular assembly	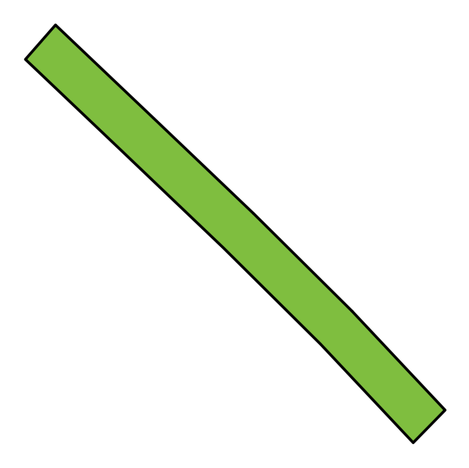
NCTC12103	*Staphylococcus sciuri subsp. sciuri*	143	1	0	0	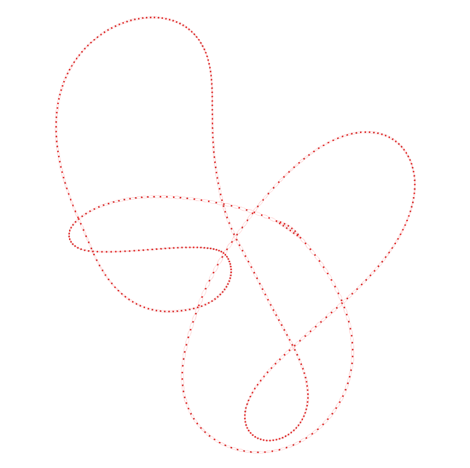	Finished circular assembly	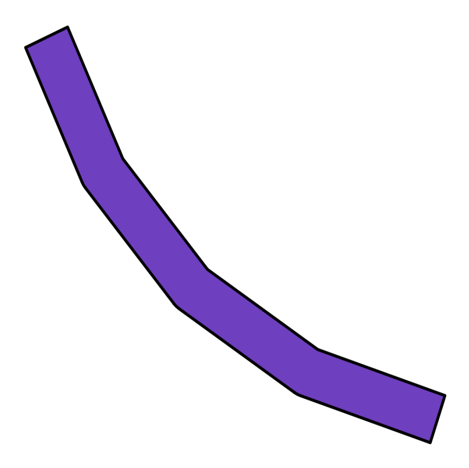
NCTC12112	*Fusobacterium ulcerans*	71	0	0	1	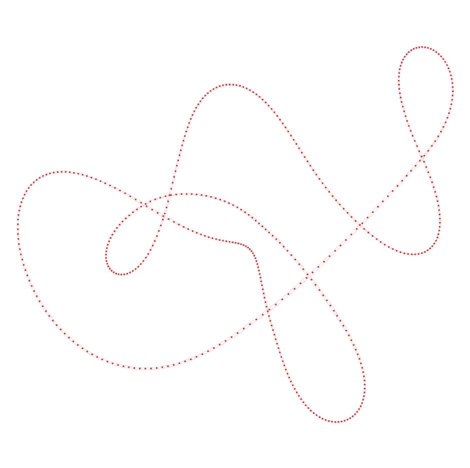	Finished circular assembly	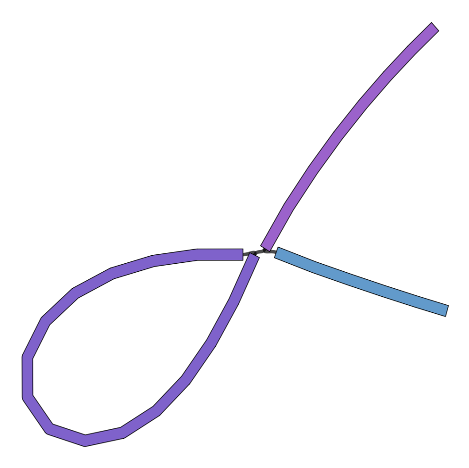
NCTC12116	*Salmonella enterica subsp. enterica*	40	Pending	Pending	Pending	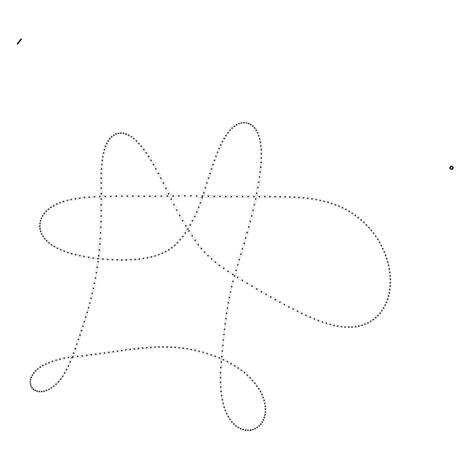	Finished circular assembly	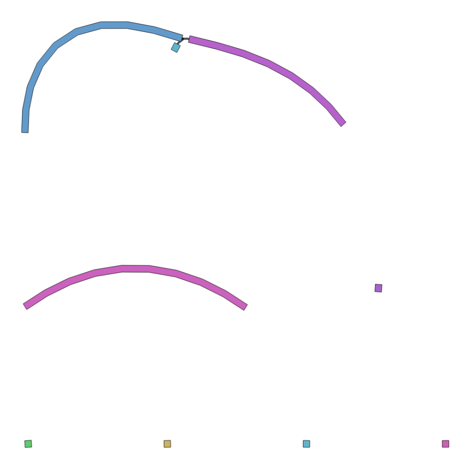
NCTC12120	*Cedecea neteri*	74	7	1	0		Mis-assembly/Fragmented	

						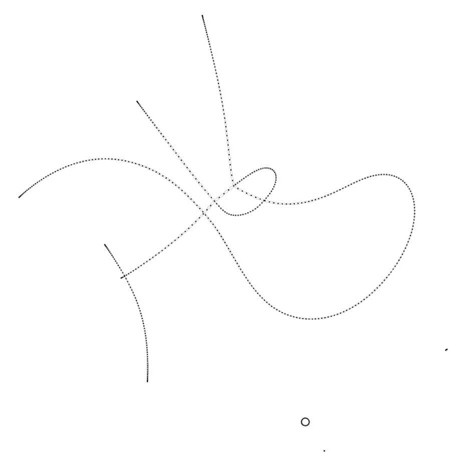		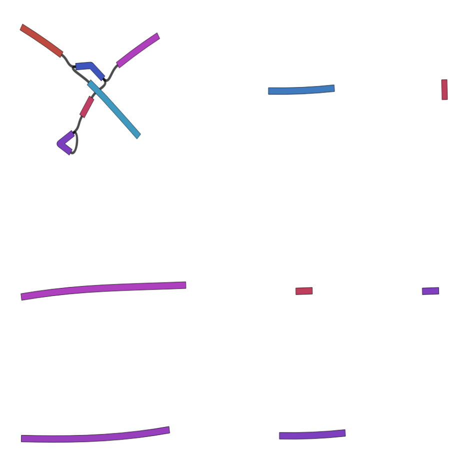
NCTC12123	*Enterobacter asburiae*	64	2	3	0	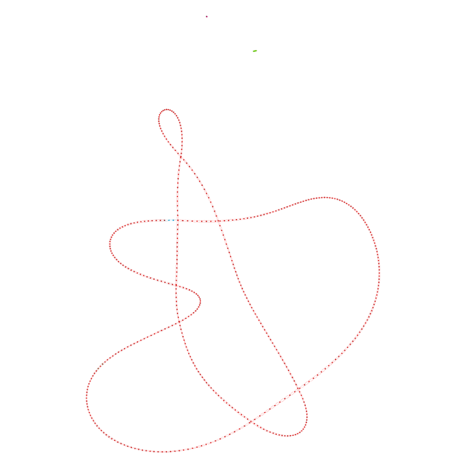	Finished assembly with multiple traversals	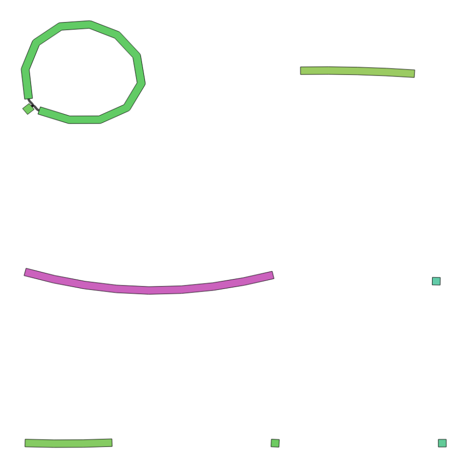
NCTC12124	*Enterobacter amnigenus*	38	1	0	0	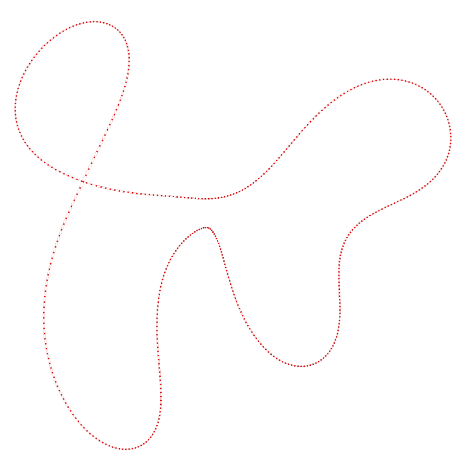	Finished circular assembly	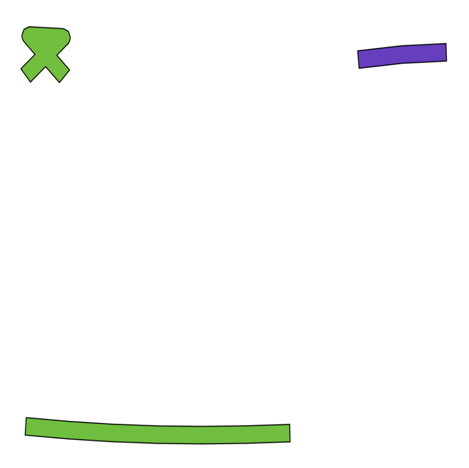
NCTC12125	*Kluyvera intermedia*	90	1	0	0	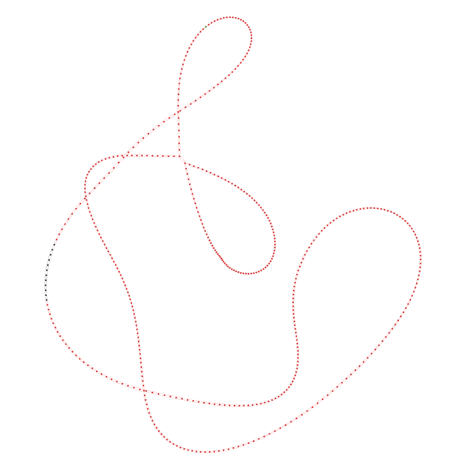	Finished assembly with multiple traversals	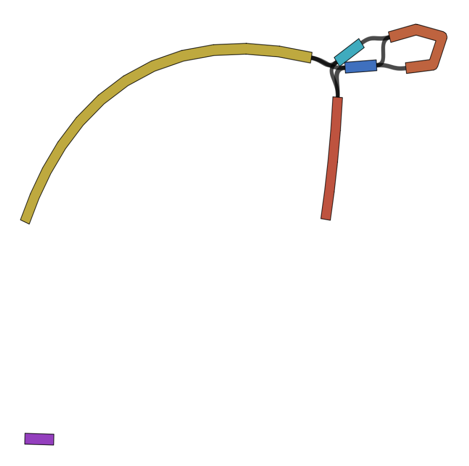
NCTC12126	*Enterobacter cancerogenus*	42	6	1	0	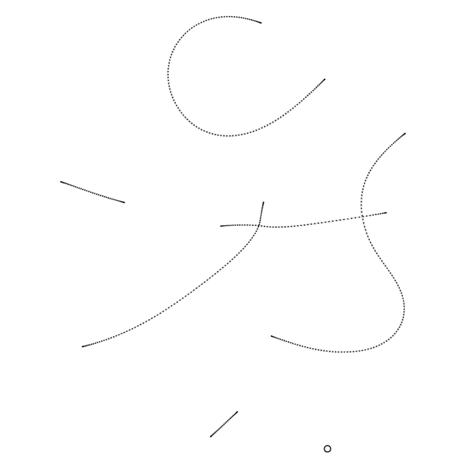	Mis-assembly/Fragmented	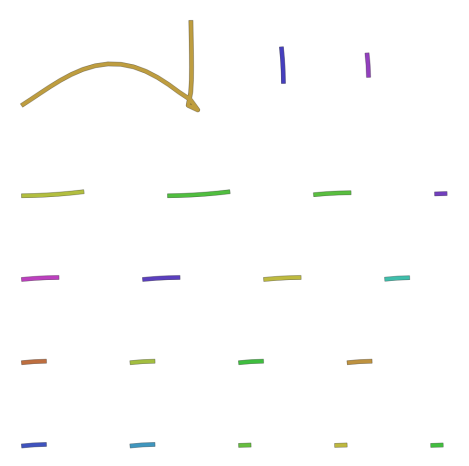
NCTC12127	*Shimwellia blattae*	79	1	0	0	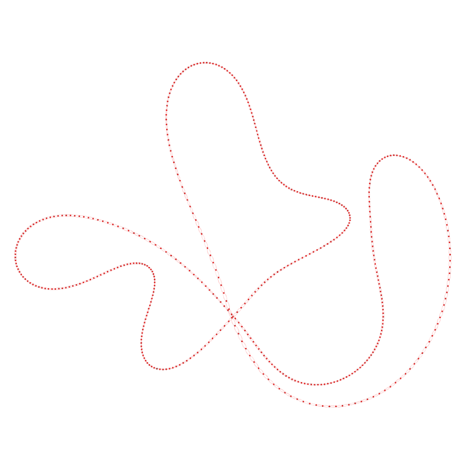	Finished circular assembly	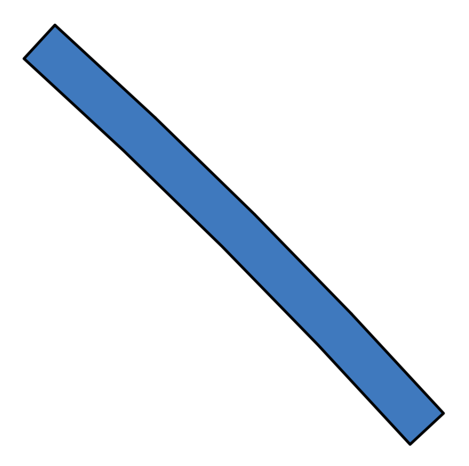
NCTC12128	*Escherichia fergusonii*	46	1	1	0		Finished circular assembly	

						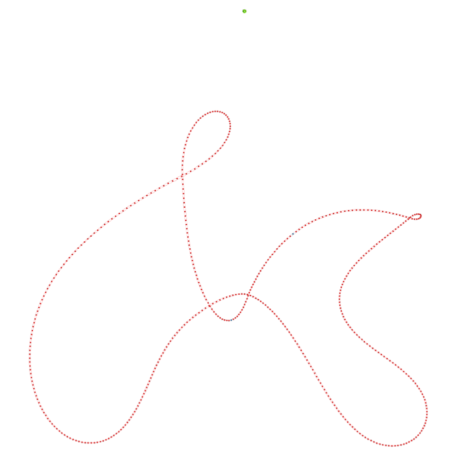		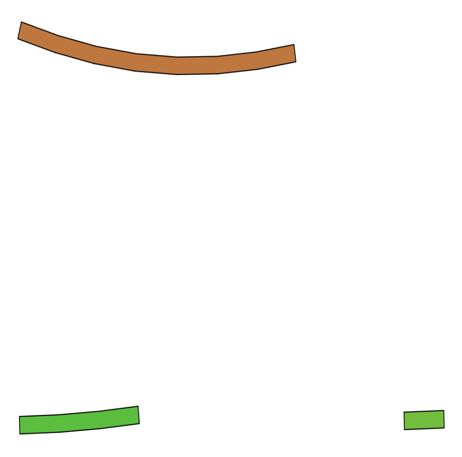
NCTC12129	*Escherichia hermannii*	70	1	0	0	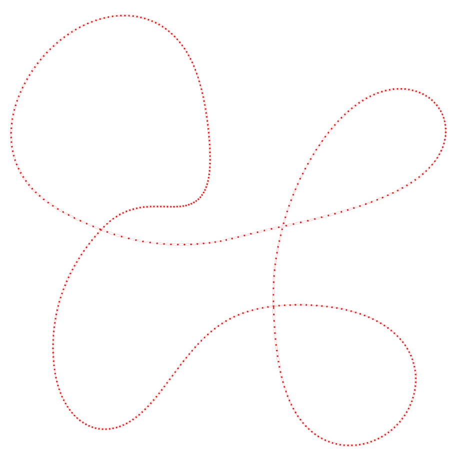	Finished circular assembly	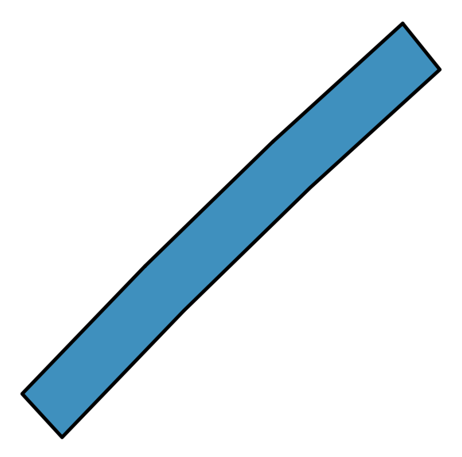
NCTC12130	*Escherichia vulneris*	62	1	0	0	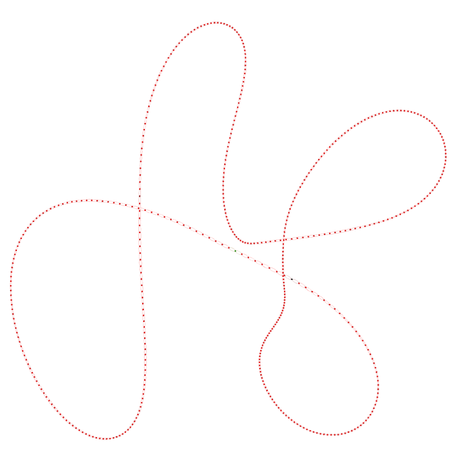	Finished circular assembly	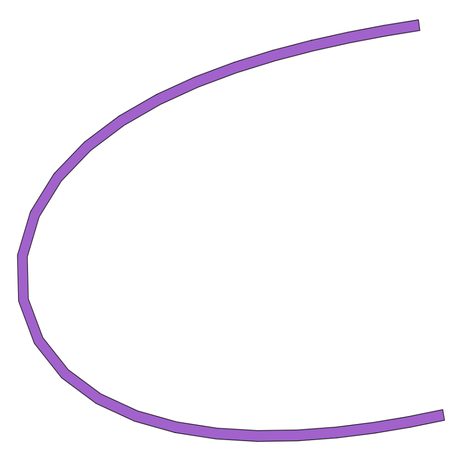
NCTC12131	*Yokenella regensburgei*	41	3	0	0	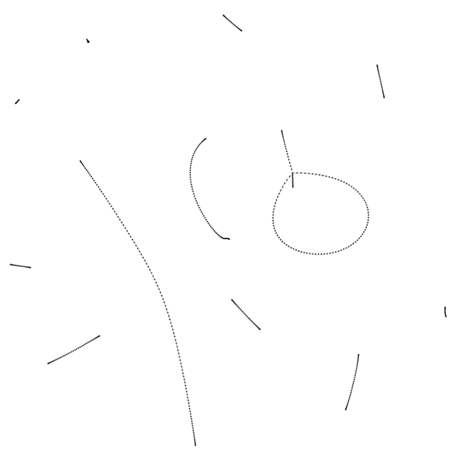	Mis-assembly/Fragmented	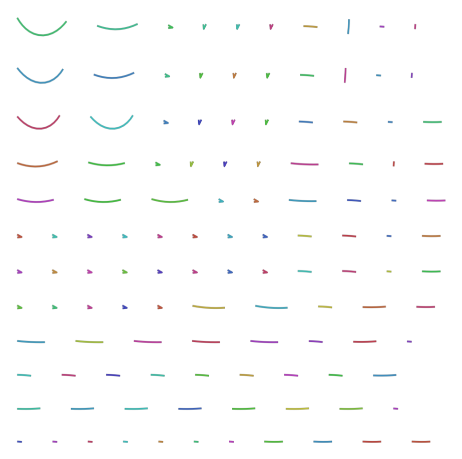
NCTC12132	*Moellerella wisconsensis*	86	1	0	0	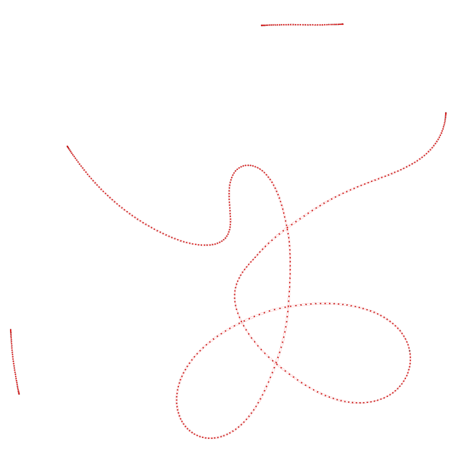	Mis-assembly/Fragmented	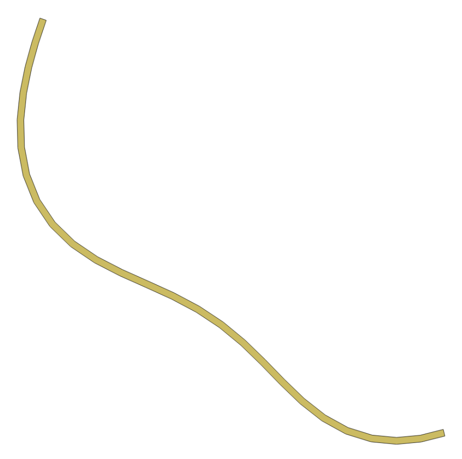
NCTC12143	*Campylobacter coli*	16	0	0	3	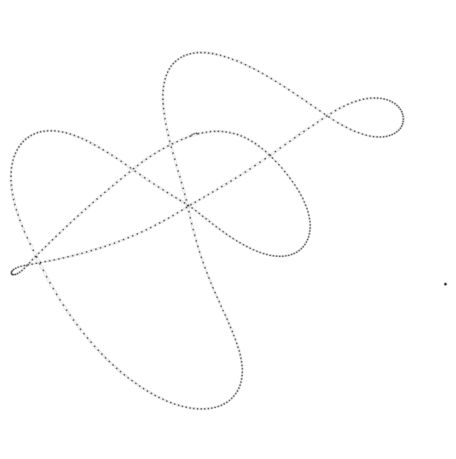	Finished circular assembly	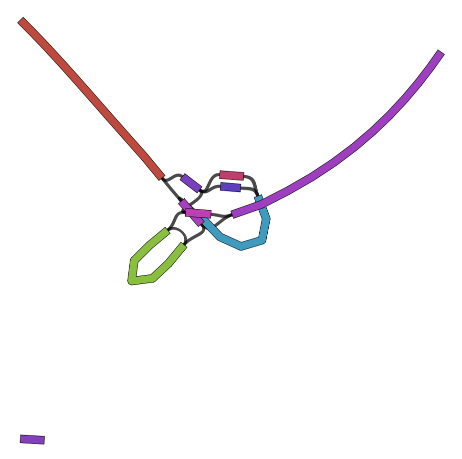
NCTC12146	*Klebsiella terrigena*	11	0	0	2		Mis-assembly/Fragmented	

						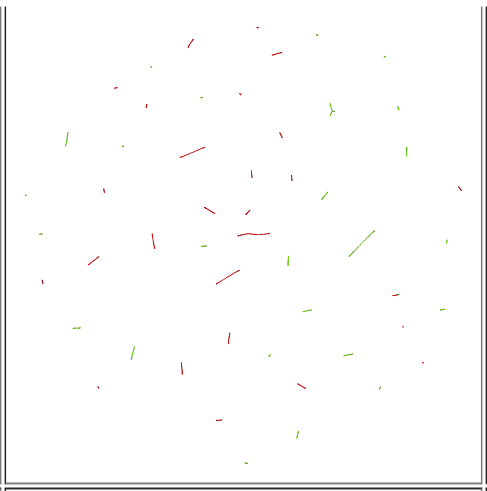		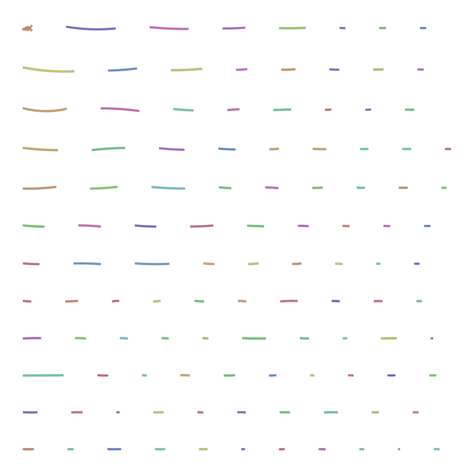
NCTC12151	*Leminorella richardii*	51	1	0	0	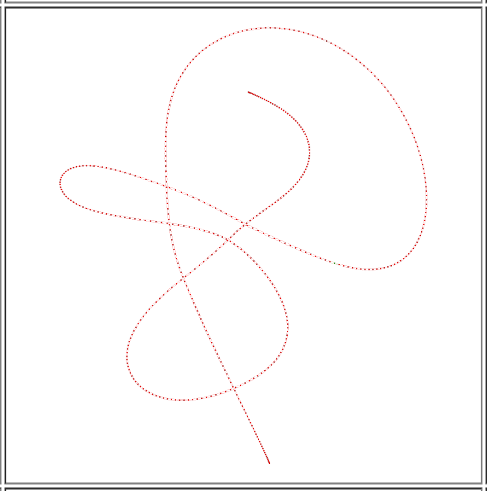	Finished assembly (lacking circularisation)	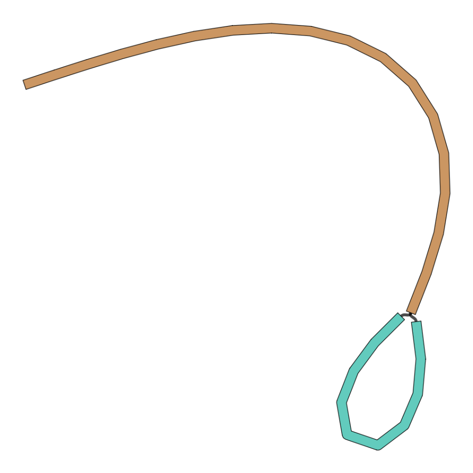
NCTC12152	*Leminorella grimontii*	88	1	0	0	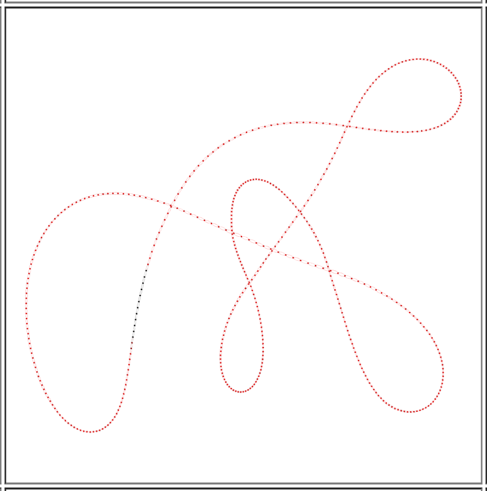	Finished circular assembly	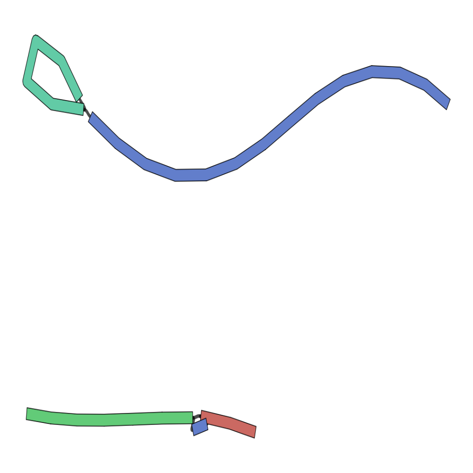
NCTC12156	*Acinetobacter baumannii*	123	1	0	1	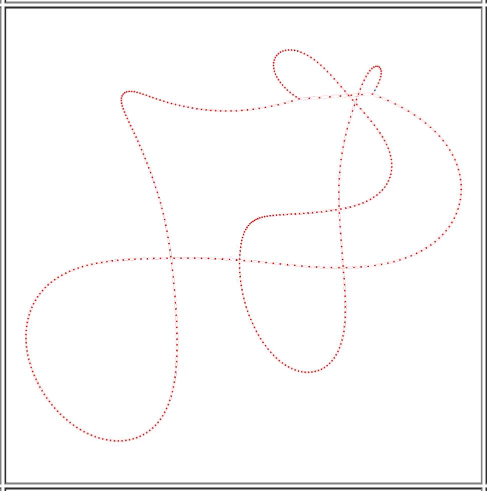	Mis-assembly/Fragmented	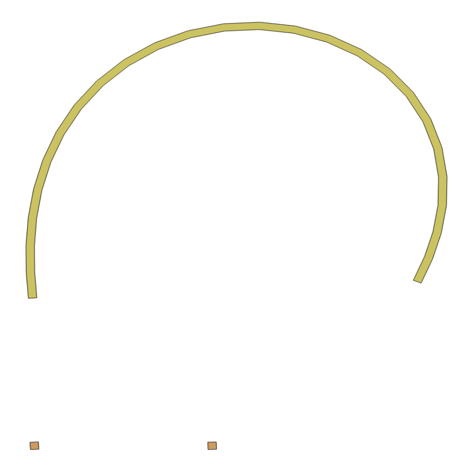
NCTC12157	*Ewingella americana*	76	1	1	0	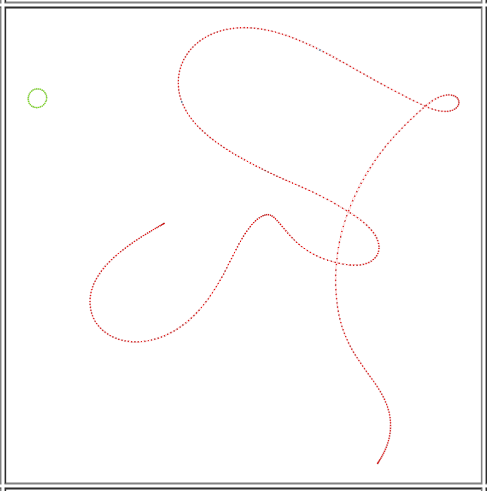	Finished assembly (lacking circularisation)	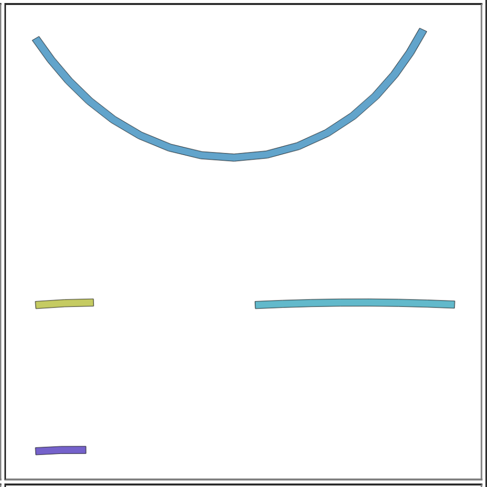
NCTC12158	*Klebsiella planticola*	16	0	0	367	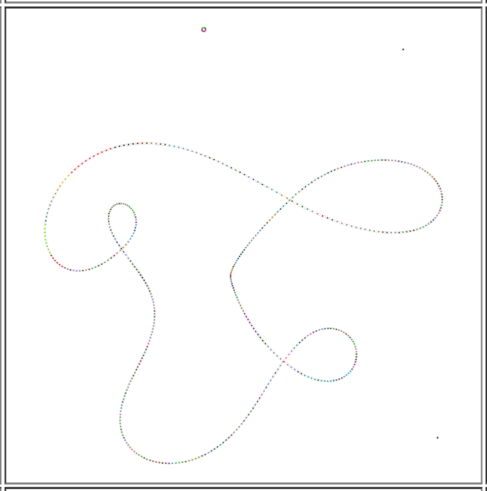	Finished circular assembly	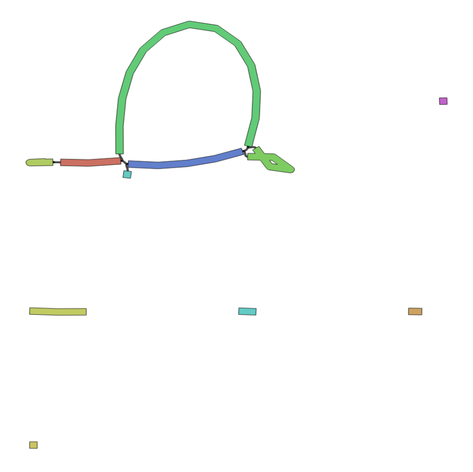
NCTC12167	*Streptococcus vestibularis*	111	1	0	0		Finished circular assembly	

						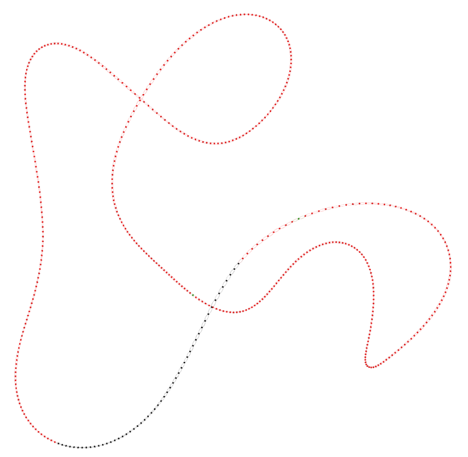		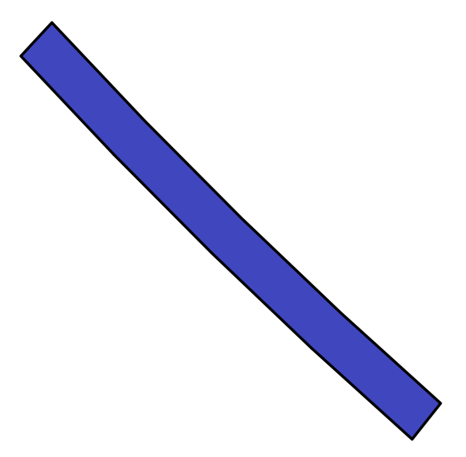
NCTC12168	*Ochrobactrum anthropi*	60	2	0	3	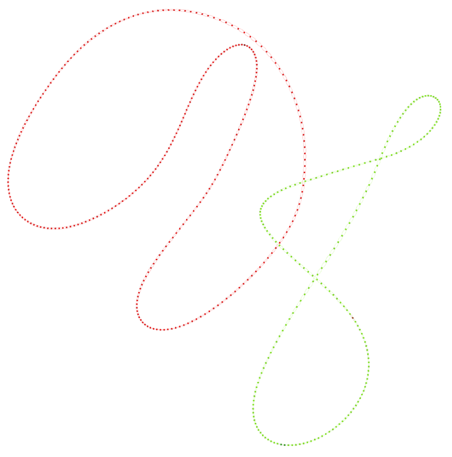	Finished circular assembly	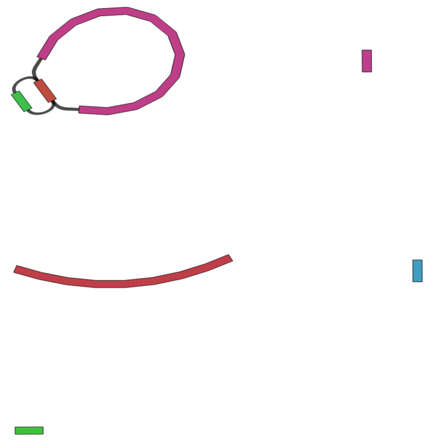
NCTC12171	*Ochrobactrum intermedium*	64	2	1	0	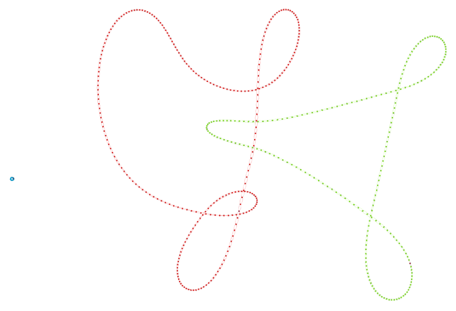	Finished circular assembly	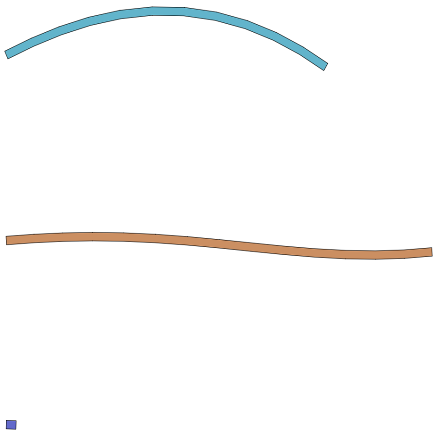
NCTC12179	*Legionella pneumophila*	33	Pending	Pending	Pending	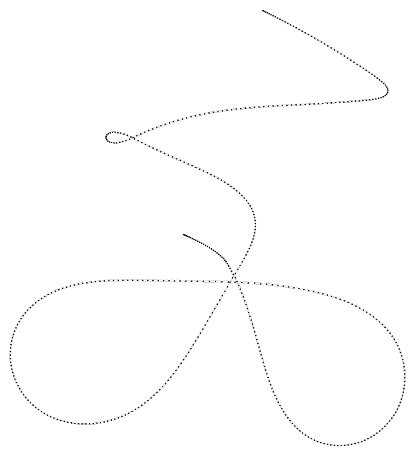	Finished assembly (lacking circularisation)	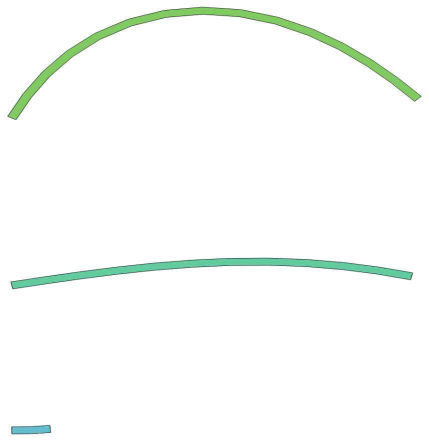
NCTC12180	*Legionella pneumophila*	47	1	0	1	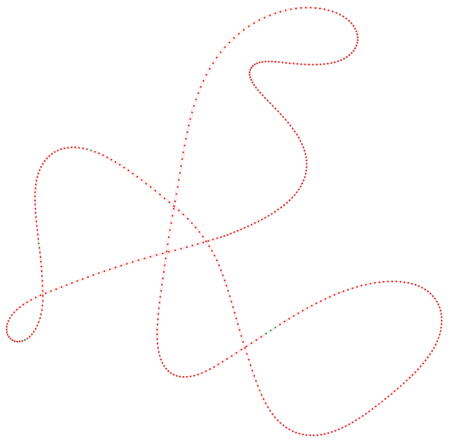	Finished circular assembly	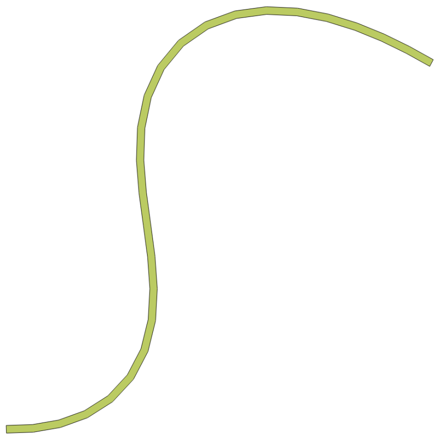
NCTC12194	*Haemophilus influenzae*	83	1	0	0	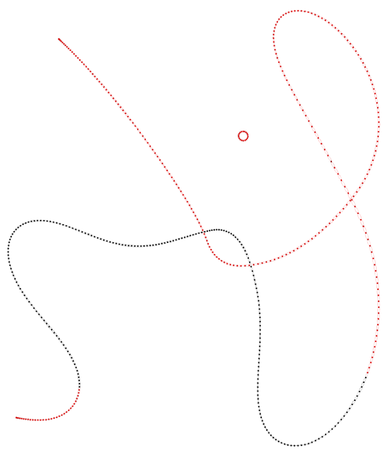	Finished assembly (lacking circularisation)	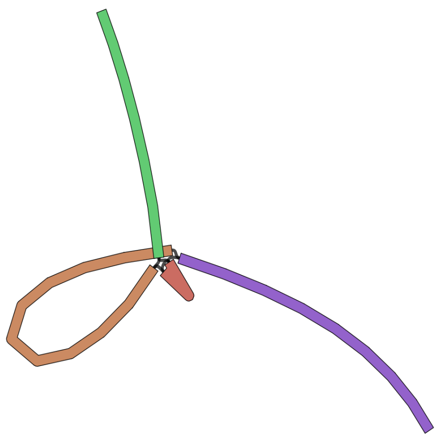
NCTC12195	*Staphylococcus gallinarum*	33	0	0	5		Mis-assembly/Fragmented	

						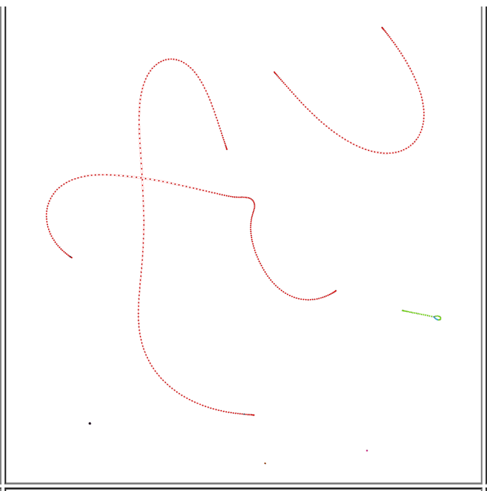		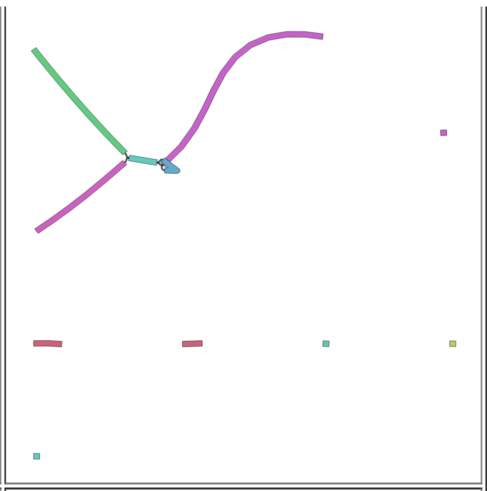
NCTC12196	*Staphylococcus caprae*	53	1	0	4	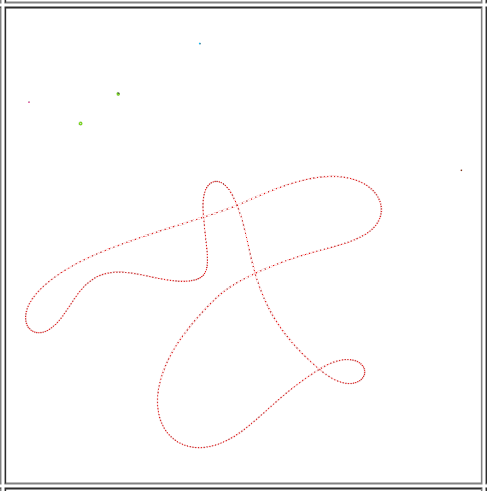	Finished circular assembly	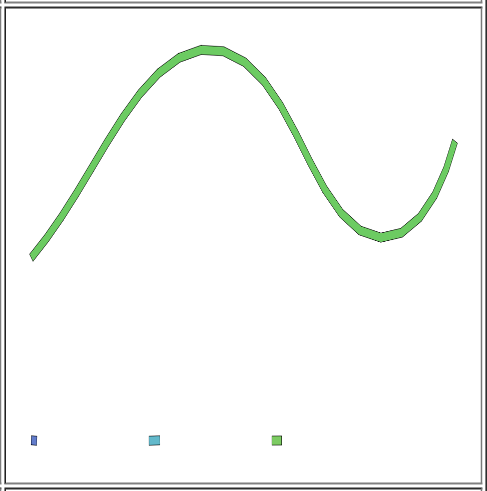
NCTC12217	*Staphylococcus lugdenensis*	74	1	0	0	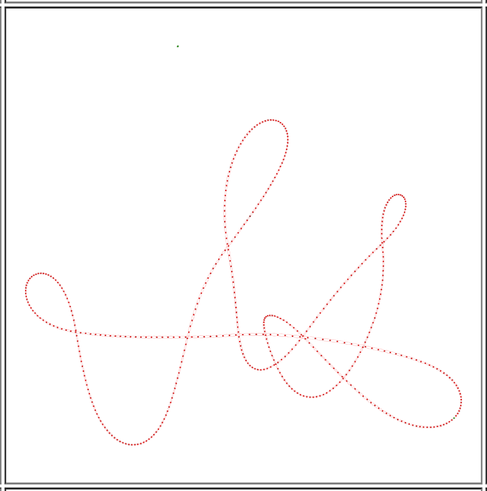	Finished circular assembly	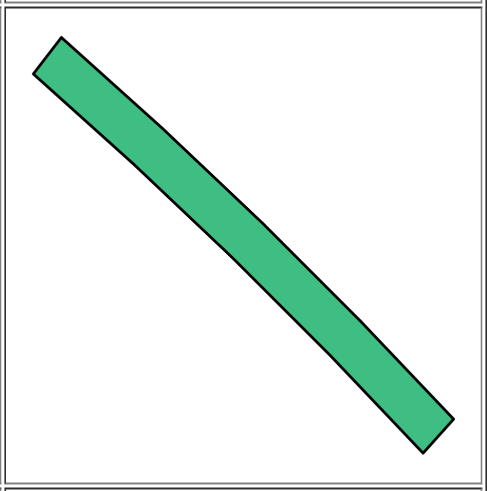
NCTC12218	*Staphylococcus schleiferi*	49	Pending	Pending	Pending	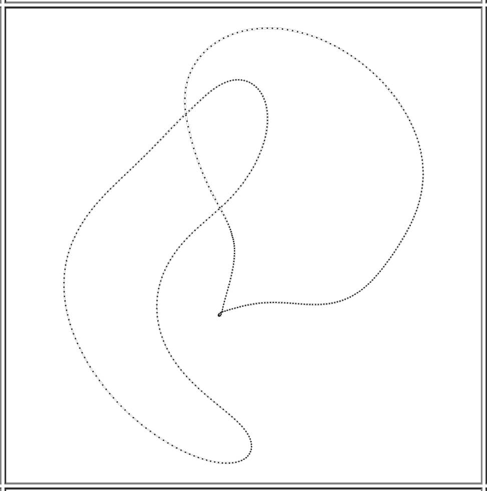	Finished circular assembly	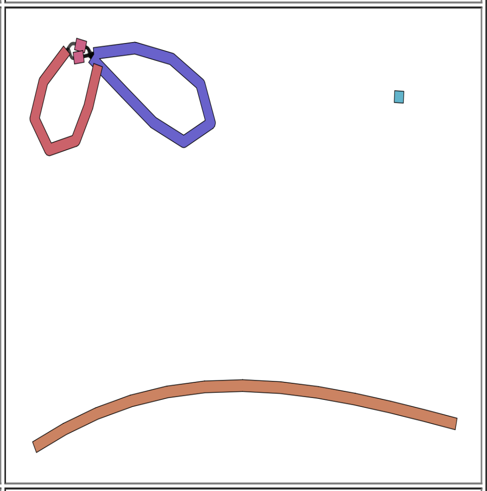
NCTC12224	*Streptococcus hyointestinalis*	38	1	1	5	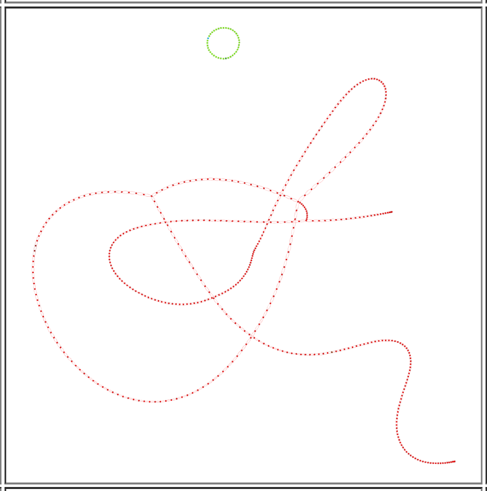	Mis-assembly/Fragmented	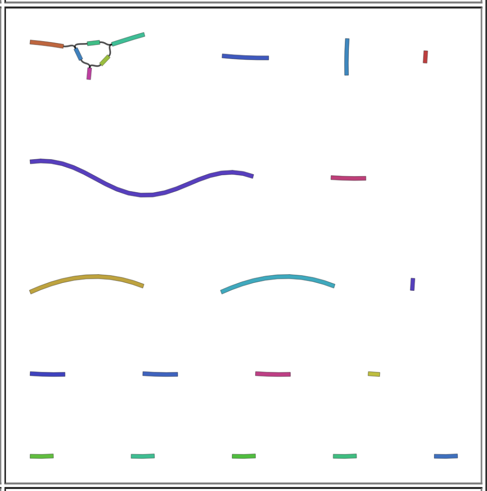
NCTC12225	*Staphylococcus delphini*	107	1	0	0	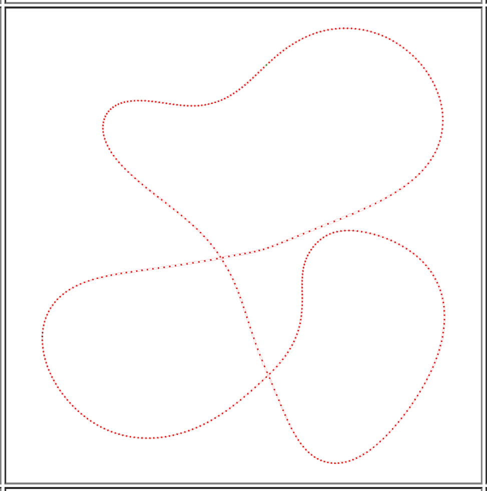	Finished circular assembly	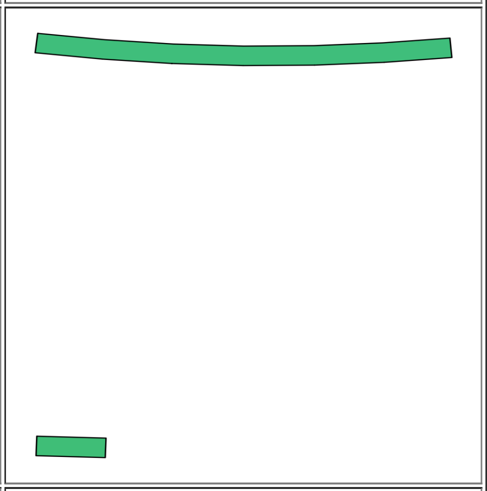
NCTC12232	*Staphylococcus aureus*	86	1	1	0		Finished circular assembly	

						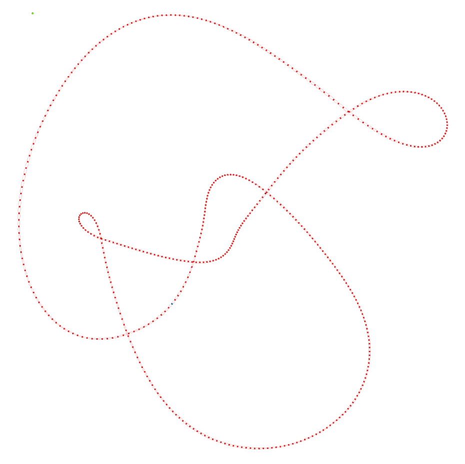		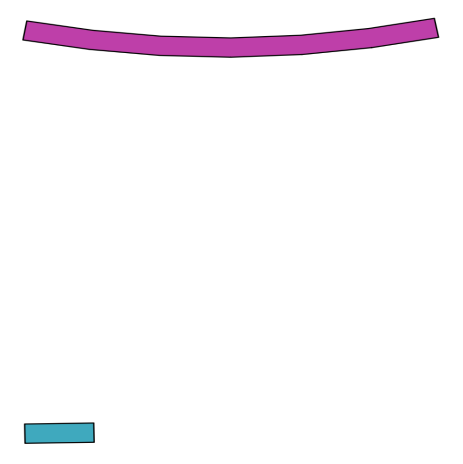
NCTC12233	*Staphylococcus aureus*	99	1	0	0	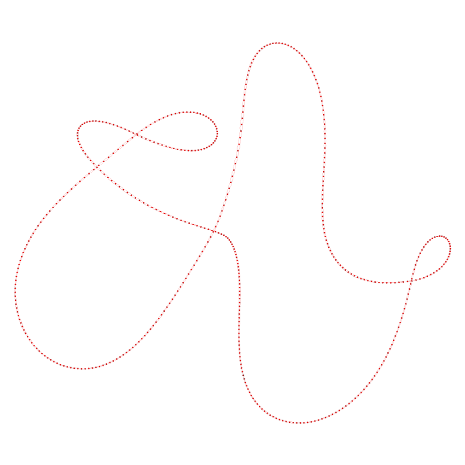	Finished circular assembly	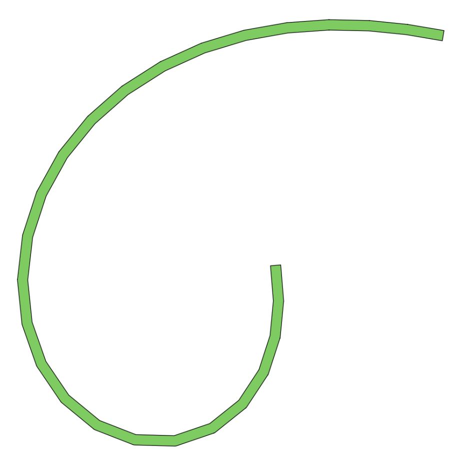
NCTC12241	*Escherichia coli*	50	1	1	1	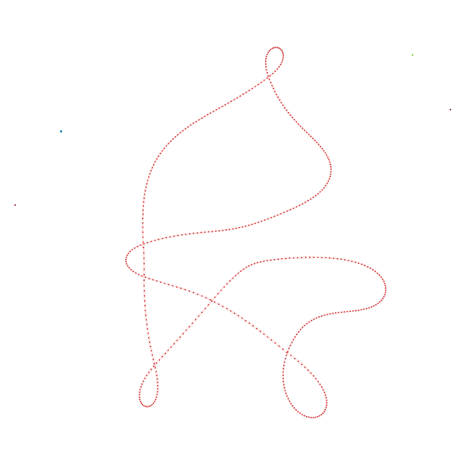	Finished circular assembly	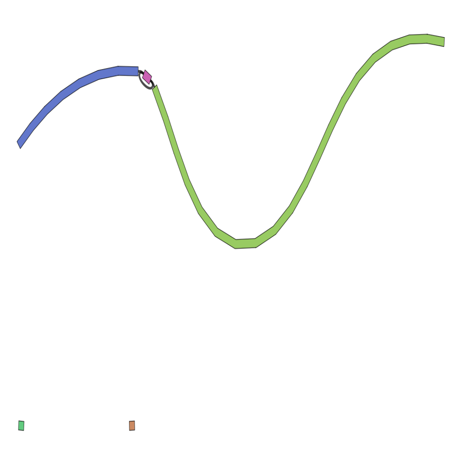
NCTC12261	*Streptococcus mitis*	39	Pending	Pending	Pending	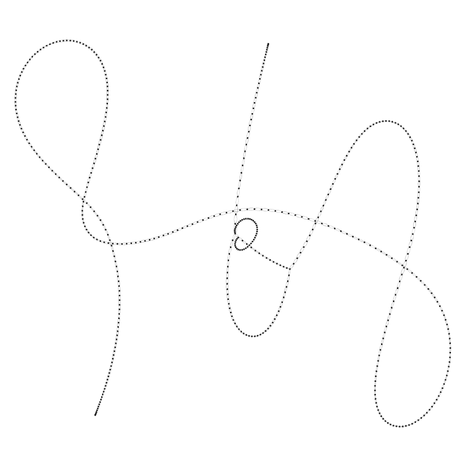	Mis-assembly/Fragmented	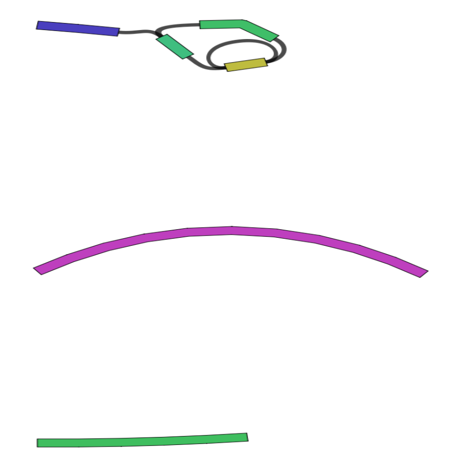
NCTC12278	*Streptococcus ferus*	185	1	0	0	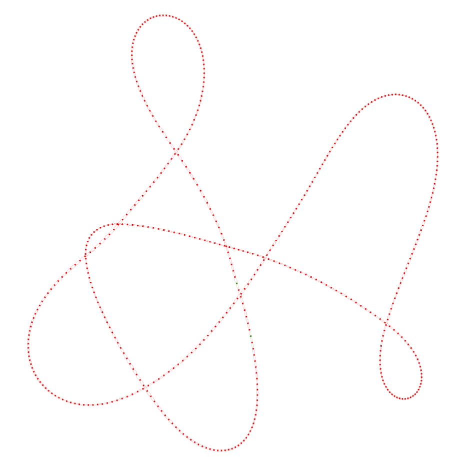	Finished circular assembly	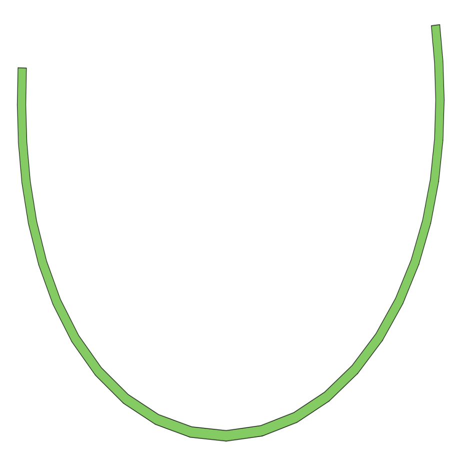
NCTC12282	*Budvicia aquatica*	31	2	0	0	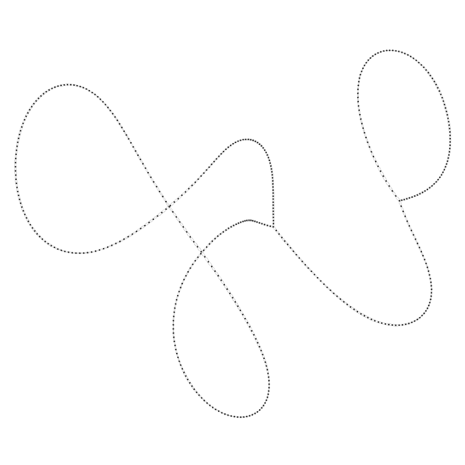	Mis-assembly/Fragmented	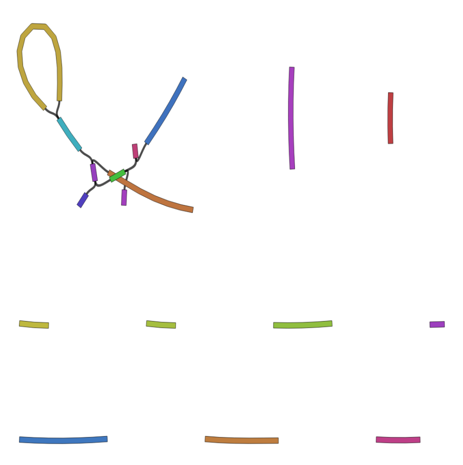
NCTC12413	*Staphylococcus arlettae*	91	1	1	3		Finished circular assembly	

						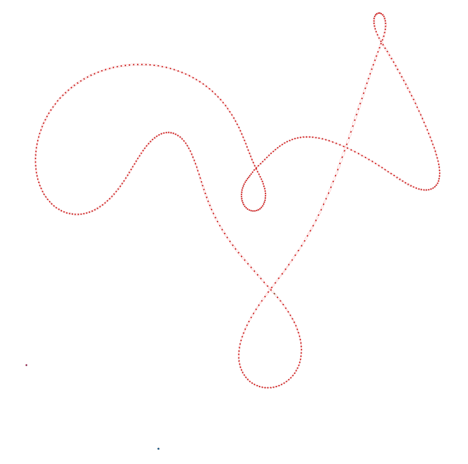		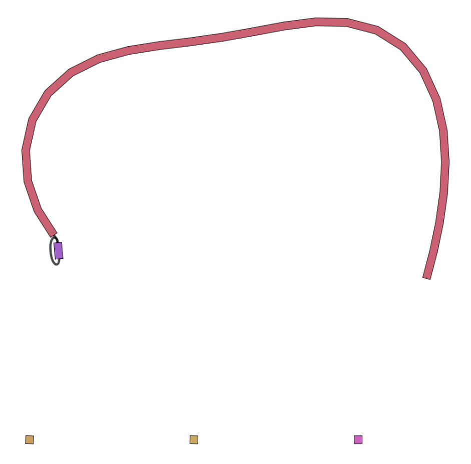
NCTC12415	*Staphylococcus kloosii*	179	1	1	0	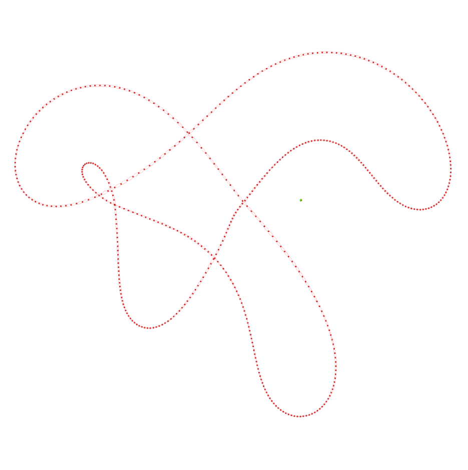	Finished circular assembly	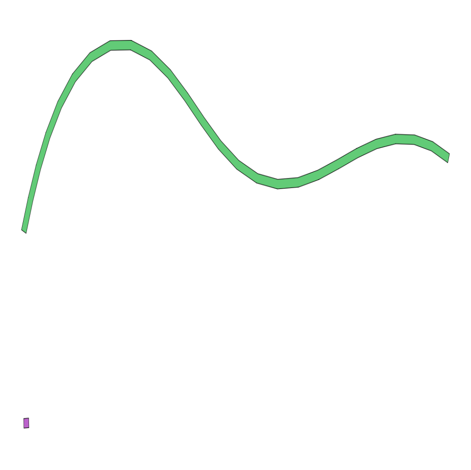
NCTC12416	*Salmonella enterica subsp. enterica*	43	0	0	3	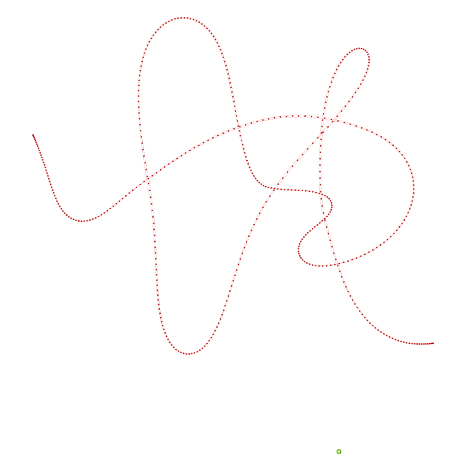	Finished assembly (lacking circularisation)	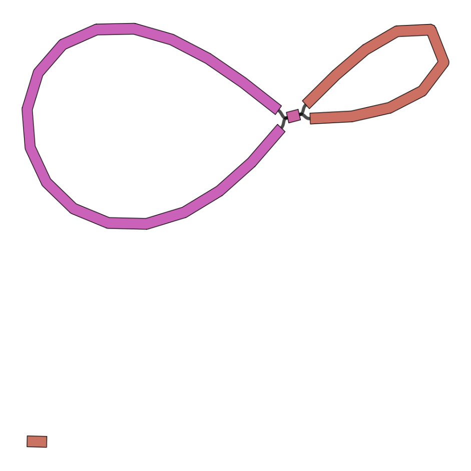
NCTC12417	*Salmonella enterica subsp. diarizonae*	77	1	2	0	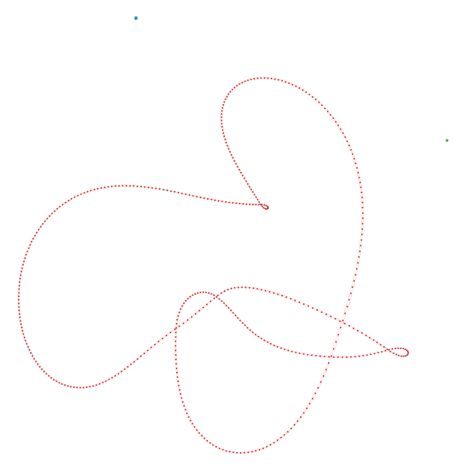	Finished circular assembly	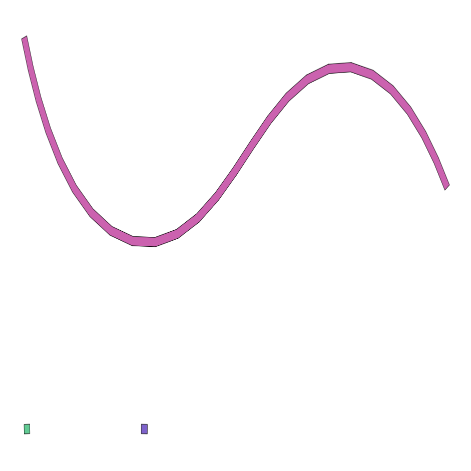
NCTC12418	*Salmonella enterica subsp. houtenae*	28	0	0	2	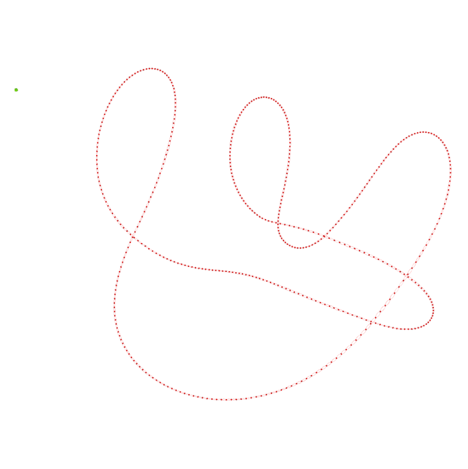	Finished circular assembly	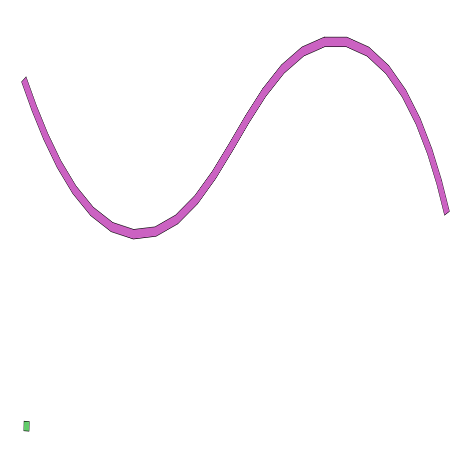
NCTC12419	*Salmonella bongori*	68	1	0	0	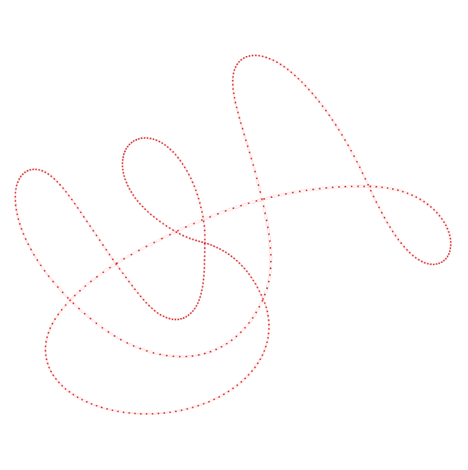	Finished circular assembly	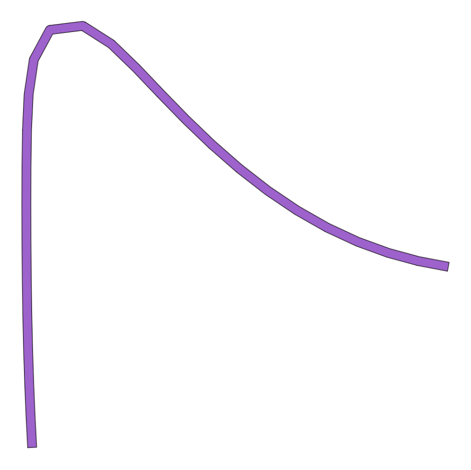
NCTC12420	*Salmonella enterica subsp. indica*	82	3	2	0		Finished circular assembly	

						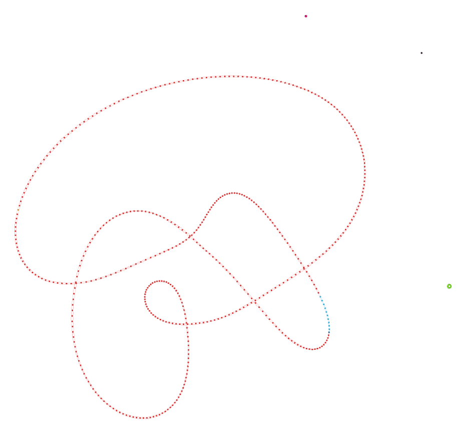		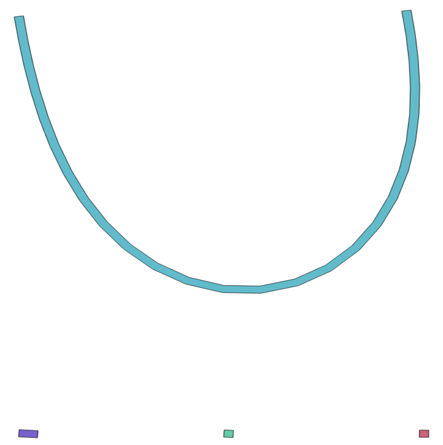
NCTC12421	*Enterococcus cecorum*	81	1	0	0	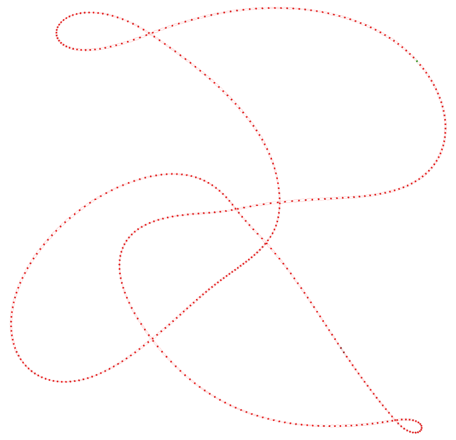	Finished circular assembly	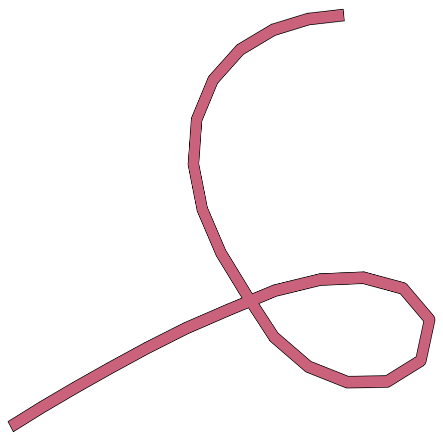
NCTC12450	*Legionella sainthelensi*	37	1	0	0	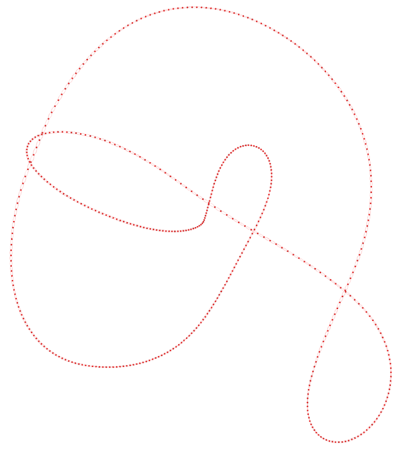	Finished circular assembly	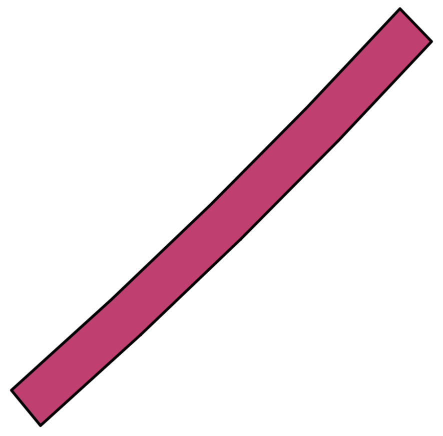
NCTC12463	*Klebsiella pneumoniae*	44	0	0	12	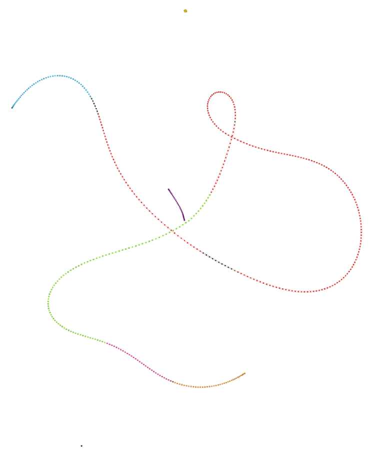	Finished assembly (lacking circularisation)	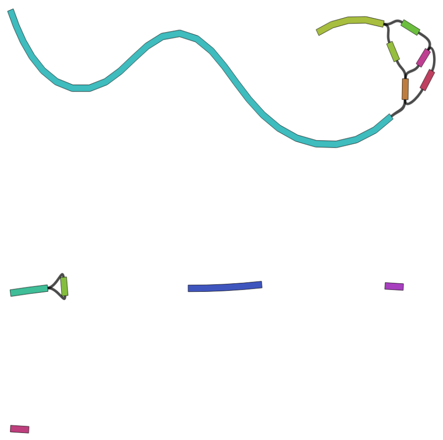
NCTC12479	*Streptococcus cristatus*	102	Pending	Pending	Pending	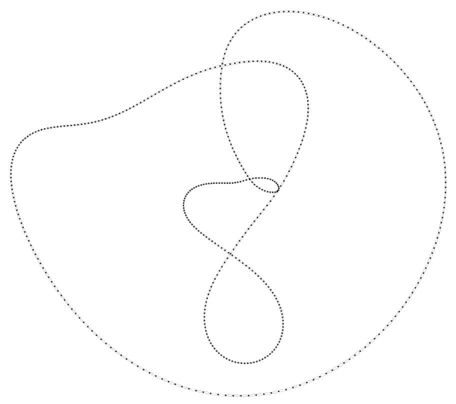	Finished circular assembly	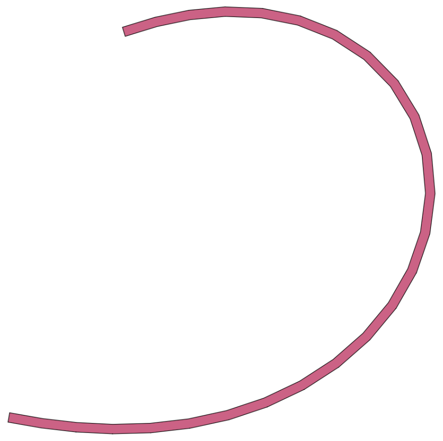
NCTC12495	*Streptococcus pneumoniae*	10	Pending	Pending	Pending	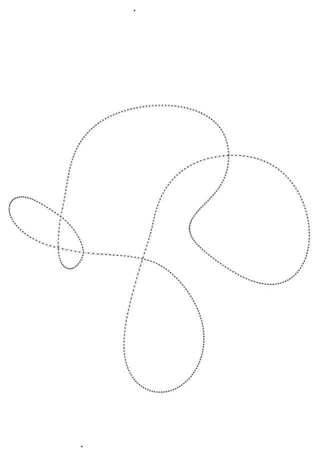	Finished circular assembly	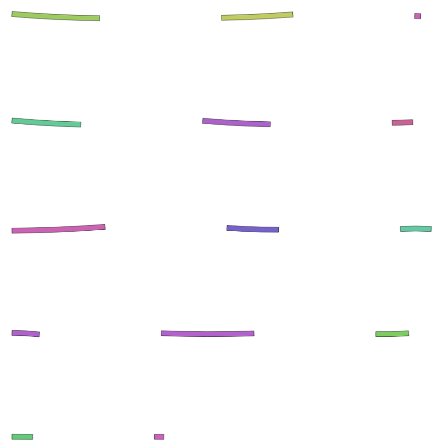
NCTC12570	*Campylobacter coli*	228	1	1	0		Finished circular assembly	

						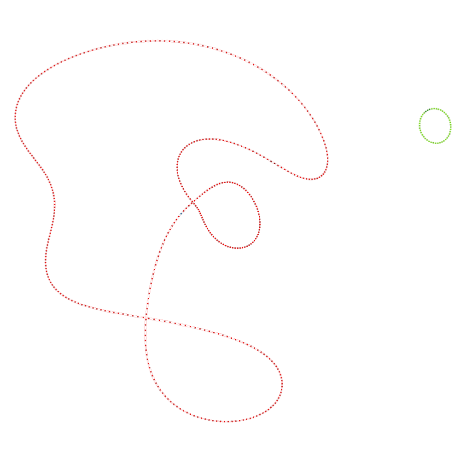		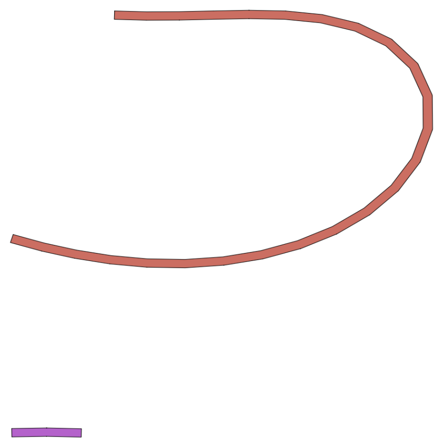
NCTC12650	*Escherichia coli*	37	1	2	0	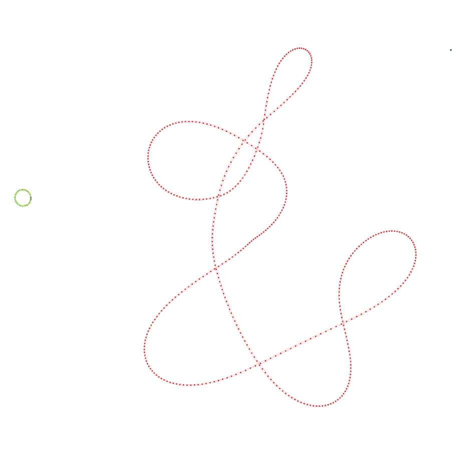	Finished circular assembly	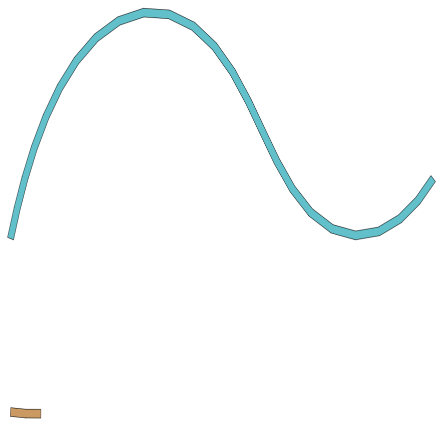
NCTC12652	*Escherichia coli*	63	1	1	2	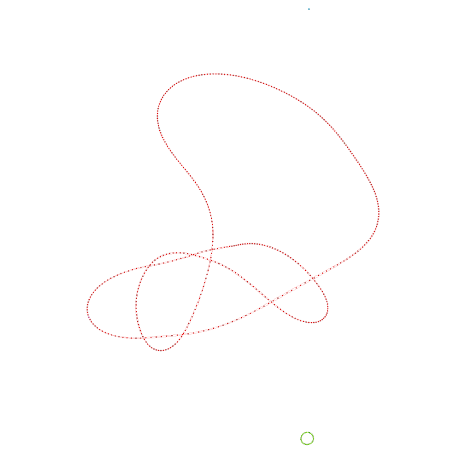	Finished circular assembly	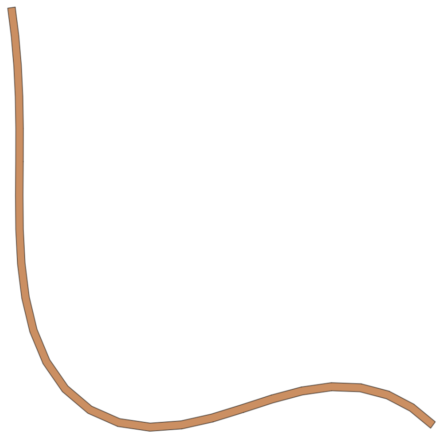
NCTC12653	*Escherichia coli*	71	Pending	Pending	Pending	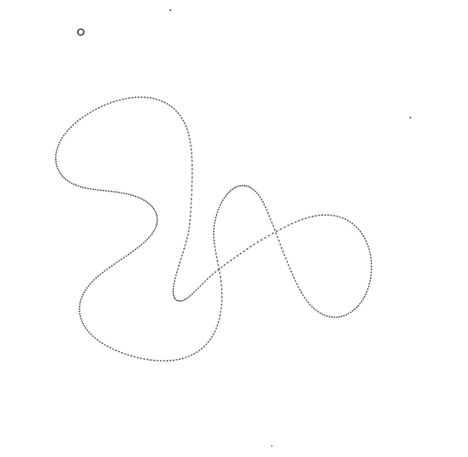	Finished circular assembly	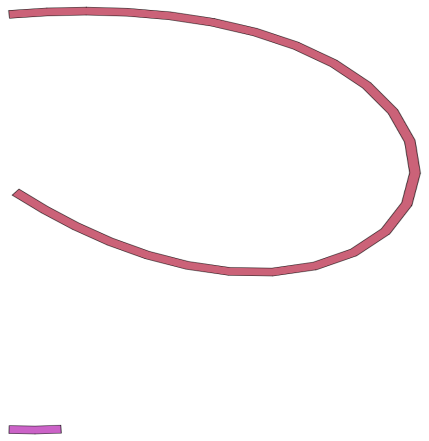
NCTC12654	*Escherichia coli*	50	0	0	5	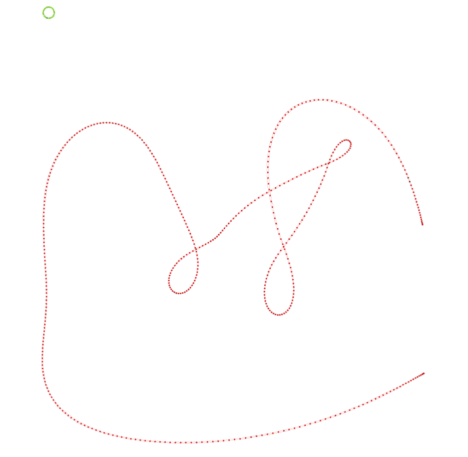	Finished assembly (lacking circularisation)	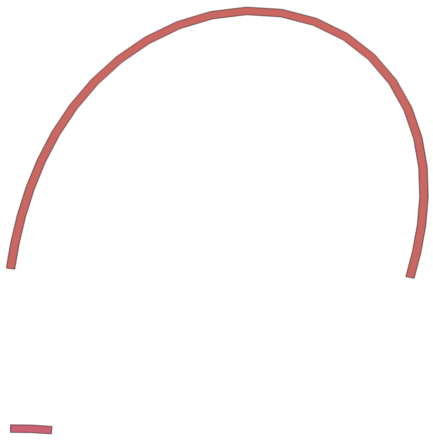
NCTC12655	*Escherichia coli*	49	1	0	0	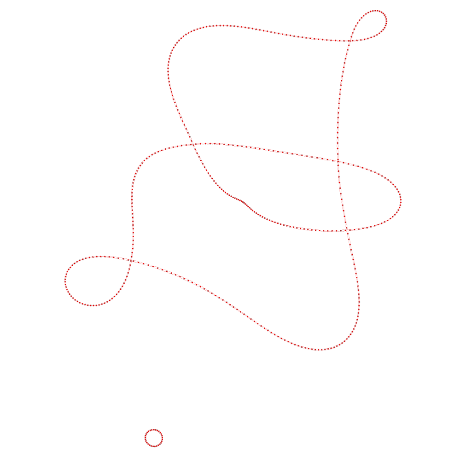	Finished circular assembly	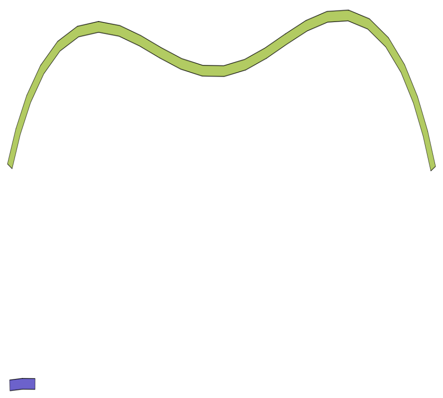
NCTC12694	*Salmonella enterica subsp. enterica*	19	0	0	121		Mis-assembly/Fragmented	

						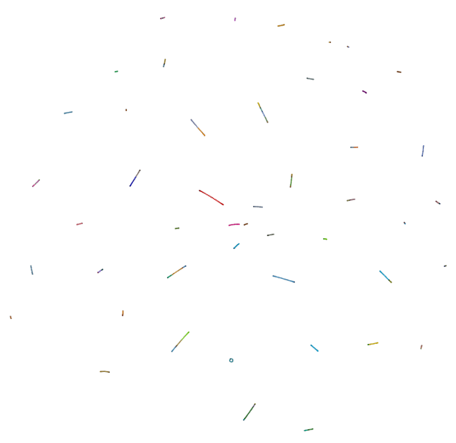		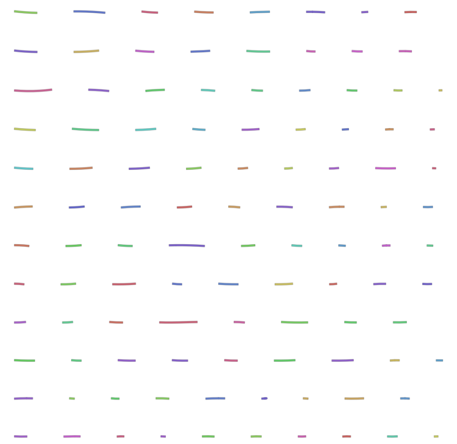
NCTC12696	*Streptococcus pyogenes*	118	Pending	Pending	Pending	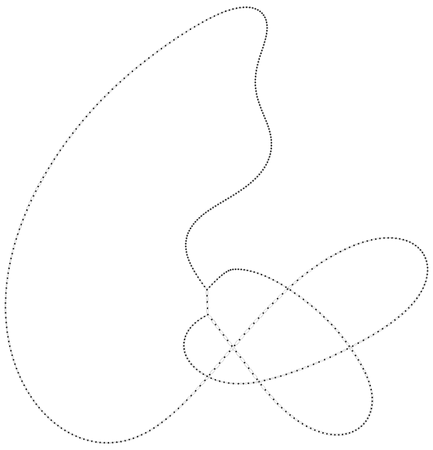	Finished assembly with multiple traversals	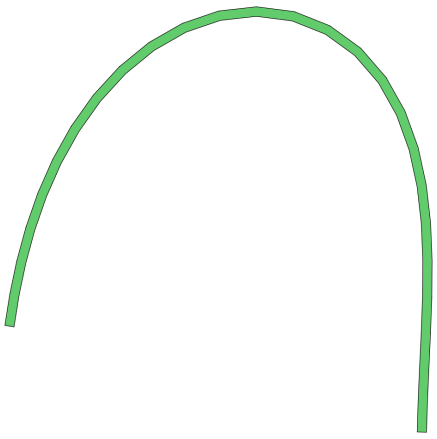
NCTC12699	*Haemophilus influenzae*	94	1	0	0	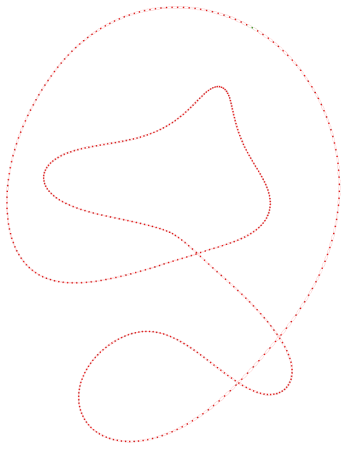	Finished circular assembly	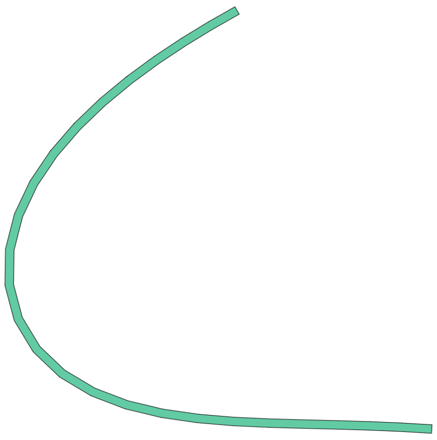
NCTC12700	*Neisseria gonorrhoeae*	172	1	1	1	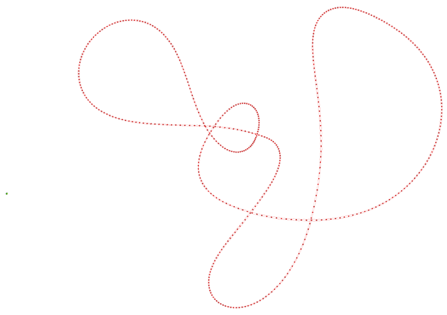	Finished circular assembly	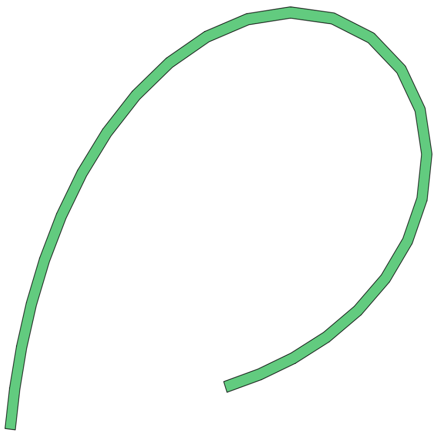
NCTC12709	*Salmonella enterica*	56	1	1	0	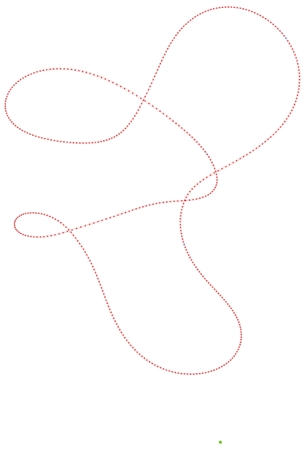	Finished circular assembly	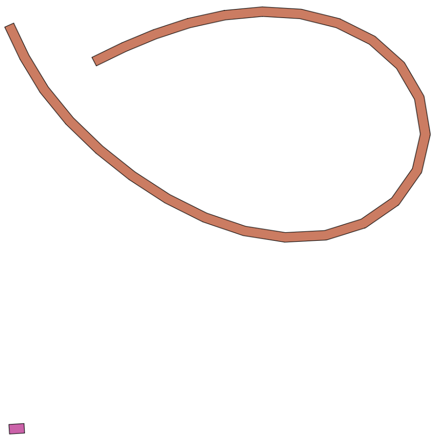
NCTC12737	*Proteus penneri*	88	1	1	0	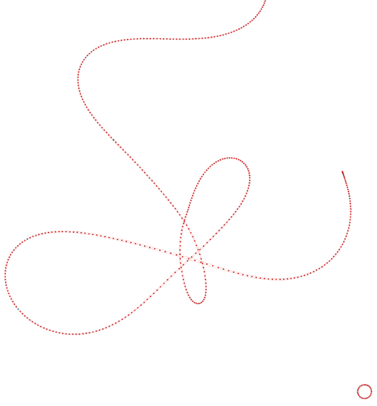	Finished assembly (lacking circularisation)	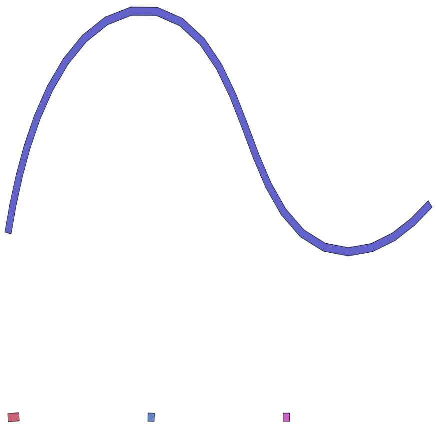
NCTC12839	*Escherichia coli*	50	1	1	0		Finished circular assembly	

						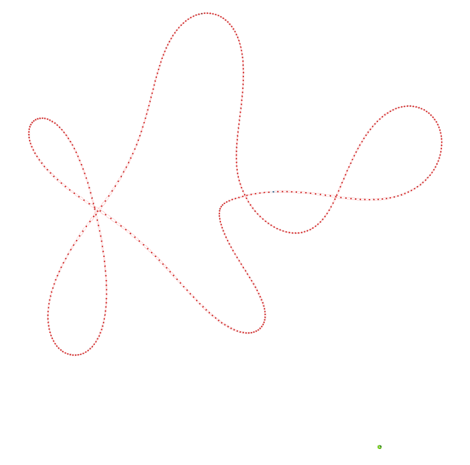		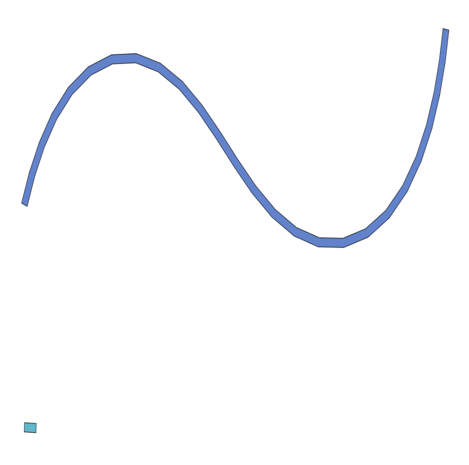
NCTC12840	*Streptococcus pyogenes*	119	1	0	0	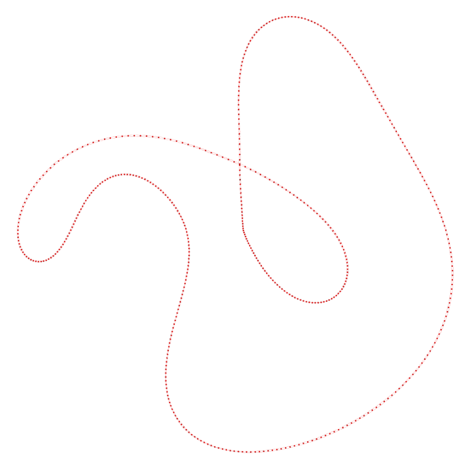	Finished circular assembly	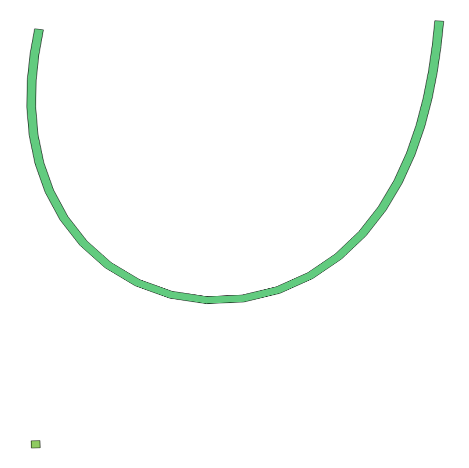
NCTC12841	*Streptococcus pyogenes*	75	Pending	Pending	Pending	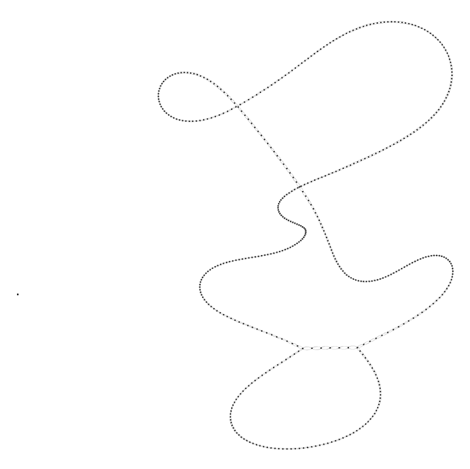	Mis-assembly	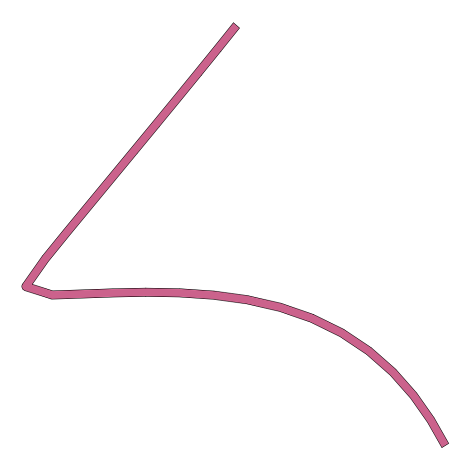
NCTC12854	*Streptococcus parasanguinis*	109	1	0	2	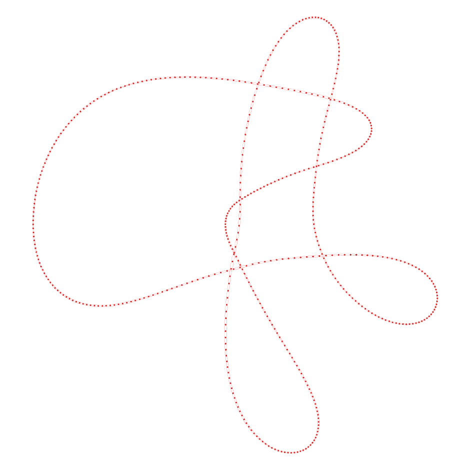	Finished circular assembly	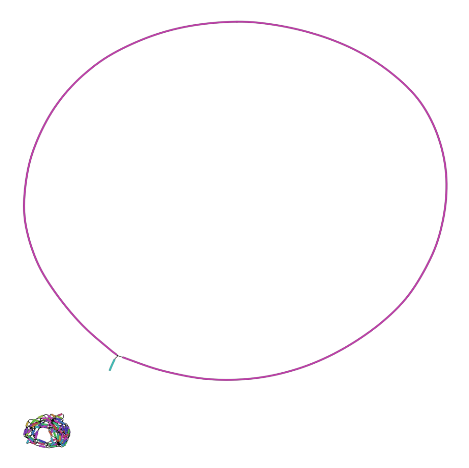
NCTC12874	*Brachyspira pilosicoli*	18	0	0	3	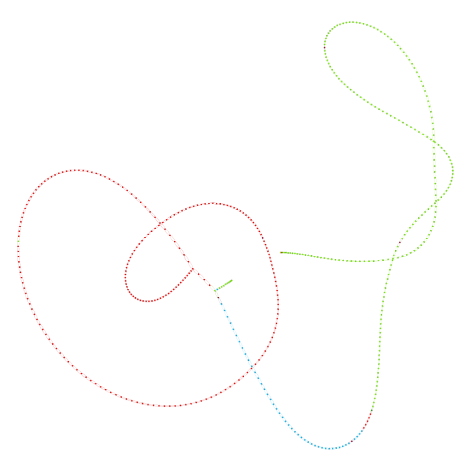	Mis-assembly/Fragmented	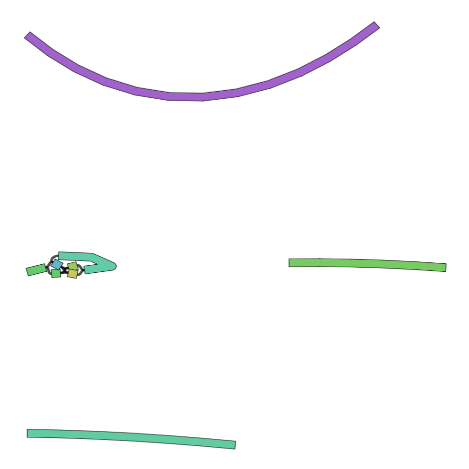
NCTC12880	*Staphylococcus aureus*	139	0	0	2	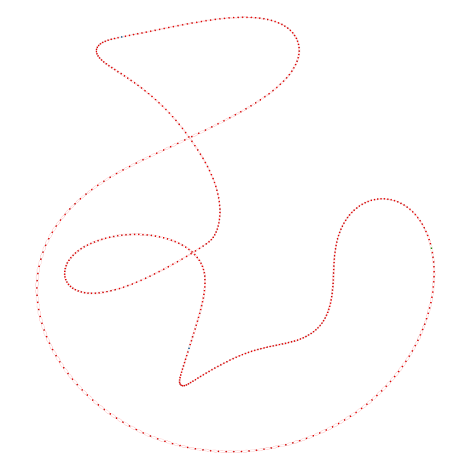	Finished circular assembly	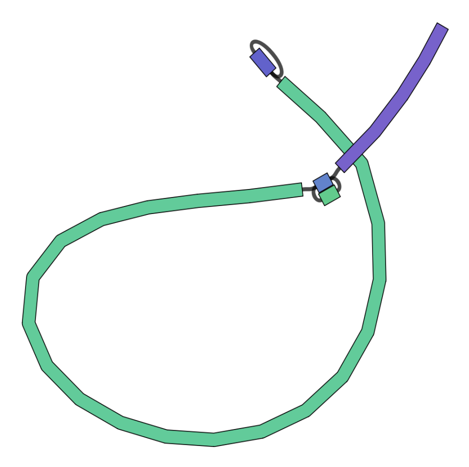
NCTC12899	*Bartonella quintana*	141	Pending	Pending	Pending		Finished assembly (lacking circularisation)	

						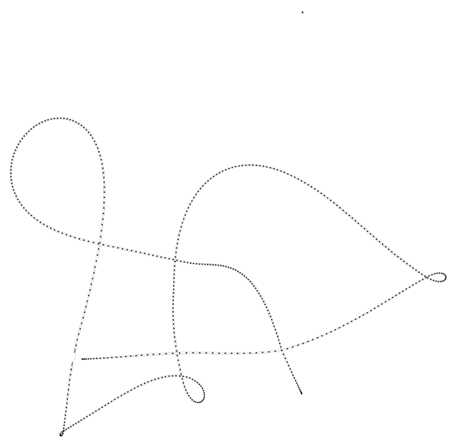		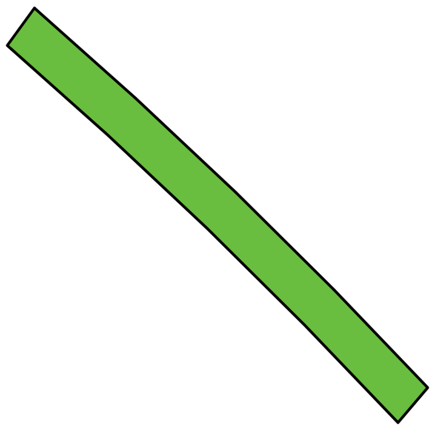
NCTC12924	*Pseudomonas aeruginosa*	34	0	0	1	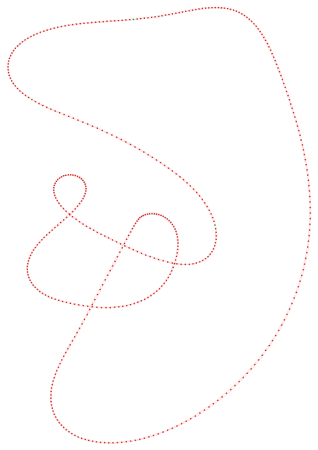	Finished circular assembly	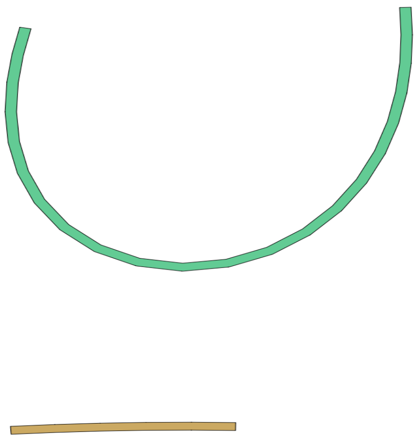
NCTC12950	*Escherichia coli*	35	1	0	1	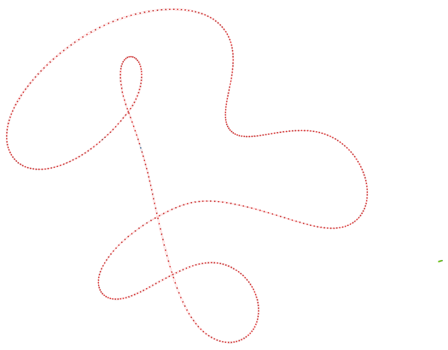	Finished circular assembly	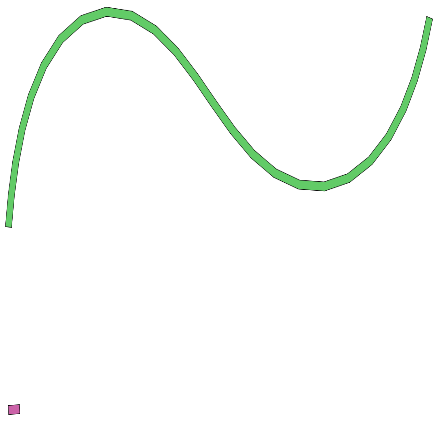
NCTC12951	*Pseudomonas aeruginosa*	36	0	0	28	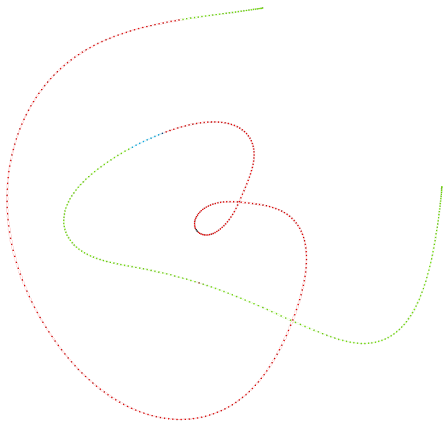	Finished assembly (lacking circularisation)	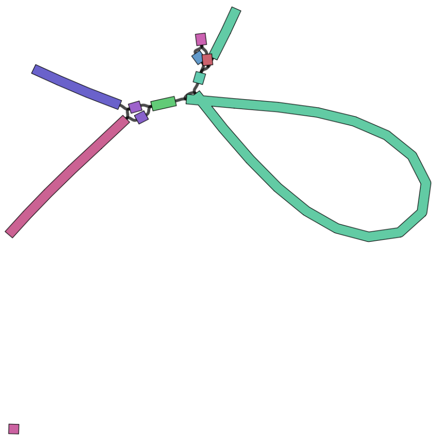
NCTC12957	*Streptococcus acidominimus*	137	0	0	5	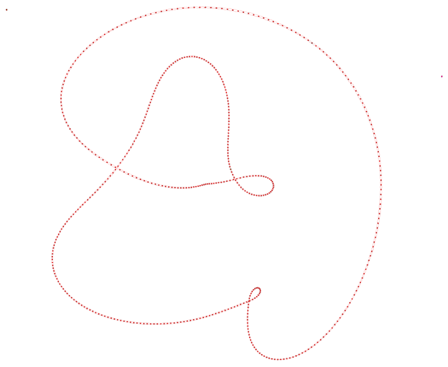	Finished circular assembly	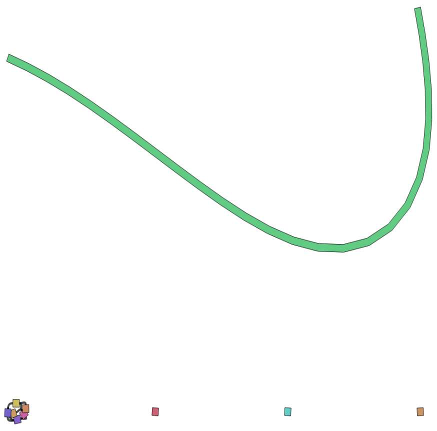
NCTC12958	*Streptococcus thermophilus*	126	1	0	0	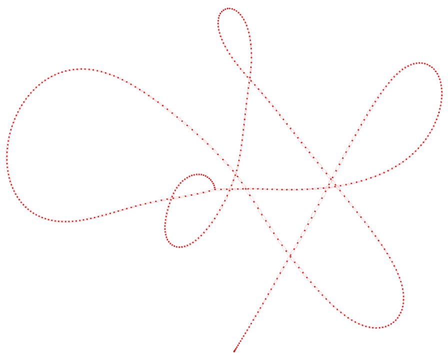	Mis-assembly/Fragmented	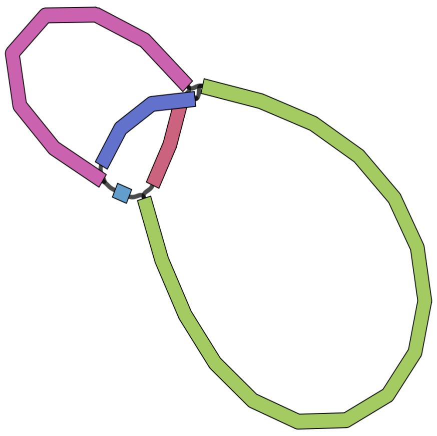
NCTC12961	*Serratia plymuthica*	37	1	0	0		Finished circular assembly	

						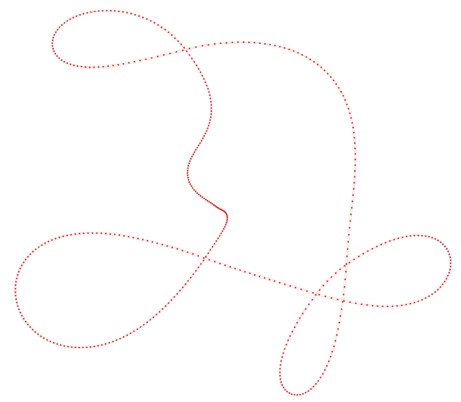		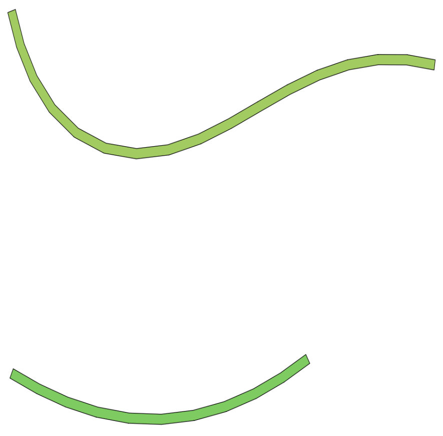
NCTC12965	*Serratia fonticola*	47	1	0	0	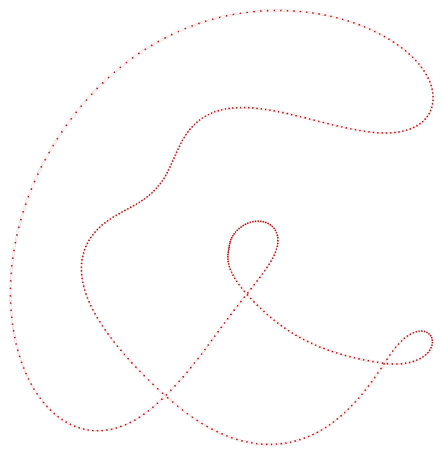	Finished circular assembly	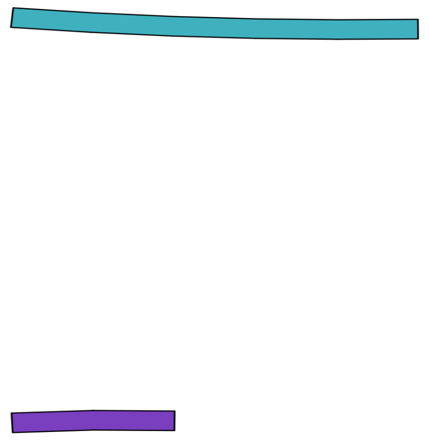
NCTC12969	*Streptococcus equinus*	69	Pending	Pending	Pending	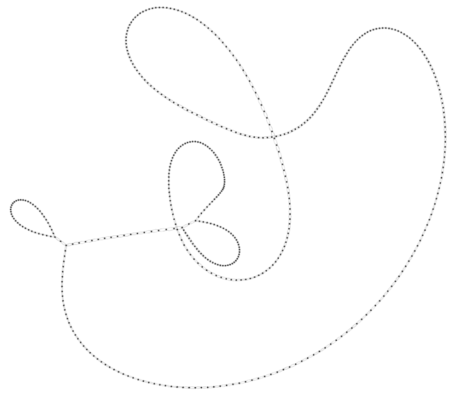	Finished assembly with multiple traversals	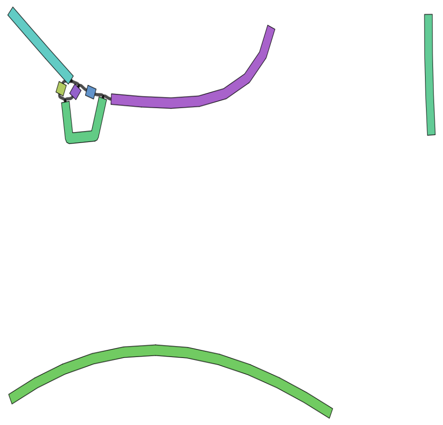
NCTC12971	*Serratia rubidaea*	58	1	0	0	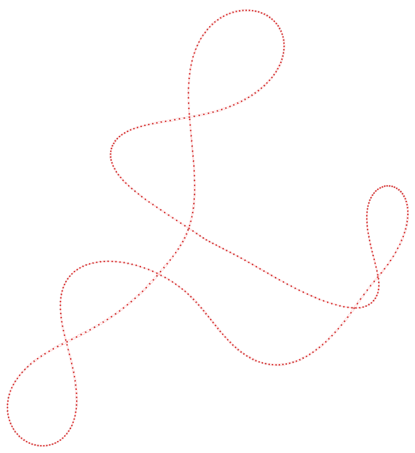	Finished circular assembly	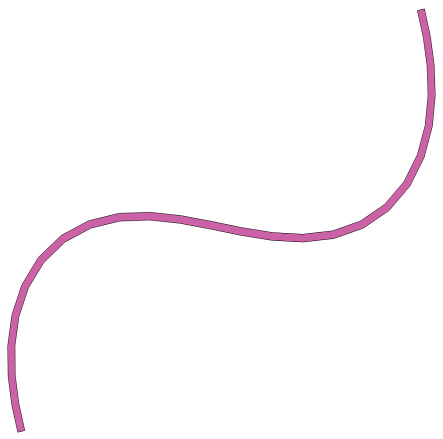
NCTC12973	*Staphylococcus aureus*	192	Pending	Pending	Pending	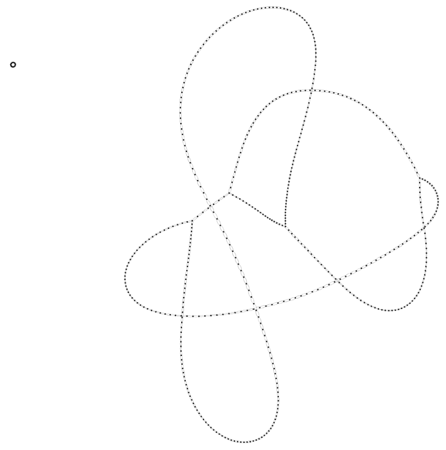	Mis-assembly/Fragmented	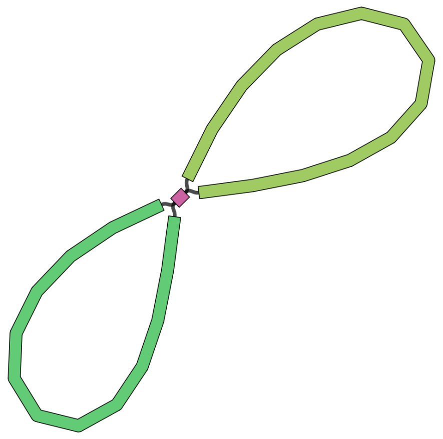
NCTC12981	*Staphylococcus aureus*	86	Pending	Pending	Pending	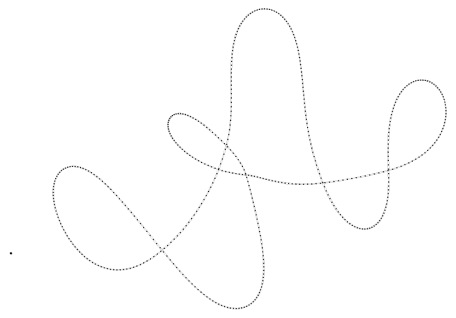	Finished circular assembly	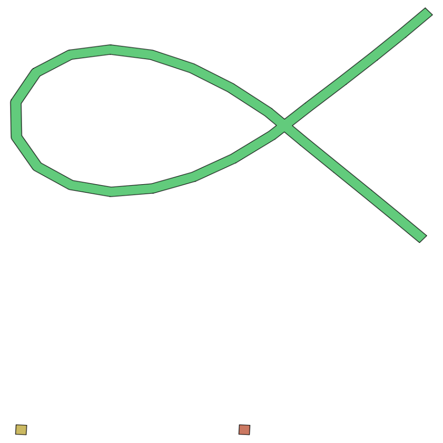
NCTC12982	*Yersinia enterocolitica subsp. enterocolitica*	46	1	0	0		Finished circular assembly	

						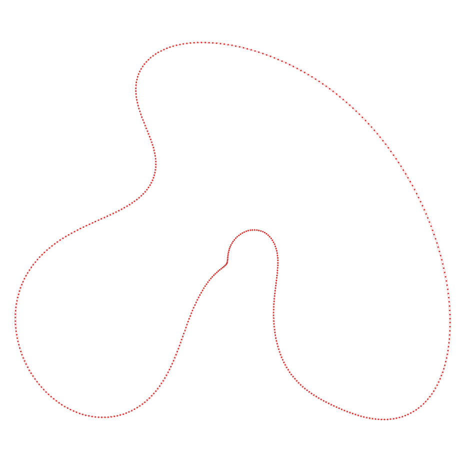		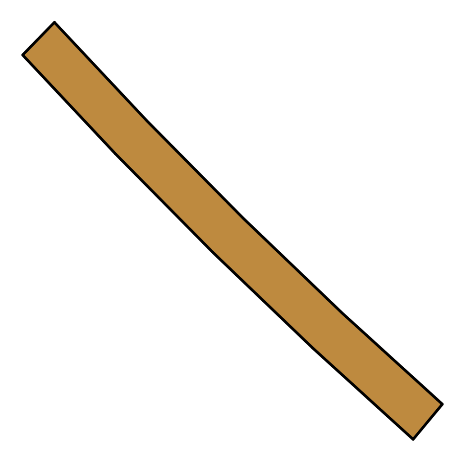
NCTC12986	*Yersinia ruckeri*	44	1	1	1	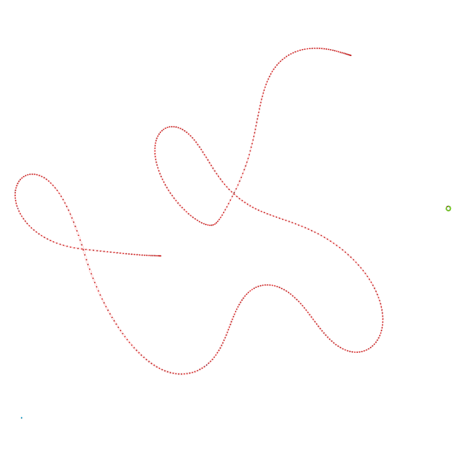	Finished assembly (lacking circularisation)	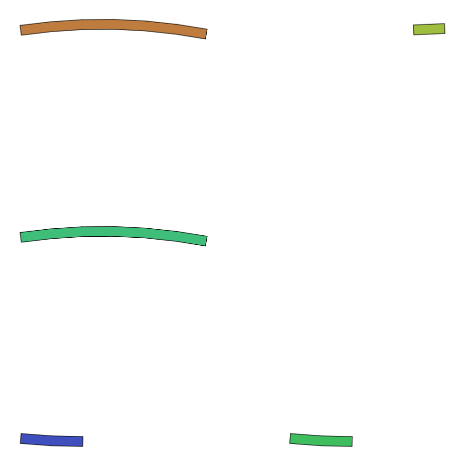
NCTC12993	*Kluyvera cryocrescens*	46	5	1	0	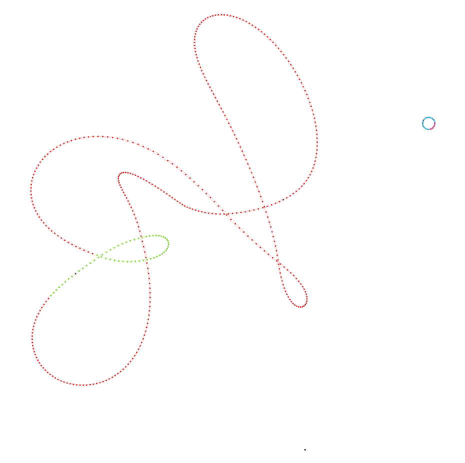	Finished circular assembly	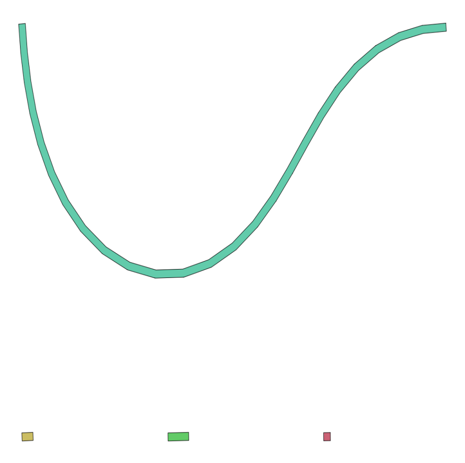
NCTC12998	*Raoultella planticola*	41	1	1	0	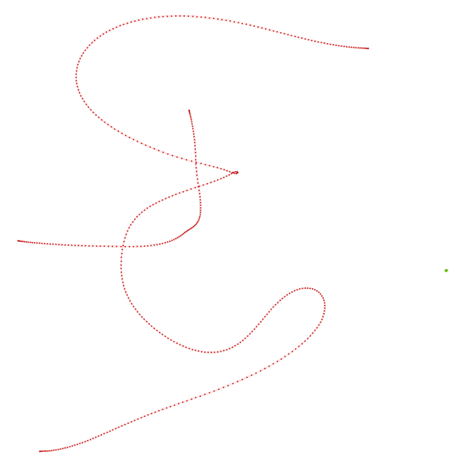	Mis-assembly/Fragmented	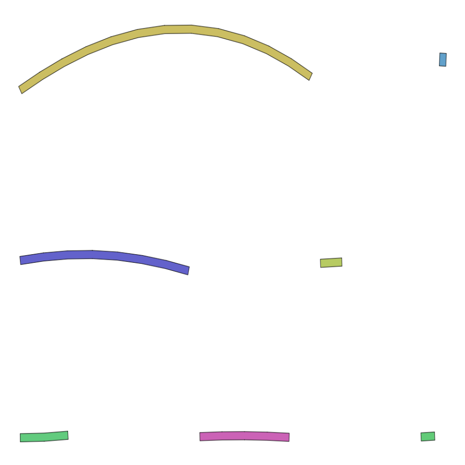
NCTC13032	*Leclercia adecarboxylata*	57	1	0	0	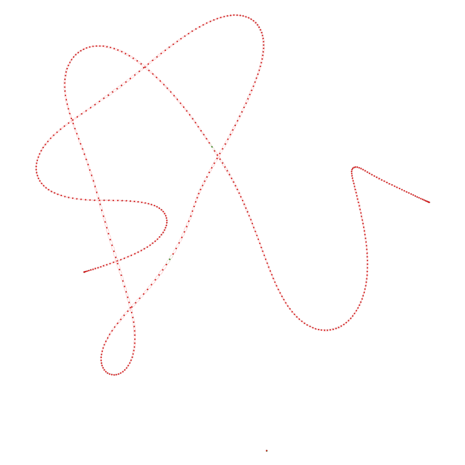	Finished circular assembly	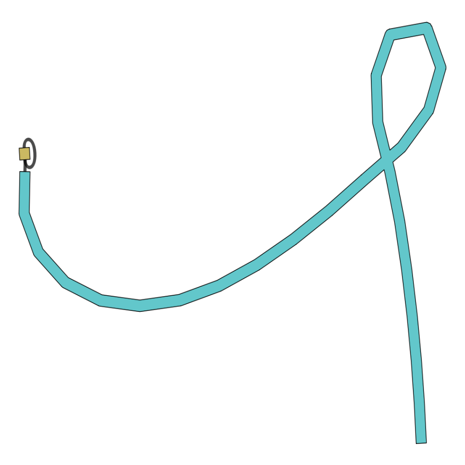
NCTC13038	*Raoultella terrigena*	70	1	0	0	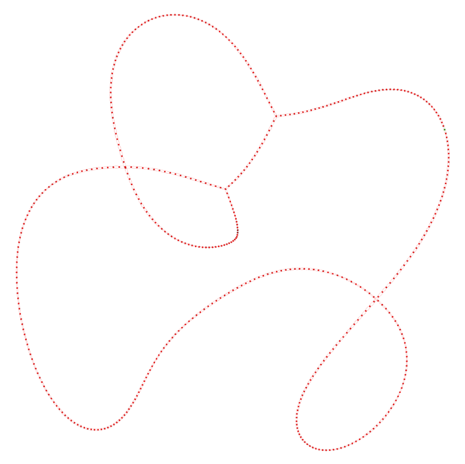	Mis-assembly/Fragmented	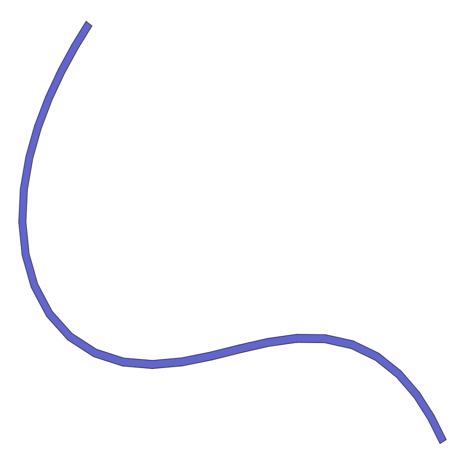
NCTC13046	*Brachyspira pilosicoli*	48	1	0	1		Finished circular assembly	

						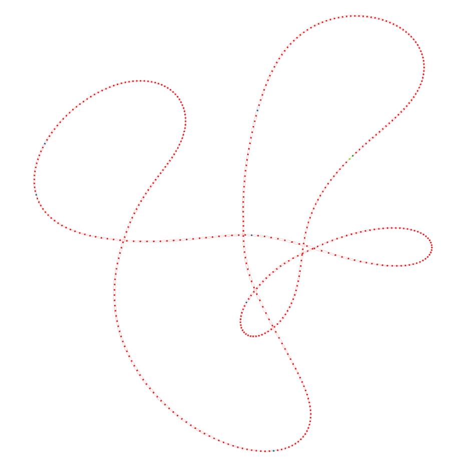		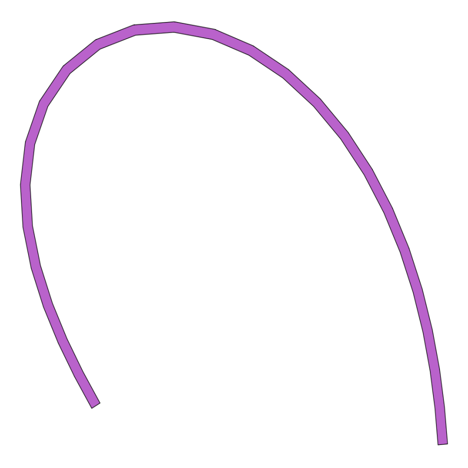
NCTC13095	*Klebsiella planticola*	38	1	1	3	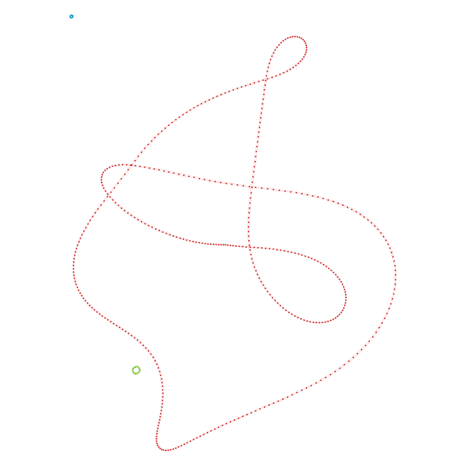	Finished circular assembly	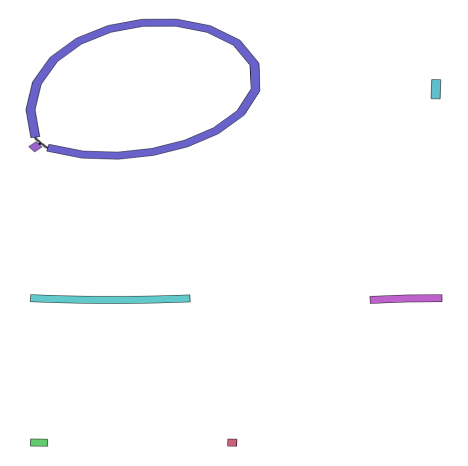
NCTC13096	*Klebsiella planticola*	53	1	0	0	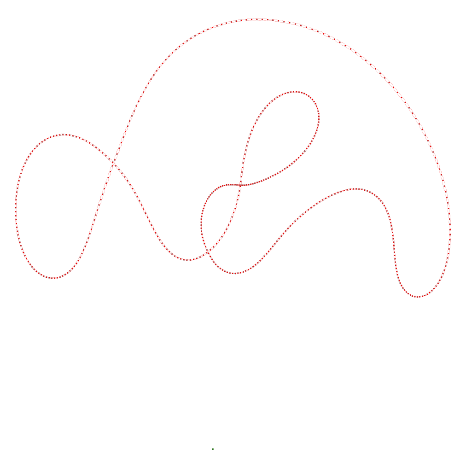	Finished circular assembly	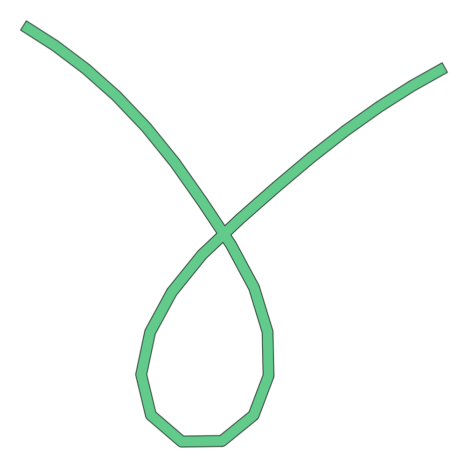
NCTC13097	*Raoultella terrigena*	73	1	1	0	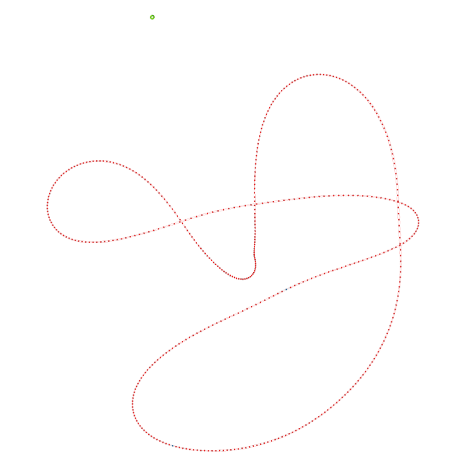	Finished circular assembly	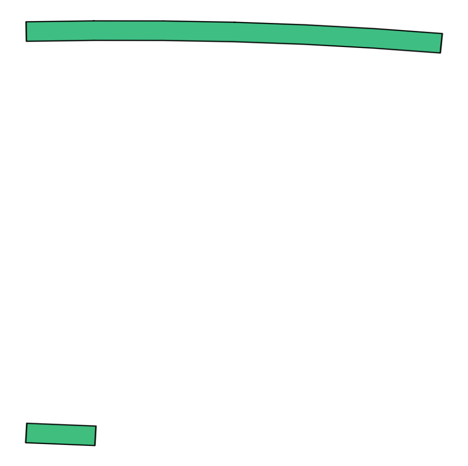
NCTC13098	*Raoultella terrigena*	24	1	0	0	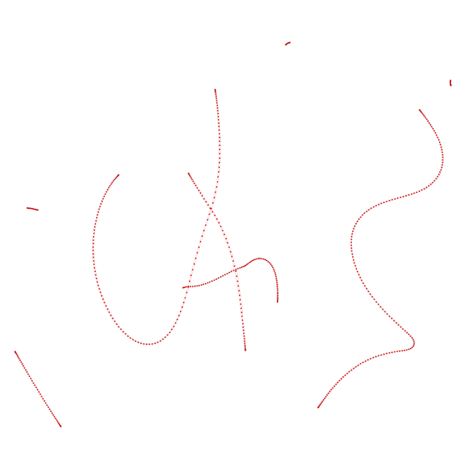	Mis-assembly/Fragmented	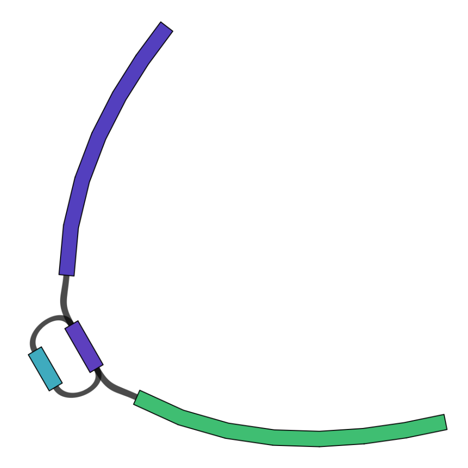
NCTC13125	*Escherichia coli*	49	1	2	4	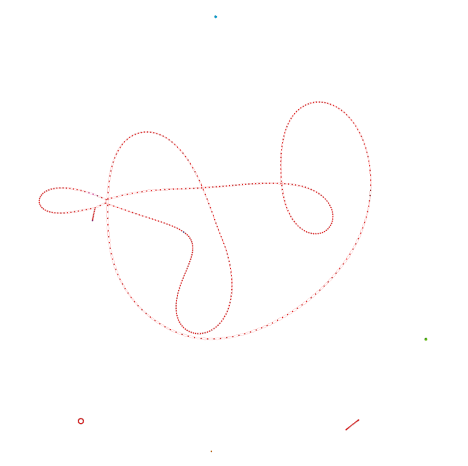	Mis-assembly/Fragmented	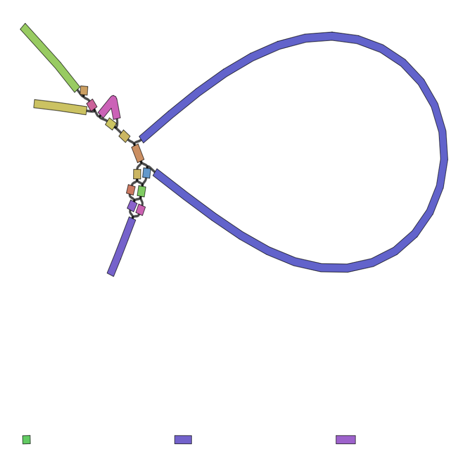
NCTC13127	*Escherichia coli*	38	1	0	3		Finished circular assembly	

						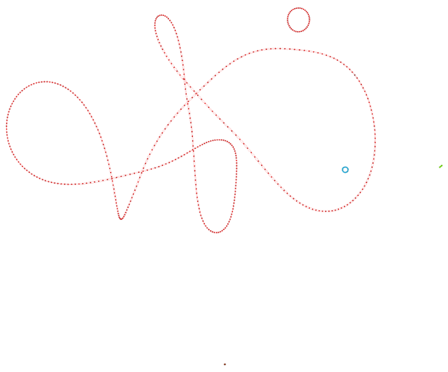		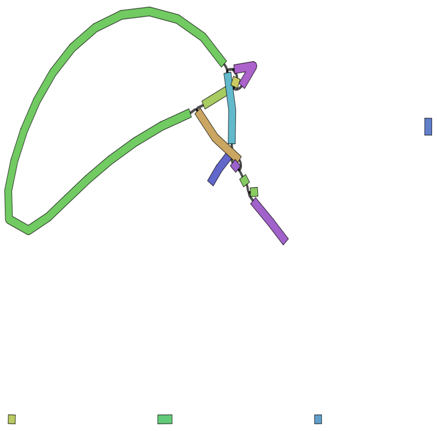
NCTC13128	*Escherichia coli*	82	0	0	3	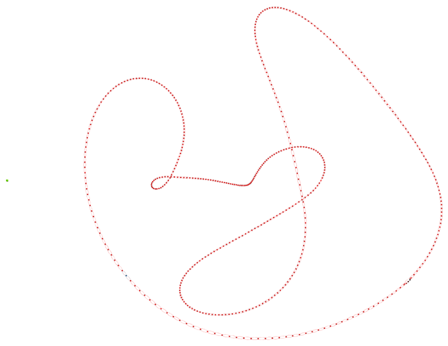	Finished circular assembly	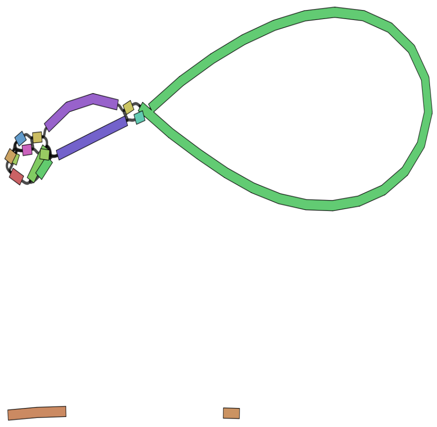
NCTC13131	*Staphylococcus aureus*	55	Pending	Pending	Pending	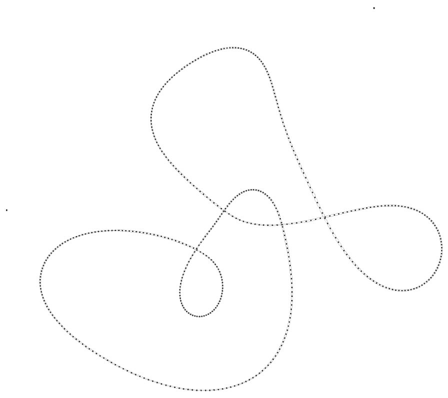	Finished circular assembly	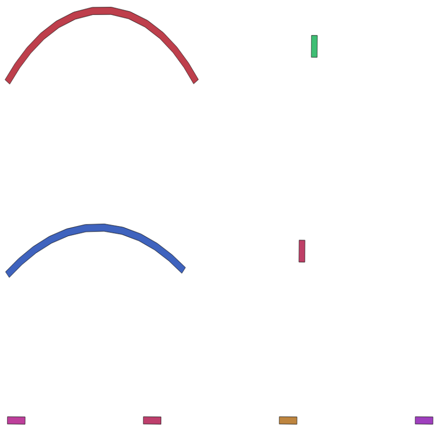
NCTC13132	*Staphylococcus aureus*	142	1	1	2	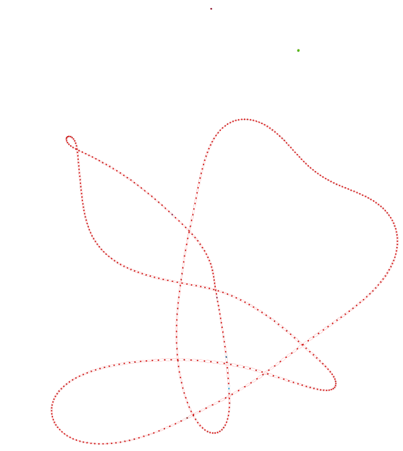	Finished circular assembly	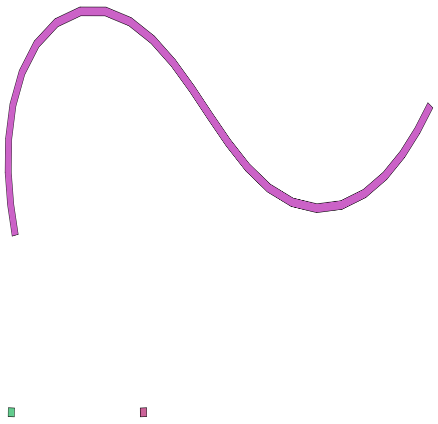
NCTC13133	*Staphylococcus aureus*	82	1	1	0	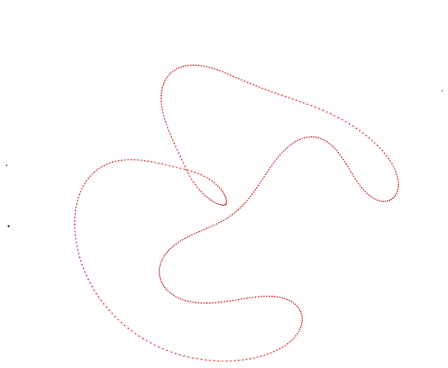	Finished circular assembly	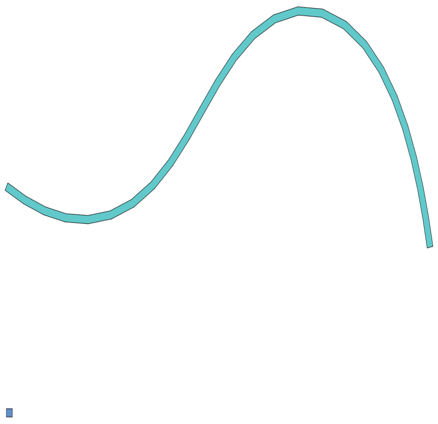
NCTC13135	*Staphylococcus aureus*	141	1	0	2	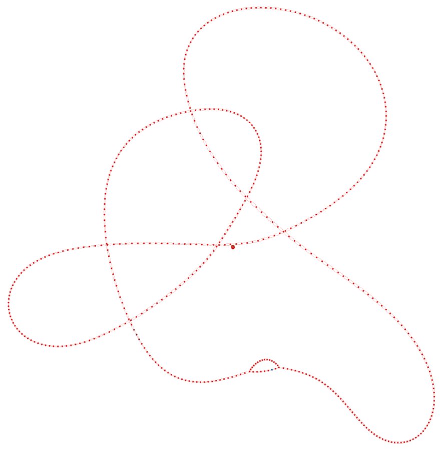	Finished circular assembly	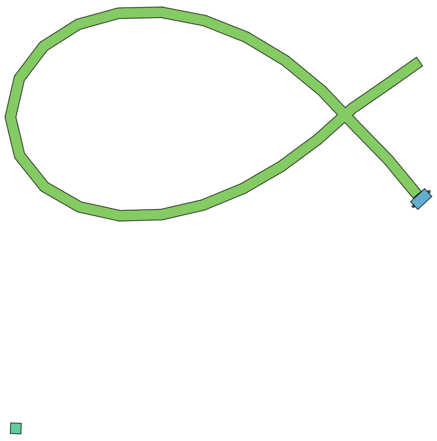
NCTC13136	*Staphylococcus aureus*	116	1	1	0		Finished circular assembly	

						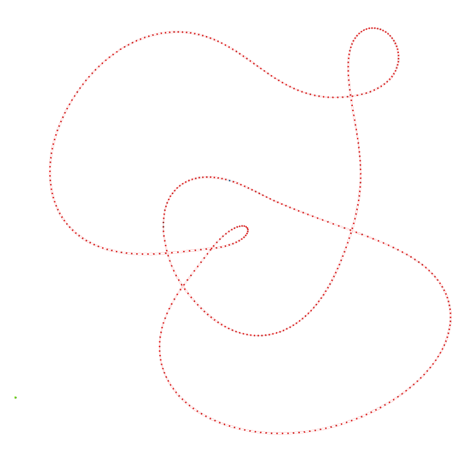		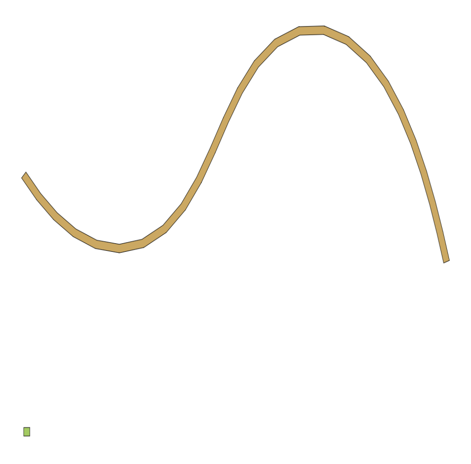
NCTC13137	*Staphylococcus aureus*	152	1	0	0	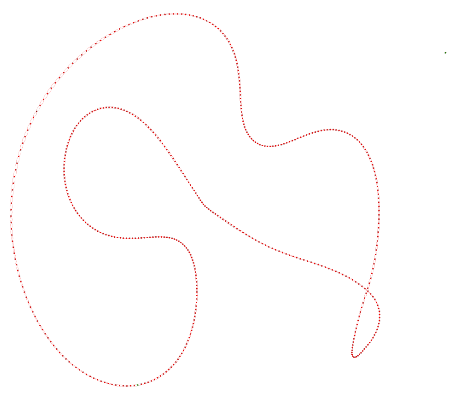	Finished circular assembly	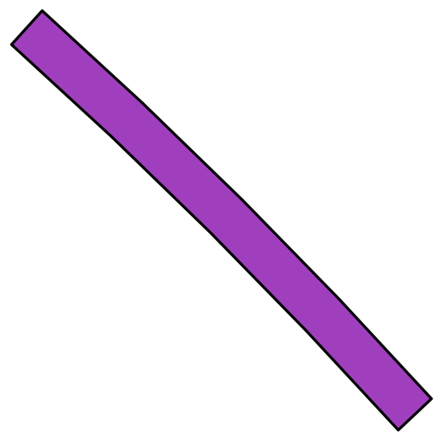
NCTC13138	*Staphylococcus aureus*	160	1	1	0	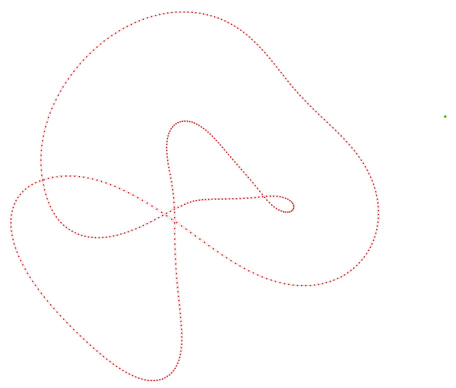	Finished circular assembly	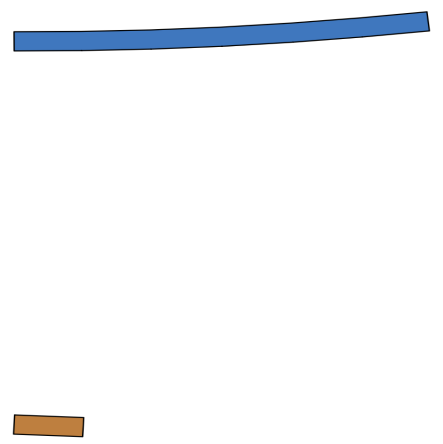
NCTC13139	*Staphylococcus aureus*	150	1	1	2	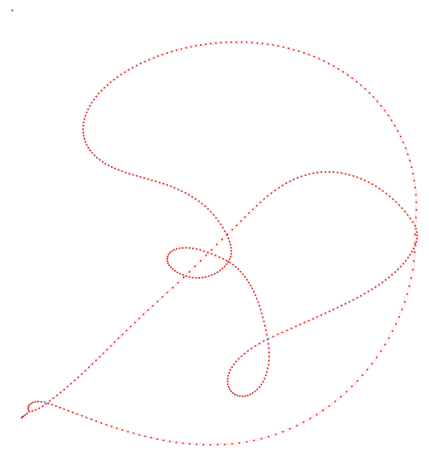	Finished circular assembly	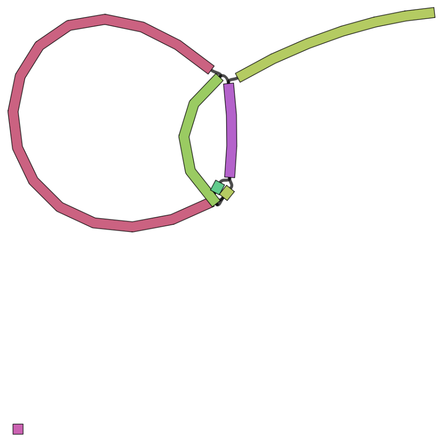
NCTC13140	*Staphylococcus aureus*	141	1	0	0	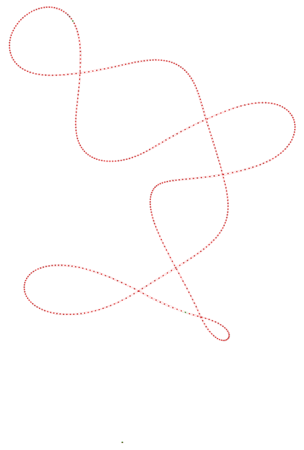	Finished circular assembly	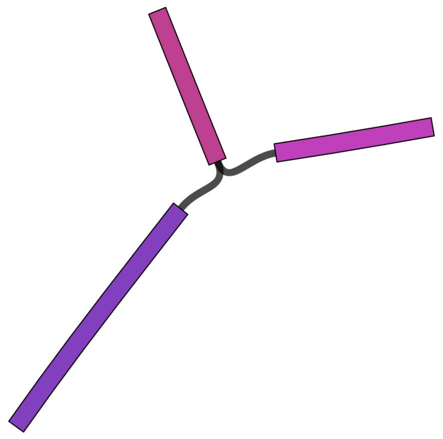
NCTC13141	*Staphylococcus aureus*	92	0	0	4	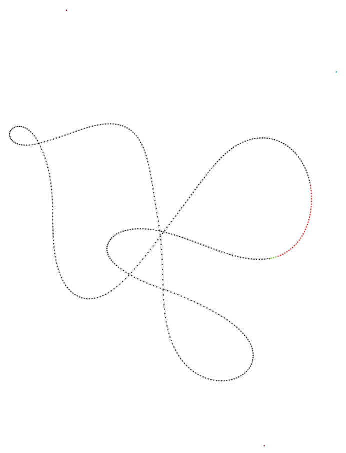	Finished circular assembly	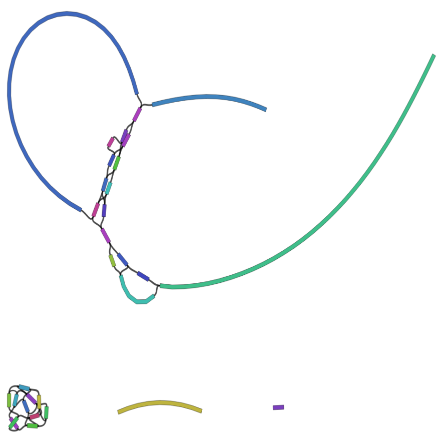
NCTC13142	*Staphylococcus aureus*	77	1	0	0		Finished circular assembly	

						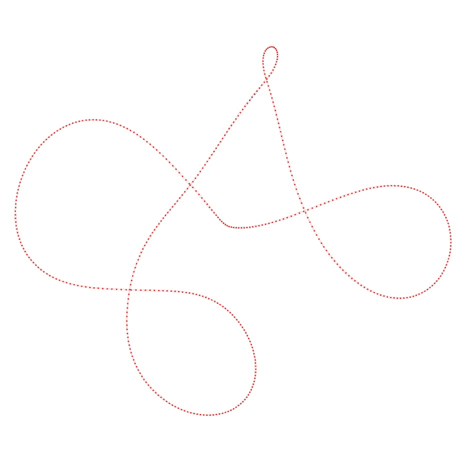		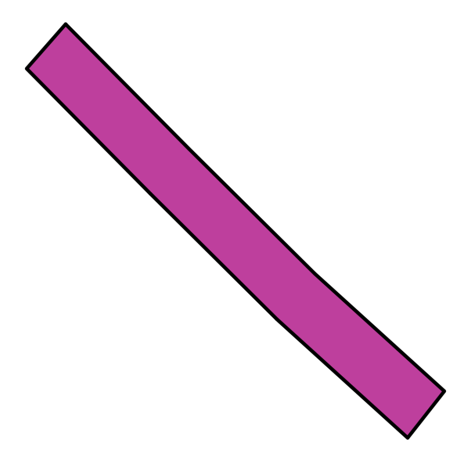
NCTC13143	*Staphylococcus aureus*	45	0	0	3	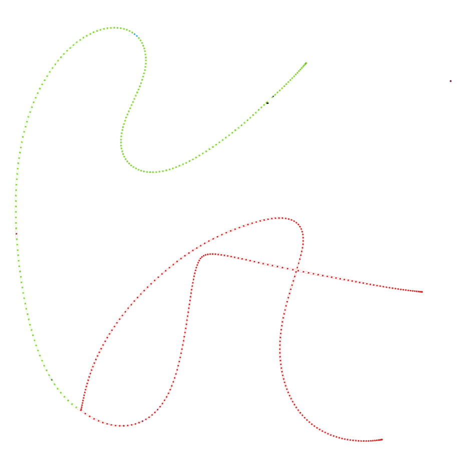	Mis-assembly/Fragmented	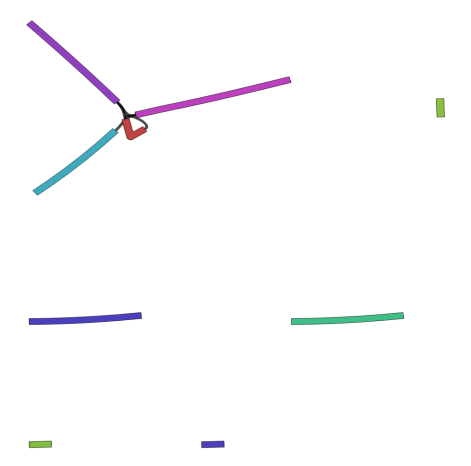
NCTC13145	*Proteus vulgaris*	12	1	0	0	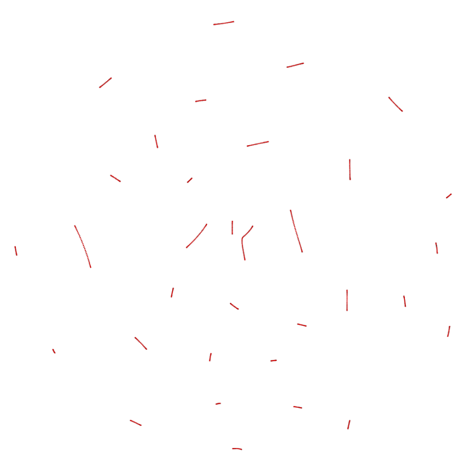	Mis-assembly/Fragmented	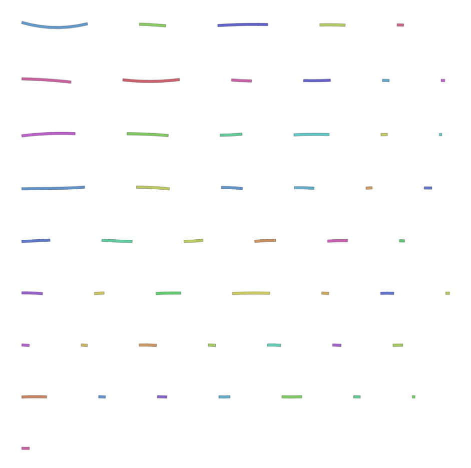
NCTC13148	*Escherichia coli*	24	0	0	6	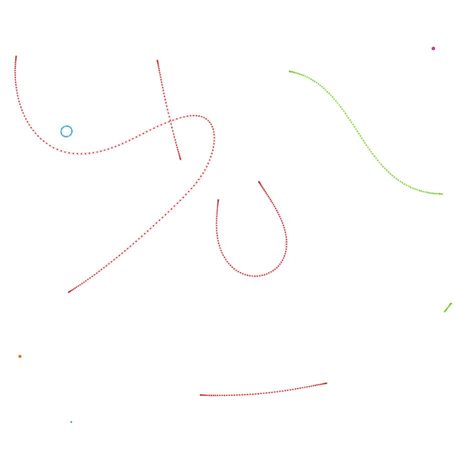	Mis-assembly/Fragmented	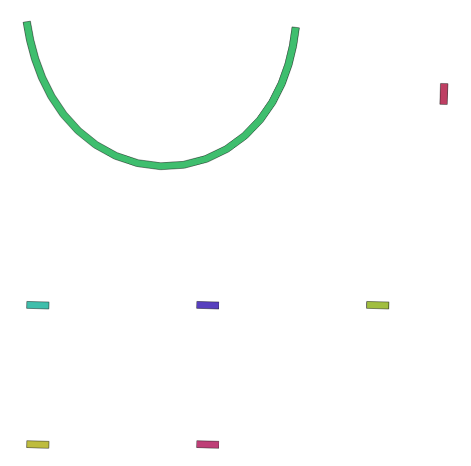
NCTC13169	*Enterococcus faecium*	139	1	1	0	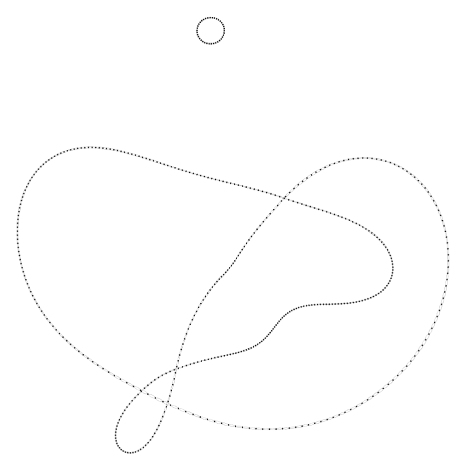	Finished circular assembly	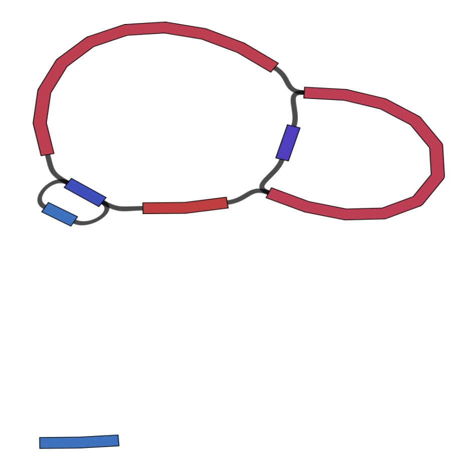
NCTC13175	*Salmonella enterica subsp. enterica serovar Goldcoast*	103	1	0	0	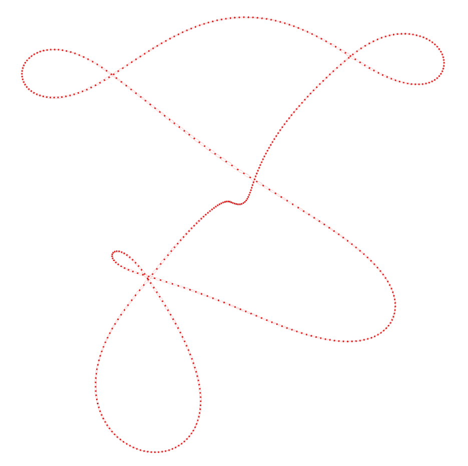	Finished circular assembly	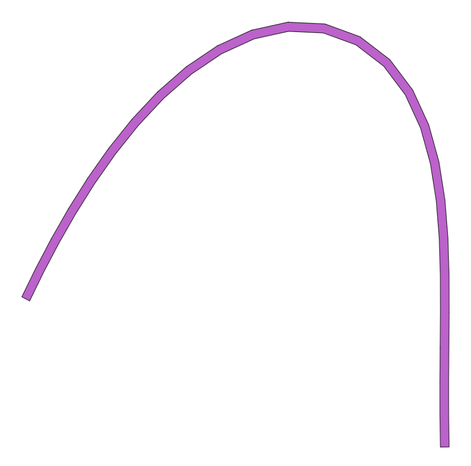
NCTC13196	*Staphylococcus aureus*	92	0	0	17		Mis-assembly/Fragmented	

						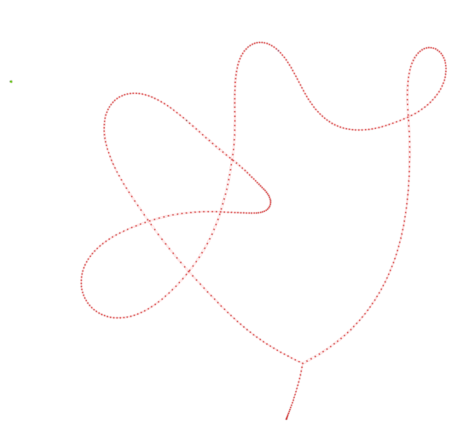		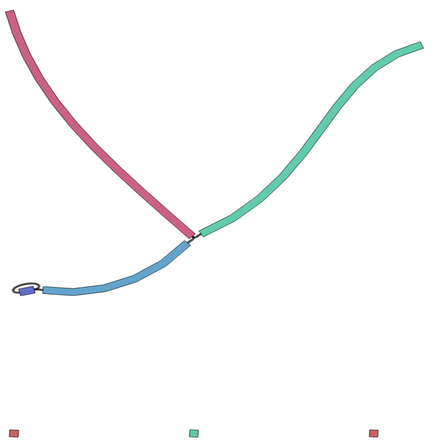
NCTC13216	*Escherichia coli*	204	0	0	3	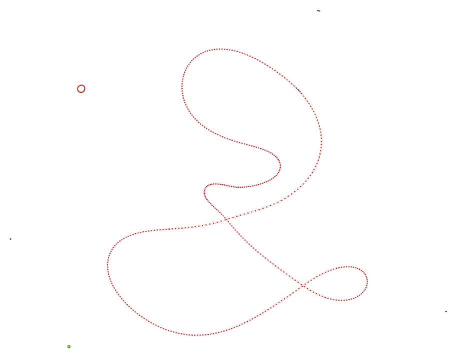	Finished circular assembly	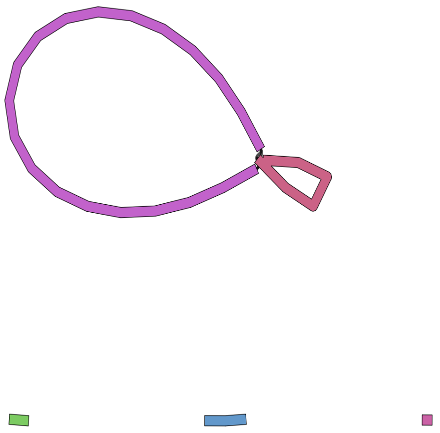
NCTC13251	*Bordetella pertussis*	65	1	0	0	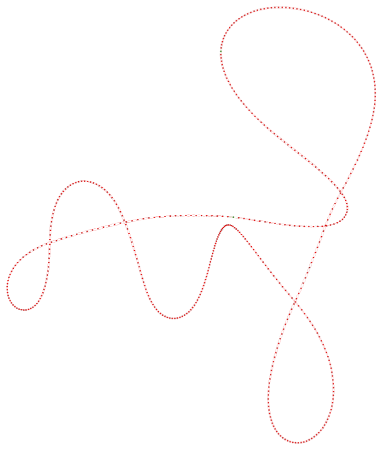	Finished circular assembly	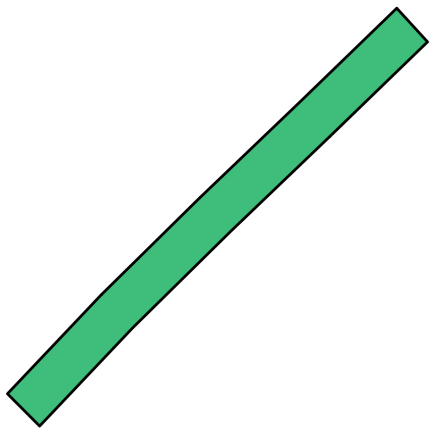
NCTC13276	*Streptococcus pneumoniae*	19	Pending	Pending	Pending	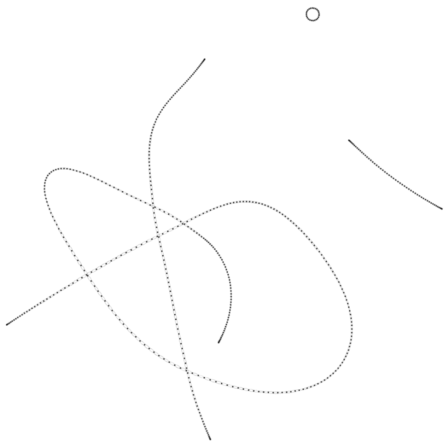	Mis-assembly/Fragmented	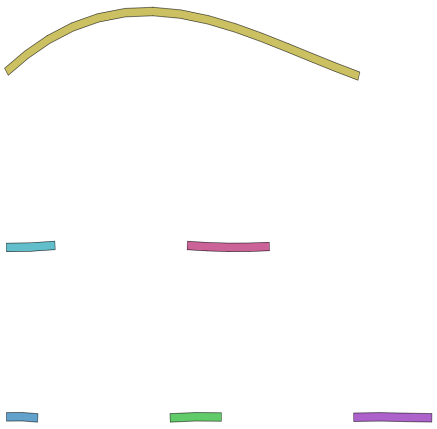
NCTC13277	*Staphylococcus aureus MRSA 252*	51	1	0	0	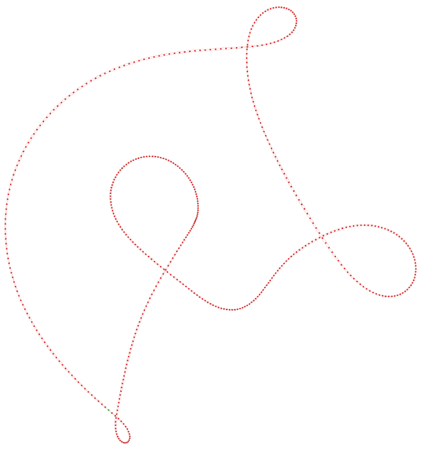	Finished circular assembly	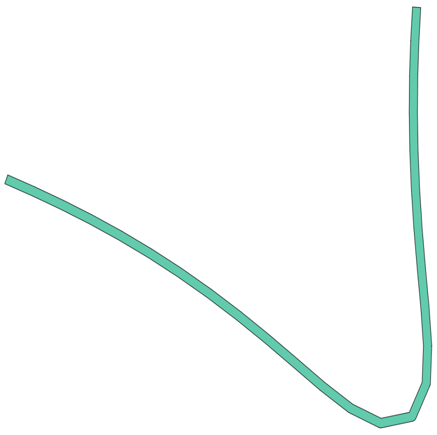
NCTC13297	*Staphylococcus aureus*	129	1	1	0	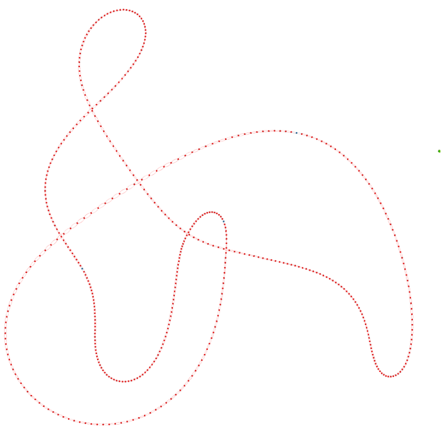	Finished circular assembly	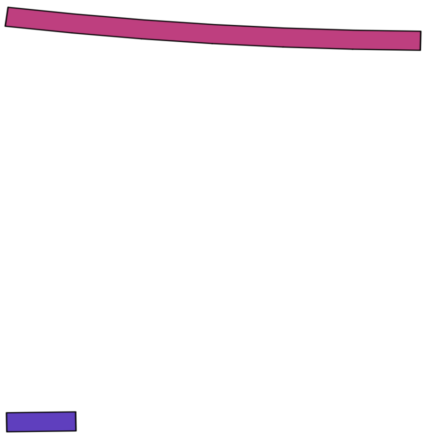
NCTC13298	*Staphylococcus aureus*	122	1	0	3		Finished circular assembly	

						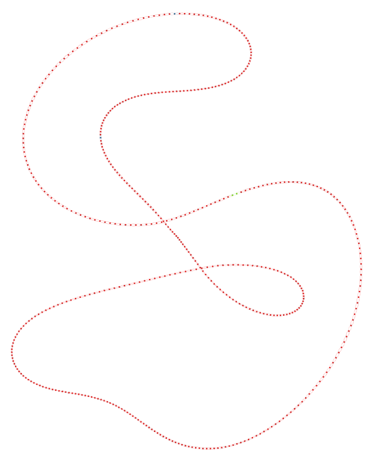		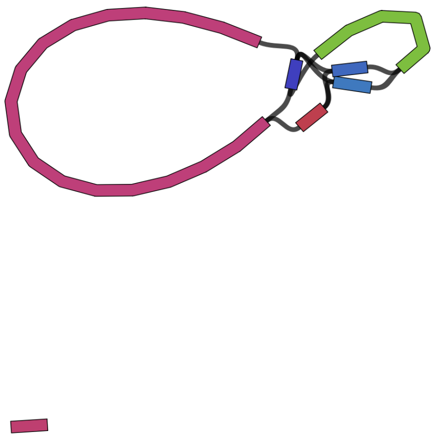
NCTC13299	*Staphylococcus aureus*	121	1	0	1	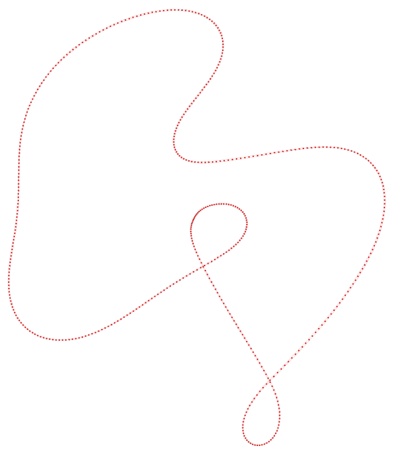	Finished circular assembly	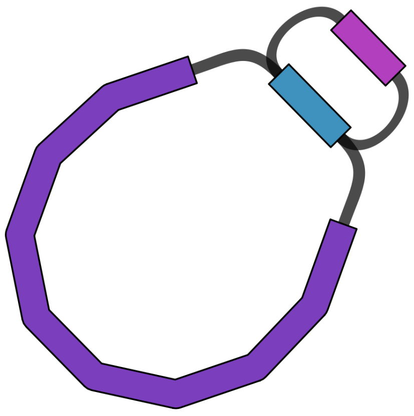
NCTC13300	*Staphylococcus aureus*	136	1	1	0	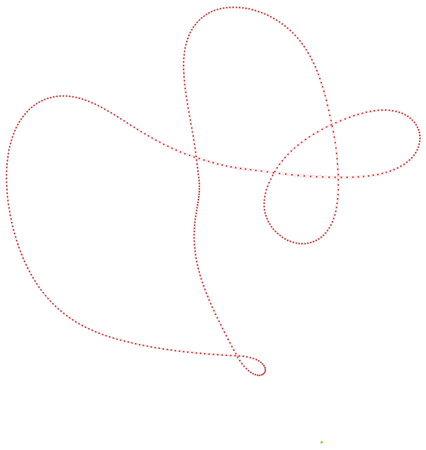	Finished circular assembly	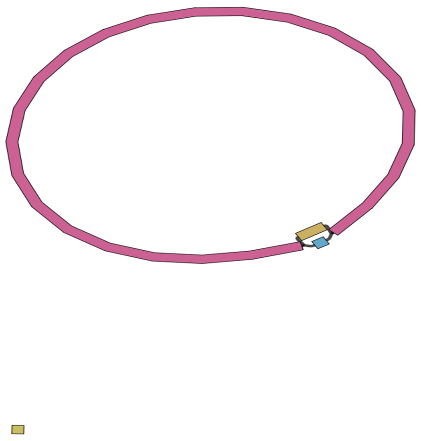
NCTC13301	*Acinetobacter baumannii*	80	0	0	3	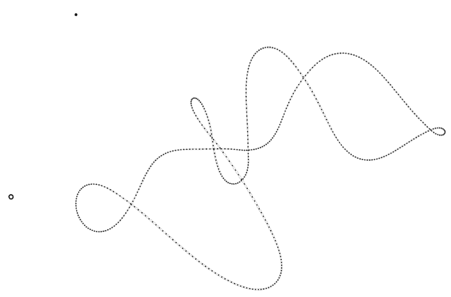	Finished circular assembly	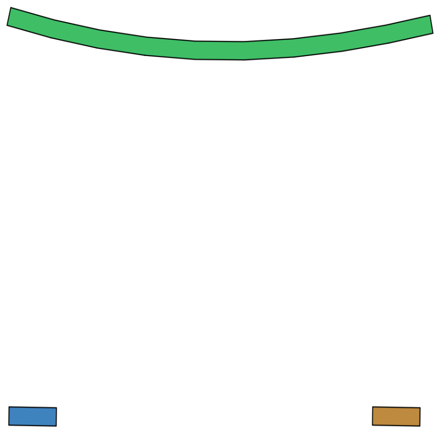
NCTC13305	*Acinetobacter baumannii*	101	0	0	7	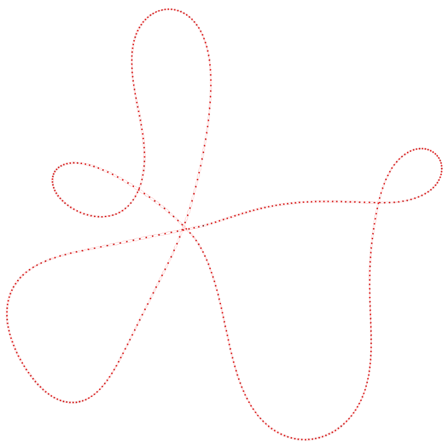	Finished circular assembly	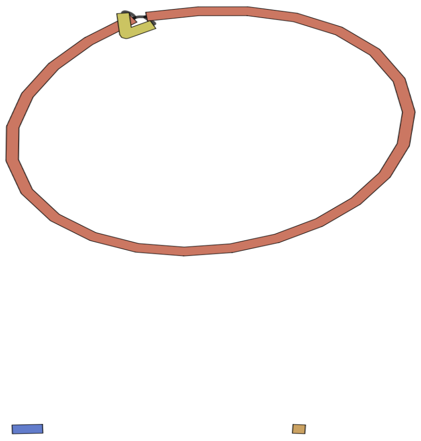
NCTC13307	*Peptoclostridium difficile*	45	4	0	0	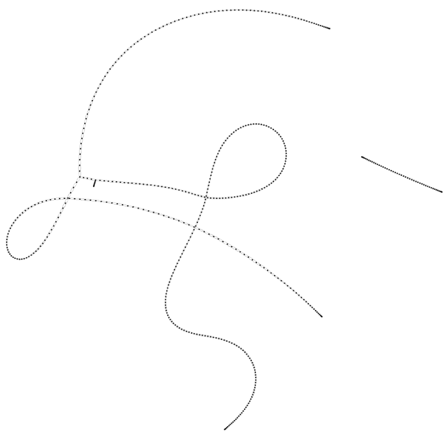	Mis-assembly/Fragmented	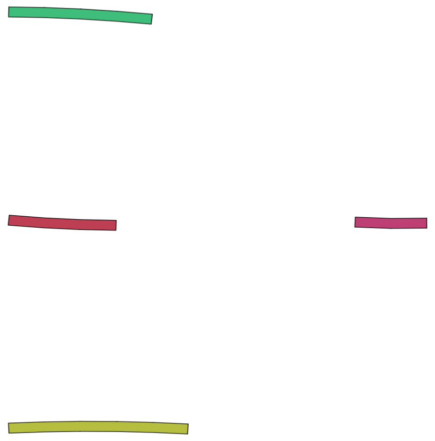
NCTC13347	*Salmonella enterica subsp. enterica serovar Typhimurium*	60	1	2	1		Finished circular assembly	

						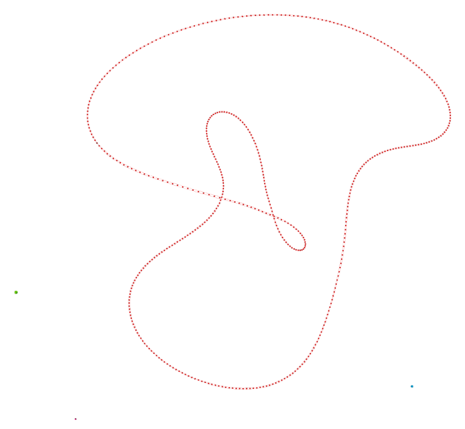		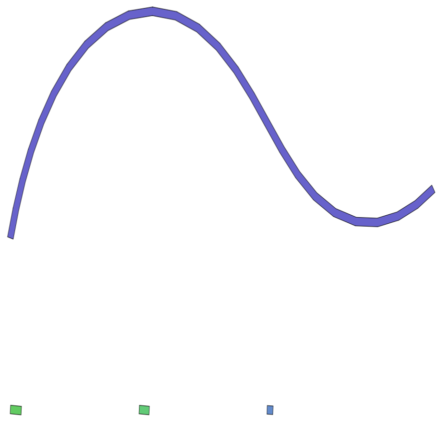
NCTC13348	*Salmonella enterica subsp. enterica serovar Typhimurium*	41	4	2	0	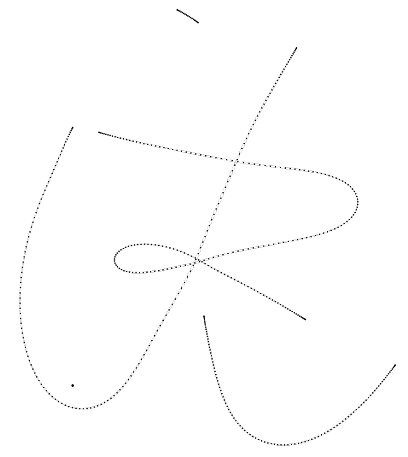	Mis-assembly/Fragmented	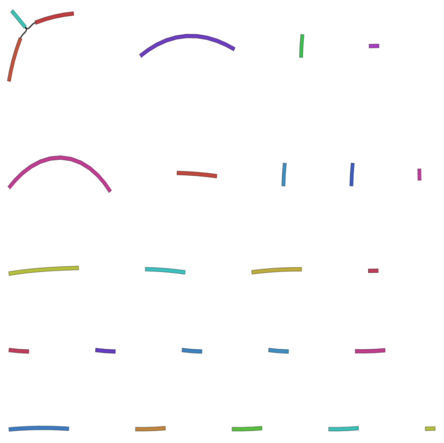
NCTC13349	*Salmonella enteritidis*	44	1	1	0	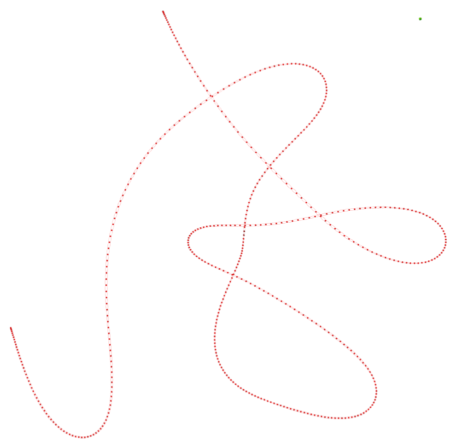	Finished circular assembly	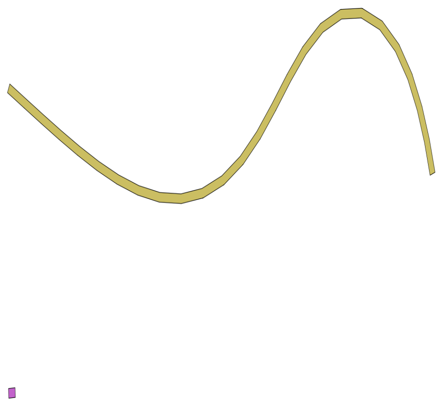
NCTC13351	*Escherichia coli*	58	1	1	0	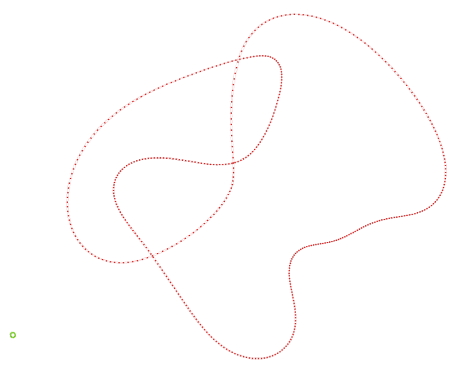	Finished circular assembly	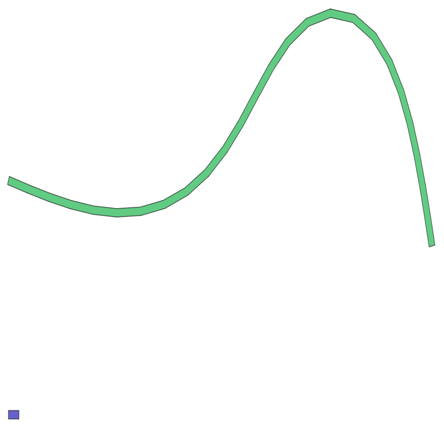
NCTC13352	*Escherichia coli*	31	1	1	2	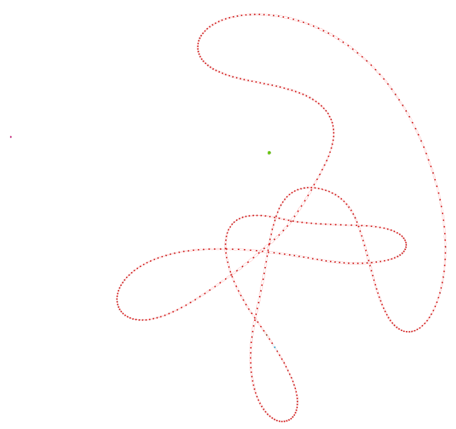	Finished circular assembly	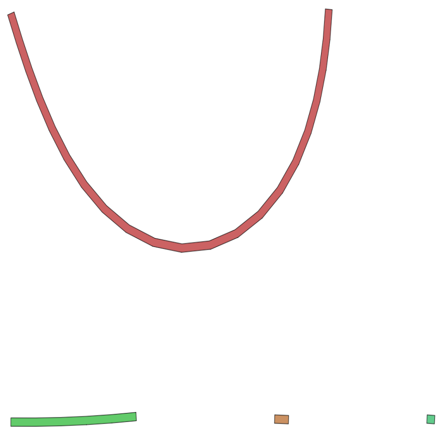
NCTC13353	*Escherichia coli*	40	0	0	12	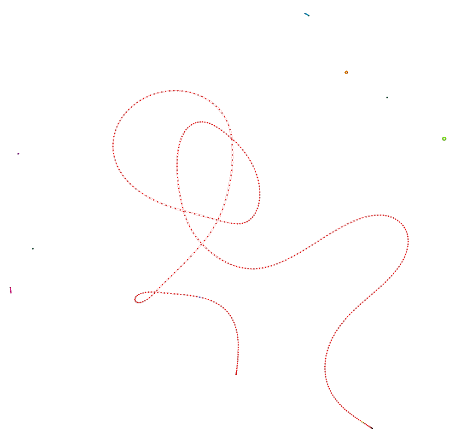	Finished assembly (lacking circularisation)	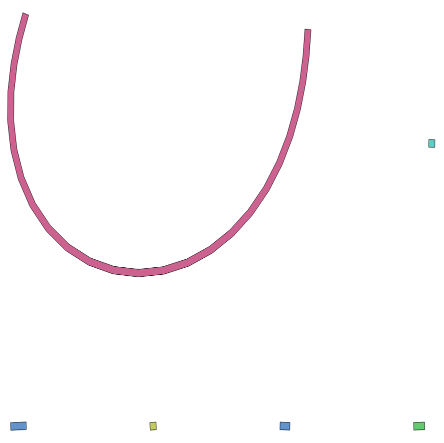
NCTC13359	*Pseudomonas aeruginosa*	37	0	0	1		Finished circular assembly	

						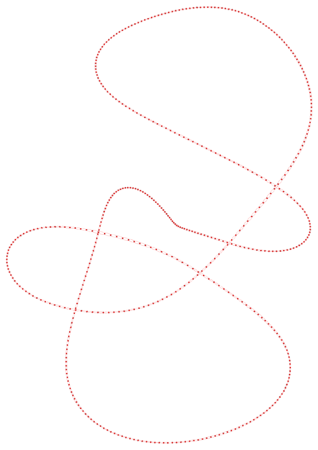		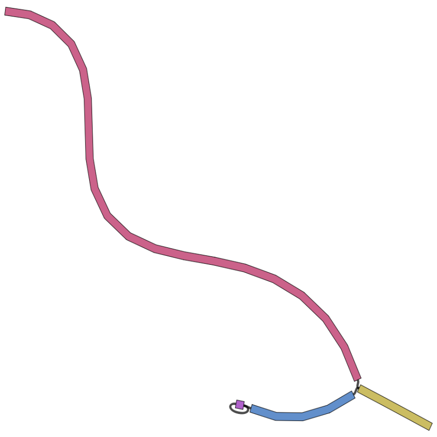
NCTC13360	*Staphylococcus epidermidis*	61	1	7	0	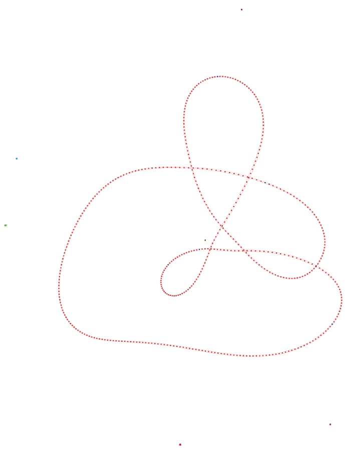	Finished circular assembly	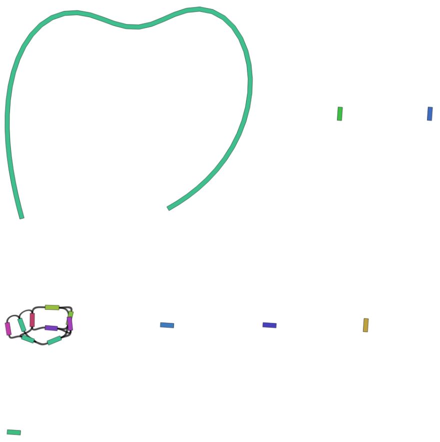
NCTC13368	*Klebsiella pneumoniae*	54	1	1	1	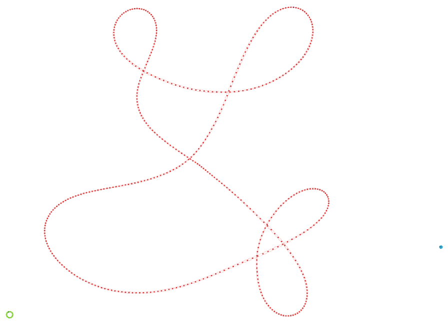	Finished circular assembly	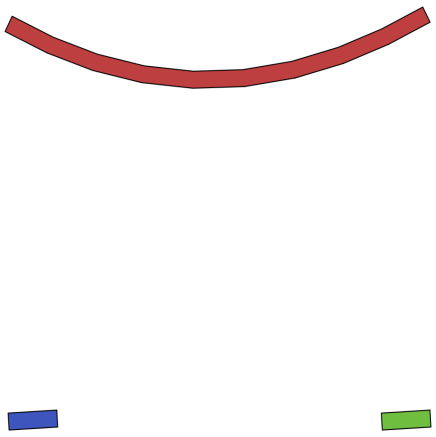
NCTC13373	*Staphylococcus aureus*	70	1	0	1	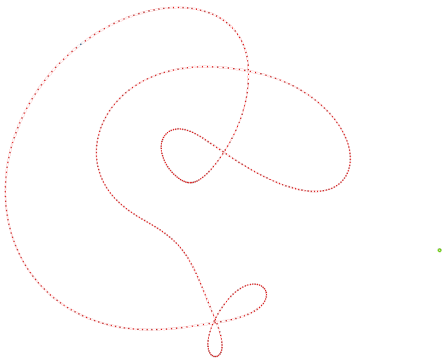	Finished circular assembly	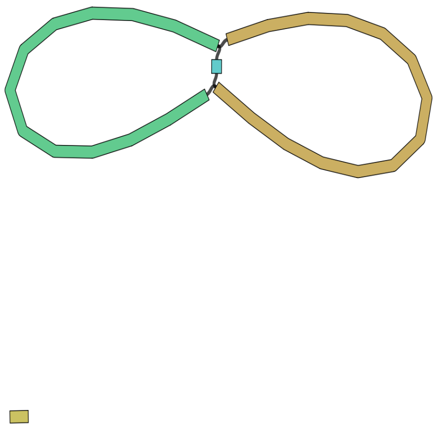
NCTC13376	*Proteus mirabilis*	57	1	1	1	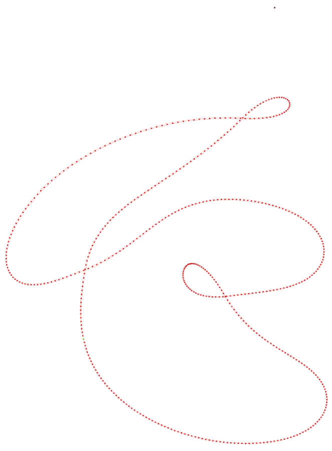	Finished circular assembly	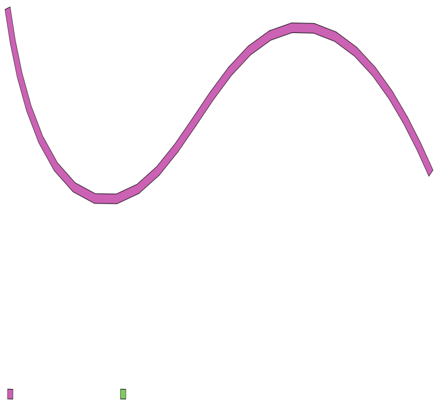
NCTC13377	*Haemophilus influenzae*	113	1	0	0	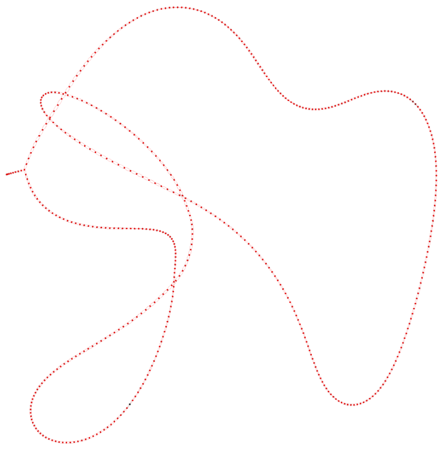	Finished circular assembly	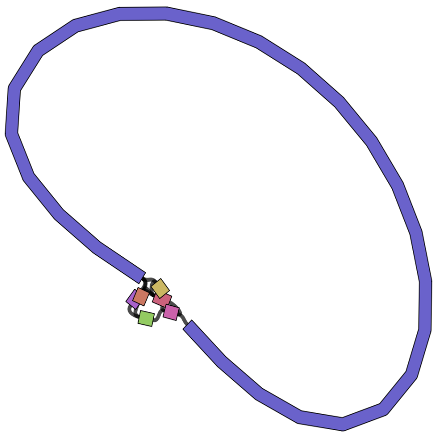
NCTC13384	*Escherichia coli*	18	1	0	5		Mis-assembly/Fragmented	

						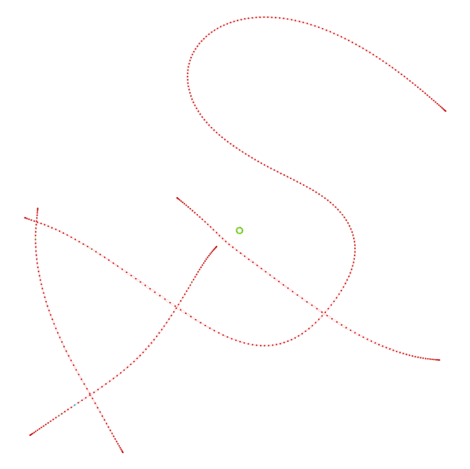		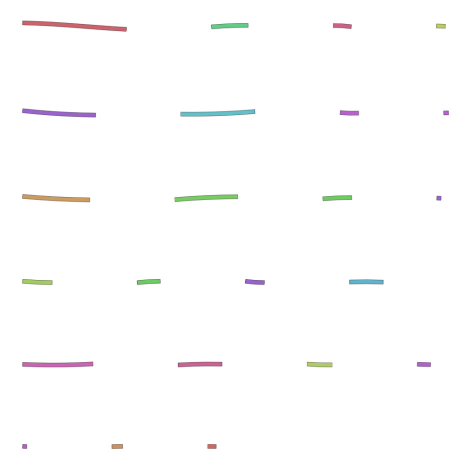
NCTC13394	*Staphylococcus aureus*	149	1	0	0	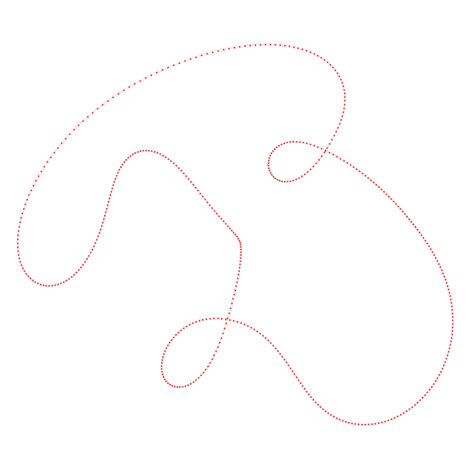	Finished circular assembly	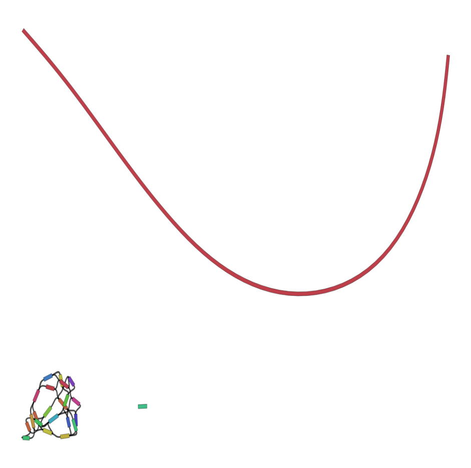
NCTC13395	*Staphylococcus aureus*	53	1	0	0	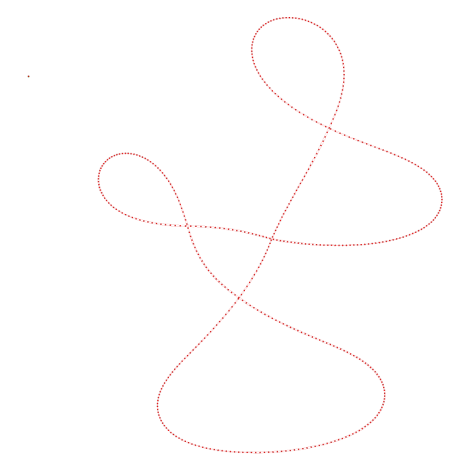	Finished circular assembly	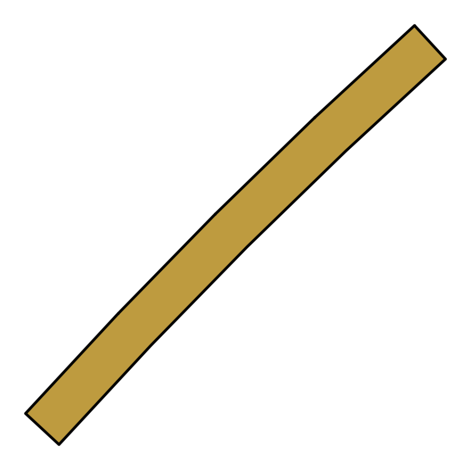
NCTC13400	*Escherichia coli*	82	1	1	0	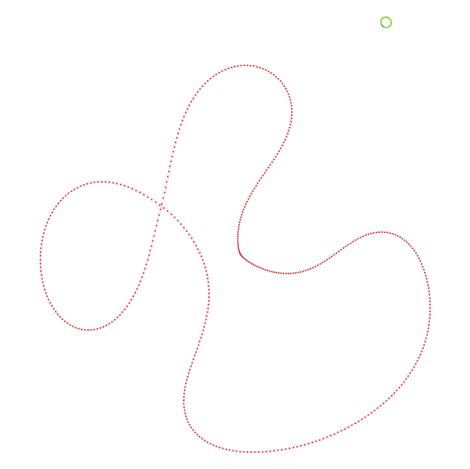	Finished circular assembly	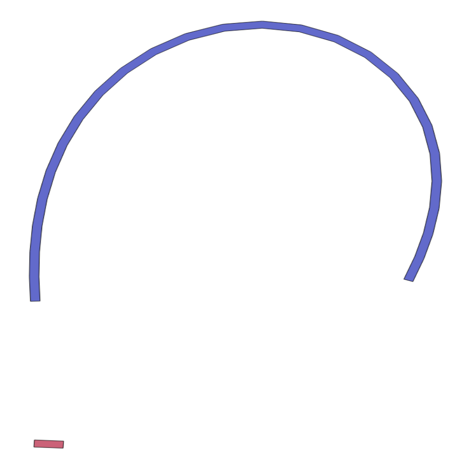
NCTC13405	*Enterobacter cloacae*	50	Pending	Pending	Pending	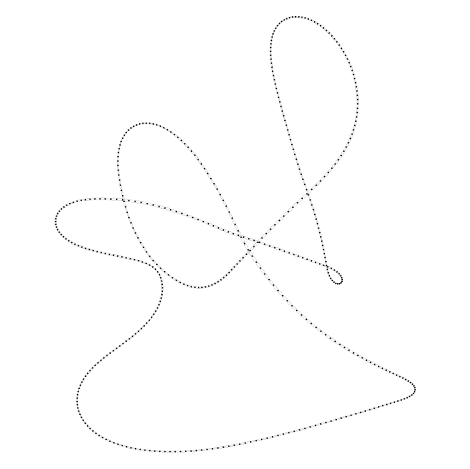	Finished circular assembly	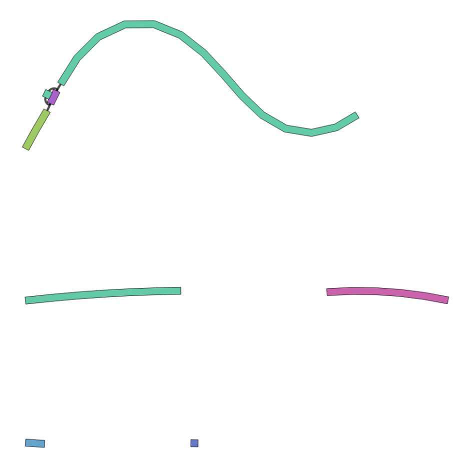
NCTC13406	*Enterobacter cloacae*	70	1	0	2	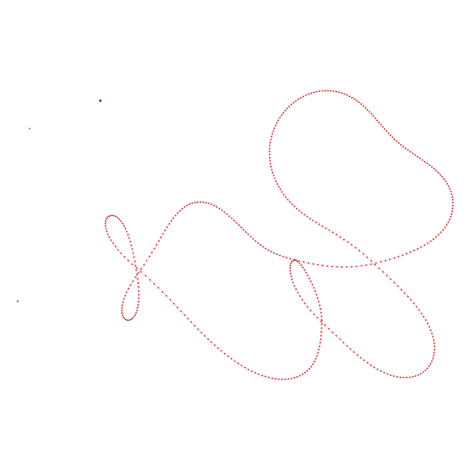	Finished circular assembly	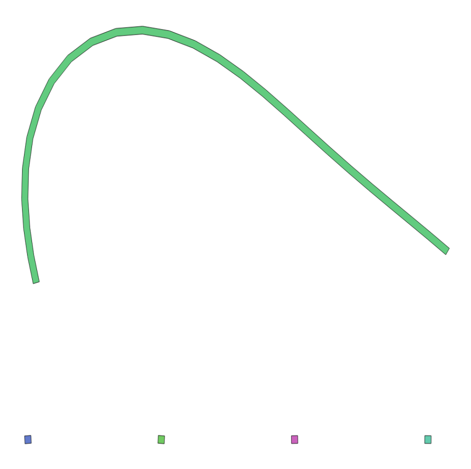
NCTC13420	*Acinetobacter baumannii*	114	0	0	6		Finished circular assembly	

						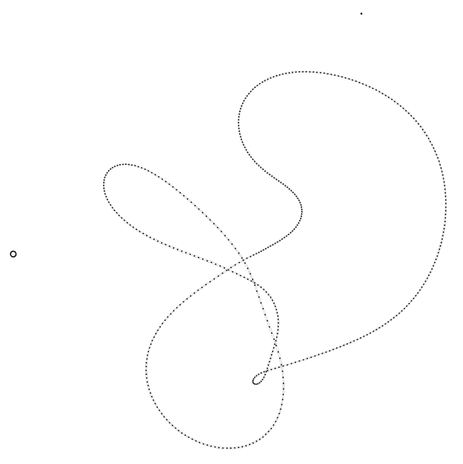		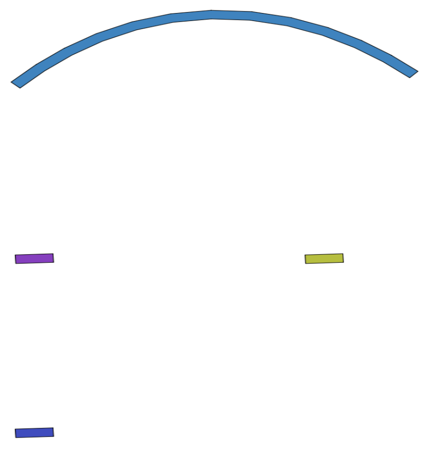
NCTC13421	*Acinetobacter baumannii*	105	0	0	1	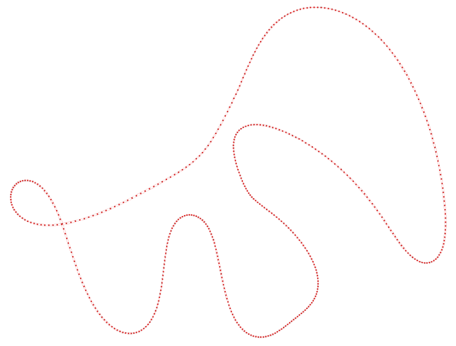	Finished circular assembly	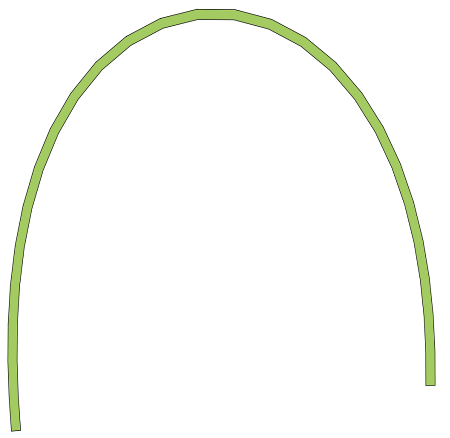
NCTC13422	*Acinetobacter baumannii*	98	1	1	1	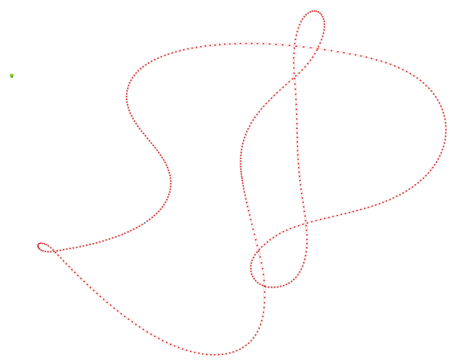	Finished circular assembly	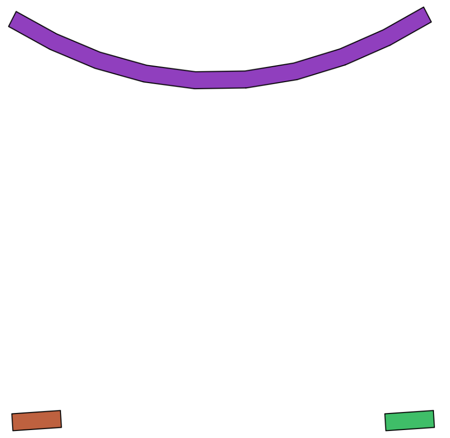
NCTC13424	*Acinetobacter baumannii*	81	1	1	1	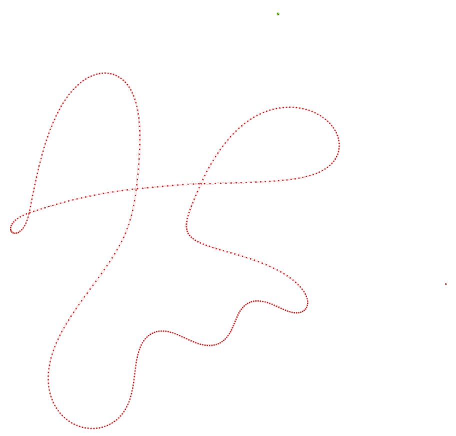	Finished circular assembly	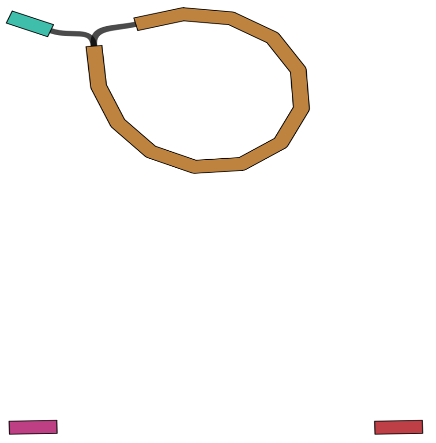
NCTC13434	*Staphylococcus aureus*	148	1	1	0	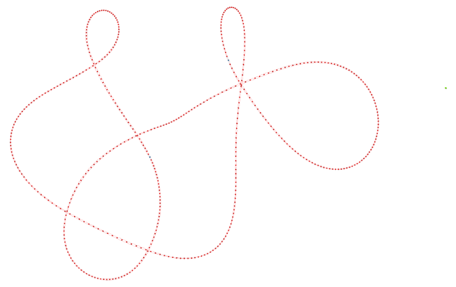	Finished circular assembly	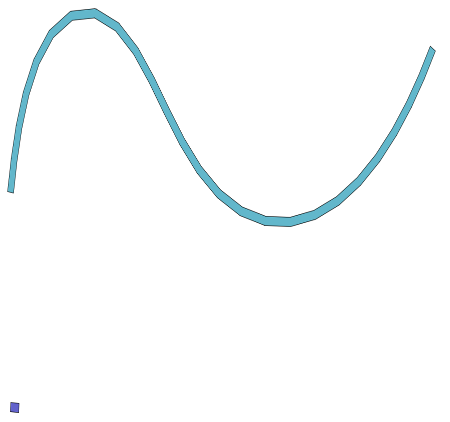
NCTC13435	*Staphylococcus aureus*	61	1	2	0	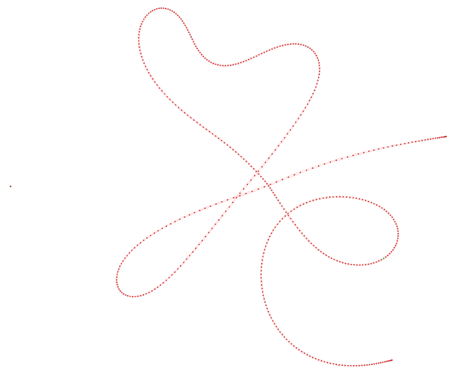	Finished assembly (lacking circularisation)	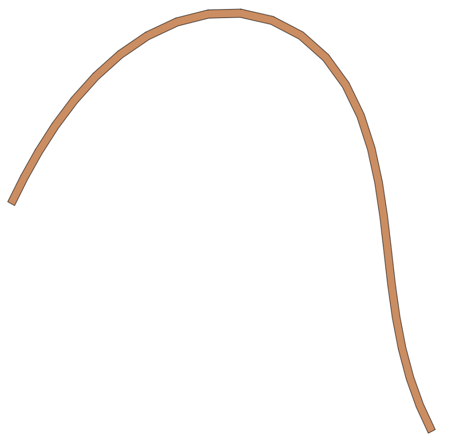
NCTC13437	*Pseudomonas aeruginosa*	29	0	0	3		Mis-assembly/Fragmented	

						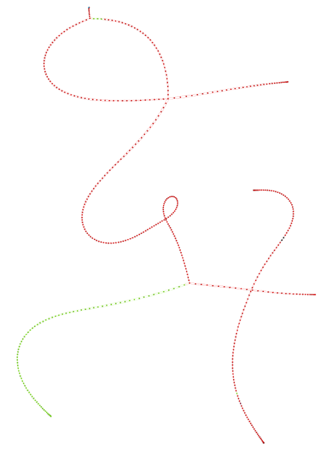		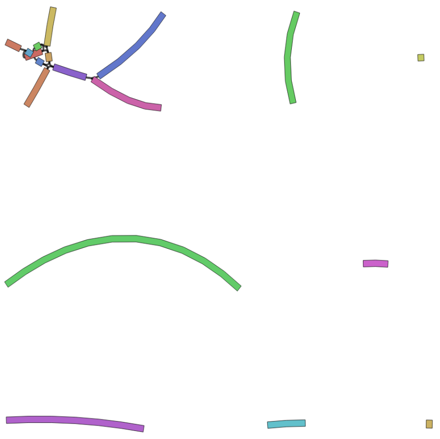
NCTC13438	*Klebsiella pneumoniae*	50	1	0	5	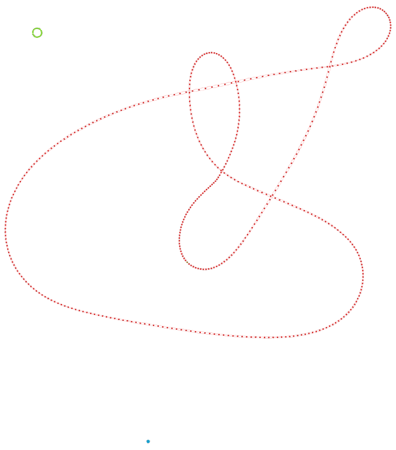	Finished circular assembly	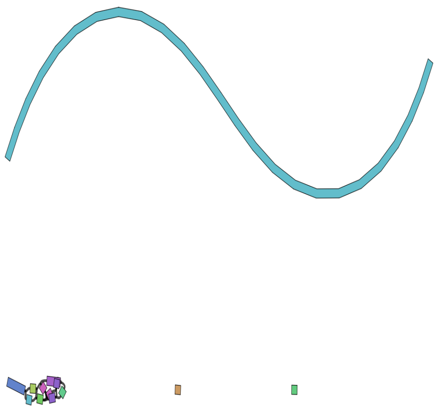
NCTC13439	*Klebsiella pneumoniae*	48	1	3	0	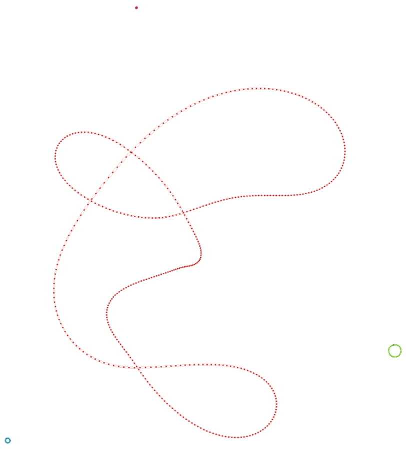	Finished circular assembly	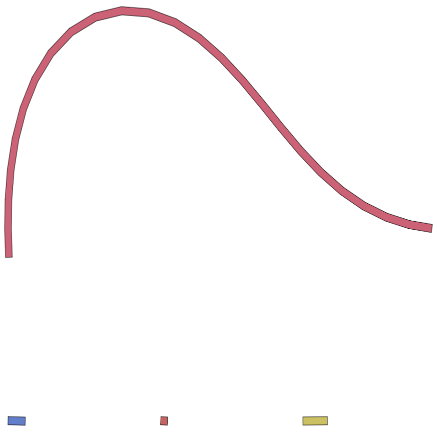
NCTC13440	*Klebsiella pneumoniae*	53	1	2	0	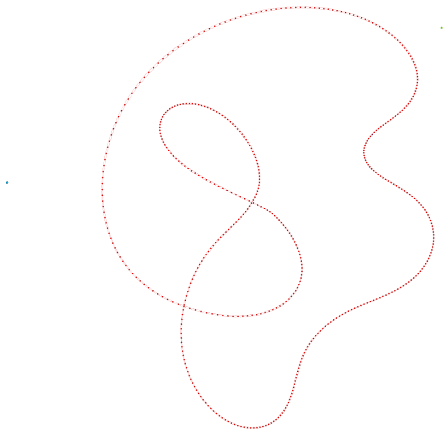	Finished circular assembly	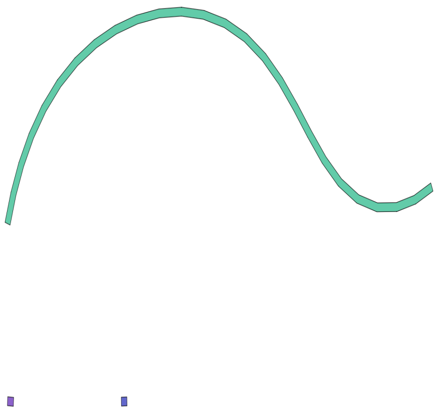
NCTC13441	*Escherichia coli*	55	1	1	5	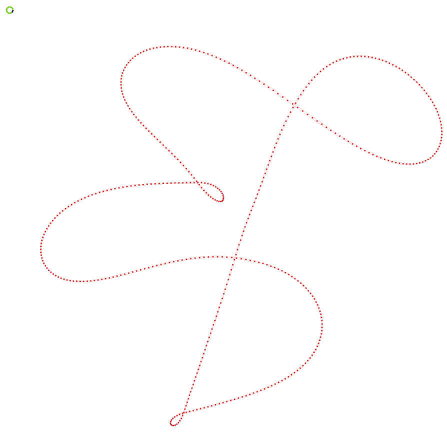	Finished circular assembly	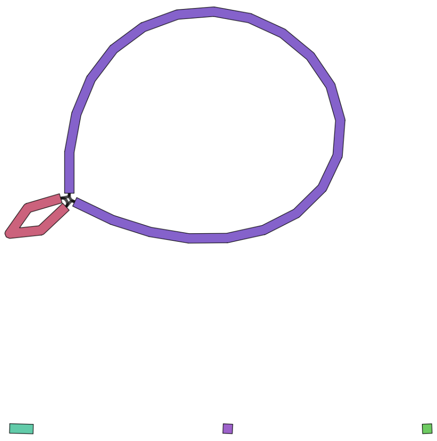
NCTC13442	*Klebsiella pneumoniae*	43	1	2	3	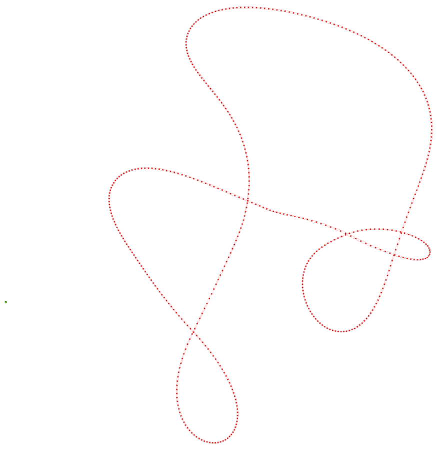	Finished circular assembly	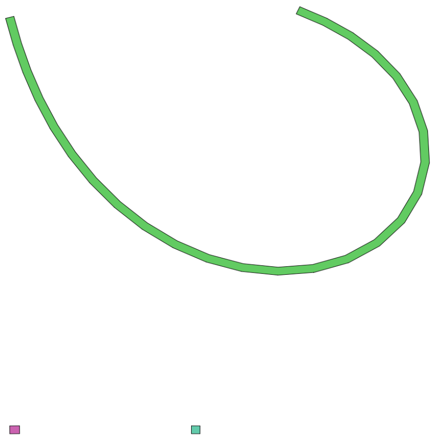
NCTC13443	*Klebsiella pneumoniae*	34	0	0	4		Finished circular assembly	

						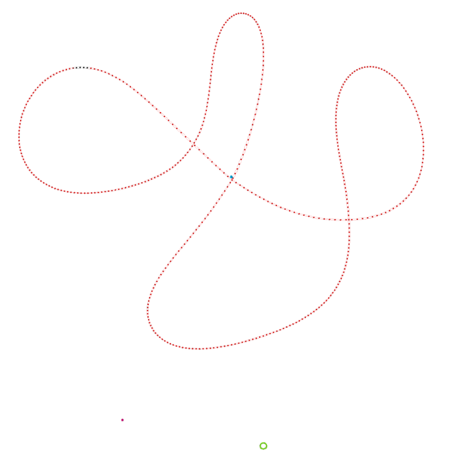		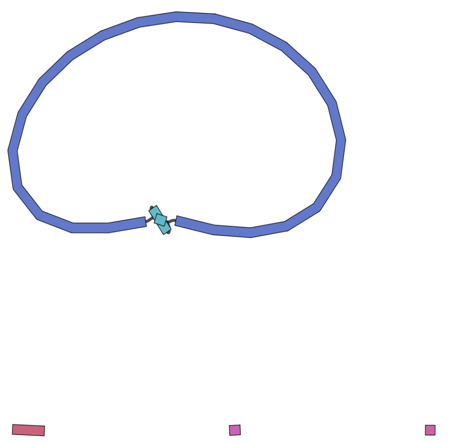	
NCTC13450	*Escherichia coli*	65	1	1	0	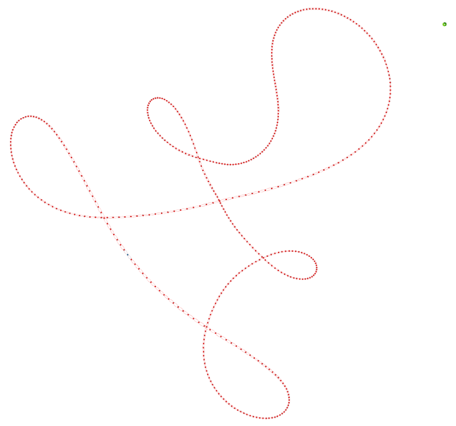	Finished circular assembly	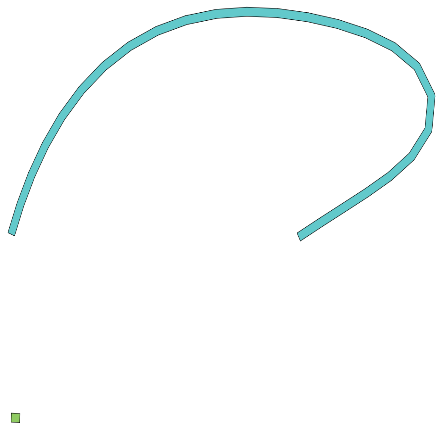	
NCTC13451	*Escherichia coli*	49	1	1	1	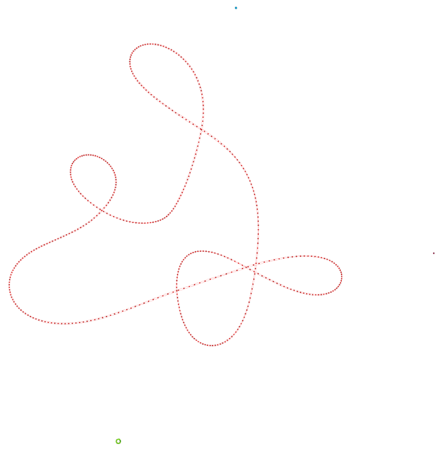	Finished circular assembly	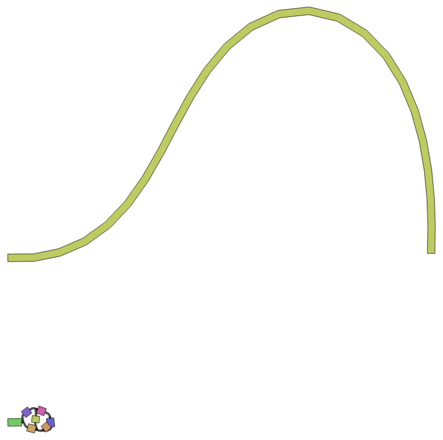	
NCTC13452	*Escherichia coli*	64	1	0	4	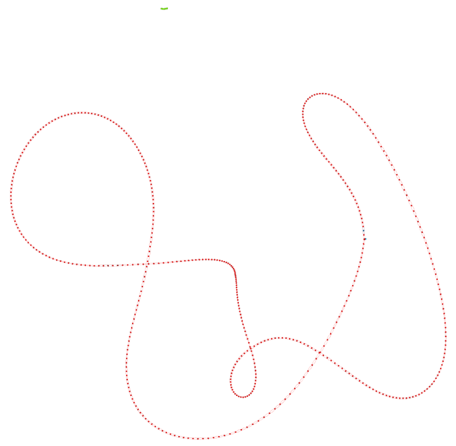	Finished circular assembly	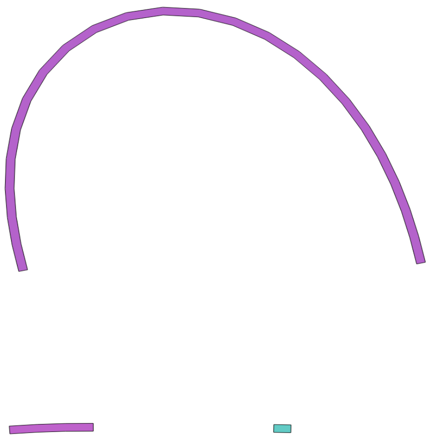	
NCTC13461	*Escherichia coli*	74	0	0	6	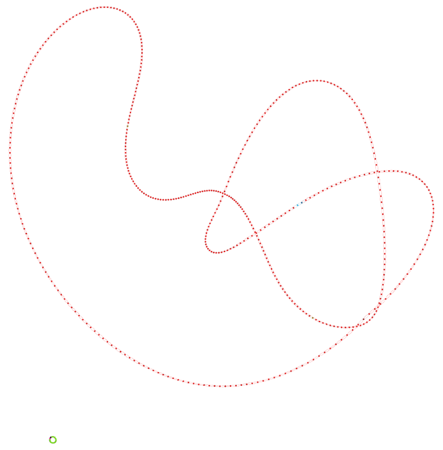	Finished circular assembly	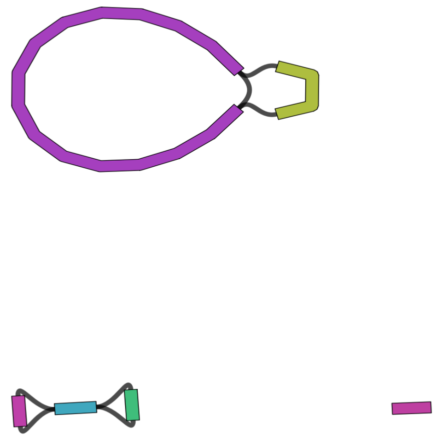	
NCTC13462	*Escherichia coli*	51	0	0	12	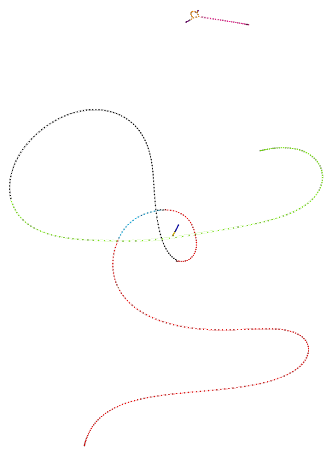	Mis-assembly/Fragmented	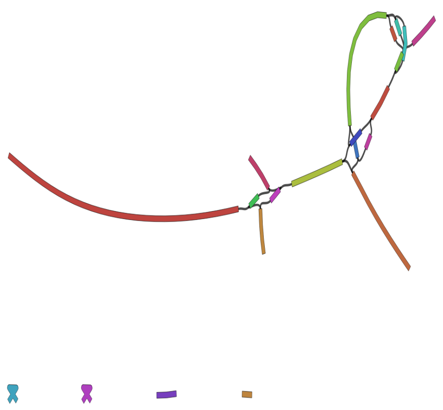	
NCTC13463	*Escherichia coli*	62	1	1	4		Mis-assembly/Fragmented		

						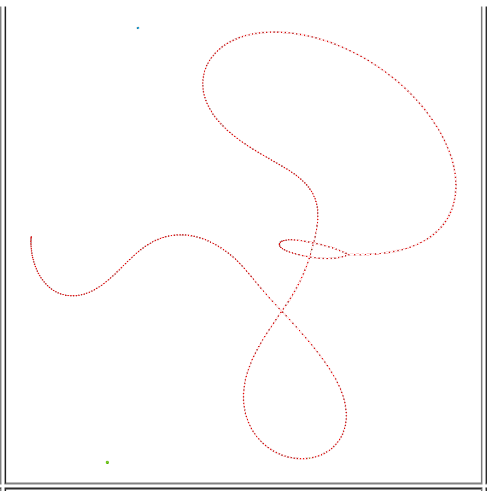		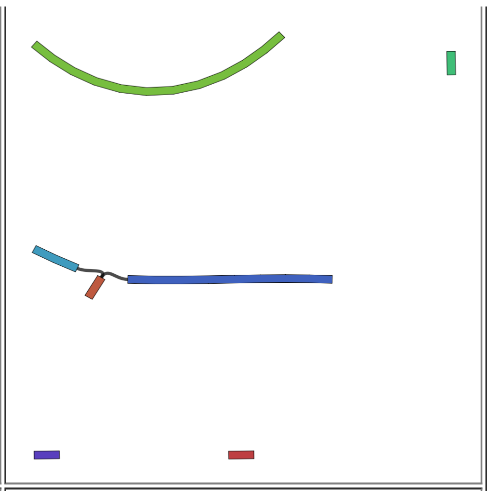
NCTC13464	*Enterobacter cloacae*	73	1	1	0	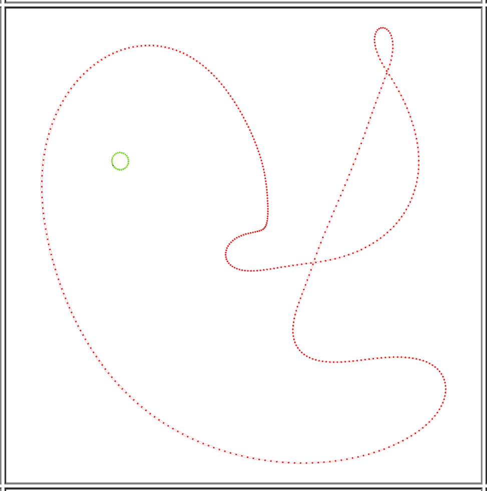	Finished circular assembly	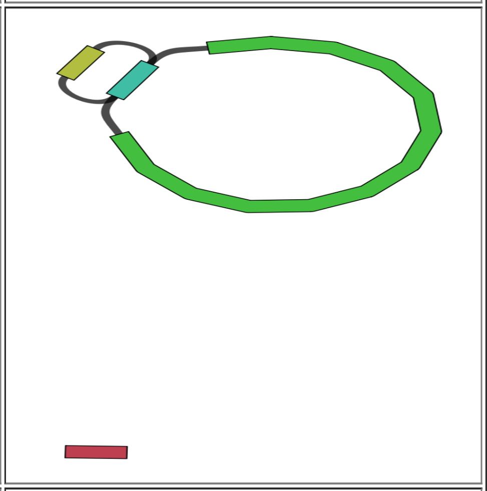
NCTC13465	*Klebsiella pneumoniae*	20	0	0	22	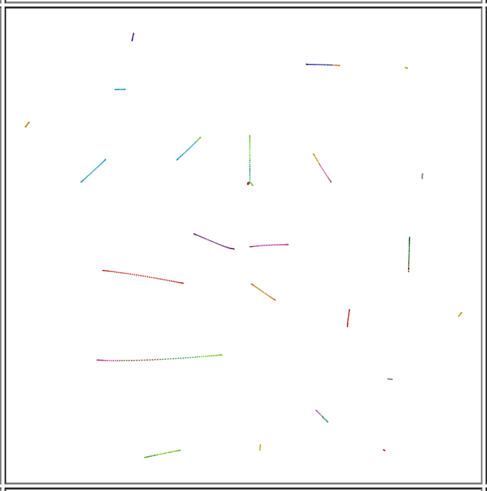	Mis-assembly/Fragmented	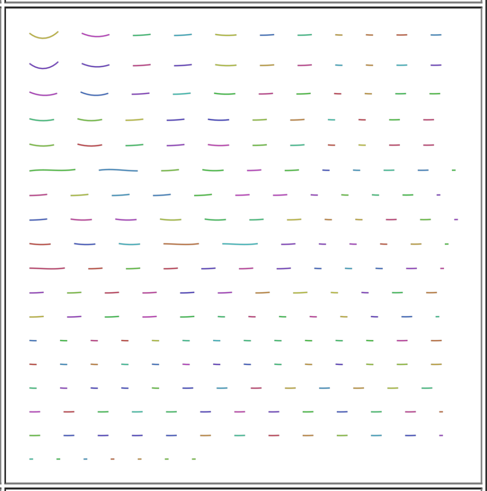
NCTC13476	*Escherichia coli*	10	Pending	Pending	Pending	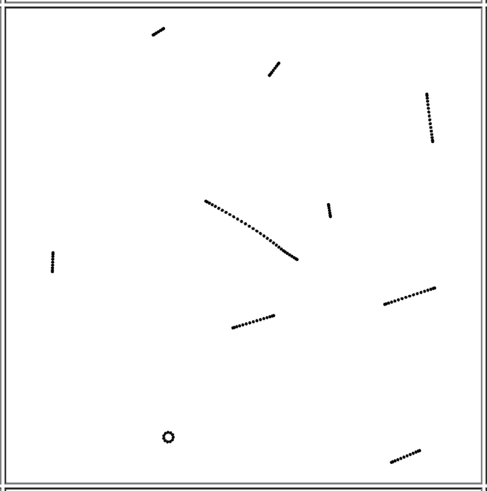	Mis-assembly/Fragmented	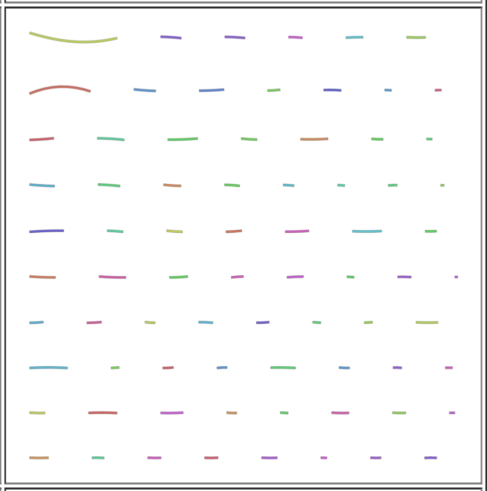
NCTC13483	*Neisseria gonorrhoeae*	150	Pending	Pending	Pending	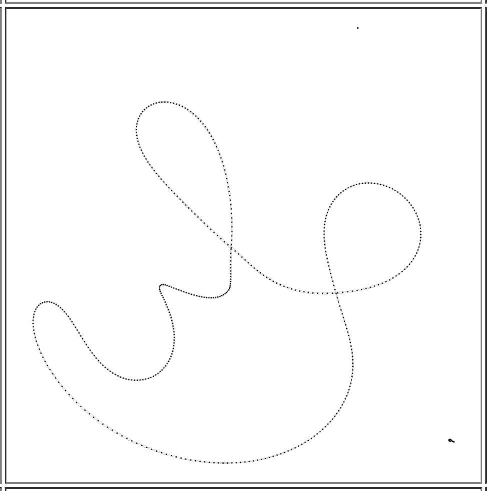	Finished circular assembly	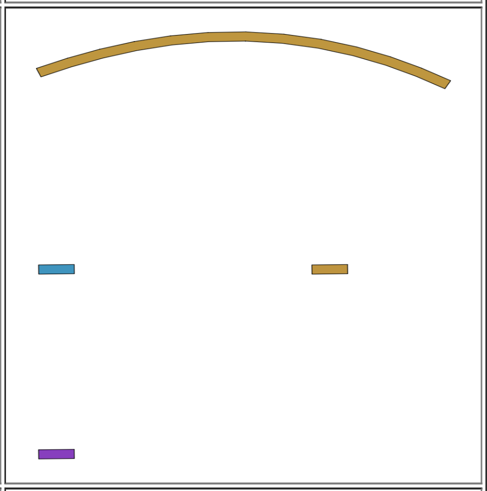
NCTC13532	*Chryseobacterium indoltheticum*	46	1	2	1	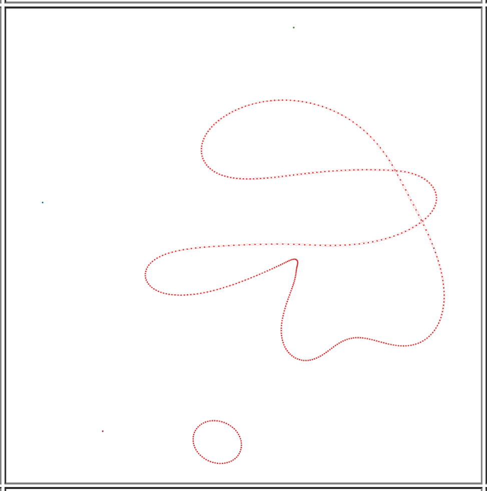	Finished circular assembly	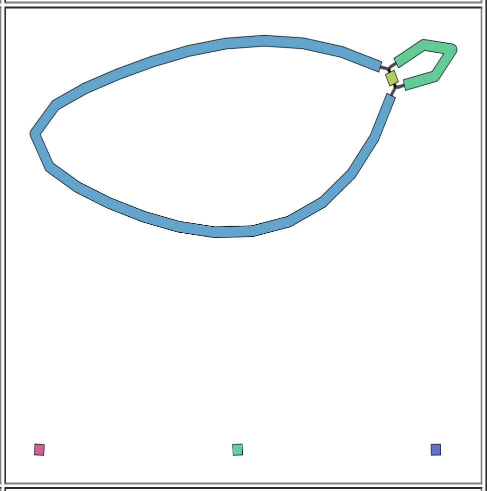
NCTC13533	*Chryseobacterium shigense*	31	0	0	3		Mis-assembly/Fragmented	

						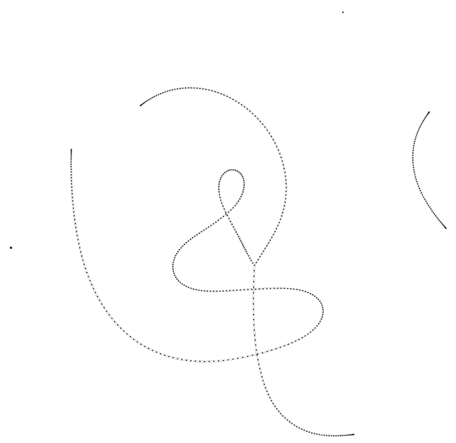		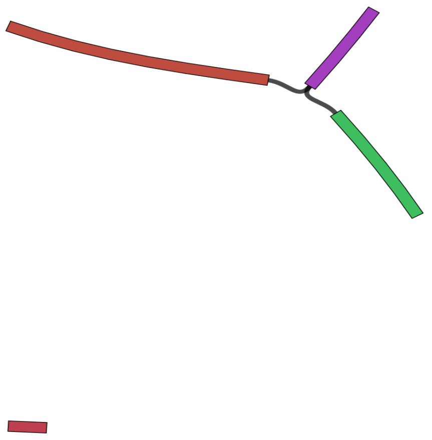
NCTC13534	*Sphingobacterium daejeonense*	39	1	0	0	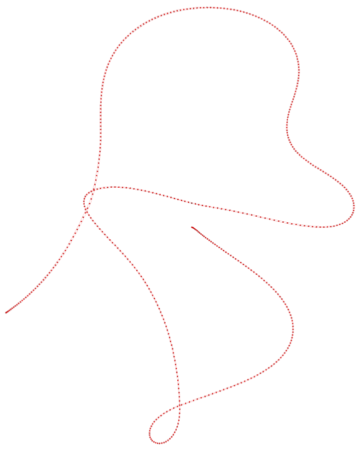	Finished assembly (lacking circularisation)	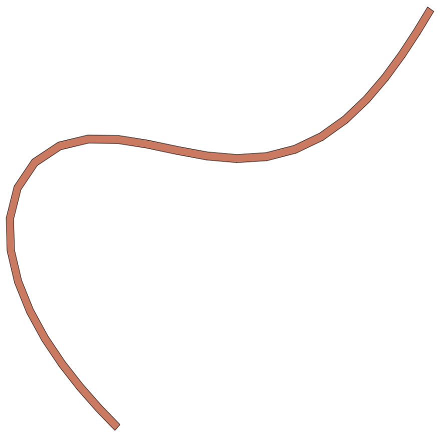
NCTC13543	*Rhizobium radiobacter*	31	0	0	12	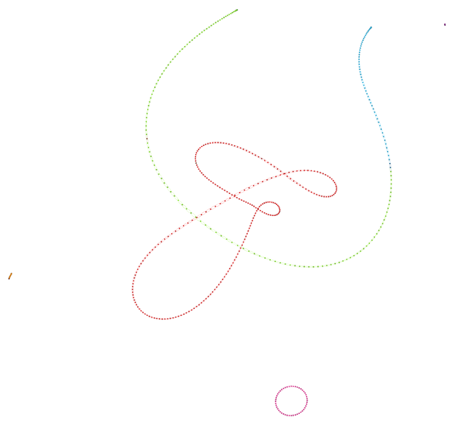	Mis-assembly	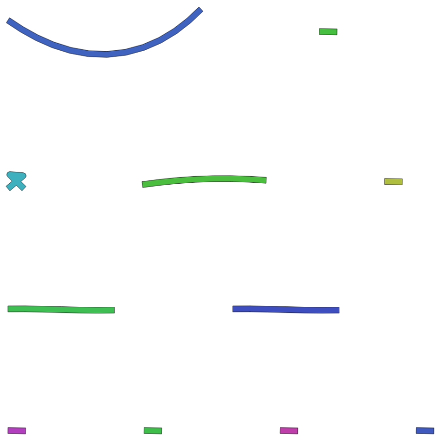
NCTC13552	*Staphylococcus aureus*	62	1	0	0	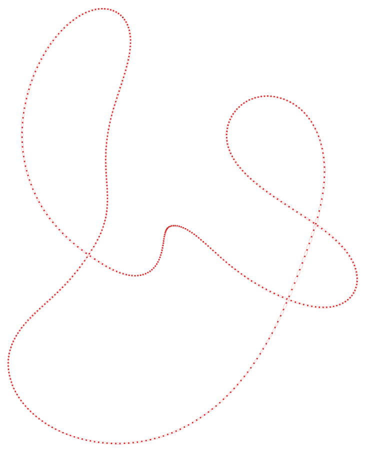	Finished circular assembly	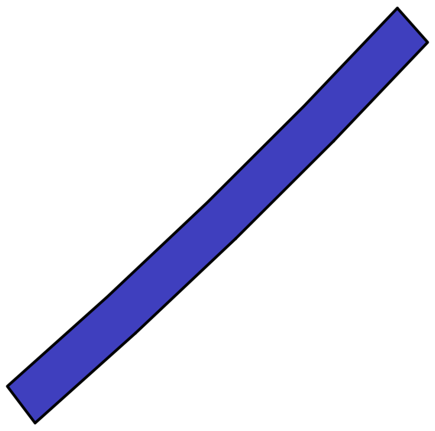
NCTC13585	*Neisseria weaveri*	155	1	0	0	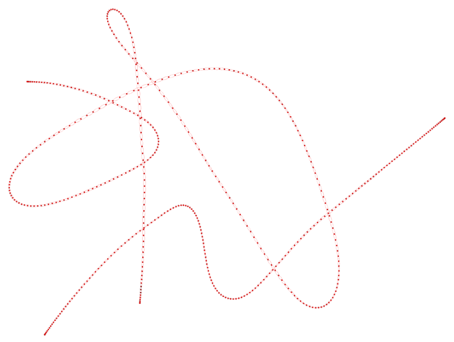	Mis-assembly/Fragmented	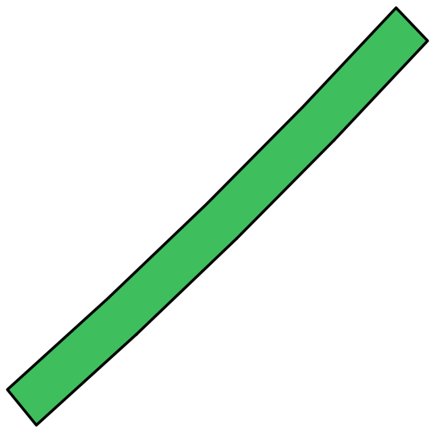
NCTC13616	*Staphylococcus aureus*	250	1	0	0	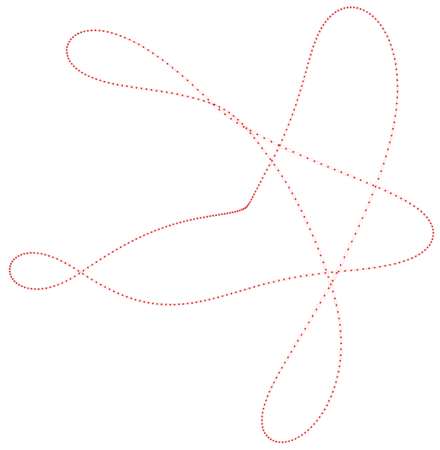	Finished circular assembly	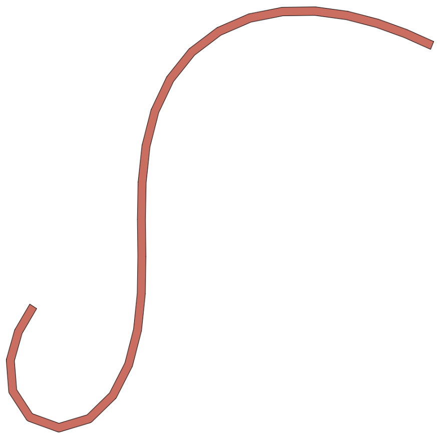
NCTC13618	*Pseudomonas aeruginosa*	43	1	0	0		Finished assembly (lacking circularisation)	

						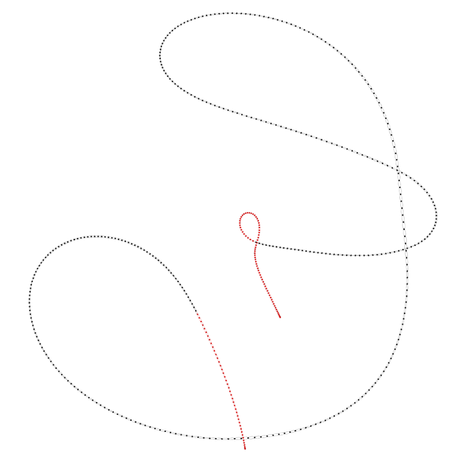		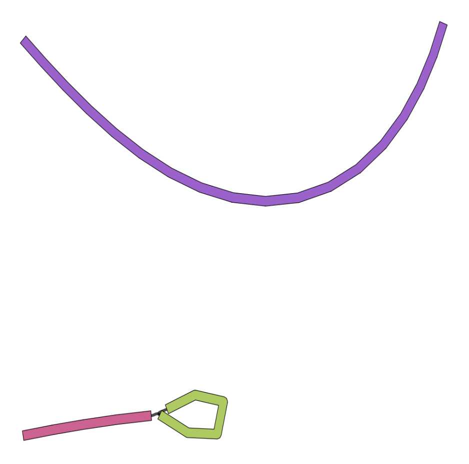
NCTC13619	*Pseudomonas aeruginosa*	26	0	0	5	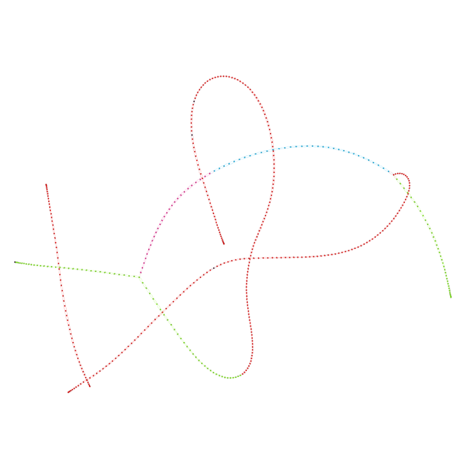	Mis-assembly/Fragmented	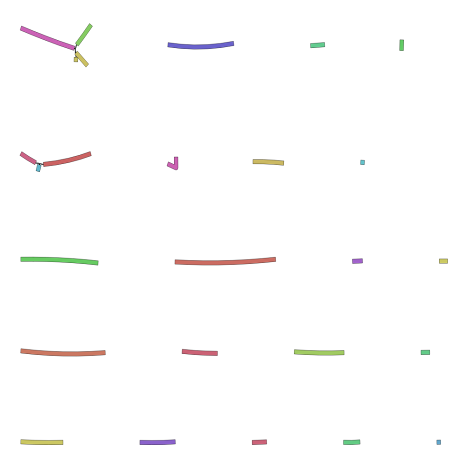
NCTC13620	*Pseudomonas aeruginosa*	33	1	0	0	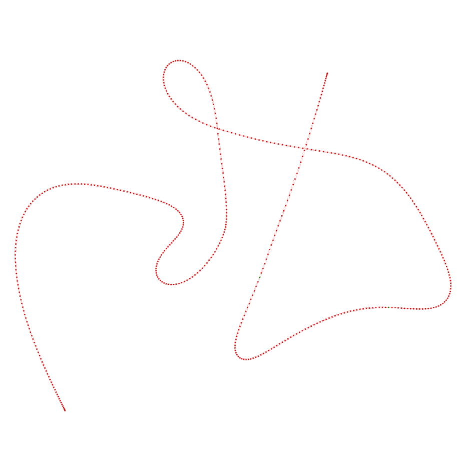	Finished assembly (lacking circularisation)	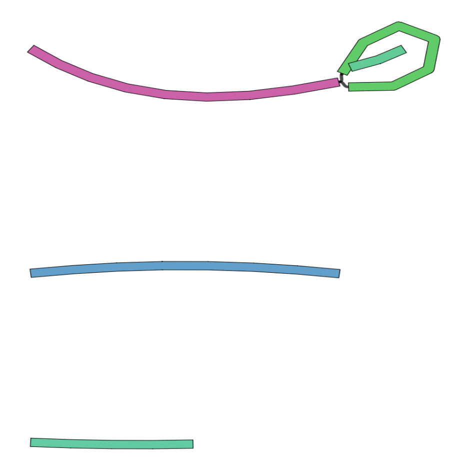
NCTC13621	*Pseudomonas aeruginosa*	23	0	0	31	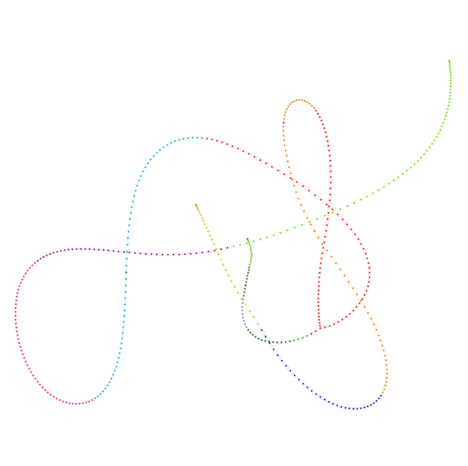	Mis-assembly/Fragmented	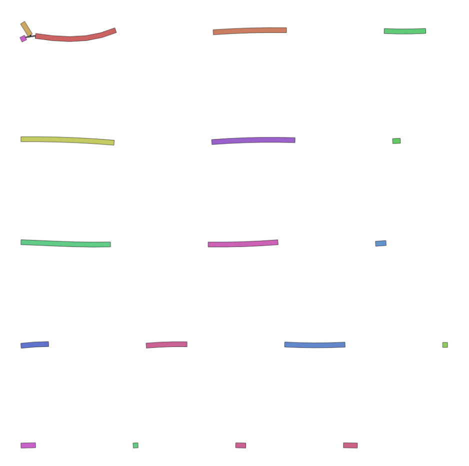
NCTC13626	*Staphylococcus aureus*	222	1	0	1	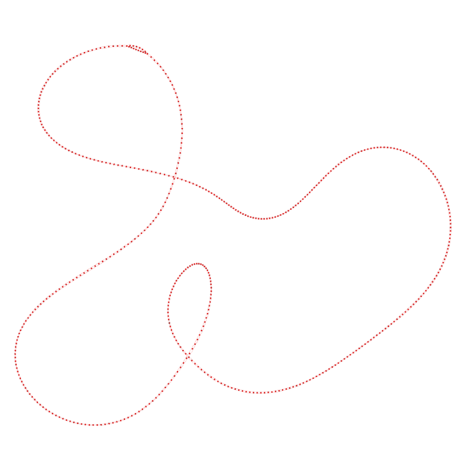	Finished circular assembly	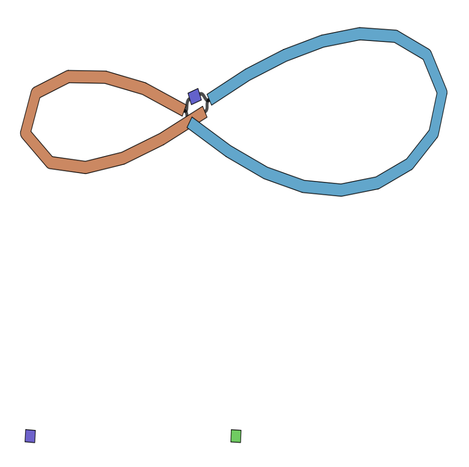
NCTC13628	*Pseudomonas aeruginosa*	47	0	0	9	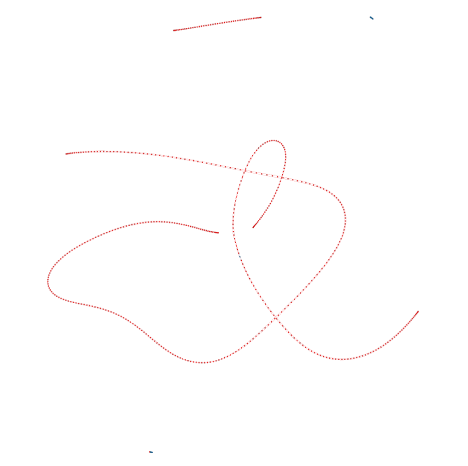	Mis-assembly/Fragmented	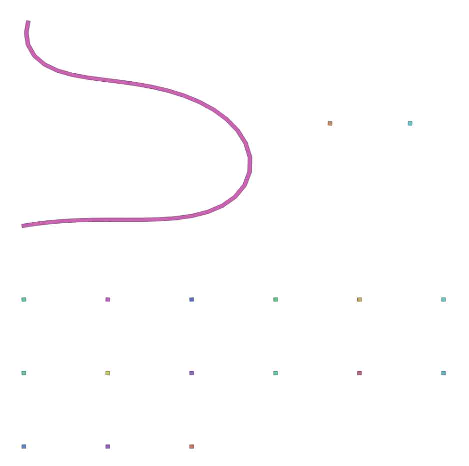
NCTC13629	*Yersinia enterocolitica subsp. enterocolitica*	49	1	0	0		Finished circular assembly	

						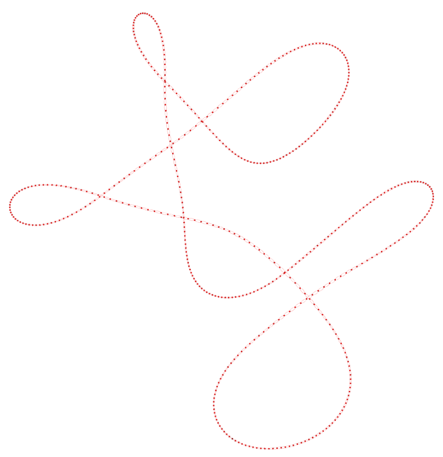		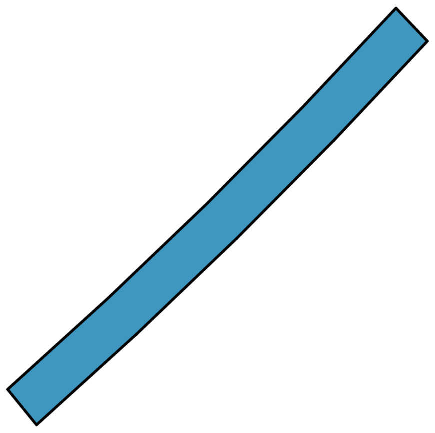
NCTC13630	*Citrobacter braakii*	43	Pending	Pending	Pending	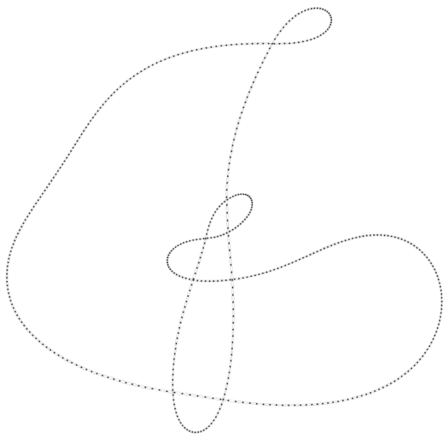	Finished circular assembly	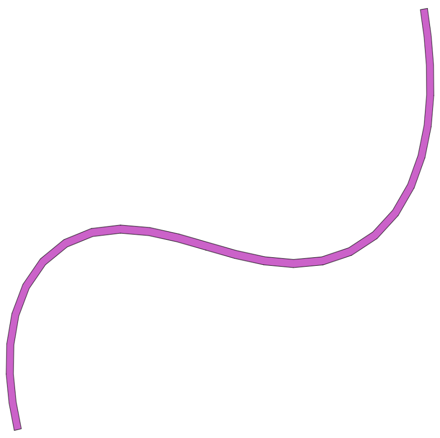
NCTC13634	*Staphylococcus saprophyticus subsp saprophyticus*	105	1	2	1	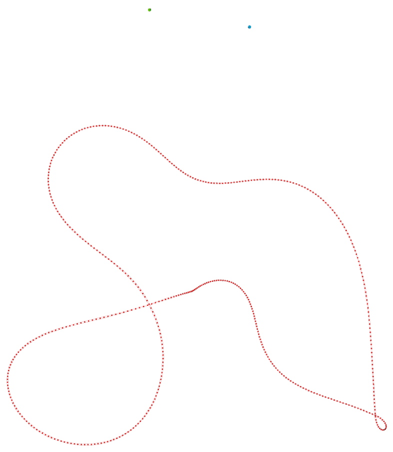	Finished circular assembly	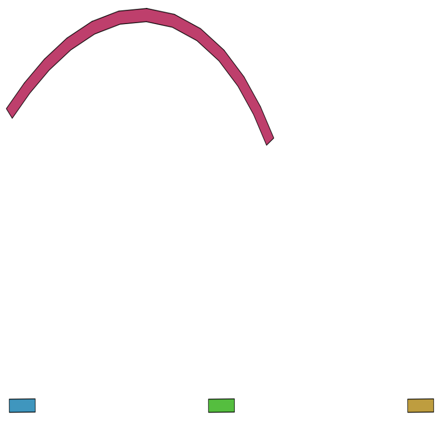
NCTC13635	*Klebsiella pneumoniae*	32	0	0	1	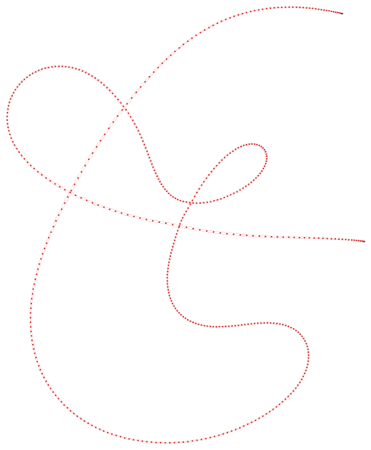	Finished circular assembly	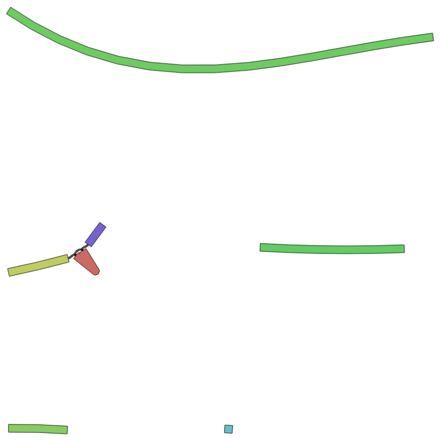
NCTC13639	*Citrobacter freundii*	39	1	1	9	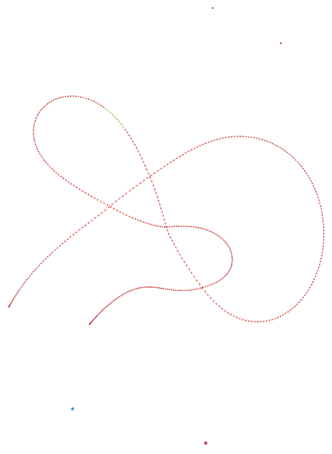	Finished circular assembly	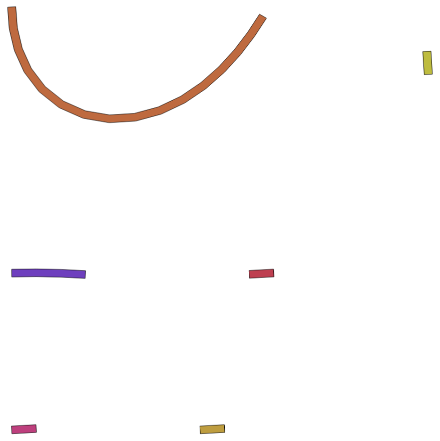
NCTC13647	*Vibrio vulnificus*	36	2	0	0	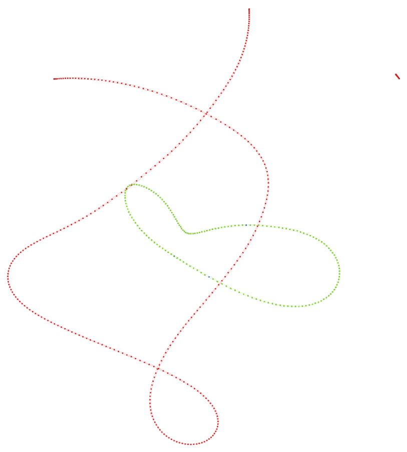	Mis-assembly	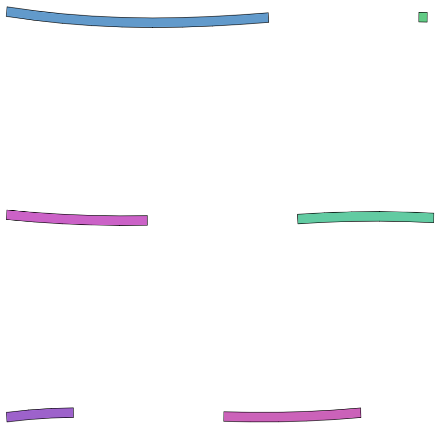
NCTC13667	*Bordetella pertussis*	92	0	0	1		Finished circular assembly	

						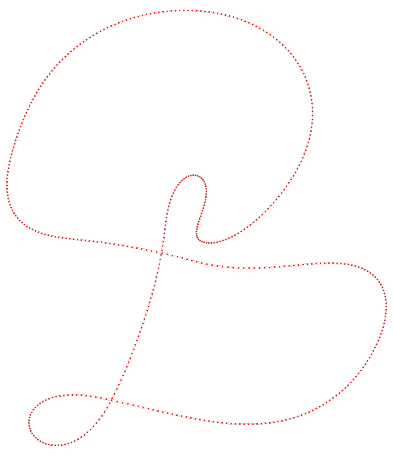		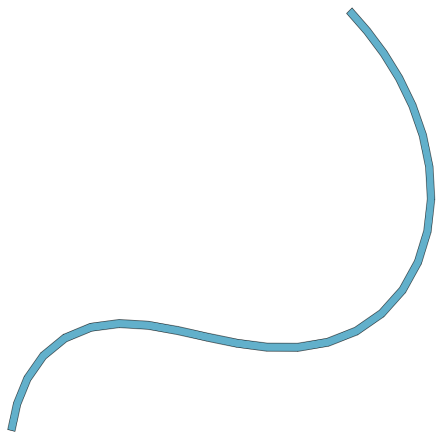
NCTC13668	*Bordetella pertussis*	42	0	0	6	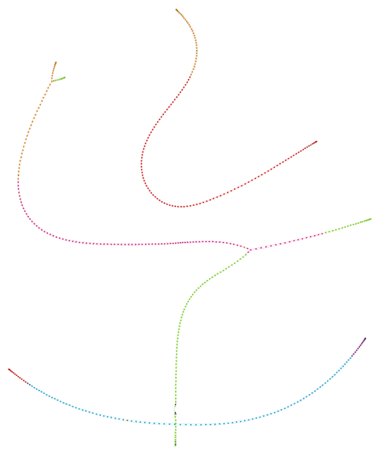	Mis-assembly/Fragmented	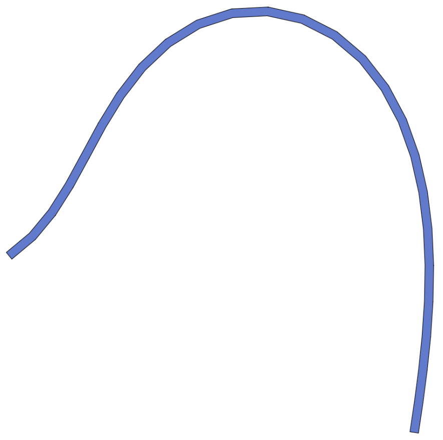
