# Supplementary figures and images for: HINGE: long-read assembly achieves optimal repeat resolution
Source: Genome Res. 2017 May;27(5):747–56. doi: 10.1101/gr.216465.116 (PMC5411769; doi:10.1101/gr.216465.116)

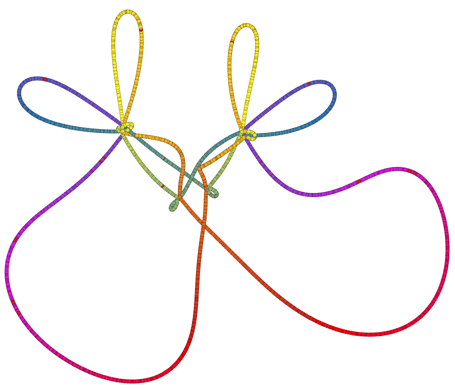

Supplement: Supplemental Material [file supp_gr.216465.116_Supplemental_Source_Code.zip › HINGE-master/misc/ecoli_shortened.png]

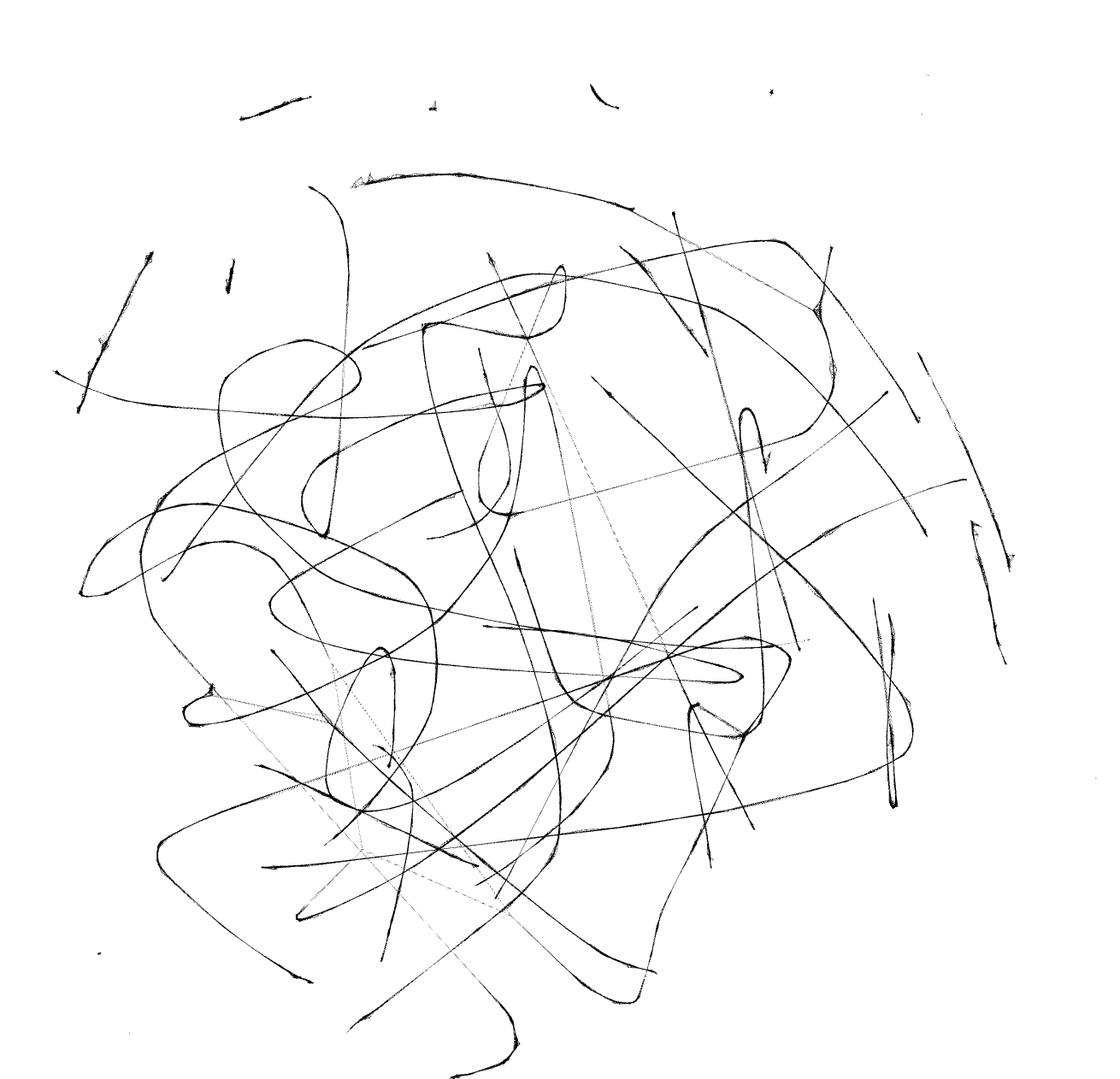

Supplement: Supplemental Material [file supp_gr.216465.116_Supplemental_Source_Code.zip › HINGE-master/misc/Falcon_ecoli_shortened.png]

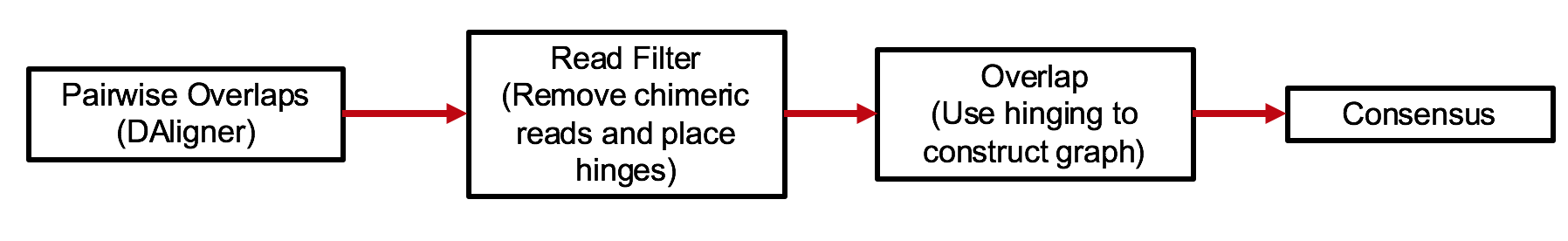

Supplement: Supplemental Material [file supp_gr.216465.116_Supplemental_Source_Code.zip › HINGE-master/misc/High_level_overview.png]

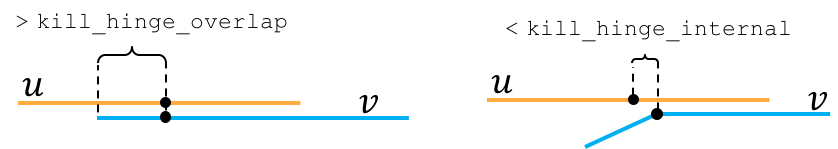

Supplement: Supplemental Material [file supp_gr.216465.116_Supplemental_Source_Code.zip › HINGE-master/misc/param_description1.png]
