# Supplementary material for: HINGE: long-read assembly achieves optimal repeat resolution
Source: Genome Res. 2017 May;27(5):747–56. doi: 10.1101/gr.216465.116 (PMC5411769; doi:10.1101/gr.216465.116)
Supplement: Supplemental Material [file supp_gr.216465.116_Supplemental_Material.pdf]

HINGE: Long-Read Assembly Achieves Optimal Repeat Resolution

Supplementary Material

NCTC ID	Accession Number
NCTC11022	ERS718598
NCTC9964	ERS747607
NCTC9657	ERS812515
NCTC4450	ERS846863
NCTC5052	ERS739094
NCTC12158	ERS659593
NCTC9006	ERS718587
NCTC9007	ERS715463
NCTC9016	ERS718589
NCTC9024	ERS702126
NCTC8333	ERS764944
NCTC8781	ERS744800
NCTC6134	ERS806215
NCTC7921	ERS744794
NCTC9002	ERS764942
NCTC9012	ERS718588
NCTC9103	ERS747613
NCTC10864	ERS715414
NCTC11692	ERS659589
NCTC12993	ERS473437
NCTC11962	ERS846861

Supplemental Table S1: Accession numbers of all the datasets used explicitly in the manuscript. The accession numbers of all 997 datasets is in the accompanying excel file.

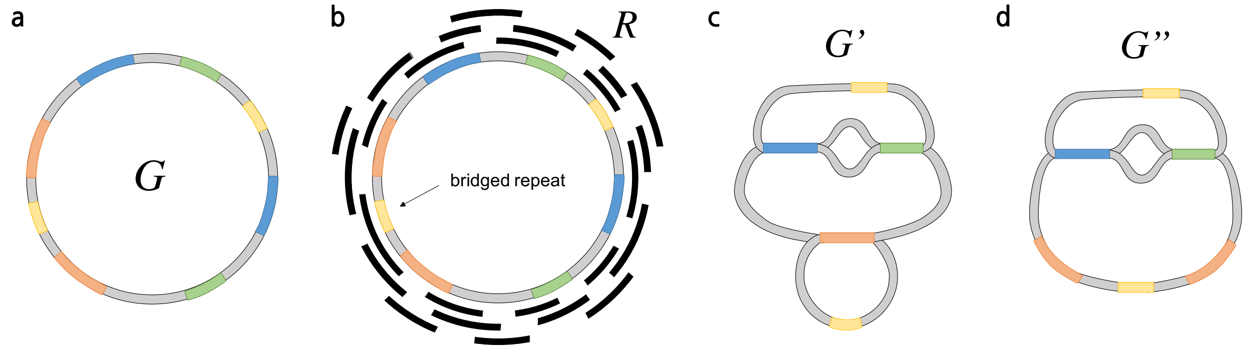

Supplemental Figure S1. Maximal Repeat Resolution. In the idealized case of error-free reads, we define the notion of Maximal Repeat Resolution as follows. **(a)** First we let *G* be the cyclic assembly graph corresponding to the perfect circular genome reconstruction. **(b)** Given a set of reads *R*, each repeat in the genome can be classified as bridged or unbridged. **(c)** The graph *G'* is defined by collapsing the segments in *G* corresponding to repeats that are unbridged by *R*. **(d)** The graph *G''* is defined by resolving any loops that can only be traversed in a single way. We call *G''* the maximally resolved assembly graph. We point out that the final loop resolution step (from G' to G'') is based on a parsimony principle, but it could be potentially incorrect if the loop is supposed to be traversed multiple times. This behavior can be prevented on HINGE by setting the parameter MAX_PLASMID_LENGTH to a value larger than the genome length. We also point out that when reads have errors, the definition of maximal repeat resolution is not as clean, since one must instead consider approximate repeats, where the level of approximation should be determined as a function of the error rate. For the purpose of this paper, we assume that the matches found by the alignment tool correspond to repeats that are similar enough to justify their collapsing on the graph. However, as explained in the end of the *Discussion* section, one could in principle carefully analyze the divergence between reads that were matched by the aligner, in order to try to resolve the graph further.

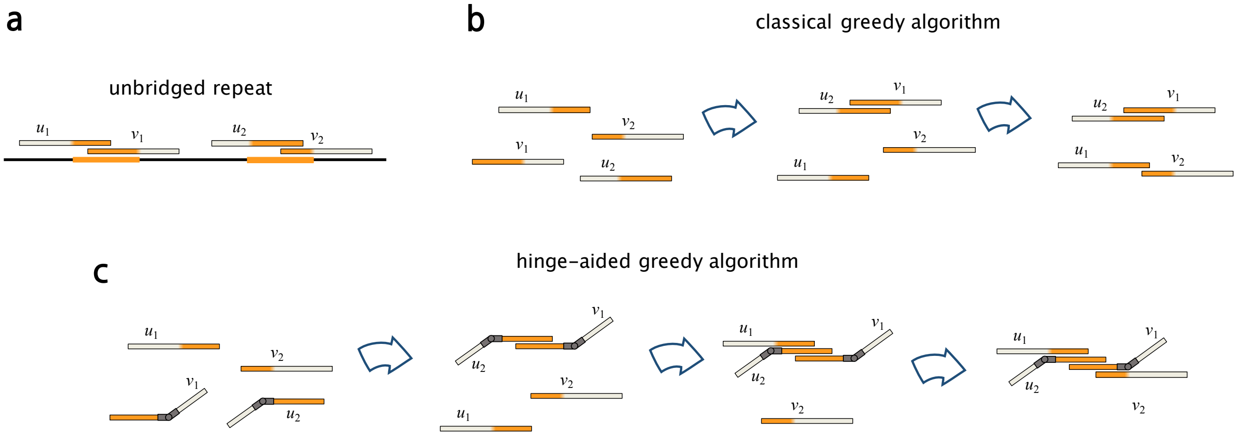

Supplemental Figure S2. Comparison between the classical greedy assembly algorithm and the hinge-aided greedy algorithm. (a) shows a hypothetical scenario where there is an unbridged repeat on a genome, whose length is comparable to the read length. **(b)** shows the steps carried out by the classical greedy algorithm. Read u_2 first picks v_1 as its successor and then u_1 picks v_2 as its successor, producing a mis-assembly. **(c)** shows the steps carried out by the hinge-aided greedy algorithm. Here an in-hinge is placed on u_2 because it is the read starting before the repeat that extends farthest into the repeat. Similarly, an out-hinge is placed on v_1 as it is the read ending outside the repeat starting earliest in the repeat. A greedy selection of matches then follows. First, u_2 picks v_1 as its successor. The successor of u_1 is the in-hinge at u_2. Notice that this is a larger match then the overlap between u_1 and v_2. Similarly, v_2 picks the out-hinge at v_1 as its predecessor. Thus the hinge-aided greedy algorithm leads to the proper collapsing of the unbridged repeat into a single segment.

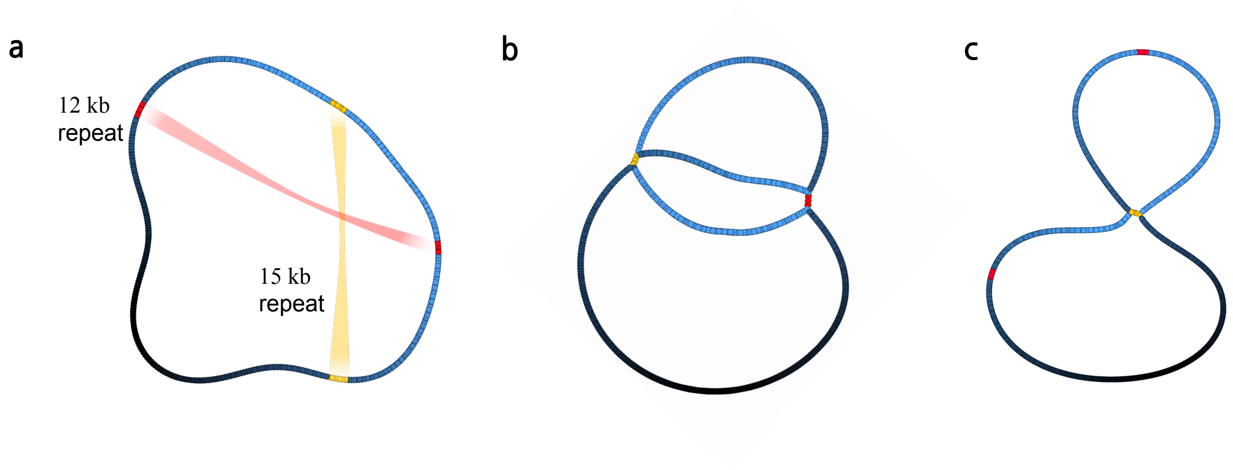

Supplemental Figure S3. We considered a simulated read dataset from a randomly generated genome of length 750 kbp, with planted repeats of lengths 12 kbp (in red) and 15 kbp (in yellow). The reads generated with the DAZZ-DB simulator bridged both repeats allowing perfect assembly to be obtained as shown in **(a).** We then cut reads that were longer than 10 kbp down to length 10 kbp. Now both repeats are unbridged and the graph obtained is as shown in **(b).** Since the red and yellow repeats were interleaved, the resulting graph allows two traversals, and cannot be resolved uniquely. **(c)** In another experiment, we truncated reads of length more than 14 kbp. As a result, only the long repeat (in yellow) was unbridged, and the maximally resolved assembly graph that only collapses the unbridged repeat was obtained**.** As this graph only allows one possible traversal, it can be resolved by HINGE (not shown here) yielding perfect assembly.

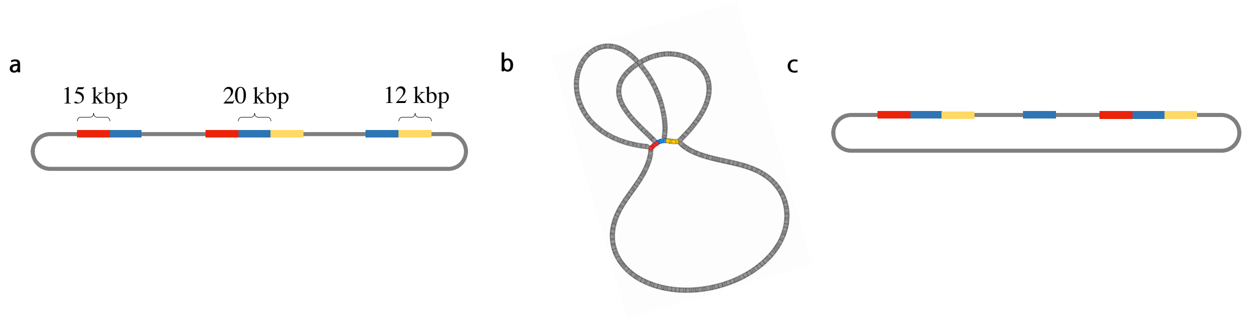

Supplemental Figure S4. (a) We generated a 2 Mbp genome with the nested repeat pattern shown above, formed by a triple repeat of length 20 kbp, and two “flanking repeats”, one of length 15 kbp and one of length 12 kbp. We then generated ten thousand reads using the DAZZ-DB simulator. **(b)** HINGE produces a graph that correctly captures this repeat structure. **(c)** Notice that the HINGE graph admits an alternative traversal that is also consistent with the reads.

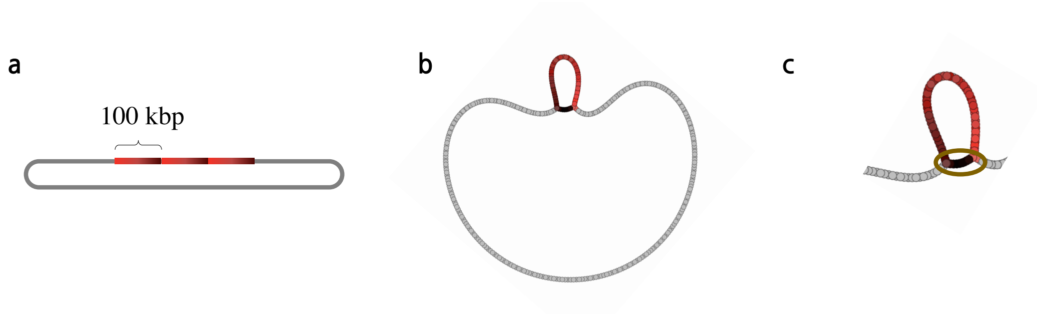

Supplemental Figure S5. (a) We generated a 4 Mbp genome with three tandem repeats of length 100 kbp. The length was chosen so that the loop on the graph would be large enough to be visible. We then generated reads using the DAZZ-DB simulator at a 50x coverage. **(b)** HINGE produces a graph where all three copies of the tandem repeat are represented in a single loop. This loop is not resolved by HINGE since its length is below the default value of MAX_PLASMID_LENGTH. Notice that the graph does not capture the number of copies of the tandem repeat. A post-processing coverage depth analysis can be used to determine the multiplicity of the loop. We note that the reads colored in black and circled in **(c)** are reads that appear at the junction of two copies of the tandem repeat.

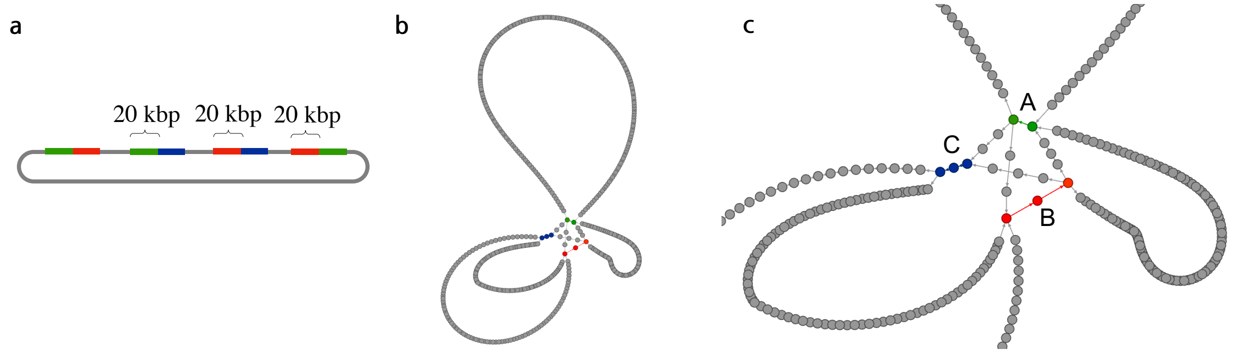

Supplemental Figure S6. (a) We generated a 5 Mbp genome with the repeat pattern AB, AC, BC, BA, where A, B and C are 20 kbp long. We then generated reads using the DAZZ-DB simulator at a 50x coverage. **(b)** HINGE produces a graph that correctly captures this repeat structure. **(c)** Zooming into the location of the repeats in the graph, we see that the segments AB, AC, BC and BA are correctly captured by the graph. The colored segments correspond to the repeats in (a), and the grey nodes connecting the colored segments correspond to reads that lie between two of the segments A, B and C.

Genome	#Chromosomal Contigs	Total length (bp)	N50	Identity (%)
NCTC10081	1	4,877,359	-	99.63
NCTC10538	1	4,823,233	-	99.60
NCTC11358	1	5,469,178	-	98.67
NCTC11637	1	1,697,088	-	99.53
NCTC11696	1	6,003,688	-	99.55
NCTC12066	1	1,753,803	-	99.65
NCTC7102	1	4,956,981	-	99.80
NCTC8198	1	1,927,056	-	99.57
NCTC9035	1	5,031,051	-	99.10
NCTC9171	1	5,176,202	-	99.74
E. coli K12 (PacBio)	1	4,674,870	-	99.9
E. coli K12 (Oxford Nanopore R9)	1	4,642,358	-	95.24
E. coli K12 (Oxford Nanopore R9)	1	4,642,358	-	97.26 (after Racon)
S. cerevisiae W303 (PacBio)	22	12,401,587	789,778	98.95

Supplemental Table S2: Validation of HINGE on several datasets. For NCTC datasets, we randomly picked 10 datasets for which HINGE and the NCTC pipeline obtained a finished assembly, and the identity was computed with respect to the reference provided by NCTC (built using HGAP, circulator, and manual tuning). The PacBio *E. coli* dataset used is at http://files.pacb.com/datasets/secondary-analysis/ecoli-k12-P4C2-20KSS/ecoliK12.tar.gz and the reference used to quantify sequence integrity is provided at http://files.pacb.com/datasets/secondary-analysis/ecoli-k12-P4C2-20KSS/ecoliK12_polished_assembly.fasta. The Oxford Nanopore R9 *E. coli* dataset is the standard dataset provided at http://s3.climb.ac.uk/nanopore/R9_Ecoli_K12_MG1655_lambda_MinKNOW_0.51.1.62.all.fasta and the reference used is at http://www.ncbi.nlm.nih.gov/nuccore/545778205?report=fasta. The *S. cerevisiae* dataset used is provided at https://gist.github.com/pb-jchin/6359919 and the reference is at http://datasets.pacb.com.s3.amazonaws.com/2013/Yeast/HGAP_Assembly/polished_assembly.fasta. For the Oxford Nanopore *E. coli* dataset, we additionally ran Racon on the finished assembly. The number of contigs for *S. cerevisiae* corresponds to the contigs that comprise 99% of the assembly length. In the two *E. coli* datasets, we have no mis-assemblies with respect to the reference. In all the NCTC datasets, HINGE and the NCTC pipeline also agree about the structure of the sequence.

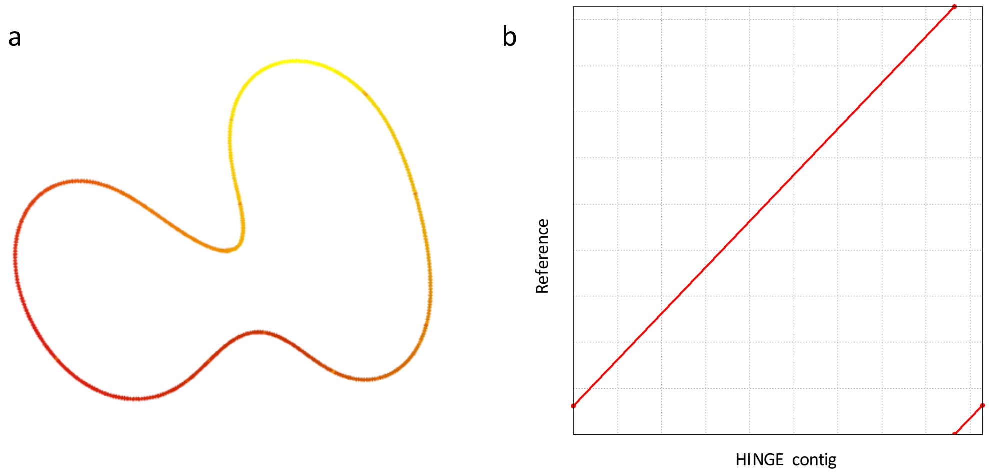

Supplemental Figure S7. Hinge was run on the Oxford Nanopore R9 *E. coli* K-12 MG1655 dataset taken from http://s3.climb.ac.uk/nanopore/R9_Ecoli_K12_MG1655_lambda_MinKNOW_0.51.1.62.all.fasta. The assembly obtained was compared to the reference obtained from http://www.ncbi.nlm.nih.gov/nuccore/545778205?report=fasta. **(a)** We colored the HINGE graph according to the position that the read aligns to in the reference. We note that a lack of sharp color gradients shows the structural integrity of the assembly. Connected components that were smaller than ten nodes (which might correspond to small plasmids or bacterial phages were deleted). **(b)** A dot-plot comparing HINGE assembly and the reference shows that the assembly agrees with the reference on most of the contig.

a

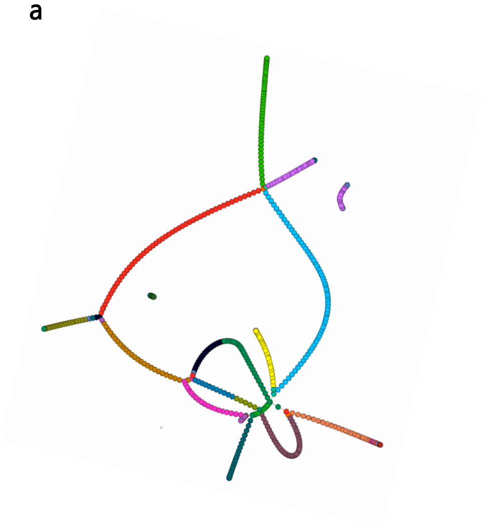

b

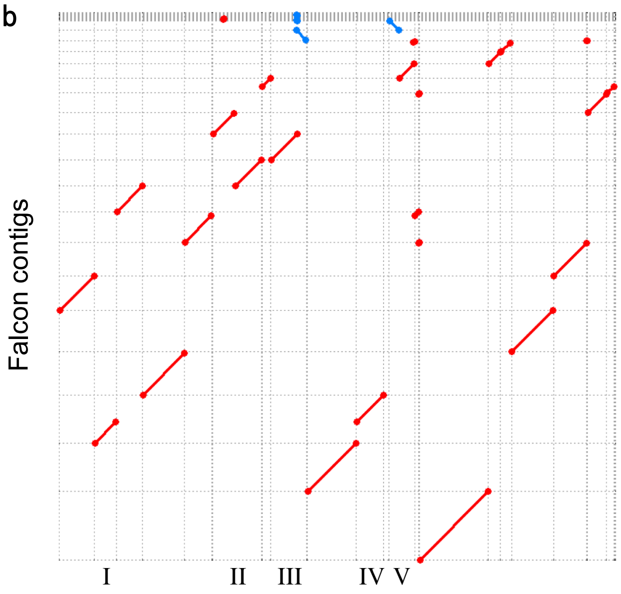

c

Running time	Contigs	Total length (bp)	N50	Identity (%)	Running time (core-hours)	
Falcon	24	12,370,681	777,787	-	137	
HINGE	22	12,401,587	789,778	98.95	Overlap	39.5
					Layout	2
					Consensus	0.33
					**Total**	41.9

Supplemental Figure S8. HINGE was run on a *Saccharomyces cerevisiae W303* PacBio dataset. **(a)** We colored the HINGE graph according to the chromosome where each read maps to (using the Falcon assembly as reference). We notice that in the HINGE graph most of the chromosomes correspond to single paths. Some of these paths are connected at their ends, due to the repetitive nature of the telomeres. **(b)** Dotplot comparing the HINGE and Falcon assemblies. Every row corresponds to a distinct Falcon contig and every column corresponds to a distinct HINGE contig. The diagonal lines represent the alignments between the two assemblies and show that most of the resulting contigs are the same. Some exceptions are HINGE's contigs I and IV (which are merged in Falcon's assembly), and HINGE's contigs II, III, and V (each of which is broken into two contigs by Falcon). **(c)** Comparison of the two assemblies under standard metrics. The number of contigs corresponds to the number of contigs comprising 99% of the total assembly length.

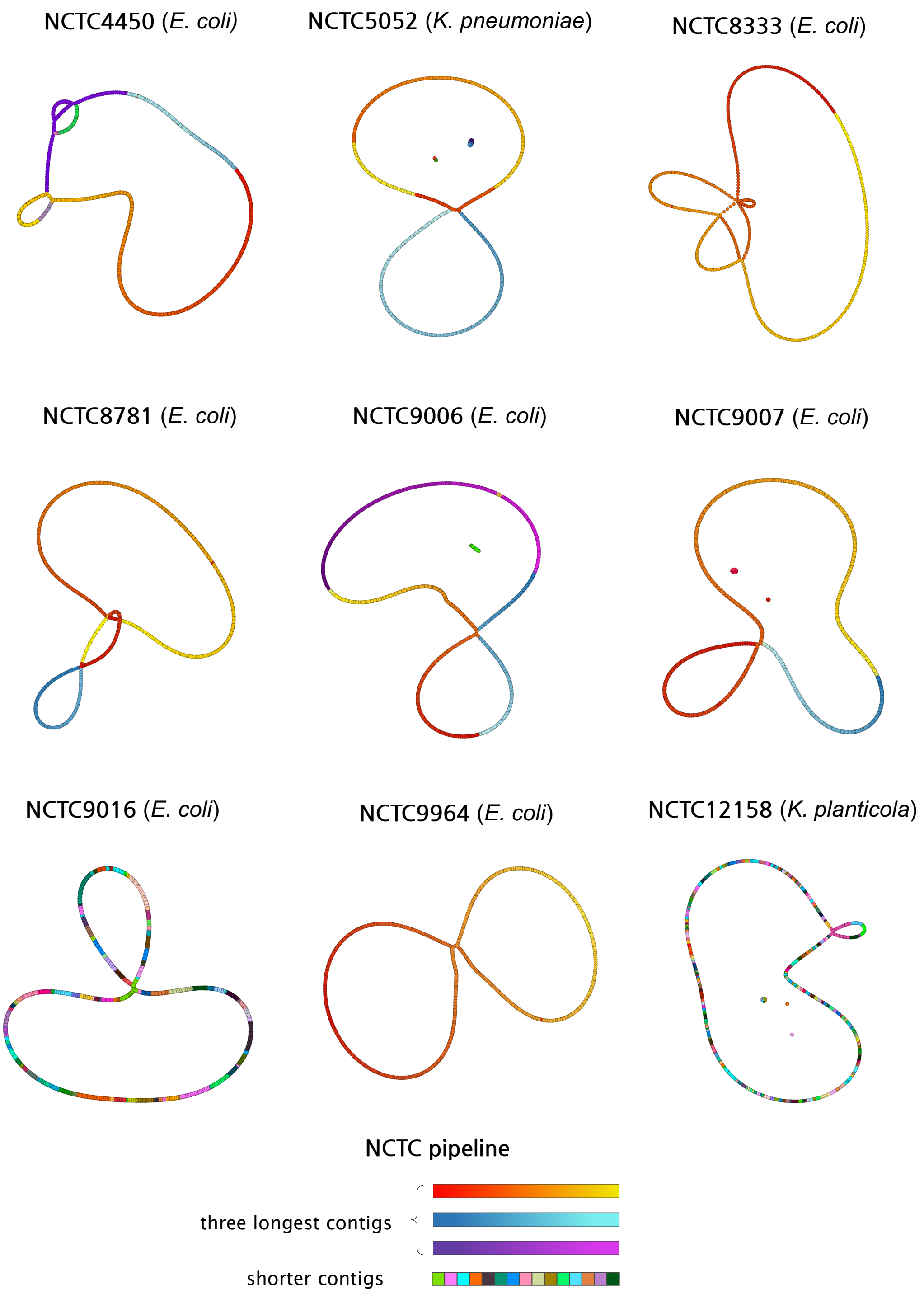

Supplemental Figure S9. Selected examples from the NCTC database where HINGE collapses unbridged repeats. In some cases, such as NCTC5052 and NCTC12158, the repeat admits only one traversal and is resolved in the loop resolution step (not shown here). However, if the graph shows an unbridged inverted repeat (such as in NCTC9006, NCTC9007, NCTC9964, and NCTC9016), or a pair of interleaved repeats (NCTC8781 and NCTC4450), or a more complex repeat pattern (NCTC8333 which has a triple repeat and a pair of interleaved repeats), multiple traversals may be possible, and HINGE produces a graph with the collapsed repeats.

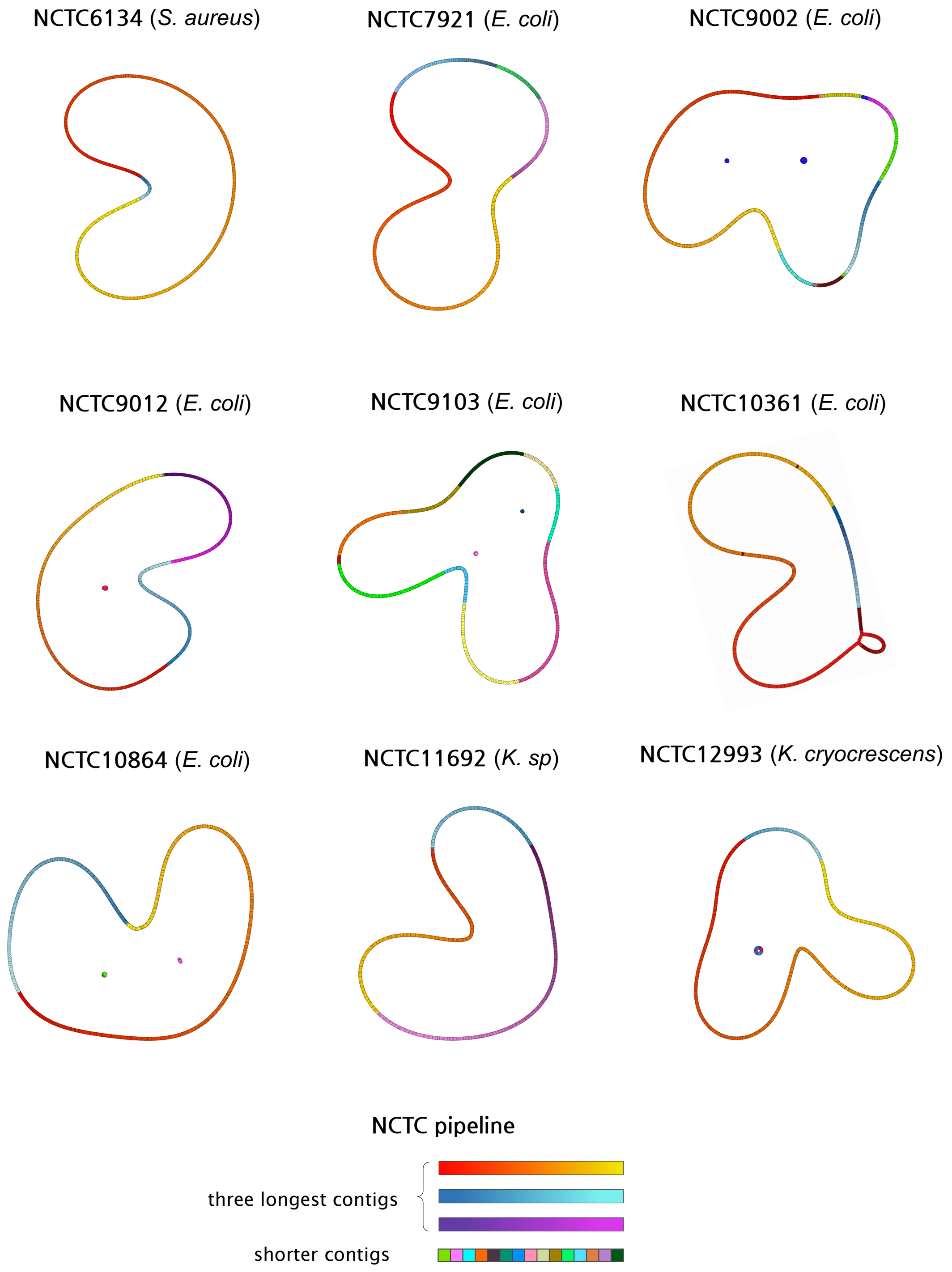

Supplemental Figure S10. Selected examples from the NCTC database where HINGE returns finished assemblies while the NCTC pipeline returns fragmented assemblies. In several of these cases, a repeat that is fundamentally resolvable causes the NCTC pipeline to fragment its assembly. HINGE, on the other hand, identifies the bridging of the repeat, and allows the contigs to be correctly connected.

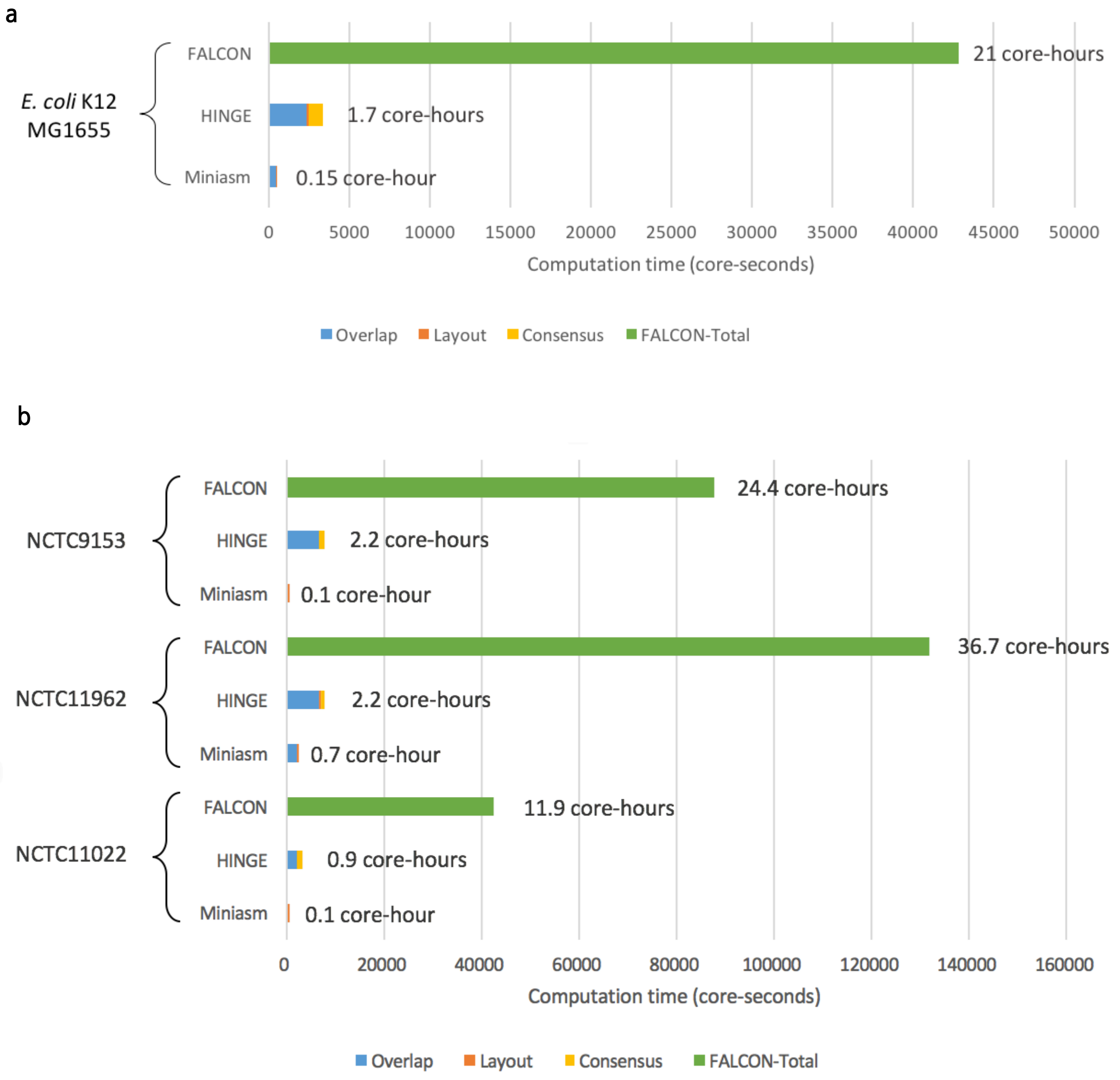

Supplemental Figure S11. Timing analysis of HINGE: Running time of HINGE, FALCON, and Miniasm on (a) the standard *E. coli* dataset of http://www.cbcb.umd.edu/software/PBcR/MHAP/, and (b) NCTC datasets NCTC9153, NCTC11962, NCTC11022. We observe that the overall runtimes of Miniasm, HINGE and FALCON are each separated by roughly one order of magnitude. The majority of HINGE runtime is consumed by the alignment step (DAligner). Miniasm, on the other hand, employs a fast overlapping tool (Minimap), but does not have a consensus step. The Overlap-Layout-Consensus steps in FALCON are not easily separated, so timing information is not broken down into individual steps. We note that HINGE and FALCON both use DAligner for getting alignments and hence HINGE is not faster than FALCON because it uses a faster alignment tool.

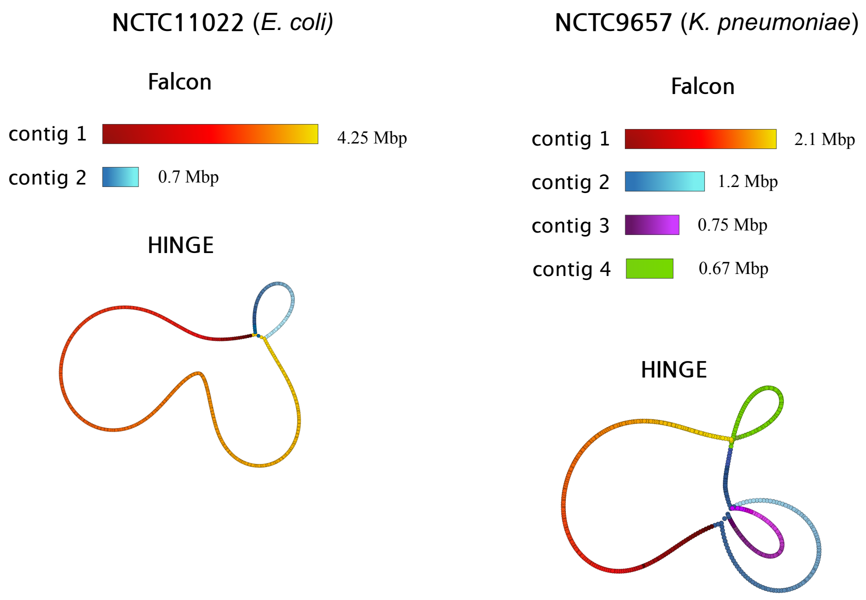

Supplemental Figure S12. HINGE assembly graphs for the NCTC datasets considered in Figure 2, colored according to the contigs produced by FALCON. In both examples, FALCON produces contigs that are similar to those produced by the NCTC pipeline (based on HGAP and Circlator). Specifically, on the NCTC11022 dataset (*Escherichia coli*), FALCON returns two contigs due to an incorrect resolution of an unbridged repeat, while HINGE produces a single large chromosomal contig of length 5 Mbp. The nodes in the HINGE graph are colored according to the position the corresponding reads align to in the FALCON-produced contigs. Running FALCON on the NCTC9657 dataset (*Klebsiella pneumoniae*) returns four large contigs, whereas HINGE's output graph has one large connected component. The large component corresponds to a circular chromosome with an unbridged triple repeat and an unbridged repeat, and is a combination of the three large contigs returned by FALCON. FALCON's second contig implicitly resolves a triple repeat that is fundamentally unresolvable given the reads (see Supplemental Figure S12), hence creating a potential mis-assembly that cannot be detected from the read data. In contrast, HINGE identifies this repeat as unresolvable, and outputs the indicated graph to prevent a mis-assembly.

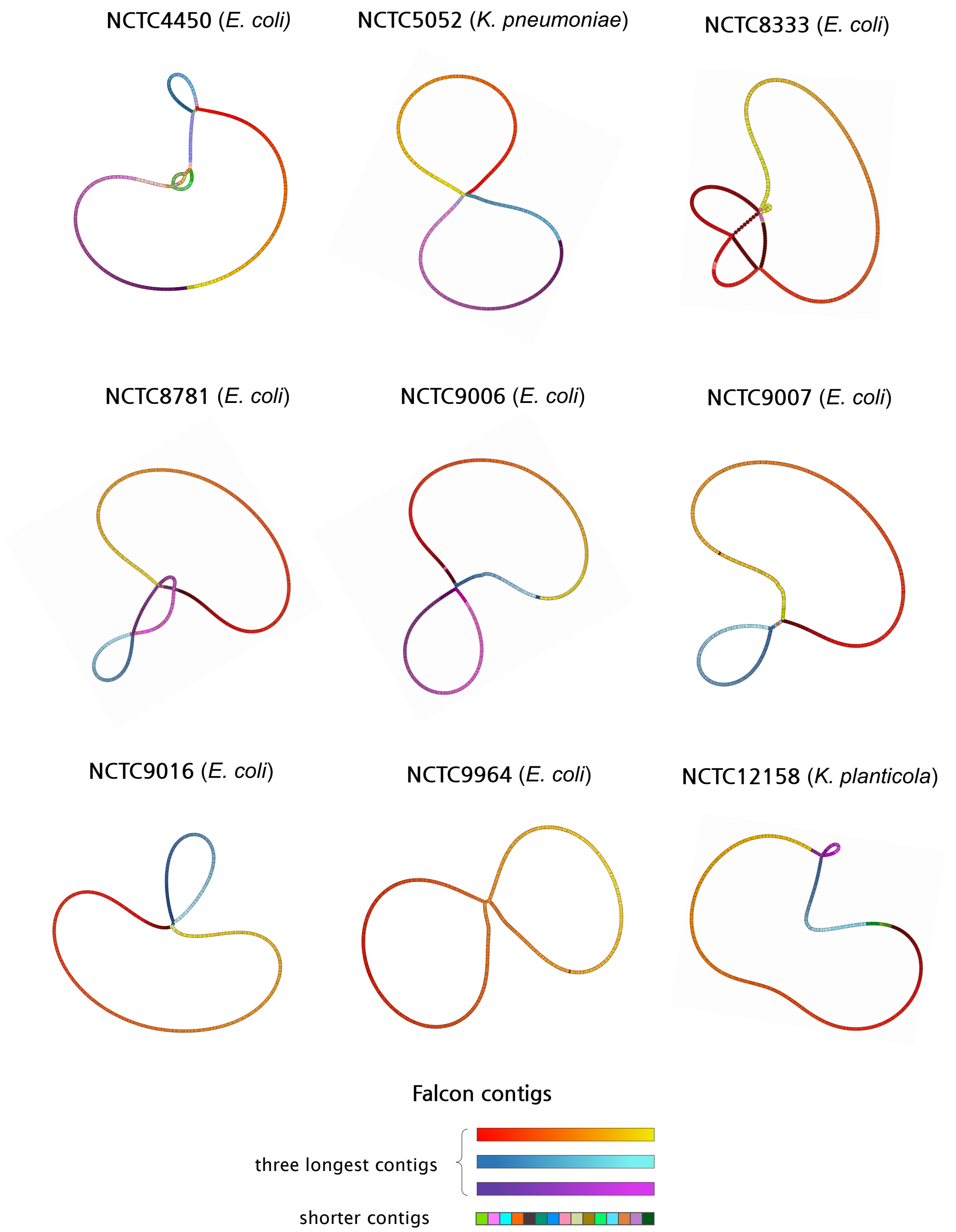

Supplemental Figure S13. HINGE assembly graphs for the same NCTC datasets considered in Supplemental Figure S3, colored according to the contigs produced by FALCON. We notice that the same issues with the output of the NCTC pipeline (based on the HGAP assembler) are observed here. While the assembly graph representation of HINGE allows unbridged repeats to be collapsed, the contigs produced by FALCON instead must break the assembly at these repeats. In addition, we notice that in NCTC4450, NCTC5052, NCTC9006, and NCTC12158, FALCON unnecessarily breaks the assembly at points that do not correspond to unbridged repeats.

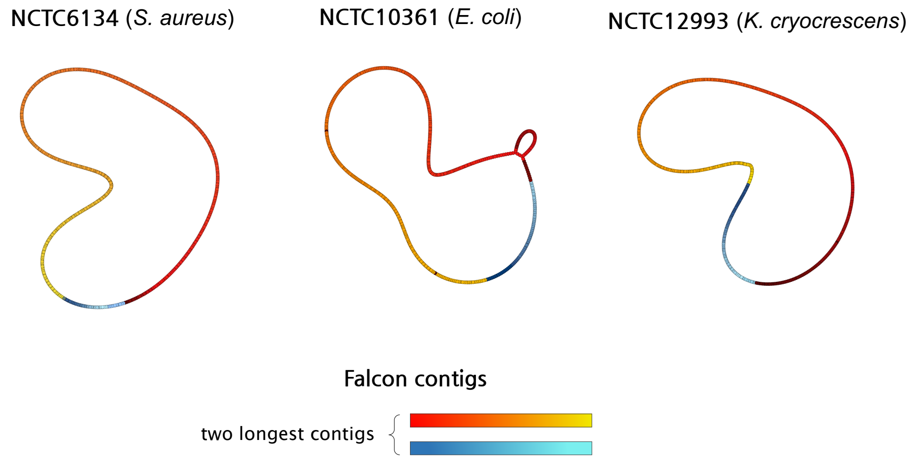

Supplemental Figure S14. We ran FALCON on the nine NCTC datasets considered in Supplemental Figure S4. In six of them (not displayed here), FALCON also achieves a finished single-contig assembly that agrees with the HINGE assembly graph. The HINGE assembly graphs for the three remaining ones are shown here, colored according to the contigs produced by FALCON.

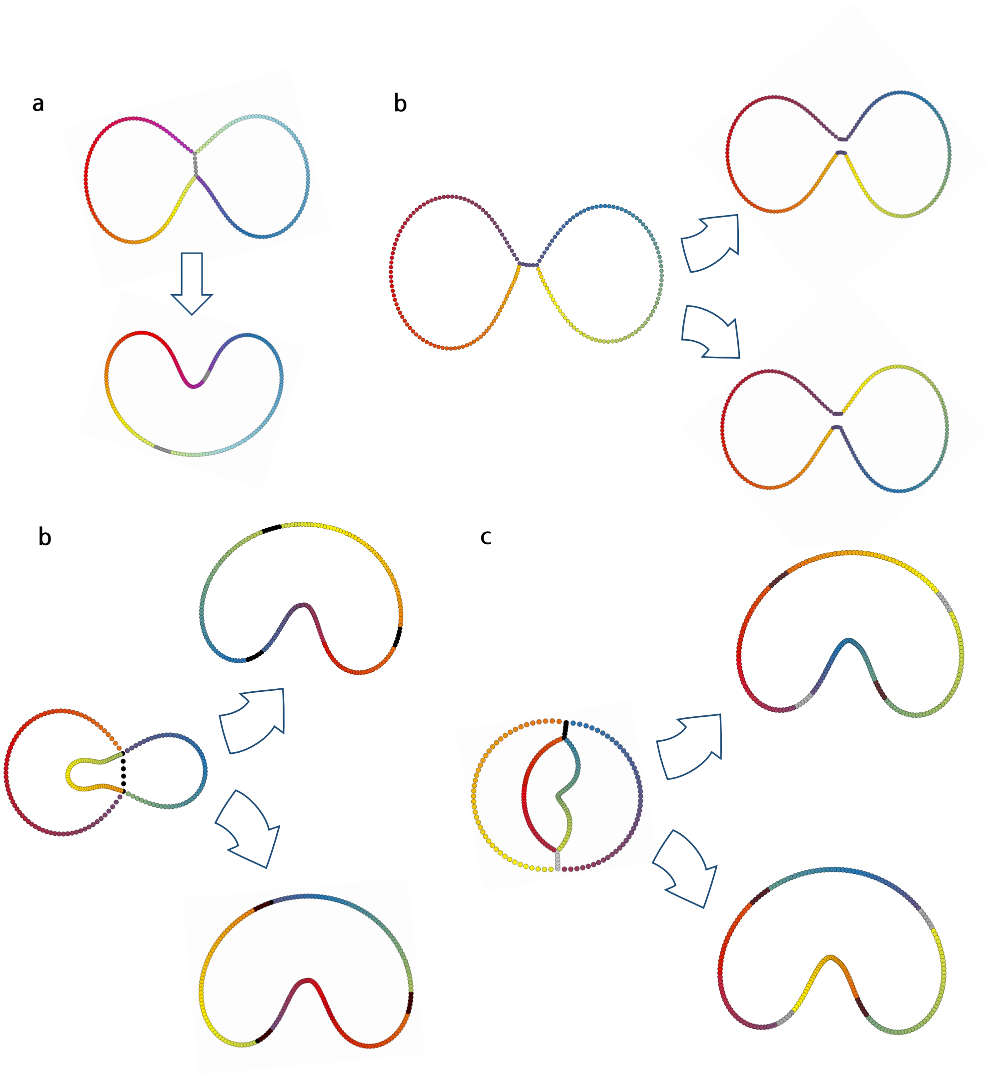

Supplemental Figure S15. Resolvability of various repeats patterns: **(a)** Assembly graph obtained when a (non-inverted) repeat that is not interleaved with any other repeat is unbridged. This repeat can be resolved, as there is only one possible traversal of the graph. **(b)** Assembly graph obtained when there is an unbridged inverted repeat. In this case, there are two possible traversals, and the repeat should not be resolved. **(c)** Assembly graph corresponding to an unbridged triple repeat, and the two traversals consistent with the data. **(d)** Assembly graph corresponding to a pair of unbridged interleaved repeats, and the two traversals consistent with the data.

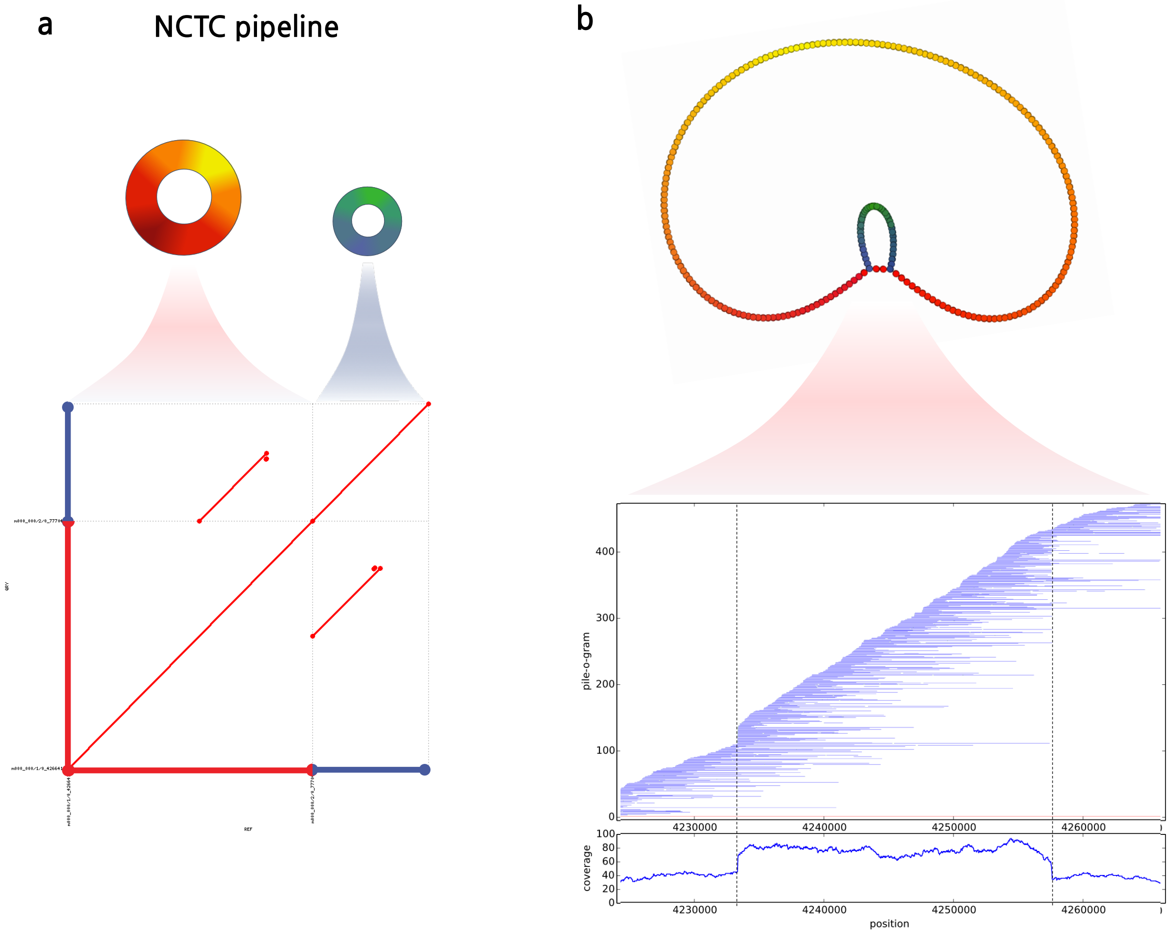

Supplemental Figure S16. Detailed analysis of NCTC11022. **(a)** shows a dot plot of the regions of the two contigs returned by the NCTC pipeline that is collapsed by HINGE as an unbridged repeat. It shows that there is a repeat of length 20 kbp between the contigs. **(b)** shows the pile-o-gram obtained by aligning all reads to the longer contig returned by the NCTC pipeline. We note that there is no read that bridges the repeat.

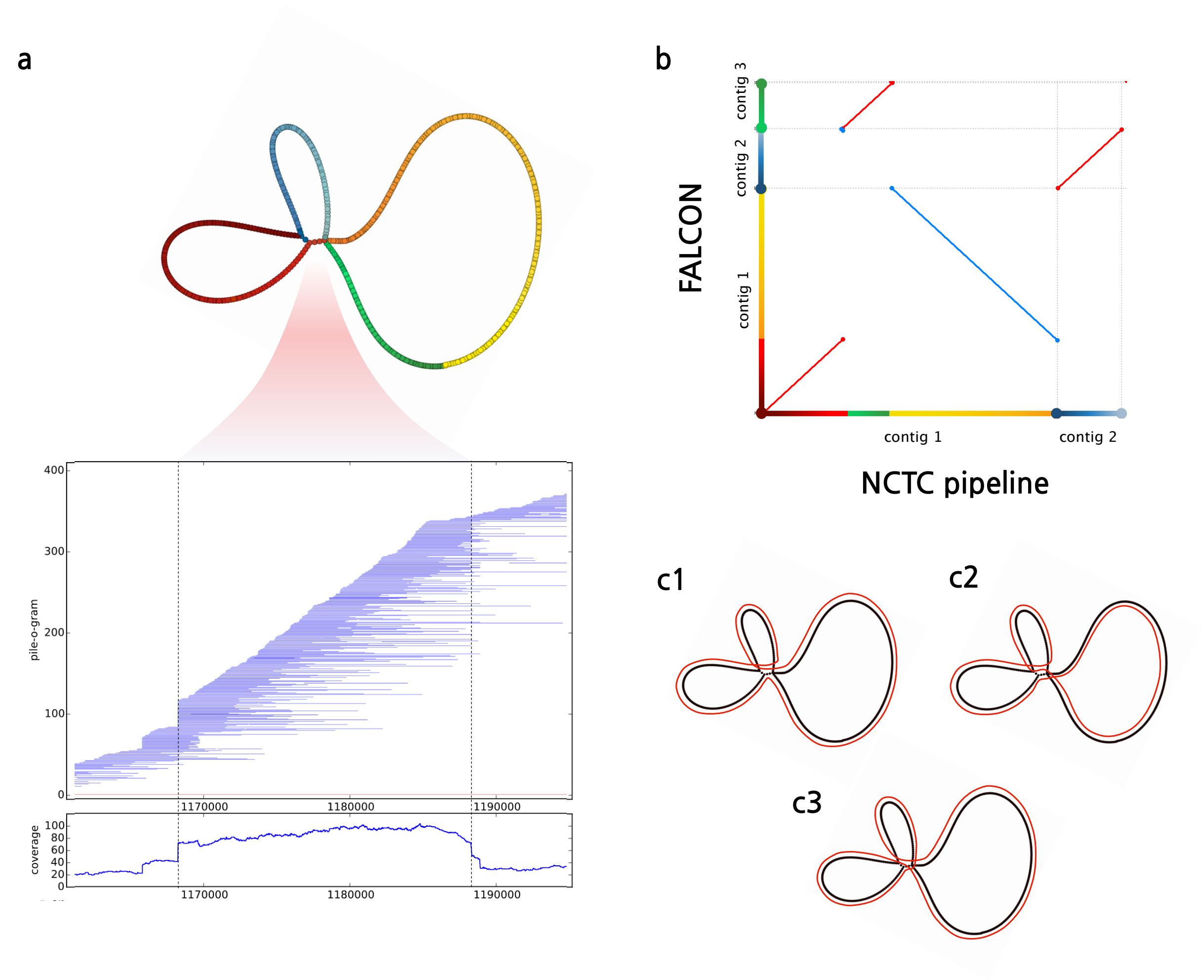

Supplemental Figure S17. Detailed analysis of NCTC9024. **(a)** We show the pile-o-gram obtained by aligning reads of length more than 3 kbp to contig 1 returned by the NCTC pipeline. The coverage depth shows that there is triple repeat of 20 kbp on this segment, and no read bridges it. **(b)** We show a dot plot (obtained with Nucmer) comparing the assemblies produced by the NCTC pipeline and by FALCON. We notice that they attempt to resolve the 20 kbp repeat in distinct ways, leading to large structural differences. **(c)** shows the three distinct traversals of the graph produced by HINGE. Contig 1 of the NCTC pipeline only agrees with traversal c1. Contig 1 of FALCON only agrees with traversal c2. Neither NCTC's contig 1 nor FALCON's contig 1 agrees with traversal c3.

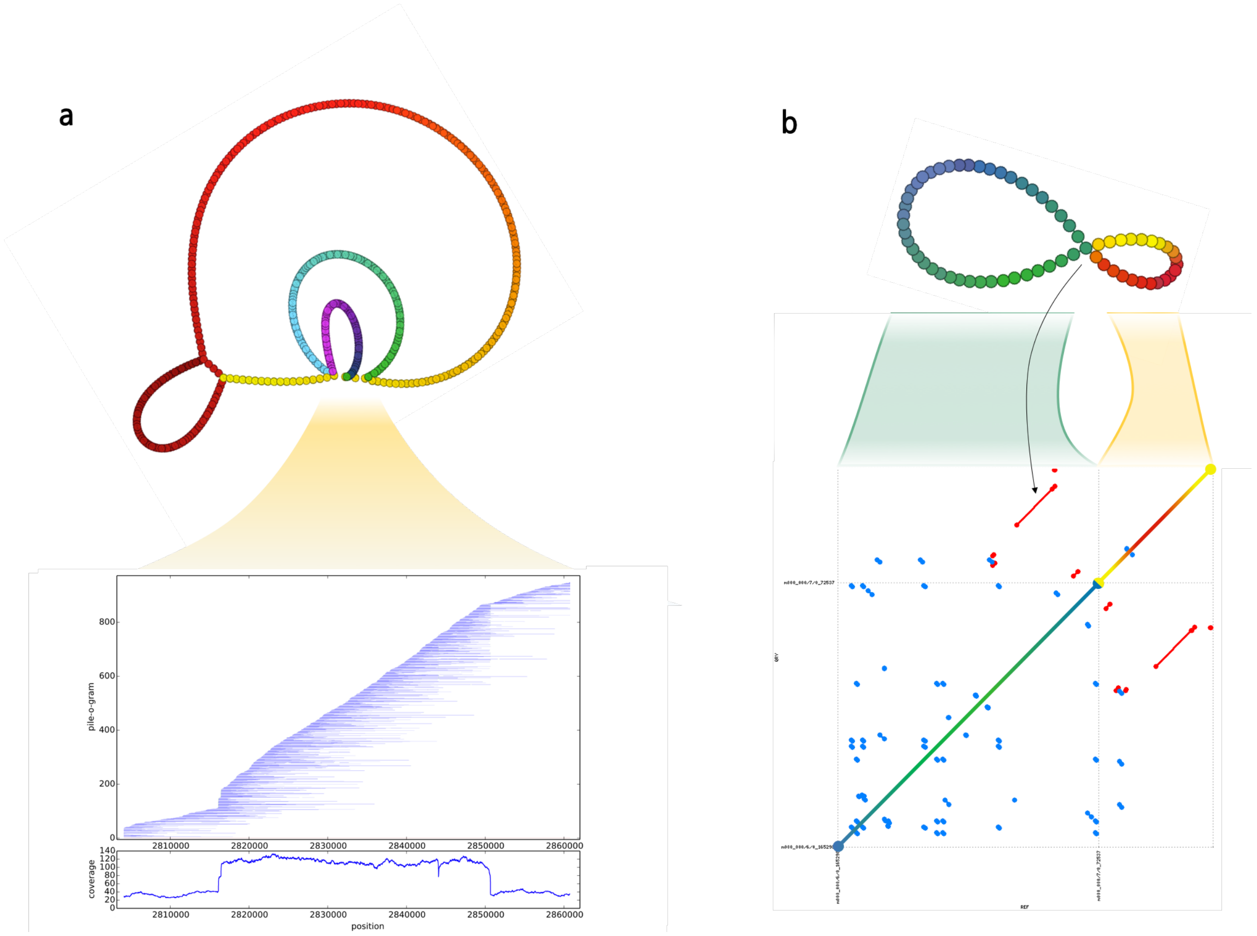

Supplemental Figure S18. Detailed analysis of NCTC9657. (a) shows the assembly graph returned by HINGE in the case of NCTC9657. The alignment of reads of length more than 10 kbp in the region HINGE declares as triple repeat is shown below. Based on the coverage profile, we note that this indeed is a triple repeat. **(b)** shows the dot plot examining the pair of plasmids more closely. It shows that the two plasmids share a repeat of length 22 kbp. HINGE merges this repeat, as it is unbridged.

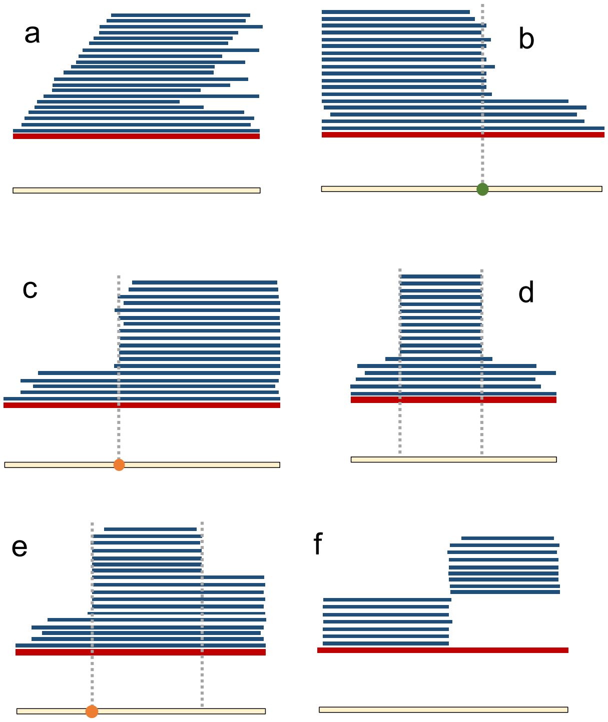

Supplemental Figure S19. Pile-o-gram analysis and repeat annotation: In order to perform the hinge placement, first we annotate starts/ends of repeats on reads by looking for sharp increases/decreases in the number of matches. Then with annotated repeats, we declare a repeat boundary as a hinge if the internal matches starting/ending there do not all end on another repeat boundary. **(a)** shows a normal read with no such sharp change. No repeat is detected here. **(b)** shows a case where we detect first that a repeat ends on the read. We then note that the matches ending in at the repeat margins start mostly at the start of the read (and not a heap in the interior of the read) and place and declare that the read does not bridge the repeat that it covers. **(c)** shows a similar case where we detect the start of a repeat. **(d)** shows a case where we annotate a repeat start and a repeat end, but note that all reads starting at the repeat start end in a repeat end (and vice-versa). Hence we declare that this is a bridging read. Both the locations however are marked as bridged repeat boundaries. **(e)** shows a pile-o-gram resulting from a repeat occurring within another repeat. In this case, we first annotate a repeat start and a repeat end. Then we note that all the reads ending at the repeat end start in a heap at the repeat start. However, the alignments starting at the repeat start do not all end in a heap. Thus we place an unbridged start-repeat annotation. The annotation of the repeat end is marked as bridged**. (f)** shows the pile-o-gram of a chimeric read.

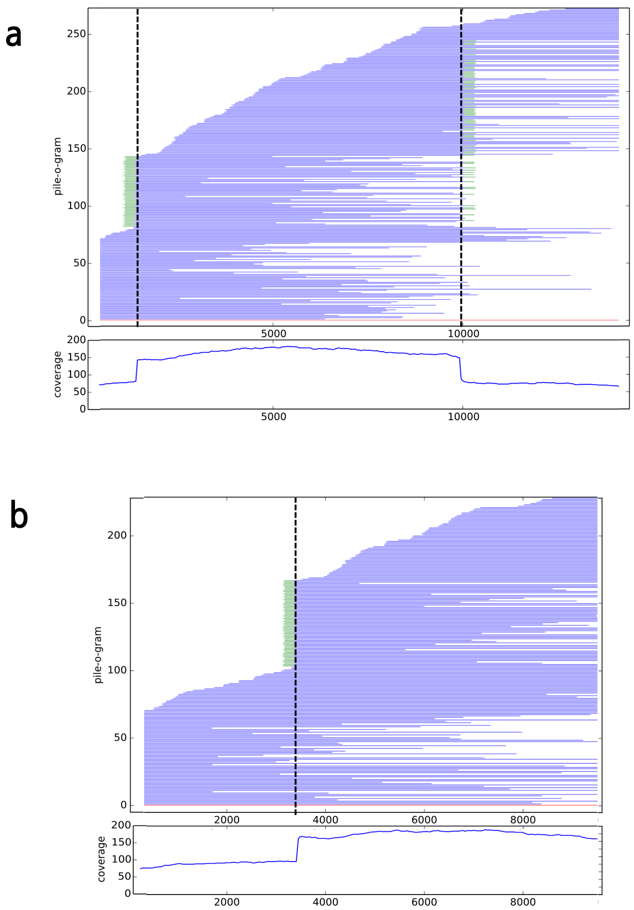

Supplemental Figure S20. Examples of pile-o-grams on real data. **(a)** shows a pile-o-gram of a read with two repeat annotations. Both these are marked as bridged, as almost all internal matches starting at the repeat start end in a pile around the repeat end. **(b)** shows a read with a repeat start annotated. As the internal matches starting here extend to the end of the read, we do not mark this as bridged.
